# Adaptive strategies of Scots pine under shade: Increase in lignin synthesis and ecotypic variation in defense‐related gene expression

**DOI:** 10.1111/ppl.13792

**Published:** 2022-10-17

**Authors:** Sonali Sachin Ranade, George Seipel, András Gorzsás, María Rosario García‐Gil

**Affiliations:** ^1^ Department of Forest Genetics and Plant Physiology Umeå Plant Science Centre, Swedish University of Agricultural Sciences Umeå Sweden; ^2^ Department of Chemistry Umeå University Umeå Sweden

## Abstract

Shade is a stressful condition for plants characterized by low Red:Far‐Red (R:FR) ratio. The northern latitudes in Sweden daily receive more hours of FR‐enriched light (twilight) or shade‐like conditions compared to southern forests during the growing season. Scots pine (*Pinus sylvestris* L.) is a shade‐intolerant species. Yet, it is well adapted to this latitudinal variation in light, which is evident by a northward increase in FR requirement to maintain growth. Shade adversely affects plant growth; it makes the plant weak and, therefore, susceptible to pathogen attack. Lignin is involved in plant protection against pathogen invasion mainly by forming a physical barrier. We studied lignin synthesis and expression of defense‐related genes (growth‐defense trade‐offs) under a low R:FR (shade) ratio in Scots pine. A higher number of immunity/defense‐related genes were up‐regulated in response to shade in northern populations compared to southern ones, which can be viewed as a local adaptation to light quality for optimal growth and survival. Light quality regulates lignin metabolism; light stimulates lignin synthesis, while shade causes a decrease in lignin synthesis in most angiosperms. In contrast, Scots pine shows an increase in lignin synthesis supported by the higher expression of a few key genes in the lignin biosynthetic pathway, a novel finding reported by our study. These findings can be applied to future breeding strategies in forestry to produce disease‐resilient trees.

## 1 INTRODUCTION

Shade or vegetative shade is defined by a decrease in the Red:Far‐Red (R:FR) ratio, where the pigments of the neighboring plants absorb R light while FR light gets reflected (Ballare et al., 1987). Similar to vegetative shade, twilight is also characterized by a low R:FR ratio, which is caused by sunlight scattering in the upper atmosphere (Nilsen, 1985). The northern latitudes in Sweden receive longer daily exposure to FR‐rich light (twilight) compared to the southern latitudes during the growing season, owing to their geographic location (Figure S1).

Tree species that grow, survive, and thrive in the shade are shade‐tolerant, while those that require full sunlight are shade‐intolerant (Grebner et al., 2021). Scots pine (*Pinus sylvestris* L.), one of the most economically important conifers of the Boreal forests, is a shade intolerant species, yet it is adapted to the local light conditions in the northern latitudes characterized by a low R:FR ratio during the growth period. Scots pine shows ecotypic variation in response to twilight, characterized by a northward increase in the requirement of additional FR light to maintain growth (Clapham et al., 2002; Ranade & García‐Gil, 2013), an adaptive strategy for optimal growth and survival. In addition, Scots pine also shows a differential response to variable light intensities of R and FR light (Razzak et al., 2017).

Plant susceptibility to diseases following pathogen attack under shade (Hussain et al., 2019) can be explained by changes in the response pathways to biotic and abiotic factors (Courbier & Pierik, 2019). Lignin plays an important role in plants by providing structural support and protecting the plant by forming a physical (Lee et al., 2019; Malinovsky et al., 2014) and chemical (Xie et al., 2018) barrier against pathogens. Lignin deposition generally occurs when the cell stops growing and is committed to programmed cell death leading to secondary thickening of the cell wall (Rogers & Campbell, 2004). This means the plant stops growing while responding to pathogens through lignification. This process is termed the growth‐defense trade‐off. Lignin biosynthesis is regulated by light quality (Dixon & Barros, 2019; Zhao & Dixon, 2011). A decrease in lignin synthesis under low light or shade is a common phenomenon in most angiosperms that leads to weaker stems and makes the plant more susceptible to diseases/pathogens (Hussain et al., 2019; Wu et al., 2017).

We previously reported that Scots pine responds to twilight (low light intensity or low R:FR) in a different way than angiosperms, although some aspects of shade response appear to be conserved (Ranade et al., 2019). The same study revealed differential regulation of the light signaling pathway and defense‐related genes in response to shade in Scots pine (Ranade et al., 2019); whether this differential gene regulation follows a latitudinal cline is not known. Therefore, in the current work, we examined the latitudinal variation in the expression of defense‐related genes under shade in Scots pine. We also included lignin analysis in this study as lignin forms one of the major components in plant defense. Moreover, the regulation of the lignin biosynthetic process and the defense mechanism at the gene level, particularly in response to light quality, is not very well understood in Scots pine compared to the well‐studied model plants like *Arabidopsis thaliana* (*Arabidopsis*).

## 2 MATERIAL AND METHODS

### 2.1 Seed germination and light conditions

Scots pine seeds were collected from natural populations in Kaunisvaara (67°5′ N) and Lammhult (56°2′ N), referred to as northern and southern pine populations, respectively. Seeds were sampled from unrelated trees at a minimum distance of 50 m from each other to ensure low consanguinity and to capture a representation of the population diversity. The percentage of germination was obtained by germinating soaked seeds on paper discs on a warm bench with controlled humidity and temperature. The percentage of germination was 98% in a batch of 200 seeds (five seeds per tree). Seventy seeds were germinated under two continuous light treatments (Treatment A, SHADE; Treatment B, SUN) as described previously (Ranade et al., 2019) in Percival (LED‐30 Elite series) growth cabinets, at a constant temperature of 22°C on moist vermiculite. Treatment A (SHADE) represented shade‐like conditions containing R and FR light wavelengths only, with an R:FR ratio of 0.2 and total light intensity of 36 μmol m^−2^ s^−1^ (R, 6 μmol m^−2^ s^−1^; FR, 30 μmol m^−2^ s^−1^). Treatment B (SUN) was used as control light treatment, which represented sun‐like conditions containing R and FR light wavelengths only, with an R:FR ratio equal to 1.2 and a total light intensity of 65 μmol m^−2^ s^−1^ (R, 35 μmol m^−2^ s^−1^; FR, 30 μmol m^−2^ s^−1^). We applied only the R and FR light qualities in this experiment, as these are the two main responsible elements that plants use to determine the shade conditions and respond accordingly. In addition, these light conditions were able to trigger the shade responses in Scots pine, as described in our earlier work (Ranade et al., 2019).

### 2.2 Fourier‐transform infrared (FTIR) spectroscopy analysis

Forty seedlings from each light treatment for each species were used for FTIR spectroscopic analyses. Whole seedlings were oven‐dried at 40°C for 48 h and ground finely in a bead mill at 30 Hz, and for 2 min. Five milligrams of the resulting powder was mixed and manually ground with 395 mg of potassium bromide (KBr, Sigma–Aldrich, FTIR spectroscopy grade) using an agate mortar and pestle. FTIR spectra were recorded in diffuse reflectance mode under vacuum (4 mbar) conditions, using a Bruker IFS 66v/S spectrometer (Bruker Optik GmbH), at a spectral resolution of 4 cm^−1^ according to the protocol described by Gorzsas & Sundberg, 2014 (Gorzsas & Sundberg, 2014). Pure KBr was used as background. Data in the spectral region 400–1900 cm^−1^ were used in the subsequent multivariate analyses as described previously (Gorzsas et al., 2011). Before multivariate analysis, spectra were baseline corrected and standardized using the built‐in 64‐point rubber band baseline correction followed by offset‐ and vector‐normalizations of the OPUS software (version 7.0.122; Bruker Optik GmbH) over the 400–1900 cm^−1^ spectral region. Multivariate analysis was performed by the SIMCA‐P software package (version 11.0.0.0, Umetrics AB). Individual OPLS‐DA (orthogonal projections to latent structures—discriminant analysis) was carried out to highlight specific differences in the chemical composition of the samples. Q2(cum) values were analyzed where Q2 is the fraction of the total variation that can be predicted by a component, as estimated by cross‐validation. Q2(cum) is the cumulative Q2 for all components, that is, a numerical measure of the predictive ability of the model, with a maximum value of 1.0 corresponding to maximum (100%) predictive ability. Q2(cum) is dependent on the number of components; it cannot reliably be used to compare models with different numbers of components. In addition, models with large differences between R2 and Q2 values (i.e., model fit and predictive ability) should be treated as unreliable. When comparing reliable models with matching numbers of components and a similar number of observations, Q2(cum) values can be used to compare the predictive ability of these models.

### 2.3 Transcriptomic analysis

Whole seedlings were used for the extraction of RNA. Three biological replicates were prepared for each light treatment by pooling three seedlings per sample to reduce variation between replicates and increase the statistical power. Isolation of total RNA, RNA sequencing (RNA‐Seq), and pre‐processing of RNA‐Seq data were carried out as described in our previous work (Ranade et al., 2019). In short, total RNA was isolated using Spectrum Plant Total RNA Kit (Sigma) following the manufacturer's instructions. RNA library preparation and subsequent sequencing (HiSeq 2500, Illumina) were performed at SciLifeLab (Stockholm). The data pre‐processing was performed as described here: https://www.epigenesys.eu/en/protocols/bio‐informatics/1283‐guidelines‐for‐rna‐seq‐data‐analysis. Scots pine reads were aligned to the v1.01 of the *Pinus taeda* genome (Zimin et al., 2014), and its annotation was retrieved from http://pinegenome.org/pinerefseq/. The RNA‐Seq data were deposited to the ENA and are accessible under the accession number PRJEB19683 (https://www.ebi.ac.uk/ena/data/view/PRJEB19683). Statistical analysis of single‐gene differential expression within and between the two latitudes in response to SHADE was determined using SUN as control. Analysis was performed using the Bioconductor (v3.3) (Gentleman et al., 2004) and DESeq2 package (v1.12.0) (Love et al., 2014). FDR‐adjusted *P‐*values were used to assess significance; a common threshold of 5% was used throughout. For the data quality assessment (QA) and visualization, the read counts were normalized using a variance stabilizing transformation implemented in DESeq2. Differential regulation of genes expressed under SHADE was determined in the populations at the two latitudes separately, referred to as “within latitude comparison (within southern latitude population and within northern latitude population),” where SUN condition was used as the control. Comparative analysis of the differentially regulated genes under SHADE (where SUN was used as the control condition) between the northern and southern populations was carried out, termed as “north versus south comparison.” North versus south comparison was performed using DESeq using a two‐factor design: design = ~treatment + latitude + treatment × latitude. Genes were categorized according to their GO categories. Pie charts for functional categorization by annotation (GO Biological Process, GO Cellular Component and GO Molecular Function) for the differentially expressed genes (DEGs) for respective treatments and comparisons are included in the Supporting Information. The percentage of annotation was calculated as the number of annotations to terms in the GOslim category × 100/number of total annotations to terms in the ontology. GO enrichment analysis of the top 30 pathways with a *P‐*value cut‐off of 0.05 (FDR) was done using ShinyGO (Ge et al., 2020) using the Arabidopsis database.

RT‐qPCR was not performed to validate the expression data as this study includes robust statistical analysis with the RNA‐Seq data (Coenye, 2021). Further, the enhanced lignin synthesis under SHADE is strongly evident from the FTIR analysis.

## 3 RESULTS

### 3.1 GO annotations and GO enrichment

As our previous study revealed differential regulation in light signaling pathway genes and defense‐related genes in response to shade in Scots pine (Ranade et al., 2019), the current analysis focuses on the latitudinal variation under shade. The details of the overall DEGs, including the within and between latitude comparisons, have been included in the Supporting Information (Tables S1–S3, Figure S2). In general, the percentage of GO annotations was similar across all the comparisons, although the number of DEGs involved in the response to SHADE was diverse (Figures S3–S20). GO enrichment analysis is included as Supporting Information (Figures S21–S34).

**FIGURE 1:**
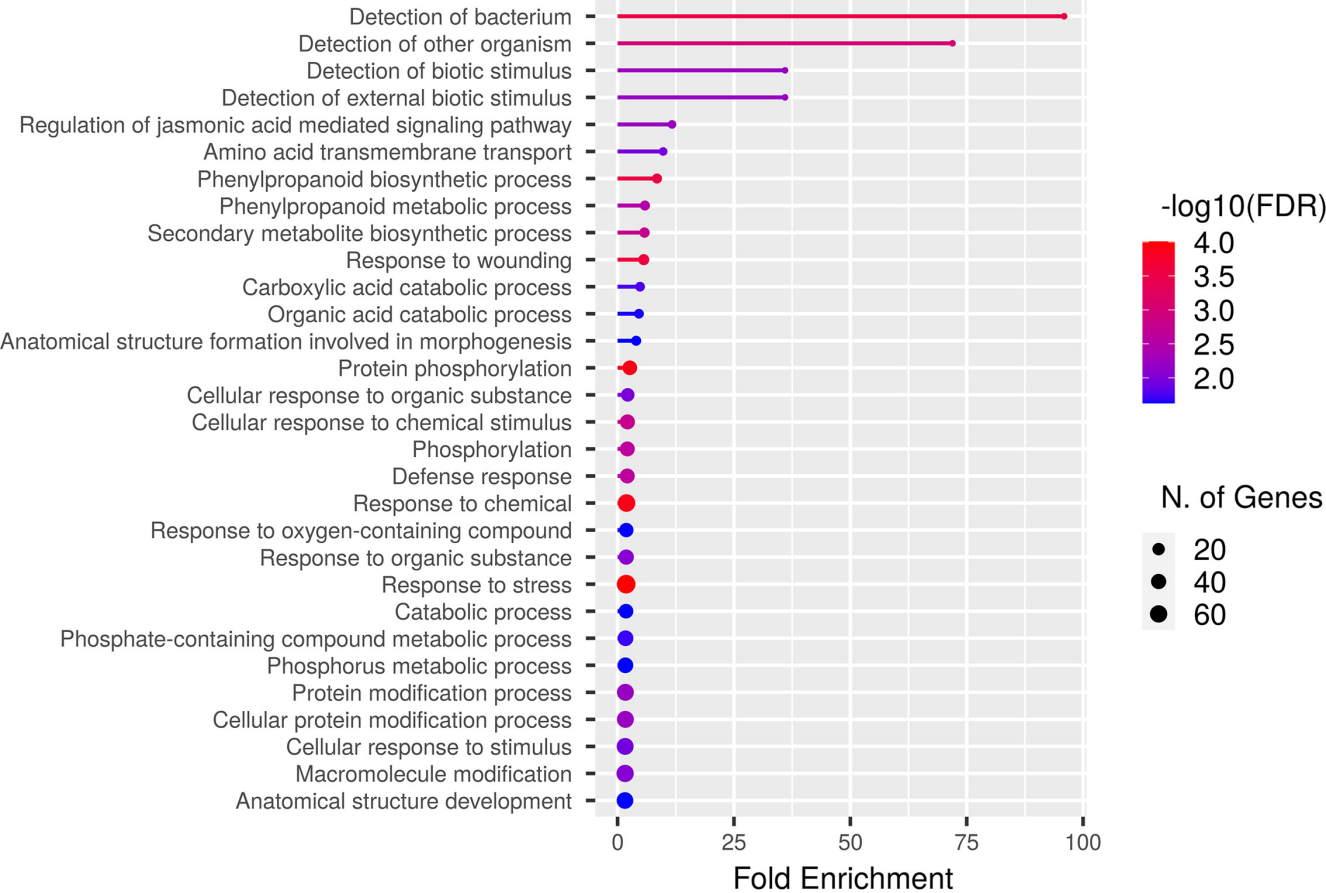
Gene ontology enrichment analysis of top 30 pathways for the biological process of the differentially regulated genes from the north versus south comparison in scots pine populations (*P*‐value cut‐off [FDR]: 0.05). Genes represented have higher expression under SHADE in the northern population as compared to the southern population.

**FIGURE 2:**
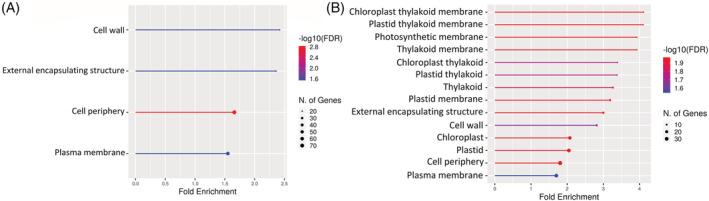
Gene ontology enrichment analysis of top 30 pathways for cellular component for the differentially regulated genes from the north versus south comparison in scots pine populations (*P*‐value cut‐off [FDR]: 0.05). **(**A**)** Genes represented have higher expression under SHADE in the northern population as compared to the southern population. **(**B**)** Genes represented have higher expression under SHADE in the southern population as compared to the northern population.

GO Biological Process and GO Cellular Component enrichment shows enrichment of a higher number of active processes, including photosynthesis and higher enrichment of cellular activity, respectively, under SUN for both within latitude comparisons (Figures S21–S24 and S27–S30). This is in accordance with the shade‐intolerant nature of Scots pine, where under SHADE, energy is channelized for reaching the sunlight. The north versus south comparison indicates an over‐representation of phenylpropanoid pathway genes and higher fold enrichment of defense‐related genes under SHADE in the northern populations (Figure 1). Simultaneously, a lower number of active cellular components under SHADE in the northern latitude (Figure 2) may indicate growth‐defense trade‐offs. There was no significant GO enrichment detected for GO Biological Process for genes having higher expression under SHADE in the southern population compared to the northern population for the north versus south latitude comparison.

### 3.2 Latitudinal variation in DEGs involved in the light signaling pathway in response to SHADE

Light signaling pathway genes associated with shade tolerance in Scots pine are identified (Ranade et al., 2019), but whether their differential regulation follows a latitudinal cline is yet not known. Therefore, in the current work, we investigated latitudinal variation in DEGs involved in the light signaling pathways. Genes distinctly involved in the light pathways, for example, the genes encoding the light‐harvesting chlorophyll‐binding proteins (LHC) and the photosystem I and II (PSI and PSII) (Vasilev & Bruce, 2004), showed latitudinal variation in their expression in Scots pine. ONE‐HELIX PROTEIN 2 (*OHP2*) (closely related to helix I of LHC protein from PSI, *LHCA4*), LIGHT‐HARVESTING COMPLEX B7 (*LHCB7*), and PHOTOSYSTEM II REACTION CENTER PSB28 PROTEIN (*PSB28*) were down‐regulated in the northern Scots pine population as compared to the southern ones in response to SHADE. EARLY LIGHT‐INDUCIBLE PROTEIN 2 (*ELIP2*), constitutive expression of which causes reduction of the pigment content of the chloroplasts (Tzvetkova‐Chevolleau et al., 2007), was up‐regulated in the northern Scots pine population as compared to the south. PHYTOCHROME A SIGNAL TRANSDUCTION 1 (*PAT1*) that positively regulates PHYTOCHROME A (*PHYA*) signal transduction in response to light (Bolle et al., 2000), was up‐regulated in the northern Scots pine population as compared to the south.

### 3.3 Latitudinal variation for defense‐related DEGs in response to SHADE

Genes involved in immunity or the defense mechanisms were analyzed for differential expression in response to SHADE. For this purpose, the genes categorized under defense response in the gene ontology (GO) were considered (e.g., GO:0042742, GO:0002229, and GO:0050832). The within latitude comparisons revealed a significantly higher number of immunity/defense‐related DEGs down‐regulated under SHADE in the northern and southern populations, respectively (*P* < 0.05). Seventy‐four defense‐related genes were up‐regulated, and 154 defense‐related genes were down‐regulated under SHADE in the southern population, while 74 defense‐related genes were up‐regulated and 114 defense‐related genes were down‐regulated under SHADE in the northern population. This finding agrees with the shade‐intolerant nature of Scots pine (Ranade et al., 2019). Interestingly, the north versus south comparisons showed a significantly higher number of immunity/defense‐related genes being up‐regulated in the northern population under SHADE (*P* < 0.05). Fifty‐six defense‐related genes were up‐regulated under SHADE in the northern population, while 28 were up‐regulated in the southern population. The higher fold enrichment of defense‐related genes under SHADE in the northern populations (Figure 1) further supports these results.

### 3.4 Effect of SHADE on lignin content

**FIGURE 3:**
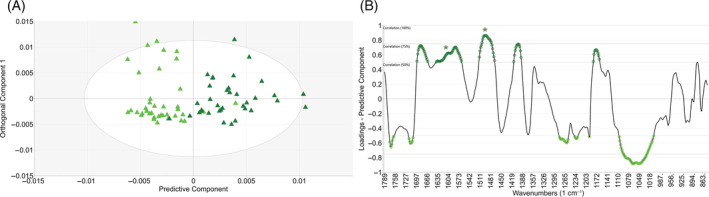
(A) OPLS‐DA scores plots of FTIR spectra from northern scots pine samples grown under SUN (light green) and SHADE (dark green) conditions. Each symbol represents one sample. (B) The corresponding correlation scaled Loadings plot for the predictive component. Bands more intense in SUN‐ and SHADE‐grown trees are marked by light and dark green, respectively. Only bands with more than 50% correlation are marked with their respective colors. Asterisks denote aromatic —C=C— bands, associated with lignin.

**FIGURE 4:**
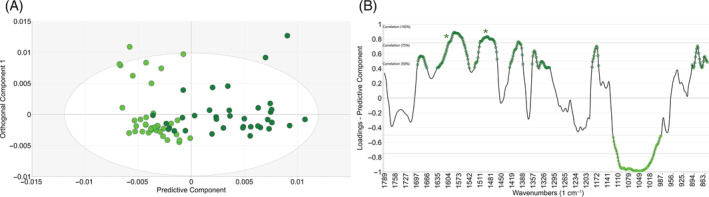
(A) OPLS‐DA scores plots of FTIR spectra from southern scots pine samples grown under SUN (light green) and SHADE (dark green) conditions. Each symbol represents one sample. (B) The corresponding correlation scaled Loadings plot for the predictive component. Bands more intense in SUN‐ and SHADE‐grown trees are marked by light and dark green, respectively. Only bands with more than 50% correlation are marked with their respective colors. Asterisks denote aromatic —C=C— bands, associated with lignin.

FTIR spectroscopy is based on molecular vibrations and primarily informs about the functional groups of the chemical compounds present in the sample. Since each IR active compound has unique FTIR spectra, FTIR spectroscopy is an excellent chemical fingerprinting tool, with the recorded spectra reflecting the entire chemical matrix of the sample, in situ (without the need for extraction) and without external agents (dyes, labels, markers). To highlight specific differences, individual OPLS‐DA (orthogonal projections to latent structures—discriminant analysis) models were created, facilitating interpretation and direct comparisons. Table 1 summarizes these models, and Figures 3 and 4 give a graphical overview. While the recorded FTIR spectra contain information about the entire chemical fingerprint of these trees, we focus only on the lignin‐related changes (marked by asterisks in the loadings plots. All bands with a higher than 50% correlation are marked in the loadings plots, using the colors of their classes, e.g., dark green for SHADE grown Scots pines.). Higher lignin synthesis was detected under SHADE conditions compared to the SUN treatments in Scots pine at both latitudes (Figures 3 and 4). In particular, Scots pine trees at both locations deposited considerably more carbohydrates in their cell walls under SUN conditions than in SHADE (light green marked bands between 1000 and 1100 cm^−1^). However, both lignin‐related spectral bands (aromatic —C=C— vibrations at ca. 1508 and 1595 cm^−1^, marked by asterisks in all Loadings plots) show consistently higher intensity for SHADE‐grown trees at both locations. The predictive ability of the models (Q2(cum) values in Table 1) appears similar, indicating relative differences in the chemical composition between SUN‐SHADE conditions. The Q2(cum) values are virtually identical for both south and north models. In most flowering plants, light promotes lignin synthesis, whereas shade reduces lignin synthesis. In contrast, FTIR data showed enhanced lignin synthesis under shade in Scots pine.

**TABLE 1:** OPLS‐DA analyses of FTIR spectra, using 1 + 2 (predictive + orthogonal) components for easy comparison

Comparison	No. samples (*N*)	R2X(cum)	R2Y(cum)	Q2(cum)	Figure representing the model
SUN versus SHADE, North	76	0.861	0.662	0.59	Figure 3
SUN versus SHADE, South	73	0.874	0.624	0.545	Figure 4

*Note*: Each model lists the number of samples included, together with the cumulative R2X, R2Y, and Q2 values. Higher Q2(cum) values generally mean stronger predictive abilities, that is, larger differences in the FTIR spectra and thereby in chemical composition.

### 3.5 DEGs involved in lignin biosynthetic process

Genes specific to lignin synthesis were analyzed for differential expression in response to SHADE. For this purpose, the genes categorized under GO:0009809 were considered. Few of the key genes specific to the lignin biosynthetic process (Herrero et al., 2013; Liu et al., 2018) that were up‐regulated under SHADE include *CINNAMYL ALCOHOL DEHYDROGENASE (CAD6, CAD9), CAFFEOYL COENZYME A O‐METHYLTRANSFERASE (CCoAOMT), HYDROXYCINNAMOYL‐COA SHIKIMATE/QUINATE HYDROXYCINNAMOYL TRANSFERASE (HCT)*, and *PEROXIDASE 52 (PRX52)*.

The transcriptomic analysis of key genes in the lignin biosynthesis pathway did not reveal a significantly higher number of lignin genes that were up‐regulated under SHADE in the northern and southern Scots pine populations. However, the FTIR data supports an increased lignin content in response to SHADE in both populations. Regarding suppressors of the lignin biosynthesis pathway, more than one copy/homolog of MYB3/MYB4 repressors were found to be differentially regulated under SHADE in the comparisons; some homologs were up‐regulated while some were down‐regulated, where down‐regulation of the repressor indicates up‐regulation of lignin pathway and vice versa (Behr et al., 2019; Ma & Constabel, 2019; Xiao et al., 2021). MYB3 and MYB4 down‐regulates *CINNAMATE 4‐HYDROXYLASE* (*C4H*); MYB4 also targets *CAD* and *CINNAMOYL COA REDUCTASE* (*CCR*) to suppress them, which leads to the suppression of the lignin synthesis pathway (Xiao et al., 2021).

In the southern Scots pine population, for the within latitude comparison, a significantly equal number (*P* < 0.05) of *MYB3*/*MYB4* copies were up/down‐regulated under SHADE—one copy of *MYB3* and three copies of *MYB4* were up‐regulated, while five copies of *MYB3* and one copy of *MYB4* were down‐regulated along with up‐regulation of one homolog each of *CCoAOMT* and *PRX52* under SHADE. Likewise, a significantly equal number (*P* < 0.05) of *MYB3* and *MYB4* copies were up/down‐regulated under SHADE conditions in the northern Scots pine population for the within latitude comparison (one copy of *MYB3* and five copies of *MYB4* were up‐regulated, while two copies of *MYB3* were down‐regulated under SHADE). In addition, four lignin‐specific genes were up‐regulated under SHADE—*HCT*, *PRX52*, *CAD9*, and *CAD6*. For the north versus south comparison, three copies of *MYBs* (two copies of *MYB3* and one copy of *MYB4*) were up‐regulated, while one copy of *MYB3* was down‐regulated along with the up‐regulation of lignin‐specific genes *PRX52* and *CAD9* under SHADE in the north as compared to the southern population.

## 4 DISCUSSION

Based on the north versus south ecotypic variation in DEGs in the light pathway, we hypothesize that northern Scots pine populations have reduced photosynthetic activity as an adaptive response to the extended twilight conditions. Because Scots pine is shade‐intolerant, this is consistent with the shade avoidance syndrome (SAS) (Ranade et al., 2019). Growth in FR‐enriched environments requires the action of PHYA, for example, in the shade‐intolerant species *Arabidopsis*; PHYA is localized to the cytosol under dark and accumulates in the nucleus in response to irradiation with FR (Bae & Choi, 2008; Wang et al., 2020). Although *PHYA* was not detected to be differentially regulated in Scots pine under SHADE, the up‐regulation of *PAT1* in the northern population under SHADE suggests enhanced positive regulation of PHYA signal transduction in the north (Bolle et al., 2000) compared to the southern population.

Northern forests in Sweden are less productive than southern forests due to harsh climatic conditions and poor soil quality, but they are more disease resistant; southern trees, while fast‐growing, have lower survival rates (Andersson et al., 2003). Overall, this phenomenon could be accounted for by the so‐called growth‐defense trade‐offs (Xie et al., 2018). Regarding disease resilience, yet another study reported that the northern populations of Scots pine showed the strongest resistance to *Gremmeniella abietina* (fungus) attacks (Hansson, 1998). In the current study, transcriptomic analysis performed separately in the southern and northern populations of Scots pine revealed a significantly higher number of defense‐related genes down‐regulated under SHADE in both populations, supporting Scots pine's overall shade‐intolerant nature (Ranade et al., 2019). Surprisingly, a higher number of defense‐related genes were found to be up‐regulated in the northern population in response to SHADE (low R:FR ratio or twilight) in the north versus south comparison. This suggests that, while FR‐enriched light is a stressful condition to both Scots pine populations, the southern population's defense mechanism suffers the most.

The north versus south comparison shows a lower number of active cellular components under SHADE in the northern latitude compared to the southern ones (Figure 2). Simultaneously, the comparison indicates an over‐representation of phenylpropanoid pathway genes and higher fold enrichment of defense‐related genes under SHADE in the northern populations (Figure 1), thus displaying the growth‐defense trade‐offs. This indicates that as Scots pine is shade‐intolerant, the northern trees are trying to optimize growth and development under the higher FR exposure by adapting to the local light conditions while keeping its other activities minimal. The GO enrichment was done using the Arabidopsis database. With a few exceptions, the lignin pathway and defense response pathways are not as widely researched in conifers as they are in model systems such as *Arabidopsis* due to constraints in finding the mutants and creation of transgenic lines in conifers. However, a few studies conducted with reference to the lignin synthesis and defense response in conifers indicate that the pathways appear to be well conserved with the flowering plants with some variations (Kolosova & Bohlmann, 2012; Kovalchuk et al., 2015; Lim et al., 2021; Pascual et al., 2016). For example, conifers lack the FERULATE 5‐HYDROXYLASE (F5H) gene, which is essential for the synthesis of S‐type lignin, but the transformation with F5H was sufficient for the production of S‐type lignin in the pine species *Pinus radiata* (Wagner et al., 2015).

We also observed a higher lignin content under SHADE in both populations, which can be interpreted as an adaptation to defend itself from pathogen attack. We propose that higher levels of lignin detected by the FTIR results under SHADE in Scots pine were observed due to the lower expression of *MYB3*/*MYB4* coupled with the higher expression of some lignin synthesis‐specific genes. However, further experimental validation is required to establish the association of the expressed genes with the elevated lignin levels. As the MYB3/MYB4 transcription factors act as lignin pathway suppressors (Behr et al., 2019; Ma & Constabel, 2019; Xiao et al., 2021), their down‐regulation along with higher expression of a few key lignin‐specific genes under SHADE leads to higher lignin synthesis observed in this study. However, a few other lignin pathway genes were also found to be down‐regulated under SHADE coupled with up‐regulation of certain other *MYB3*/*MYB4* homologs, yet the higher lignin under SHADE is strongly evident from the FTIR data. The lignin pathway in Scots Pine may involve a complex network of feedback loops or alternative pathways, and there may be variation in the binding capacity of the different MYB family members or interaction/competition between them, in addition to post‐translational modifications in response to SHADE that requires further investigation. Similar reasons were discussed for the abundance of grass MYB4 homologs bound to the promoter of lignin biosynthetic genes, which were not correlated with the expression level of the *MYB4* genes (Agarwal et al., 2016; Miyamoto et al., 2020). A recent study in *Ginkgo biloba* reports that its *MYB4* homolog (*GbMYBR1*) differs from MYB4‐type repressor genes in *Arabidopsis*, as *GbMYBR1* lacks the key repressor motifs (EAR/TLLLFR) in the C‐terminal region of the gene but still functions as a repressor of lignin pathway (Su et al., 2020). This suggests that gymnosperms may have alternative mechanisms which differ from the flowering plants. Further analysis of the motifs in the MYB factors detected in the current study will provide insights into the mode of action/association (if any) of individual MYB members with repression of the lignin pathway.

The two most commercially significant conifer species in Swedish forestry, Scots pine and Norway spruce (*Picea abies*), respond differently to shade or low R:FR; Scots pine is shade intolerant, while Norway spruce is shade tolerant. SHADE treatment significantly enhanced the hypocotyl growth compared to the SUN treatment in Scots pine, while in Norway spruce, hypocotyl elongation was not enhanced by the SHADE treatment (Ranade et al., 2019). Yet, both species show latitudinal variation in response to light spectra; there is a requirement of FR light to maintain growth in the trees from northern latitudes (Clapham et al., 1998, 2002; Ranade & García‐Gil, 2013, 2021). Further, a recent study on Norway spruce reported an increase in lignin synthesis under shade conditions and revealed a higher number of defense‐related genes being up‐regulated in the northern population compared to the southern ones in response to shade (Ranade et al., 2022), which is similar to Scots pine. Lignin synthesis in response to shade conditions further supports the argument that these two conifer species have adopted alternative strategies regarding lignin synthesis and defense, which differ from the angiosperms.

## 5 CONCLUSIONS

We hypothesize that extended twilight exposure throughout the growing season in the most northern latitudes has been a determining factor promoting local adaptation in the northern Scots pine communities. Adaptation to such stressful light conditions has resulted in northern Socts pine populations being more disease resistant and achieving higher survival rates than the southern population. Although previous investigations already support this reasoning (Andersson et al., 2003; Hansson, 1998), this notion needs to be functionally confirmed. This study reports a novel finding of increased lignin synthesis in Scots pine under shade, which is a contrasting phenomenon when compared to flowering plants. These findings can be applied to future forestry breeding strategies to produce disease‐resistant trees.

## AUTHOR CONTRIBUTIONS

Sonali Sachin Ranade contributed with experimental design, experiment performance, data collection, data analysis and interpretation, and manuscript writing. George Seipel contributed with experiment performance, data collection, data analysis, and interpretation. András Gorzsás contributed with FTIR spectroscopic analyses, data interpretation and manuscript writing. María Rosario García‐Gil contributed with experimental design, data analysis and interpretation, and manuscript writing. All authors read and approved the manuscript.

## Supporting information


**Table S1** Gene expression in response to SHADE in Scots pine at latitude 56
**Table S2** Gene expression in response to SHADE in Scots pine at latitude 67
**Table S3** Gene expression in response to SHADE in Scots pine ‐ latitude 67 versus latitude 56 (north versus south)
**Figure S1** Local light conditions in Sweden throughout the year.
**Figure S2** Venn diagram of the differentially expressed genes under SUN and SHADE for the within latitude comparison in the southern and northern Scots pine population, respectively.
**Figures S3–S20** Pie charts for functional categorization by annotation (GO Biological Process, GO Cellular Component and GO Molecular Function) for the differentially regulated genes for respective treatments and comparisons in Scots pine.
**Figures S21–S34** Gene ontology enrichment analysis for the differentially regulated genes for respective treatments and comparisons in Scots pine (GO Biological Process, GO Cellular Component and GO Molecular Function).

## Data Availability

The RNA‐Seq data were deposited to the ENA and are accessible under the accession number PRJEB19683 (https://www.ebi.ac.uk/ena/data/view/PRJEB19683). All other data are included in the Supplementary data.
