# Supplementary material for: Adaptive strategies of Scots pine under shade: Increase in lignin synthesis and ecotypic variation in defense‐related gene expression
Source: Physiol Plant. 2022 Oct 17;174(5):e13792. doi: 10.1111/ppl.13792 (PMC9827939; doi:10.1111/ppl.13792)

Table S1 Gene expression in response to SHADE in Scots pine at latitude 56

PITA_ID	Confidence	Best BLAST Arabidopsis	Best BLAST Conifer spp	Expression	Gene Model Description	Primary Gene Symbol	GO term-GO ID	baseMean	log2FoldChange	lfcSE	stat	pvalue	padj
PITA_000000037	High quality	AT2G23620	MA_26076g0010	shade<sun	Encodes a protein shown to have carboxy	METHYL ESTERASE 1 (MES1)	hydrolase activity, acting on ester b	239.72546	-2.066712507	0.57467091	-3.596340901	0.000322725	0.004725178
PITA_000000043	Low quality	AT3G54000	MA_213656g0010	shade<sun	TIP41-like protein;(source:Araport11)		biological_process_unknown-GO:0(	462.45546	-1.071781436	0.29493084	-3.634009388	0.000279051	0.004176464
PITA_000000098	High quality	AT1G76490	MA_19627g0010	shade<sun	Encodes a 3-hydroxy-3-methylglutaryl coe	HYDROXY METHYLGLUTARYL COA	coenzyme A metabolic process-GO:	52.550218	-22.4390271	4.4083279	-5.09014474	3.58E-07	1.19E-05
PITA_000000191	High quality	AT4G26090	MA_13844g0010	shade>sun	Encodes a plasma membrane protein witl	RESISTANT TO P. SYRINGAE 2 (RPS2	ADP binding-GO:0043531,defense	339.74465	3.05741362	0.9817977	3.114097359	0.001845086	0.021514853
PITA_000000256	Low quality	AT5G16080	MA_10296151g0010	shade>sun	carboxyesterase 17;(source:Araport11)	CARBOXYESTERASE 17 (CXE17)	cellular_component_unknown-GO:	26.846335	2.267400872	0.49537739	4.577118189	4.71E-06	9.78E-05
PITA_000000276	Low quality	AT2G36770	MA_104361g0010	shade>sun	UDP-Glycosyltransferase superfamily protein;(source:Araport11)		chloroplast-GO:0009507,transferas	166.67712	0.645948949	0.21636398	2.985473553	0.002831396	0.030985638
PITA_000000277	High quality	AT4G17500	MA_166248g0010	shade>sun	Encodes a member of the ERF (ethylene r	ETHYLENE RESPONSIVE ELEMENT E	cell division-GO:0051301,DNA bind	54.32478	2.230186689	0.73706057	3.02578481	0.002479887	0.027779945
PITA_000000286	Low quality	AT4G18550	MA_10435579g0020	shade<sun	DSEL is cytosolic acylhydrolase that shows	DAD1-LIKE SEEDING ESTABLISHMEI	acylglycerol lipase activity-GO:0047	120.20174	-1.430774304	0.25883194	-5.527812074	3.24E-08	1.87E-06
PITA_000000295	High quality	AT1G01880	MA_10432278g0010	shade<sun	Encodes one of two GEN1 homologs in Ar	ORTHOLOG OF HSGEN1 (GEN1)	5'-flap endonuclease activity-GO:0(	553.65216	-1.082466556	0.38071077	-2.843278036	0.00446521	0.045000038
PITA_000000327	Low quality	AT3G10870	MA_498387g0020	shade<sun	Encodes a methyl IAA esterase. Methyl IA	METHYL ESTERASE 17 (MES17)	hydrolase activity, acting on ester b	23.572508	-21.35301597	4.4094576	-4.84254933	1.28E-06	3.20E-05
PITA_000000414	High quality	AT4G16740	MA_74237g0020	shade<sun	Encodes an (E,E)-alpha-farnesene synthas	TERPENE SYNTHASE 03 (TPS03)	(E)-beta-ocimene synthase activity-	26.275109	-21.49994244	4.40924566	-4.876104457	1.08E-06	2.80E-05
PITA_000000568	High quality	AT3G51740	MA_17859g0010	shade>sun	encodes a leucine-repeat receptor kinase	INFLORESCENCE MERISTEM RECEP	cell wall-GO:0005618,kinase activit	43.726462	1.159809668	0.31453386	3.687392089	0.000226564	0.00347833
PITA_000000598	Low quality	AT5G23680	MA_83723g0010	shade>sun	Sterile alpha motif (SAM) domain-containing protein;(source:Araport11)		biological_process_unknown-GO:0(	47.403096	1.241834763	0.29947535	4.146701037	3.37E-05	0.0006139
PITA_000000653	High quality	AT1G62940	MA_8946476g0010	shade<sun	encodes an acyl-CoA synthetase, has in vi	ACYL-COA SYNTHETASE 5 (ACOS5)	4-coumarate-CoA ligase activity-GC	177.76117	-1.880312466	0.59863553	-3.140997103	0.001683737	0.0199524
PITA_000000671	High quality	AT3G13220	MA_114524g0010	shade<sun	Encodes a ATP-binding cassette transport	ATP-BINDING CASSETTE G26 (ABCC	ATPase-coupled transmembrane tr	70.717523	-22.84103454	4.40809223	-5.181614484	2.20E-07	9.17E-06
PITA_000000744	Low quality	AT1G68620	MA_170302g0010	shade<sun	alpha/beta-Hydrolases superfamily protein;(source:Araport11)		cellular response to hypoxia-GO:00	52.715327	-1.188996957	0.30741488	-3.867727436	0.000109854	0.001810264
PITA_000000800	High quality	AT5G51300	MA_8555g0010	shade>sun	Encodes a nuclear localized splicing factor	ARABIDOPSIS SF1 HOMOLOG (ATSI	mRNA binding-GO:0003729,nucleo	64.398344	0.797085533	0.2760525	2.887441847	0.003883884	0.039943173
PITA_000000809	Low quality	AT2G22795	MA_649817g0010	shade<sun	hypothetical protein;(source:Araport11)		biological_process_unknown-GO:0(	91.972264	-9.773098837	2.12964477	-4.589074645	4.45E-06	9.28E-05
PITA_000000819	High quality	AT1G18140	MA_75861g0010	shade>sun	putative laccase, a member of laccase fan	LACCASE 1 (LAC1)	Casparian strip-GO:0048226,coppe	45.519138	1.202493458	0.32195028	3.735028467	0.000187694	0.002943335
PITA_000000882	High quality	AT4G09800	MA_10429265g0010	shade<sun	encodes a ribosomal protein S18C, a cons	S18 RIBOSOMAL PROTEIN (RPS18C	cell wall-GO:0005618,cytoplasm-G	137.90106	-0.730658842	0.24569029	-2.973901988	0.00294039	0.031987044
PITA_000000886	High quality	AT2G05710	MA_7469g0010	shade<sun	Encodes an aconitase that can catalyze th	ACONITASE 3 (ACO3)	4 iron, 4 sulfur cluster binding-GO:(	182.37924	-0.581777899	0.16011059	-3.633600273	0.000279494	0.004180652
PITA_000000910	High quality	AT3G58580	MA_10919g0010	shade>sun	Encodes a protein that is involved in mRN	(ATCCR4B)	3'-5'-exoribonuclease activity-GO:0	31.302571	1.176817862	0.36185273	3.252201184	0.001145149	0.014194087
PITA_000000917	Low quality	AT1G61040	MA_1773g0010	shade<sun	Encodes a yeast Paf1C subunit homolog r	VERNALIZATION INDEPENDENCE 5	Cdc73/Paf1 complex-GO:0016593,	62.263577	-1.569289807	0.48524378	-3.234023566	0.001220594	0.015041851
PITA_000000937	High quality	AT1G64880	MA_955371g0010	shade<sun	Ribosomal protein S5 family protein;(source:Araport11)		chloroplast-GO:0009507,endosome	171.43898	-0.732049147	0.19364708	-3.78032621	0.000156623	0.00250206
PITA_000000994	High quality	AT4G04950	MA_10436042g0030	shade>sun	Encodes a monothiol glutaredoxin that is	MONOTHIOL GLUTAREDOXIN 17 ((	auxin polar transport-GO:0009926,	115.01153	1.675786834	0.54044261	3.100767393	0.001930199	0.022351089
PITA_000001014	Low quality	AT1G12060	MA_10430846g0020	shade<sun	A member of Arabidopsis BAG (Bcl-2-assc	BCL-2-ASSOCIATED ATHANOGENE	chaperone binding-GO:0051087,nu	70.717523	-22.84103454	4.40809223	-5.181614484	2.20E-07	9.17E-06
PITA_000001080	High quality	AT5G42190	MA_10428641g0010	shade>sun	Similar to SKP1 in yeast and humans whic	(SKP1B)	cullin family protein binding-GO:00	121.01943	23.90838197	4.3283673	5.52364906	3.32E-08	1.87E-06
PITA_000001090	High quality	AT2G39510	MA_10432576g0020	shade>sun	Encodes a plasma membrane-localized ar	USUALLY MULTIPLE ACIDS MOVE II	chloroplast-GO:0009507,L-glutama	35.528881	1.745604734	0.58591894	2.979259759	0.002889457	0.03149988
PITA_000001316	Low quality	AT4G25570	MA_831591g0010	shade>sun	Encodes cytochrome b561.	(ACYB-2)	chloroplast-GO:0009507,extracellu	7.6347301	6.676089096	2.36227746	2.826124035	0.004711502	0.046982918
PITA_000001436	High quality	AT5G03860	MA_120902g0010	shade>sun	Encodes a protein with malate synthase a	MALATE SYNTHASE (MLS)	cytoplasm-GO:0005737,glyoxylate	18.454494	2.257493022	0.59638768	3.785277775	0.000153537	0.002457359
PITA_000001454	High quality	AT4G26270	MA_66347g0010	shade<sun	phosphofructokinase 3;(source:Araport11	PHOSPHOFRUCTOKINASE 3 (PFK3)	6-phosphofructokinase activity-GO:	133.72104	-1.037483371	0.26335363	-3.939506683	8.16E-05	0.001380116
PITA_000001475	High quality	AT1G77460	MA_160330g0010	shade>sun	Encodes a plasma membrane, microtubul	CELLULOSE SYNTHASE INTERACTIV	cellulose synthase complex-GO:001	54.980897	0.942844796	0.26939679	3.499836781	0.000465543	0.006543155
PITA_000001617	High quality	AT2G23450	MA_169512g0010	shade<sun	Protein kinase superfamily protein;(source:Araport11)		cell surface receptor signaling pathy	20.238106	-21.14561367	4.4097939	-4.795147836	1.63E-06	3.81E-05
PITA_000001737	Low quality	AT1G47750	MA_20641g0020	shade<sun	member of the peroxin11 (PEX11) gene fa	PEROXIN 11A (PEX11A)	identical protein binding-GO:00428	224.93488	-0.509648878	0.16788666	-3.035672345	0.0024	0.026974739
PITA_000001781	High quality	AT3G55120	MA_118809g0010	shade<sun	Catalyzes the conversion of chalcones inte	TRANSPARENT TESTA 5 (TT5)	chalcone isomerase activity-GO:00	1801.8428	-1.62215003	0.49493429	-3.277505848	0.001047286	0.013137596
PITA_000001823	High quality	AT5G16715	MA_374514g0010	shade<sun	protein EMBRYO DEFECTIVE 2247;(source	EMBRYO DEFECTIVE 2247 (EMB22	aminoacyl-tRNA editing activity-GO	559.55519	-1.208797781	0.17422634	-6.938088427	3.97E-12	6.32E-10
PITA_000001891	High quality	AT2G44130	MA_10314385g0010	shade>sun	Encodes a member of a family of F-box pr	KISS ME DEADLY 3 (KMD3)	cytoplasm-GO:0005737,cytosol-GC	15.876854	1.945361114	0.50882762	3.823222297	0.000131719	0.002138908
PITA_000001899	High quality	AT1G22840	MA_18730g0010	shade<sun	Encodes cytochrome c. Contains two site	CYTOCHROME C-1 (CYTC-1)	copper ion binding-GO:0005507,cy	119.33775	-0.737929465	0.1927057	-3.829308008	0.000128504	0.002092011
PITA_000001959	Low quality	AT2G37050	MA_77429g0020	shade>sun	Leucine-rich repeat protein kinase family protein;(source:Araport11)		ATP binding-GO:0005524,chloropla	397.57409	12.37875254	1.28496123	9.633561098	5.77E-22	5.91E-19
PITA_000002072	Low quality	AT5G41761	MA_810002g0010	shade>sun	hypothetical protein;(source:Araport11)		biological_process_unknown-GO:0(	28.411614	1.280657193	0.42906258	2.984779533	0.002837828	0.031029502
PITA_000002082	High quality	AT4G36810	MA_1011718g0010	shade<sun	Encodes a protein with geranylgeranyl py	GERANYLGERANYL PYROPHOSPHA	chloroplast-GO:0009507,cytosol-G	450.76888	-1.558116026	0.29573135	-5.268687401	1.37E-07	6.41E-06
PITA_000002202	High quality	AT1G22900	MA_10426515g0010	shade<sun	Disease resistance-responsive (dirigent-like protein) family protein;(source:Ar		extracellular region-GO:0005576	534.27607	-1.678583582	0.38043477	-4.412277003	1.02E-05	0.00020196
PITA_000002246	Low quality	AT5G47690	MA_30580g0010	shade>sun	One of 5 PO76/PDS5 cohesion cofactor or	(PDS5A)	chromatin-GO:0000785,cytosol-GC	84.266093	10.14065349	1.60555099	6.315995907	2.68E-10	2.81E-08
PITA_000002257	High quality	AT3G61590	MA_922832g0010	shade>sun	F-box protein that is involved in some asp	HAWAIIAN SKIRT (HWS)	extracellular region-GO:0005576,g	173.88493	1.465727592	0.29168719	5.024998225	5.03E-07	1.60E-05
PITA_000002281	High quality	AT4G34830	MA_122602g0010	shade<sun	Encodes MRL1, a conserved pentatricope	MATURATION OF RBCL 1 (MRL1)	chloroplast-GO:0009507,mRNA sta	334.39044	-0.997510287	0.25317212	-3.940047935	8.15E-05	0.001377916
PITA_000002312	Low quality	AT1G62760	MA_10436535g0030	shade>sun	Pectin methylesterase inhibitor that contr	(ATPMEI10)	enzyme inhibitor activity-GO:0004E	14.514036	2.481170891	0.6154997	4.031148849	5.55E-05	0.000975545
PITA_000002313	High quality	AT4G38620	MA_19163g0010	shade>sun	Encodes a R2R3 MYB protein which is inv	MYB DOMAIN PROTEIN 4 (MYB4)	regulation of phenylpropanoid met	46.369665	1.416052292	0.37800346	3.746135762	0.000179579	0.002831693
PITA_000002369	High quality	AT2G40610	MA_10232070g0010	shade<sun	member of Alpha-Expansin Gene Family.	EXPANSIN A8 (EXPA8)	extracellular region-GO:0005576,p	17.679381	-20.92872422	4.41014042	-4.745591347	2.08E-06	4.59E-05
PITA_000002438	High quality	AT5G52390	MA_55307g0020	shade>sun	PAR1 protein;(source:Araport11)		biological_process_unknown-GO:0(	216.04494	24.71425455	4.32820048	5.710053092	1.13E-08	7.81E-07
PITA_000002536	High quality	AT5G08680	MA_11179g0010	shade<sun	Encodes the mitochondrial ATP synthase beta-subunit. This subunit is encode		ATPase activity-GO:0016887,coppe	541.54225	-0.879374104	0.25017109	-3.515090822	0.000439604	0.00623677
PITA_000002541	High quality	AT5G17230	MA_86494g0010	shade<sun	Encodes phytoene synthase that is the rat	PHYTOENE SYNTHASE (PSY)	carotenoid biosynthetic process-GC	215.2059	-0.592043324	0.16124373	-3.671729325	0.000240915	0.003663507
PITA_000002561	High quality	AT5G47810	MA_101793g0010	shade<sun	phosphofructokinase 2;(source:Araport11	PHOSPHOFRUCTOKINASE 2 (PFK2)	6-phosphofructokinase activity-GO:	91.991029	-9.770692226	1.47990723	-6.602232916	4.05E-11	5.06E-09
PITA_000002687	High quality	AT2G27230	MA_10436087g0020	shade<sun	Encodes a nuclear-localized transcriptiona	LONESOME HIGHWAY (LHW)	DNA-binding transcription factor ac	161.37227	-1.272424684	0.39393943	-3.230000832	0.001237899	0.015233112
PITA_000002706	High quality	AT4G02360	MA_250036g0010	shade<sun	transmembrane protein, putative (Protein of unknown function, DUF538);(sou		biological_process_unknown-GO:0(	290.34476	-2.344128821	0.82415984	-2.844264785	0.004451404	0.044896239
PITA_000002781	High quality	AT5G07050	MA_71776g0010	shade<sun	nodulin MtN21-like transporter family prc	USUALLY MULTIPLE ACIDS MOVE II	chloroplast-GO:0009507,plasma m	344.11404	-3.890358516	0.89017845	-4.370313066	1.24E-05	0.000240693
PITA_000002784	High quality	AT2G41070	MA_28402g0010	shade>sun	Transcription factor homologous to ABI5.	ENHANCED EM LEVEL (EEL)	abscisic acid-activated signaling pat	51.566297	1.462358696	0.40216842	3.636184844	0.000276706	0.004148643
PITA_000002785	High quality	AT5G41380	MA_10435431g0020	shade<sun	CCT motif family protein;(source:Araport11)		nucleus-GO:0005634,protein bindi	120.30368	-0.858078455	0.29747288	-2.88456032	0.003919607	0.040177871
PITA_000002797	High quality	AT4G31750	MA_118899g0010	shade>sun	Encodes HopW1-1-Interacting protein 2 ('	HOPW1-1-INTERACTING 2 (WIN2)	cytosol-GO:0005829,defense respo	28.959488	1.487848521	0.36964149	4.025112323	5.69E-05	0.000996125
PITA_000002896	High quality	AT1G03090	MA_898073g0010	shade>sun	MCCA is the biotinylated subunit of the di	(MCCA)	CoA carboxylase activity-GO:00164	108.02247	23.75154617	4.32841291	5.487356833	4.08E-08	2.16E-06
PITA_000002933	Low quality	AT4G24220	MA_102653g0020	shade>sun	encodes a progesterone-5beta-reductase	VEIN PATTERNING 1 (VEP1)	cytosol-GO:0005829,delta4-3-oxos	132.43718	1.263743097	0.42676096	2.961243451	0.003063996	0.033092712
PITA_000002950	High quality	AT4G33170	MA_8868720g0010	shade<sun	Tetratricopeptide repeat (TPR)-like superfamily protein;(source:Araport11)		chloroplast-GO:0009507,zinc ion bi	924.23386	-1.342551158	0.26378871	-5.089494477	3.59E-07	1.19E-05
PITA_000002968	High quality	AT2G21940	MA_10427362g0010	shade<sun	Encodes a shikimate kinase. Its transcripts	SHIKIMATE KINASE 1 (SK1)	chloroplast-GO:0009507,chorismat	475.111	-0.740411218	0.17356902	-4.265802952	1.99E-05	0.000375053
PITA_000003008	High quality	AT2G37270	MA_20587g0020	shade<sun	One of two genes encoding the ribosomal	RIBOSOMAL PROTEIN 5B (RPS5B)	cell wall-GO:0005618,chloroplast-C	145.64282	-2.204523944	0.36534231	-6.034132545	1.60E-09	1.39E-07
PITA_000003022	High quality	AT4G00030	MA_75636g0010	shade<sun	Plastid-lipid associated protein PAP / fibri	FIBRILLIN9 (FBN9)	biological_process_unknown-GO:0(	333.88035	-0.716489647	0.21356356	-3.354924552	0.000793866	0.010372107
PITA_000003054	Low quality	AT4G24090	MA_24252g0010	shade<sun	homer protein;(source:Araport11)		biological_process_unknown-GO:0(	245.38544	-1.019758172	0.33377733	-3.055205008	0.002249067	0.025506694
PITA_000003056	Low quality	AT5G55040	MA_10431815g0010	shade>sun	DNA-binding bromodomain-containing protein;(source:Araport11)		biological_process_unknown-GO:0(	50.789418	0.970700173	0.2746887	3.533819125	0.000409601	0.005863091
PITA_000003292	High quality	AT5G17920	MA_10090g0010	shade<sun	Encodes a cytosolic cobalamin-independe	METHIONINE SYNTHESIS 1 (ATMS1	5-methyltetrahydropteroyltriglutan	607.90921	-1.176331387	0.27530663	-4.27280437	1.93E-05	0.000363999
PITA_000003310	High quality	AT4G17610	MA_10429620g0010	shade>sun	tRNA/rRNA methyltransferase (SpoU) fan	TRNA METHYLTRANSFERASE 3 (TRI	chloroplast-GO:0009507,RNA bindi	34.670181	8.859457686	2.25804698	3.923504588	8.73E-05	0.001469305
PITA_000003360	Low quality	AT5G11480	MA_311787g0010	shade<sun	P-loop containing nucleoside triphosphate	(ENGB-2)	nucleus-GO:0005634	190.59971	-0.651223906	0.2323631	-2.802613227	0.005069042	0.049620805
PITA_000003395	High quality	AT1G63900	MA_10426532g0010	shade>sun	Encodes a RING-type ubiquitin E3 ligase o	DIAP1-LIKE PROTEIN 1 (DAL1)	chloroplast-GO:0009507,chloropla:	64.599193	0.737630967	0.24594083	2.999221283	0.002706706	0.029850706
PITA_000003453	High quality	AT2G37220	MA_320479g0010	shade<sun	Encodes a chloroplast RNA binding protein. A substrate of the type III effecto		chloroplast-GO:0009507,chloropla:	890.76177	-0.83498191	0.162745	-5.130614781	2.89E-07	1.18E-05
PITA_000003611	High quality	AT3G52960	MA_61158g0010	shade<sun	Thioredoxin superfamily protein;(source:/	PEROXIREDOXIN-II-E (PRXIIE)	cell redox homeostasis-GO:004545	399.00679	-1.274164917	0.18889349	-6.745414839	1.53E-11	2.16E-09
PITA_000003632	High quality	AT2G32230	MA_10050138g0010	shade<sun	Encodes a protein-only RNase P that is inv	PROTEINACEOUS RNASE P 1 (PROR	chloroplast-GO:0009507,mitochon	617.29188	-1.241839161	0.27432131	-4.526951056	5.98E-06	0.000122299
PITA_000003823	High quality	AT4G26780	MA_315276g0010	shade<sun	unknown function	(AR192)	adenyl-nucleotide exchange factor	123.14195	-0.69938323	0.21765624	-3.213246918	0.001312434	0.016031318
PITA_000003837	High quality	AT5G43940	MA_10427177g0010	shade>sun	Encodes a glutathione-dependent formald	SENSITIVE TO HOT TEMPERATURES	alcohol dehydrogenase activity, zin	430.47598	2.220745819	0.60071895	3.696813326	0.000218323	0.003369987
PITA_000003843	High quality	AT3G25400	MA_99024g0010	shade<sun	dCTP pyrophosphatase-like protein;(source:Araport11)		dCTP diphosphatase activity-GO:00	875.39282	-1.690280209	0.48249239	-3.503226682	0.000459658	0.00647814
PITA_000003860	High quality	AT5G42510	MA_46983g0010	shade<sun	Disease resistance-responsive (dirigent-like protein) family protein;(source:Ar		extracellular region-GO:0005576	340.65915	-0.87310111	0.15116633	-5.775764273	7.66E-09	5.62E-07
PITA_000003882	High quality	AT1G70940	MA_100472g0010	shade>sun	A regulator of auxin efflux and involved in	PIN-FORMED 3 (PIN3)	auxin efflux-GO:0010315,auxin effl	33.043787	1.038242436	0.34007913	3.052943695	0.002266085	0.02568831
PITA_000003887	High quality	AT5G39530	MA_96307g0010	shade<sun	hypothetical protein (DUF1997);(source:Araport11)		biological_process_unknown-GO:0(	375.90322	-0.75518368	0.19416397	-3.889412075	0.000100487	0.001672047
PITA_000003909	High quality	AT5G48150	MA_10426489g0020	shade<sun	Member of GRAS gene family. Semi-domi	PHYTOCHROME A SIGNAL TRANSD	callus formation-GO:1990110,cytop	35.358761	-21.90137695	4.40877489	-4.967678662	6.78E-07	2.02E-05
PITA_000003932	High quality	AT5G03340	MA_10168585g0010	shade<sun	ATPase, AAA-type, CDC48 protein;(source	CELL DIVISION CYCLE 48C (ATCDC4	ATPase activity-GO:0016887,autop	60.714318	-22.53151824	4.4082046	-5.11126871	3.20E-07	1.18E-05
PITA_000004048	High quality	AT1G22700	MA_133089g0010	shade<sun	Encodes a TPR protein with homology to '	(PYG7)	chloroplast-GO:0009507,chloropla:	309.50663	-0.814983617	0.1824089	-4.467893823	7.90E-06	0.000158408
PITA_000004058	High quality	AT4G24380	MA_27893g0010	shade<sun	dihydrofolate reductase;(source:Araport11)		chloroplast-GO:0009507,cytoplasm	176.1577	-1.816945556	0.21073884	-8.621787614	6.59E-18	3.25E-15
PITA_000004062	Low quality	AT4G27230	MA_191819g0010	shade<sun	Encodes HTA2, a histone H2A protein.	HISTONE H2A 2 (HTA2)	chromatin silencing-GO:0006342,c	479.17347	-1.106903301	0.23646793	-4.680987042	2.85E-06	6.13E-05
PITA_000004067	High quality	AT5G64570	MA_77922g0010	shade<sun	Encodes a beta-d-xylosidase that belongs	BETA-D-XYLOSIDASE 4 (XYL4)	alpha-L-arabinofuranosidase activit	212.32936	-0.766799675	0.26976713	-2.842450349	0.00447682	0.045099298
PITA_000004071	High quality	AT3G59030	MA_45377g0010	shade<sun	Encodes a proton antiporter. Involved in t	TRANSPARENT TESTA 12 (TT12)	antiporter activity-GO:0015297,chl	3306.8177	-3.102673873	0.12468026	-24.88504534	1.08E-136	2.77E-132
PITA_000004076	High quality	AT2G44350	MA_10430772g0010	shade<sun	encodes a mitochrondrion targeted citrat	(ATCS)	ATP binding-GO:0005524,carbohyc	116.83119	-0.859707449	0.27275015	-3.151996252	0.001621584	0.019314191
PITA_000004115	Low quality	AT1G68840	MA_178646g0010	shade>sun	Rav2 is part of a complex that has been n	RELATED TO ABI3/VP1 2 (RAV2)	cellular response to hypoxia-GO:00	14.43464	3.025673577	0.70082698	4.31729039	1.58E-05	0.000302087
PITA_000004138	High quality	AT5G59480	MA_120403g0010	shade>sun	Haloacid dehalogenase-like hydrolase (HAD) superfamily protein;(source:Arap		nucleus-GO:0005634	20.312602	2.111250076	0.46972817	4.494620908	6.97E-06	0.000141308
PITA_000004166	High quality	AT1G67750	MA_10426822g0020	shade<sun	Pectate lyase family protein;(source:Araport11)		extracellular region-GO:0005576,m	1568.7928	-13.86482036	1.47950258	-9.371271494	7.17E-21	6.80E-18
PITA_000004190	High quality	AT1G77120	MA_95933g0010	shade<sun	Catalyzes the reduction of acetaldehyde u	ALCOHOL DEHYDROGENASE 1 (ADI	alcohol dehydrogenase (NAD+) acti	274.4202	-1.876254931	0.42545014	-4.410046556	1.03E-05	0.000203737
PITA_000004200	High quality	AT1G11860	MA_10429284g0010	shade<sun	Glycine cleavage T-protein family;(source:Araport11)		aminomethyltransferase activity-G(	335.6787	-0.755803416	0.25420842	-2.973164349	0.002947466	0.032036802
PITA_000004207	High quality	AT1G32470	MA_20265g0010	shade<sun	Single hybrid motif superfamily protein;(source:Araport11)		cytoplasm-GO:0005737,cytosol-GC	545.0353	-0.77698449	0.18423875	-4.217269617	2.47E-05	0.000460531
PITA_000004344	High quality	AT3G16640	MA_62989g0010	shade<sun	Encodes a protein homologous to transla	TRANSLATIONALLY CONTROLLED T	apoplast-GO:0048046,auxin homec	286.20443	-2.114546112	0.45547553	-4.642502139	3.44E-06	7.32E-05
PITA_000004527	High quality	AT1G10930	MA_10436537g0010	shade>sun	DNA helicase involved in the maintenance	(RECQ4A)	3'-5' DNA helicase activity-GO:0043	100.26488	1.815913608	0.31479239	5.768607062	7.99E-09	5.81E-07
PITA_000004562	High quality	AT4G20960	MA_10437279g0010	shade<sun	encodes diaminohydroxyphosphoribosyla	PYRIMIDINE DEAMINASE (PYRD)	chloroplast-GO:0009507,chloropla:	295.02451	-0.678335951	0.14296	-4.744935289	2.09E-06	4.60E-05
PITA_000004637	High quality	AT3G24030	MA_136233g0010	shade<sun	hydroxyethylthiazole kinase family protein;(source:Araport11)		cellular_component_unknown-GO:	201.32693	-0.838067339	0.21836804	-3.837866314	0.000124108	0.002030772
PITA_000004768	High quality	AT1G43670	MA_104409g0010	shade<sun	Encodes a fructose-1,6-bisphosphatase. T	FRUCTOSE-1,6-BISPHOSPHATASE (I	cytoplasm-GO:0005737,cytosol-GC	322.09299	-0.923388066	0.19170908	-4.816611018	1.46E-06	3.57E-05
PITA_000004775	High quality	AT3G15810	MA_445275g0010	shade>sun	LURP-one-like protein (DUF567);(source:Araport11)		cytoplasm-GO:0005737,cytosol-GC	116.4604	1.884773496	0.38532497	4.891386845	1.00E-06	2.70E-05
PITA_000005012	Low quality	AT3G50380	MA_10433872g0010	shade>sun	vacuolar protein sorting-associated protein, putative (DUF1162);(source:Arap		extrinsic component of membrane-	231.33499	1.071862614	0.32215558	3.327158295	0.000877365	0.011284564
PITA_000005041	High quality	AT4G33300	MA_127848g0010	shade>sun	Encodes a member of the ADR1 family nu	ADR1-LIKE 1 (ADR1-L1)	ADP binding-GO:0043531,apoplast	19.043114	1.514229058	0.50146156	3.01963136	0.002530825	0.028227078
PITA_000005048	High quality	AT5G19940	MA_10436223g0010	shade<sun	Enables plants to cope with moderate ligh	FIBRILLIN6 (FBN6)	biological_process_unknown-GO:0(	1534.7736	-1.058258597	0.26358207	-4.014911199	5.95E-05	0.001036665
PITA_000005132	High quality	AT5G50250	MA_10434075g0010	shade<sun	Encodes a RNA binding protein. A substra	CHLOROPLAST RNA-BINDING PROT	chloroplast-GO:0009507,chloropla:	542.2877	-0.79333059	0.14625235	-5.424395546	5.82E-08	2.98E-06
PITA_000005186	High quality	AT5G55090	MA_876922g0010	shade<sun	member of MEKK subfamily	MITOGEN-ACTIVATED PROTEIN KIN	nucleus-GO:0005634,protein kinase	52.056017	-2.406944806	0.74538834	-3.229115167	0.001241739	0.015273029
PITA_000005283	High quality	AT4G34200	MA_53302g0010	shade<sun	Encodes a 3-phosphoglycerate dehydroge	EMBRYO SAC DEVELOPMENT ARRE	ATP binding-GO:0005524,chloropla	418.00844	-0.995902759	0.26363063	-3.777644339	0.000158319	0.002522854
PITA_000005330	High quality	AT1G62670	MA_112687g0010	shade<sun	Encodes a pentatricopeptide repeat prote	RNA PROCESSING FACTOR 2 (RPF2	mitochondrion-GO:0005739,RNA 5	188.009	-0.703443342	0.23913823	-2.94157622	0.003265465	0.034871568
PITA_000005519	High quality	AT3G28860	MA_10431815g0020	shade<sun	Encodes a member of the ATP-binding ca	ATP-BINDING CASSETTE B19 (ABCB	acropetal auxin transport-GO:0010	141.35716	-0.634761909	0.18159574	-3.49546694	0.000473233	0.006636666
PITA_000005633	High quality	AT3G12040	MA_176351g0010	shade>sun	Encodes a 3-methyladenine-DNA glycosylase. Arabdiopsis cDNA complements		alkylbase DNA N-glycosylase activit	46.240306	1.494254325	0.34955727	4.274705288	1.91E-05	0.000361173
PITA_000005786	Low quality	AT1G33990	MA_10429301g0010	shade<sun	Encodes a protein predicted to act as a ca	METHYL ESTERASE 14 (MES14)	jasmonic acid metabolic process-G(	105.75202	-1.880894259	0.65526057	-2.870452391	0.004098849	0.041784765
PITA_000005863	Low quality	AT5G65660	MA_91809g0010	shade>sun	hydroxyproline-rich glycoprotein family protein;(source:Araport11)		biological_process_unknown-GO:0(	101.02423	1.393878813	0.38097585	3.658706526	0.000253491	0.00383653
PITA_000005890	Low quality	AT1G54520	MA_10432911g0010	shade<sun	FLAP1 is a chloroplast membrane protein	FLUCTUATING-LIGHT- ACCLIMATIC	chloroplast-GO:0009507,chloropla:	583.67946	-0.88271898	0.21325913	-4.139184995	3.49E-05	0.000632115
PITA_000005896	High quality	AT3G29360	MA_422175g0010	shade<sun	Encodes one of four UDP-glucose dehydro	UDP-GLUCOSE DEHYDROGENASE 2	carbohydrate metabolic process-G(	126.1198	-0.794923962	0.19326792	-4.113067265	3.90E-05	0.000703115
PITA_000005911	High quality	AT1G01050	MA_93206g0010	shade<sun	Encodes a soluble protein with inorganic	PYROPHOSPHORYLASE 1 (PPa1)	cell wall biogenesis-GO:0042546,cy	99.568509	-0.806435797	0.23245511	-3.469210886	0.00052199	0.007198227
PITA_000005919	High quality	AT4G33510	MA_54864g0010	shade<sun	Enzyme catalyzing the first committed ste	3-DEOXY-D-ARABINO-HEPTULOSOI	3-deoxy-7-phosphoheptulonate syn	3970.9693	-1.299763504	0.36392976	-3.57146806	0.000354986	0.005156255
PITA_000006000	High quality	AT1G27680	MA_88294g0010	shade<sun	ADP-glucose pyrophosphorylase catalyzes	ADPGLC-PPASE LARGE SUBUNIT (A	chloroplast-GO:0009507,glucose-1	556.63991	-1.728734614	0.43723201	-3.953815333	7.69E-05	0.001308727
PITA_000006272	High quality	AT1G12680	MA_10341791g0010	shade>sun	phosphoenolpyruvate carboxylase-relatec	PHOSPHOENOLPYRUVATE CARBOX	calcium-dependent protein serine/t	108.02247	23.75154617	4.32841291	5.487356833	4.08E-08	2.16E-06
PITA_000006274	High quality	AT4G16160	MA_21820g0010	shade<sun	Homologous to pea OEP16 and barley pP	(ATOEP16-2)	amino acid transmembrane transp	158.05136	-0.575497156	0.18054313	-3.187588183	0.001434647	0.017354011
PITA_000006279	Low quality	AT4G28290	MA_10427809g0010	shade<sun	hypothetical protein;(source:Araport11)		biological_process_unknown-GO:0(	515.74047	-1.963321407	0.25294987	-7.76170146	8.38E-15	2.38E-12
PITA_000006336	High quality	AT3G54470	MA_43985g0010	shade<sun	encodes the bi-functional orotate phosphoribosyltransferase/orotidine-5'-phc		cellular response to phosphate star	176.60854	-0.653450929	0.17462717	-3.741977385	0.000182578	0.002873667
PITA_000006379	Low quality	AT1G56500	MA_287596g0010	shade<sun	Encodes a thylakoid membrane protein w	SUPPRESSOR OF QUENCHING 1 (SC	chloroplast-GO:0009507,chloropla:	371.469	-1.039045288	0.27170444	-3.824174884	0.000131211	0.002132008
PITA_000006546	Low quality	AT4G14690	MA_10432802g0010	shade<sun	Encodes an early light-induced protein. El	EARLY LIGHT-INDUCIBLE PROTEIN :	cellular response to blue light-GO:0	60.714318	-22.53151824	4.4082046	-5.11126871	3.20E-07	1.18E-05
PITA_000006557	High quality	AT1G08500	MA_94496g0010	shade<sun	early nodulin-like protein 18;(source:Arap	EARLY NODULIN-LIKE PROTEIN 18 (	anchored component of membrane	425.01866	-2.098394687	0.39975676	-5.249178698	1.53E-07	7.05E-06
PITA_000006627	High quality	AT5G52450	MA_14711g0010	shade<sun	MATE efflux family protein;(source:Araport11)		antiporter activity-GO:0015297,me	1052.1486	-1.488485494	0.3208468	-4.639240649	3.50E-06	7.41E-05
PITA_000006650	High quality	AT4G17940	MA_13355g0010	shade<sun	Tetratricopeptide repeat (TPR)-like superfamily protein;(source:Araport11)		biological_process_unknown-GO:0(	573.72809	-0.911601467	0.26813001	-3.399848759	0.000674231	0.009044377
PITA_000006677	High quality	AT1G80760	MA_60111g0010	shade<sun	Encodes a protein with boron transporter	NOD26-LIKE INTRINSIC PROTEIN 6;	active borate transmembrane trans	601.67881	-0.83596326	0.23175786	-3.607054606	0.000309693	0.004552586
PITA_000006702	High quality	AT5G06690	MA_102427g0010	shade<sun	Encodes a thioredoxin (WCRKC1) localize	WCRKC THIOREDOXIN 1 (WCRKC1)	cell redox homeostasis-GO:004545	304.1359	-0.44921725	0.13119262	-3.424104469	0.000616829	0.008353124
PITA_000006797	High quality	AT2G22410	MA_47813g0010	shade>sun	SLOW GROWTH 1;(source:Araport11)	SLOW GROWTH 1 (SLO1)	chloroplast-GO:0009507,chloropla:	48.258603	9.336122962	1.64932191	5.660582619	1.51E-08	1.01E-06
PITA_000006858	High quality	AT1G15960	MA_10430859g0010	shade<sun	member of Nramp2 family	NRAMP METAL ION TRANSPORTER	cadmium ion transmembrane trans	296.18426	-1.683172923	0.50798804	-3.313410515	0.000921656	0.011794985
PITA_000006881	High quality	AT5G13630	MA_108278g0010	shade<sun	Encodes magnesium chelatase involved ir	GENOMES UNCOUPLED 5 (GUN5)	chlorophyll biosynthetic process-G(	647.89974	-0.840884382	0.24848792	-3.384005052	0.000714367	0.009458897
PITA_000006988	Low quality	AT2G19810	MA_98656g0010	shade>sun	Encodes Oxidation-related Zinc Finger 1 ((	OXIDATION-RELATED ZINC FINGER	defense response to virus-GO:0051	31.387239	1.314693447	0.35730592	3.679461682	0.000233727	0.003571167
PITA_000007097	High quality	AT2G18050	MA_5951905g0010	shade>sun	encodes a structurally divergent linker his	HISTONE H1-3 (HIS1-3)	chromosome condensation-GO:003	90.045934	0.953428293	0.28138134	3.388384896	0.000703055	0.009357505
PITA_000007207	Low quality	AT4G33590	MA_58991g0010	shade>sun	transmembrane protein;(source:Araport11)		extracellular region-GO:0005576,tr	33.894182	1.039912007	0.35515881	2.928019732	0.003411284	0.036023159
PITA_000007214	High quality	AT3G49142	MA_10430171g0010	shade<sun	Tetratricopeptide repeat (TPR)-like superfamily protein;(source:Araport11)		intracellular membrane-bounded o	230.47493	-1.095341375	0.29641776	-3.695262256	0.00021966	0.003386545
PITA_000007288	Low quality	AT1G21600	MA_61848g0010	shade<sun	Present in transcriptionally active plastid	PLASTID TRANSCRIPTIONALLY ACTI	chloroplast-GO:0009507,chloropla:	231.11692	-0.694629704	0.23293287	-2.982102507	0.002862761	0.03124876
PITA_000007311	High quality	AT3G21360	MA_10431045g0030	shade>sun	2-oxoglutarate (2OG) and Fe(II)-dependent oxygenase superfamily protein;(sc		cytosol-GO:0005829,nucleus-GO:0(	80.679622	23.34247454	4.32855692	5.392668955	6.94E-08	3.49E-06
PITA_000007357	High quality	AT2G22240	MA_5176g0010	shade<sun	** Referred to as MIPS1 in Mitsuhashi et	MYO-INOSITOL-1-PHOSPHATE SYN	cellular response to phosphate star	1038.6752	-1.434088541	0.4520846	-3.172168563	0.001513052	0.018165131
PITA_000007371	High quality	AT5G35525	MA_483455g0010	shade>sun	PLAC8 family protein;(source:Araport11)		biological_process_unknown-GO:0(	33.147225	1.067746141	0.34765902	3.071245331	0.002131679	0.024326222
PITA_000007413	High quality	AT5G05580	MA_140292g0010	shade<sun	Encodes a temperature sensitive plastidic	FATTY ACID DESATURASE 8 (FAD8)	1-16:0-2-18:2-digalactosyldiacylgly	585.58425	-1.595938421	0.21843202	-7.306339126	2.75E-13	5.86E-11
PITA_000007422	High quality	AT5G41460	MA_12294g0010	shade<sun	transferring glycosyl group transferase (DUF604);(source:Araport11)		acetylglucosaminyltransferase activ	116.25888	-0.6311576	0.21789029	-2.896676088	0.003771389	0.038958344
PITA_000007472	High quality	AT3G53710	MA_46769g0010	shade>sun	A member of ARF GAP domain (AGD), A t	ARF-GAP DOMAIN 6 (AGD6)	cytosol-GO:0005829,Golgi apparat	54.063344	0.764740675	0.26363382	2.900768512	0.003722488	0.038626475
PITA_000007611	High quality	AT1G77760	MA_16402g0010	shade<sun	Encodes the cytosolic minor isoform of nit	NITRATE REDUCTASE 1 (NIA1)	chloroplast-GO:0009507,cytosol-G	330.28753	-2.007303158	0.44326649	-4.528434257	5.94E-06	0.000121638
PITA_000007644	High quality	AT1G73260	MA_14420g0010	shade<sun	Encodes a trypsin inhibitor involved in mc	KUNITZ TRYPSIN INHIBITOR 1 (KTI1	defense response to bacterium-GO	20.290264	-2.282267001	0.70232263	-3.249599128	0.001155678	0.014317659
PITA_000007756	High quality	AT1G67110	MA_5526g0010	shade<sun	cytochrome P450, family 735, subfamily /	CYTOCHROME P450, FAMILY 735,	extracellular region-GO:0005576,h	12142.043	-1.599416491	0.24858026	-6.43420561	1.24E-10	1.42E-08
PITA_000007776	High quality	AT4G22530	MA_34035g0010	shade<sun	S-adenosyl-L-methionine-dependent methyltransferases superfamily protein;(		cytoplasm-GO:0005737,cytosol-GC	1012.9013	-1.685454073	0.58368394	-2.887614264	0.003881756	0.039937326
PITA_000007881	High quality	AT4G31480	MA_896862g0010	shade<sun	Required for plant growth, salt tolerance,	(β1-COP)	chloroplast-GO:0009507,COPI vesi	35.980002	-8.418765323	1.66890963	-5.044470443	4.55E-07	1.46E-05
PITA_000008020	High quality	AT1G55850	MA_10436687g0010	shade>sun	encodes a protein similar to cellulose synt	CELLULOSE SYNTHASE LIKE E1 (CSL	cellulose biosynthetic process-GO:C	125.07463	1.617484668	0.51662016	3.130897298	0.001742731	0.020518554
PITA_000008099	High quality	AT4G30550	MA_125470g0010	shade>sun	Class I glutamine amidotransferase-like su	GAMMA-GLUTAMYL PEPTIDASE 3 (	cytoplasm-GO:0005737,cytosol-GC	39.256731	9.03825343	2.19990779	4.108469208	3.98E-05	0.000715749

PITA_000008114	High quality	AT5G21326	MA_819000g0010	shade>sun	Ca2+regulated serine-threonine protein k	CALCINEURIN B-LIKE PROTEIN (CBL	cytoplasm-GO:0005737,cytosol-GC	42.981358	2.405559654	0.71014244	3.387432601	0.0007055	0.009365711
PITA_000008149	High quality	AT5G20680	MA_430863g0010	shade<sun	Encodes a member of the TBL (TRICHOMI	TRICHOME BIREFRINGENCE-LIKE 1(	endosome-GO:0005768,Golgi appa	395.74995	-0.718461742	0.18309897	-3.923898358	8.71E-05	0.001467869
PITA_000008331	High quality	AT3G20500	MA_10436580g0020	shade<sun	purple acid phosphatase 18;(source:Arap	PURPLE ACID PHOSPHATASE 18 (PA	acid phosphatase activity-GO:0003	771.06955	-1.665874636	0.23285062	-7.154263364	8.41E-13	1.58E-10
PITA_000008357	Low quality	AT1G15760	MA_158232g0010	shade>sun	Sterile alpha motif (SAM) domain-containing protein;(source:Araport11)		biological_process_unknown-GO:0(	135.02809	24.05501755	4.32832795	5.557577383	2.74E-08	1.63E-06
PITA_000008377	High quality	AT4G38620	MA_22140g0010	shade>sun	Encodes a R2R3 MYB protein which is inv	MYB DOMAIN PROTEIN 4 (MYB4)	regulation of phenylpropanoid met	29.512164	1.791521134	0.38156562	4.695184877	2.66E-06	5.74E-05
PITA_000008420	High quality	AT5G08050	MA_114154g0010	shade<sun	Encodes a grana core localized protein. M	(RIQ1)	chloroplast-GO:0009507,chloropla:	1534.6745	-1.288510736	0.21926213	-5.876576656	4.19E-09	3.29E-07
PITA_000008435	Low quality	AT5G65250	MA_575381g0010	shade<sun	transmembrane protein;(source:Araport11)		biological_process_unknown-GO:0(	149.38815	-0.623374375	0.1715728	-3.633293758	0.000279826	0.00418318
PITA_000008562	High quality	AT5G61190	MA_10434363g0010	shade<sun	putative endonuclease or glycosyl hydrolase with C2H2-type zinc finger doma		chloroplast-GO:0009507,nucleic ac	17.679381	-20.92872422	4.41014042	-4.745591347	2.08E-06	4.59E-05
PITA_000008606	High quality	AT5G38520	MA_10433730g0010	shade<sun	CLD1 is involved in steady-state chloroph	CHLOROPHYLL DEPHYTYLASE1 (CLI	chlorophyll metabolic process-GO:(	1139.2629	-1.091030343	0.15847587	-6.884520429	5.80E-12	8.84E-10
PITA_000008626	Low quality	AT4G30490	MA_80622g0010	shade>sun	AFG1-like ATPase family protein;(source:Araport11)		ATPase activity-GO:0016887,biolog	30.965996	1.116032124	0.35580925	3.136602349	0.001709178	0.020188479
PITA_000008702	High quality	AT1G60470	MA_3384g0010	shade>sun	Predicted to encode a galactinol synthase	GALACTINOL SYNTHASE 4 (GolS4)	galactose metabolic process-GO:00	20.711173	1.245001052	0.43636606	2.853111544	0.004329342	0.043768573
PITA_000008714	High quality	AT5G06720	MA_125040g0020	shade<sun	Encodes a peroxidase with diverse roles ir	PEROXIDASE 2 (PA2)	defense response to nematode-GO	52.550218	-22.4390271	4.4083279	-5.09014474	3.58E-07	1.19E-05
PITA_000008784	High quality	AT2G30780	MA_898819g0010	shade<sun	Tetratricopeptide repeat (TPR)-like superfamily protein;(source:Araport11)		biological_process_unknown-GO:0(	384.58007	-0.851358367	0.25565268	-3.330136724	0.000868033	0.011203932
PITA_000009146	High quality	AT5G19820	MA_10432206g0010	shade<sun	Encodes an importin that transports HYL1	(KARYOPHERIN ENABLING THE TRA	cell wall-GO:0005618,cytoplasm-G	52.550218	-22.4390271	4.4083279	-5.09014474	3.58E-07	1.19E-05
PITA_000009193	Low quality	AT3G12110	MA_10043102g0010	shade>sun	Encodes an actin that is expressed predor	ACTIN-11 (ACT11)	actin cytoskeleton organization-GO	98.683367	10.36823236	2.24583389	4.616651477	3.90E-06	8.19E-05
PITA_000009256	High quality	AT2G34690	MA_10427262g0010	shade<sun	Gene product transports the glycolipid pr	ACCELERATED CELL DEATH 11 (ACI	cell death-GO:0008219,ceramide 1	60.714318	-22.53151824	4.4082046	-5.11126871	3.20E-07	1.18E-05
PITA_000009269	High quality	AT5G62420	MA_44602g0010	shade<sun	NAD(P)-linked oxidoreductase superfamily protein;(source:Araport11)		alditol:NADP+ 1-oxidoreductase act	135.55966	-0.869450635	0.20456208	-4.250302166	2.13E-05	0.000400209
PITA_000009358	High quality	AT1G73050	MA_46380g0010	shade>sun	Glucose-methanol-choline (GMC) oxidoreductase family protein;(source:Arap		cyanide biosynthetic process-GO:0(	135.83421	0.737225233	0.24544254	3.003657128	0.002667558	0.02950518
PITA_000009371	Low quality	AT2G03200	MA_15755g0010	shade>sun	Atypical aspartic protease which modulat	ATYPICAL ASPARTIC PROTEASE IN I	extracellular region-GO:0005576,p	70.18471	1.00463896	0.28424497	3.534412422	0.000408683	0.005853216
PITA_000009507	High quality	AT4G36820	MA_390657g0010	shade<sun	Mitochondrial calcium channel.	(MCU4)	calcium channel activity-GO:00052	130.97653	-1.657330623	0.24750794	-6.696070411	2.14E-11	2.93E-09
PITA_000009549	High quality	AT3G57680	MA_74926g0010	shade<sun	Peptidase S41 family protein;(source:Araport11)		endopeptidase activity-GO:000417	1320.9801	-0.700309396	0.21319171	-3.284881026	0.001020254	0.012838661
PITA_000009608	Low quality	AT3G09550	MA_10430545g0010	shade<sun	Ankyrin repeat family protein;(source:Araport11)		biological_process_unknown-GO:0(	60.714318	-22.53151824	4.4082046	-5.11126871	3.20E-07	1.18E-05
PITA_000009655	High quality	AT4G02780	MA_10433916g0010	shade<sun	Catalyzes the conversion of geranylgeran	GA REQUIRING 1 (GA1)	chloroplast-GO:0009507,diterpeno	20.238106	-21.14561367	4.4097939	-4.795147836	1.63E-06	3.81E-05
PITA_000009668	High quality	AT3G27180	MA_10426931g0010	shade<sun	S-adenosyl-L-methionine-dependent methyltransferases superfamily protein;(		chloroplast-GO:0009507,RNA meth	331.06111	-0.584559607	0.17724911	-3.297955182	0.000973917	0.012377201
PITA_000009750	Low quality	AT1G49230	MA_85088g0010	shade<sun	RING/U-box superfamily protein;(source:	ARABIDOPSIS TOXICOS EN LEVADU	protein ubiquitination-GO:0016567	60.714318	-22.53151824	4.4082046	-5.11126871	3.20E-07	1.18E-05
PITA_000009808	High quality	AT5G62790	MA_8158336g0010	shade<sun	1-Deoxy-d-xylulose 5-phosphate reductoi:	1-DEOXY-D-XYLULOSE 5-PHOSPHAT	1-deoxy-D-xylulose-5-phosphate re	301.10377	-0.727117972	0.19215983	-3.783922794	0.000154376	0.002467699
PITA_000009923	High quality	AT4G01370	MA_10432928g0010	shade<sun	Encodes a nuclear and cytoplasmically loc	MAP KINASE 4 (MPK4)	cell plate-GO:0009504,cellular resp	70.717523	-22.84103454	4.40809223	-5.181614484	2.20E-07	9.17E-06
PITA_000009955	Low quality	AT3G02330	MA_10138132g0010	shade<sun	Involved in cytidine to uridine editing of tl	MITOCHONDRIAL EDITING FACTOR	cytidine to uridine editing-GO:0016	1430.0243	-2.893583378	0.69471335	-4.165147235	3.11E-05	0.000570363
PITA_000010154	High quality	AT3G62700	MA_629244g0010	shade<sun	member of MRP subfamily	ATP-BINDING CASSETTE C14 (ABCC	ABC-type xenobiotic transporter ac	126.35402	-1.752415836	0.34283714	-5.111511057	3.20E-07	1.18E-05
PITA_000010201	High quality	AT5G05270	MA_502949g0010	shade<sun	Chalcone-flavanone isomerase family pro	CHALCONE ISOMERASE LIKE (CHIL)	chalcone isomerase activity-GO:00	398.03768	-2.147409969	0.15728502	-13.65298428	1.94E-42	1.24E-38
PITA_000010254	High quality	AT1G22640	MA_180449g0010	shade<sun	MYB-type transcription factor (MYB3) tha	MYB DOMAIN PROTEIN 3 (MYB3)	response to wounding-GO:000961	450.76403	-3.22679141	0.80206827	-4.023088234	5.74E-05	0.001004043
PITA_000010271	High quality	AT4G36360	MA_10427427g0010	shade<sun	putative beta-galactosidase (BGAL3 gene)	BETA-GALACTOSIDASE 3 (BGAL3)	beta-galactosidase activity-GO:000	498.43372	-1.130029567	0.15075103	-7.495999226	6.58E-14	1.60E-11
PITA_000010337	Low quality	AT4G26850	MA_56912g0020	shade<sun	Encodes a novel protein involved in ascor	VITAMIN C DEFECTIVE 2 (VTC2)	cytoplasm-GO:0005737,defense re	236.78706	-0.835678206	0.17292302	-4.832660204	1.35E-06	3.32E-05
PITA_000010358	High quality	AT4G13940	MA_10433454g0020	shade<sun	Encodes a S-adenosyl-L-homocysteine hyc	HOMOLOGY-DEPENDENT GENE SIL	adenosylhomocysteinase activity-G	250.81201	-0.702130097	0.21752582	-3.227801217	0.001247456	0.015333527
PITA_000010404	Low quality	AT3G60520	MA_10433910g0010	shade<sun	zinc ion-binding protein;(source:Araport11)		cellular response to hypoxia-GO:00	35.358761	-21.90137695	4.40877489	-4.967678662	6.78E-07	2.02E-05
PITA_000010409	High quality	AT3G48310	MA_10436871g0020	shade>sun	putative cytochrome P450	CYTOCHROME P450, FAMILY 71, SI	heme binding-GO:0020037,iron ior	11.804548	3.069004067	0.91805392	3.342945339	0.000828942	0.010764479
PITA_000010423	Low quality	AT5G16760	MA_41802g0010	shade<sun	Encodes a inositol 1,3,4-trisphosphate 5/(	INOSITOL 1,3,4-TRISPHOSPHATE 5/	cytoplasm-GO:0005737,cytosol-GC	183.44331	-0.831299142	0.20712724	-4.013470888	5.98E-05	0.001041596
PITA_000010453	High quality	AT2G29630	MA_10434897g0010	shade<sun	Encodes a protein involved in thiamin bio	THIAMINC (THIC)	ADP-ribose pyrophosphohydrolase	209.97859	-1.095302366	0.21113485	-5.187691095	2.13E-07	9.17E-06
PITA_000010567	High quality	AT1G17050	MA_17853g0010	shade<sun	Encodes one of the two paralogous solan	SOLANESYL DIPHOSPHATE SYNTHA	all-trans-nonaprenyl-diphosphate s	383.40001	-1.122179196	0.17317751	-6.479936226	9.18E-11	1.09E-08
PITA_000010577	High quality	AT2G02990	MA_95383g0020	shade>sun	Encodes a member of the ribonuclease T2	RIBONUCLEASE 1 (RNS1)	aging-GO:0007568,anthocyanin-co	121.01943	23.90838197	4.3283673	5.52364906	3.32E-08	1.87E-06
PITA_000010599	High quality	AT2G36780	MA_10426944g0020	shade<sun	UDP-Glycosyltransferase superfamily protein;(source:Araport11)		chloroplast-GO:0009507,transferas	23.572508	-21.35301597	4.4094576	-4.84254933	1.28E-06	3.20E-05
PITA_000010811	High quality	AT3G21190	MA_24566g0010	shade<sun	O-fucosyltransferase family protein;(sour	MANNAN SYNTHESIS RELATED 1 (N	cell wall pectin biosynthetic proces:	317.19585	-0.85373766	0.30295748	-2.818011527	0.004832208	0.04783269
PITA_000010966	High quality	AT3G11050	MA_139907g0010	shade>sun	ferritin 2;(source:Araport11)	FERRITIN 2 (FER2)	chloroplast-GO:0009507,cytoplasm	425.79153	3.047229064	0.96195708	3.167739124	0.001536293	0.018383829
PITA_000011054	High quality	AT3G56430	MA_206524g0010	shade<sun	TIM domain protein. Associates with com	ATTIM21-LIKE 2 (ATTIM21L-2)	biological_process_unknown-GO:0(	144.67989	-0.564585694	0.19159909	-2.946703378	0.003211811	0.034383525
PITA_000011252	High quality	AT1G10510	MA_19479g0010	shade<sun	RNI-like superfamily protein;(source:Arap	EMBRYO DEFECTIVE 2004 (emb20(	chloroplast-GO:0009507,chloropla:	145.9313	-0.588303022	0.1946305	-3.022666202	0.002505585	0.028018781
PITA_000011255	High quality	AT4G15630	MA_10431939g0010	shade>sun	Uncharacterized protein family (UPF0497	CASP-LIKE PROTEIN 1E1 (CASPL1E1	4 iron, 4 sulfur cluster binding-GO:(	15.082661	1.586249249	0.50002772	3.172322606	0.001512249	0.018164013
PITA_000011323	High quality	AT5G04430	MA_10437213g0010	shade<sun	Gene model AT5G04430.1 produces activ	BINDING TO TOMV RNA 1L (LONG	cytoplasm-GO:0005737,cytosol-GC	152.75501	-1.220889491	0.40548681	-3.010922802	0.002604551	0.028898324
PITA_000011377	Low quality	AT2G42350	MA_136974g0020	shade>sun	RING/U-box superfamily protein;(source:	ARABIDOPSIS T??XICOS EN LEVADU	nucleus-GO:0005634,protein ubiqu	48.50471	1.201782669	0.4050089	2.967299431	0.003004282	0.03260617
PITA_000011478	Low quality	AT4G36040	MA_10427774g0010	shade<sun	Chaperone DnaJ-domain superfamily prot	DNAJ11 (J11)	chloroplast-GO:0009507,nucleus-G	323.94528	-1.850267582	0.4528636	-4.085706137	4.39E-05	0.000781995
PITA_000011481	High quality	AT4G39470	MA_10426317g0010	shade<sun	Tetratricopeptide repeat (TPR)-like superfamily protein;(source:Araport11)		biological_process_unknown-GO:0(	365.03053	-1.052156187	0.13008451	-8.088251278	6.05E-16	2.04E-13
PITA_000011482	High quality	AT3G48280	MA_353950g0010	shade<sun	putative cytochrome P450	CYTOCHROME P450, FAMILY 71, SI	Golgi apparatus-GO:0005794,Golgi	376.02387	-1.270501949	0.14492388	-8.766684839	1.84E-18	1.00E-15
PITA_000011568	Low quality	AT3G55250	MA_11912g0010	shade<sun	Encodes a nucleus-encoded protein, Phot	PIGMENT DEFECTIVE 329 (PDE329)	chloroplast-GO:0009507,chloropla:	213.7128	-0.637706566	0.22301846	-2.859434034	0.004243977	0.043007422
PITA_000011629	High quality	AT5G17710	MA_10432570g0020	shade<sun	Chloroplast GrpE protein involved in chlo	EMBRYO DEFECTIVE 1241 (EMB12	adenyl-nucleotide exchange factor	242.90904	-0.577037215	0.15782634	-3.65615288	0.000256029	0.003865792
PITA_000011688	Low quality	AT3G47790	MA_10430842g0010	shade>sun	ABC2 homolog 7;(source:Araport11)	ATP-BINDING CASSETTE A8 (ABCA8	ATPase activity-GO:0016887,ATPas	714.97327	1.348714887	0.37123333	3.633065195	0.000280074	0.004184446
PITA_000011689	High quality	AT3G47780	MA_119138g0010	shade>sun	member of ATH subfamily The mRNA is c	ATP-BINDING CASSETTE A7 (ABCA7	ATPase activity-GO:0016887,ATPas	594.23261	1.288118248	0.36270201	3.551450585	0.000383114	0.005511675
PITA_000011783	High quality	AT1G54180	MA_129791g0010	shade>sun	Belongs to five-member BRX gene family.	BREVIS RADIX-LIKE 3 (BRX-LIKE3)	biological_process_unknown-GO:0(	513.05854	2.063525675	0.56514158	3.651342881	0.000260873	0.003931976
PITA_000011784	Low quality	AT1G72880	MA_10234g0010	shade<sun	Survival protein SurE-like phosphatase/nucleotidase;(source:Araport11)		5'-nucleotidase activity-GO:000825	52.550218	-22.4390271	4.4083279	-5.09014474	3.58E-07	1.19E-05
PITA_000011808	High quality	AT1G11530	MA_164160g0010	shade>sun	Encodes a monocysteinic thioredoxin, thi	C-TERMINAL CYSTEINE RESIDUE IS	cytoplasm-GO:0005737,cytosol-GC	28.426623	2.055909885	0.47729664	4.307404879	1.65E-05	0.000314495
PITA_000011915	Low quality	AT5G63450	MA_18375g0010	shade<sun	AtWRKY33 regulates root apoplastic barri	CYTOCHROME P450, FAMILY 94, SI	chloroplast-GO:0009507,heme bin	377.96732	-11.81128883	1.72704599	-6.839012347	7.97E-12	1.19E-09
PITA_000011954	Low quality	AT1G76940	MA_405456g0010	shade<sun	RNA-binding (RRM/RBD/RNP motifs) fam	(NSRA)	mRNA binding-GO:0003729,nucleu	84.89986	-9.655848307	1.6910945	-5.709821845	1.13E-08	7.81E-07
PITA_000011965	High quality	AT3G48000	MA_138834g0010	shade<sun	Encodes a putative (NAD+) aldehyde dehy	ALDEHYDE DEHYDROGENASE 2B4 (	aldehyde dehydrogenase (NAD+) a	77.542895	-0.798427577	0.25851447	-3.088521852	0.002011549	0.023167635
PITA_000012058	High quality	AT5G47230	MA_115348g0020	shade<sun	encodes a member of the ERF (ethylene r	ETHYLENE RESPONSIVE ELEMENT E	DNA binding-GO:0003677,DNA-bin	52.550218	-22.4390271	4.4083279	-5.09014474	3.58E-07	1.19E-05
PITA_000012070	Low quality	AT1G27850	MA_10429385g0010	shade>sun	Encodes a microtubule-associated proteir	BASIC PROLINE-RICH PROTEIN5 (BF	anisotropic cell growth-GO:005121	57.7863	0.834183636	0.25600401	3.258478809	0.001120112	0.013944501
PITA_000012080	Low quality	AT1G64660	MA_50045g0010	shade>sun	Encodes a functional methionine gamma-	METHIONINE GAMMA-LYASE (MGI	carbon-sulfur lyase activity-GO:001	18.623602	1.879251673	0.49006435	3.834703922	0.000125716	0.002051833
PITA_000012120	High quality	AT3G23330	MA_175688g0010	shade<sun	Tetratricopeptide repeat (TPR)-like superfamily protein;(source:Araport11)		intracellular membrane-bounded o	536.05675	-1.449765271	0.16463317	-8.806033748	1.30E-18	7.38E-16
PITA_000012128	High quality	AT3G01480	MA_7732g0010	shade<sun	Encodes a chloroplast cyclophilin function	CYCLOPHILIN 38 (CYP38)	chloroplast-GO:0009507,chloropla:	443.54033	-0.829830876	0.15671305	-5.295225207	1.19E-07	5.65E-06
PITA_000012151	High quality	AT5G48940	MA_10429514g0010	shade>sun	RGFR2 is a leucine--rich repeat receptor k	(RGFR2)	maintenance of root meristem ider	121.01943	23.90838197	4.3283673	5.52364906	3.32E-08	1.87E-06
PITA_000012188	Low quality	AT2G02960	MA_10433939g0010	shade>sun	RING/FYVE/PHD zinc finger superfamily protein;(source:Araport11)		membrane-GO:0016020,nucleus-G	63.765518	0.890947718	0.2656963	3.353255995	0.000798668	0.010416733
PITA_000012496	High quality	AT3G23400	MA_10429604g0010	shade<sun	Encodes FIBRILLIN 4 (FIB4). The fibrillins a	FIBRILLIN 4 (FIB4)	chloroplast-GO:0009507,chloropla:	539.17255	-1.273360124	0.35125609	-3.625161684	0.000288781	0.004289499
PITA_000012498	High quality	AT2G34590	MA_118815g0010	shade<sun	Transketolase family protein;(source:Araport11)		acetyl-CoA biosynthetic process fro	121.24423	-0.890647153	0.23073202	-3.860093431	0.000113344	0.001864165
PITA_000012622	Low quality	AT3G60370	MA_170550g0010	shade<sun	Encodes an immunophilin, FKBP20-2, that belongs to the FK-506 binding prot		chloroplast-GO:0009507,chloropla:	445.57612	-1.432102064	0.24601604	-5.821173512	5.84E-09	4.45E-07
PITA_000012712	Low quality	AT1G72310	MA_6697g0010	shade<sun	Encodes a putative RING-H2 zinc finger pr	(ATL3)	nucleus-GO:0005634,protein ubiqu	26.107157	-21.49092332	4.40925805	-4.87404527	1.09E-06	2.81E-05
PITA_000012746	High quality	AT3G43540	MA_10098847g0010	shade<sun	initiation factor 4F subunit (DUF1350);(source:Araport11)		chloroplast-GO:0009507,extracellu	2268.6605	-1.006275662	0.29440807	-3.417962173	0.000630919	0.008530395
PITA_000012766	High quality	AT1G79460	MA_57103g0010	shade<sun	Encodes for a protein with ent-kaurene sy	GA REQUIRING 2 (GA2)	chloroplast-GO:0009507,chloropla:	538.8324	-1.217309452	0.20196168	-6.027427701	1.67E-09	1.44E-07
PITA_000012787	High quality	AT4G27990	MA_961483g0010	shade<sun	One of four Arabidopsis homologs of bact	(YLMG1-2)	chloroplast-GO:0009507,chloropla:	171.16541	-0.610456287	0.20328459	-3.002963841	0.002673642	0.029549686
PITA_000012823	High quality	AT5G56660	MA_10428403g0010	shade>sun	encodes IAA-amino acid hydrolase that ha	IAA-LEUCINE RESISTANT (ILR)-LIKE	auxin metabolic process-GO:00098	65.114429	1.155902036	0.30299819	3.814881022	0.000136249	0.002199907
PITA_000013064	High quality	AT2G16230	MA_67006g0010	shade<sun	O-Glycosyl hydrolases family 17 protein;(source:Araport11)		anchored component of plasma me	117.67698	-1.287864683	0.40533219	-3.177306663	0.001486498	0.01787147
PITA_000013083	High quality	AT1G28960	MA_21957g0010	shade<sun	Encodes a ppGpp pyrophosphohydrolase.	NUDIX HYDROLASE HOMOLOG 15	acetyl-CoA hydrolase activity-GO:0(	106.91048	-0.783823148	0.26999872	-2.903062486	0.003695329	0.038420619
PITA_000013279	Low quality	AT2G03270	MA_10435405g0010	shade<sun	DNA-binding protein;(source:Araport11)		5'-3' DNA helicase activity-GO:0043	62.862931	-0.808142036	0.2740987	-2.948361469	0.003194633	0.034223621
PITA_000013363	High quality	AT2G03890	MA_2516g0010	shade>sun	Phosphoinositide kinase which undergo a	PHOSPHOINOSITIDE 4-KINASE GAN	1-phosphatidylinositol 4-kinase acti	22.616928	1.288838507	0.4120447	3.127909464	0.001760544	0.020694534
PITA_000013409	High quality	AT4G28660	MA_18349g0010	shade<sun	Similar to PsbW subunit of photosystem I	PHOTOSYSTEM II REACTION CENTE	chloroplast-GO:0009507,chloropla:	553.87999	-0.583072126	0.11000479	-5.300424877	1.16E-07	5.51E-06
PITA_000013474	High quality	AT3G15680	MA_101037g0010	shade>sun	Ran BP2/NZF zinc finger-like superfamily protein;(source:Araport11)		mRNA binding-GO:0003729,nucleu	34.099415	1.485891401	0.34463328	4.311514595	1.62E-05	0.000309857
PITA_000013522	High quality	AT2G21100	MA_10068901g0010	shade<sun	Disease resistance-responsive (dirigent-like protein) family protein;(source:Ar		cell differentiation-GO:0030154,tis:	44.644289	-1.945847604	0.54965337	-3.540135861	0.000399921	0.005743791
PITA_000013593	Low quality	AT1G54690	MA_94328g0020	shade>sun	Encodes HTA3, a histone H2A protein. H2	GAMMA HISTONE VARIANT H2AX (	chromatin silencing-GO:0006342,D	55.765589	0.996913746	0.27689913	3.600277581	0.000317878	0.004664877
PITA_000013640	High quality	AT1G02660	MA_75319g0010	shade>sun	PLIP2 is a glycerolipid A1 lipase with subs	PLASTID LIPASE2 (PLIP2)	1-18:1-2-16:0-monogalactosyldiacy	47.775914	1.061339017	0.28123185	3.77389342	0.000160719	0.002554751
PITA_000013682	Low quality	AT5G13200	MA_10113221g0010	shade<sun	Encodes a protein with unknown function	GEM-RELATED 5 (GER5)	cytoplasm-GO:0005737,embryo de	26.275109	-21.49994244	4.40924566	-4.876104457	1.08E-06	2.80E-05
PITA_000013695	High quality	AT1G19180	MA_10158949g0010	shade<sun	JAZ1 is a nuclear-localized protein involve	JASMONATE-ZIM-DOMAIN PROTEI	defense response to bacterium-GO	126.97468	-2.314440195	0.51052191	-4.533478714	5.80E-06	0.000118957
PITA_000013716	Low quality	AT1G27340	MA_10432527g0010	shade<sun	Encodes a putative F-box protein that is ir	LEAF CURLING RESPONSIVENESS (L	leaf vascular tissue pattern formati	52.550218	-22.4390271	4.4083279	-5.09014474	3.58E-07	1.19E-05
PITA_000013835	Low quality	AT5G01310	MA_306658g0010	shade>sun	Encodes a protein that has adenylylsulfat	APRATAXIN-LIKE (APTX)	adenylylsulfatase activity-GO:0047	35.006133	8.87289845	1.93609299	4.582888582	4.59E-06	9.52E-05
PITA_000013884	Low quality	AT5G17210	MA_94549g0010	shade<sun	transmembrane protein, putative (DUF1218);(source:Araport11)		biological_process_unknown-GO:0(	289.80255	-2.084638376	0.61938248	-3.365672169	0.000763574	0.010006656
PITA_000013910	High quality	AT3G22060	MA_113766g0010	shade>sun	contains Pfam profile: PF01657 Domain of unknown function that is usually a:		cell wall-GO:0005618,cytosol-GO:0	39.459463	9.046112227	2.2348652	4.04772164	5.17E-05	0.00091528
PITA_000013954	Low quality	AT3G02660	MA_766311g0010	shade<sun	Tyrosyl-tRNA synthetase, class Ib, bacteri	EMBRYO DEFECTIVE 2768 (emb27(	chloroplast-GO:0009507,chloropla:	248.89708	-0.857971012	0.25732797	-3.33415372	0.000855594	0.011071269
PITA_000014031	High quality	AT4G27220	MA_10435615g0010	shade<sun	NB-ARC domain-containing disease resistance protein;(source:Araport11)		ADP binding-GO:0043531,mitocho	138.63091	-1.181237557	0.31261002	-3.778629826	0.000157694	0.002516024
PITA_000014143	Low quality	AT2G36370	MA_817606g0010	shade>sun	ubiquitin-protein ligase;(source:Araport11)		protein ubiquitination-GO:0016567	50.825864	0.859301728	0.29983701	2.86589618	0.004158306	0.042304622
PITA_000014187	High quality	AT2G01890	MA_55937g0010	shade<sun	Encodes a purple acid phosphatase (PAP)	PURPLE ACID PHOSPHATASE 8 (PAI	acid phosphatase activity-GO:0003	131.24553	-1.908339476	0.6132653	-3.111768245	0.001859704	0.021646958
PITA_000014289	High quality	AT5G09810	MA_10427661g0030	shade<sun	Member of Actin gene family.Mutants are	ACTIN 7 (ACT7)	cell division-GO:0051301,cell wall-(	588.16819	-12.44949192	1.84396543	-6.751477929	1.46E-11	2.08E-09
PITA_000014330	High quality	AT2G46700	MA_3361g0010	shade<sun	CDPK-related kinase 3;(source:Araport11)	CDPK-RELATED KINASE 3 (CRK3)	ATP binding-GO:0005524,calcium i	28.287009	-21.59902252	4.40911624	-4.898719231	9.65E-07	2.61E-05
PITA_000014352	High quality	AT1G28330	MA_192059g0010	shade>sun	dormancy-associated protein (DRM1)	DORMANCY-ASSOCIATED PROTEIN	molecular_function_unknown-GO:(	9.2483264	4.416443555	1.37771995	3.205617782	0.001347729	0.016403345
PITA_000014374	Low quality	AT5G38210	MA_50079g0010	shade>sun	Protein kinase family protein;(source:Ara	LEAF RUST 10 DISEASE-RESISTANCE	extracellular region-GO:0005576,ki	71.521607	1.041309645	0.30101376	3.459342323	0.000541496	0.007431209
PITA_000014427	High quality	AT3G48990	MA_101658g0010	shade<sun	Encodes an oxalyl-CoA synthetase and is r	ACYL-ACTIVATING ENZYME 3 (AAE	apoplast-GO:0048046,chloroplast-(	286.36853	-1.909863441	0.34044015	-5.609982983	2.02E-08	1.24E-06
PITA_000014448	High quality	AT3G49780	MA_168710g0010	shade<sun	Phytosulfokine 3 precursor, coding for a u	PHYTOSULFOKINE 4 PRECURSOR (F	cell differentiation-GO:0030154,cel	1140.618	-1.191753096	0.1756372	-6.785311576	1.16E-11	1.70E-09
PITA_000014450	High quality	AT4G09650	MA_657g0010	shade<sun	Encodes the chloroplast ATPase delta-sub	ATP SYNTHASE DELTA-SUBUNIT GE	ATP synthesis coupled proton trans	363.13895	-0.702638949	0.212667	-3.303939683	0.000953363	0.01216429
PITA_000014471	High quality	AT1G53210	MA_10281743g0010	shade>sun	Encodes a Na+/Ca 2+ exchanger-like prot	NA+/CA2+ EXCHANGER (NCL)	calcium ion binding-GO:0005509,c	14.083906	1.606490127	0.56536011	2.841534246	0.004489703	0.045193519
PITA_000014598	Low quality	AT5G49690	MA_9720131g0020	shade<sun	UDP-Glycosyltransferase superfamily prot	UDP-GLYCOSYLTRANSFERASE 91C1	cytosol-GO:0005829,intracellular n	242.82021	-1.51700169	0.33918229	-4.472526267	7.73E-06	0.000155623
PITA_000014719	Low quality	AT5G53140	MA_10426103g0010	shade<sun	Protein phosphatase 2C family protein;(source:Araport11)		chloroplast-GO:0009507,cytosol-G	143.92899	-2.796342945	0.46481331	-6.01605614	1.79E-09	1.53E-07
PITA_000014772	High quality	AT2G26330	MA_5825101g0010	shade<sun	Homologous to receptor protein kinases.	ERECTA (ER)	cellular heat acclimation-GO:00703	760.19535	-12.81932876	4.40747311	-2.90854384	0.003631163	0.037953801
PITA_000014815	High quality	AT4G34860	MA_7394g0010	shade<sun	Plant neutral invertase family protein;(so	ALKALINE/NEUTRAL INVERTASE B (	cytosol-GO:0005829,glycopeptide	200.12544	-0.644119621	0.19503377	-3.302605589	0.00095791	0.012210136
PITA_000014818	High quality	AT5G05390	MA_10427033g0010	shade<sun	putative laccase, a member of laccase fa	LACCASE 12 (LAC12)	copper ion binding-GO:0005507,ex	248.30732	-11.20591256	1.53116919	-7.318533209	2.51E-13	5.40E-11
PITA_000014878	Low quality	AT1G74670	MA_7575249g0010	shade>sun	Gibberellin-regulated family protein;(sour	GA-STIMULATED ARABIDOPSIS 6 ((	extracellular region-GO:0005576,g	41.162907	1.766137794	0.47091195	3.750462853	0.000176508	0.002790141
PITA_000014902	High quality	AT1G53090	MA_110435g0020	shade<sun	Encodes a member of the SPA (suppresso	SPA1-RELATED 4 (SPA4)	ATP binding-GO:0005524,chloropla	197.91206	-1.078590108	0.29666079	-3.635769002	0.000277153	0.00415291
PITA_000015018	High quality	AT4G15910	MA_10428902g0010	shade<sun	encodes a gene whose transcript level in	DROUGHT-INDUCED 21 (DI21)	chloroplast-GO:0009507,cold acclir	86.763363	-1.375078745	0.25707478	-5.348944607	8.85E-08	4.32E-06
PITA_000015178	High quality	AT4G15510	MA_81231g0010	shade<sun	A nuclear-encoded thylakoid lumenal pro	PSBP-DOMAIN PROTEIN1 (PPD1)	calcium ion binding-GO:0005509,cl	1378.7675	-0.949931794	0.20598878	-4.611570599	4.00E-06	8.38E-05
PITA_000015201	High quality	AT5G61500	MA_10436674g0010	shade>sun	autophagy-related (ATG) gene	(ATG3)	Atg8 ligase activity-GO:0019776,au	69.683972	0.9660809	0.30881053	3.128393683	0.001757646	0.020675147
PITA_000015366	Low quality	AT3G46640	MA_303578g0010	shade>sun	Encodes a myb family transcription factor	PHYTOCLOCK 1 (PCL1)	circadian rhythm-GO:0007623,DNA	41.504705	1.096623086	0.3339232	3.284057769	0.001023239	0.0128699
PITA_000015384	Low quality	AT1G06550	MA_105804g0010	shade<sun	ATP-dependent caseinolytic (Clp) protease/crotonase family protein;(source:		3-hydroxyisobutyryl-CoA hydrolase	280.14817	-1.029737441	0.26799267	-3.842408906	0.000121833	0.001996091
PITA_000015505	High quality	AT3G52970	MA_10432446g0010	shade<sun	member of CYP76G	CYTOCHROME P450, FAMILY 76, SI	chloroplast-GO:0009507,heme bin	101.94724	-2.884699252	0.6369078	-4.529225817	5.92E-06	0.00012128
PITA_000015531	High quality	AT4G00900	MA_104021g0010	shade>sun	Type IIA (SERCA-type) Ca2+ ATPase, catal	ER-TYPE CA2+-ATPASE 2 (ECA2)	ATPase activity-GO:0016887,calciu	26.2626	1.794159537	0.5747286	3.121750898	0.00179779	0.021050664
PITA_000015787	Low quality	AT5G36110	MA_896041g0010	shade<sun	Encodes a member of the CYP716A subfa	CYTOCHROME P450, FAMILY 716,	extracellular region-GO:0005576,h	419.85243	-1.610062581	0.36276117	-4.438354245	9.06E-06	0.000180089
PITA_000015795	High quality	AT3G17970	MA_10432470g0010	shade<sun	Integral chloroplast outer membrane pro	TRANSLOCON AT THE OUTER MEM	chloroplast-GO:0009507,chloropla:	119.09664	-0.627374407	0.20871503	-3.005889933	0.002648048	0.029304763
PITA_000015984	High quality	AT1G67340	MA_122700g0010	shade>sun	HCP-like superfamily protein with MYND-type zinc finger;(source:Araport11)		nucleus-GO:0005634,protein bindi	16.028796	1.476461287	0.49512977	2.981968306	0.002864016	0.031249141
PITA_000016124	High quality	AT3G28040	MA_36115g0010	shade>sun	Leucine-rich receptor-like protein kinase family protein;(source:Araport11)		kinase activity-GO:0016301,phosph	114.52095	10.58300869	1.29828259	8.151544821	3.59E-16	1.26E-13
PITA_000016168	High quality	AT2G33570	MA_2788g0010	shade<sun	glycosyltransferase family protein (DUF23	GALACTAN SYNTHASE 1 (GALS1)	beta-N-acetylglucosaminylglycopep	520.54133	-0.883882467	0.2042088	-4.328326966	1.50E-05	0.000287772
PITA_000016180	High quality	AT3G06060	MA_29607g0010	shade>sun	Encodes one of the Arabidopsis proteins (	TSC10A (TSC10A)	3-dehydrosphinganine reductase a	108.59441	1.053354693	0.2243166	4.695839154	2.66E-06	5.73E-05
PITA_000016189	High quality	AT5G01320	MA_12415g0010	shade>sun	Thiamine pyrophosphate dependent pyruvate decarboxylase family protein;(s		carboxy-lyase activity-GO:0016831	114.52095	10.58300869	1.29828259	8.151544821	3.59E-16	1.26E-13
PITA_000016359	Low quality	AT5G07040	MA_10021g0010	shade>sun	RING/U-box superfamily protein;(source:	ARABIDOPSIS T??XICOS EN LEVADU	nucleus-GO:0005634,protein ubiqu	53.61478	0.861163588	0.30662817	2.808494662	0.004977371	0.048991598
PITA_000016513	Low quality	AT4G37300	MA_29029g0010	shade>sun	maternal effect embryo arrest 59;(source	MATERNAL EFFECT EMBRYO ARRE!	cytosol-GO:0005829,embryo devel	34.035645	8.832345307	2.71456162	3.253691214	0.00113916	0.014126691
PITA_000016536	High quality	AT2G28470	MA_87238g0010	shade<sun	putative beta-galactosidase (BGAL8 gene)	BETA-GALACTOSIDASE 8 (BGAL8)	beta-galactosidase activity-GO:000	843.90122	-2.114296426	0.22740835	-9.297356108	1.44E-20	1.23E-17
PITA_000016546	High quality	AT5G05390	MA_10191230g0010	shade>sun	putative laccase, a member of laccase fa	LACCASE 12 (LAC12)	copper ion binding-GO:0005507,ex	30.581892	2.455717481	0.55953096	4.388885798	1.14E-05	0.000222547
PITA_000016598	Low quality	AT4G34340	MA_166792g0010	shade>sun	TBP-associated factor 8;(source:Araport1	TBP-ASSOCIATED FACTOR 8 (TAF8)	nucleus-GO:0005634,protein bindi	27.961785	1.617175713	0.54297644	2.978353388	0.002898016	0.031579746
PITA_000016656	High quality	AT4G22010	MA_10236360g0010	shade<sun	SKU5 similar 4;(source:Araport11)	SKU5 SIMILAR 4 (sks4)	copper ion binding-GO:0005507,ex	35.358761	-21.90137695	4.40877489	-4.967678662	6.78E-07	2.02E-05
PITA_000016722	High quality	AT1G65060	MA_109119g0010	shade<sun	encodes an isoform of 4-coumarate:CoA l	4-COUMARATE:COA LIGASE 3 (4CL	4-coumarate-CoA ligase activity-GC	244.38501	-1.861066921	0.32550178	-5.717532276	1.08E-08	7.65E-07
PITA_000016797	High quality	AT1G12570	MA_41268g0010	shade<sun	Ortholog of maize IPE1 gene which is involved in pollen exine development.		extracellular region-GO:0005576,fl	101.7481	-2.567159622	0.50516925	-5.081781231	3.74E-07	1.24E-05
PITA_000016872	High quality	AT1G08550	MA_10426812g0010	shade<sun	Violaxanthin deepoxidase involved in xan	NON-PHOTOCHEMICAL QUENCHIN	chlorophyll metabolic process-GO:(	1090.251	-0.852901433	0.2765999	-3.083520343	0.002045671	0.023449216
PITA_000016907	High quality	AT2G29760	MA_8607116g0010	shade<sun	Encodes a chloroplast RNA editing factor.	ORGANELLE TRANSCRIPT PROCESS	chloroplast-GO:0009507,chloropla:	249.20071	-1.137013173	0.21398342	-5.313557354	1.08E-07	5.19E-06
PITA_000016931	High quality	AT3G57430	MA_10429371g0010	shade<sun	Encodes a chloroplast RNA editing factor.	ORGANELLE TRANSCRIPT PROCESS	chloroplast-GO:0009507,chloropla:	165.25833	-1.653794814	0.56402817	-2.932113858	0.003366633	0.035610383
PITA_000017071	High quality	AT4G01650	MA_202526g0010	shade<sun	Polyketide cyclase / dehydrase and lipid transport protein;(source:Araport11)		biological_process_unknown-GO:0(	189.52892	-0.667372081	0.18593565	-3.589263689	0.000331613	0.004841478
PITA_000017081	High quality	AT3G54890	MA_10431493g0010	shade<sun	Encodes a component of the light harvest	PHOTOSYSTEM I LIGHT HARVESTIN	chlorophyll binding-GO:0016168,cl	1189.2399	-0.626559061	0.19766128	-3.169862348	0.001525112	0.018292768
PITA_000017096	High quality	AT2G26560	MA_468579g0010	shade<sun	Encodes a lipid acyl hydrolase with wide s	PHOSPHOLIPASE A 2A (PLA2A)	acylglycerol lipase activity-GO:0047	20.272356	-21.1416098	4.40979104	-4.794242992	1.63E-06	3.82E-05
PITA_000017574	High quality	AT2G33170	MA_10432079g0010	shade<sun	Leucine-rich repeat receptor-like protein kinase family protein;(source:Arapor		plasma membrane-GO:0005886,pr	35.033479	-21.88927922	4.40878685	-4.964921176	6.87E-07	2.02E-05
PITA_000017576	Low quality	AT2G21970	MA_118719g0010	shade<sun	stress enhanced protein 2 (SEP2) chloropl	STRESS ENHANCED PROTEIN 2 (SEF	cellular response to high light inten	282.48254	-0.550976182	0.152641	-3.609621042	0.000306645	0.004512963
PITA_000017726	Low quality	AT1G60500	MA_10431097g0010	shade<sun	Dynamin related protein 4C;(source:Arap	DYNAMIN RELATED PROTEIN 4C (D	axon-GO:0030424,cytoplasm-GO:0	36.796412	-8.447120807	1.4922556	-5.660639369	1.51E-08	1.01E-06
PITA_000017769	High quality	AT1G09850	MA_22695g0010	shade<sun	Arabidopsis thaliana papain-like cysteine	XYLEM BARK CYSTEINE PEPTIDASE	cysteine-type endopeptidase activit	391.31197	-2.018208397	0.1653185	-12.20800091	2.82E-34	7.21E-31
PITA_000017776	High quality	AT2G30860	MA_10142767g0010	shade<sun	Encodes glutathione transferase belongin	GLUTATHIONE S-TRANSFERASE PHI	apoplast-GO:0048046,chloroplast-(	572.75911	-2.670921193	0.66698039	-4.004497358	6.21E-05	0.001077163
PITA_000017778	High quality	AT2G30870	MA_137828g0010	shade<sun	early dehydration-induced gene ERD13 h	GLUTATHIONE S-TRANSFERASE PHI	apoplast-GO:0048046,cell wall-GO	592.00538	-2.796439962	0.78564775	-3.559406804	0.000371693	0.005365459
PITA_000017849	High quality	AT3G22600	MA_494876g0010	shade>sun	Glycosylphosphatidylinositol (GPI)-anchor	GLYCOSYLPHOSPHATIDYLINOSITOL	anchored component of membrane	84.266093	10.14065349	1.60555099	6.315995907	2.68E-10	2.81E-08
PITA_000017885	High quality	AT1G10020	MA_25897g0010	shade>sun	formin-like protein (DUF1005);(source:Araport11)		biological_process_unknown-GO:0(	34.679285	0.9445118	0.33328263	2.833966482	0.004597415	0.046028945
PITA_000017955	High quality	AT1G77670	MA_89872g0010	shade<sun	Pyridoxal phosphate (PLP)-dependent transferases superfamily protein;(sourc		biosynthetic process-GO:0009058,	169.45896	-0.625441464	0.18485231	-3.383465705	0.000715771	0.009472597
PITA_000017990	High quality	AT3G30390	MA_10432714g0010	shade<sun	Encodes a putative amino acid transporter.		amino acid transmembrane transp	60.714318	-22.53151824	4.4082046	-5.11126871	3.20E-07	1.18E-05

PITA_000018010	High quality	AT3G25560	MA_20016g0010	shade<sun	NSP-interacting kinase 2;(source:Araport1	NSP-INTERACTING KINASE 2 (NIK2)	plasma membrane-GO:0005886,pr	264.55224	-0.701576672	0.19651434	-3.570104273	0.000356839	0.005177301
PITA_000018011	High quality	AT1G15110	MA_459670g0010	shade>sun	PSS1 encodes a base-exchange-type Phos	PHOSPHATIDYLSERINE SYNTHASE 1	CDP-diacylglycerol-serine O-phosph	22.861201	1.34342191	0.43070899	3.119094181	0.00181408	0.021221997
PITA_000018066	High quality	AT5G17820	MA_4883828g0010	shade<sun	Peroxidase superfamily protein;(source:A	PEROXIDASE 57 (PER57)	cell wall-GO:0005618,extracellular	17.404771	-20.94419205	4.41018187	-4.749054039	2.04E-06	4.59E-05
PITA_000018068	High quality	AT1G12900	MA_63231g0010	shade>sun	glyceraldehyde 3-phosphate dehydrogena	GLYCERALDEHYDE 3-PHOSPHATE D	apoplast-GO:0048046,chloroplast-(	64.916136	1.037816409	0.33392749	3.107909473	0.001884158	0.02190173
PITA_000018264	High quality	AT1G34370	MA_10428315g0010	shade>sun	Encodes a putative nuclear Cys(2)His(2)-ty	SENSITIVE TO PROTON RHIZOTOXI	cellular response to oxygen levels-G	75.66788	1.270598611	0.37304576	3.406012737	0.000659191	0.008879834
PITA_000018395	High quality	AT4G15475	MA_62909g0010	shade>sun	Contributes to UV tolerance through nucl	RADIATION SENSITIVE 7B (RAD7B)	cytoplasm-GO:0005737,nucleotide	37.028354	1.07891182	0.33864693	3.185948956	0.001442801	0.017427941
PITA_000018476	High quality	AT4G03280	MA_733845g0010	shade<sun	Encodes the Rieske FeS center of cytochro	PHOTOSYNTHETIC ELECTRON TRAN	chloroplast-GO:0009507,chloropla:	913.08781	-0.840534054	0.29760307	-2.824346018	0.004737721	0.04709781
PITA_000018489	Low quality	AT2G25680	MA_155905g0010	shade<sun	Encodes a high-affinity molybdate transpo	MOLYBDATE TRANSPORTER 1 (MO	chloroplast-GO:0009507,mitochon	175.39895	-1.078346406	0.29527455	-3.652012737	0.000260193	0.003924041
PITA_000018490	High quality	AT2G38740	MA_10430307g0010	shade<sun	HAD-type phosphosugar phosphatase.	BROAD-RANGE SUGAR PHOSPHATI	cytoplasm-GO:0005737,cytosol-GC	331.8313	-1.360647514	0.2718417	-5.005293544	5.58E-07	1.76E-05
PITA_000018527	High quality	AT4G00905	MA_10429937g0010	shade<sun	NC domain-containing protein-like protein;(source:Araport11)		biological_process_unknown-GO:0(	115.9513	-0.789340701	0.21758583	-3.627721124	0.000285934	0.004255129
PITA_000018591	High quality	AT5G36930	MA_10427996g0010	shade>sun	Disease resistance protein (TIR-NBS-LRR class) family;(source:Araport11)		ADP binding-GO:0043531,cytoplas	122.33536	1.845999662	0.39877949	4.629123869	3.67E-06	7.75E-05
PITA_000018651	High quality	AT5G15780	MA_10429446g0010	shade>sun	Pollen Ole e 1 allergen and extensin family protein;(source:Araport11)		biological_process_unknown-GO:0(	134.45794	1.120173067	0.38595561	2.902336518	0.003703904	0.038478531
PITA_000018728	Low quality	AT1G80550	MA_21160g0010	shade<sun	Pentatricopeptide repeat (PPR) superfamily protein;(source:Araport11)		biological_process_unknown-GO:0(	202.34982	-0.989198426	0.32327999	-3.059881424	0.002214246	0.025167519
PITA_000018786	High quality	AT2G37240	MA_10426424g0010	shade<sun	Thioredoxin superfamily protein;(source:Araport11)		antioxidant activity-GO:0016209,ch	846.67432	-0.694443017	0.17880803	-3.883735073	0.000102864	0.001703843
PITA_000018796	High quality	AT4G26180	MA_10433305g0010	shade<sun	Encodes a mitochondrial CoA transporter	COA CARRIER 2 (COAC2)	coenzyme A transmembrane transp	543.43027	-1.0044993	0.24490586	-4.101573183	4.10E-05	0.00073433
PITA_000018799	Low quality	AT3G07270	MA_102960g0010	shade<sun	GTP cyclohydrolase I;(source:Araport11)		7,8-dihydroneopterin 3'-triphospha	159.90918	-0.796861983	0.20883192	-3.815805429	0.000135739	0.002194454
PITA_000018802	High quality	AT5G25460	MA_54830g0010	shade>sun	Encodes a DUF642 cell wall protein.	DUF642 L-GALL RESPONSIVE GENE	extracellular region-GO:0005576,m	49.80389	1.899497315	0.47492323	3.999588155	6.35E-05	0.001097905
PITA_000018803	High quality	AT2G34510	MA_44504g0020	shade>sun	Protein of unknown function, DUF642. Fo	(ATHB-1)	anchored component of membrane	22.607413	2.660848448	0.58631067	4.53829103	5.67E-06	0.000116369
PITA_000018953	Low quality	AT2G38780	MA_10435866g0010	shade<sun	cytochrome C oxidase subunit;(source:Araport11)		biological_process_unknown-GO:0(	465.35151	-0.859305776	0.28741325	-2.989791757	0.002791677	0.030655774
PITA_000018993	High quality	AT5G45910	MA_76943g0010	shade<sun	GDSL-motif esterase/acyltransferase/lipase. Enzyme group with broad substr		hydrolase activity, acting on ester b	1149.6304	-3.239710341	0.5080541	-6.376703419	1.81E-10	2.01E-08
PITA_000019077	High quality	AT2G46470	MA_10427651g0010	shade<sun	inner membrane protein OXA1-like protei	INNER MEMBRANE PROTEIN OXA1	integral component of mitochondri	106.72336	-0.697379872	0.22299296	-3.127362688	0.001763822	0.020709744
PITA_000019181	Low quality	AT4G24210	MA_233275g0010	shade>sun	F-box protein that is involved in GA signal	SLEEPY1 (SLY1)	gibberellic acid mediated signaling	54.942684	9.523594586	2.42088245	3.933935159	8.36E-05	0.001410657
PITA_000019340	High quality	AT5G54250	MA_175873g0010	shade>sun	member of Cyclic nucleotide gated chann	CYCLIC NUCLEOTIDE-GATED CATIO	calmodulin binding-GO:0005516,ca	28.447507	1.528033837	0.42102362	3.629330431	0.000284157	0.004235565
PITA_000019363	High quality	AT5G14540	MA_10428704g0010	shade>sun	basic salivary proline-rich-like protein (DU	FLOE2 (FLOE2)	biological_process_unknown-GO:0(	61.611132	0.788837256	0.25892656	3.046567508	0.002314705	0.026158413
PITA_000019441	High quality	AT1G79220	MA_104598g0010	shade<sun	Mitochondrial transcription termination f	(MTERF17)	chloroplast-GO:0009507,chloropla:	31.530131	-21.74010696	4.4089398	-4.930914897	8.18E-07	2.34E-05
PITA_000019455	High quality	AT1G69550	MA_97184g0010	shade>sun	disease resistance protein (TIR-NBS-LRR class);(source:Araport11)		ADP binding-GO:0043531,cellular_	23.398956	1.893301239	0.58717135	3.224444188	0.001262175	0.01548719
PITA_000019532	High quality	AT5G46110	MA_10428353g0020	shade<sun	Encodes a chloroplast triose phosphate /	ACCLIMATION OF PHOTOSYNTHES	antiporter activity-GO:0015297,chl	166.90319	-0.680038508	0.19332197	-3.517647394	0.00043539	0.006180421
PITA_000019626	Low quality	AT4G11910	MA_10433870g0010	shade<sun	Acts antagonistically with SGR1 to balanc	NONYELLOWING 2 (NYE2)	chloroplast-GO:0009507,negative r	244.6544	-0.538025541	0.18640178	-2.886375529	0.003897069	0.040030539
PITA_000019775	High quality	AT5G08650	MA_742675g0010	shade<sun	Critical for chloroplast protein synthesis (	(CPLEPA)	chloroplast-GO:0009507,mRNA bir	113.3207	-2.856787062	0.47206708	-6.051654878	1.43E-09	1.26E-07
PITA_000019802	High quality	AT2G16430	MA_10430628g0010	shade<sun	Encodes an acid phosphatase involved pla	PURPLE ACID PHOSPHATASE 10 (P/	acid phosphatase activity-GO:0003	201.38785	-0.969473899	0.26780731	-3.620042695	0.000294554	0.004357567
PITA_000019805	High quality	AT2G40260	MA_84935g0010	shade<sun	Homeodomain-like superfamily protein;(source:Araport11)		DNA-binding transcription factor ac	18.486728	-7.453730308	1.69351397	-4.401339722	1.08E-05	0.000211275
PITA_000019890	High quality	AT5G28840	MA_10426186g0010	shade<sun	Encodes a protein with GDP-D-mannose 3	GDP-D-MANNOSE 3',5'-EPIMERASE	cytosol-GO:0005829,GDP-mannose	232.36805	-1.250355191	0.42568928	-2.93724846	0.003311387	0.035166925
PITA_000019993	High quality	AT2G37420	MA_10434146g0010	shade<sun	ATP binding microtubule motor family protein;(source:Araport11)		ATP-dependent microtubule motor	17.679381	-20.92872422	4.41014042	-4.745591347	2.08E-06	4.59E-05
PITA_000020176	Low quality	AT4G02500	MA_10428947g0010	shade>sun	Encodes a protein with xylosyltransferase	UDP-XYLOSYLTRANSFERASE 2 (XT2)	cytosol-GO:0005829,endosome-GC	216.04494	24.71425455	4.32820048	5.710053092	1.13E-08	7.81E-07
PITA_000020294	Low quality	AT5G56600	MA_17008g0010	shade<sun	Encodes profilin3, a low-molecular weigh	PROFILIN 3 (PRF3)	actin filament polymerization-GO:0	15.445257	-7.194036788	1.84986811	-3.888945782	0.000100681	0.001673088
PITA_000020313	High quality	AT3G07130	MA_618219g0010	shade<sun	Encodes PAP15, a purple acid phosphatas	PURPLE ACID PHOSPHATASE 15 (P/	acid phosphatase activity-GO:0003	389.90362	-1.079453307	0.24356727	-4.431848698	9.34E-06	0.000185467
PITA_000020331	High quality	AT4G21490	MA_10437271g0010	shade<sun	NAD(P)H dehydrogenase B3;(source:Arap	NAD(P)H DEHYDROGENASE B3 (ND	calcium ion binding-GO:0005509,m	110.15194	-0.704176313	0.23478132	-2.999285886	0.002706133	0.029850706
PITA_000020469	High quality	AT5G51780	MA_14544g0010	shade<sun	basic helix-loop-helix (bHLH) DNA-binding	(BHLH)	DNA-binding transcription factor ac	24.285727	-21.33972849	4.40939647	-4.839603026	1.30E-06	3.24E-05
PITA_000020512	Low quality	AT3G13770	MA_122546g0010	shade<sun	Pentatricopeptide repeat (PPR) superfamily protein;(source:Araport11)		intracellular membrane-bounded o	360.91823	-1.15330782	0.3508598	-3.28709022	0.001012284	0.012757169
PITA_000020691	High quality	AT3G49260	MA_6756408g0010	shade<sun	IQ-domain 21;(source:Araport11)	IQ-DOMAIN 21 (iqd21)	biological_process_unknown-GO:0(	52.550218	-22.4390271	4.4083279	-5.09014474	3.58E-07	1.19E-05
PITA_000021021	High quality	AT1G11080	MA_118714g0010	shade>sun	serine carboxypeptidase-like 31;(source:A	SERINE CARBOXYPEPTIDASE-LIKE 3	extracellular region-GO:0005576,se	21.415685	1.9131603	0.64890639	2.948283948	0.003195434	0.034223621
PITA_000021151	High quality	AT1G22990	MA_10428986g0010	shade<sun	Heavy metal transport/detoxification sup	HEAVY METAL ASSOCIATED ISOPRE	cadmium ion homeostasis-GO:005!	2311.5647	-2.595679878	0.45086804	-5.757072242	8.56E-09	6.17E-07
PITA_000021167	High quality	AT2G38240	MA_12129g0010	shade>sun	One of 4 paralogs encoding a 2-oxoglutar	JASMONATE-INDUCED OXYGENASE	cytoplasm-GO:0005737,cytosol-GC	30.392775	1.28686404	0.43906831	2.93089709	0.003379847	0.035735396
PITA_000021184	High quality	AT5G42390	MA_298732g0010	shade<sun	Encodes a chloroplast-localized metallopr	STROMAL PROCESSING PEPTIDASE	chloroplast-GO:0009507,chloropla:	231.46305	-0.953249443	0.25722495	-3.705898077	0.000210643	0.003267481
PITA_000021201	High quality	AT5G51260	MA_131674g0010	shade<sun	HAD superfamily, subfamily IIIB acid phosphatase;(source:Araport11)		acid phosphatase activity-GO:0003	372.00118	-1.159311586	0.3442525	-3.36761991	0.0007582	0.00994672
PITA_000021229	Low quality	AT4G26500	MA_181487g0010	shade<sun	Sulfur acceptor that interacts with and ac	CHLOROPLAST SULFUR E (CPSUFE)	chloroplast-GO:0009507,chloropla:	344.87745	-0.792385603	0.21294729	-3.721041004	0.000198403	0.003088577
PITA_000021275	High quality	AT1G68650	MA_22128g0010	shade<sun	Member of the UPF0016 family of memb	PHOTOSYNTHESIS-AFFECTED MUT/	calcium ion transmembrane transp	245.05986	-0.899827149	0.16415603	-5.481535727	4.22E-08	2.23E-06
PITA_000021420	High quality	AT2G06050	MA_10427053g0010	shade<sun	Encodes a 12-oxophytodienoate reductas	OXOPHYTODIENOATE-REDUCTASE	12-oxophytodienoate reductase act	4199.9829	-4.874758155	0.51547031	-9.456913488	3.17E-21	3.12E-18
PITA_000021458	Low quality	AT4G35490	MA_24522g0020	shade<sun	mitochondrial ribosomal protein L11;(sou	MITOCHONDRIAL RIBOSOMAL PRC	large ribosomal subunit-GO:00159:	52.550218	-22.4390271	4.4083279	-5.09014474	3.58E-07	1.19E-05
PITA_000021466	High quality	AT4G27410	MA_75192g0010	shade>sun	Encodes a NAC transcription factor induce	RESPONSIVE TO DESICCATION 26 (	DNA binding-GO:0003677,DNA-bin	12.840396	2.236312659	0.67969211	3.290184838	0.001001216	0.012667558
PITA_000021473	High quality	AT2G32300	MA_163393g0010	shade>sun	Encodes a uclacyanin, a protein precursor	UCLACYANIN 1 (UCC1)	anchored component of membrane	41.296736	1.114869525	0.34836598	3.20028241	0.00137293	0.016670455
PITA_000021530	High quality	AT2G43790	MA_10437020g0010	shade<sun	Encodes a MAP kinase induced by pathog	MAP KINASE 6 (MPK6)	abscisic acid-activated signaling pat	26.275109	-21.49994244	4.40924566	-4.876104457	1.08E-06	2.80E-05
PITA_000021645	High quality	AT2G38120	MA_10293595g0010	shade<sun	Encodes an auxin influx transporter. AUX	AUXIN RESISTANT 1 (AUX1)	amino acid transmembrane transp	170.48416	-0.843010388	0.24534036	-3.436085296	0.000590185	0.008030532
PITA_000021755	High quality	AT5G59610	MA_10428997g0010	shade<sun	Chaperone DnaJ-domain superfamily prot	DNA J PROTEIN C73 (DJC73)	chaperone cofactor-dependent pro	220.33476	-0.610241789	0.16596564	-3.676916355	0.00023607	0.003600531
PITA_000021762	High quality	AT5G07280	MA_37224g0010	shade<sun	Encodes EMS1 (EXCESS MICROSPOROCYT	EXCESS MICROSPOROCYTES1 (EMS	anther wall tapetum cell fate specif	51.2083	-8.926585773	1.61126081	-5.54012466	3.02E-08	1.78E-06
PITA_000021795	High quality	AT4G11070	MA_6177g0010	shade>sun	member of WRKY Transcription Factor; G	(WRKY41)	DNA-binding transcription factor ac	29.484246	8.625708946	2.53905913	3.397206804	0.000680775	0.009113061
PITA_000021801	Low quality	AT1G64160	MA_8342156g0010	shade<sun	Encodes a dirigent protein involved in the	DIRIGENT PROTEIN 5 (DIR5)	(-)-pinoresinol biosynthetic process	366.86278	-2.96905393	0.75152876	-3.950685689	7.79E-05	0.001323322
PITA_000021824	Low quality	AT2G36690	MA_10433348g0010	shade<sun	Protein belonging to the Fe-dependent 2-	GERMINATION INSENSITIVE TO AB	2-oxoglutarate-dependent dioxyger	103.69758	-1.351591675	0.39972227	-3.381326929	0.000721367	0.009541713
PITA_000021863	High quality	AT5G53390	MA_18089g0010	shade>sun	Encodes a member of the bifunctional wa	FOLDED PETAL 1 (FOP1)	diacylglycerol O-acyltransferase act	28.630238	8.582963228	1.47746441	5.809252097	6.28E-09	4.69E-07
PITA_000021922	High quality	AT5G35530	MA_120861g0010	shade<sun	Ribosomal protein S3 family protein;(source:Araport11)		cytosol-GO:0005829,cytosolic ribos	197.27063	-0.735419732	0.19890278	-3.697382911	0.000217834	0.003366496
PITA_000021944	High quality	AT5G21090	MA_10430827g0010	shade<sun	Leucine-rich repeat (LRR) family protein;(source:Araport11)		signal transduction-GO:0007165	58.299469	-9.116818806	1.59714208	-5.708207766	1.14E-08	7.84E-07
PITA_000021945	High quality	AT1G53920	MA_17845g0010	shade<sun	Contains lipase signature motif and GDSL	GDSL-MOTIF LIPASE 5 (GLIP5)	lipase activity-GO:0016298,mitoch	722.79006	-5.456101781	1.92055402	-2.840899936	0.004498643	0.045265713
PITA_000022109	High quality	AT5G22020	MA_12783g0010	shade<sun	Calcium-dependent phosphotriesterase superfamily protein;(source:Araport1		biosynthetic process-GO:0009058,(	236.9123	-0.883186268	0.24330779	-3.629913623	0.000283516	0.004228468
PITA_000022137	High quality	AT1G09700	MA_10152247g0010	shade<sun	Encodes a nuclear dsRNA binding protein	HYPONASTIC LEAVES 1 (HYL1)	double-stranded RNA binding-GO:0	183.36902	-10.76791152	1.68657219	-6.384494894	1.72E-10	1.91E-08
PITA_000022177	High quality	AT1G71697	MA_101657g0010	shade<sun	Encodes choline kinase. mRNA levels are	CHOLINE KINASE 1 (CK1)	choline kinase activity-GO:0004103	240.10263	-1.0698016	0.36962072	-2.894322561	0.003799776	0.03917257
PITA_000022181	High quality	AT2G18950	MA_10436616g0010	shade<sun	Encodes homogentisate phytyltransferase	HOMOGENTISATE PHYTYLTRANSFE	cell wall organization-GO:0071555,	304.99088	-1.169870486	0.19204792	-6.091555065	1.12E-09	1.01E-07
PITA_000022209	High quality	AT4G25000	MA_30833g0020	shade>sun	Predicted to be secreted protein based or	ALPHA-AMYLASE-LIKE (AMY1)	alpha-amylase activity-GO:000455(	31.649895	1.963861304	0.41939255	4.682632755	2.83E-06	6.09E-05
PITA_000022338	High quality	AT3G12770	MA_10355730g0010	shade<sun	Encodes a pentatricopeptide repeat prote	MITOCHONDRIAL EDITING FACTOR	chloroplast-GO:0009507,mitochon	184.40211	-1.182149328	0.28319877	-4.174274292	2.99E-05	0.000550253
PITA_000022428	High quality	AT4G00660	MA_7069g0010	shade<sun	RNAhelicase-like 8;(source:Araport11)	RNAHELICASE-LIKE 8 (RH8)	cytoplasm-GO:0005737,cytoplasmi	155.02927	-0.57174677	0.19370522	-2.951633239	0.003160981	0.033941705
PITA_000022498	High quality	AT3G14930	MA_10435805g0010	shade<sun	Uroporphyrinogen decarboxylase;(source	(HEME1)	chlorophyll biosynthetic process-G(	1294.2113	-1.807098152	0.37796529	-4.781121926	1.74E-06	4.05E-05
PITA_000022505	High quality	AT1G14190	MA_137064g0010	shade<sun	Glucose-methanol-choline (GMC) oxidoreductase family protein;(source:Arap		chloroplast-GO:0009507,flavin ade	209.08432	-0.724167953	0.21376379	-3.387701772	0.000704809	0.009361378
PITA_000022587	High quality	AT2G27610	MA_20782g0010	shade<sun	Tetratricopeptide repeat (TPR)-like superfamily protein;(source:Araport11)		intracellular membrane-bounded o	469.08418	-1.800747755	0.2024002	-8.896966453	5.74E-19	3.58E-16
PITA_000022609	Low quality	AT3G49950	MA_94287g0010	shade>sun	GRAS family transcription factor;(source:Araport11)		DNA-binding transcription factor ac	64.531408	0.878431358	0.27296667	3.218090174	0.001290472	0.015804121
PITA_000022625	High quality	AT3G63410	MA_215523g0010	shade<sun	Encodes a MPBQ/MSBQ methyltransfera:	ALBINO OR PALE GREEN MUTANT :	2-methyl-6-geranylgeranyl-1,4-ben	337.2961	-0.917770573	0.16596471	-5.52991387	3.20E-08	1.87E-06
PITA_000022636	Low quality	AT1G05350	MA_10427922g0010	shade<sun	NAD(P)-binding Rossmann-fold superfamily protein;(source:Araport11)		cytoplasm-GO:0005737,cytosol-GC	124.38514	-2.131168034	0.54862004	-3.88459747	0.0001025	0.001698905
PITA_000022694	High quality	AT2G39650	MA_659011g0010	shade>sun	cruciferin (DUF506);(source:Araport11)		biological_process_unknown-GO:0(	38.607738	1.823592801	0.52052511	3.503371419	0.000459408	0.00647814
PITA_000022734	High quality	AT3G19540	MA_10431995g0010	shade<sun	glutamyl-tRNA (Gln) amidotransferase su	BOUNDARY OF ROP DOMAIN4 (BD	biological_process_unknown-GO:0(	60.714318	-22.53151824	4.4082046	-5.11126871	3.20E-07	1.18E-05
PITA_000022742	High quality	AT4G24800	MA_10693g0010	shade>sun	MA3 domain-containing protein;(source:/	EIN2 C-TERMINUS INTERACTING PI	cytoplasm-GO:0005737,cytosol-GC	33.356343	1.471765986	0.36004703	4.087704786	4.36E-05	0.00077637
PITA_000022751	High quality	AT5G11520	MA_72677g0010	shade<sun	Encodes the chloroplastic isozyme of aspa	ASPARTATE AMINOTRANSFERASE :	2-oxoglutarate metabolic process-G	300.34049	-1.125071053	0.17827493	-6.310876585	2.77E-10	2.86E-08
PITA_000022824	Low quality	AT5G51190	MA_10031781g0010	shade>sun	encodes a member of the ERF (ethylene r	ETHYLENE RESPONSE FACTOR 105	DNA-binding transcription factor ac	69.834529	1.557671059	0.51512881	3.023847694	0.002495821	0.027921788
PITA_000022967	High quality	AT1G23820	MA_17812g0010	shade<sun	Spermidine synthase.	SPERMIDINE SYNTHASE 1 (SPDS1)	chloroplast-GO:0009507,cytosol-G(	148.57231	-0.889961539	0.22387216	-3.975311286	7.03E-05	0.00120557
PITA_000023013	Low quality	AT2G43950	MA_104060g0010	shade<sun	Constitutes a peptide sensitive ion chann	CHLOROPLAST OUTER ENVELOPE F	cation transport-GO:0006812,chlor	221.39852	-0.826012833	0.1731111	-4.771576346	1.83E-06	4.23E-05
PITA_000023015	High quality	AT4G39090	MA_9992178g0010	shade<sun	Similar to cysteine proteinases, induced b	RESPONSIVE TO DEHYDRATION 19	cysteine-type endopeptidase activit	20.238106	-21.14561367	4.4097939	-4.795147836	1.63E-06	3.81E-05
PITA_000023020	High quality	AT4G31540	MA_482507g0010	shade<sun	A member of EXO70 gene family, putative	EXOCYST SUBUNIT EXO70 FAMILY	cytoplasm-GO:0005737,cytosol-GC	124.27592	-1.496962865	0.21587653	-6.934347389	4.08E-12	6.45E-10
PITA_000023021	High quality	AT3G02690	MA_8499291g0010	shade<sun	Nucleotide/sugar transporter family protein		chloroplast-GO:0009507,integral co	462.84057	-0.972981548	0.25997469	-3.742601021	0.000182125	0.002868305
PITA_000023032	High quality	AT5G49720	MA_102312g0010	shade<sun	Encodes a membrane-bound endo-1,4-be	GLYCOSYL HYDROLASE 9A1 (GH9A:	cell plate-GO:0009504,cellulase act	646.77418	-12.58651507	1.87876127	-6.699369029	2.09E-11	2.88E-09
PITA_000023131	Low quality	AT2G31840	MA_875929g0010	shade<sun	Thioredoxin superfamily protein;(source:/	MESOPHYLL-CELL RNAI LIBRARY LII	chloroplast-GO:0009507,chloropla:	308.77082	-0.886206528	0.27700365	-3.199259436	0.001377811	0.016713875
PITA_000023242	Low quality	AT1G54200	MA_63144g0010	shade>sun	DNA mismatch repair Msh6-like protein;(	BIG GRAIN 3 (BG3)	auxin transport-GO:0060918,nucle	78.513461	10.03834398	2.08477203	4.815079944	1.47E-06	3.59E-05
PITA_000023291	High quality	AT2G35930	MA_481299g0010	shade<sun	Encodes a cytoplasmically localized U-box	PLANT U-BOX 23 (PUB23)	cytoplasm-GO:0005737,cytosol-GC	26.275109	-21.49994244	4.40924566	-4.876104457	1.08E-06	2.80E-05
PITA_000023415	High quality	AT3G02450	MA_10436247g0010	shade<sun	Proteolytically inactive member of the Fts	FTSH (FILAMENTATION-TEMPERAT	ATP-dependent peptidase activity-(	136.64857	-0.896699824	0.20240256	-4.430279165	9.41E-06	0.000186677
PITA_000023599	High quality	AT2G23790	MA_16420g0020	shade<sun	calcium uniporter (DUF607);(source:Araport11)		calcium channel activity-GO:00052	26.275109	-21.49994244	4.40924566	-4.876104457	1.08E-06	2.80E-05
PITA_000023652	High quality	AT4G16310	MA_10435906g0020	shade<sun	FAD-dependent lysine-specific histone de	LSD1-LIKE 3 (LDL3)	mitochondrion-GO:0005739,oxidor	39.412664	-22.04498447	4.40863388	-5.000411702	5.72E-07	1.79E-05
PITA_000023659	High quality	AT5G61210	MA_136883g0010	shade<sun	membrane localized t-SNARE SNAP25 hor	SOLUBLE N-ETHYLMALEIMIDE-SEN:	cell plate-GO:0009504,cytokinesis l	954.61382	-0.885519798	0.15100872	-5.864030751	4.52E-09	3.53E-07
PITA_000023892	Low quality	AT5G47690	MA_5897151g0010	shade<sun	One of 5 PO76/PDS5 cohesion cofactor o	(PDS5A)	chromatin-GO:0000785,cytosol-GC	107.91656	-0.828930988	0.26396965	-3.140251122	0.001688031	0.019994028
PITA_000023933	High quality	AT2G01820	MA_10431591g0010	shade<sun	Transmembrane kinase (TMK), member c	TRANSMEMBRANE KINASE 3 (TMK	plant-type cell wall-GO:0009505,pl	99.368409	-0.778939551	0.23683475	-3.288958053	0.00100559	0.012691548
PITA_000023966	High quality	AT4G31770	MA_10435439g0010	shade<sun	Encodes a RNA lariat debranching enzym	DEBRANCHING ENZYME 1 (DBR1)	embryo development ending in see	52.550218	-22.4390271	4.4083279	-5.09014474	3.58E-07	1.19E-05
PITA_000024037	High quality	AT5G05340	MA_10435976g0010	shade>sun	Encodes a protein with sequence similarit	PEROXIDASE 52 (PRX52)	apoplast-GO:0048046,cell wall-GO	72.444721	0.956276409	0.34062405	2.807424831	0.004993933	0.049111141
PITA_000024038	High quality	AT3G07990	MA_45108g0010	shade<sun	serine carboxypeptidase-like 27;(source:A	SERINE CARBOXYPEPTIDASE-LIKE 2	extracellular region-GO:0005576,se	1136.7452	-1.225671906	0.3704501	-3.308601939	0.00093763	0.011975481
PITA_000024052	Low quality	AT3G54000	MA_213656g0010	shade>sun	TIP41-like protein;(source:Araport11)		biological_process_unknown-GO:0(	28.316827	1.167752327	0.36843167	3.169522132	0.001526898	0.018297057
PITA_000024111	High quality	AT4G27230	MA_191819g0010	shade>sun	Encodes HTA2, a histone H2A protein.	HISTONE H2A 2 (HTA2)	chromatin silencing-GO:0006342,c	114.52095	10.58300869	1.29828259	8.151544821	3.59E-16	1.26E-13
PITA_000024242	Low quality	AT2G32980	MA_10427389g0010	shade<sun	HAUS augmin-like complex subunit;(sourc	AUGMIN SUBUNIT 2 (AUG2)	cytokinesis by cell plate formation-(	2539.6477	-1.317669553	0.34635666	-3.804371949	0.000142164	0.002291093
PITA_000024245	High quality	AT4G17560	MA_947876g0010	shade<sun	Ribosomal protein L19 family protein;(source:Araport11)		chloroplast-GO:0009507,chloropla:	456.4102	-0.806629935	0.21295592	-3.787778858	0.000152	0.002437331
PITA_000024269	High quality	AT3G13120	MA_10432148g0010	shade<sun	Ribosomal protein S10p/S20e family prot	PLASTID RIBOSOMAL PROTEIN OF	chloroplast-GO:0009507,chloropla:	581.53939	-0.744612331	0.23690992	-3.143018787	0.001672151	0.019851852
PITA_000024299	High quality	AT5G38890	MA_63326g0010	shade>sun	Nucleic acid-binding, OB-fold-like protein;(source:Araport11)		cytoplasm-GO:0005737,nuclear ex	246.2998	11.68788242	2.44063363	4.788872145	1.68E-06	3.92E-05
PITA_000024301	Low quality	AT4G01026	MA_14451g0010	shade<sun	Encodes a member of the PYR (pyrabactir	PYR1-LIKE 7 (PYL7)	abscisic acid binding-GO:0010427,a	316.82154	-2.381753925	0.35795846	-6.653716042	2.86E-11	3.71E-09
PITA_000024614	High quality	AT5G21482	MA_32541g0010	shade<sun	This gene used to be called AtCKX5. It enc	CYTOKININ OXIDASE 7 (CKX7)	cytokinin catabolic process-GO:000	202.13825	-4.867392985	1.6799747	-2.897301357	0.00376388	0.038925754
PITA_000024661	Low quality	AT1G65910	MA_18975g0010	shade>sun	NAC domain containing protein 28;(sourc	NAC DOMAIN CONTAINING PROTE	DNA binding-GO:0003677,DNA-bin	432.08988	25.50658086	4.32809423	5.89325914	3.79E-09	3.06E-07
PITA_000024847	High quality	AT3G12750	MA_9267623g0010	shade<sun	A member of Zrt- and Irt-related protein (	ZINC TRANSPORTER 1 PRECURSOR	membrane-GO:0016020,plasma m	35.358761	-21.90137695	4.40877489	-4.967678662	6.78E-07	2.02E-05
PITA_000025013	High quality	AT2G36190	MA_10380652g0010	shade<sun	cwINV4 appears to function as a cell wall-	CELL WALL INVERTASE 4 (cwINV4)	cell wall-GO:0005618,nectar secret	17.516739	-20.95349401	4.41016289	-4.751183692	2.02E-06	4.57E-05
PITA_000025149	High quality	AT4G23160	MA_3721g0010	shade<sun	Encodes a cysteine-rich receptor-like prot	CYSTEINE-RICH RLK (RECEPTOR-LIK	ATP binding-GO:0005524,defense r	30.357159	-21.69383454	4.40899933	-4.920353335	8.64E-07	2.39E-05
PITA_000025219	High quality	AT3G23920	MA_124514g0010	shade<sun	Encodes a chloroplast beta-amylase. Is ne	BETA-AMYLASE 1 (BAM1)	amylopectin maltohydrolase activit	267.27333	-0.556753203	0.14971552	-3.718740696	0.000200218	0.003114942
PITA_000025263	High quality	AT5G06710	MA_194299g0010	shade>sun	Homeobox-leucine zipper protein.	HOMEOBOX FROM ARABIDOPSIS T	DNA-binding transcription factor ac	55.635855	9.541728602	1.97435257	4.83283925	1.35E-06	3.32E-05
PITA_000025316	High quality	AT4G00730	MA_93471g0010	shade>sun	Encodes a homeodomain protein of the H	ANTHOCYANINLESS 2 (ANL2)	anthocyanin accumulation in tissue	38.573342	1.787306409	0.44180076	4.045503235	5.22E-05	0.000921444
PITA_000025324	High quality	AT1G70250	MA_10436581g0010	shade>sun	Encodes a Protease inhibitor/seed storag	GLYCOSYLPHOSPHATIDYLINOSITOL	cellular_component_unknown-GO:	33.041858	2.525424885	0.65149018	3.876382097	0.000106021	0.001752737
PITA_000025460	High quality	AT4G34050	MA_9065834g0010	shade<sun	Methyltransferase in the lignin biosynthet	CAFFEOYL COENZYME A O-METHYI	caffeoyl-CoA O-methyltransferase a	506.58406	-1.363341043	0.1793877	-7.599969388	2.96E-14	7.82E-12
PITA_000025526	High quality	AT5G42800	MA_79460g0010	shade<sun	dihydroflavonol reductase. Catalyzes the	DIHYDROFLAVONOL 4-REDUCTASE	anthocyanin-containing compound	217.15492	-1.80607969	0.27301904	-6.615215195	3.71E-11	4.75E-09
PITA_000025543	High quality	AT4G27600	MA_159115g0010	shade<sun	Encodes a phosphofructokinase B-type ca	GENES NECESSARY FOR THE ACHIE	chloroplast-GO:0009507,phosphot	2000.2913	-1.398583581	0.22867775	-6.115958334	9.60E-10	8.87E-08
PITA_000025634	High quality	AT1G74970	MA_18349g0020	shade<sun	ribosomal protein S9, nuclear encoded co	RIBOSOMAL PROTEIN S9 (RPS9)	chloroplast-GO:0009507,chloropla:	468.46191	-0.769249814	0.16130256	-4.768986897	1.85E-06	4.28E-05
PITA_000025647	High quality	AT3G19940	MA_457005g0010	shade>sun	Encodes a hexose-H(+) symporter that cat	SUGAR TRANSPORT PROTEIN 10 (S	carbohydrate transmembrane tran	20.678168	1.356157065	0.44983589	3.014781855	0.002571641	0.028584518
PITA_000025670	High quality	AT3G48730	MA_10085364g0010	shade<sun	glutamate-1-semialdehyde 2,1-aminomut	GLUTAMATE-1-SEMIALDEHYDE 2,1	chlorophyll biosynthetic process-G(	475.23753	-0.560323489	0.18953154	-2.956360089	0.003112934	0.033536399
PITA_000025831	High quality	AT5G21105	MA_19018g0010	shade<sun	Plant L-ascorbate oxidase;(source:Araport11)		cell wall-GO:0005618,copper ion bi	248.84781	-1.086105472	0.27949714	-3.885927002	0.00010194	0.001690879
PITA_000025836	High quality	AT1G74030	MA_10436771g0010	shade<sun	Encodes the plastid-localized phosphoenc	ENOLASE 1 (ENO1)	chloroplast-GO:0009507,chloropla:	171.275	-0.967708355	0.27596848	-3.506590134	0.000453888	0.00641562
PITA_000025876	High quality	AT3G55240	MA_47319g0010	shade>sun	Overexpression leads to PEL (Pseudo-Etiolation in Light) phenotype.		chloroplast-GO:0009507,developm	11.069477	3.631153643	0.81490701	4.455911638	8.35E-06	0.000166736
PITA_000025912	Low quality	AT1G16880	MA_853979g0010	shade<sun	Encodes a ACT domain-containing proteir	ACT DOMAIN REPEATS 11 (ACR11)	chloroplast-GO:0009507,chloropla:	337.51039	-0.875245967	0.13247789	-6.60673246	3.93E-11	4.93E-09
PITA_000025923	High quality	AT3G47090	MA_168077g0010	shade<sun	Leucine-rich repeat protein kinase family protein;(source:Araport11)		chloroplast-GO:0009507,kinase act	60.714318	-22.53151824	4.4082046	-5.11126871	3.20E-07	1.18E-05
PITA_000026003	High quality	AT1G28440	MA_113767g0010	shade>sun	HAESA-like 1;(source:Araport11)	HAESA-LIKE 1 (HSL1)	kinase activity-GO:0016301,phosph	56.177395	9.555733659	1.66620304	5.73503555	9.75E-09	6.93E-07
PITA_000026020	High quality	AT5G15780	MA_73048g0010	shade<sun	Pollen Ole e 1 allergen and extensin family protein;(source:Araport11)		biological_process_unknown-GO:0(	485.80603	-1.357335359	0.41504858	-3.270304816	0.001074317	0.013446284
PITA_000026026	High quality	AT4G30620	MA_165748g0020	shade<sun	Homolog of STIC2, recent duplication.	STIC2 LIKE (STCL)	biological_process_unknown-GO:0(	213.19333	-0.590911547	0.17111984	-3.453203067	0.000553972	0.007590214
PITA_000026292	Low quality	AT2G17530	MA_10321423g0010	shade>sun	Protein kinase superfamily protein;(source:Araport11)		cytoplasm-GO:0005737,intracellula	108.02247	23.75154617	4.32841291	5.487356833	4.08E-08	2.16E-06
PITA_000026312	High quality	AT4G21990	MA_16080g0010	shade<sun	Encodes a protein disulfide isomerase-like	APS REDUCTASE 3 (APR3)	adenylyl-sulfate reductase (glutathi	640.46335	-1.457132623	0.26882952	-5.420285012	5.95E-08	3.04E-06
PITA_000026326	High quality	AT3G09040	MA_192667g0010	shade<sun	Pentatricopeptide repeat (PPR) superfami	MITOCHONDRIAL RNA EDITING FA	intracellular membrane-bounded o	116.01977	-1.812974293	0.43837378	-4.135681447	3.54E-05	0.000640024
PITA_000026395	High quality	AT3G28345	MA_107576g0010	shade<sun	Encodes an ATP-binding cassette (ABC) tr	ATP-BINDING CASSETTE B15 (ABCB	ATPase activity-GO:0016887,ATPas	53.521197	-8.991851792	1.88278391	-4.775827827	1.79E-06	4.15E-05
PITA_000026428	High quality	AT5G43430	MA_10434886g0010	shade<sun	Encodes the electron transfer flavoproteir	ELECTRON TRANSFER FLAVOPROTE	chlorophyll catabolic process-GO:0(	71.406112	-0.837424357	0.26352335	-3.17779942	0.001483974	0.017849507
PITA_000026572	High quality	AT2G28000	MA_10436772g0010	shade>sun	Encodes chaperonin-60 alpha, a molecula	CHAPERONIN-60ALPHA (CPN60A)	apoplast-GO:0048046,chloroplast-(	108.02247	23.75154617	4.32841291	5.487356833	4.08E-08	2.16E-06
PITA_000026574	High quality	AT1G44575	MA_575444g0010	shade<sun	Encoding PSII-S (CP22), a ubiquitous pigm	NONPHOTOCHEMICAL QUENCHIN(	chlorophyll binding-GO:0016168,ch	1678.6866	-1.40940332	0.50280139	-2.803101504	0.005061374	0.049564688
PITA_000026702	High quality	AT5G19420	MA_10435870g0010	shade>sun	Regulator of chromosome condensation (RCC1) family with FYVE zinc finger d		chloroplast-GO:0009507,cytoplasm	46.238039	0.816499527	0.28944169	2.820946483	0.004788219	0.047525858
PITA_000026705	High quality	AT1G20510	MA_10430515g0010	shade<sun	OPC-8:0 CoA ligase1;(source:Araport11)	OPC-8:0 COA LIGASE1 (OPCL1)	4-coumarate-CoA ligase activity-GC	60.714318	-22.53151824	4.4082046	-5.11126871	3.20E-07	1.18E-05
PITA_000026719	Low quality	AT4G02800	MA_84757g0010	shade<sun	GRIP/coiled-coil protein;(source:Araport1	(MADA1)	biological_process_unknown-GO:0(	243.61963	-0.667935353	0.14951706	-4.467285243	7.92E-06	0.000158707
PITA_000026738	Low quality	AT3G56360	MA_432009g0010	shade<sun	hypothetical protein;(source:Araport11)		biological_process_unknown-GO:0(	153.81506	-3.288094301	0.91360404	-3.599036518	0.000319398	0.004679149
PITA_000026770	High quality	AT5G04150	MA_92487g0010	shade<sun	Encodes a member of the basic helix-loop	(BHLH101)	cellular response to iron ion starvat	267.06547	-0.822861993	0.20034698	-4.10718443	4.01E-05	0.000718731
PITA_000026802	High quality	AT2G31900	MA_10433033g0020	shade>sun	Encodes an novel myosin isoform.	MYOSIN-LIKE PROTEIN XIF (XIF)	actin cytoskeleton-GO:0015629,act	68.857253	0.861289102	0.28158449	3.058723535	0.002222822	0.025242581
PITA_000026831	High quality	AT4G11070	MA_6177g0010	shade<sun	member of WRKY Transcription Factor; G	(WRKY41)	DNA-binding transcription factor ac	52.550218	-22.4390271	4.4083279	-5.09014474	3.58E-07	1.19E-05
PITA_000026837	High quality	AT4G02570	MA_10316827g0010	shade>sun	Encodes a cullin that is a component of S	CULLIN 1 (CUL1)	condensed nuclear chromosome-G	270.05617	24.97459543	4.328158	5.770259648	7.91E-09	5.77E-07
PITA_000027031	High quality	AT1G71695	MA_10275776g0010	shade<sun	Peroxidase superfamily protein;(source:Araport11)		cell wall-GO:0005618,extracellular	70.717523	-22.84103454	4.40809223	-5.181614484	2.20E-07	9.17E-06
PITA_000027040	High quality	AT1G68510	MA_91369g0010	shade>sun	LOB domain protein.	LOB DOMAIN-CONTAINING PROTE	cellular_component_unknown-GO:	762.65576	13.31844913	2.64166419	5.041688936	4.61E-07	1.48E-05
PITA_000027067	High quality	AT2G28080	MA_220991g0010	shade<sun	UDP-Glycosyltransferase superfamily protein;(source:Araport11)		transferase activity, transferring gly	129.07662	-1.803722082	0.62939878	-2.865785783	0.004159756	0.042304622
PITA_000027082	High quality	AT4G26090	MA_486866g0010	shade<sun	Encodes a plasma membrane protein witl	RESISTANT TO P. SYRINGAE 2 (RPS2	ADP binding-GO:0043531,defense	68.748921	-1.771558108	0.5693636	-3.11147063	0.00186158	0.021658945
PITA_000027088	Low quality	AT5G39360	MA_20557g0010	shade<sun	EID1-like 2;(source:Araport11)	EID1-LIKE 2 (EDL2)	biological_process_unknown-GO:0(	35.358761	-21.90137695	4.40877489	-4.967678662	6.78E-07	2.02E-05

PITA_000027131	Low quality	AT4G23140	MA_9137g0010	shade<sun	Arabidopsis thaliana receptor-like protein	CYSTEINE-RICH RLK (RECEPTOR-LIK	chloroplast-GO:0009507,cytosol-G	70.717523	-22.84103454	4.40809223	-5.181614484	2.20E-07	9.17E-06
PITA_000027132	High quality	AT4G23180	MA_18132g0010	shade<sun	Encodes a receptor-like protein kinase. Na	CYSTEINE-RICH RLK (RECEPTOR-LIK	cytosol-GO:0005829,defense resp	87.516319	1.646760893	0.39809107	4.136643636	3.52E-05	0.000638249
PITA_000027319	High quality	AT5G40010	MA_23842g0010	shade<sun	Encodes a mitochondrial ATPase involved	AAA-ATPASE 1 (AATP1)	ATPase activity-GO:0016887,endop	34.530423	2.443966457	0.62893105	3.885905235	0.000101949	0.001690879
PITA_000027327	High quality	AT5G66460	MA_110162g0010	shade<sun	Encodes a endo-beta-mannanase involved	ENDO-BETA-MANNASE 7 (MAN7)	cell periphery-GO:0071944,extrace	159.99774	-1.540797076	0.19198787	-8.025491646	1.01E-15	3.20E-13
PITA_000027349	High quality	AT2G30020	MA_1274g0010	shade<sun	Encodes AP2C1. Belongs to the clade B o	(AP2C1)	abscisic acid-activated signaling pat	23.572508	-21.35301597	4.4094576	-4.84254933	1.28E-06	3.20E-05
PITA_000027373	High quality	AT3G59890	MA_122229g0010	shade<sun	Dihydrodipicolinate reductase, bacterial/plant;(source:Araport11)		4-hydroxy-tetrahydrodipicolinate re	131.28523	-0.700540444	0.24283691	-2.884818692	0.003916391	0.040177871
PITA_000027382	Low quality	AT5G03450	MA_97097g0010	shade<sun	Transducin/WD40 repeat-like superfamily protein;(source:Araport11)		interstrand cross-link repair-GO:00	808.42328	-1.900627872	0.59977919	-3.168879344	0.001530279	0.018328993
PITA_000027404	Low quality	AT3G22530	MA_10330g0020	shade<sun	heat shock protein;(source:Araport11)		biological_process_unknown-GO:00	24.424525	1.215197682	0.40975941	2.965637043	0.003020567	0.032734105
PITA_000027487	High quality	AT2G33150	MA_10427773g0010	shade<sun	Encodes an organellar (peroxisome, glyox	PEROXISOMAL 3-KETOACYL-COA T	acetyl-CoA C-acyltransferase activit	132.77382	-1.246469054	0.36792987	-3.387789923	0.000704582	0.009361378
PITA_000027558	High quality	AT2G33580	MA_892990g0010	shade<sun	Encodes a putative LysM-containing recep	LYSM-CONTAINING RECEPTOR-LIKE	cellular response to chitin-GO:0071	572.84803	-1.862701345	0.26822491	-6.944550135	3.80E-12	6.11E-10
PITA_000027578	High quality	AT2G35730	MA_10434829g0020	shade<sun	Heavy metal transport/detoxification supe	HEAVY METAL ASSOCIATED PROTE	metal ion transport-GO:0030001,m	1018.4889	-0.625365665	0.19925525	-3.138515368	0.00169806	0.020084955
PITA_000027646	High quality	AT3G02960	MA_91779g0010	shade<sun	Heavy metal transport/detoxification supe	HEAVY METAL ASSOCIATED PROTE	metal ion transport-GO:0030001,p	70.717523	-22.84103454	4.40809223	-5.181614484	2.20E-07	9.17E-06
PITA_000027688	Low quality	AT1G11290	MA_100606g0010	shade<sun	Pentatricopeptide Repeat Protein contain	CHLORORESPIRATORY REDUCTION	chloroplast-GO:0009507,endonucle	150.36173	1.542293722	0.54182279	2.846491054	0.004420397	0.044601076
PITA_000027780	High quality	AT2G38940	MA_366260g0010	shade<sun	Encodes Pht1;4, a member of the Pht1 fa	PHOSPHATE TRANSPORTER 1;4 (PH	cytoplasm-GO:0005737,Golgi appa	167.47009	-10.6372845	1.74409691	-6.099021454	1.07E-09	9.80E-08
PITA_000027912	High quality	AT3G12120	MA_203704g0010	shade<sun	Major enzyme responsible for the synthes	FATTY ACID DESATURASE 2 (FAD2)	1-16:0-2-18:1-phosphatidylcholine	578.80045	-1.553772025	0.20270408	-7.665223386	1.79E-14	4.86E-12
PITA_000027981	High quality	AT3G26380	MA_10434513g0010	shade<sun	APSE is a member of the Glycoside Hydro	ARAPASE (APSE)	arabinogalactan protein metabolic	2290.8576	-0.842952868	0.17259574	-4.883972526	1.04E-06	2.80E-05
PITA_000027982	High quality	AT5G08370	MA_54403g0020	shade<sun	Member of Glycoside Hydrolase Family 27	ALPHA-GALACTOSIDASE 2 (AGAL2)	alpha-galactosidase activity-GO:00	17.516739	-20.95349401	4.41016289	-4.751183692	2.02E-06	4.57E-05
PITA_000028119	High quality	AT3G01930	MA_40195g0010	shade<sun	Major facilitator superfamily protein;(source:Araport11)		membrane-GO:0016020,mitochon	11.506973	2.441172721	0.74876985	3.260244397	0.001113162	0.013878221
PITA_000028190	High quality	AT3G05590	MA_10433593g0010	shade<sun	Encodes phosphoribosylanthranilate isom	PHOSPHORIBOSYLANTHRANILATE	chloroplast-GO:0009507,phosphor	148.75126	-0.842192838	0.22826406	-3.68955515	0.000224647	0.003450959
PITA_000028233	High quality	AT4G12910	MA_8787g0010	shade<sun	serine carboxypeptidase-like 20;(source:A	SERINE CARBOXYPEPTIDASE-LIKE 2	extracellular region-GO:0005576,p	7847.3574	-2.617419581	0.21918966	-11.94134613	7.20E-33	1.68E-29
PITA_000028403	High quality	AT3G13540	MA_19052g0010	shade<sun	Encodes a member of the MYB family of t	MYB DOMAIN PROTEIN 5 (MYB5)	cell differentiation-GO:0030154,DN	41.781918	0.859828004	0.30581916	2.81162963	0.004929122	0.048723547
PITA_000028484	Low quality	AT4G27450	MA_109804g0010	shade<sun	aluminum induced protein with YGL and L	HYPOXIA RESPONSE UNKNOWN P	cellular response to hypoxia-GO:00	7.0175117	3.154744801	1.05707682	2.984404476	0.002841309	0.031054307
PITA_000028719	High quality	AT3G47520	MA_10431009g0010	shade<sun	Encodes a protein with NAD-dependent m	MALATE DEHYDROGENASE (MDH)	apoplast-GO:0048046,chloroplast-G	181.52915	24.4760016	4.32824088	5.654953659	1.56E-08	1.02E-06
PITA_000028738	Low quality	AT5G44210	MA_10426586g0010	shade<sun	encodes a member of the ERF (ethylene r	ERF DOMAIN PROTEIN 9 (ERF9)	DNA binding-GO:0003677,DNA-bin	34.077865	0.980248135	0.33377723	2.936833485	0.003315821	0.035189201
PITA_000028834	High quality	AT2G40890	MA_109548g0010	shade<sun	encodes coumarate 3-hydroxylase (C3H),	CYTOCHROME P450, FAMILY 98, S	coumarin biosynthetic process-GO:	157.87831	-1.269360834	0.23986842	-5.291904699	1.21E-07	5.73E-06
PITA_000028919	Low quality	AT2G32480	MA_117901g0010	shade<sun	Metalloprotease essential for plastid deve	ARABIDOPSIS SERIN PROTEASE (AR	chloroplast-GO:0009507,chloropla	156.96265	-0.679264952	0.22505768	-3.018181639	0.002542964	0.028350122
PITA_000028995	High quality	AT5G64610	MA_10429572g0010	shade<sun	Encodes an enzyme with histone acetyltra	HISTONE ACETYLTRANSFERASE OF	DNA repair-GO:0006281,histone ac	36.564163	0.91895249	0.32364466	2.839387139	0.004520028	0.045049872
PITA_000029224	Low quality	AT3G25130	MA_14986g0010	shade<sun	acidic leucine-rich nuclear phosphoprotein	32 family B protein;(source:Arapor	biological_process_unknown-GO:00	380.41929	0.767938006	0.24693656	3.109859504	0.001871764	0.021767539
PITA_000029439	High quality	AT3G14690	MA_10337928g0010	shade<sun	putative cytochrome P450 The mRNA is c	CYTOCHROME P450, FAMILY 72, S	chloroplast-GO:0009507,cytosol-G	20.117352	-7.583889665	1.72750332	-4.390086892	1.13E-05	0.00022166
PITA_000029507	High quality	AT5G57540	MA_8261658g0010	shade<sun	Encodes a xyloglucan endotransglucosyla	XYLOGLUCAN ENDOTRANSGLUCOS	cell wall biogenesis-GO:0042546,e	119.89432	1.974979733	0.2268848	8.704768909	3.18E-18	1.70E-15
PITA_000029611	High quality	AT1G10370	MA_14940g0020	shade<sun	Encodes GSTU17 (Glutathione S-Transfer	EARLY-RESPONSIVE TO DEHYDRATI	chloroplast-GO:0009507,cytoplasm	214.21607	-1.91553804	0.3356589	-5.706799575	1.15E-08	7.88E-07
PITA_000029627	High quality	AT5G61400	MA_69436g0010	shade<sun	Pentatricopeptide repeat (PPR) superfamily protein;(source:Araport11)		biological_process_unknown-GO:00	209.10122	-0.874994115	0.29184408	-2.998156115	0.002716185	0.02992946
PITA_000029637	High quality	AT3G44110	MA_10430505g0010	shade<sun	homologous to the co-chaperon DNAJ pro	(J3)	ATP binding-GO:0005524,cell wall-	22.90419	8.261047707	1.58356958	5.216725441	1.82E-07	8.30E-06
PITA_000029644	High quality	AT5G51600	MA_10426277g0020	shade<sun	Mutant has defective roots. Essential for p	PLEIADE (PLE)	cortical microtubule-GO:0055028,c	252.82033	-0.714507818	0.25434016	-2.809467408	0.004962354	0.048894178
PITA_000029719	High quality	AT4G19860	MA_102148g0010	shade<sun	Encodes a cytosolic calcium-independent	phospholipase A.	cytoplasm-GO:0005737,cytosol-GC	267.36992	-1.393908214	0.35181749	-3.962020832	7.43E-05	0.001269604
PITA_000029791	High quality	AT5G23420	MA_86565g0010	shade<sun	Encodes HMGB6, a protein belonging to t	HIGH-MOBILITY GROUP BOX 6 (HM	DNA binding-GO:0003677,DNA-bin	87.211241	1.801741431	0.37966103	4.745658127	2.08E-06	4.59E-05
PITA_000029837	High quality	AT4G36850	MA_87001g0010	shade<sun	PQ-loop repeat family protein / transmem	brane family protein;(source:Arapo	biological_process_unknown-GO:00	58.892591	1.584974728	0.30627314	5.175036592	2.28E-07	9.49E-06
PITA_000029882	Low quality	AT3G11180	MA_10430508g0010	shade<sun	One of 4 paralogs encoding a 2-oxoglutar	JASMONATE-INDUCED OXYGENASE	cytoplasm-GO:0005737,dioxygena	445.08684	12.54157804	1.81002017	6.928971428	4.24E-12	6.66E-10
PITA_000029961	High quality	AT1G78020	MA_14341g0010	shade<sun	FCS like zinc finger 6 is induced during en	FCS LIKE ZINC FINGER 6 (FLZ6)	cell growth-GO:0016049,cytoplasm	738.23514	0.625045303	0.20916228	2.988327049	0.002805092	0.030757251
PITA_000029965	High quality	AT5G19200	MA_681548g0010	shade<sun	Encodes one of the Arabidopsis proteins (	TSC10B (TSC10B)	3-dehydrosphinganine reductase ac	54.317441	1.810139311	0.62479097	2.897191858	0.003765194	0.038925754
PITA_000030002	High quality	AT5G66870	MA_290245g0010	shade<sun	Encodes LOB domain protein whose overe	ASYMMETRIC LEAVES 2-LIKE 1 (ASL	leaf morphogenesis-GO:0009965,n	24.591652	1.519618443	0.51258193	2.964635193	0.003030421	0.032799243
PITA_000030083	High quality	AT5G09220	MA_6860g0010	shade<sun	member of AAAP family The mRNA is cell	AMINO ACID PERMEASE 2 (AAP2)	acidic amino acid transport-GO:001	10.306886	7.109058514	2.16098088	3.289736844	0.001002811	0.012668962
PITA_000030199	Low quality	AT5G08630	MA_91633g0010	shade<sun	DDT domain-containing protein;(source:A	DDT-WAC PROTEIN1 (DDW1)	biological_process_unknown-GO:00	479.47093	-2.267470029	0.65975941	-3.436813472	0.000588601	0.008013232
PITA_000030269	High quality	AT1G47530	MA_31315g0010	shade<sun	MATE efflux family protein;(source:Arapo	(DTX33)	antiporter activity-GO:0015297,cyt	87.717671	10.19874607	2.44527534	4.170686188	3.04E-05	0.000557073
PITA_000030329	Low quality	AT5G66460	MA_111906g0010	shade<sun	Encodes a endo-beta-mannanase involved	ENDO-BETA-MANNASE 7 (MAN7)	cell periphery-GO:0071944,extrace	84.266093	10.14065349	1.60555099	6.315995907	2.68E-10	2.81E-08
PITA_000030442	High quality	AT5G52440	MA_919815g0010	shade<sun	HCF106; nuclear gene for chloroplast. Thy	HIGH CHLOROPHYLL FLUORESCENC	chloroplast-GO:0009507,chloropla	203.99855	-0.739425929	0.16958894	-4.36010715	1.30E-05	0.000251816
PITA_000030522	High quality	AT4G34730	MA_7478542g0010	shade<sun	ribosome-binding factor A family protein;	RBFA DOMAIN-CONTAINING PROT	chloroplast-GO:0009507,ribosoma	363.52789	-1.023740643	0.20880091	-4.902950993	9.44E-07	2.58E-05
PITA_000030650	High quality	AT1G12220	MA_37473g0010	shade<sun	Resistance gene, mediates resistance aga	RESISTANT TO P. SYRINGAE 5 (RPS	ADP binding-GO:0043531,cell deat	60.714318	-22.53151824	4.4082046	-5.11126871	3.20E-07	1.18E-05
PITA_000030667	High quality	AT1G01090	MA_10426096g0010	shade<sun	pyruvate dehydrogenase E1 alpha subuni	PYRUVATE DEHYDROGENASE E1 AL	acetyl-CoA biosynthetic process fro	119.3011	-0.644824408	0.20710212	-3.113557756	0.001848463	0.02153569
PITA_000030674	High quality	AT1G76110	MA_48961g0010	shade<sun	HMG (high mobility group) box protein wit	ARID/BRIGHT DNA-binding doma	DNA binding-GO:0003677,DNA-bin	25.301858	1.36945707	0.47750542	2.867940376	0.004131534	0.042051001
PITA_000030698	High quality	AT4G16820	MA_179419g0010	shade<sun	Encodes a lipase that hydrolyzes phospha	PHOSPHOLIPASE A I BETA 2 (PLA-I	chloroplast-GO:0009507,extracellu	35.358761	-21.90137695	4.40877489	-4.967678662	6.78E-07	2.02E-05
PITA_000030909	High quality	AT2G23810	MA_24323g0010	shade<sun	Member of TETRASPANIN family	TETRASPANIN8 (TET8)	defense response to Gram-negative	992.99642	-1.251177159	0.19824344	-6.311316867	2.77E-10	2.86E-08
PITA_000031023	Low quality	AT5G14890	MA_10431198g0020	shade<sun	potassium transporter;(source:Araport11)		extracellular region-GO:0005576	66.262348	9.793905293	1.35414595	7.232533023	4.74E-13	9.27E-11
PITA_000031137	Low quality	AT3G61870	MA_80427g0010	shade<sun	plant/protein;(source:Araport11)		biological_process_unknown-GO:00	1090.9589	-1.092252126	0.14692358	-7.434151468	1.05E-13	2.47E-11
PITA_000031148	High quality	AT1G14590	MA_386090g0010	shade<sun	Nucleotide-diphospho-sugar transferase family protein;(source:Araport11)		biological_process_unknown-GO:00	92.454477	-1.277597185	0.4313607	-2.961783914	0.003058624	0.033048621
PITA_000031224	Low quality	AT1G21910	MA_10020434g0010	shade<sun	encodes a member of the DREB subfamily	DEHYDRATION RESPONSE ELEMEN	cellular response to freezing-GO:00	19.455072	8.025343237	2.13640085	3.756478216	0.000172321	0.002730695
PITA_000031236	High quality	AT4G39330	MA_10427985g0010	shade<sun	cinnamyl alcohol dehydrogenase 9;(sourc	CINNAMYL ALCOHOL DEHYDROGE	apoplast-GO:0048046,cinnamyl-alc	54.72956	-1.126448089	0.40068659	-2.811294719	0.004934257	0.048723547
PITA_000031332	Low quality	AT1G32520	MA_93555g0010	shade<sun	TLDc domain protein;(source:Araport11)	OXIDATION RESISTANCE 4 (OXR4)	biological_process_unknown-GO:00	400.58691	-0.820852433	0.18473153	-4.443488635	8.85E-06	0.000176253
PITA_000031391	High quality	AT3G54740	MA_34382g0010	shade<sun	zein-binding protein (Protein of unknown	function, DUF593);(source:Araport1	biological_process_unknown-GO:00	109.33358	0.758055362	0.22397998	3.38447816	0.000713137	0.009448253
PITA_000031446	Low quality	AT5G63020	MA_166614g0010	shade<sun	Nucleotide binding leucine rich repeat pro	SUPPRESSORS OF TOPP4-1 (SUT1)	ADP binding-GO:0043531,cytoplas	58.343556	9.609934207	1.82769934	5.257940409	1.46E-07	6.75E-06
PITA_000031479	High quality	AT4G28740	MA_173127g0010	shade<sun	LOW PSII ACCUMULATION-like protein;(source:Araport11)		chloroplast-GO:0009507,chloropla	526.73873	-0.734087893	0.22677838	-3.237027712	0.001207817	0.014913102
PITA_000031627	High quality	AT2G39980	MA_34530g0020	shade<sun	HXXXD-type acyl-transferase family protein;(source:Araport11)		cytoplasm-GO:0005737,response t	106.86975	-3.279915136	0.35193983	-9.319533786	1.17E-20	1.07E-17
PITA_000031654	High quality	AT1G07990	MA_10435320g0020	shade<sun	SIT4 phosphatase-associated family protein;(source:Araport11)		chloroplast-GO:0009507,chloropla	32.381708	4.054292268	1.23259435	3.289234824	0.001004602	0.012685326
PITA_000031694	High quality	AT1G64510	MA_10429326g0010	shade<sun	Translation elongation factor EF1B/ribosc	PLASTID RIBOSOMAL PROTEIN OF	chloroplast-GO:0009507,chloropla	323.62343	-0.7926562	0.16723682	-4.739722923	2.14E-06	4.70E-05
PITA_000031809	High quality	AT4G28100	MA_141029g0010	shade<sun	transmembrane protein;(source:Araport11)		anchored component of membrane	346.71429	-0.617249251	0.13079073	-4.719365565	2.37E-06	5.16E-05
PITA_000031810	High quality	AT5G03300	MA_255g0010	shade<sun	Encodes adenosine kinase 2 (ADK2), a typ	ADENOSINE KINASE 2 (ADK2)	adenosine kinase activity-GO:0004	178.82101	-0.760473084	0.21552399	-3.528484542	0.000417946	0.00596587
PITA_000031839	High quality	AT2G26490	MA_19051g0010	shade<sun	JGB contains seven WD40 repeats and is	JINGUBANG (JGB)	biological_process_unknown-GO:00	29.719068	2.447577545	0.74315068	3.293514497	0.000989432	0.01254326
PITA_000031933	High quality	AT5G26340	MA_130810g0010	shade<sun	Encodes a protein with high affinity, hexo	(MSS1)	carbohydrate transmembrane tran	245.38681	-1.496686706	0.32052711	-4.669454386	3.02E-06	6.46E-05
PITA_000031948	High quality	AT5G54160	MA_10426788g0010	shade<sun	A caffeic acid/5-hydroxyferulic acid O-me	O-METHYLTRANSFERASE 1 (OMT1)	acetylserotonin O-methyltransferas	17.516739	-20.95349401	4.41016289	-4.751183692	2.02E-06	4.57E-05
PITA_000031953	High quality	AT2G01170	MA_10435539g0010	shade<sun	Encodes a bidirectional amino acid transp	BIDIRECTIONAL AMINO ACID TRAN	arginine transmembrane transporte	159.23417	-1.06637332	0.30560399	-3.48939591	0.000484114	0.00675964
PITA_000032002	High quality	AT2G38090	MA_908g0010	shade<sun	Duplicated homeodomain-like superfamily protein;(source:Araport11)		DNA-binding transcription factor ac	17.516739	-20.95349401	4.41016289	-4.751183692	2.02E-06	4.57E-05
PITA_000032024	High quality	AT1G11915	MA_104588g0010	shade<sun	wall-associated receptor kinase galacturonan-binding protein;(source:Araport		extracellular region-GO:0005576,p	100.37718	-0.769088676	0.23074991	-3.332996649	0.00085916	0.0111118
PITA_000032112	High quality	AT4G17090	MA_3193g0010	shade<sun	Encodes a beta-amylase targeted to the c	CHLOROPLAST BETA-AMYLASE (CT-	amylopectin maltohydrolase activit	993.72327	-1.116276779	0.35817615	-3.116558067	0.001829756	0.02135661
PITA_000032138	High quality	AT1G07670	MA_10427630g0010	shade<sun	TPLATE complex protein involved in clathr	ENDOMEMBRANE-TYPE CA-ATPASE	ATPase activity-GO:0016887,calciu	235.54038	11.62338235	1.9951628	5.825781414	5.68E-09	4.35E-07
PITA_000032194	High quality	AT4G30940	MA_10430453g0020	shade<sun	BTB/POZ domain with WD40/YVTN repeat-like protein;(source:Araport11)		nucleus-GO:0005634,protein homo	35.358761	-21.90137695	4.40877489	-4.967678662	6.78E-07	2.02E-05
PITA_000032300	High quality	AT5G56600	MA_10426093g0020	shade<sun	Encodes profilin3, a low-molecular weigh	PROFILIN 3 (PRF3)	actin filament polymerization-GO:0	69.138664	9.855158599	2.14196698	4.600985313	4.20E-06	8.78E-05
PITA_000032321	High quality	AT2G07690	MA_10426777g0010	shade<sun	Member of the minichromosome mainter	MINICHROMOSOME MAINTENANC	3'-5' DNA helicase activity-GO:004	52.550218	-22.4390271	4.4083279	-5.09014474	3.58E-07	1.19E-05
PITA_000032339	High quality	AT2G36460	MA_386405g0010	shade<sun	Aldolase superfamily protein;(source:Ara	FRUCTOSE-BISPHOSPHATE ALDOLA	cellular response to hypoxia-GO:00	324.90124	-0.87665247	0.17138957	-5.114969721	3.14E-07	1.18E-05
PITA_000032419	High quality	AT3G61590	MA_177764g0010	shade<sun	F-box protein that is involved in some asp	HAWAIIAN SKIRT (HWS)	extracellular region-GO:0005576,g	68.989204	-1.341205891	0.32319159	-4.149878686	3.33E-05	0.000606302
PITA_000032491	High quality	AT5G50375	MA_9262292g0010	shade<sun	Converts pentacyclic cyclopropyl sterols t	CYCLOPROPYL ISOMERASE (CPI1)	cycloeucalenol cycloisomerase acti	364.22063	-1.153838558	0.3669162	-3.14469229	0.001662616	0.019766143
PITA_000032496	Low quality	AT2G35630	MA_276224g0010	shade<sun	Member of the MAP215 family of microtu	MICROTUBULE ORGANIZATION 1 (	cell plate formation involved in plar	181.52915	24.4760016	4.32824088	5.654953659	1.56E-08	1.02E-06
PITA_000032587	High quality	AT5G53250	MA_10113222g0010	shade<sun	arabinogalactan protein 22;(source:Arapc	ARABINOGALACTAN PROTEIN 22 (	anchored component of membrane	72.718145	-1.833628904	0.38415816	-4.773109304	1.81E-06	4.20E-05
PITA_000032785	High quality	AT1G27170	MA_89418g0010	shade<sun	transmembrane receptors / ATP binding protein;(source:Araport11)		ADP binding-GO:0043531,cytoplas	70.717523	-22.84103454	4.40809223	-5.181614484	2.20E-07	9.17E-06
PITA_000032826	High quality	AT5G50890	MA_10212g0010	shade<sun	alpha/beta-Hydrolases superfamily protein;(source:Araport11)		nucleus-GO:0005634	60.714318	-22.53151824	4.4082046	-5.11126871	3.20E-07	1.18E-05
PITA_000032918	High quality	AT4G01870	MA_10434906g0020	shade<sun	tolB protein-like protein;(source:Araport11)		chloroplast-GO:0009507,cytosol-G	15.350709	1.452234448	0.49697578	2.922143291	0.003476316	0.036589187
PITA_000033137	High quality	AT5G46330	MA_85663g0010	shade<sun	Encodes a leucine-rich receptor serine/thre	FLAGELLIN-SENSITIVE 2 (FLS2)	defense response by callose deposi	162.0337	24.31540594	4.32827129	5.617810044	1.93E-08	1.19E-06
PITA_000033196	High quality	AT5G19160	MA_10143614g0010	shade<sun	Encodes a member of the TBL (TRICHOMI	TRICHOME BIREFRINGENCE-LIKE 1	Golgi apparatus-GO:0005794,nucle	60.714318	-22.53151824	4.4082046	-5.11126871	3.20E-07	1.18E-05
PITA_000033273	High quality	AT5G52450	MA_10434642g0010	shade<sun	MATE efflux family protein;(source:Araport11)		antiporter activity-GO:0015297,me	21.463913	1.708429081	0.47849787	3.570400588	0.000356436	0.005147438
PITA_000033275	High quality	AT5G11460	MA_481693g0010	shade<sun	FCS like zinc finger 10 is induced during e	FCS LIKE ZINC FINGER 10 (FLZ10)	cell growth-GO:0016049,cytoplasm	387.68959	-0.496488337	0.12085136	-4.108256047	3.99E-05	0.000715907
PITA_000033380	High quality	AT1G05670	MA_7256709g0010	shade<sun	Pentatricopeptide repeat (PPR-like) superfamily protein;(source:Araport11)		endoplasmic reticulum-GO:000578	30.357159	-21.69383454	4.40899933	-4.920353335	8.64E-07	2.39E-05
PITA_000033409	High quality	AT4G29670	MA_10436837g0010	shade<sun	Encodes a member of the thioredoxin fam	ATYPICAL CYS HIS RICH THIOREDO	chloroplast-GO:0009507,oxidoredu	142.79661	-0.64847593	0.20639888	-3.141857748	0.001678796	0.019903057
PITA_000033440	High quality	AT2G04850	MA_10431938g0010	shade<sun	Auxin-responsive family protein;(source:Araport11)		extracellular region-GO:0005576	910.26586	-1.794006765	0.62195533	-2.884462417	0.003920826	0.040177871
PITA_000033475	High quality	AT5G65360	MA_10432805g0020	shade<sun	Histone superfamily protein;(source:Arap	HISTONE 3.1 (H3.1)	chromocenter-GO:0010369,nucleu	17.516739	-20.95349401	4.41016289	-4.751183692	2.02E-06	4.57E-05
PITA_000033492	Low quality	AT4G25030	MA_83834g0010	shade<sun	Plastid localized protein of unknown func	NON HOST RESISTANCE 2B (ATNHR	chloroplast-GO:0009507,defense re	134.39212	-1.025931073	0.21645655	-4.739662802	2.14E-06	4.70E-05
PITA_000033503	High quality	AT1G11545	MA_10436348g0010	shade<sun	xyloglucan endotransglucosylase/hydrola	XYLOGLUCAN ENDOTRANSGLUCOS	cell wall biogenesis-GO:0042546,e	22.061349	1.664066245	0.44815078	3.71318388	0.000204668	0.003180303
PITA_000033647	High quality	AT3G14200	MA_204527g0010	shade<sun	Chaperone DnaJ-domain superfamily protein;(source:Araport11)		biological_process_unknown-GO:00	1655.504	-1.318965807	0.34422344	-3.831714075	0.000127254	0.00207429
PITA_000033769	High quality	AT3G52540	MA_10329547g0010	shade<sun	Protein kinase superfamily protein;(source:Araport11)		ATP binding-GO:0005524,nucleus-G	17.404771	-20.94419205	4.41018187	-4.749054039	2.04E-06	4.59E-05
PITA_000033770	High quality	AT3G47570	MA_10350490g0010	shade<sun	Leucine-rich repeat protein kinase family protein;(source:Araport11)		kinase activity-GO:0016301,plasma	30.357159	-21.69383454	4.40899933	-4.920353335	8.64E-07	2.39E-05
PITA_000033851	High quality	AT1G57560	MA_197412g0010	shade<sun	Member of the R2R3 factor gene family.	MYB DOMAIN PROTEIN 50 (MYB50	DNA-binding transcription factor ac	17.679381	-20.92872422	4.41014042	-4.745591347	2.08E-06	4.59E-05
PITA_000033880	High quality	AT5G64410	MA_10185385g0010	shade<sun	oligopeptide transporter	OLIGOPEPTIDE TRANSPORTER 4 (O	integral component of plasma mem	15.732031	2.247752199	0.59980081	3.747497796	0.000178607	0.002818101
PITA_000033895	Low quality	AT1G56430	MA_10047260g0010	shade<sun	Encodes a protein with nicotianamine syn	NICOTIANAMINE SYNTHASE 4 (NA	nicotianamine biosynthetic process	242.99634	-1.154072368	0.34244832	-3.370062865	0.00075151	0.009874129
PITA_000033943	Low quality	AT3G05380	MA_519985g0010	shade<sun	Tetratricopeptide repeat (TPR)-like superf	(MEF26)	intracellular membrane-bounded o	225.07166	-1.654223796	0.41859634	-3.951835352	7.76E-05	0.001317854
PITA_000034022	High quality	AT5G52060	MA_10425833g0010	shade<sun	A member of Arabidopsis BAG (Bcl-2-assoc	BCL-2-ASSOCIATED ATHANOGENE	adenyl-nucleotide exchange factor	167.20986	-0.685880648	0.17342545	-3.954901942	7.66E-05	0.001306265
PITA_000034167	High quality	AT5G06490	MA_10323287g0010	shade<sun	RING/U-box superfamily protein;(source:A	ARABIDOPSIS T??XICOS EN LEVADU	nucleus-GO:0005634,protein ubiqu	52.550218	-22.4390271	4.4083279	-5.09014474	3.58E-07	1.19E-05
PITA_000034214	High quality	AT1G60360	MA_10428893g0010	shade<sun	RING/U-box superfamily protein;(source:A	BCA2A ZINC FINGER ATL 17 (BTL17	ubiquitin protein ligase activity-GO	47.145015	-22.29213208	4.40843355	-5.056701391	4.27E-07	1.38E-05
PITA_000034238	High quality	AT4G36430	MA_10227622g0010	shade<sun	Peroxidase superfamily protein;(source:Araport11)		cell wall-GO:0005618,extracellular	26.275109	-21.49994244	4.40924566	-4.876104457	1.08E-06	2.80E-05
PITA_000034245	High quality	AT4G04930	MA_469702g0010	shade<sun	Encodes a sphingolipid delta4-desaturase	(DES-1-LIKE)	ceramide biosynthetic process-GO:	138.20668	-0.868159395	0.26676197	-3.254434669	0.001136183	0.014096595
PITA_000034250	High quality	AT2G04570	MA_166543g0010	shade<sun	GDSL-motif esterase/acyltransferase/lipa	OCCLUDED STOMATAL PORES 1 (O	acyl-[acyl-carrier-protein] hydrolase	377.96732	-11.81128883	1.72704599	-6.839012347	7.97E-12	1.19E-09
PITA_000034360	High quality	AT1G74930	MA_10434101g0020	shade<sun	encodes a member of the DREB subfamily	(ORA47)	cell division-GO:0051301,defense r	90.018724	23.49507644	4.32849788	5.427997682	5.70E-08	2.93E-06
PITA_000034362	High quality	AT2G21860	MA_89112g0010	shade<sun	violaxanthin de-epoxidase-like protein;(source:Araport11)		chloroplast-GO:0009507,magnesiu	519.64297	-1.050187588	0.14825465	-7.08367388	1.40E-12	2.48E-10
PITA_000034403	Low quality	AT5G62140	MA_422522g0010	shade<sun	ATP-dependent Clp protease ATP-binding subunit;(source:Araport11)		chloroplast-GO:0009507,plastid-G	432.20404	-0.737755694	0.15661953	-4.710496067	2.47E-06	5.36E-05
PITA_000034452	High quality	AT5G15280	MA_100612g0010	shade<sun	Pentatricopeptide repeat (PPR) superfamily protein;(source:Araport11)		biological_process_unknown-GO:00	70.717523	-22.84103454	4.40809223	-5.181614484	2.20E-07	9.17E-06
PITA_000034587	High quality	AT1G68710	MA_66524g0010	shade<sun	ATPase E1-E2 type family protein / haloac	AMINOPHOSPHOLIPID ATPASE 9 (	ATPase-coupled intramembrane lip	67.84583	0.77663208	0.27111727	2.864561424	0.004175872	0.042434809
PITA_000034686	High quality	AT4G17500	MA_6447416g0010	shade<sun	Encodes a member of the ERF (ethylene r	ETHYLENE RESPONSIVE ELEMENT E	cell division-GO:0051301,DNA bind	30.357159	-21.69383454	4.40899933	-4.920353335	8.64E-07	2.39E-05
PITA_000034759	High quality	AT5G62040	MA_112333g0010	shade<sun	BFT is a member of The FLOWERING LOC	BROTHER OF FT AND TFL1 (BFT)	cytoplasm-GO:0005737,flower dev	108.02247	23.75154617	4.32841291	5.487356833	4.08E-08	2.16E-06
PITA_000034937	Low quality	AT4G25170	MA_18528g0010	shade<sun	Uncharacterized conserved protein (UCP012943);(source:Araport11)		biological_process_unknown-GO:00	49.528587	2.117516289	0.68652259	3.084408774	0.002039571	0.023421232
PITA_000034993	Low quality	AT3G12012	MA_9999544g0020	shade<sun	peptide upstream ORF			169.62192	-0.787063533	0.19962878	-3.942635557	8.06E-05	0.001364029
PITA_000035062	High quality	AT4G00620	MA_112201g0010	shade<sun	Amino acid dehydrogenase family protein	EMBRYO DEFECTIVE 3127 (EMB31	chloroplast-GO:0009507,cytosol-G	160.63357	-1.76203532	0.46673821	-3.775211244	0.000159872	0.00254286
PITA_000035125	High quality	AT2G15490	MA_10661g0020	shade<sun	UDP-glycosyltransferase 73B4;(source:Ar	UDP-GLYCOSYLTRANSFERASE 73B4	chloroplast-GO:0009507,cytosol-G	85.548245	-2.305165034	0.69305156	-3.326108985	0.000880675	0.011321447
PITA_000035159	High quality	AT1G60710	MA_10432302g0010	shade<sun	Encodes ATB2.	(ATB2)	cytoplasm-GO:0005737,cytosol-G	60.714318	-22.53151824	4.4082046	-5.11126871	3.20E-07	1.18E-05
PITA_000035179	High quality	AT2G17880	MA_56308g0010	shade<sun	Chaperone DnaJ-domain superfamily prot	DNA J PROTEIN C24 (DJC24)	chloroplast-GO:0009507	28.664526	2.050226989	0.43754398	4.685762043	2.79E-06	6.01E-05
PITA_000035185	High quality	AT1G11910	MA_101119g0010	shade<sun	Encodes an aspartic proteinase that form	ASPARTIC PROTEINASE A1 (APA1)	cytosol-GO:0005829,endopeptidas	181.52915	24.4760016	4.32824088	5.654953659	1.56E-08	1.02E-06
PITA_000035220	High quality	AT1G24620	MA_8414519g0010	shade<sun	Encodes a EF-hand calcium-binding prote	CALMODULIN LIKE 25 (CML25)	calcium ion binding-GO:0005509,c	12.301644	2.463761505	0.72552514	3.395832021	0.000684203	0.009145548
PITA_000035221	High quality	AT4G08250	MA_73173g0010	shade<sun	GRAS family transcription factor;(source:Araport11)		DNA-binding transcription factor ac	197.49226	-2.097132124	0.63208836	-3.317783189	0.000907349	0.011640977
PITA_000035441	High quality	AT2G01660	MA_10351081g0010	shade<sun	Encodes a plasmodesmal protein that ma	PLASMODESMATA-LOCATED PROT	extracellular region-GO:0005576,m	5.5283368	2.764927285	0.95597604	2.892255847	0.003824521	0.039399477
PITA_000035567	High quality	AT5G02120	MA_10117352g0010	shade<sun	Encodes a one helix protein homologous	ONE-HELIX LHC-LIKE PROTEIN 1 (O	chloroplast-GO:0009507,chloropla	218.92866	-0.597565651	0.21253823	-2.811567879	0.004930069	0.048723547
PITA_000035728	High quality	AT4G37690	MA_101873g0010	shade<sun	Unlike its close paralog MUCI10 (At2g22S	GLYCOSYLTRANSFERASE 6 (GT6)	endosome-GO:0005768,galactosylt	70.717523	-22.84103454	4.40809223	-5.181614484	2.20E-07	9.17E-06
PITA_000035829	High quality	AT5G48220	MA_10433830g0010	shade<sun	Aldolase-type TIM barrel family protein;(source:Araport11)		chloroplast-GO:0009507,chloropla	615.42059	-0.99985771	0.30465808	-3.281901182	0.001031097	0.012956006
PITA_000035844	Low quality	AT2G18860	MA_10433463g0010	shade<sun	Syntaxin/t-SNARE family protein;(source:Araport11)		Golgi vesicle transport-GO:004819	121.01943	23.90838197	4.3283673	5.52364906	3.32E-08	1.87E-06
PITA_000035848	Low quality	AT4G27030	MA_368556g0010	shade<sun	Encodes an unusual palmitate desaturase	FATTY ACID DESATURASE A (FADA)	chloroplast-GO:0009507,chloropla	31087.968	-1.673879962	0.39962258	-4.188652122	2.81E-05	0.000518879
PITA_000035864	High quality	AT5G65730	MA_476450g0010	shade<sun	xyloglucan endotransglucosylase/hydrola	XYLOGLUCAN ENDOTRANSGLUCOS	cell wall biogenesis-GO:0042546,e	10.236217	3.040400336	0.94635066	3.212762935	0.001314648	0.01604647
PITA_000036005	High quality	AT4G21970	MA_737152g0010	shade<sun	senescence regulator (Protein of unknown	function, DUF584);(source:Araport	biological_process_unknown-GO:00	17.464529	2.056014893	0.53839696	3.818771398	0.000134118	0.002170982
PITA_000036055	High quality	AT1G80160	MA_34359g0010	shade<sun	Vicinal oxygen chelate (VOC) superfamily	GLYOXYLASE I 7 (GLYI7)	cytoplasm-GO:0005737	46.376899	9.279009475	1.35509479	6.847498466	7.52E-12	1.13E-09
PITA_000036084	High quality	AT5G55580	MA_161180g0010	shade<sun	Encodes a mitochondrial transcription ter	(MTERF9)	chloroplast-GO:0009507,chloropla	793.42043	-1.240909429	0.26734568	-4.641591499	3.46E-06	7.34E-05
PITA_000036151	High quality	AT1G02850	MA_139193g0010	shade<sun	beta glucosidase 11;(source:Araport11)	BETA GLUCOSIDASE 11 (BGLU11)	beta-glucosidase activity-GO:00842	199.8674	-0.981437537	0.21558348	-4.552471026	5.30E-06	0.000109054
PITA_000036228	High quality	AT2G01860	MA_10281691g0010	shade<sun	Tetratricopeptide repeat (TPR)-like superf	EMBRYO DEFECTIVE 975 (EMB975	chloroplast-GO:0009507,chloropla	219.44942	-0.523882668	0.18486048	-2.83393546	0.004597862	0.046028945
PITA_000036269	Low quality	AT2G09990	MA_32670g0010	shade<sun	Ribosomal protein S5 domain 2-like superfamily protein;(source:Araport11)		cell wall-GO:0005618,chloroplast-G	47.145015	-22.29213208	4.40843355	-5.056701391	4.27E-07	1.38E-05
PITA_000036284	High quality	AT1G15410	MA_9130g0010	shade<sun	aspartate-glutamate racemase family protein;(source:Araport11)		amino-acid racemase activity-GO:0	1193.7456	-1.823316372	0.27832742	-6.550976414	5.72E-11	7.04E-09
PITA_000036322	High quality	AT3G20390	MA_429896g0010	shade<sun	Encodes a plastidial RidA (Reactive Interm	REACTIVE INTERMEDIATE DEAMIN	2-iminobutanoate/2-iminopropano	26.275109	-21.49994244	4.40924566	-4.876104457	1.08E-06	2.80E-05
PITA_000036403	High quality	AT1G53280	MA_10431694g0020	shade<sun	Encodes a homolog of animal DJ-1 super	DJ-1 HOMOLOG B (DJ1B)	chloroplast-GO:0009507,chloropla	52.108013	-1.156510145	0.29685822	-3.895833543	9.79E-05	0.001630476
PITA_000036407	High quality	AT3G01120	MA_2215g0010	shade<sun	encodes a cystathionine gamma-synthase	METHIONINE OVERACCUMULATIO	chloroplast-GO:0009507,chloropla	190.7851	-0.686352798	0.2268736	-3.025265112	0.002484153	0.02780649
PITA_000036627	High quality	AT3G54390	MA_113047g0010	shade<sun	sequence-specific DNA binding transcription factor;(source:Araport11)		DNA-binding transcription factor ac	43.215401	1.099000343	0.35466509	3.098698938	0.001943724	0.022481884
PITA_000036885	High quality	AT1G68920	MA_96671g0010	shade<sun	basic helix-loop-helix (bHLH) DNA-binding	CIB1 LIKE PROTEIN 1 (CIL1)	DNA-binding transcription factor ac	14.623265	2.1753608	0.61768975	3.521769299	0.000428677	0.006093456
PITA_000037155	High quality	AT2G16060	MA_32466g0010	shade<sun	Encodes a class 1 nonsymbiotic hemoglot	HEMOGLOBIN 1 (HB1)	cell wall-GO:0005618,cellular resp	305.28977	-2.202535076	0.61582552	-3.576557006	0.000348149	0.005068454
PITA_000037272	High quality	AT2G22590	MA_940843g0010	shade<sun	UDP-Glycosyltransferase superfamily protein;(source:Araport11)		intracellular membrane-bounded o	7.9246342	-6.227154205	1.6725918	-3.72305675	0.000196825	0.003067743
PITA_000037341	High quality	AT4G30410	MA_136773g0010	shade<sun	sequence-specific DNA binding transcriptio	IBH1-LIKE 1 (IBL1)	brassinosteroid mediated signaling	70.717523	-22.84103454	4.40809223	-5.181614484	2.20E-07	9.17E-06
PITA_000037401	High quality	AT2G32260	MA_10435627g0010	shade<sun	phosphorylcholine cytidylyltransferase;(so	PHOSPHORYLCHOLINE CYTIDYLYLT	biosynthetic process-GO:0009058,c	17.516739	-20.95349401	4.41016289	-4.751183692	2.02E-06	4.57E-05
PITA_000037430	High quality	AT5G10530	MA_13256g0010	shade<sun	Concanavalin A-like lectin protein kinase f	L-TYPE LECTIN RECEPTOR KINASE I	defense response to bacterium-GO	45.995515	-8.769466674	1.48795542	-5.893635369	3.78E-09	3.06E-07

PITA_000037433	High quality	AT5G50180	MA_39561g0010	shade<sun	Protein kinase superfamily protein;(source:Araport11)		cytoplasm-GO:0005737,protein kin	122.80029	-0.564570226	0.20008737	-2.821618453	0.004778199	0.047444791
PITA_000037524	Low quality	AT5G36100	MA_10434951g0010	shade<sun	transmembrane protein;(source:Araport11)		biological_process_unknown-GO:0(	23.572508	-21.35301597	4.4094576	-4.84254933	1.28E-06	3.20E-05
PITA_000037580	High quality	AT1G08250	MA_7947g0030	shade<sun	Encodes a plastid-localized arogenate deh	AROGENATE DEHYDRATASE 6 (ADT	arogenate dehydratase activity-GO	1117.4872	-2.490052886	0.28000272	-8.892959761	5.95E-19	3.63E-16
PITA_000037674	High quality	AT1G69780	MA_238039g0010	shade>sun	Encodes a homeodomain leucine zipper c	(ATHB13)	cotyledon morphogenesis-GO:0048	96.86113	2.949370746	0.7546545	3.908239784	9.30E-05	0.001557097
PITA_000037697	Low quality	AT4G20360	MA_942991g0010	shade<sun	Nuclear transcribed, plastid localized EF-T	RAB GTPASE HOMOLOG E1B (ATRA	apoplast-GO:0048046,chloroplast-(	672.56461	-0.782126794	0.20860613	-3.749299139	0.000177329	0.00280139
PITA_000037699	High quality	AT1G67340	MA_219531g0010	shade<sun	HCP-like superfamily protein with MYND-type zinc finger;(source:Araport11)		nucleus-GO:0005634,protein bindi	219.53741	-1.946924999	0.65938237	-2.952649479	0.003150595	0.033856666
PITA_000037724	High quality	AT4G33865	MA_10436379g0010	shade<sun	Ribosomal protein S14p/S29e family protein;(source:Araport11)		cytosolic small ribosomal subunit-G	115.36098	-0.657066196	0.23020655	-2.854246282	0.004313907	0.043664246
PITA_000037764	High quality	AT5G43940	MA_10430507g0040	shade<sun	Encodes a glutathione-dependent formald	SENSITIVE TO HOT TEMPERATURES	alcohol dehydrogenase activity, zin	52.550218	-22.4390271	4.4083279	-5.09014474	3.58E-07	1.19E-05
PITA_000037768	Low quality	AT2G45470	MA_10436490g0010	shade>sun	Fasciclin-like arabinogalactan protein. Pos	FASCICLIN-LIKE ARABINOGALACTA	anchored component of membrane	65.46333	9.776300229	2.70391271	3.615612361	0.000299638	0.004420013
PITA_000037787	Low quality	AT5G67620	MA_76415g0010	shade>sun	PADRE protein up-regulated after infection by S. sclerotiorum.		cytoplasm-GO:0005737,microtubu	41.367891	0.986165794	0.32891422	2.998246124	0.002715383	0.02992946
PITA_000038005	High quality	AT2G20750	MA_10427897g0010	shade<sun	member of BETA-EXPANSINS. Naming con	EXPANSIN B1 (EXPB1)	chloroplast-GO:0009507,extracellu	141.53513	-0.744875979	0.22281556	-3.343016021	0.000828731	0.010764479
PITA_000038039	High quality	AT3G18890	MA_10429419g0010	shade<sun	NAD(P)-binding Rossmann-fold superfami	TRANSLOCON AT THE INNER ENVEI	chloroplast-GO:0009507,chloroplas	698.92292	-1.158145055	0.27649682	-4.188637942	2.81E-05	0.000518879
PITA_000038064	High quality	AT4G37530	MA_119796g0010	shade<sun	Peroxidase superfamily protein;(source:Araport11)		extracellular region-GO:0005576,h	7.7977638	-6.20554957	1.79945158	-3.448578251	0.000563546	0.007709022
PITA_000038178	High quality	AT4G38620	MA_2026g0010	shade>sun	Encodes a R2R3 MYB protein which is inv	MYB DOMAIN PROTEIN 4 (MYB4)	regulation of phenylpropanoid met	242.51277	1.905158425	0.67424522	2.825616501	0.004718972	0.047011153
PITA_000038318	High quality	AT2G26710	MA_65486g0010	shade>sun	Encodes a member of the cytochrome p4	PHYB ACTIVATION TAGGED SUPPR	brassinosteroid homeostasis-GO:0(	29.055064	1.088881103	0.38506976	2.827750202	0.004687637	0.046781373
PITA_000038360	High quality	AT4G36040	MA_57712g0010	shade>sun	Chaperone DnaJ-domain superfamily prot	DNAJ11 (J11)	chloroplast-GO:0009507,nucleus-G	8.0675273	2.561409092	0.82545514	3.103026419	0.001915525	0.02221593
PITA_000038493	High quality	AT5G20900	MA_19869g0010	shade<sun	jasmonate-zim-domain protein 12;(source	JASMONATE-ZIM-DOMAIN PROTEI	nucleus-GO:0005634,protein bindi	135.77834	-1.852862669	0.34458622	-5.377065543	7.57E-08	3.75E-06
PITA_000038632	High quality	AT5G64830	MA_10430592g0010	shade>sun	programmed cell death 2 C-terminal domain-containing protein;(source:Arap		cytoplasm-GO:0005737,molecular_	32.800129	0.949166263	0.33284032	2.85171661	0.004348385	0.043926406
PITA_000038638	High quality	AT5G05550	MA_10430713g0010	shade>sun	Encodes trihelix-domain transcription fact	VFP5 (VFP5)	DNA-binding transcription factor ac	64.831	1.738106094	0.39057962	4.450068524	8.58E-06	0.000171071
PITA_000038737	High quality	AT3G52500	MA_46900g0010	shade<sun	Eukaryotic aspartyl protease family protein;(source:Araport11)		cell wall-GO:0005618,extracellular	40.476212	-21.86659121	4.40860183	-4.959983247	7.05E-07	2.04E-05
PITA_000038786	High quality	AT1G76590	MA_14971g0010	shade>sun	PLATZ transcription factor family protein;	(PLATZ2)	biological_process_unknown-GO:0(	39.101364	1.650289657	0.33167808	4.975576514	6.51E-07	2.01E-05
PITA_000038886	High quality	AT5G05280	MA_181940g0010	shade>sun	Encodes a RING-finger E3 ligase protein th	DEFECTIVE IN ANTHER DEHISCENCI	anther dehiscence-GO:0009901,po	91.107516	10.25325612	1.67778613	6.111181821	9.89E-10	9.11E-08
PITA_000038919	High quality	AT1G24590	MA_77420g0010	shade<sun	Encodes a member of the ERF (ethylene r	DORNROSCHEN-LIKE (DRNL)	cotyledon development-GO:00488:	2837.1301	-3.342071408	0.76359954	-4.376733123	1.20E-05	0.000234069
PITA_000038969	High quality	AT5G61850	MA_108198g0010	shade>sun	Encodes transcriptional regulator that prc	LEAFY (LFY)	chromatin DNA binding-GO:00314(	42.446848	1.454693252	0.46114033	3.154556574	0.001607422	0.019163345
PITA_000039093	High quality	AT5G01880	MA_41514g0010	shade<sun	RING/U-box superfamily protein;(source:/	DAF-LIKE GENE 2 (DAFL2)	protein ubiquitination-GO:0016567	26.275109	-21.49994244	4.40924566	-4.876104457	1.08E-06	2.80E-05
PITA_000039171	High quality	AT5G52390	MA_55307g0020	shade<sun	PAR1 protein;(source:Araport11)		biological_process_unknown-GO:0(	52.550218	-22.4390271	4.4083279	-5.09014474	3.58E-07	1.19E-05
PITA_000039295	High quality	AT1G52740	MA_100300g0010	shade>sun	Encodes HTA9, a histone H2A protein. Los	HISTONE H2A PROTEIN 9 (HTA9)	chromatin silencing-GO:0006342,d	71.766463	9.908755716	1.32801378	7.461335034	8.57E-14	2.07E-11
PITA_000039352	High quality	AT1G58340	MA_2674g0010	shade>sun	Encodes a plant MATE (multidrug and tox	(ZF14)	antiporter activity-GO:0015297,chl	33.075393	1.194974252	0.396101	3.016842279	0.002554227	0.028450908
PITA_000039373	Low quality	AT1G68780	MA_903114g0010	shade<sun	RNI-like superfamily protein;(source:Araport11)		mitochondrion-GO:0005739,plasm	17.516739	-20.95349401	4.41016289	-4.751183692	2.02E-06	4.57E-05
PITA_000039391	High quality	AT1G07530	MA_45656g0030	shade<sun	Encodes a member of the GRAS family of	SCARECROW-LIKE 14 (SCL14)	cytosol-GO:0005829,DNA-binding t	70.717523	-22.84103454	4.40809223	-5.181614484	2.20E-07	9.17E-06
PITA_000039485	High quality	AT1G42970	MA_161458g0010	shade<sun	Encodes chloroplast localized glyceraldeh	GLYCERALDEHYDE-3-PHOSPHATE D	apoplast-GO:0048046,chloroplast-(	1045.8736	-0.855465439	0.24176018	-3.538487693	0.000402426	0.005773292
PITA_000039609	Low quality	AT3G24300	MA_469277g0010	shade<sun	Encodes a plasma membrane localized an	AMMONIUM TRANSPORTER 1;3 (A	ammonium transmembrane transp	1045.8224	-1.307369993	0.2136747	-6.118506239	9.45E-10	8.76E-08
PITA_000039677	Low quality	AT5G17520	MA_210362g0010	shade<sun	Encodes a maltose transporter that is exp	ROOT CAP 1 (RCP1)	carbohydrate metabolic process-G(	244.66223	-1.416704211	0.46462971	-3.04910381	0.002295252	0.025995932
PITA_000039730	High quality	AT5G23530	MA_131537g0010	shade<sun	carboxyesterase 18;(source:Araport11)	CARBOXYESTERASE 18 (CXE18)	carboxylic ester hydrolase activity-(	4440.5603	-3.020715684	0.67986436	-4.443115198	8.87E-06	0.000176422
PITA_000039736	High quality	AT2G28470	MA_72251g0010	shade>sun	putative beta-galactosidase (BGAL8 gene)	BETA-GALACTOSIDASE 8 (BGAL8)	beta-galactosidase activity-GO:000	81.572532	2.401935253	0.62871583	3.820382965	0.000133245	0.002159575
PITA_000040012	High quality	AT1G13260	MA_17571g0010	shade>sun	Encodes an AP2/B3 domain transcription	RELATED TO ABI3/VP1 1 (RAV1)	cellular response to hypoxia-GO:00	31.423985	1.575002522	0.47134925	3.341476656	0.00083334	0.010816106
PITA_000040134	Low quality	AT3G14200	MA_5156g0010	shade>sun	Chaperone DnaJ-domain superfamily protein;(source:Araport11)		biological_process_unknown-GO:0(	36.25909	1.086382608	0.32775136	3.314654736	0.000917564	0.011754364
PITA_000040209	High quality	AT5G17680	MA_135183g0010	shade<sun	disease resistance protein (TIR-NBS-LRR class);(source:Araport11)		ADP binding-GO:0043531,chloropl:	73.781949	-22.8989311	4.40806388	-5.19478205	2.05E-07	9.17E-06
PITA_000040257	High quality	AT5G13870	MA_10251013g0010	shade<sun	EXGT-A4, endoxyloglucan transferase,	XYLOGLUCAN ENDOTRANSGLUCOS	cell wall biogenesis-GO:0042546,ex	252.36406	-4.404003285	0.73908601	-5.958715554	2.54E-09	2.12E-07
PITA_000040347	High quality	AT5G45340	MA_295001g0010	shade<sun	Encodes a protein with ABA 8'-hydroxylas	CYTOCHROME P450, FAMILY 707,	(+)-abscisic acid 8'-hydroxylase acti	179.34146	-1.108775886	0.19177367	-5.781689843	7.40E-09	5.44E-07
PITA_000040507	High quality	AT5G13170	MA_10429987g0020	shade<sun	Encodes a member of the SWEET sucrose	SENESCENCE-ASSOCIATED GENE 29	carbohydrate transport-GO:000864	190.0003	-10.81949645	1.92636098	-5.616546715	1.95E-08	1.20E-06
PITA_000040536	Low quality	AT1G78690	MA_10436600g0010	shade>sun	Encodes a lysoglycerophospholipid O-acyl	(At1g78690p)	glycerophospholipid metabolic proc	28.630238	8.582963228	1.47746441	5.809252097	6.28E-09	4.69E-07
PITA_000040591	High quality	AT5G49160	MA_10436985g0010	shade<sun	Encodes a cytosine methyltransferase ME	METHYLTRANSFERASE 1 (MET1)	C-5 methylation of cytosine-GO:00(	314.52708	-0.479592346	0.15734385	-3.048052778	0.002303295	0.026063973
PITA_000040698	High quality	AT3G13540	MA_53373g0010	shade<sun	Encodes a member of the MYB family of t	MYB DOMAIN PROTEIN 5 (MYB5)	cell differentiation-GO:0030154,DN	1257.9641	-4.465121465	0.664858	-6.715902404	1.87E-11	2.59E-09
PITA_000040742	High quality	AT1G26560	MA_356467g0010	shade<sun	beta glucosidase 40;(source:Araport11)	BETA GLUCOSIDASE 40 (BGLU40)	apoplast-GO:0048046,beta-glucosi	234.06998	-1.645518226	0.55977523	-2.939605272	0.003286306	0.035006542
PITA_000040853	High quality	AT5G14320	MA_10431241g0010	shade<sun	Ribosomal protein S13/S18 family;(source	EMBRYO DEFECTIVE 3137 (EMB31:	chloroplast-GO:0009507,chloroplas	394.31188	-0.64996989	0.19882078	-3.269124491	0.001078808	0.013489317
PITA_000040956	Low quality	AT3G22850	MA_10432644g0010	shade>sun	aluminum induced protein with YGL and LRDR motifs;(source:Araport11)		cytoplasm-GO:0005737,cytosol-GC	24.174172	2.89935436	0.51436667	5.636746137	1.73E-08	1.11E-06
PITA_000041111	Low quality	AT4G33590	MA_41383g0010	shade<sun	transmembrane protein;(source:Araport11)		extracellular region-GO:0005576,tr	469.71695	-0.906585274	0.18331959	-4.945381259	7.60E-07	2.19E-05
PITA_000041197	High quality	AT1G75450	MA_138486g0010	shade>sun	This gene used to be called AtCKX6. It enc	CYTOKININ OXIDASE 5 (CKX5)	cytokinin catabolic process-GO:000	79.435407	2.512049444	0.39936118	6.290169338	3.17E-10	3.22E-08
PITA_000041247	High quality	AT1G76880	MA_79519g0010	shade>sun	DF1 is a putative transcription factor requ	(DF1)	DNA-binding transcription factor ac	44.697082	1.195774717	0.33321414	3.588607385	0.000332449	0.004850915
PITA_000041259	Low quality	AT1G76940	MA_10308664g0010	shade>sun	RNA-binding (RRM/RBD/RNP motifs) fam	(NSRA)	mRNA binding-GO:0003729,nucleu	162.0337	24.31540594	4.32827129	5.617810044	1.93E-08	1.19E-06
PITA_000041293	High quality	AT1G79930	MA_10430733g0010	shade<sun	encodes high molecular weight heat shoc	HEAT SHOCK PROTEIN 91 (HSP91)	ATP binding-GO:0005524,cytoplasr	52.550218	-22.4390271	4.4083279	-5.09014474	3.58E-07	1.19E-05
PITA_000041395	High quality	AT1G67850	MA_10431458g0010	shade<sun	lysine ketoglutarate reductase trans-splicing protein (DUF707);(source:Arapor		biological_process_unknown-GO:0(	60.714318	-22.53151824	4.4082046	-5.11126871	3.20E-07	1.18E-05
PITA_000041417	High quality	AT5G60200	MA_7537g0010	shade>sun	Encodes a Dof-type transcription factor. P	TARGET OF MONOPTEROS 6 (TMO	DNA-binding transcription factor ac	51.992668	0.906791748	0.27011454	3.357063861	0.000787749	0.010302697
PITA_000041425	Low quality	AT3G01480	MA_7732g0010	shade>sun	Encodes a chloroplast cyclophilin function	CYCLOPHILIN 38 (CYP38)	chloroplast-GO:0009507,chloroplas	27.278012	8.512987848	2.28170866	3.730970564	0.000190744	0.002980207
PITA_000041426	Low quality	AT1G03230	MA_10135085g0010	shade<sun	Eukaryotic aspartyl protease family prote	SECRETED ASPARTIC PROTEASE 1 (	aspartic-type endopeptidase activit	23.572508	-21.35301597	4.4094576	-4.84254933	1.28E-06	3.20E-05
PITA_000041509	Low quality	AT1G74160	MA_4736g0010	shade<sun	Member of a small gene family in Arabido	TON1 RECRUITING MOTIF 4 (TRM4	molecular_function_unknown-GO:(	60.714318	-22.53151824	4.4082046	-5.11126871	3.20E-07	1.18E-05
PITA_000041777	Low quality	AT3G18930	MA_10260361g0010	shade>sun	RING/U-box superfamily protein;(source:/	ARABIDOPSIS T??XICOS EN LEVADU	chloroplast-GO:0009507,nucleus-G	58.230151	1.213855378	0.36991331	3.28145908	0.001032715	0.012969969
PITA_000041803	High quality	AT3G15850	MA_112563g0010	shade<sun	Chloroplastic enzyme responsible for the	FATTY ACID DESATURASE 5 (FAD5)	1-18:1-2-16:0-monogalactosyldiacy	4835.9442	-1.206008776	0.22357737	-5.394145063	6.89E-08	3.48E-06
PITA_000041996	High quality	AT3G18030	MA_329767g0010	shade<sun	flavin mononucleotide flavoprotein involv	HAL3-LIKE PROTEIN A (HAL3A)	coenzyme A biosynthetic process-G	142.86414	-0.905018714	0.24896984	-3.635053618	0.000277923	0.004162016
PITA_000042025	High quality	AT5G55000	MA_10436992g0010	shade<sun	FH protein interacting protein FIP2	(FIP2)	extracellular region-GO:0005576,p	21.020087	-21.19302767	4.40970434	-4.805997415	1.54E-06	3.72E-05
PITA_000042062	High quality	AT2G35800	MA_60518g0010	shade>sun	Encodes a predicted calcium-dependent S	S-ADENOSYL METHIONINE TRANSP	calcium ion binding-GO:0005509,cl	41.963715	1.334054661	0.39472369	3.379717718	0.000725603	0.009587846
PITA_000042174	Low quality	AT1G04120	MA_281476g0010	shade<sun	encodes a high-affinity inositol hexakisph	ATP-BINDING CASSETTE C5 (ABCC5	ABC-type xenobiotic transporter ac	476.48278	-2.23972785	0.70819488	-3.162586894	0.001563741	0.018677364
PITA_000042418	High quality	AT4G34050	MA_4334g0010	shade>sun	Methyltransferase in the lignin biosynthet	CAFFEOYL COENZYME A O-METHYI	caffeoyl-CoA O-methyltransferase a	39.412484	2.147286421	0.57475852	3.735980152	0.000186985	0.00293582
PITA_000042475	High quality	AT3G04450	MA_66960g0010	shade<sun	Homeodomain-like superfamily protein;(source:Araport11)		DNA-binding transcription factor ac	85.107171	-0.725529872	0.23398944	-3.100695002	0.00193067	0.022351089
PITA_000042488	Low quality	AT4G34090	MA_184970g0010	shade<sun	cyclin delta-3;(source:Araport11)	PROTEIN IN CHLOROPLAST ATPASE	chloroplast-GO:0009507,chloroplas	791.69693	-0.839121048	0.29094983	-2.884074737	0.003925656	0.040211277
PITA_000042515	High quality	AT1G47260	MA_10435782g0010	shade<sun	Encodes mitochondrial gamma carbonic a	GAMMA CARBONIC ANHYDRASE 2	anther dehiscence-GO:0009901,cai	164.92222	-0.649324071	0.17583591	-3.692784159	0.000221812	0.003417671
PITA_000042741	High quality	AT4G01800	MA_8475350g0010	shade<sun	Encodes the ATPase subunit of the chloro	ALBINO OR GLASSY YELLOW 1 (AGY	ABC-type protein transporter activi	262.78729	-0.801458993	0.20267423	-3.954419893	7.67E-05	0.00130803
PITA_000042846	High quality	AT1G23740	MA_10436234g0010	shade<sun	AOR is an alkenal/one oxidoreductase tha	ALKENAL/ONE OXIDOREDUCTASE (	2-alkenal reductase (NADP+) activit	26.107157	-21.49092332	4.40925805	-4.87404527	1.09E-06	2.81E-05
PITA_000042892	High quality	AT5G07470	MA_102643g0010	shade<sun	ubiquitous enzyme that repairs oxidativel	PEPTIDEMETHIONINE SULFOXIDE F	cellular response to oxidative stress	196.16219	-2.054051149	0.23661358	-8.68103642	3.92E-18	2.01E-15
PITA_000042912	High quality	AT4G34760	MA_7654907g0010	shade>sun	SAUR-like auxin-responsive protein family	SMALL AUXIN UPREGULATED RNA	mitochondrion-GO:0005739,molec	26.659426	8.479898903	2.74990428	3.083706934	0.002044389	0.02344501
PITA_000042993	High quality	AT3G52190	MA_307089g0010	shade>sun	Encodes a plant specific protein structura	PHOSPHATE TRANSPORTER TRAFFI	cellular response to phosphate star	90.764575	22.54018959	4.32849343	5.207398364	1.92E-07	8.62E-06
PITA_000043075	High quality	AT1G17420	MA_10429541g0010	shade>sun	LOX3 encode a Lipoxygenase. Lipoxygena	LIPOXYGENASE 3 (LOX3)	anther dehiscence-GO:0009901,an	223.4206	1.429390658	0.30441374	4.695552326	2.66E-06	5.74E-05
PITA_000043149	High quality	AT3G60070	MA_140361g0010	shade<sun	Major facilitator superfamily protein;(source:Araport11)		carbohydrate transport-GO:000864	133.40556	-10.30857581	2.12575144	-4.849379661	1.24E-06	3.17E-05
PITA_000043173	High quality	AT2G20940	MA_17250g0010	shade<sun	transmembrane protein, putative (DUF1279);(source:Araport11)		biological_process_unknown-GO:0(	118.98586	-0.795730026	0.20364034	-3.907526594	9.32E-05	0.001560678
PITA_000043373	Low quality	AT1G07160	MA_10432464g0020	shade>sun	Protein phosphatase 2C family protein;(source:Araport11)		chloroplast-GO:0009507,magnesiu	8.0164666	6.746480329	2.3300614	2.895408818	0.00378665	0.039100217
PITA_000043456	High quality	AT1G34060	MA_10436004g0020	shade<sun	Pyridoxal phosphate (PLP)-dependent trai	TRYPTOPHAN AMINOTRANSFERAS	carbon-sulfur lyase activity-GO:001	337.65561	-2.003452414	0.65808986	-3.044344743	0.002331878	0.026329242
PITA_000043522	Low quality	AT5G23570	MA_137185g0010	shade>sun	Required for posttranscriptional gene sile	SUPPRESSOR OF GENE SILENCING :	cytoplasm-GO:0005737,cytosol-GC	92.953463	0.677879881	0.23913183	2.834753893	0.0045861	0.045947124
PITA_000043545	Low quality	AT3G57120	MA_10158g0020	shade<sun	Protein kinase superfamily protein;(source:Araport11)		ATP binding-GO:0005524,phospho	35.358761	-21.90137695	4.40877489	-4.967678662	6.78E-07	2.02E-05
PITA_000043744	High quality	AT1G22160	MA_6300g0010	shade<sun	senescence-associated family protein (DUF581);(source:Araport11)		cytosol-GO:0005829,kinase binding	268.77246	-1.602291623	0.19937507	-8.036569508	9.24E-16	2.99E-13
PITA_000043782	High quality	AT4G35740	MA_10427022g0010	shade>sun	Encodes RECQ3, an ATP-dependent helica	(RecQl3)	3'-5' DNA helicase activity-GO:0043	82.641472	10.11249851	2.26254199	4.469529651	7.84E-06	0.000157324
PITA_000043841	High quality	AT3G07880	MA_10430494g0010	shade<sun	RhoGTPase GDP dissociation inhibitor (Rh	SUPERCENTIPEDE1 (SCN1)	cell tip growth-GO:0009932,cytopla	384.21537	-1.192915544	0.3583536	-3.32887839	0.000871965	0.011243339
PITA_000043903	Low quality	AT3G08640	MA_107207g0010	shade<sun	alphavirus core family protein (DUF3411)	RETICULATA-RELATED 3 (RER3)	chloroplast-GO:0009507,chloroplas	137.13654	-0.789654815	0.18864267	-4.185981975	2.84E-05	0.000524605
PITA_000043913	High quality	AT4G24520	MA_10433352g0010	shade>sun	Encodes a cyp450 reductase likely to be ir	P450 REDUCTASE 1 (ATR1)	chloroplast-GO:0009507,cytosol-G(	42.133046	9.140725959	1.72803293	5.289671175	1.23E-07	5.77E-06
PITA_000044043	Low quality	AT1G03440	MA_88551g0010	shade<sun	Leucine-rich repeat (LRR) family protein;(source:Araport11)		defense response-GO:0006952,mit	52.550218	-22.4390271	4.4083279	-5.09014474	3.58E-07	1.19E-05
PITA_000044051	Low quality	AT1G62310	MA_132844g0010	shade<sun	Encodes a probable H3K9me2 demethyla	(JMJ29)	chromatin-GO:0000785,chromatin	70.717523	-22.84103454	4.40809223	-5.181614484	2.20E-07	9.17E-06
PITA_000044086	Low quality	AT5G48485	MA_106330g0010	shade<sun	Encodes a putative apoplastic lipid transfe	DEFECTIVE IN INDUCED RESISTANC	endoplasmic reticulum-GO:000578	525.79922	-0.766947515	0.23865389	-3.213639317	0.001310642	0.016020487
PITA_000044353	High quality	AT1G14340	MA_371748g0010	shade>sun	ACD11 binding partner, negatively regula	BPA1-LIKE 3 (BPL3)	biological_process_unknown-GO:0(	36.189576	3.267584747	0.59853125	5.459338577	4.78E-08	2.51E-06
PITA_000044382	High quality	AT5G02070	MA_418666g0010	shade>sun	Protein kinase family protein;(source:Araport11)		cell surface receptor signaling path	61.552172	2.039828852	0.42239489	4.829198648	1.37E-06	3.37E-05
PITA_000044507	Low quality	AT4G02420	MA_76027g0010	shade<sun	Concanavalin A-like lectin protein kinase f	L-TYPE LECTIN RECEPTOR KINASE I\	defense response to bacterium-GO	259.70118	-11.26981471	1.94797938	-5.785387058	7.23E-09	5.34E-07
PITA_000044575	High quality	AT3G61250	MA_128244g0010	shade>sun	LATE MERISTEM IDENTITY2 (LMI2) is a tai	MYB DOMAIN PROTEIN 17 (MYB17	DNA-binding transcription factor ac	13.876958	2.170824047	0.66698343	3.254689628	0.001135163	0.014096595
PITA_000044603	Low quality	AT1G19835	MA_10428427g0010	shade>sun	TCS1 encodes a coiled-coil domain protei	TRICHOME CELL SHAPE 1 (TCS1)	cortical microtubule-GO:0055028,c	108.02247	23.75154617	4.32841291	5.487356833	4.08E-08	2.16E-06
PITA_000044658	Low quality	AT5G19140	MA_65113g0010	shade<sun	aluminum induced protein with YGL and L	(AILP1)	chloroplast-GO:0009507,cytoplasm	19.38074	-1.624648875	0.50770263	-3.200000891	0.001374272	0.016678838
PITA_000044668	High quality	AT4G18750	MA_322292g0010	shade<sun	Encodes a pentatricopeptide (PPR) protei	DEFECTIVELY ORGANIZED TRIBUTA	chloroplast-GO:0009507,chloroplas	134.43463	-1.918217965	0.32624396	-5.879704109	4.11E-09	3.25E-07
PITA_000044855	High quality	AT2G30200	MA_10428989g0010	shade<sun	Malonyl-ACP expressed in developing see	EMBRYO DEFECTIVE 3147 (EMB31	[acyl-carrier-protein] S-malonyltrar	148.94974	-0.996841887	0.19780633	-5.039484332	4.67E-07	1.49E-05
PITA_000044859	High quality	AT3G27810	MA_10320g0010	shade>sun	Encodes a member of the R2R3-MYB tran	MYB DOMAIN PROTEIN 21 (MYB21	gibberellic acid mediated signaling	35.169661	1.495857984	0.36113383	4.142115407	3.44E-05	0.000624975
PITA_000044876	High quality	AT2G31750	MA_10428919g0010	shade<sun	Encodes an auxin glycosyltransferase that	UDP-GLUCOSYL TRANSFERASE 74D	auxin metabolic process-GO:00098	962.40101	-13.16002358	1.56641303	-8.401375188	4.41E-17	1.88E-14
PITA_000044970	High quality	AT2G26250	MA_309409g0010	shade>sun	epidermis-specific, encodes KCS10, a puta	3-KETOACYL-COA SYNTHASE 10 (KC	3-oxo-arachidoyl-CoA synthase acti	9.5588836	2.16151912	0.69125736	3.126938298	0.00176637	0.020730157
PITA_000045073	Low quality	AT4G37870	MA_101646g0010	shade>sun	Encodes a phosphoenolpyruvate carboxyl	PHOSPHOENOLPYRUVATE CARBOX	cellular response to phosphate star	36.994749	2.567527889	0.70971201	3.617703889	0.000297228	0.004389515
PITA_000045099	Low quality	AT2G01610	MA_8433628g0010	shade<sun	Plant invertase/pectin methylesterase inhibitor superfamily protein;(source:A		enzyme inhibitor activity-GO:0004(	26.275109	-21.49994244	4.40924566	-4.876104457	1.08E-06	2.80E-05
PITA_000045210	Low quality	AT1G18485	MA_33151g0010	shade<sun	Pentatricopeptide repeat (PPR) superfamily protein;(source:Araport11)		mitochondrion-GO:0005739,mRNA	264.38605	-1.457022828	0.27529071	-5.292669791	1.21E-07	5.72E-06
PITA_000045397	Low quality	AT5G56550	MA_345439g0010	shade>sun	Encodes OXIDATIVE STRESS 3 (OXS3)&#6!	OXIDATIVE STRESS 3 (OXS3)	molecular_function_unknown-GO:(	14.205145	3.794955068	0.82343456	4.608690518	4.05E-06	8.48E-05
PITA_000045419	High quality	AT1G29660	MA_1194g0010	shade<sun	GDSL-motif esterase/acyltransferase/lipase. Enzyme group with broad substr		apoplast-GO:0048046,extracellular	30.700459	-8.186833525	1.54211504	-5.308834489	1.10E-07	5.30E-06
PITA_000045438	High quality	AT4G20140	MA_10428862g0010	shade>sun	Encodes GASSHO1 (GSO1), a putative leu	GASSHO1 (GSO1)	Casparian strip-GO:0048226,embry	10.306886	7.109058514	2.16098088	3.289736884	0.001002811	0.012668962
PITA_000045440	Low quality	AT5G41890	MA_117003g0010	shade<sun	GDSL-motif esterase/acyltransferase/lipase. Enzyme group with broad substr		hydrolase activity, acting on ester b	16.211824	-2.170542861	0.63324738	-3.427638103	0.000608857	0.008258262
PITA_000045482	High quality	AT5G54130	MA_10437257g0040	shade<sun	Calcium-binding endonuclease/exonuclease/phosphatase family;(source:Arap		3'-5'-exoribonuclease activity-GO:0	566.41633	-1.057375518	0.14866748	-7.112352617	1.14E-12	2.09E-10
PITA_000045488	Low quality	AT3G62630	MA_101436g0010	shade>sun	stress response NST1-like protein (DUF1645);(source:Araport11)		biological_process_unknown-GO:0(	21.926302	1.976675433	0.68468347	2.886991612	0.003889446	0.039984317
PITA_000045570	High quality	AT1G07230	MA_10432139g0010	shade>sun	non-specific phospholipase C1;(source:Ar	NON-SPECIFIC PHOSPHOLIPASE C1	Golgi cis cisterna-GO:0000137,hydr	31.517581	8.721955618	1.54380615	5.64964431	1.61E-08	1.04E-06
PITA_000045571	Low quality	AT2G38150	MA_10436829g0020	shade>sun	alpha 1,4-glycosyltransferase family protein;(source:Araport11)		Golgi apparatus-GO:0005794,trans	31.779658	2.472767837	0.76880915	3.216361101	0.001298274	0.015884468
PITA_000045591	High quality	AT3G57560	MA_623831g0010	shade<sun	encodes a N-acetylglutamate kinase, invo	N-ACETYL-L-GLUTAMATE KINASE (N	acetylglutamate kinase activity-GO	123.23605	-0.781014748	0.19784705	-3.947568406	7.89E-05	0.001338005
PITA_000045597	Low quality	AT5G04820	MA_61037g0010	shade>sun	ovate family protein 13;(source:Araport11	OVATE FAMILY PROTEIN 13 (OFP13	negative regulation of transcription	30.941232	1.369166034	0.37844902	3.617834794	0.000297078	0.004389515
PITA_000045651	High quality	AT5G48740	MA_9168890g0010	shade<sun	Leucine-rich repeat protein kinase family protein;(source:Araport11)		chloroplast-GO:0009507,kinase act	238.08538	-11.14495973	1.99694487	-5.581005224	2.39E-08	1.44E-06
PITA_000045711	High quality	AT3G17020	MA_139433g0010	shade>sun	Adenine nucleotide alpha hydrolases-like superfamily protein;(source:Araport		cytoplasm-GO:0005737,cytosol-GC	38.055305	1.335210248	0.4092695	3.262423014	0.001104642	0.013778697
PITA_000045734	High quality	AT3G11180	MA_77600g0010	shade<sun	One of 4 paralogs encoding a 2-oxoglutar	JASMONATE-INDUCED OXYGENASE	cytoplasm-GO:0005737,dioxygena:	40.476212	-21.86659121	4.40860183	-4.959983247	7.05E-07	2.04E-05
PITA_000045962	Low quality	AT1G20770	MA_10430983g0010	shade<sun	coiled-coil protein;(source:Araport11)		extrinsic component of endoplasmi	99.861728	-0.878532371	0.25635999	-3.426948111	0.000610406	0.008274891
PITA_000045973	High quality	AT5G40380	MA_10436076g0010	shade>sun	Encodes a cysteine-rich receptor-like prot	CYSTEINE-RICH RLK (RECEPTOR-LIK	chloroplast-GO:0009507,kinase act	75.946742	1.610581103	0.353533	4.555674007	5.22E-06	0.000107492
PITA_000045996	High quality	AT1G28590	MA_60155g0010	shade<sun	GDSL-motif esterase/acyltransferase/lipase. Enzyme group with broad substr		extracellular region-GO:0005576,h	397.97493	-1.637904998	0.36454539	-4.493007002	7.02E-06	0.000142271
PITA_000046138	Low quality	AT2G21270	MA_122976g0010	shade<sun	ubiquitin fusion degradation 1;(source:Ar	UBIQUITIN FUSION DEGRADATION	chloroplast-GO:0009507,cytosol-G(	60.714318	-22.53151824	4.4082046	-5.11126871	3.20E-07	1.18E-05
PITA_000046196	High quality	AT5G17450	MA_101835g0010	shade>sun	Heavy metal transport/detoxification sup	HEAVY METAL ASSOCIATED ISOPRE	cadmium ion homeostasis-GO:005!	159.47715	11.06073696	2.12270236	5.210686706	1.88E-07	8.54E-06
PITA_000046249	High quality	AT5G67385	MA_10429476g0010	shade>sun	Phototropic-responsive NPH3 family prot	NRL PROTEIN FOR CHLOROPLAST M	defense response to bacterium-GO	41.444115	0.966436723	0.34024333	2.84042815	0.004505302	0.045303298
PITA_000046293	High quality	AT5G60530	MA_182721g0010	shade<sun	late embryogenesis abundant protein-related / LEA protein-like protein;(sourc		cytoplasm-GO:0005737,molecular_	2471.9253	-1.693425552	0.29799569	-5.682718291	1.33E-08	9.00E-07
PITA_000046349	High quality	AT2G24630	MA_10429365g0010	shade<sun	encodes a XyG glucan synthase; gene sim	(ATCSLC08)	cell wall organization-GO:0071555,	82.62278	-0.818286645	0.25482778	-3.211135992	0.001322114	0.016114556
PITA_000046471	High quality	AT5G39830	MA_141320g0010	shade<sun	Encodes DEG8. Forms a hexamer with DE	DEGRADATION OF PERIPLASMIC PI	chloroplast-GO:0009507,chloroplas	70.717523	-22.84103454	4.40809223	-5.181614484	2.20E-07	9.17E-06
PITA_000046579	High quality	AT5G61780	MA_74605g0010	shade>sun	Involved in the regulation of AtGA20ox3	TUDOR-SN PROTEIN 2 (TUDOR2)	cellular response to heat-GO:0034(	432.08988	25.50658086	4.32809423	5.89325914	3.79E-09	3.06E-07
PITA_000046733	Low quality	AT1G45170	MA_69201g0010	shade<sun	outer envelope pore 24B-like protein;(source:Araport11)		etioplast membrane-GO:0034426,i	125.17284	-0.687196818	0.19221361	-3.575172574	0.000349997	0.005089564
PITA_000046824	Low quality	AT1G09760	MA_764890g0010	shade<sun	U2 small nuclear ribonucleoprotein A;(sou	U2 SMALL NUCLEAR RIBONUCLEOI	Cajal body-GO:0015030,cytoplasm	434.80613	-0.925847252	0.20035607	-4.621009251	3.82E-06	8.02E-05
PITA_000046829	High quality	AT5G23960	MA_10426561g0010	shade<sun	Encodes a sesquiterpene synthase involve	TERPENE SYNTHASE 21 (TPS21)	(-)-E-beta-caryophyllene synthase a	52.550218	-22.4390271	4.4083279	-5.09014474	3.58E-07	1.19E-05
PITA_000047007	Low quality	AT1G72470	MA_89549g0010	shade>sun	A member of EXO70 gene family, putative	EXOCYST SUBUNIT EXO70 FAMILY	cytosol-GO:0005829,exocyst-GO:0(	162.0337	24.31540594	4.32827129	5.617810044	1.93E-08	1.19E-06
PITA_000047072	High quality	AT4G10780	MA_212337g0010	shade<sun	LRR and NB-ARC domains-containing disease resistance protein;(source:Arap(		ADP binding-GO:0043531,chloropl:	441.78784	-12.03698173	1.52093917	-7.914176982	2.49E-15	7.50E-13
PITA_000047173	High quality	AT4G10490	MA_113081g0010	shade<sun	2-oxoglutarate (2OG) and Fe(II)-depender	DMR6-LIKE OXYGENASE 2 (DLO2)	chloroplast-GO:0009507,defense r(	82.311914	-1.76867063	0.62187255	-2.844104658	0.004453642	0.044901124
PITA_000047195	High quality	AT3G51680	MA_18498g0010	shade>sun	NAD(P)-binding Rossmann-fold superfami	SHORT-CHAIN DEHYDROGENASE/R	cytoplasm-GO:0005737	81.016852	23.32680868	4.3285545	5.389052785	7.08E-08	3.53E-06
PITA_000047332	High quality	AT3G25250	MA_55799g0010	shade<sun	Arabidopsis protein kinase The mRNA is c	AGC2 KINASE 1 (AGC2-1)	cellular response to hypoxia-GO:00	469.13133	-1.701794828	0.53938183	-3.155083713	0.001604521	0.019137664
PITA_000047341	High quality	AT5G21940	MA_102706g0010	shade>sun	hybrid signal transduction histidine kinase M-like protein;(source:Araport11)		nucleus-GO:0005634,protein bindi	58.999792	2.430365239	0.39152424	6.207445196	5.39E-10	5.24E-08
PITA_000047368	Low quality	AT1G25560	MA_23673g0010	shade>sun	Encodes a member of the RAV transcripti	TEMPRANILLO 1 (TEM1)	cellular response to hypoxia-GO:00	114.52095	10.58300869	1.29828259	8.151544821	3.59E-16	1.26E-13
PITA_000047508	Low quality	AT1G63850	MA_10437242g0020	shade<sun	BTB/POZ domain-containing protein;(source:Araport11)		nucleus-GO:0005634,protein ubiqu	53.038142	-22.45050813	4.40831978	-5.092758525	3.53E-07	1.19E-05
PITA_000047517	Low quality	AT3G03150	MA_10436059g0010	shade>sun	hypothetical protein;(source:Araport11)		biological_process_unknown-GO:0(	35.590883	0.963475644	0.32242046	2.988258393	0.002805722	0.030757251
PITA_000047558	High quality	AT5G23140	MA_207420g0010	shade<sun	One of several nuclear-encoded ClpPs (ca	NUCLEAR-ENCODED CLP PROTEASE	ATPase binding-GO:0051117,ATP-c	317.46212	-0.600710536	0.17794078	-3.375901442	0.000735743	0.009696809
PITA_000047584	High quality	AT5G57050	MA_26265g0010	shade>sun	Encodes a protein phosphatase 2C and is	ABA INSENSITIVE 2 (ABI2)	cytoplasm-GO:0005737,cytosol-GC	22.012202	1.580867289	0.49854304	3.170974519	0.001519285	0.018231416
PITA_000047748	High quality	AT4G38620	MA_22140g0010	shade<sun	Encodes a R2R3 MYB protein which is inv	MYB DOMAIN PROTEIN 4 (MYB4)	regulation of phenylpropanoid met	204.8016	-4.656800238	0.67748814	-6.873626191	6.26E-12	9.48E-10
PITA_000047765	High quality	AT5G18670	MA_21789g0010	shade>sun	putative beta-amylase BMY3 (BMY3)	BETA-AMYLASE 3 (BMY3)	beta-amylase activity-GO:0016161,	47.87412	1.906090547	0.32675764	5.833346572	5.43E-09	4.17E-07
PITA_000047782	Low quality	AT4G36180	MA_58502g0010	shade<sun	LRR-RLK which regulates lateral root deve	MUSTACHES-LIKE (MUL)	kinase activity-GO:0016301,plasma	86.383756	-1.967862428	0.2750527	-7.154492305	8.40E-13	1.58E-10
PITA_000047857	Low quality	AT4G08700	MA_93277g0010	shade>sun	Member of a family of proteins related to	PURINE PERMEASE 13 (PUP13)	membrane-GO:0016020,purine nu	70.94086	9.891994194	1.68739848	5.862275158	4.57E-09	3.55E-07
PITA_000047880	Low quality	AT5G23730	MA_23011g0010	shade<sun	Encodes REPRESSOR OF UV-B PHOTOMO	REPRESSOR OF UV-B PHOTOMORP	chloroplast organization-GO:00096	870.65853	-0.463565228	0.14782029	-3.136005458	0.00171266	0.020220287
PITA_000047960	High quality	AT1G06030	MA_104118g0010	shade<sun	Encodes a member of the fructokinase ge	FRUCTOKINASE 6 (FRK6)	cytoplasm-GO:0005737,cytosol-GC	447.8022	-1.044491304	0.36474748	-2.86360118	0.00418855	0.042513037
PITA_000047961	High quality	AT5G26170	MA_76002g0010	shade<sun	member of WRKY Transcription Factor; G	WRKY DNA-BINDING PROTEIN 50 (	defense response to fungus-GO:00!	1681.3621	-3.18945077	0.52615639	-6.061792356	1.35E-09	1.18E-07
PITA_000048002	High quality	AT3G07470	MA_10434011g0010	shade<sun	transmembrane protein, putative (Protein of unknown function, DUF538);(sou		biological_process_unknown-GO:0(	1695.3024	-2.737124438	0.37114403	-7.374830868	1.65E-13	3.70E-11
PITA_000048103	High quality	AT4G02080	MA_14388g0010	shade<sun	A member of ARF-like GTPase family. A th	SECRETION-ASSOCIATED RAS SUPE	COPII vesicle coat-GO:0030127,enc	52.550218	-22.4390271	4.4083279	-5.09014474	3.58E-07	1.19E-05
PITA_000048165	High quality	AT3G03760	MA_962403g0010	shade>sun	LOB domain-containing protein 20;(sourc	LOB DOMAIN-CONTAINING PROTE	hormone-mediated signaling pathw	33.450206	1.863916734	0.49456467	3.768802813	0.000164032	0.00260418

PITA_000048203	Low quality	AT1G28280	MA_77052g0010	shade>sun	VQ motif-containing protein;(source:Arap	MPK3/6-TARGETED VQP 1 (MVQ1)	negative regulation of cellular defe	49.092585	0.870536533	0.27795567	3.13192585	0.001736637	0.020456213
PITA_000048230	High quality	AT5G20030	MA_35064g0010	shade<sun	Plant Tudor-like RNA-binding protein;(source:Araport11)		biological_process_unknown-GO:0	54.648778	-9.019762272	1.53392973	-5.880166535	4.10E-09	3.25E-07
PITA_000048231	High quality	AT5G20030	MA_35064g0010	shade>sun	Plant Tudor-like RNA-binding protein;(source:Araport11)		biological_process_unknown-GO:0	62.929471	1.729481752	0.57979338	2.982927709	0.002855054	0.031177922
PITA_000048290	High quality	AT1G73340	MA_266173g0010	shade<sun	ADTO1 is required for the activation of sy	ABIETANE DITERPENE OXIDASE 1 (/	brassinosteroid biosynthetic proces	296.98191	-1.635829437	0.5328491	-3.069967537	0.00214082	0.024397921
PITA_000048324	Low quality	AT5G18860	MA_10435980g0010	shade<sun	Encodes a purine nucleoside hydrolase ac	NUCLEOSIDE HYDROLASE 3 (NSH3)	adenosine catabolic process-GO:00	270.81055	-1.157987421	0.38919053	-2.975374101	0.002926314	0.031860995
PITA_000048407	Low quality	AT1G30370	MA_10435754g0010	shade<sun	Encodes a mitochondria-localized class III	DAD1-LIKE ACYLHYDROLASE (DLAH	extracellular region-GO:0005576,li	20.238106	-21.14561367	4.4097939	-4.795147836	1.63E-06	3.81E-05
PITA_000048531	High quality	AT3G48080	MA_10437224g0010	shade<sun	alpha/beta-Hydrolases superfamily protein;(source:Araport11)		lipid metabolic process-GO:000662	497.44426	-12.20752128	4.35482427	-2.803217885	0.005059548	0.049564688
PITA_000048695	High quality	AT2G47000	MA_10432169g0010	shade>sun	Encodes an auxin efflux transmembrane t	ATP-BINDING CASSETTE B4 (ABCB4	ABC-type xenobiotic transporter ac	108.02247	23.75154617	4.32841291	5.487356833	4.08E-08	2.16E-06
PITA_000048755	Low quality	AT1G66080	MA_10426052g0030	shade<sun	Encodes a glucose-regulated protein that	HIKESHI-LIKE PROTEIN1 (HLP1)	cellular response to hypoxia-GO:00	52.550218	-22.4390271	4.4083279	-5.09014474	3.58E-07	1.19E-05
PITA_000048836	High quality	AT1G26780	MA_4704g0010	shade<sun	Encodes LOF1 (LATERAL ORGAN FUSION1	MYB DOMAIN PROTEIN 117 (MYB1	DNA-binding transcription factor ac	129.79873	-1.059649979	0.33232038	-3.18863978	0.001429439	0.017299182
PITA_000048857	Low quality	AT2G16910	MA_126263g0010	shade<sun	Encodes a basic helix-loop helix transcript	ABORTED MICROSPORES (AMS)	anther wall tapetum development-	56.504628	-5.077429969	1.50548815	-3.372613701	0.000744583	0.009799588
PITA_000048928	High quality	AT5G56150	MA_612949g0010	shade>sun	ubiquitin-conjugating enzyme 30;(source:	UBIQUITIN-CONJUGATING ENZYMI	nucleus-GO:0005634,protein bindi	44.940401	1.256067337	0.29262738	4.292378082	1.77E-05	0.000335312
PITA_000049047	High quality	AT4G19170	MA_90573g0010	shade>sun	chloroplast-targeted member of a family	NINE-CIS-EPOXYCAROTENOID DIO›	carotene catabolic process-GO:001	78.026741	0.860465658	0.23077412	3.728605596	0.000192542	0.003004644
PITA_000049063	High quality	AT4G01310	MA_8012412g0010	shade<sun	Ribosomal L5P family protein;(source:Ara	PLASTID RIBOSOMAL PROTEINS OF	chloroplast-GO:0009507,chloropla	488.25358	-0.56146497	0.15771149	-3.560076526	0.000370747	0.005357834
PITA_000049150	High quality	AT4G02450	MA_74917g0010	shade<sun	Encodes one of two isoforms of a co-chap	(P23-1)	chaperone binding-GO:0051087,ch	35.358761	-21.90137695	4.40877489	-4.967678662	6.78E-07	2.02E-05
PITA_000049280	High quality	AT1G09740	MA_100052g0010	shade<sun	Adenine nucleotide alpha hydrolases-like superfamily protein;(source:Araport		cytoplasm-GO:0005737	964.32351	-3.215284514	0.78382512	-4.102043223	4.10E-05	0.000733352
PITA_000049317	Low quality	AT4G32880	MA_134472g0010	shade<sun	member of homeodomain-leucine zipper	HOMEOBOX GENE 8 (HB-8)	DNA-binding transcription factor ac	161.90353	-0.856430552	0.23304707	-3.674925271	0.000237919	0.003622254
PITA_000049369	High quality	AT5G67360	MA_78338g0010	shade<sun	Encodes a subtilisin-like serine protease e	(ARA12)	apoplast-GO:0048046,cell wall-GO	35.033479	-21.88927922	4.40878685	-4.964921176	6.87E-07	2.02E-05
PITA_000049461	Low quality	AT2G15890	MA_123309g0010	shade>sun	Encodes CBP1, a regulator of transcriptio	MATERNAL EFFECT EMBRYO ARRE!	cellular response to hypoxia-GO:00	12.476743	2.504977309	0.61970562	4.042205254	5.30E-05	0.000932576
PITA_000049547	High quality	AT4G05100	MA_10429939g0010	shade>sun	Member of the R2R3 factor gene family.	MYB DOMAIN PROTEIN 74 (MYB74	DNA-binding transcription factor ac	81.016852	23.32680868	4.3285545	5.389052785	7.08E-08	3.53E-06
PITA_000049588	High quality	AT5G03795	MA_61350g0010	shade<sun	Exostosin family protein;(source:Araport11)		nucleus-GO:0005634,protein glyco	40.476212	-21.86659121	4.40860183	-4.959983247	7.05E-07	2.04E-05
PITA_000049636	High quality	AT5G14040	MA_20200g0010	shade<sun	Encodes a mitochondrial phosphate trans	PHOSPHATE TRANSPORTER 3;1 (PH	cell wall-GO:0005618,chloroplast-C	163.78225	-0.528932755	0.18691507	-2.829802551	0.004657674	0.046518609
PITA_000049753	High quality	AT1G03080	MA_102054g0010	shade>sun	kinase interacting (KIP1-like) family protei	NETWORKED 1D (NET1D)	actin filament binding-GO:0051015	39.638392	0.923426201	0.32007702	2.885012464	0.003913982	0.040172041
PITA_000049768	Low quality	AT5G47750	MA_108401g0020	shade>sun	D6PK family kinase involved in pulse-indu	D6 PROTEIN KINASE LIKE 2 (D6PKL2	basipetal auxin transport-GO:0010!	56.586173	1.13359788	0.38203886	2.967231892	0.003004942	0.03260617
PITA_000049803	Low quality	AT1G21350	MA_98916g0010	shade<sun	Thioredoxin superfamily protein;(source:Araport11)		antioxidant activity-GO:0016209,ch	26.372638	-7.968022871	1.82890435	-4.356719303	1.32E-05	0.000255357
PITA_000049831	High quality	AT4G01970	MA_10436532g0010	shade<sun	Encodes a a raffinose and high affinity sta	STACHYOSE SYNTHASE (STS)	cytoplasm-GO:0005737,galactinol-	47.145015	-22.29213208	4.40843355	-5.056701391	4.27E-07	1.38E-05
PITA_000049968	High quality	AT5G42650	MA_10030456g0010	shade>sun	Encodes a member of the cytochrome p4	ALLENE OXIDE SYNTHASE (AOS)	allene oxide synthase activity-GO:0	48.258603	9.336122962	1.64932191	5.660582619	1.51E-08	1.01E-06
PITA_000050084	High quality	AT3G47550	MA_10435273g0010	shade>sun	RING/FYVE/PHD zinc finger superfamily protein;(source:Araport11)		cytoplasm-GO:0005737,membrane	20.075795	1.310020473	0.45498903	2.879235318	0.003986408	0.040768341
PITA_000050156	High quality	AT5G67500	MA_28816g0010	shade<sun	Encodes a voltage-dependent anion chan	VOLTAGE DEPENDENT ANION CHA	anion transmembrane transport-G(	303.06847	-0.606982156	0.1356443	-4.474807718	7.65E-06	0.000154091
PITA_000050239	High quality	AT5G26660	MA_357440g0010	shade<sun	myb domain protein 86;(source:Araport1	MYB DOMAIN PROTEIN 86 (MYB86	negative regulation of transcription	7545.0265	-1.231848554	0.30137587	-4.087415971	4.36E-05	0.000776796
PITA_000050361	Low quality	AT4G19670	MA_10053g0010	shade>sun	RING/U-box superfamily protein;(source:Araport11)		cytoplasm-GO:0005737,nucleic aci	58.672863	0.915655239	0.26587164	3.443974904	0.000573229	0.007837291
PITA_000050396	High quality	AT5G08250	MA_1163276g0010	shade<sun	Cytochrome P450 superfamily protein;(source:Araport11)		cellular_component_unknown-GO:	70.717523	-22.84103454	4.40809223	-5.181614484	2.20E-07	9.17E-06
PITA_000050533	High quality	AT5G63930	MA_10427804g0010	shade<sun	Leucine-rich repeat protein kinase family protein;(source:Araport11)		ATP binding-GO:0005524,chloropla	60.714318	-22.53151824	4.4082046	-5.11126871	3.20E-07	1.18E-05
PITA_000050612	High quality	AT3G12670	MA_1481g0010	shade>sun	Cytidine triphosphate synthase.	EMBRYO DEFECTIVE 2742 (emb274	CTP biosynthetic process-GO:0006	35.604539	1.04417492	0.35142415	2.971266835	0.00296574	0.032208085
PITA_000050673	High quality	AT3G10600	MA_78005g0010	shade<sun	Encodes a member of the cationic amino	CATIONIC AMINO ACID TRANSPOR	amino acid transmembrane transp	52.550218	-22.4390271	4.4083279	-5.09014474	3.58E-07	1.19E-05
PITA_000050690	High quality	AT2G47010	MA_10432238g0020	shade<sun	calcium/calcium/calmodulin-dependent Serine/Threonine-kinase;(source:Ara		cellular_component_unknown-GO:	60.714318	-22.53151824	4.4082046	-5.11126871	3.20E-07	1.18E-05
PITA_000050718	High quality	AT4G21970	MA_19074g0010	shade<sun	senescence regulator (Protein of unknown function, DUF584);(source:Araport		biological_process_unknown-GO:0	47.145015	-22.29213208	4.40843355	-5.056701391	4.27E-07	1.38E-05
PITA_000050721	Low quality	AT2G45260	MA_118728g0010	shade>sun	Negatively regulates autophagy by direct	CHIQUITA1 (CHIQ1)	autophagosome-GO:0005776,nega	283.05314	11.88862635	1.33468355	8.907449529	5.22E-19	3.43E-16
PITA_000050763	High quality	AT2G16500	MA_8291837g0010	shade<sun	Encodes a arginine decarboxylase (ADC),	ARGININE DECARBOXYLASE 1 (ADC	arginine catabolic process-GO:0006	130.0136	-0.849904943	0.20468799	-4.15219747	3.29E-05	0.000600617
PITA_000050812	High quality	AT1G02520	MA_180044g0010	shade>sun	Encodes an ATP-binding cassette (ABC) tr	ATP-BINDING CASSETTE B11 (ABCB	ATPase activity-GO:0016887,ATPas	51.723325	1.020156677	0.30287818	3.368207874	0.000756585	0.009930618
PITA_000050841	High quality	AT2G36970	MA_19907g0010	shade>sun	UDP-Glycosyltransferase superfamily protein;(source:Araport11)		transferase activity, transferring gly	22.887557	2.023889657	0.65890996	3.071572425	0.002129345	0.024310418
PITA_000050892	High quality	AT4G34670	MA_124901g0010	shade<sun	Ribosomal protein S3Ae;(source:Araport11)		cell wall-GO:0005618,cytoplasm-G	120.55163	-0.686134432	0.19828026	-3.46042739	0.000539319	0.007409266
PITA_000050967	High quality	AT2G25870	MA_10429896g0010	shade<sun	Encodes an endoribonuclease that is requ	YBEY ENDORIBONUCLEASE (ATYBE	chloroplast-GO:0009507,chloropla	228.09877	-1.017441926	0.26094097	-3.899126726	9.65E-05	0.001611605
PITA_000051095	High quality	AT3G28917	MA_2285g0010	shade>sun	mini zinc finger 2;(source:Araport11)	MINI ZINC FINGER 2 (MIF2)	cytoplasm-GO:0005737,DNA bindi	35.053687	1.581467684	0.52858591	2.991883913	0.002772617	0.030472605
PITA_000051338	High quality	AT1G13570	MA_140786g0010	shade<sun	F-box/RNI-like superfamily protein;(source:Araport11)		biological_process_unknown-GO:0	34.607161	-21.87264303	4.40880414	-4.961128308	7.01E-07	2.04E-05
PITA_000051369	High quality	AT3G51240	MA_10435304g0010	shade<sun	Encodes flavanone 3-hydroxylase that is c	FLAVANONE 3-HYDROXYLASE (F3H	cytoplasm-GO:0005737,dioxygena	411.10075	-2.597888807	0.45962725	-5.652164429	1.58E-08	1.03E-06
PITA_000051456	Low quality	AT5G60600	MA_88006g0010	shade<sun	Encodes a chloroplast-localized hydroxy-	4-HYDROXY-3-METHYLBUT-2-ENYL	4 iron, 4 sulfur cluster binding-GO:	229.49601	-1.577683328	0.26017277	-6.063983191	1.33E-09	1.18E-07
PITA_000051460	High quality	AT4G08685	MA_285833g0010	shade<sun	Encodes a protein, expressed in leaves, w	(SAH7)	biological_process_unknown-GO:0	35.358761	-21.90137695	4.40877489	-4.967678662	6.78E-07	2.02E-05
PITA_000051588	Low quality	AT4G33110	MA_270209g0010	shade<sun	S-adenosyl-L-methionine-dependent methyltransferases superfamily protein;(		cytoplasm-GO:0005737,plasma me	193.63999	-0.957956859	0.28903813	-3.314292306	0.000918754	0.011763727
PITA_000051602	High quality	AT1G30000	MA_10426596g0010	shade>sun	alpha-mannosidase 3;(source:Araport11)	ALPHA-MANNOSIDASE 3 (MNS3)	alpha-mannosidase activity-GO:000	81.016852	23.32680868	4.3285545	5.389052785	7.08E-08	3.53E-06
PITA_000051806	High quality	AT1G04920	MA_103595g0010	shade<sun	Encodes a protein with putative sucrose-p	SUCROSE PHOSPHATE SYNTHASE 3	Golgi apparatus-GO:0005794,sucro	103.3694	-0.927684655	0.2521378	-3.67927643	0.000233897	0.003571631
PITA_000051920	Low quality	AT5G05440	MA_554564g0010	shade<sun	Encodes a member of the PYR (pyrabactir	PYRABACTIN RESISTANCE 1-LIKE 5	abscisic acid binding-GO:0010427,a	341.63271	-11.66590812	1.78362403	-6.540564544	6.13E-11	7.44E-09
PITA_000052208	Low quality	AT1G70300	MA_103581g0010	shade>sun	potassium transporter	K+ UPTAKE PERMEASE 6 (KUP6)	membrane-GO:0016020,mitochon	36.54903	8.935549356	2.57712906	3.467249464	0.000525814	0.007243161
PITA_000052243	Low quality	AT5G57625	MA_79998g0010	shade>sun	CAP (Cysteine-rich secretory proteins, Ant	(ATCAPE5)	biological_process_unknown-GO:0	24.935786	1.672100915	0.5492182	3.044511135	0.002330588	0.026326289
PITA_000052296	High quality	AT1G22640	MA_130918g0010	shade<sun	MYB-type transcription factor (MYB3) tha	MYB DOMAIN PROTEIN 3 (MYB3)	response to wounding-GO:000961	1157.3372	-1.595060625	0.28582735	-5.580503795	2.40E-08	1.45E-06
PITA_000052328	High quality	AT1G26880	MA_109553g0010	shade>sun	Ribosomal protein L34e superfamily protein;(source:Araport11)		cytoplasm-GO:0005737,cytosolic la	110.63685	2.265739876	0.45213605	5.011190516	5.41E-07	1.71E-05
PITA_000052348	High quality	AT2G44020	MA_78196g0010	shade<sun	Mitochondrial transcription termination factor family protein;(source:Araport		developmental process-GO:00325C	358.79045	-0.679373892	0.17226416	-3.943791234	8.02E-05	0.001358367
PITA_000052444	High quality	AT3G14940	MA_20154g0020	shade<sun	Encodes a cytosolic phosphoenolpyruvate	PHOSPHOENOLPYRUVATE CARBOX	apoplast-GO:0048046,chloroplast-	203.68858	-0.488480305	0.16555558	-2.950551796	0.003172069	0.034044565
PITA_000052495	High quality	AT3G61440	MA_6291g0010	shade<sun	Encodes a cysteine synthase isomer CysC	CYSTEINE SYNTHASE C1 (CYSC1)	chloroplast-GO:0009507,copper ior	60.714318	-22.53151824	4.4082046	-5.11126871	3.20E-07	1.18E-05
PITA_000052560	High quality	AT1G80170	MA_127755g0010	shade<sun	Pectin lyase-like superfamily protein;(source:Araport11)		carbohydrate metabolic process-G(	273.72786	-0.885653043	0.15711523	-5.636964995	1.73E-08	1.11E-06
PITA_000052621	Low quality	AT2G38500	MA_24218g0010	shade>sun	2-oxoglutarate (2OG) and Fe(II)-dependent oxygenase superfamily protein;(sc		biological_process_unknown-GO:0	7.9727723	3.036981702	0.92171316	3.294931468	0.000984457	0.012492892
PITA_000052658	High quality	AT4G36530	MA_196209g0010	shade<sun	alpha/beta-Hydrolases superfamily protein;(source:Araport11)		chloroplast-GO:0009507,chloropla	647.7942	-1.331421507	0.18886746	-7.049501923	1.80E-12	3.09E-10
PITA_000052698	High quality	AT4G02750	MA_55648g0010	shade<sun	Tetratricopeptide repeat (TPR)-like superf	(SSTPR)	acquisition of seed longevity-GO:01	101.56095	-3.039273183	0.69843849	-4.351525933	1.35E-05	0.000260892
PITA_000052706	Low quality	AT2G48070	MA_122925g0010	shade<sun	Encodes a chloroplast protein RPH1 (resis	RESISTANCE TO PHYTOPHTHORA 1	chloroplast-GO:0009507,defense r	281.44481	-0.514698605	0.14884775	-3.457886278	0.000544431	0.007467484
PITA_000052749	Low quality	AT5G15800	MA_5917g0010	shade<sun	Encodes a MADS box transcription factor	SEPALLATA1 (SEP1)	DNA-binding transcription factor ac	1244.7449	-13.53089764	2.59420697	-5.215812697	1.83E-07	8.32E-06
PITA_000052948	High quality	AT1G52240	MA_10199247g0010	shade<sun	dynein light chain flagellar outer arm-like		DNA binding-GO:0003677,DNA-binding transcription factor activity-GO:0003700,response to UV-B-GO:0010224,regulation of phenylpropanoid metabolic process-GO:2000762,negative regulation of transcription, DNA-templated-GO:0045892,response to UV-B-GO:0010224,transcription cis-regulatory region binding-GO:0000976,negative regulation of sinapate ester biosynthetic process-GO:1903086,nucleus-GO:0005634 DNA binding-GO:0003677,protein binding-GO:0005515	174.71258	-0.489510696	0.1644893	-2.975942492	0.002920896	0.031815531
PITA_000052976	Low quality	AT4G01150	MA_60075g0010	shade<sun	Integral thylakoid membrane protein requ	CURVATURE THYLAKOID 1A (CURT	chloroplast-GO:0009507,chloropla	350.34255	-1.044589264	0.29990071	-3.483117	0.000495612	0.006875436
PITA_000053085	High quality	AT4G05420	MA_41156g0010	shade<sun	Structurally similar to damaged DNA bind	DAMAGED DNA BINDING PROTEIN	Cul4-RING E3 ubiquitin ligase comp	60.714318	-22.53151824	4.4082046	-5.11126871	3.20E-07	1.18E-05
PITA_000053150	High quality	AT3G51280	MA_10429050g0010	shade<sun	Tetratricopeptide repeat (TPR)-like superfamily protein;(source:Araport11)		RNA processing-GO:0006396,negative regulation of cellular process-GO:0048523	30.408535	-21.68945139	4.40899702	-4.919361767	8.68E-07	2.39E-05
PITA_000053170	High quality	AT5G09850	MA_23914g0010	shade>sun	Transcription elongation factor (TFIIS) fan	MEDIATOR 26C (MED26C)	cytosol-GO:0005829,nucleus-GO:0	31.591738	0.968298832	0.33954094	2.851788165	0.004347406	0.043926406
PITA_000053252	High quality	AT4G11370	MA_10433991g0020	shade<sun	Encodes a putative RING-H2 finger protei	RING-H2 FINGER A1A (RHA1A)	protein ubiquitination-GO:0016567	348.77509	-1.795563866	0.53753708	-3.340353518	0.000836718	0.01084346
PITA_000053351	High quality	AT3G21420	MA_10427811g0010	shade>sun	LATERAL BRANCHING OXIDOREDUCTASE	LATERAL BRANCHING OXIDOREDU	cytoplasm-GO:0005737,dioxygena	9.0646116	3.522222951	1.18782672	2.965266632	0.003024207	0.032759683
PITA_000053360	High quality	AT1G58370	MA_476611g0010	shade<sun	Encodes a protein with xylanase activity.	(RXF12)	cell wall-GO:0005618,endo-1,4-bet	351.27344	-1.134305349	0.2111401	-5.37228753	7.77E-08	3.82E-06
PITA_000053411	High quality	AT5G05350	MA_10428976g0010	shade>sun	PLAC8 family protein;(source:Araport11)		cyclase activity-GO:0009975,nucleu	120.4951	1.335461746	0.36707545	3.63811244	0.000274644	0.004122551
PITA_000053531	High quality	AT2G39890	MA_10433063g0010	shade<sun	Encodes a proline transporter with affinity	PROLINE TRANSPORTER 1 (PROT1)	amino acid transmembrane transp	361.12341	-11.74572165	2.10414104	-5.582193132	2.38E-08	1.44E-06
PITA_000053541	High quality	AT3G14740	MA_37406g0010	shade<sun	RING/FYVE/PHD zinc finger superfamily protein;(source:Araport11)		nucleus-GO:0005634	165.79441	-2.314558389	0.51141323	-4.525808614	6.02E-06	0.000122863
PITA_000053639	High quality	AT4G26190	MA_74105g0010	shade<sun	Haloacid dehalogenase-like hydrolase (HAD) superfamily protein;(source:Arap		biological_process_unknown-GO:0	146.68263	-0.809305468	0.28040742	-2.886177049	0.003899527	0.040039733
PITA_000053679	High quality	AT3G54190	MA_158067g0010	shade<sun	Transducin/WD40 repeat-like superfamily protein;(source:Araport11)		biological_process_unknown-GO:0	42.923142	-8.674732828	1.81516638	-4.779029034	1.76E-06	4.09E-05
PITA_000053682	High quality	AT1G75290	MA_176417g0010	shade<sun	encodes a protein whose sequence is similar to an isoflavone reductase		chloroplast-GO:0009507,cytoplasm	954.09435	-3.582639032	0.32787369	-10.92688798	8.57E-28	1.69E-24
PITA_000053758	Low quality	AT3G57450	MA_19912g0010	shade<sun	hypothetical protein;(source:Araport11)		biological_process_unknown-GO:0	39.412664	-22.04498447	4.40863388	-5.000411702	5.72E-07	1.79E-05
PITA_000053829	High quality	AT4G23160	MA_145923g0010	shade>sun	Encodes a cysteine-rich receptor-like prot	CYSTEINE-RICH RLK (RECEPTOR-LIK	ATP binding-GO:0005524,defense r	25.087729	8.392221649	2.03472048	4.124508366	3.72E-05	0.000670005
PITA_000053952	High quality	AT1G01280	MA_54958g0010	shade>sun	member of CYP703A CYP703A2 is expres	CYTOCHROME P450, FAMILY 703,	heme binding-GO:0020037,iron ior	25.830249	1.778203474	0.42731123	4.16137782	3.16E-05	0.000579032
PITA_000053972	High quality	AT1G11290	MA_100606g0010	shade<sun	Pentatricopeptide Repeat Protein contain	CHLORORESPIRATORY REDUCTION	chloroplast-GO:0009507,endonucle	68.326543	-9.34484505	1.80672526	-5.172255704	2.31E-07	9.61E-06
PITA_000054022	High quality	AT2G36870	MA_306910g0010	shade<sun	Encodes a xyloglucan endotransglycosylas	XYLOGLUCAN ENDOTRANSGLUCOS	carbohydrate metabolic process-G(	47.145015	-22.29213208	4.40843355	-5.056701391	4.27E-07	1.38E-05
PITA_000054044	High quality	AT1G22640	MA_116862g0010	shade>sun	MYB-type transcription factor (MYB3) tha	MYB DOMAIN PROTEIN 3 (MYB3)	response to wounding-GO:000961	951.11293	2.64501894	0.73792798	3.584386309	0.000337872	0.004924427
PITA_000054423	High quality	AT5G21482	MA_173695g0010	shade>sun	This gene used to be called AtCKX5. It enc	CYTOKININ OXIDASE 7 (CKX7)	cytokinin catabolic process-GO:000	135.02809	24.05501755	4.32832795	5.557577383	2.74E-08	1.63E-06
PITA_000054462	Low quality	AT4G30780	MA_126742g0010	shade<sun	ATP-dependent DNA helicase;(source:Araport11)		biological_process_unknown-GO:0	22.86906	-21.3124522	4.40951969	-4.833282011	1.34E-06	3.31E-05
PITA_000054468	High quality	AT5G25800	MA_116229g0010	shade<sun	Polynucleotidyl transferase, ribonuclease H-like superfamily protein;(source:A		exonuclease activity-GO:0004527,r	26.275109	-21.49994244	4.40924566	-4.876104457	1.08E-06	2.80E-05
PITA_000054482	High quality	AT2G36320	MA_10426546g0020	shade>sun	A20/AN1-like zinc finger family protein;(source:Araport11)		biological_process_unknown-GO:0	36.021494	0.990244541	0.32044033	3.090261901	0.001999801	0.023078367
PITA_000054484	Low quality	AT2G46690	MA_40440g0010	shade<sun	SAUR-like auxin-responsive protein family	SMALL AUXIN UPREGULATED RNA	molecular_function_unknown-GO:	35.358761	-21.90137695	4.40877489	-4.967678662	6.78E-07	2.02E-05
PITA_000054543	High quality	AT3G21420	MA_10431216g0020	shade<sun	LATERAL BRANCHING OXIDOREDUCTASE	LATERAL BRANCHING OXIDOREDU	cytoplasm-GO:0005737,dioxygena	13.403871	-6.996242494	1.53131814	-4.568771373	4.91E-06	0.000101561
PITA_000054576	High quality	AT1G48130	MA_92424g0010	shade<sun	encodes a protein similar to the 1-cystein	1-CYSTEINE PEROXIREDOXIN 1 (PEI	cell redox homeostasis-GO:004545	453.8594	-0.67200607	0.2313976	-2.904118631	0.003682886	0.038369138
PITA_000054602	High quality	AT5G40380	MA_27101g0010	shade<sun	Encodes a cysteine-rich receptor-like prot	CYSTEINE-RICH RLK (RECEPTOR-LIK	chloroplast-GO:0009507,kinase act	70.717523	-22.84103454	4.40809223	-5.181614484	2.20E-07	9.17E-06
PITA_000054611	High quality	AT4G29910	MA_10436793g0010	shade<sun	Origin Recognition Complex subunit 5. Inv	ORIGIN RECOGNITION COMPLEX P	DNA replication-GO:0006260,DNA	152.65414	-1.54766845	0.48491639	-3.19161915	0.001414777	0.017146058
PITA_000054642	High quality	AT2G33150	MA_161036g0010	shade>sun	Encodes an organellar (peroxisome, glyox	PEROXISOMAL 3-KETOACYL-COA T	acetyl-CoA C-acyltransferase activit	74.18114	9.956731936	1.93052368	5.157529031	2.50E-07	1.03E-05
PITA_000054850	High quality	AT1G72210	MA_686524g0010	shade>sun	basic helix-loop-helix (bHLH) DNA-binding	(BHLH096)	DNA-binding transcription factor ac	126.97208	0.934517184	0.21181039	4.41204594	1.02E-05	0.00020202
PITA_000054961	High quality	AT5G16080	MA_893141g0010	shade<sun	carboxyesterase 17;(source:Araport11)	CARBOXYESTERASE 17 (CXE17)	cellular_component_unknown-GO:	129.81746	-1.071426495	0.30969406	-3.459628764	0.000540921	0.007427289
PITA_000055015	High quality	AT2G31510	MA_170316g0010	shade<sun	IBR domain-containing protein;(source:Ar	ARIADNE 7 (ARI7)	cytoplasm-GO:0005737,positive re	60.714318	-22.53151824	4.4082046	-5.11126871	3.20E-07	1.18E-05
PITA_000055104	High quality	AT4G30470	MA_10265574g0010	shade>sun	NAD(P)-binding Rossmann-fold superfamily protein;(source:Araport11)		nucleus-GO:0005634,oxidoreducta	136.08947	1.356892841	0.38176223	3.554287813	0.000379004	0.005456988
PITA_000055198	Low quality	AT1G08315	MA_196504g0010	shade>sun	ARM repeat superfamily protein;(source:Araport11)		cytoplasm-GO:0005737,nucleus-G(	31.302539	1.008197397	0.34682085	2.906968815	0.003649496	0.038083247
PITA_000055201	High quality	AT4G01070	MA_10433602g0010	shade<sun	the glycosyltransferase (UGT72B1) is invo	(GT72B1)	chloroplast-GO:0009507,hydroquir	683.26543	-12.66578758	1.78034657	-7.114225867	1.13E-12	2.07E-10
PITA_000055228	Low quality	AT3G23240	MA_442451g0010	shade<sun	encodes a member of the ERF (ethylene r	ETHYLENE RESPONSE FACTOR 1 (EI	defense response-GO:0006952,DN/	35.358761	-21.90137695	4.40877489	-4.967678662	6.78E-07	2.02E-05
PITA_000055323	High quality	AT4G31550	MA_107950g0010	shade>sun	member of WRKY Transcription Factor; G	WRKY DNA-BINDING PROTEIN 11 ('	calmodulin binding-GO:0005516,de	50.683834	0.975140292	0.27396017	3.55942362	0.00037167	0.005365459
PITA_000055397	High quality	AT1G32230	MA_577g0010	shade>sun	Encodes a protein belonging to the (ADP-	RADICAL-INDUCED CELL DEATH1 (F	cytoplasm-GO:0005737,defense re	13.302517	1.595709195	0.54105274	2.94926738	0.003185283	0.034157755
PITA_000055405	High quality	AT3G19330	MA_115167g0010	shade<sun	transmembrane protein, putative (DUF677);(source:Araport11)		mitochondrion-GO:0005739,nucleu	52.550218	-22.4390271	4.4083279	-5.09014474	3.58E-07	1.19E-05
PITA_000055433	High quality	AT2G41250	MA_962052g0010	shade>sun	Haloacid dehalogenase-like hydrolase (HAD) superfamily protein;(source:Arap		oxoacid metabolic process-GO:004	86.865189	1.211540248	0.3408342	3.554632302	0.000378508	0.005454605
PITA_000055435	High quality	AT1G07890	MA_79741g0010	shade<sun	Encodes a cytosolic ascorbate peroxidase	ASCORBATE PEROXIDASE 1 (APX1)	cell wall-GO:0005618,cellular respo	674.75711	-1.02853824	0.25551108	-4.025415438	5.69E-05	0.000995523
PITA_000055582	High quality	AT1G69550	MA_10141g0040	shade<sun	disease resistance protein (TIR-NBS-LRR class);(source:Araport11)		ADP binding-GO:0043531,cellular_	70.717523	-22.84103454	4.40809223	-5.181614484	2.20E-07	9.17E-06
PITA_000055602	Low quality	AT1G20560	MA_10436294g0010	shade>sun	acyl activating enzyme 1;(source:Araport1	ACYL ACTIVATING ENZYME 1 (AAE	AMP binding-GO:0016208,chloropl	57.413875	1.51529649	0.38357263	3.950481249	7.80E-05	0.001323576
PITA_000055660	High quality	AT4G36920	MA_130948g0020	shade>sun	Encodes a floral homeotic gene, a membe	APETALA 2 (AP2)	cell differentiation-GO:0030154,DN	106.75363	0.657063869	0.20424141	3.217094266	0.00129496	0.015851504
PITA_000055676	High quality	AT1G22400	MA_10143270g0010	shade<sun	UDP-Glycosyltransferase superfamily prot	(UGT85A1)	cis-zeatin O-beta-D-glucosyltransfe	70.717523	-22.84103454	4.40809223	-5.181614484	2.20E-07	9.17E-06
PITA_000055688	High quality	AT2G30695	MA_62309g0010	shade<sun	bacterial trigger factor;(source:Araport11)		chaperone-mediated protein foldin	976.50244	-0.789897347	0.16904525	-4.672697772	2.97E-06	6.36E-05
PITA_000055798	High quality	AT2G15220	MA_10430419g0020	shade<sun	Plant basic secretory protein (BSP) family protein;(source:Araport11)		extracellular region-GO:0005576	5.148419	-5.603991955	1.97414056	-2.838699564	0.004529778	0.045471802
PITA_000055888	Low quality	AT5G20810	MA_40236g0010	shade>sun	SAUR-like auxin-responsive protein family	SMALL AUXIN UPREGULATED RNA	nucleus-GO:0005634,peroxisome-(	44.00488	1.87319261	0.54311838	3.448958207	0.000562754	0.007702298
PITA_000055983	Low quality	AT2G38770	MA_127081g0010	shade<sun	P-loop containing nucleoside triphosphate	EMBRYO DEFECTIVE 2765 (EMB27	catalytic step 2 spliceosome-GO:00	20.238106	-21.14561367	4.4097939	-4.795147836	1.63E-06	3.81E-05
PITA_000055990	High quality	AT2G16850	MA_17793g0010	shade>sun	plasma membrane intrinsic protein 2;(sou	PLASMA MEMBRANE INTRINSIC PF	cytoplasm-GO:0005737,plasma me	120.99119	1.787202238	0.61630947	2.899845512	0.003733466	0.038681911
PITA_000056002	High quality	AT1G48520	MA_889802g0010	shade<sun	Encodes Glu-tRNA(Gln) amidotransferase	GLU-ADT SUBUNIT B (GATB)	chloroplast-GO:0009507,chloropla	380.1757	-1.071114996	0.19634573	-5.455249757	4.89E-08	2.56E-06
PITA_000056045	High quality	AT4G03210	MA_120431g0010	shade<sun	Encodes a member of xyloglucan endotra	XYLOGLUCAN ENDOTRANSGLUCOS	cell wall biogenesis-GO:0042546,ex	17.516739	-20.95349401	4.41016289	-4.751183692	2.02E-06	4.57E-05
PITA_000056112	High quality	AT1G17650	MA_10048552g0010	shade<sun	Glyoxylate reductase located in chloropla	GLYOXYLATE REDUCTASE 2 (GLYR2	chloroplast-GO:0009507,chloropla	135.86193	-0.790363495	0.27224378	-2.903146228	0.003694341	0.038420619
PITA_000056115	Low quality	AT1G15140	MA_172152g0010	shade<sun	FAD/NAD(P)-binding oxidoreductase;(sou	FERREDOXIN-NADP(+) OXIDOREDU	chloroplast-GO:0009507,chloropla	206.95433	-0.470258757	0.16111019	-2.918864084	0.003513094	0.036930748
PITA_000056275	Low quality	AT5G42200	MA_289646g0010	shade<sun	RING/U-box superfamily protein;(source:	ARABIDOPSIS T??XICOS EN LEVADU	cellular response to hypoxia-GO:00	35.358761	-21.90137695	4.40877489	-4.967678662	6.78E-07	2.02E-05
PITA_000056326	High quality	AT2G38540	MA_7195626g0010	shade<sun	Non-specific lipid transfer protein. Binds c	LIPID TRANSFER PROTEIN 1 (LP1)	apoplast-GO:0048046,calmodulin t	449.22729	-2.829546191	0.72899792	-3.881418753	0.000103849	0.001719044
PITA_000056392	High quality	AT1G26260	MA_42080g0010	shade>sun	Encodes CIB5 (cryptochrome-interacting I	CRYPTOCHROME-INTERACTING BA	DNA-binding transcription factor ac	32.784215	1.35846649	0.37504864	3.622107489	0.000292213	0.004332704
PITA_000056403	Low quality	AT4G33160	MA_40909g0010	shade<sun	F-box family protein;(source:Araport11)		nucleus-GO:0005634,ubiquitin-dep	97.508695	-0.66257606	0.21134285	-3.135076797	0.001718091	0.02027506
PITA_000056416	High quality	AT1G01800	MA_10430538g0010	shade>sun	NAD(P)-binding Rossmann-fold superfamily protein;(source:Araport11)		chloroplast-GO:0009507,cytoplasm	56.314649	1.383410246	0.47418253	2.917463555	0.003528909	0.03706657
PITA_000056550	Low quality	AT2G28670	MA_16930g0010	shade<sun	Encodes a protein of unknown function th	ENHANCED SUBERIN 1 (ESB1)	Casparian strip-GO:0048226,lignin	35.358761	-21.90137695	4.40877489	-4.967678662	6.78E-07	2.02E-05
PITA_000056642	High quality	AT3G46940	MA_30958g0010	shade>sun	DUTP-PYROPHOSPHATASE-LIKE 1;(source	DUTP-PYROPHOSPHATASE-LIKE 1 (I	cytosol-GO:0005829,DNA repair-G	350.06133	12.19503541	1.68160678	7.252013701	4.11E-13	8.21E-11
PITA_000056756	Low quality	AT1G74160	MA_4736g0010	shade>sun	Member of a small gene family in Arabido	TON1 RECRUITING MOTIF 4 (TRM4	molecular_function_unknown-GO:	67.345428	9.816933141	1.47476265	6.656619037	2.80E-11	3.68E-09
PITA_000056904	High quality	AT4G17900	MA_8773709g0010	shade<sun	PLATZ transcription factor family protein;	(PLATZ11)	biological_process_unknown-GO:0	48.571454	-22.31053738	4.40840329	-5.060911151	4.17E-07	1.37E-05

PITA_000056952	Low quality	AT1G32300	MA_10434662g0010	shade>sun	D-arabinono-1,4-lactone oxidase family p	L -GULONO-1,4-LACTONE (L -GULL	D-arabinono-1,4-lactone oxidase ac	109.93747	0.997425245	0.29007533	3.438504202	0.000584937	0.007967595
PITA_000057017	Low quality	AT5G59970	MA_10427181g0010	shade>sun	Histone superfamily protein;(source:Araport11)		nucleus-GO:0005634,plastid-GO:0	108.31323	1.54752407	0.52411223	2.952657831	0.00315051	0.033856666
PITA_000057147	High quality	AT5G63450	MA_18375g0010	shade>sun	AtWRKY33 regulates root apoplastic barr	CYTOCHROME P450, FAMILY 94, SI	chloroplast-GO:0009507,heme bin	108.02247	23.75154617	4.32841291	5.487356833	4.08E-08	2.16E-06
PITA_000057185	High quality	AT3G01790	MA_109054g0010	shade>sun	Ribosomal protein L13 family protein;(source:Araport11)		chloroplast-GO:0009507,mitochon	428.37694	-0.734134387	0.22061373	-3.327691276	0.000875689	0.011274324
PITA_000057198	High quality	AT1G71080	MA_35723g0010	shade>sun	RNA polymerase II transcription elongation factor;(source:Araport11)		nucleus-GO:0005634,regulation of	270.05617	24.97459543	4.328158	5.770259648	7.91E-09	5.77E-07
PITA_000057220	High quality	AT1G22380	MA_22116g0010	shade>sun	Encodes a putative UDP-glucosyl transfera	UDP-GLUCOSYL TRANSFERASE 85A	glucuronosyltransferase activity-GC	180.8968	-1.669408603	0.54711299	-3.051304988	0.00227849	0.02581751
PITA_000057367	High quality	AT3G54050	MA_41114g0010	shade>sun	Encodes a chloroplastic fructose 1,6-bispl	HIGH CYCLIC ELECTRON FLOW 1 (H	apoplast-GO:0048046,chloroplast-(	960.5417	-0.820189646	0.24106868	-3.402306914	0.000668196	0.008982232
PITA_000057440	Low quality	AT4G22758	MA_268144g0010	shade>sun	PPR containing protein;(source:Araport11)		nucleus-GO:0005634	81.159226	-1.15043376	0.28403381	-4.050340884	5.11E-05	0.000905721
PITA_000057460	High quality	AT1G62390	MA_82470g0010	shade>sun	Encodes one of the 36 carboxylate clamp	PHOX2 (Phox2)	cytosol-GO:0005829,mRNA binding	38.079318	-8.500644622	1.84463118	-4.608316667	4.06E-06	8.49E-05
PITA_000057483	High quality	AT1G08470	MA_10429962g0010	shade>sun	Although this enzyme is predicted to enco	STRICTOSIDINE SYNTHASE-LIKE 3 (S	biosynthetic process-GO:0009058,(	177.74565	-0.663165858	0.2265195	-2.927632514	0.003415535	0.03605318
PITA_000057526	Low quality	AT1G68530	MA_10605g0020	shade>sun	Encodes KCS6, a member of the 3-ketoac	3-KETOACYL-COA SYNTHASE 6 (KCS	3-oxo-arachidoyl-CoA synthase act	302.54858	25.14714461	4.32813976	5.810150776	6.24E-09	4.69E-07
PITA_000057796	Low quality	AT3G23760	MA_114452g0010	shade>sun	transferring glycosyl group transferase;(source:Araport11)		biological_process_unknown-GO:00	231.41109	-1.153778618	0.22211513	-5.194507152	2.05E-07	9.17E-06
PITA_000057803	Low quality	AT3G56580	MA_76949g0010	shade>sun	Encodes a functional E3 ubiquitin ligase ir	RING ZINC FINGER 1 (RZF1)	negative regulation of proline biosy	26.275109	-21.49994244	4.40924566	-4.876104457	1.08E-06	2.80E-05
PITA_000057878	High quality	AT1G05675	MA_732668g0010	shade>sun	UDP-Glycosyltransferase superfamily protein;(source:Araport11)		quercetin 3-O-glucosyltransferase a	964.96519	-2.75826326	0.27811616	-9.917666359	3.49E-23	4.70E-20
PITA_000057915	Low quality	AT1G80820	MA_29397g0010	shade>sun	Encodes an cinnamoyl CoA reductase isof	CINNAMOYL COA REDUCTASE (CCF	catalytic activity-GO:0003824,cellu	26.275109	-21.49994244	4.40924566	-4.876104457	1.08E-06	2.80E-05
PITA_000057946	High quality	AT3G57520	MA_139754g0010	shade>sun	SIP2 encodes a raffinose-specific alpha-ga	SEED IMBIBITION 2 (SIP2)	cytoplasm-GO:0005737,galactinol-	3851.4398	-1.818149503	0.39825032	-4.56534345	4.99E-06	0.000103068
PITA_000057974	High quality	AT4G12650	MA_10428354g0010	shade>sun	Endomembrane protein 70 protein family	(TMN12)	endosome-GO:0005768,Golgi appa	65.436585	2.528442987	0.6962622	3.631452332	0.000281831	0.004205781
PITA_000058013	High quality	AT1G70520	MA_123360g0010	shade>sun	Encodes a cysteine-rich receptor-like protein kinase. Involved in regulating mi		kinase activity-GO:0016301,plasma	52.550218	-22.4390271	4.4083279	-5.09014474	3.58E-07	1.19E-05
PITA_000058080	High quality	AT3G28345	MA_929827g0010	shade>sun	Encodes an ATP-binding cassette (ABC) tr	ATP-BINDING CASSETTE B15 (ABCB	ATPase activity-GO:0016887,ATPas	110.21886	1.110426127	0.23819277	4.661880022	3.13E-06	6.69E-05
PITA_000058201	High quality	AT2G32440	MA_107159g0010	shade>sun	ent-kaurenoic acid hydroxylase (KAO2)	ENT-KAURENOIC ACID HYDROXYLA	brassinosteroid biosynthetic proces	197.55136	-10.87559616	1.86291019	-5.837960522	5.28E-09	4.06E-07
PITA_000058378	Low quality	AT5G43830	MA_12061g0010	shade>sun	aluminum induced protein with YGL and LRDR motifs;(source:Araport11)		chloroplast-GO:0009507,cytosol-G(	11.085276	7.213745813	2.3913908	3.016548285	0.002556705	0.028466131
PITA_000058387	High quality	AT1G77490	MA_119996g0010	shade>sun	Encodes a chloroplastic thylakoid ascorba	THYLAKOIDAL ASCORBATE PEROXI	cellular response to oxidative stres	140.84862	-0.724883228	0.24649586	-2.940752149	0.003274164	0.034920779
PITA_000058403	High quality	AT1G64970	MA_10432303g0020	shade>sun	gamma-tocopherol methyltransferase (g-	GAMMA-TOCOPHEROL METHYLTR	chloroplast-GO:0009507,methyltra	16.374395	-7.278855394	1.56898056	-4.639225978	3.50E-06	7.41E-05
PITA_000058436	High quality	AT3G49590	MA_183150g0020	shade>sun	Autophagy-related protein 13;(source:Ara	AUTOPHAGY-RELATED 13 (ATG13)	Atg1/ULK1 kinase complex-GO:199	44.358126	1.10344703	0.31528389	3.499852211	0.000465516	0.006543155
PITA_000058449	Low quality	AT2G14095	MA_10152409g0010	shade>sun	hypothetical protein;(source:Araport11)		biological_process_unknown-GO:00	75.775062	-1.363957983	0.28935869	-4.713727379	2.43E-06	5.28E-05
PITA_000058464	High quality	AT5G43860	MA_337265g0010	shade>sun	Encodes a chlorophyllase, the first enzym	CHLOROPHYLLASE 2 (CLH2)	chlorophyll catabolic process-GO:0	67.280586	2.995908137	0.78087599	3.836599117	0.00012475	0.002039182
PITA_000058504	High quality	AT1G09250	MA_218073g0010	shade>sun	basic helix-loop-helix (bHLH) DNA-binding	ATBS1 INTERACTING FACTOR 4 (AI	DNA-binding transcription factor ac	82.310359	0.736469136	0.26210276	2.809848841	0.004956477	0.048869514
PITA_000058518	High quality	AT1G48100	MA_101764g0010	shade>sun	Pectin lyase-like superfamily protein;(sour	POLYGALACTURONASE INVOLVED	carbohydrate metabolic process-G(	434.0981	-1.861529565	0.32861071	-5.664847529	1.47E-08	9.92E-07
PITA_000058547	Low quality	AT5G23530	MA_131537g0010	shade>sun	carboxyesterase 18;(source:Araport11)	CARBOXYESTERASE 18 (CXE18)	carboxylic ester hydrolase activity-(	40.973086	2.153484319	0.42537763	5.062523622	4.14E-07	1.36E-05
PITA_000058559	Low quality	AT5G19400	MA_10435713g0020	shade>sun	Encodes SMG7, a protein that possesses a	(SMG7)	meiotic cell cycle-GO:0051321,nucl	173.91352	-1.669792378	0.57197571	-2.919341393	0.003507718	0.036889385
PITA_000058630	High quality	AT5G44050	MA_829125g0010	shade>sun	MATE efflux family protein;(source:Arapo	(ATDTX28)	antiporter activity-GO:0015297,me	90.764575	22.54018959	4.32849343	5.207398364	1.92E-07	8.62E-06
PITA_000058678	High quality	AT3G48770	MA_10426213g0030	shade>sun	ATP/DNA binding protein;(source:Araport11)		biological_process_unknown-GO:00	26.107157	-21.49206233	4.40925805	-4.87404527	1.09E-06	2.81E-05
PITA_000058730	High quality	AT4G01050	MA_157164g0010	shade>sun	hydroxyproline-rich glycoprotein family p	THYLAKOID RHODANESE-LIKE (TRO	chloroplast-GO:0009507,chloropla	824.6468	-1.061514073	0.14934269	-7.107907802	1.18E-12	2.14E-10
PITA_000058755	High quality	AT2G38140	MA_202598g0010	shade>sun	plastid-specific ribosomal protein 4 (PSRP	PLASTID-SPECIFIC RIBOSOMAL PRC	chloroplast-GO:0009507,chloropla	263.07304	-0.618084397	0.14407428	-4.290039819	1.79E-05	0.000338611
PITA_000058780	High quality	AT5G19730	MA_20853g0010	shade>sun	Pectin lyase-like superfamily protein;(source:Araport11)		carboxylic ester hydrolase activity-(	685.56811	-0.553693598	0.1772951	-3.12300558	0.001790144	0.020980318
PITA_000058827	High quality	AT3G22990	MA_1130411g0010	shade>sun	Armadillo-repeat containing protein. Invo	LEAF AND FLOWER RELATED (LFR)	anther development-GO:0048653,/	549.99448	-12.35239519	4.37970015	-2.820374631	0.004796761	0.047571991
PITA_000058842	High quality	AT2G20830	MA_206805g0010	shade>sun	folic acid binding / transferase;(source:Araport11)		chloroplast-GO:0009507,folic acid l	363.17973	-1.089411638	0.25580561	-4.258748017	2.06E-05	0.000386234
PITA_000059054	High quality	AT5G45930	MA_48250g0010	shade>sun	encodes a second Chl I gene (CHLI2), a su	MAGNESIUM CHELATASE I2 (CHLI2	ATPase activity-GO:0016887,chlorc	30.357159	-21.69383454	4.40899933	-4.920353335	8.64E-07	2.39E-05
PITA_000059140	Low quality	AT1G06340	MA_172637g0030	shade>sun	Plant Tudor-like protein;(source:Araport11)		biological_process_unknown-GO:00	7.0959683	-6.082479388	1.64981344	-3.686767984	0.00022712	0.003484779
PITA_000059270	High quality	AT1G65730	MA_46073g0010	shade>sun	Arabidopsis thaliana metal-nicotianamine	YELLOW STRIPE LIKE 7 (YSL7)	membrane-GO:0016020,nucleus-G	455.13348	-4.106265239	1.10310836	-3.722449574	0.000197299	0.003073261
PITA_000059333	High quality	AT5G63960	MA_36703g0010	shade>sun	Encodes the catalytic subunits of DNA pol	GIGANTEA SUPPRESSOR5 (GIS5)	3'-5'-exodeoxyribonuclease activity	198.78704	11.37878847	1.29771607	8.768318972	1.81E-18	1.00E-15
PITA_000059389	High quality	AT1G56220	MA_15605g0010	shade>sun	Dormancy/auxin associated family protein;(source:Araport11)		molecular_function_unknown-GO:0	11.481933	4.749913308	1.01928863	4.660077772	3.16E-06	6.74E-05
PITA_000059481	High quality	AT4G10500	MA_186973g0010	shade>sun	2-oxoglutarate (2OG) and Fe(II)-depender	DMR6-LIKE OXYGENASE 1 (DLO1)	chloroplast-GO:0009507,defense r	28.287009	-21.59902252	4.40911624	-4.898719231	9.65E-07	2.61E-05
PITA_000059492	High quality	AT5G20610	MA_10431323g0010	shade>sun	Encodes a member of a plant specific C2 c	PLASTID MOVEMENT IMPAIRED1-F	actin filament-based movement-GC	17.516739	-20.95349401	4.41016289	-4.751183692	2.02E-06	4.57E-05
PITA_000059712	High quality	AT1G09740	MA_100052g0010	shade>sun	Adenine nucleotide alpha hydrolases-like superfamily protein;(source:Araport		cytoplasm-GO:0005737	13.495924	5.69837339	1.81252131	3.143893175	0.001667163	0.019811001
PITA_000059892	High quality	AT1G15000	MA_83401g0010	shade>sun	serine carboxypeptidase-like 50;(source:A	SERINE CARBOXYPEPTIDASE-LIKE 5	chloroplast-GO:0009507,serine-typ	352.73659	-1.425574114	0.48960602	-2.911676041	0.003594953	0.037682992
PITA_000059957	Low quality	AT5G19140	MA_762051g0010	shade>sun	aluminum induced protein with YGL and L	(AILP1)	chloroplast-GO:0009507,cytoplasm	27.368323	2.674929538	0.47309045	5.654160975	1.57E-08	1.02E-06
PITA_000059979	High quality	AT5G48380	MA_171402g0010	shade>sun	Encodes a BAK1-interacting receptor-like	BAK1-INTERACTING RECEPTOR-LIK	defense response to bacterium-GO	50.501646	2.807487407	0.91556644	3.066393961	0.002166576	0.024658528
PITA_000059996	Low quality	AT3G27160	MA_249305g0010	shade>sun	GHS1 encodes plastid ribosomal protein S	GLUCOSE HYPERSENSITIVE 1 (GHS1	chloroplast-GO:0009507,chloropla	475.47336	-0.764529106	0.18271246	-4.184329431	2.86E-05	0.000528055
PITA_000060012	Low quality	AT1G15550	MA_666770g0010	shade>sun	Involved in later steps of the gibberellic ac	GIBBERELLIN 3-OXIDASE 1 (GA3OX	cytoplasm-GO:0005737,dioxygenas	14.591253	1.698165941	0.58122659	2.921693491	0.00348134	0.036627011
PITA_000060035	Low quality	AT2G46600	MA_1552801g0010	shade>sun	Calcium-binding EF-hand family protein;(source:Araport11)		calcium ion binding-GO:0005509,n	27.060063	1.803872207	0.40861281	4.414624686	1.01E-05	0.000200089
PITA_000060057	Low quality	AT3G17390	MA_141759g0010	shade>sun	S-adenosylmethionine synthetase	METHIONINE OVER-ACCUMULATO	cell wall-GO:0005618,cytoplasm-G	73.592823	-9.448397202	1.48186851	-6.376002398	1.82E-10	2.01E-08
PITA_000060247	High quality	AT5G65700	MA_943683g0010	shade>sun	Encodes a CLAVATA1-related receptor kir	BARELY ANY MERISTEM 1 (BAM1)	anther development-GO:0048653,(	329.44286	-11.61326062	1.87237742	-6.202414384	5.56E-10	5.39E-08
PITA_000060533	High quality	AT2G01300	MA_118031g0010	shade>sun	mediator of RNA polymerase II transcription subunit;(source:Araport11)		biological_process_unknown-GO:00	132.04488	-10.29371254	2.00194202	-5.141863456	2.72E-07	1.11E-05
PITA_000060617	High quality	AT3G22960	MA_3663g0010	shade>sun	encodes a chloroplast pyruvate kinase alp	(PKP-ALPHA)	chloroplast-GO:0009507,chloropla	294.13777	-1.580267228	0.27554025	-5.735159363	9.74E-09	6.93E-07
PITA_000060782	High quality	AT1G55580	MA_10255g0010	shade>sun	Encodes a member of the GRAS family of	LATERAL SUPPRESSOR (LAS)	axillary shoot meristem initiation-G	162.0337	24.31540594	4.32827129	5.617810044	1.93E-08	1.19E-06
PITA_000060797	High quality	AT2G33470	MA_403012g0010	shade>sun	glycolipid transfer protein 1;(source:Arap	GLYCOLIPID TRANSFER PROTEIN 1	ceramide 1-phosphate binding-GO:	24.169213	1.559979438	0.45338882	3.440709999	0.00058019	0.007924003
PITA_000060911	High quality	AT3G10050	MA_10434827g0010	shade>sun	first enzyme in the biosynthetic pathway c	L-O-METHYLTHREONINE RESISTAN	chloroplast-GO:0009507,isoleucine	66.295654	-1.313629023	0.28386133	-4.62771398	3.70E-06	7.80E-05
PITA_000060923	High quality	AT4G01130	MA_9754208g0010	shade>sun	GDSL-motif esterase/acyltransferase/lipase. Enzyme group with broad substr		chloroplast-GO:0009507,cytosol-G(	828.16515	-1.347019813	0.33368626	-4.036785323	5.42E-05	0.000953065
PITA_000061026	Low quality	AT5G28150	MA_122006g0010	shade>sun	hypothetical protein (DUF868);(source:Araport11)		biological_process_unknown-GO:00	21.020087	-21.19302767	4.40970434	-4.805997415	1.54E-06	3.72E-05
PITA_000061058	High quality	AT4G28890	MA_179334g0010	shade>sun	RING/U-box superfamily protein;(source:/	ARABIDOPSIS T??XICOS EN LEVADU	nucleus-GO:0005634,protein ubiqu	37.917094	1.184961832	0.40742592	2.908410514	0.003632711	0.037954496
PITA_000061086	Low quality	AT1G49000	MA_92747g0010	shade>sun	transmembrane protein;(source:Araport11)		biological_process_unknown-GO:00	15.331838	-7.182671807	1.52436561	-4.711908838	2.45E-06	5.33E-05
PITA_000061115	High quality	AT1G05835	MA_7163462g0010	shade>sun	PhD finger protein.		cell fate determination-GO:000170	12.811922	2.362410268	0.68889252	3.429287134	0.000605169	0.008212595
PITA_000061123	High quality	AT1G71695	MA_57932g0010	shade>sun	Peroxidase superfamily protein;(source:Araport11)		cell wall-GO:0005618,extracellular	56.391786	1.67659765	0.48283004	3.472438539	0.000515753	0.00712758
PITA_000061127	Low quality	AT5G11590	MA_5530g0010	shade>sun	encodes a member of the DREB subfamily	TINY2 (TINY2)	DNA-binding transcription factor ac	28.15704	2.298991894	0.48596086	4.730816955	2.24E-06	4.89E-05
PITA_000061139	High quality	AT3G24460	MA_164476g0010	shade>sun	Serinc-domain containing serine and sphingolipid biosynthesis protein;(source		membrane-GO:0016020	81.016852	23.32680868	4.3285545	5.389052785	7.08E-08	3.53E-06
PITA_000061205	High quality	AT4G10955	MA_299373g0010	shade>sun	alpha/beta-Hydrolases superfamily protein;(source:Araport11)		nucleus-GO:0005634	46.93454	2.822365745	0.66751553	4.228164885	2.36E-05	0.000440071
PITA_000061331	High quality	AT4G02750	MA_101924g0010	shade>sun	Tetratricopeptide repeat (TPR)-like superf	(SSTPR)	acquisition of seed longevity-GO:01	114.52095	10.58300869	1.29828259	8.151544821	3.59E-16	1.26E-13
PITA_000061450	High quality	AT1G27620	MA_10392111g0010	shade>sun	HXXXD-type acyl-transferase family protein;(source:Araport11)		nucleus-GO:0005634,transferase a	34.418404	2.008457155	0.44990699	4.464160773	8.04E-06	0.000160817
PITA_000061461	Low quality	AT2G36145	MA_66902g0010	shade>sun	hypothetical protein;(source:Araport11)		biological_process_unknown-GO:00	236.33545	-1.555032811	0.53872859	-2.88648653	0.003895694	0.040030539
PITA_000061475	Low quality	AT4G08280	MA_10253568g0010	shade>sun	Thioredoxin superfamily protein;(source:Araport11)		chloroplast-GO:0009507,molecular	36.588781	-8.445258249	1.7040129	-4.956099949	7.19E-07	2.08E-05
PITA_000061510	High quality	AT4G30110	MA_138917g0010	shade>sun	encodes a protein similar to Zn-ATPase, a	HEAVY METAL ATPASE 2 (HMA2)	ABC-type cadmium transporter act	290.53153	-1.504150031	0.4905155	-3.066467907	0.002166041	0.024658528
PITA_000061530	High quality	AT5G59070	MA_1799g0010	shade>sun	UDP-Glycosyltransferase superfamily protein;(source:Araport11)		endosome-GO:0005768,Golgi appa	412.56133	-1.401575634	0.42638205	-3.287135679	0.001012121	0.012757169
PITA_000061585	High quality	AT2G39220	MA_83822g0010	shade>sun	Phospholipase pPLAIIIa involved in seed g	PATATIN-LIKE PROTEIN 6 (PLP6)	hydrolase activity-GO:0016787,lip	422.52721	-11.97257032	1.58712531	-7.543557063	4.57E-14	1.16E-11
PITA_000061628	High quality	AT5G02790	MA_37646g0010	shade>sun	Glutathione S-transferase family protein;(	GLUTATHIONE TRANSFERASE L3 (G	cytoplasm-GO:0005737,cytosol-GC	52.550218	-22.4390271	4.4083279	-5.09014474	3.58E-07	1.19E-05
PITA_000061725	High quality	AT2G15230	MA_138441g0010	shade>sun	Lipase active on medium and short chain	LIPASE 1 (LIP1)	extracellular region-GO:0005576,e:	90.967548	0.942024813	0.2523579	3.732892038	0.000189294	0.002962979
PITA_000061727	High quality	AT3G53980	MA_104866g0010	shade>sun	Bifunctional inhibitor/lipid-transfer protein/seed storage 2S albumin superfan		extracellular region-GO:0005576,fa	35.358761	-21.90137695	4.40877489	-4.967678662	6.78E-07	2.02E-05
PITA_000061745	High quality	AT2G42040	MA_169331g0010	shade>sun	WRC protein;(source:Araport11)		developmental process-GO:00325C	380.68663	-1.132587342	0.26039706	-4.349462826	1.36E-05	0.00026316
PITA_000061834	High quality	AT1G05675	MA_310012g0010	shade>sun	UDP-Glycosyltransferase superfamily protein;(source:Araport11)		quercetin 3-O-glucosyltransferase a	171.78143	11.16799284	1.28547222	8.687852377	3.69E-18	1.93E-15
PITA_000061894	High quality	AT5G07990	MA_9169998g0010	shade>sun	Required for flavonoid 3' hydroxylase acti	TRANSPARENT TESTA 7 (TT7)	extracellular region-GO:0005576,fl	134.67496	-10.32236549	1.55097585	-6.655400518	2.83E-11	3.69E-09
PITA_000061944	High quality	AT4G09810	MA_10597g0010	shade>sun	Nucleotide-sugar transporter family prote	UDP-RHA/UDP-GAL TRANSPORTER	antiporter activity-GO:0015297,glu	203.24289	-1.497101623	0.39442309	-3.795674404	0.000147243	0.002366974
PITA_000061968	High quality	AT5G53190	MA_18013g0010	shade>sun	Nodulin MtN3 family protein;(source:Ara	(SWEET3)	carbohydrate transport-GO:000864	35.358761	-21.90137695	4.40877489	-4.967678662	6.78E-07	2.02E-05
PITA_000061977	High quality	AT1G33390	MA_10428112g0010	shade>sun	Over-expression of this gene results in ste	FASCIATED STEM 4 (FAS4)	intracellular anatomical structure-G	46.714898	1.062639898	0.29030491	3.660426936	0.000251795	0.003813114
PITA_000062034	High quality	AT1G73220	MA_6288g0010	shade>sun	Encodes Organic Cation Transporter 1 (O(	ORGANIC CATION/CARNITINE TRAI	cadaverine transport-GO:0015839,(	73.892629	-1.088563404	0.35771806	-3.043076421	0.002341729	0.026417174
PITA_000062104	High quality	AT4G30610	MA_10277041g0010	shade>sun	Encodes a secreted glycosylated serine ca	BRI1 SUPPRESSOR 1 (BRS1)	brassinosteroid mediated signaling	677.40057	-12.65342285	1.55820911	-8.120490895	4.64E-16	1.59E-13
PITA_000062135	High quality	AT3G20570	MA_5355712g0010	shade>sun	early nodulin-like protein 9;(source:Arapc	EARLY NODULIN-LIKE PROTEIN 9 (E	anchored component of membrane	17.679381	-20.92872422	4.41014042	-4.745591347	2.08E-06	4.59E-05
PITA_000062213	High quality	AT3G05950	MA_160196g0010	shade>sun	RmlC-like cupins superfamily protein;(source:Araport11)		cell wall-GO:0005618,extracellular	121.01943	23.90838197	4.3283673	5.52364906	3.32E-08	1.87E-06
PITA_000062225	Low quality	AT1G68490	MA_8052g0010	shade>sun	translocase subunit seca;(source:Araport11)		biological_process_unknown-GO:00	36.543555	1.087475618	0.35202882	3.089166427	0.00200719	0.023142779
PITA_000062246	High quality	AT1G72390	MA_911375g0010	shade>sun	nuclear receptor coactivator;(source:Arap	PHYTOCHROME-DEPENDENT LATE	circadian rhythm-GO:0007623,cytc	52.550218	-22.4390271	4.4083279	-5.09014474	3.58E-07	1.19E-05
PITA_000062321	High quality	AT4G30110	MA_138917g0010	shade>sun	encodes a protein similar to Zn-ATPase, a	HEAVY METAL ATPASE 2 (HMA2)	ABC-type cadmium transporter act	55.109359	1.267276671	0.42121493	3.008622422	0.00262435	0.029280211
PITA_000062340	High quality	AT1G68020	MA_48114g0010	shade>sun	Encodes an enzyme putatively involved in	(ATTPS6)	alpha,alpha-trehalose-phosphate sy	33.606654	0.961743846	0.33876512	2.83896955	0.004525948	0.045451163
PITA_000062415	High quality	AT2G21050	MA_131574g0010	shade>sun	Encodes LAX2 (LIKE AUXIN RESISTANT), a	LIKE AUXIN RESISTANT 2 (LAX2)	amino acid transmembrane transpo	84.984071	1.091184953	0.34683256	3.146143326	0.001654389	0.019677472
PITA_000062453	High quality	AT5G38450	MA_35890g0010	shade>sun	cytochrome P450, family 735, subfamily /	CYTOCHROME P450, FAMILY 735,	heme binding-GO:0020037,iron ior	135.45543	-0.740112241	0.18095465	-4.09004258	4.31E-05	0.000770194
PITA_000062495	High quality	AT3G30530	MA_38931g0010	shade>sun	basic leucine-zipper 42;(source:Araport11	BASIC LEUCINE-ZIPPER 42 (bZIP42)	DNA-binding transcription factor ac	24.776516	2.091367756	0.71973294	2.905755241	0.003663679	0.03820012
PITA_000062679	Low quality	AT4G19100	MA_268175g0010	shade>sun	Protein of unknown function (DUF3464) ir	PHOTOSYNTHESIS AFFECTED MUT/	chloroplast-GO:0009507,molecular	293.66106	-0.820789599	0.19795159	-4.146415752	3.38E-05	0.000614229
PITA_000062778	High quality	AT5G65380	MA_170499g0010	shade>sun	MATE efflux family protein;(source:Araport11)		antiporter activity-GO:0015297,me	23.572508	-21.3530159	4.4094576	-4.84254933	1.28E-06	3.20E-05
PITA_000062784	High quality	AT1G26770	MA_40637g0020	shade>sun	Encodes an expansin. Naming convention	EXPANSIN A10 (EXPA10)	extracellular region-GO:0005576,p	448.52315	-1.212970045	0.29005794	-4.181819811	2.89E-05	0.000533534
PITA_000062909	High quality	AT4G24130	MA_5629g0010	shade>sun	DUF538 family protein (Protein of unknown function, DUF538);(source:Arapo		biological_process_unknown-GO:00	184.63571	-1.010685338	0.29379966	-3.440049398	0.000581608	0.007934906
PITA_000063015	High quality	AT4G32820	MA_10373626g0020	shade>sun	Tetratricopeptide repeat (TPR)-like superf	CALCINEURIN BINDING PROTEIN 1	DNA replication-independent nucle	20.238106	-21.14561367	4.4097939	-4.795147836	1.63E-06	3.81E-05
PITA_000063023	High quality	AT3G47620	MA_635g0010	shade>sun	Encodes a transcription factor AtTCP14 th	TEOSINTE BRANCHED, CYCLOIDEA	DNA-binding transcription factor ac	48.939351	0.915909309	0.28341714	3.231665193	0.001230712	0.015151954
PITA_000063135	High quality	AT1G27840	MA_721749g0010	shade>sun	Disease resistance protein (TIR-NBS-LRR class);(source:Araport11)		ADP binding-GO:0043531,chloropl:	114.68239	-10.09085367	1.94588896	-5.185729448	2.15E-07	9.17E-06
PITA_000063162	High quality	AT5G36930	MA_10426388g0010	shade>sun	Disease resistance protein (TIR-NBS-LRR class) family;(source:Araport11)		ADP binding-GO:0043531,chloropl:	52.550218	-22.4390271	4.4083279	-5.09014474	3.58E-07	1.19E-05
PITA_000063274	High quality	AT4G38250	MA_10433138g0010	shade>sun	Transmembrane amino acid transporter f	(AT4G38250)	amino acid transmembrane transpo	35.033479	-21.88927922	4.40878685	-4.964921176	6.87E-07	2.02E-05
PITA_000063289	High quality	AT3G54420	MA_10435326g0010	shade>sun	encodes an EP3 chitinase that is expresse	HOMOLOG OF CARROT EP3-3 CHIT	cell wall-GO:0005618,cell wall mac	359.22431	-5.860012052	0.82898837	-7.068871287	1.56E-12	2.74E-10
PITA_000063373	High quality	AT3G22142	MA_10199979g0010	shade>sun	Encodes a Protease inhibitor/seed storage/LTP family protein		cellular_component_unknown-GO:	517.45067	-12.26474146	1.85485226	-6.612247095	3.79E-11	4.80E-09
PITA_000063409	High quality	AT1G63000	MA_10426762g0010	shade>sun	nucleotide-rhamnose synthase/epimerase	NUCLEOTIDE-RHAMNOSE SYNTHAS	chloroplast-GO:0009507,cytosol-G	22.90419	8.261047707	1.58356958	5.216725441	1.82E-07	8.30E-06
PITA_000063794	High quality	AT5G07990	MA_126854g0010	shade>sun	Required for flavonoid 3' hydroxylase acti	TRANSPARENT TESTA 7 (TT7)	extracellular region-GO:0005576,fl	47.108077	9.301315407	2.16310778	4.299977793	1.71E-05	0.000324258
PITA_000063814	High quality	AT2G32780	MA_108353g0010	shade>sun	ubiquitin-specific protease 1;(source:Arap	UBIQUITIN-SPECIFIC PROTEASE 1 (l	cysteine-type endopeptidase activit	61.327353	-9.185039522	1.48384966	-6.190006845	6.02E-10	5.73E-08
PITA_000063837	High quality	AT3G47090	MA_168077g0010	shade>sun	Leucine-rich repeat protein kinase family protein;(source:Araport11)		chloroplast-GO:0009507,kinase act	363.0583	25.14604215	4.32811455	5.809929909	6.25E-09	4.69E-07
PITA_000063899	High quality	AT4G39850	MA_10435403g0010	shade>sun	Encodes a peroxisomal protein of the ATP	ATP-BINDING CASSETTE D1 (ABCD1	ATP binding-GO:0005524,ATPase a	379.32645	-1.140175275	0.12332821	-9.245048284	2.35E-20	1.88E-17
PITA_000064023	High quality	AT4G37850	MA_50811g0010	shade>sun	basic helix-loop-helix (bHLH) DNA-binding superfamily protein;(source:Arapor		DNA-binding transcription factor ac	1395.1227	-1.183505907	0.35050467	-3.376576691	0.000733939	0.009678019
PITA_000064096	Low quality	AT5G59500	MA_842948g0010	shade>sun	protein C-terminal S-isoprenylcysteine carboxyl O-methyltransferase;(source:		extracellular region-GO:0005576,p	392.74955	-1.340434512	0.31115543	-4.307925883	1.65E-05	0.000313989
PITA_000064129	High quality	AT2G23350	MA_10435577g0010	shade>sun	polyadenylate-binding protein, putative /	POLY(A) BINDING PROTEIN 4 (PAB4	cytosol-GO:0005829,mRNA 3'-UTR	44.755136	1.850496184	0.63784593	2.901164842	0.003717782	0.038607045
PITA_000064335	Low quality	AT1G79940	MA_1249g0010	shade>sun	J domain protein localized in ER membran	(ATERDJ2A)	chloroplast-GO:0009507,endoplasr	117.77019	10.62334258	2.04081189	5.205449186	1.94E-07	8.68E-06
PITA_000064358	High quality	AT2G20780	MA_41476g0010	shade>sun	Major facilitator superfamily protein;(source:Araport11)		carbohydrate transmembrane transp	114.88239	1.445526406	0.30713481	4.706488375	2.52E-06	5.46E-05
PITA_000064379	High quality	AT1G01490	MA_553359g0010	shade>sun	Heavy metal transport/detoxification supe	HEAVY METAL PROTEIN 1 (ATHMP(	chloroplast-GO:0009507,metal ion	19.793915	-21.11518951	4.40984638	-4.788191625	1.68E-06	3.92E-05
PITA_000064414	High quality	AT1G50320	MA_10433314g0010	shade>sun	encodes a prokaryotic thioredoxin	THIOREDOXIN X (THX)	cell redox homeostasis-GO:004545	181.74284	-0.535366407	0.18010517	-2.972521065	0.002953649	0.032090392
PITA_000064425	High quality	AT1G72510	MA_94444g0010	shade>sun	DUF1677 family protein (DUF1677);(source:Araport11)		biological_process_unknown-GO:00	79.551724	1.333808507	0.45008635	2.963450269	0.003042112	0.032911875
PITA_000064466	High quality	AT1G01250	MA_115502g0010	shade>sun	encodes a member of the DREB subfamily	A-4 of ERF/AP2 transcription factor	DNA-binding transcription factor ac	1568.3997	-1.178185495	0.24195489	-4.869442695	1.12E-06	2.87E-05
PITA_000064516	High quality	AT1G58340	MA_170275g0010	shade>sun	Encodes a plant MATE (multidrug and tox	(ZF14)	antiporter activity-GO:0015297,chl	186.70343	-10.79353798	1.60352077	-6.731149446	1.68E-11	2.34E-09
PITA_000064584	High quality	AT3G01100	MA_317011g0010	shade>sun	unknown protein, has cDNAs and ESTs as	HYPOTHETICAL PROTEIN 1 (HYP1)	calcium activated cation channel ac	81.698873	1.168376402	0.32305057	3.616698115	0.000298385	0.004404059
PITA_000064636	Low quality	AT2G41475	MA_217472g0010	shade>sun	Embryo-specific protein 3, (ATS3);(source	EMBRYO-SPECIFIC PROTEIN 3A (AT	biological_process_unknown-GO:00	65.687422	-1.395538055	0.28066155	-4.972316514	6.62E-07	2.02E-05
PITA_000064692	High quality	AT1G73500	MA_10194g0020	shade>sun	member of MAP Kinase Kinase family. Au	MAP KINASE KINASE 9 (MKK9)	camalexin biosynthetic process-GO	39.469551	1.28684211	0.4277122	3.008663539	0.002623995	0.029280211
PITA_000064696	Low quality	AT1G19530	MA_79150g0010	shade>sun	DNA polymerase epsilon catalytic subunit A;(source:Araport11)		anaerobic respiration-GO:0009061	15.58568	1.946607287	0.56097618	3.470035567	0.000520389	0.007180029
PITA_000064698	Low quality	AT3G15110	MA_837813g0010	shade>sun	transmembrane protein;(source:Araport11)		biological_process_unknown-GO:00	211.0568	-0.68251097	0.19293078	-3.537594998	0.000403789	0.0057896
PITA_000064728	High quality	AT5G03260	MA_33057g0010	shade>sun	LAC11 is a putative laccase, a member of	LACCASE 11 (LAC11)	copper ion binding-GO:0005507,ex	1309.6277	-2.111786235	0.74732131	-2.825807583	0.004716158	0.047011049
PITA_000064744	Low quality	AT3G52610	MA_230067g0010	shade>sun	GATA zinc finger protein;(source:Araport11)		biological_process_unknown-GO:00	87.515334	10.19494322	1.770238	5.759080541	8.46E-09	6.12E-07
PITA_000064754	High quality	AT1G11050	MA_54558g0020	shade>sun	Protein kinase superfamily protein;(source:Araport11)		chloroplast-GO:0009507,kinase act	56.574018	-22.53390044	4.40826287	-5.111741537	3.19E-07	1.18E-05
PITA_000064889	Low quality	AT5G41330	MA_161562g0010	shade>sun	BTB/POZ domain with WD40/YVTN repeat-like protein;(source:Araport11)		nucleus-GO:0005634,protein homc	32.46252	8.764078662	1.58195671	5.540024334	3.02E-08	1.78E-06
PITA_000064895	Low quality	AT1G48030	MA_4137775g0010	shade>sun	Encodes a mitochondrial lipoamide dehyc	MITOCHONDRIAL LIPOAMIDE DEH	apoplast-GO:0048046,ATP binding-	164.84434	-0.709399702	0.18724679	-3.788581412	0.00015151	0.002432518
PITA_000065026	High quality	AT5G47940	MA_111138g0010	shade>sun	40S ribosomal protein S27;(source:Araport11)		biological_process_unknown-GO:00	121.01943	23.90838197	4.3283673	5.52364906	3.32E-08	1.87E-06
PITA_000065032	Low quality	AT5G07330	MA_10337581g0010	shade>sun	NFU1 iron-sulfur cluster protein;(source:Araport11)		biological_process_unknown-GO:00	35.10338	1.58200219	0.36102378	4.38198894	1.18E-05	0.000229013
PITA_000065215	High quality	AT4G32410	MA_102060g0010	shade>sun	Encodes a cellulose synthase isomer. CES/	CELLULOSE SYNTHASE 1 (CESA1)	cellulose biosynthetic process-GO:C	20.238106	-21.14561367	4.4097939	-4.795147836	1.63E-06	3.81E-05
PITA_000065339	High quality	AT3G20330	MA_1829g0010	shade>sun	encodes aspartate carbamoyltransferase	PYRIMIDINE B (PYRB)	amino acid binding-GO:0016597,as	240.08175	-0.685257544	0.17108441	-4.005376881	6.19E-05	0.001074944
PITA_000065352	High quality	AT2G38820	MA_132127g0010	shade>sun	DNA-directed RNA polymerase subunit beta-beta protein, putative (DUF506);		biological_process_unknown-GO:00	21.109658	2.157560013	0.57190229	3.772602503	0.000161554	0.002566417
PITA_000065353	High quality	AT4G22970	MA_10428193g0010	shade>sun	hypothetical protein (DUF506);(source:Araport11)		biological_process_unknown-GO:00	20.974068	2.123850605	0.52856632	4.018134588	5.87E-05	0.001023983
PITA_000065550	High quality	AT2G30250	MA_14480g0020	shade>sun	Encodes a transcription factor WRKY6. R	(WRKY6)	cellular response to boron-containi	26.275109	-21.49994244	4.40924566	-4.876104457	1.08E-06	2.80E-05
PITA_000065705	High quality	AT3G20820	MA_175577g0010	shade>sun	Leucine-rich repeat (LRR) family protein;(source:Araport11)		apoplast-GO:0048046,cell wall-GO	94.51966	22.95135002	4.32847329	5.302412306	1.14E-07	5.46E-06
PITA_000065708	High quality	AT4G14130	MA_342978g0010	shade>sun	xyloglucan endotransglycosylase-related p	XYLOGLUCAN ENDOTRANSGLUCOS	cell wall biogenesis-GO:0042546,ex	326.82357	-1.632281031	0.41625007	-3.921395171	8.80E-05	0.001478683
PITA_000065711	High quality	AT2G45560	MA_10427075g0010	shade>sun	cytochrome P450 monooxygenase	CYTOCHROME P450, FAMILY 76, SI	chloroplast-GO:0009507,heme bin	2686.0698	-2.794362673	0.68346045	-4.088550636	4.34E-05	0.000774117
PITA_000065863	Low quality	AT5G49480	MA_10204459g0010	shade>sun	AtCP1 encodes a novel Ca2+-binding prot	CA2+-BINDING PROTEIN 1 (CP1)	calcium ion binding-GO:0005509,cl	185.66384	-1.516764663	0.42999644	-3.527389368	0.000419679	0.005983932
PITA_000066022	High quality	AT4G16890	MA_10428545g0010	shade>sun	Encodes a Toll Interleukin1 receptor-nucl	SUPPRESSOR OF NPR1-1, CONSTITI	ADP binding-GO:0043531,cytoplas	64.139906	-1.327783056	0.46366241	-2.863684901	0.004187443	0.042513037
PITA_000066234	High quality	AT3G11220	MA_10432066g0010	shade>sun	A subunit of Elongator, a histone acetyl tr	ELONGATA 1 (ELO1)	cell division-GO:0051301,cellular re	52.550218	-22.4390271	4.4083279	-5.09014474	3.58E-07	1.19E-05
PITA_000066271	High quality	AT3G08690	MA_194g0030	shade>sun	ubiquitin-conjugating enzyme 11;(source:	UBIQUITIN-CONJUGATING ENZYM	nucleus-GO:0005634,protein ubiqu	44.832887	0.851370716	0.29354983	2.900259661	0.003728536	0.038671674
PITA_000066391	High quality	AT4G32300	MA_10429842g0010	shade>sun	S-domain-2 5;(source:Araport11)	S-DOMAIN-2 5 (SD2-5)	calmodulin binding-GO:0005516,e>	59.504037	-9.144513047	1.82735605	-5.004231697	5.61E-07	1.77E-05
PITA_000066482	Low quality	AT1G19210	MA_314205g0010	shade>sun	encodes a member of the DREB subfamily	(ERF017)	DNA-binding transcription factor ac	30.357159	-21.69383454	4.40899933	-4.920353335	8.64E-07	2.39E-05
PITA_000066493	High quality	AT5G61110	MA_87055g0010	shade>sun	Arabidopsis ribosomal protein	RIBOSOMAL PROTEIN S27 (RS27A)	cell wall-GO:0005618,cytoplasm-G	104.13512	-0.829901144	0.23909946	-3.470945312	0.00051863	0.007163467
PITA_000066527	High quality	AT2G37050	MA_10430556g0020	shade>sun	Leucine-rich repeat protein kinase family protein;(source:Araport11)		ATP binding-GO:0005524,chloropla	17.404771	-20.94419205	4.41018187	-4.749054039	2.04E-06	4.59E-05

PITA_000066650	High quality	AT5G51010	MA_10432860g0010	shade<sun	Rubredoxin-like superfamily protein;(source:Araport11)		chloroplast-GO:0009507,iron ion b	366.73344	-1.190192609	0.24307995	-4.89630093	9.77E-07	2.64E-05
PITA_000066671	High quality	AT3G47550	MA_10435273g0010	shade<sun	RING/FYVE/PHD zinc finger superfamily protein;(source:Araport11)		cytoplasm-GO:0005737,membrane	60.714318	-22.53151824	4.4082046	-5.11126871	3.20E-07	1.18E-05
PITA_000066722	Low quality	AT2G39990	MA_8759628g0010	shade<sun	translation initiation factor eIF2 p47 subu	EUKARYOTIC TRANSLATION INITIAT	chloroplast-GO:0009507,cytoplasm	114.10134	-0.846956365	0.21889789	-3.869184737	0.0001092	0.001800638
PITA_000066828	High quality	AT2G46410	MA_9991g0010	shade<sun	Nuclear-localized R3-type MYB transcripti	CAPRICE (CPC)	cell differentiation-GO:0030154,DN	70.717523	-22.84103454	4.40809223	-5.181614484	2.20E-07	9.17E-06
PITA_000067174	High quality	AT2G32010	MA_116552g0010	shade>sun	Encodes an inositol polyphosphate 5?-pho	CVP2 LIKE 1 (CVL1)	cellular response to salt stress-GO:(	25.274645	1.372415488	0.4854701	2.826982546	0.004698889	0.046875398
PITA_000067201	High quality	AT1G28230	MA_5803g0010	shade<sun	Encodes a transporter that transports pur	PURINE PERMEASE 1 (PUP1)	chloroplast-GO:0009507,cytokinin	17.679381	-20.92872422	4.41014042	-4.745591347	2.08E-06	4.59E-05
PITA_000067230	High quality	AT5G21950	MA_394003g0010	shade>sun	alpha/beta-Hydrolases superfamily protein;(source:Araport11)		biological_process_unknown-GO:0	242.03887	24.86127221	4.32817767	5.744050756	9.24E-09	6.63E-07
PITA_000067231	Low quality	AT5G46330	MA_478919g0010	shade<sun	Encodes a leucine-rich repeat serine/thre	FLAGELLIN-SENSITIVE 2 (FLS2)	defense response by callose deposi	60.714318	-22.53151824	4.4082046	-5.11126871	3.20E-07	1.18E-05
PITA_000067357	Low quality	AT2G03890	MA_2516g0010	shade<sun	Phosphoinositide kinase which undergo a	PHOSPHOINOSITIDE 4-KINASE GAM	1-phosphatidylinositol 4-kinase acti	52.550218	-22.4390271	4.4083279	-5.09014474	3.58E-07	1.19E-05
PITA_000067504	High quality	AT1G18010	MA_140137g0010	shade<sun	Major facilitator superfamily protein;(source:Araport11)		molecular_function_unknown-GO:(	468.10188	-2.123060494	0.47643299	-4.456157625	8.34E-06	0.000166675
PITA_000067593	Low quality	AT4G24210	MA_233275g0010	shade<sun	F-box protein that is involved in GA signal	SLEEPY1 (SLY1)	gibberellic acid mediated signaling	70.717523	-22.84103454	4.40809223	-5.181614484	2.20E-07	9.17E-06
PITA_000067662	Low quality	AT1G75620	MA_10437233g0040	shade<sun	glyoxal oxidase-related protein;(source:Araport11)		biological_process_unknown-GO:0	23.572508	-21.35301597	4.4094576	-4.84254933	1.28E-06	3.20E-05
PITA_000067679	Low quality	AT3G56290	MA_55006g0010	shade<sun	potassium transporter;(source:Araport11)		chloroplast organization-GO:00096	3367.1505	-1.272247513	0.20983082	-6.063206067	1.33E-09	1.18E-07
PITA_000067694	High quality	AT1G20230	MA_482767g0010	shade<sun	Pentatricopeptide repeat (PPR) superfamily protein;(source:Araport11)		mitochondrion-GO:0005739,zinc io	472.1978	-1.378316567	0.31715481	-4.345879496	1.39E-05	0.000267091
PITA_000067762	High quality	AT1G55020	MA_10427506g0010	shade>sun	lipoxygenase, a defense gene conferring r	LIPOXYGENASE 1 (LOX1)	cytoplasm-GO:0005737,defense re	290.62975	1.910137329	0.56117694	3.403805805	0.00066454	0.008937781
PITA_000067860	Low quality	AT1G77280	MA_121123g0010	shade>sun	kinase with adenine nucleotide alpha hydrolases-like domain-containing prote		cytoplasm-GO:0005737,phosphory	108.02247	23.75154617	4.32841291	5.487356833	4.08E-08	2.16E-06
PITA_000067916	High quality	AT5G51110	MA_167002g0010	shade<sun	Encodes a protein involved in Rubisco ass	SDIR1-INTERACTING PROTEIN1 (SD	4-alpha-hydroxytetrahydrobiopteri	578.7848	-0.56118605	0.14038022	-3.997614704	6.40E-05	0.001105602
PITA_000067970	High quality	AT5G11950	MA_47673g0010	shade>sun	Encodes a protein of unknown function. It	LONELY GUY 8 (LOG8)	cytokinin biosynthetic process-GO:(	26.658018	2.696456541	0.66912083	4.029849949	5.58E-05	0.000980278
PITA_000068009	High quality	AT5G20480	MA_146510g0010	shade<sun	Encodes a predicted leucine-rich repeat re	EF-TU RECEPTOR (EFR)	detection of bacterium-GO:001604	160.82836	-1.097203784	0.26145263	-4.196568096	2.71E-05	0.000502499
PITA_000068043	High quality	AT3G22660	MA_138177g0010	shade>sun	rRNA processing protein-like protein;(sou	(EBP2)	GTPase activity-GO:0003924,nucle	181.52915	24.4760016	4.32824088	5.654953659	1.56E-08	1.02E-06
PITA_000068103	High quality	AT2G29040	MA_10434404g0030	shade<sun	Exostosin family protein;(source:Araport11)		Golgi apparatus-GO:0005794,prote	60.714318	-22.53151824	4.4082046	-5.11126871	3.20E-07	1.18E-05
PITA_000068128	High quality	AT5G57540	MA_8261658g0010	shade<sun	Encodes a xyloglucan endotransglucosyla	XYLOGLUCAN ENDOTRANSGLUCOS	cell wall biogenesis-GO:0042546,ex	573.21968	-12.41244749	1.46056855	-8.498366928	1.92E-17	8.45E-15
PITA_000068155	Low quality	AT5G53440	MA_93646g0010	shade>sun	LOW protein: zinc finger CCCH domain protein;(source:Araport11)		biological_process_unknown-GO:0	34.46958	1.011834967	0.32884922	3.076896343	0.00209168	0.023912387
PITA_000068237	High quality	AT1G71870	MA_334549g0010	shade<sun	Metabolite transporter involved in the an	(BIGE1A)	antiporter activity-GO:0015297,cyt	93.57	-1.555083477	0.3760012	-4.13584715	3.54E-05	0.000640015
PITA_000068247	Low quality	AT1G21460	MA_481649g0010	shade>sun	Nodulin MtN3 family protein;(source:Ara	(SWEET1)	carbohydrate transport-GO:000864	116.68171	0.826255392	0.19701887	4.193788025	2.74E-05	0.000508332
PITA_000068335	High quality	AT3G14420	MA_15820g0010	shade<sun	Encodes a glycolate oxidase that modulat	GLYCOLATE OXIDASE 1 (GOX1)	apoplast-GO:0048046,chloroplast-(	315.4139	-11.55016234	1.64601749	-7.017035007	2.27E-12	3.74E-10
PITA_000068417	High quality	AT1G74960	MA_10428839g0010	shade<sun	Encodes a plastidic beta-ketoacyl-ACP syn	FATTY ACID BIOSYNTHESIS 1 (FAB1	3-oxoacyl-[acyl-carrier-protein] syn	37.485822	-21.97331978	4.4086968	-4.984085037	6.23E-07	1.93E-05
PITA_000068615	High quality	AT4G13710	MA_10430145g0020	shade<sun	Pectin lyase-like superfamily protein;(source:Araport11)		extracellular region-GO:0005576,ly	521.86124	-1.555275426	0.53405077	-2.912223935	0.003588653	0.037632357
PITA_000068736	Low quality	AT3G19895	MA_109588g0010	shade>sun	RING/U-box superfamily protein;(source:Araport11)		biological_process_unknown-GO:0	61.234799	2.313730866	0.57439385	4.028126128	5.62E-05	0.000986815
PITA_000068740	High quality	AT1G12280	MA_10435336g0010	shade<sun	Encodes a NB-LRR protein SUMM2 involv	SUPPRESSOR OF MKK1 MKK2 2 (SU	ADP binding-GO:0043531,defense	106.51595	-1.805297378	0.42800122	-4.217972529	2.47E-05	0.000459432
PITA_000068821	High quality	AT1G56140	MA_10328256g0010	shade<sun	Leucine-rich repeat transmembrane protein kinase;(source:Araport11)		plasma membrane-GO:0005886,pr	26.275109	-21.49994244	4.40924566	-4.876104457	1.08E-06	2.80E-05
PITA_000068850	High quality	AT2G26650	MA_10432065g0010	shade>sun	Encodes AKT1, a member of the Shaker fa	K+ TRANSPORTER 1 (KT1)	identical protein binding-GO:00428	66.067748	1.205735962	0.3228065	3.735166252	0.000187591	0.002943335
PITA_000068881	High quality	AT1G27620	MA_10392111g0010	shade<sun	HXXXD-type acyl-transferase family protein;(source:Araport11)		nucleus-GO:0005634,transferase a	792.26925	-12.87910592	1.98445491	-6.489996751	8.58E-11	1.02E-08
PITA_000069015	Low quality	AT2G27510	MA_10264739g0010	shade<sun	ferredoxin 3;(source:Araport11)	FERREDOXIN 3 (FD3)	chloroplast-GO:0009507,electron t	131.82173	-0.762747255	0.24626691	-3.097238088	0.001953329	0.022582778
PITA_000069205	Low quality	AT5G32440	MA_9004g0010	shade>sun	Ubiquitin system component Cue protein;(source:Araport11)		nucleus-GO:0005634,ubiquitin binc	60.519935	0.928377775	0.28682147	3.236779251	0.001208869	0.014918898
PITA_000069214	Low quality	AT1G74930	MA_10062104g0010	shade<sun	encodes a member of the DREB subfamily	(ORA47)	cell division-GO:0051301,defense r	30.357159	-21.69383454	4.40899933	-4.920353335	8.64E-07	2.39E-05
PITA_000069232	High quality	AT5G49800	MA_952002g0010	shade>sun	Polyketide cyclase/dehydrase and lipid transport superfamily protein;(source:		lipid binding-GO:0008289,mitochoı	16.036422	1.848618552	0.54927853	3.365539431	0.000763941	0.010006656
PITA_000069337	High quality	AT5G16820	MA_41252g0020	shade<sun	Encodes a putative transcription factor wl	HEAT SHOCK FACTOR 3 (HSF3)	cellular response to heat-GO:0034€	1378.7518	-0.718427527	0.22879422	-3.140059776	0.001689134	0.019997843
PITA_000069506	High quality	AT2G38110	MA_10429049g0010	shade>sun	bifunctional sn-glycerol-3-phosphate 2-O-	GLYCEROL-3-PHOSPHATE SN-2-ACY	cutin biosynthetic process-GO:001(	63.408504	1.04839415	0.31848298	3.291837326	0.000995352	0.012612056
PITA_000069525	High quality	AT2G20030	MA_12352g0010	shade<sun	RING/U-box superfamily protein;(source:/	ARABIDOPSIS T??XICOS EN LEVADU	nucleus-GO:0005634,protein ubiqu	17.516739	-20.95349401	4.41016289	-4.751183692	2.02E-06	4.57E-05
PITA_000069529	High quality	AT3G04120	MA_10426544g0010	shade<sun	encodes cytosolic GADPH (C subunit) invo	GLYCERALDEHYDE-3-PHOSPHATE D	apoplast-GO:0048046,chloroplast-(	156.45526	-0.876426493	0.23957924	-3.658190426	0.000254002	0.003841991
PITA_000069616	Low quality	AT5G12010	MA_301324g0010	shade>sun	nuclease;(source:Araport11)		chloroplast-GO:0009507,nucleus-G<br>biological_process_unknown-GO:0008150,molecular_function_unknown-GO:0003674,cellular_component_unknown-GO:0005575	93.665495	2.146366938	0.6130094	3.501360586	0.000462889	0.006513002
PITA_000069686	High quality	AT3G02645	MA_10435738g0010	shade<sun	transmembrane protein, putative (DUF247);(source:Araport11)			20.238106	-21.14561367	4.4097939	-4.795147836	1.63E-06	3.81E-05
PITA_000069801	High quality	AT5G38280	MA_10435621g0020	shade>sun	putative receptor serine/threonine kinase	PR5-LIKE RECEPTOR KINASE (PR5K)	extracellular region-GO:0005576,ki	85.883473	1.852646817	0.5494612	3.371751828	0.000746917	0.00982386
PITA_000069951	High quality	AT1G67750	MA_101171g0010	shade>sun	Pectate lyase family protein;(source:Araport11)		extracellular region-GO:0005576,m	6934.8764	2.737485175	0.78799478	3.47398899	0.000512782	0.007090347
PITA_000069964	Low quality	AT1G61040	MA_1773g0010	shade>sun	Encodes a yeast Paf1C subunit homolog r	VERNALIZATION INDEPENDENCE 5	Cdc73/Paf1 complex-GO:0016593,	162.0337	24.31540594	4.32827129	5.617810044	1.93E-08	1.19E-06
PITA_000070030	High quality	AT1G68560	MA_102800g0010	shade<sun	Encodes a bifunctional alpha-l-arabinofur	ALPHA-XYLOSIDASE 1 (XYL1)	alpha-D-xyloside xylohydrolase-GO	52.550218	-22.4390271	4.4083279	-5.09014474	3.58E-07	1.19E-05
PITA_000070060	High quality	AT1G29820	MA_10436126g0010	shade>sun	Magnesium transporter CorA-like family protein;(source:Araport11)		metal ion transmembrane transpor	138.47259	0.923179817	0.21076301	4.380179608	1.19E-05	0.000230572
PITA_000070105	Low quality	AT1G66880	MA_10087424g0010	shade>sun	Protein kinase superfamily protein;(source:Araport11)		cellular response to hypoxia-GO:00	108.02247	23.75154617	4.32841291	5.487356833	4.08E-08	2.16E-06
PITA_000070107	High quality	AT4G08980	MA_206006g0010	shade<sun	Encodes an F-box gene that is a novel neg	F-BOX WITH WD-40 2 (FBW2)	negative regulation of gene express	26.275109	-21.49994244	4.40924566	-4.876104457	1.08E-06	2.80E-05
PITA_000070381	High quality	AT2G17260	MA_37580g0010	shade<sun	Encodes a glutamate receptor. Involved ir	GLUTAMATE RECEPTOR 2 (GLR2)	calcium channel activity-GO:00052	42.05307	-8.645464928	1.70958727	-5.05704804	4.26E-07	1.38E-05
PITA_000070434	High quality	AT5G17540	MA_253750g0010	shade>sun	HXXXD-type acyl-transferase family protein;(source:Araport11)		cytoplasm-GO:0005737,transferase	72.91381	1.269516895	0.37510218	3.384456165	0.000713194	0.009448253
PITA_000070479	High quality	AT1G73580	MA_130250g0010	shade>sun	Calcium-dependent lipid-binding (CaLB dc	C2-DOMAIN ABA-RELATED3 (CAR3	GTPase activator activity-GO:0005(	30.733159	1.609457819	0.36602326	4.397146301	1.10E-05	0.000215067
PITA_000070498	High quality	AT5G17330	MA_442130g0010	shade<sun	Encodes one of two isoforms of glutamate	GLUTAMATE DECARBOXYLASE (GA	calmodulin binding-GO:0005516,cy	401.32779	-1.344308955	0.17705871	-7.592447532	3.14E-14	8.20E-12
PITA_000070503	High quality	AT5G02860	MA_156405g0010	shade<sun	Pentatricopeptide repeat (PPR) superfamily protein;(source:Araport11)		biological_process_unknown-GO:0	350.91081	-0.792479147	0.22666624	-3.496238124	0.000471867	0.00662114
PITA_000070592	Low quality	AT1G58400	MA_777544g0010	shade<sun	Disease resistance protein (CC-NBS-LRR class) family;(source:Araport11)		ADP binding-GO:0043531	17.679381	-20.92872422	4.41014042	-4.745591347	2.08E-06	4.59E-05
PITA_000070615	High quality	AT3G46580	MA_92628g0020	shade<sun	Protein containing a putative methyl-CpG	METHYL-CPG-BINDING DOMAIN PI	enzyme binding-GO:0019899,heter	17.516739	-20.95349401	4.41016289	-4.751183692	2.02E-06	4.57E-05
PITA_000070643	High quality	AT5G01740	MA_39321g0010	shade<sun	Unknown gene, induced by abiotic stress treatments.		biological_process_unknown-GO:0	2954.7567	-2.055528719	0.64902871	-3.167084434	0.001539756	0.018416662
PITA_000070651	Low quality	AT5G41120	MA_86228g0010	shade<sun	Esterase/lipase/thioesterase family prote	(ELT4)	chloroplast-GO:0009507,transferas	52.550218	-22.4390271	4.4083279	-5.09014474	3.58E-07	1.19E-05
PITA_000070669	High quality	AT3G01410	MA_381654g0010	shade>sun	Polynucleotidyl transferase, ribonuclease H-like superfamily protein;(source:A		cytoplasm-GO:0005737,nucleic aci	52.744261	1.873121883	0.53572326	3.496435581	0.000471518	0.006619869
PITA_000070827	High quality	AT1G77700	MA_93606g0010	shade<sun	Pathogenesis-related thaumatin superfan	UBIQUITIN FOLD MODIFIER 1 (UFM	defense response-GO:0006952,ext	5.5129561	-5.720261318	1.66103218	-3.443799223	0.000573601	0.0078382
PITA_000070966	High quality	AT5G65700	MA_52165g0010	shade>sun	Encodes a CLAVATA1-related receptor kir	BARELY ANY MERISTEM 1 (BAM1)	anther development-GO:0048653,f	144.02996	24.14989881	4.3283067	5.57952578	2.41E-08	1.45E-06
PITA_000071009	High quality	AT1G73220	MA_6288g0010	shade>sun	Encodes Organic Cation Transporter 1 (O	ORGANIC CATION/CARNITINE TRA	cadaverine transport-GO:0015839,	148.36228	10.95666965	1.85924506	5.893074506	3.79E-09	3.06E-07
PITA_000071108	High quality	AT1G22640	MA_180449g0010	shade<sun	MYB-type transcription factor (MYB3) tha	MYB DOMAIN PROTEIN 3 (MYB3)	response to wounding-GO:000961	43.824626	-8.704513808	1.76960178	-4.918911084	8.70E-07	2.39E-05
PITA_000071154	High quality	AT4G26890	MA_15568g0010	shade<sun	Member of MEKK subfamily. Involved in v	MITOGEN-ACTIVATED PROTEIN KII	nucleus-GO:0005634,protein autop	20.238106	-21.14561367	4.4097939	-4.795147836	1.63E-06	3.81E-05
PITA_000071369	High quality	AT3G10390	MA_14874g0010	shade>sun	Encodes a plant homolog of a SWIRM dor	FLOWERING LOCUS D (FLD)	chloroplast-GO:0009507,histone de	45.80838	9.261038034	1.37475003	6.736525064	1.62E-11	2.28E-09
PITA_000071550	Low quality	AT2G19130	MA_10235107g0010	shade>sun	S-locus lectin protein kinase family protein;(source:Araport11)		calmodulin binding-GO:0005516,ex	121.01943	23.90838197	4.3283673	5.52364906	3.32E-08	1.87E-06
PITA_000071597	High quality	AT2G02400	MA_10435810g0010	shade<sun	NAD(P)-binding Rossmann-fold superfamily protein;(source:Araport11)		oxidoreductase activity, acting on t	40.476212	-21.86659121	4.40860183	-4.959983247	7.05E-07	2.04E-05
PITA_000071777	Low quality	AT3G49170	MA_185098g0010	shade<sun	Tetratricopeptide repeat (TPR)-like superf	EMBRYO DEFECTIVE 2261 (EMB22	chloroplast-GO:0009507,embryo d	277.95065	-1.28760231	0.29878947	-4.309396614	1.64E-05	0.000312373
PITA_000071810	High quality	AT3G48310	MA_10399g0010	shade<sun	putative cytochrome P450	CYTOCHROME P450, FAMILY 71, SI	heme binding-GO:0020037,iron ior	342.61099	-2.224930831	0.39299359	-5.661493919	1.50E-08	1.01E-06
PITA_000071917	Low quality	AT4G09340	MA_169781g0010	shade<sun	SPla/RYanodine receptor (SPRY) domain-containing protein;(source:Araport11		cell surface receptor signaling path	52.550218	-22.4390271	4.4083279	-5.09014474	3.58E-07	1.19E-05
PITA_000071926	High quality	AT5G44510	MA_10221218g0010	shade<sun	Encodes TAO1 (Target of AvrB Operation)	TARGET OF AVRB OPERATION1 (TA	ADP binding-GO:0043531,chloropl	23.572508	-21.35301597	4.4094576	-4.84254933	1.28E-06	3.20E-05
PITA_000071960	High quality	AT3G47520	MA_10431009g0010	shade<sun	Encodes a protein with NAD-dependent m	MALATE DEHYDROGENASE (MDH)	apoplast-GO:0048046,chloroplast-(	154.65592	-0.755423767	0.26746866	-2.82434494	0.004737737	0.04709781
PITA_000072001	High quality	AT4G19810	MA_10427204g0010	shade>sun	ChiC encodes a Class V chitinase that is a	CLASS V CHITINASE (ChiC)	cell wall-GO:0005618,chitin binding	28.008796	8.551694388	2.12426799	4.025713522	5.68E-05	0.000995221
PITA_000072155	Low quality	AT5G42200	MA_289646g0010	shade>sun	RING/U-box superfamily protein;(source:/	ARABIDOPSIS T??XICOS EN LEVADU	cellular response to hypoxia-GO:00	15.954459	7.739127923	2.23515522	3.462456598	0.000535268	0.007357568
PITA_000072253	High quality	AT1G24030	MA_9033g0010	shade<sun	Protein kinase superfamily protein;(sourc	PBS1-LIKE 28 (PBL28)	ATP binding-GO:0005524,mitochor	151.78403	-0.691124458	0.24419505	-2.830214824	0.004651676	0.046476831
PITA_000072503	High quality	AT1G76690	MA_592495g0010	shade<sun	Encodes one of the closely related 12-oxo	12-OXOPHYTODIENOATE REDUCTA	12-oxophytodienoate reductase act	275.85932	-1.189870326	0.39150764	-3.039200775	0.002372067	0.026712355
PITA_000072561	High quality	AT5G06570	MA_129063g0010	shade<sun	alpha/beta-Hydrolases superfamily protein;(source:Araport11)		hydrolase activity-GO:0016787,me	35.358761	-21.90137695	4.40877489	-4.967678662	6.78E-07	2.02E-05
PITA_000072598	High quality	AT4G39140	MA_1160135g0020	shade<sun	RING/U-box superfamily protein;(source:Araport11)		nucleus-GO:0005634	26.275109	-21.49994244	4.40924566	-4.876104457	1.08E-06	2.80E-05
PITA_000072599	High quality	AT3G19810	MA_466704g0010	shade<sun	BTB/POZ domain protein, putative (DUF1	DOMAIN OF UNKNOWN FUNCTIOI	chloroplast-GO:0009507,molecular	287.93767	-0.62419184	0.16940447	-3.684624287	0.00022904	0.003510031
PITA_000072605	Low quality	AT3G21480	MA_10427227g0050	shade<sun	BRCT domain-containing DNA repair protein;(source:Araport11)		biological_process_unknown-GO:0	367.96412	-11.7721581	1.47452192	-7.983711811	1.42E-15	4.38E-13
PITA_000072608	Low quality	AT4G15480	MA_195838g0010	shade<sun	Encodes a protein that might have sinapic	(UGT84A1)	cinnamate beta-D-glucosyltransfer	2202.3534	-3.390154694	0.68754414	-4.930817517	8.19E-07	2.34E-05
PITA_000072844	High quality	AT5G18590	MA_447541g0010	shade>sun	Galactose oxidase/kelch repeat superfamily protein;(source:Araport11)		biological_process_unknown-GO:0	68.326064	0.900730179	0.30175804	2.984941776	0.002836323	0.031026297
PITA_000073011	High quality	AT5G47500	MA_274107g0010	shade>sun	predicted to encode a pectin methylester	PECTIN METHYLESTERASE 5 (PME5	carboxylic ester hydrolase activity-(	40.521035	1.160213715	0.34323818	3.380200078	0.000724331	0.009575975
PITA_000073036	High quality	AT5G17680	MA_135183g0010	shade>sun	disease resistance protein (TIR-NBS-LRR class);(source:Araport11)		ADP binding-GO:0043531,chloropl	38.769724	2.163094655	0.74140418	2.917564669	0.003527765	0.03706657
PITA_000073053	High quality	AT3G13080	MA_101915g0010	shade<sun	encodes an ATP-dependent MRP-like ABC	ATP-BINDING CASSETTE C3 (ABCC3	ABC-type glutathione S-conjugate t	30.357159	-21.69383454	4.40899933	-4.920353335	8.64E-07	2.39E-05
PITA_000073094	High quality	AT2G26870	MA_10435847g0020	shade>sun	Non-specific phospholipase C2 involved ir	NON-SPECIFIC PHOSPHOLIPASE C2	chloroplast-GO:0009507,defense re	35.006133	8.87289845	1.93609299	4.582888582	4.59E-06	9.52E-05
PITA_000073289	High quality	AT5G42500	MA_8750226g0010	shade<sun	Disease resistance-responsive (dirigent-like protein) family protein;(source:Ar		extracellular region-GO:0005576	164.24022	-1.867693433	0.53240712	-3.508017389	0.00045146	0.00638728
PITA_000073314	Low quality	AT4G37420	MA_10973g0010	shade<sun	glycosyltransferase family protein (DUF23);(source:Araport11)		biological_process_unknown-GO:0	247.99325	-11.20394114	1.70230047	-6.581647202	4.65E-11	5.76E-09
PITA_000073394	Low quality	AT2G20030	MA_12352g0010	shade>sun	RING/U-box superfamily protein;(source:/	ARABIDOPSIS T??XICOS EN LEVADU	nucleus-GO:0005634,protein ubiqu	24.345255	2.704736814	0.64799914	4.173982115	2.99E-05	0.000550253
PITA_000073422	High quality	AT4G03420	MA_10431340g0010	shade>sun	hypothetical protein (DUF789);(source:Araport11)		biological_process_unknown-GO:0	46.592052	0.84963378	0.29072257	2.922489884	0.003472449	0.036589187
PITA_000073607	High quality	AT3G09930	MA_5971026g0020	shade<sun	GDSL-motif esterase/acyltransferase/lipase. Enzyme group with broad substr		hydrolase activity, acting on ester b	77.676279	-1.208852427	0.42711622	-2.830265773	0.004650935	0.046476831
PITA_000073728	High quality	AT4G09160	MA_9244g0010	shade>sun	PATLs belong to a family of proteins havir	(PATL5)	cellular response to auxin stimulus-	238.78962	11.64313155	2.48907003	4.67770348	2.90E-06	6.22E-05
PITA_000073858	High quality	AT5G18840	MA_10430268g0010	shade>sun	Major facilitator superfamily protein;(source:Araport11)		carbohydrate transmembrane tran	25.979128	1.390335634	0.39376985	3.530833164	0.000414253	0.005919749
PITA_000074129	High quality	AT2G25220	MA_135680g0010	shade<sun	Protein kinase superfamily protein;(source:Araport11)		chloroplast-GO:0009507,extracellu	137.9584	-10.35786206	2.11939783	-4.88717215	1.02E-06	2.75E-05
PITA_000074140	High quality	AT5G44050	MA_94941g0010	shade<sun	MATE efflux family protein;(source:Arapo	(ATDTX28)	antiporter activity-GO:0015297,me	186.5161	-0.631801243	0.17905652	-3.528501816	0.000417919	0.00596587
PITA_000074149	Low quality	AT3G61460	MA_169947g0010	shade>sun	Encodes a novel ring finger protein and fc	BRASSINOSTEROID-RESPONSIVE RI	brassinosteroid mediated signaling	34.162876	1.00635215	0.33731621	2.983408837	0.002850569	0.031142228
PITA_000074150	High quality	AT3G01750	MA_40566g0010	shade<sun	Ankyrin repeat family protein;(source:Araport11)		cytoplasm-GO:0005737	35.033479	-21.88927922	4.40878685	-4.964921176	6.87E-07	2.02E-05
PITA_000074157	High quality	AT3G52930	MA_101067g0010	shade<sun	Aldolase superfamily protein;(source:Ara	FRUCTOSE-BISPHOSPHATE ALDOLA	apoplast-GO:0048046,cell wall-GO	296.41641	-0.867410088	0.18763666	-4.622817715	3.79E-06	7.96E-05
PITA_000074177	Low quality	AT2G39670	MA_952069g0010	shade<sun	Radical SAM superfamily protein;(source:Araport11)		cytoplasm-GO:0005737,RNA methy	343.92733	-1.212843367	0.38861464	-3.120941038	0.001802741	0.021098994
PITA_000074179	High quality	AT2G03200	MA_19106g0010	shade<sun	Atypical aspartic protease which modulat	ATYPICAL ASPARTIC PROTEASE IN I	extracellular region-GO:0005576,p	465.22615	-3.752998084	0.54185613	-6.926189234	4.32E-12	6.75E-10
PITA_000074269	Low quality	AT4G39780	MA_66969g0010	shade<sun	encodes a member of the DREB subfamily	A-6 of ERF/AP2 transcription factor	DNA binding-GO:0003677,DNA-bin	122.96702	-0.727388043	0.24692645	-2.945768041	0.003221539	0.03445997
PITA_000074356	High quality	AT3G51680	MA_36042g0010	shade<sun	NAD(P)-binding Rossmann-fold superfami	SHORT-CHAIN DEHYDROGENASE/R	cytoplasm-GO:0005737	26.275109	-21.49994244	4.40924566	-4.876104457	1.08E-06	2.80E-05
PITA_000074362	High quality	AT1G01300	MA_333359g0010	shade<sun	Eukaryotic aspartyl protease family protein;(source:Araport11)		aspartic-type endopeptidase activit	60.273721	-9.163958029	1.47963684	-6.193383257	5.89E-10	5.63E-08
PITA_000074379	High quality	AT1G67820	MA_381787g0010	shade<sun	Protein phosphatase 2C family protein;(source:Araport11)		chloroplast-GO:0009507,magnesiu	160.58869	-0.785997943	0.18760117	-4.189728354	2.79E-05	0.000517138
PITA_000074422	High quality	AT1G22640	MA_446064g0010	shade<sun	MYB-type transcription factor (MYB3) tha	MYB DOMAIN PROTEIN 3 (MYB3)	response to wounding-GO:000961	413.81646	-1.521433002	0.12185044	-12.48606913	8.89E-36	3.80E-32
PITA_000074558	High quality	AT2G22070	MA_66499g0010	shade<sun	pentatricopeptide (PPR) repeat-containing protein;(source:Araport11)		intracellular membrane-bounded o	141.79692	-1.332963671	0.42300974	-3.151141789	0.001626335	0.019361781
PITA_000074626	High quality	AT5G27030	MA_10434172g0010	shade>sun	TOPLESS-related 3;(source:Araport11)	TOPLESS-RELATED 3 (TPR3)	cytoplasm-GO:0005737,primary sh	44.760845	9.227575499	2.47480039	3.728614049	0.000192536	0.003004644
PITA_000075062	Low quality	AT5G23690	MA_79766g0010	shade<sun	Polynucleotide adenylyltransferase family protein;(source:Araport11)		cellular_component_unknown-GO:	210.25717	-10.9658335	1.47090056	-7.455183458	8.97E-14	2.15E-11
PITA_000075150	Low quality	AT3G29185	MA_31719g0010	shade<sun	Encodes a chloroplast protein that interac	BIOGENESIS FACTOR REQUIRED FC	cell division-GO:0051301,chloropla	396.35981	-0.627608655	0.2136038	-2.938190469	0.003301341	0.035108284
PITA_000075217	Low quality	AT3G63540	MA_132558g0010	shade<sun	Conceptual translation of this open reading frame gave the sequence of a 22		calcium ion binding-GO:0005509,cy	2286.2667	-1.663027849	0.40215491	-4.135291679	3.55E-05	0.000640225
PITA_000075240	High quality	AT1G20560	MA_10436294g0010	shade<sun	acyl activating enzyme 1;(source:Araport	ACYL ACTIVATING ENZYME 1 (AAE	AMP binding-GO:0016208,chloropl	297.73574	-0.702785338	0.25023911	-2.808455187	0.004977981	0.048991598
PITA_000075320	Low quality	AT1G08070	MA_65500g0010	shade<sun	Encodes a chloroplast RNA editing factor.	ORGANELLE TRANSCRIPT PROCESS	chloroplast-GO:0009507,chloropla	242.18369	-1.069036795	0.36730966	-2.910451077	0.003609075	0.03778149
PITA_000075341	Low quality	AT1G53820	MA_222729g0010	shade<sun	RING/U-box superfamily protein;(source:/	ARABIDOPSIS T??XICOS EN LEVADU	nucleus-GO:0005634,protein ubiqu	26.275109	-21.49994244	4.40924566	-4.876104457	1.08E-06	2.80E-05
PITA_000075382	Low quality	AT1G36050	MA_56684g0010	shade<sun	Endoplasmic reticulum vesicle transporter protein;(source:Araport11)		COPII-coated ER to Golgi transport	36.403545	-8.434551227	1.85065713	-4.557597994	5.17E-06	0.000106598
PITA_000075562	Low quality	AT1G53330	MA_80720g0010	shade<sun	encodes a member of the pentatricopeptide repeat (PPR) gene family. T-DNA		chloroplast-GO:0009507,embryo d	84.63886	-0.809383424	0.27410414	-2.952831809	0.003148735	0.033856666
PITA_000075760	Low quality	AT3G53810	MA_10430511g0010	shade<sun	Concanavalin A-like lectin protein kinase f	L-TYPE LECTIN RECEPTOR KINASE I\	cytosol-GO:0005829,defense respo	1267.8058	-1.420222983	0.40754195	-3.484851031	0.000492411	0.006845637
PITA_000075797	Low quality	AT4G31390	MA_10436955g0010	shade<sun	Protein kinase superfamily protein;(sourc	ABC1-LIKE KINASE RELATED TO CHI	ATP binding-GO:0005524,cellular r	474.80969	-0.844508621	0.24117567	-3.50163279	0.000462416	0.006509929
PITA_000075879	High quality	AT5G25380	MA_28323g0010	shade<sun	core cell cycle genes	CYCLIN A2;1 (CYCA2;1)	cyclin-dependent protein kinase ho	1994.8335	-1.078435069	0.36764427	-2.93336563	0.003353088	0.035481763
PITA_000075981	High quality	AT4G11650	MA_10434201g0010	shade>sun	osmotin-like protein	OSMOTIN 34 (OSM34)	defense response-GO:0006952,def	151.27429	22.98752146	4.32829156	5.310991907	1.09E-07	5.25E-06
PITA_000076136	Low quality	AT5G22040	MA_117242g0010	shade<sun	ubiquitin carboxyl-terminal hydrolase;(source:Araport11)		biological_process_unknown-GO:0	254.69958	-11.24159671	1.67934754	-6.694026368	2.17E-11	2.96E-09
PITA_000076156	High quality	AT4G25810	MA_10430703g0010	shade>sun	xyloglucan endotransglycosylase-related	XYLOGLUCAN ENDOTRANSGLYCOS	cell wall-GO:0005618,cell wall biog	15.981174	2.441369584	0.61161601	3.991670474	6.56E-05	0.001129118
PITA_000076178	High quality	AT3G53700	MA_69152g0010	shade<sun	Pentatricopeptide repeat (PPR) superfami	MATERNAL EFFECT EMBRYO ARRE	chloroplast-GO:0009507,embryo d	147.83782	-0.832389606	0.27723348	-3.002485838	0.002677844	0.029583364
PITA_000076215	Low quality	AT1G76080	MA_99302g0010	shade<sun	Encodes a thioredoxin like protein. Locali	CHLOROPLASTIC DROUGHT-INDUC	cell redox homeostasis-GO:004545	640.02661	-1.186184973	0.25080508	-4.729509436	2.25E-06	4.92E-05
PITA_000076228	High quality	AT5G46220	MA_33920g0010	shade<sun	Encodes a protein with alkaline ceramida	TURGOR REGULATION DEFECT 1 (T	chloroplast-GO:0009507,dihydroce	17.679381	-20.92872422	4.41014042	-4.745591347	2.08E-06	4.59E-05
PITA_000076245	High quality	AT1G26560	MA_488148g0010	shade>sun	beta glucosidase 40;(source:Araport11)	BETA GLUCOSIDASE 40 (BGLU40)	apoplast-GO:0048046,beta-glucosi	471.08077	12.62340277	1.97026749	6.406948703	1.48E-10	1.67E-08
PITA_000076316	High quality	AT1G79220	MA_280158g0010	shade>sun	Mitochondrial transcription termination f	(MTERF17)	chloroplast-GO:0009507,chloropla	64.257678	1.743990039	0.51228884	3.404310048	0.000663314	0.008925985
PITA_000076385	Low quality	AT2G44810	MA_10436267g0010	shade<sun	Mutant has defects in anther dehiscence,	DEFECTIVE ANTHER DEHISCENCE 1	1-acyl-2-lysophosphatidylserine acy	18.289356	-7.440812118	1.87377109	-3.971035823	7.16E-05	0.001224953
PITA_000076462	Low quality	AT5G15600	MA_110382g0010	shade<sun	SPIRAL1-LIKE4 belongs to a six-member g	SPIRAL1-LIKE4 (SP1L4)	cortical microtubule organization-G	266.54195	-0.554269499	0.167195	-3.315108156	0.000916077	0.011741188
PITA_000076746	High quality	AT5G13120	MA_275653g0010	shade<sun	Encodes a lumenal cyclophilin with peptic	PHOTOSYNTHETIC NDH SUBCOMP	chloroplast-GO:0009507,chloropla	84.065626	-2.843609909	0.54501346	-5.217504036	1.81E-07	8.29E-06
PITA_000076762	High quality	AT1G28650	MA_10376648g0010	shade<sun	GDSL-motif esterase/acyltransferase/lipase. Enzyme group with broad substr		extracellular region-GO:0005576,h	52.550218	-22.4390271	4.4083279	-5.09014474	3.58E-07	1.19E-05
PITA_000076804	High quality	AT1G11080	MA_118714g0010	shade<sun	serine carboxypeptidase-like 31;(source:A	SERINE CARBOXYPEPTIDASE-LIKE 3	extracellular region-GO:0005576,se	126.87846	-1.477380686	0.32761375	-4.509519747	6.50E-06	0.000132474
PITA_000077027	High quality	AT5G09650	MA_10426244g0010	shade<sun	Encodes a protein with inorganic pyrophc	PYROPHOSPHORYLASE 6 (PPa6)	chloroplast-GO:0009507,chloropla	52.550218	-22.4390271	4.4083279	-5.09014474	3.58E-07	1.19E-05
PITA_000077043	High quality	AT5G39980	MA_112272g0010	shade<sun	PDM3 belongs to the P subfamily of PPR	EMBRYO LETHAL 3140 (EMB3140)	chloroplast-GO:0009507,chloropla	206.43543	-1.10888678	0.39260276	-2.824449773	0.004736187	0.04709781
PITA_000077159	High quality	AT3G15730	MA_6712g0010	shade<sun	Encodes phospholipase D alpha 1 (PLD al	PHOSPHOLIPASE D ALPHA 1 (PLDAI	abscisic acid-activated signaling pat	200.35452	-0.603845197	0.18444453	-3.273857958	0.001060899	0.013290009
PITA_000077173	High quality	AT2G28490	MA_286762g0010	shade<sun	RmlC-like cupins superfamily protein;(source:Araport11)		biological_process_unknown-GO:0	23.572508	-21.35301597	4.4094576	-4.84254933	1.28E-06	3.20E-05
PITA_000077283	High quality	AT5G65550	MA_36867g0010	shade<sun	UDP-Glycosyltransferase superfamily protein;(source:Araport11)		chloroplast-GO:0009507,intracellu	1923.8065	-5.730939694	0.64299472	-8.91288765	4.97E-19	3.43E-16
PITA_000077290	High quality	AT4G24340	MA_10428395g0010	shade<sun	Phosphorylase superfamily protein;(source:Araport11)		catalytic activity-GO:0003824,cyto	374.15279	-2.370526957	0.26080137	-9.089396054	9.96E-20	7.73E-17
PITA_000077389	High quality	AT5G59845	MA_132217g0010	shade<sun	Member of a family of proteins named as	GIBBERELLIC ACID STIMULATED AI	cytoplasm-GO:0005737,extracellul	184.39787	-1.648699385	0.54584742	-3.020439981	0.002524077	0.028176361
PITA_000077570	High quality	AT5G15290	MA_93690g0010	shade>sun	Uncharacterized protein family (UPF0497	CASPARIAN STRIP MEMBRANE DOI	4 iron, 4 sulfur cluster binding-GO:(	68.616411	2.337684716	0.8112478	2.88159145	0.003956724	0.040497117
PITA_000077719	High quality	AT3G25810	MA_10121006g0010	shade<sun	Terpenoid cyclases/Protein prenyltransferases superfamily protein;(source:Ar		(4S)-limonene synthase activity-GO	263.2064	-3.089951528	0.61489819	-5.025143283	5.03E-07	1.60E-05
PITA_000077730	Low quality	AT3G07350	MA_6561322g0010	shade>sun	sulfate/thiosulfate import ATP-binding protein, putative (DUF506);(source:Ar		cellular response to hypoxia-GO:00	19.086825	7.998022172	1.69640438	4.714690833	2.42E-06	5.26E-05
PITA_000077781	Low quality	AT1G17370	MA_10429328g0010	shade>sun	Encodes an RNA?binding protein involved	OLIGOURIDYLATE BINDING PROTEI	mRNA binding-GO:0003729,protei	16.41467	7.780435965	1.80567455	4.308880559	1.64E-05	0.000312869

PITA_000077840	High quality	AT1G80160	MA_15220g0010	shade<sun	Vicinal oxygen chelate (VOC) superfamily	GLYOXYLASE I 7 (GLYI7)	cytoplasm-GO:0005737	66.756815	-1.341880389	0.44620911	-3.007290437	0.002635878	0.02918269
PITA_000077857	High quality	AT1G74950	MA_68496g0010	shade<sun	Key regulator in alternative splicing in the	(TIFY10B)	identical protein binding-GO:00428	105.12858	-9.966161933	1.47429265	-6.759961755	1.38E-11	2.00E-09
PITA_000077860	High quality	AT3G55740	MA_184924g0010	shade>sun	Encodes a proline transporter with affinit	PROLINE TRANSPORTER 2 (PROT2)	amino acid transmembrane transp	69.852379	1.348291059	0.38703568	3.483635041	0.000494654	0.006869353
PITA_000077875	High quality	AT4G20140	MA_10429751g0010	shade<sun	Encodes GASSHO1 (GSO1), a putative leu	GASSHO1 (GSO1)	Casparian strip-GO:0048226,embry	17.679381	-20.92872422	4.41014042	-4.745591347	2.08E-06	4.59E-05
PITA_000078156	High quality	AT3G07350	MA_13827g0010	shade<sun	sulfate/thiosulfate import ATP-binding protein, putative (DUF506);(source:Ara		cellular response to hypoxia-GO:00	53.038142	-22.45050813	4.40831978	-5.092758525	3.53E-07	1.19E-05
PITA_000078199	High quality	AT3G29970	MA_126544g0010	shade<sun	B12D protein;(source:Araport11)		biological_process_unknown-GO:0	20.238106	-21.14561367	4.4097939	-4.795147836	1.63E-06	3.81E-05
PITA_000078207	Low quality	AT2G30350	MA_10429286g0010	shade<sun	Excinuclease ABC, C subunit, N-terminal;(	HYL1 INTERACTING GIY-YIG LIKE EN	5'-flap endonuclease activity-GO:00	170.44593	-0.542999023	0.18183083	-2.986286834	0.002823876	0.03092978
PITA_000078355	Low quality	AT3G10340	MA_10429279g0010	shade<sun	Encodes PAL4, a putative a phenylalanine	PHENYLALANINE AMMONIA-LYASE	ammonia-lyase activity-GO:001684	32.153988	-8.256177517	1.87070605	-4.41340184	1.02E-05	0.000201068
PITA_000078553	Low quality	AT1G73880	MA_57964g0010	shade<sun	UDP-glucosyl transferase 89B1;(source:Ar	UDP-GLUCOSYL TRANSFERASE 89B	daphnetin 3-O-glucosyltransferase	498.62921	-1.160362399	0.36619933	-3.168663321	0.001531417	0.018334045
PITA_000078556	High quality	AT1G78440	MA_26738g0010	shade<sun	Encodes a gibberellin 2-oxidase that acts	ARABIDOPSIS THALIANA GIBBEREL	C-19 gibberellin 2-beta-dioxygenase	699.888	-3.074174327	0.45996116	-6.683551928	2.33E-11	3.13E-09
PITA_000078623	High quality	AT1G22640	MA_8147g0020	shade<sun	MYB-type transcription factor (MYB3) tha	MYB DOMAIN PROTEIN 3 (MYB3)	response to wounding-GO:000961	1330.2923	-2.798657908	0.30930865	-9.048107519	1.45E-19	1.10E-16
PITA_000078652	Low quality	AT3G22400	MA_3501g0020	shade>sun	Encodes lipoxygenase5 (LOX5). LOX5 activ	(LOX5)	cytoplasm-GO:0005737,lateral roo	12.733009	1.899791245	0.59833033	3.175154509	0.001497567	0.017996108
PITA_000078699	Low quality	AT3G62630	MA_70531g0010	shade<sun	stress response NST1-like protein (DUF1645);(source:Araport11)		biological_process_unknown-GO:0	52.550218	-22.4390271	4.4083279	-5.09014474	3.58E-07	1.19E-05
PITA_000078742	High quality	AT3G24240	MA_18697g0010	shade>sun	RGFR1 is a leucine--rich repeat receptor k	(RGFR1)	maintenance of root meristem iden	77.04093	0.899394495	0.32070372	2.804440464	0.0050404	0.049441069
PITA_000078761	High quality	AT4G13420	MA_100446g0010	shade<sun	Encodes a protein of the KUP/HAK/KT pot	HIGH AFFINITY K+ TRANSPORTER 5	membrane-GO:0016020,nucleus-G	682.02136	-1.293038961	0.35076349	-3.686355621	0.000227488	0.003488337
PITA_000078764	Low quality	AT1G15690	MA_121856g0010	shade<sun	Encodes a H(+)-translocating (pyrophosph	(AVP1)	auxin polar transport-GO:0009926,	210.57886	-0.834332036	0.25010958	-3.335865984	0.000850341	0.011008869
PITA_000078883	High quality	AT1G32100	MA_10432947g0010	shade<sun	Encodes a pinoresinol reductase involved	PINORESINOL REDUCTASE 1 (PRR1	cytoplasm-GO:0005737,lignan bios	329.0431	-1.198856214	0.21183303	-5.659439448	1.52E-08	1.01E-06
PITA_000079020	Low quality	AT1G02040	MA_178315g0010	shade<sun	C2H2-type zinc finger family protein;(source:Araport11)		DNA-binding transcription factor ac	211.63154	-1.667475186	0.44516424	-3.745752735	0.000179854	0.002834273
PITA_000079033	High quality	AT3G11980	MA_55232g0010	shade<sun	Similar to fatty acid reductases.	MALE STERILITY 2 (MS2)	alcohol-forming fatty acyl-CoA redu	71.126397	-1.152989689	0.39370716	-2.928546385	0.00340551	0.035977023
PITA_000079043	High quality	AT3G23730	MA_44609g0010	shade<sun	xyloglucan endotransglucosylase/hydrola	XYLOGLUCAN ENDOTRANSGLUCOS	cell wall biogenesis-GO:0042546,ex	153.64366	-10.51244154	2.09039426	-5.028927669	4.93E-07	1.57E-05
PITA_000079098	High quality	AT3G57810	MA_20446g0010	shade<sun	Cysteine proteinases superfamily protein;(source:Araport11)		chloroplast-GO:0009507,mitochon	103.74418	-0.695993229	0.2008849	-3.464636798	0.000530948	0.007302104
PITA_000079120	High quality	AT5G06290	MA_92546g0010	shade<sun	Encodes a 2-Cys peroxiredoxin (2-Cys PrxI	2-CYSTEINE PEROXIREDOXIN B (2-C	antioxidant activity-GO:0016209,ap	402.45846	-0.790625961	0.26041354	-3.036040124	0.002397075	0.026970249
PITA_000079217	High quality	AT1G80830	MA_100211g0010	shade<sun	Thought to be involved in iron homeostas	NATURAL RESISTANCE-ASSOCIATEI	cadmium ion transmembrane trans	186.6659	-10.7940901	1.90241379	-5.673891839	1.40E-08	9.46E-07
PITA_000079320	High quality	AT2G38970	MA_15445g0010	shade>sun	Zinc finger (C3HC4-type RING finger) family protein;(source:Araport11)		extracellular region-GO:0005576	47.747819	0.917365777	0.27924536	3.285160277	0.001019244	0.012832249
PITA_000079467	Low quality	AT3G54090	MA_659042g0010	shade<sun	Encodes a fructokinase-like protein (AT3G	FRUCTOKINASE-LIKE 1 (FLN1)	chloroplast nucleoid-GO:0042644,c	26.275109	-21.49994244	4.40924566	-4.876104457	1.08E-06	2.80E-05
PITA_000079538	High quality	AT3G50170	MA_86436g0010	shade<sun	transmembrane protein, putative (DUF247);(source:Araport11)		biological_process_unknown-GO:0	235.29684	-2.285123071	0.62099269	-3.679790589	0.000233426	0.003569909
PITA_000079568	High quality	AT4G19420	MA_52077g0010	shade<sun	Pectinacetylesterase family protein;(sourc	PECTIN ACETYLESTERASE 8 (PAE8)	cell wall organization-GO:0071555,	397.40581	-2.46991554	0.4550241	-5.428098266	5.70E-08	2.93E-06
PITA_000079585	Low quality	AT2G44130	MA_10305005g0020	shade<sun	Encodes a member of a family of F-box pr	KISS ME DEADLY 3 (KMD3)	cytoplasm-GO:0005737,cytosol-GC	812.13282	-2.610562559	0.72652283	-3.593228506	0.000326606	0.004776542
PITA_000079630	High quality	AT5G66120	MA_4908g0010	shade<sun	3-dehydroquinate synthase;(source:Araport11)		3-dehydroquinate synthase activity	270.80943	-0.762155844	0.17046955	-4.470920813	7.79E-06	0.00015655
PITA_000079828	Low quality	AT5G25810	MA_82481g0010	shade>sun	encodes a member of the DREB subfamily	TINY (tny)	DNA-binding transcription factor ac	89.4797	0.992144825	0.35418674	2.801191365	0.005091432	0.049801903
PITA_000079852	Low quality	AT3G48090	MA_422896g0010	shade>sun	Component of R gene-mediated disease r	ENHANCED DISEASE SUSCEPTIBILIT	aerenchyma formation-GO:001061	108.02247	23.75154617	4.32841291	5.487356833	4.08E-08	2.16E-06
PITA_000079989	High quality	AT4G13650	MA_653417g0010	shade<sun	Pentatricopeptide repeat (PPR) superfamily protein;(source:Araport11)		mitochondrion-GO:0005739,zinc io	595.63775	-1.917976074	0.31569068	-6.075491658	1.24E-09	1.11E-07
PITA_000080057	High quality	AT1G48300	MA_585559g0010	shade<sun	Cytosolic iron-sulfur protein with a [2Fe-2	DIACYLGLYCEROL ACYLTRANSFERA	cytosol-GO:0005829,diacylglycerol	194.59179	-1.612248959	0.30603192	-5.268237875	1.38E-07	6.41E-06
PITA_000080147	Low quality	AT2G16920	MA_85108g0010	shade>sun	ubiquitin-conjugating enzyme 23;(source:	UBIQUITIN-CONJUGATING ENZYMI	cytosol-GO:0005829,nucleus-GO:0	138.27733	10.85509623	2.06891237	5.246764609	1.55E-07	7.12E-06
PITA_000080189	High quality	AT1G71790	MA_51020g0020	shade<sun	Encodes a heterodimeric actin binding pr	CAPPING PROTEIN B (CPB)	actin cytoskeleton organization-GO	180.64343	-0.808885416	0.27565803	-2.93438007	0.003342147	0.035420895
PITA_000080210	High quality	AT5G41800	MA_95623g0010	shade>sun	Transmembrane amino acid transporter family protein;(source:Araport11)		amino acid transmembrane transp	87.560963	1.07004838	0.3187965	3.356524832	0.000789286	0.010317532
PITA_000080233	High quality	AT5G60770	MA_80387g0010	shade<sun	member of High affinity nitrate transport	NITRATE TRANSPORTER 2.4 (NRT2.	cellular response to nitrate-GO:007	959.6575	-1.717876938	0.52696232	-3.259961633	0.001114273	0.013885304
PITA_000080249	High quality	AT3G28860	MA_10431815g0020	shade>sun	Encodes a member of the ATP-binding ca	ATP-BINDING CASSETTE B19 (ABCB	acropetal auxin transport-GO:0010	222.54342	11.54160687	1.83496514	6.289823502	3.18E-10	3.22E-08
PITA_000080483	High quality	AT4G35880	MA_32374g0010	shade<sun	Eukaryotic aspartyl protease family protein;(source:Araport11)		anchored component of membrane	21.973785	-2.671068635	0.8979029	-2.974785606	0.002931934	0.031908611
PITA_000080632	High quality	AT2G23540	MA_10434622g0020	shade>sun	GDSL-motif esterase/acyltransferase/lipase. Enzyme group with broad substr		chloroplast-GO:0009507,hydrolase	70.356025	1.135078551	0.3612488	3.142096402	0.001677428	0.019896052
PITA_000080705	Low quality	AT1G72710	MA_6447g0010	shade>sun	Encodes a member of the casein kinase 1	CASEIN KINASE 1-LIKE PROTEIN 2 (	cytoplasm-GO:0005737,endocytosi	78.513461	10.03834398	2.08477203	4.815079944	1.47E-06	3.59E-05
PITA_000080797	High quality	AT5G13200	MA_10434405g0010	shade>sun	Encodes a protein with unknown function	GEM-RELATED 5 (GER5)	cytoplasm-GO:0005737,embryo de	83.367428	1.452852296	0.46516381	3.123313238	0.001788273	0.020977599
PITA_000080874	Low quality	AT1G17615	MA_10433120g0010	shade<sun	TN2 is an atypical TIR-NBS protein that la	TIR-NBS2 (TN2)	ADP binding-GO:0043531,chloropl	60.714318	-22.53151824	4.4082046	-5.11126871	3.20E-07	1.18E-05
PITA_000080884	High quality	AT5G02070	MA_5680g0010	shade<sun	Protein kinase family protein;(source:Araport11)		cell surface receptor signaling path	4281.7754	-2.33694721	0.68891077	-3.392234972	0.00069325	0.009245933
PITA_000080919	High quality	AT2G16850	MA_10177437g0010	shade<sun	plasma membrane intrinsic protein 2;(sou	PLASMA MEMBRANE INTRINSIC PF	cytoplasm-GO:0005737,plasma me	509.39916	-12.24180607	1.67610925	-7.303704126	2.80E-13	5.92E-11
PITA_000081147	Low quality	AT4G24220	MA_10437028g0020	shade<sun	encodes a progesterone-5beta-reductase	VEIN PATTERNING 1 (VEP1)	cytosol-GO:0005829,delta4-3-oxos	48.627997	-1.2519494	0.39623501	-3.159613305	0.001579787	0.018851433
PITA_000081324	High quality	AT3G58680	MA_103473g0010	shade>sun	One of three genes in A. thaliana encodin	MULTIPROTEIN BRIDGING FACTOR	cytoplasm-GO:0005737,cytosol-GC	43.482146	1.453893618	0.46484868	3.127670742	0.001761974	0.020697542
PITA_000081622	High quality	AT1G26760	MA_135344g0010	shade<sun	SET domain protein 35;(source:Araport11	SET DOMAIN PROTEIN 35 (SDG35)	biological_process_unknown-GO:0	144.54125	-10.42535697	1.66858671	-6.248016303	4.16E-10	4.14E-08
PITA_000081672	Low quality	AT3G10210	MA_10433197g0010	shade<sun	SEC14 cytosolic factor family protein / phosphoglyceride transfer family prote		biological_process_unknown-GO:0	35.358761	-21.90137695	4.40877489	-4.967678662	6.78E-07	2.02E-05
PITA_000081818	High quality	AT4G38400	MA_10430035g0010	shade>sun	member of EXPANSIN-LIKE. Naming conv	EXPANSIN-LIKE A2 (EXLA2)	extracellular region-GO:0005576,p	19.744869	1.910342735	0.64652052	2.954806042	0.003128657	0.033677445
PITA_000081945	Low quality	AT1G51310	MA_10436627g0010	shade<sun	tRNA (5-methylaminomethyl-2-thiouridylate)-methyltransferase;(source:Arap		chloroplast-GO:0009507,sulfurtran	63.770371	-2.219014616	0.32802265	-6.764821364	1.33E-11	1.94E-09
PITA_000082413	Low quality	AT4G19970	MA_607856g0010	shade>sun	nucleotide-diphospho-sugar transferase family protein;(source:Araport11)		cytoplasm-GO:0005737	37.09057	8.956821878	2.05786493	4.352482871	1.35E-05	0.000259951
PITA_000082449	High quality	AT5G47840	MA_10427036g0010	shade<sun	adenosine monophosphate kinase;(sourc	ADENOSINE MONOPHOSPHATE KI	adenylate kinase activity-GO:0004C	22441.04	-0.842351577	0.15018874	-5.608620074	2.04E-08	1.25E-06
PITA_000082538	Low quality	AT5G23190	MA_10178635g0020	shade<sun	cytochrome P450 CYP86B1, nuclear gene	CYTOCHROME P450, FAMILY 86, SI	chloroplast-GO:0009507,endoplasr	17.516739	-20.95349401	4.41016289	-4.751183692	2.02E-06	4.57E-05
PITA_000082596	High quality	AT4G16260	MA_219456g0010	shade<sun	Encodes a putative beta-1,3-endoglucanase that interacts with the 30C02 cyst		anchored component of plasma me	55.805249	-3.528194115	1.16313931	-3.033337529	0.002418649	0.02714144
PITA_000082666	High quality	AT1G12580	MA_10430455g0020	shade<sun	phosphoenolpyruvate carboxylase-relatec	PHOSPHOENOLPYRUVATE CARBOX	calcium-dependent protein serine/t	60.714318	-22.53151824	4.4082046	-5.11126871	3.20E-07	1.18E-05
PITA_000082667	Low quality	AT1G63450	MA_9990400g0010	shade<sun	Encodes a xyloglucan-specific galacturonc	ROOT HAIR SPECIFIC 8 (RHS8)	cell wall biogenesis-GO:0042546,G	1002.2572	-3.546999692	0.94529167	-3.752280687	0.000175233	0.002771692
PITA_000082733	Low quality	AT1G08700	MA_474310g0010	shade<sun	Encodes a protein similar to animal prese	PRESENILIN-1 (PS1)	aspartic-type endopeptidase activit	59.57496	-1.49061566	0.31839631	-4.681636046	2.85E-06	6.11E-05
PITA_000082807	Low quality	AT5G18980	MA_23409g0010	shade<sun	ARM repeat superfamily protein;(source:Araport11)		biological_process_unknown-GO:0	80.879754	-1.934100635	0.48158296	-4.016131788	5.92E-05	0.001032016
PITA_000082884	Low quality	AT3G26140	MA_463555g0010	shade<sun	Cellulase (glycosyl hydrolase family 5) protein;(source:Araport11)		extracellular region-GO:0005576,m	255.16756	-0.721856852	0.25688471	-2.810042098	0.004953502	0.048863359
PITA_000082917	High quality	AT1G11650	MA_953867g0010	shade<sun	Encodes an RNA binding protein with thre	(RBP45B)	cytosol-GO:0005829,extracellular r	38.651429	-1.248602541	0.42999764	-2.903742751	0.00368731	0.038399607
PITA_000083181	High quality	AT1G56150	MA_84242g0010	shade<sun	SAUR-like auxin-responsive protein family	SMALL AUXIN UPREGULATED 71 (S	cytoplasm-GO:0005737,molecular_	47.145015	-22.29213208	4.40843355	-5.056701391	4.27E-07	1.38E-05
PITA_000083329	Low quality	AT1G33140	MA_38141g0010	shade<sun	Encodes ribosomal protein L9. Identified i	PIGGYBACK2 (PGY2)	adaxial/abaxial pattern specificatio	158.57922	-0.718266272	0.19748863	-3.637000558	0.000275831	0.004137955
PITA_000083410	High quality	AT4G23900	MA_10428684g0020	shade<sun	Nucleoside diphosphate kinase family protein;(source:Araport11)		chloroplast-GO:0009507,CTP biosy	181.27591	-0.897743034	0.20485407	-4.382353985	1.17E-05	0.000228978
PITA_000083608	High quality	AT1G14870	MA_102043g0010	shade>sun	PCR2 encodes a membrane protein involv	PLANT CADMIUM RESISTANCE 2 (P	cytoplasm-GO:0005737,molecular_	11.929209	1.979112898	0.641413	3.085551607	0.00203175	0.02336284
PITA_000083624	Low quality	AT3G53040	MA_111913g0010	shade>sun	late embryogenesis abundant protein, putative / LEA protein;(source:Araport		cytosol-GO:0005829,floral organ al	92.184885	10.27016666	2.10633483	4.875847151	1.08E-06	2.80E-05
PITA_000083709	Low quality	AT1G26650	MA_8219g0010	shade<sun	Son of sevenless protein;(source:Araport11)		biological_process_unknown-GO:0	20.238106	-21.14561367	4.4097939	-4.795147836	1.63E-06	3.81E-05
PITA_000083749	High quality	AT2G39210	MA_10432157g0010	shade<sun	Major facilitator superfamily transmembr	PICLORAM RESISTANT30 (PIC30)	chloroplast-GO:0009507,inorganic	63.08278	-9.227115615	1.66252228	-5.550070337	2.86E-08	1.69E-06
PITA_000084088	High quality	AT5G13840	MA_10290598g0010	shade>sun	FIZZY-related 3;(source:Araport11)	FIZZY-RELATED 3 (FZR3)	anaphase-promoting complex-GO:(	76.347301	9.998017361	1.31266935	7.616554267	2.61E-14	6.95E-12
PITA_000084107	Low quality	AT1G79630	MA_2329g0010	shade>sun	Protein phosphatase 2C family protein;(source:Araport11)		chloroplast-GO:0009507,cytoplasm	30.709224	1.083367053	0.3643783	2.973193094	0.00294719	0.032036802
PITA_000084196	High quality	AT4G23180	MA_9664966g0010	shade<sun	Encodes a receptor-like protein kinase. Na	CYSTEINE-RICH RLK (RECEPTOR-LIK	cytosol-GO:0005829,defense respo	40.476212	-21.86659121	4.40860183	-4.959983247	7.05E-07	2.04E-05
PITA_000084229	Low quality	AT4G10500	MA_9118954g0010	shade>sun	2-oxoglutarate (2OG) and Fe(II)-depender	DMR6-LIKE OXYGENASE 1 (DLO1)	chloroplast-GO:0009507,defense re	108.02247	23.75154617	4.32841291	5.487356833	4.08E-08	2.16E-06
PITA_000084230	High quality	AT5G05340	MA_10070756g0010	shade<sun	Encodes a protein with sequence similarit	PEROXIDASE 52 (PRX52)	apoplast-GO:0048046,cell wall-GO	60.714318	-22.53151824	4.4082046	-5.11126871	3.20E-07	1.18E-05
PITA_000084257	Low quality	AT1G65900	MA_49077g0010	shade>sun	plant/protein;(source:Araport11)		biological_process_unknown-GO:0	70.176955	0.722585066	0.23572057	3.065430702	0.002173567	0.024727105
PITA_000084427	High quality	AT5G48940	MA_705973g0010	shade<sun	RGFR2 is a leucine--rich repeat receptor k	(RGFR2)	maintenance of root meristem iden	26.275109	-21.49994244	4.40924566	-4.876104457	1.08E-06	2.80E-05
PITA_000084468	High quality	AT1G17230	MA_10047384g0010	shade<sun	Leucine-rich receptor-like protein kinase family protein;(source:Araport11)		plasma membrane-GO:0005886,pr	26.275109	-21.49994244	4.40924566	-4.876104457	1.08E-06	2.80E-05
PITA_000084544	Low quality	AT1G29070	MA_10435644g0010	shade<sun	Ribosomal protein L34;(source:Araport11	PLASTID RIBOSOMAL PROTEINS OF	chloroplast-GO:0009507,mitochon	364.31593	-0.857367761	0.15187498	-5.64522066	1.65E-08	1.06E-06
PITA_000084575	High quality	AT1G49760	MA_111003g0010	shade<sun	polyadenylate-binding protein, putative /	POLY(A) BINDING PROTEIN 8 (PAB8	cytoplasm-GO:0005737,cytosol-GC	91.982743	-1.047026001	0.35963653	-2.911344961	0.003598765	0.037707514
PITA_000084868	Low quality	AT5G44510	MA_553625g0020	shade>sun	Encodes TAO1 (Target of AvrB Operation)	TARGET OF AVRB OPERATION1 (TA	ADP binding-GO:0043531,chloropl	71.025877	1.880918466	0.30511072	6.164707862	7.06E-10	6.67E-08
PITA_000084952	Low quality	AT3G19430	MA_10430750g0010	shade<sun	late embryogenesis abundant protein-related / LEA protein-like protein;(sourc		extracellular region-GO:0005576,n	2434.375	-1.774260166	0.23844506	-7.440959987	1.00E-13	2.37E-11
PITA_000084998	Low quality	AT3G16510	MA_10427865g0010	shade>sun	Calcium-dependent lipid-binding (CaLB domain) family protein;(source:Arapo		biological_process_unknown-GO:0	20.270607	1.964973384	0.6115371	3.213171204	0.00131278	0.016031318
PITA_000085062	High quality	AT4G28780	MA_15196g0010	shade<sun	GDSL-motif esterase/acyltransferase/lipase. Enzyme group with broad substr		chloroplast-GO:0009507,hydrolase	242.17271	-3.931282137	0.64804318	-6.066389144	1.31E-09	1.17E-07
PITA_000085160	High quality	AT5G10370	MA_10436572g0010	shade>sun	helicase domain-containing protein / IBR domain-containing protein / zinc fin		intracellular anatomical structure-G	81.016852	23.32680868	4.3285545	5.389052785	7.08E-08	3.53E-06
PITA_000085305	High quality	AT5G42190	MA_10428641g0010	shade<sun	Similar to SKP1 in yeast and humans whic	(SKP1B)	cullin family protein binding-GO:00	17.516739	-20.95349401	4.41016289	-4.751183692	2.02E-06	4.57E-05
PITA_000085516	High quality	AT5G47910	MA_107643g0010	shade<sun	NADPH/respiratory burst oxidase protein	RESPIRATORY BURST OXIDASE HON	calcium ion binding-GO:0005509,ca	101.04253	-0.994101062	0.2924333	-3.399411269	0.000675311	0.009054116
PITA_000085662	High quality	AT3G26060	MA_10197498g0010	shade<sun	encodes periredoxin Q which decompose	PEROXIREDOXIN Q (PRXQ)	antioxidant activity-GO:0016209,ce	1125.9367	-1.288966279	0.22215975	-5.801979329	6.55E-09	4.86E-07
PITA_000085701	High quality	AT1G68130	MA_92989g0010	shade>sun	Encodes the longer of two splice variants	INDETERMINATE(ID)-DOMAIN 14 (	DNA-binding transcription factor ac	42.138416	0.917158638	0.31260858	2.933888249	0.003347447	0.035451375
PITA_000085734	High quality	AT5G51980	MA_10432505g0010	shade>sun	Transducin/WD40 repeat-like superfamily protein;(source:Araport11)		biological_process_unknown-GO:0	56.147989	1.762399896	0.6123118	2.878271985	0.003998602	0.040860418
PITA_000085776	High quality	AT2G31955	MA_10433768g0010	shade<sun	COFACTOR OF NITRATE REDUCTASE AND	COFACTOR OF NITRATE REDUCTAS	4 iron, 4 sulfur cluster binding-GO:(	21.020087	-21.19302767	4.40970434	-4.805997415	1.54E-06	3.72E-05
PITA_000085827	Low quality	AT5G64260	MA_10425915g0010	shade>sun	EXORDIUM like 2;(source:Araport11)	EXORDIUM LIKE 2 (EXL2)	cell wall-GO:0005618,cellular respo	15.056325	2.628541045	0.79896786	3.289920892	0.001002156	0.012668962
PITA_000085879	Low quality	AT5G48130	MA_14938g0020	shade<sun	Phototropic-responsive NPH3 family protein;(source:Araport11)		protein ubiquitination-GO:0016567	30.357159	-21.69383454	4.40899933	-4.920353335	8.64E-07	2.39E-05
PITA_000085909	High quality	AT5G10750	MA_689126g0010	shade<sun	enhanced disease resistance-like protein (DUF1336);(source:Araport11)		biological_process_unknown-GO:0	156.31415	-1.957381242	0.5616354	-3.4851458	0.000491869	0.006841814
PITA_000086167	Low quality	AT3G49070	MA_74847g0010	shade<sun	transmembrane protein, putative (DUF677);(source:Araport11)		chloroplast-GO:0009507	16.061281	-2.03098247	0.6360359	-3.193188421	0.001407111	0.017061221
PITA_000086212	Low quality	AT3G57300	MA_3312g0010	shade>sun	Encodes the Arabidopsis INO80 ortholog	INO80 ORTHOLOG (INO80)	3'-5' DNA helicase activity-GO:0043	70.691984	1.185986147	0.30244414	3.921339429	8.81E-05	0.001478683
PITA_000086371	Low quality	AT5G01110	MA_10430089g0010	shade<sun	Tetratricopeptide repeat (TPR)-like superfamily protein;(source:Araport11)		mitochondrion-GO:0005739	505.81192	-0.797909878	0.23504187	-3.39475627	0.000686897	0.009175829
PITA_000086385	High quality	AT3G51300	MA_184760g0010	shade<sun	Encodes a pollen-specific Rop GTPase, me	RHO-RELATED PROTEIN FROM PLA	actin filament organization-GO:000	166.88461	-1.03875728	0.32575434	-3.188774958	0.001428771	0.017299182
PITA_000086525	High quality	AT5G15310	MA_9483804g0010	shade>sun	Member of the R2R3 factor gene family.	MYB DOMAIN PROTEIN 16 (MYB16	cell morphogenesis-GO:0000902,cu	24.255273	2.079841014	0.57472157	3.618867152	0.000295895	0.00437488
PITA_000086616	Low quality	AT3G15095	MA_115155g0010	shade<sun	Encodes HCF243 (high chlorophyll fluores	HIGH CHLOROPHYLL FLUORESCENC	chloroplast-GO:0009507,nucleus-G	60.714318	-22.53151824	4.4082046	-5.11126871	3.20E-07	1.18E-05
PITA_000086631	Low quality	AT5G15350	MA_10432284g0020	shade>sun	early nodulin-like protein 17;(source:Arap	EARLY NODULIN-LIKE PROTEIN 17 (	anchored component of membrane	57.260475	9.582965772	1.33675526	7.168825926	7.56E-13	1.45E-10
PITA_000086876	Low quality	AT1G27180	MA_100966g0020	shade>sun	disease resistance protein (TIR-NBS-LRR class);(source:Araport11)		ADP binding-GO:0043531,cytoplas	74.18114	9.956731936	1.93052368	5.157529031	2.50E-07	1.03E-05
PITA_000086977	High quality	AT4G05440	MA_20748g0010	shade<sun	Plays a role in pollen development and m	EMBRYO SAC DEVELOPMENT ARRE	cell division site-GO:0032153,cytop	60.714318	-22.53151824	4.4082046	-5.11126871	3.20E-07	1.18E-05
PITA_000087198	High quality	AT4G04630	MA_93306g0010	shade>sun	senescence regulator (Protein of unknown function, DUF584);(source:Araport		biological_process_unknown-GO:0	9.5522709	4.019538636	0.95119072	4.225796718	2.38E-05	0.000444078
PITA_000087211	Low quality	AT5G07860	MA_102961g0010	shade<sun	HXXXD-type acyl-transferase family protein;(source:Araport11)		cytoplasm-GO:0005737,transferase	45.535739	-22.24090834	4.40846951	-5.045040755	4.53E-07	1.46E-05
PITA_000087226	Low quality	AT3G43660	MA_8280g0010	shade>sun	The gene encodes a putative nodulin-like21 protein.		cellular manganese ion homeostasi	53.235094	1.840569258	0.52755102	3.488893397	0.000485025	0.006768669
PITA_000087249	High quality	AT5G45290	MA_104063g0010	shade>sun	RING/U-box superfamily protein;(source:Araport11)		nucleus-GO:0005634,zinc ion bindi	165.28294	11.11244557	2.20039682	5.050200702	4.41E-07	1.43E-05
PITA_000087333	High quality	AT4G39230	MA_10427035g0010	shade<sun	encodes a protein whose sequence is sim	PHENYLCOUMARAN BENZYLIC ETH	cytoplasm-GO:0005737,lignan bios	9285.651	-1.940341319	0.54022581	-3.59172272	0.000328499	0.00479875
PITA_000087361	High quality	AT4G27220	MA_10052798g0010	shade>sun	NB-ARC domain-containing disease resistance protein;(source:Araport11)		ADP binding-GO:0043531,mitochoi	216.04494	24.71425455	4.32820048	5.710053092	1.13E-08	7.81E-07
PITA_000087391	Low quality	AT2G19130	MA_120873g0010	shade<sun	S-locus lectin protein kinase family protein;(source:Araport11)		calmodulin binding-GO:0005516,ex	17.679381	-20.92872422	4.41014042	-4.745591347	2.08E-06	4.59E-05
PITA_000087736	High quality	AT2G36970	MA_10223011g0010	shade<sun	UDP-Glycosyltransferase superfamily protein;(source:Araport11)		transferase activity, transferring gly	148.86026	-1.089474155	0.25128079	-4.335684227	1.45E-05	0.000278938
PITA_000087745	High quality	AT1G27170	MA_89418g0010	shade>sun	transmembrane receptors / ATP binding protein;(source:Araport11)		ADP binding-GO:0043531,cytoplas	99.905232	0.962135779	0.32903282	2.924133173	0.003454169	0.036415961
PITA_000087851	Low quality	AT2G30370	MA_22430g0010	shade<sun	Encodes a small, potentially secreted prot	CHALLAH (CHAL)	guard cell differentiation-GO:00100	158.39854	-0.871730774	0.29504249	-2.954594058	0.003130807	0.033686431
PITA_000087852	High quality	AT1G15520	MA_10425791g0020	shade>sun	ABC transporter family involved in ABA tr	ATP-BINDING CASSETTE G40 (ABCC	abscisic acid transport-GO:008016	58.343556	9.609934207	1.82769934	5.257940409	1.46E-07	6.75E-06
PITA_000087955	Low quality	AT1G14980	MA_10429427g0010	shade<sun	Encodes mitochondrial-localized chaperoi	CHAPERONIN 10 (CPN10)	ATP binding-GO:0005524,chaperor	118.57266	-10.13910762	1.92037699	-5.279748533	1.29E-07	6.07E-06
PITA_000088023	High quality	AT5G50900	MA_10427334g0010	shade>sun	ARM repeat superfamily protein;(source:Araport11)		biological_process_unknown-GO:0	184.81365	1.011690864	0.26775279	3.778451187	0.000157807	0.002516261
PITA_000088185	Low quality	AT1G04950	MA_10433952g0010	shade<sun	Encodes one of two Arabidopsis proteins	TATA BOX ASSOCIATED FACTOR II !	histone acetylation-GO:0016573,nu	259.75081	-0.843773609	0.18956537	-4.451095839	8.54E-06	0.000170387
PITA_000088262	Low quality	AT2G34510	MA_10433113g0010	shade<sun	Protein of unknown function, DUF642. Fo	(ATHB-1)	anchored component of membrane	329.18751	-1.627952452	0.25675348	-6.340527243	2.29E-10	2.50E-08
PITA_000088265	High quality	AT4G36470	MA_10433097g0010	shade<sun	S-adenosyl-L-methionine-dependent methyltransferases superfamily protein;(		methylation-GO:0032259,nucleus-(	45.995515	-8.769466674	1.48795542	-5.893635369	3.78E-09	3.06E-07
PITA_000088342	High quality	AT3G50950	MA_10297274g0010	shade<sun	Encodes a canonical CC-type NLR protein	HOPZ-ACTIVATED RESISTANCE 1 (Z	ADP binding-GO:0043531,defense	70.717523	-22.84103454	4.40809223	-5.181614484	2.20E-07	9.17E-06
PITA_000088455	Low quality	AT1G48175	MA_9503281g0010	shade>sun	Cytidine/deoxycytidylate deaminase fami	EMBRYO DEFECTIVE 2191 (emb219	cytoplasm-GO:0005737,embryo de	60.855967	0.81911475	0.28872282	2.837028075	0.004553561	0.045656846
PITA_000088592	High quality	AT2G36380	MA_10426781g0010	shade<sun	pleiotropic drug resistance 6;(source:Arap	ATP-BINDING CASSETTE G34 (ABCC	ATPase-coupled transmembrane tr	23.572508	-21.35301597	4.4094576	-4.84254933	1.28E-06	3.20E-05
PITA_000088610	High quality	AT1G75350	MA_10430444g0010	shade<sun	Ribosomal protein L31;(source:Araport11	EMBRYO DEFECTIVE 2184 (emb218	chloroplast-GO:0009507,chloropla	277.5872	-0.645267962	0.17793523	-3.626420401	0.000287377	0.004271131
PITA_000088703	High quality	AT1G80920	MA_10431645g0020	shade>sun	A nuclear encoded soluble protein found i	(J8)	chloroplast-GO:0009507,chloropla	27.027637	2.197438848	0.72272117	3.040507099	0.002361801	0.02661613
PITA_000088727	High quality	AT5G05700	MA_221777g0010	shade<sun	Encodes an arginyl-tRNA:protein transfer	ARGININE-TRNA PROTEIN TRANSFE	arginyltransferase activity-GO:0004	116.59894	-10.11588036	1.58860067	-6.367793099	1.92E-10	2.11E-08
PITA_000088760	High quality	AT4G22010	MA_10432190g0020	shade>sun	SKU5 similar 4;(source:Araport11)	SKU5 SIMILAR 4 (sks4)	copper ion binding-GO:0005507,ex	108.02247	23.75154617	4.32841291	5.487356833	4.08E-08	2.16E-06
PITA_000088884	High quality	AT3G48700	MA_10429351g0010	shade>sun	carboxyesterase 13;(source:Araport11)	CARBOXYESTERASE 13 (CXE13)	cytosol-GO:0005829,hydrolase acti	41.430351	1.102158162	0.39144331	2.815626546	0.004868222	0.048170568
PITA_000088967	High quality	AT1G10630	MA_24477g0010	shade<sun	A member of ARF GTPase family. A thalia	ADP-RIBOSYLATION FACTOR A1F (/	copper ion binding-GO:0005507,cy	36.428591	-21.93892968	4.40873446	-4.976242023	6.48E-07	2.01E-05
PITA_000088969	High quality	AT5G47470	MA_61847g0010	shade<sun	nodulin MtN21-like transporter family pr	USUALLY MULTIPLE ACIDS MOVE II	plasma membrane-GO:0005886,tr	616.55922	-1.684510845	0.55099277	-3.057228579	0.002233939	0.025346344
PITA_000089008	Low quality	AT3G50150	MA_10154577g0010	shade>sun	transmembrane protein, putative (DUF247);(source:Araport11)		biological_process_unknown-GO:0	59.440863	2.071069429	0.61319832	3.377487137	0.000731514	0.009651007
PITA_000089069	High quality	AT1G69040	MA_10143217g0010	shade<sun	ACT-domain containing protein involved i	ACT DOMAIN REPEAT 4 (ACR4)	chloroplast-GO:0009507,cytosol-G	52.550218	-22.4390271	4.4083279	-5.09014474	3.58E-07	1.19E-05
PITA_000089114	High quality	AT3G42960	MA_107740g0010	shade>sun	Arabidopsis homolog of TASSELSEED2. Ex	TAPETUM 1 (ATA1)	cellular_component_unknown-GO:	121.01943	23.90838197	4.3283673	5.52364906	3.32E-08	1.87E-06
PITA_000089399	High quality	AT4G38540	MA_441480g0010	shade<sun	FAD/NAD(P)-binding oxidoreductase family protein;(source:Araport11)		FAD binding-GO:0071949,mitochor	140.61346	-0.979790371	0.30173605	-3.24717702	0.001165559	0.014433089
PITA_000089670	Low quality	AT5G17460	MA_10432236g0010	shade<sun	glutamyl-tRNA (Gln) amidotransferase su	DROUGHT AND FREEZING RESPON	mitochondrion-GO:0005739,nucleu	123.39432	-0.594321417	0.20721507	-2.868137938	0.004128955	0.042048116
PITA_000089869	Low quality	AT2G02240	MA_22084g0010	shade>sun	F-box family protein;(source:Araport11)	MATERNAL EFFECT EMBRYO ARRE!	chloroplast-GO:0009507,embryo d	121.01943	23.90838197	4.3283673	5.52364906	3.32E-08	1.87E-06
PITA_000090093	High quality	AT3G02830	MA_1139g0010	shade<sun	Encodes a zinc finger protein that binds tc	ZINC FINGER PROTEIN 1 (ZFN1)	cytosol-GO:0005829,DNA binding-(	125.74374	-0.748682745	0.25303954	-2.95875797	0.003088816	0.033318617
PITA_000090268	High quality	AT1G19900	MA_10429735g0020	shade>sun	RUBY encodes a secreted galactose oxida	RUBY PARTICLES IN MUCILAGE (RU	cell wall modification-GO:0042545,	33.706437	8.818819347	1.78968478	4.927582458	8.33E-07	2.38E-05
PITA_000090286	Low quality	AT4G06634	MA_10428468g0030	shade<sun	Encodes an ABA responsive C2H2-type zir	YIN YANG 1 (YY1)	DNA-binding transcription factor ac	352.54081	-0.86560949	0.27212446	-3.180932366	0.001468019	0.017674203
PITA_000090487	Low quality	AT3G03150	MA_10436059g0010	shade<sun	hypothetical protein;(source:Araport11)		biological_process_unknown-GO:0	137.50365	-1.167158183	0.21584792	-5.407317268	6.40E-08	3.25E-06
PITA_000090588	High quality	AT1G75490	MA_904750g0010	shade<sun	dehydration-responsive element-binding 2D-like		transcription cis-regulatory region b	26.275109	-21.49994244	4.40924566	-4.876104457	1.08E-06	2.80E-05
PITA_000090739	High quality	AT3G51895	MA_8073g0010	shade<sun	Encodes a chloroplast-localized sulfate tra	SULFATE TRANSPORTER 3;1 (SULTF	anion:anion antiporter activity-GO:	40.476212	-21.86659121	4.40860183	-4.959983247	7.05E-07	2.04E-05
PITA_000090763	High quality	AT5G13930	MA_10426264g0020	shade<sun	Encodes chalcone synthase (CHS), a key e	TRANSPARENT TESTA 4 (TT4)	auxin polar transport-GO:0009926,	58.720253	-2.665935029	0.70089576	-3.803611303	0.000142602	0.002296697
PITA_000091033	High quality	AT1G67720	MA_2906g0010	shade<sun	Leucine-rich repeat protein kinase family protein;(source:Araport11)		kinase activity-GO:0016301,phosph	93.433743	-1.072463077	0.32507137	-3.299161911	0.00096974	0.01233636
PITA_000091053	Low quality	AT5G58360	MA_10434319g0010	shade<sun	ovate family protein 3;(source:Araport11)	OVATE FAMILY PROTEIN 3 (OFP3)	DNA binding-GO:0003677,negative	391.7684	-0.73807366	0.19961105	-3.697559192	0.000217682	0.003366191
PITA_000091181	Low quality	AT5G36970	MA_722404g0010	shade<sun	NDR1/HIN1-like protein, expression induc	NDR1/HIN1-LIKE 25 (NHL25)	defense response to other organisn	30.357159	-21.69383454	4.40899933	-4.920353335	8.64E-07	2.39E-05
PITA_000091267	Low quality	AT5G48810	MA_15972g0010	shade<sun	Encodes a cytochrome b5 isoform that lo	CYTOCHROME B5 ISOFORM D (CB5	defense response to bacterium-GO	399.003	-1.931790451	0.32546894	-5.935406465	2.93E-09	2.42E-07
PITA_000091387	Low quality	AT2G44270	MA_10434783g0010	shade>sun	cytoplasmic tRNA 2-thiolation 1 isoform X1		cytosolic tRNA wobble base thiouri	108.02247	23.75154617	4.32841291	5.487356833	4.08E-08	2.16E-06
PITA_000091420	High quality	AT4G14320	MA_10427098g0010	shade<sun	Zinc-binding ribosomal protein family pro	RPL36A B (RPL36AB)	cytoplasm-GO:0005737,cytosol-GC	95.361631	-0.816361221	0.24761235	-3.296932609	0.00097747	0.01241619
PITA_000091450	High quality	AT5G01410	MA_10426564g0010	shade<sun	Encodes a protein predicted to function ir	REDUCED SUGAR RESPONSE 4 (RSF	amine-lyase activity-GO:0016843,c	438.99812	-1.33788988	0.28899571	-4.629445533	3.67E-06	7.75E-05
PITA_000091481	High quality	AT2G17040	MA_101849g0010	shade>sun	Member of the NAC transcription factor f	NAC DOMAIN CONTAINING PROTE	DNA binding-GO:0003677,DNA-bin	175.03067	11.1950013	1.71137441	6.541526646	6.09E-11	7.43E-09

PITA_000091747	Low quality	AT1G34300	MA_132034g0010	shade>sun	lectin protein kinase family protein;(source:Araport11)		calmodulin binding-GO:0005516,ex	121.01943	23.90838197	4.3283673	5.52364906	3.32E-08	1.87E-06
PITA_000091837	High quality	AT2G45570	MA_493501g0010	shade<sun	member of CYP76C	CYTOCHROME P450, FAMILY 76, SI	heme binding-GO:0020037,iron ior	1149.0644	-3.214265263	0.4110347	-7.819936547	5.28E-15	1.54E-12
PITA_000091893	High quality	AT3G59420	MA_96803g0010	shade>sun	Encodes a membrane localized protein wi	CRINKLY4 (CR4)	cell surface-GO:0009986,embryo d	93.022936	0.760770823	0.27128163	2.804358	0.005041689	0.049441069
PITA_000091975	High quality	AT2G40000	MA_10429454g0010	shade>sun	ortholog of sugar beet HS1 PRO-1 2;(sour	ORTHOLOG OF SUGAR BEET HS1 PI	cellular response to hypoxia-GO:00	121.01943	23.90838197	4.3283673	5.52364906	3.32E-08	1.87E-06
PITA_000092076	High quality	AT3G46440	MA_174294g0010	shade<sun	encodes a protein similar to UDP-glucuroı	UDP-XYL SYNTHASE 5 (UXS5)	chloroplast-GO:0009507,cytoplasm	18.398206	-7.445949344	1.51491413	-4.915096639	8.87E-07	2.43E-05
PITA_000092077	High quality	AT5G57550	MA_11177g0010	shade>sun	xyloglucan endotransglycosylase-related ı	XYLOGLUCAN ENDOTRANSGLUCOS	extracellular region-GO:0005576,hy	12.82741	7.424990252	2.07036291	3.58632306	0.000335373	0.004890799
PITA_000092078	High quality	AT1G28440	MA_113767g0010	shade<sun	HAESA-like 1;(source:Araport11)	HAESA-LIKE 1 (HSL1)	kinase activity-GO:0016301,phosph	30.357159	-21.69383454	4.40899933	-4.920353335	8.64E-07	2.39E-05
PITA_000092255	High quality	AT5G10240	MA_10428957g0010	shade>sun	Encodes asparagine synthetase (ASN3).	ASPARAGINE SYNTHETASE 3 (ASN3	asparagine biosynthetic process-GC	15.15483	1.929011486	0.67869368	2.842241718	0.004479751	0.045111078
PITA_000092372	High quality	AT4G30825	MA_187850g0010	shade<sun	P-class pentatricopeptide repeat (PPR) pro	BIOGENESIS FACTOR REQUIRED FC	chloroplast stroma-GO:0009570,DI	687.6605	-1.185305486	0.20368097	-5.81942196	5.91E-09	4.49E-07
PITA_000092461	High quality	AT1G80490	MA_10436445g0020	shade<sun	Encodes a protein with a Lissen-cephaly ty	TOPLESS-RELATED 1 (TPR1)	cytoplasm-GO:0005737,cytosol-GC	52.550218	-22.4390271	4.4083279	-5.09014474	3.58E-07	1.19E-05
PITA_000092506	High quality	AT2G40000	MA_98236g0010	shade<sun	ortholog of sugar beet HS1 PRO-1 2;(sour	ORTHOLOG OF SUGAR BEET HS1 PI	cellular response to hypoxia-GO:00	28.287009	-21.59902252	4.40911624	-4.898719231	9.65E-07	2.61E-05
PITA_000092603	High quality	AT1G53540	MA_10291578g0010	shade>sun	Member of the class I small heat-shock pr	(HSP17.6C)	cytoplasm-GO:0005737,protein coı	87.864413	2.935897889	0.97452879	3.012633305	0.002589917	0.028748411
PITA_000093079	High quality	AT1G51990	MA_10432299g0010	shade<sun	O-methyltransferase family protein;(source:Araport11)		aromatic compound biosynthetic p	60.714318	-22.53151824	4.4082046	-5.11126871	3.20E-07	1.18E-05
PITA_000093189	High quality	AT1G51060	MA_10431879g0010	shade>sun	Encodes HTA10, a histone H2A protein. Tl	HISTONE H2A 10 (HTA10)	cell wall-GO:0005618,chromatin sil	413.02396	0.902953815	0.2323082	3.886878778	0.000101541	0.001686299
PITA_000093316	Low quality	AT5G64930	MA_10429308g0010	shade>sun	Regulator of expression of pathogenesis-r	CONSTITUTIVE EXPRESSION OF PR	aging-GO:0007568,chloroplast-GO	114.85553	1.537153393	0.46703875	3.291275924	0.00099734	0.012624761
PITA_000093339	Low quality	AT1G48950	MA_10437121g0010	shade>sun	C3HC zinc finger-like protein;(source:Arap	(MEM1)	biological_process_unknown-GO:0(	23.035588	8.269078721	2.42738118	3.406584347	0.000657812	0.008865924
PITA_000093379	High quality	AT5G16370	MA_10436072g0030	shade>sun	acyl activating enzyme 5;(source:Araport1	ACYL ACTIVATING ENZYME 5 (AAE5	chloroplast-GO:0009507,peroxisom	14.505987	7.602105643	1.94009003	3.918429313	8.91E-05	0.001495666
PITA_000093495	High quality	AT3G25950	MA_10434585g0020	shade<sun	TRAM, LAG1 and CLN8 (TLC) lipid-sensing domain containing protein;(source:		biological_process_unknown-GO:0(	20.238106	-21.14561367	4.4097939	-4.795147836	1.63E-06	3.81E-05
PITA_000093533	High quality	AT4G22820	MA_211897g0010	shade<sun	A member of the A20/AN1 zinc finger prc	STRESS ASSOCIATED PROTEIN 9 (SA	defense response to other organisn	65.706538	-9.286175864	1.46689095	-6.330515483	2.44E-10	2.64E-08
PITA_000093588	Low quality	AT5G45190	MA_10111260g0010	shade<sun	Encodes a cyclin T partner CYCT1;5. Plays important roles in infection with Ca		cyclin/CDK positive transcription ele	35.358761	-21.90137695	4.40877489	-4.967678662	6.78E-07	2.02E-05
PITA_000093718	Low quality	AT5G15290	MA_93690g0010	shade<sun	Uncharacterized protein family (UPF0497	CASPARIAN STRIP MEMBRANE DOI	4 iron, 4 sulfur cluster binding-GO:(	47.145015	-22.29213208	4.40843355	-5.056701391	4.27E-07	1.38E-05
PITA_000093888	High quality	AT3G27970	MA_8332g0010	shade<sun	Exonuclease family protein;(source:Araport11)		3'-5' exonuclease activity-GO:0008	236.12864	-2.923910518	0.46069111	-6.346791732	2.20E-10	2.41E-08
PITA_000093955	High quality	AT4G31940	MA_10055697g0010	shade<sun	The gene encodes a cytochrome P450 enz	CYTOCHROME P450, FAMILY 82, SI	cellular response to iron ion-GO:00	1951.4116	-3.888380723	0.317262	-12.2560557	1.56E-34	4.44E-31
PITA_000094182	High quality	AT2G31970	MA_10429947g0010	shade>sun	Encodes the Arabidopsis RAD50 homolog	(RAD50)	chromosome organization involved	40.919702	1.04778478	0.31184054	3.360001856	0.000779419	0.010198965
PITA_000094398	Low quality	AT2G16860	MA_90172g0010	shade>sun	GCIP-interacting family protein;(source:Ar	(ATSYF2)	catalytic step 2 spliceosome-GO:00	121.01943	23.90838197	4.3283673	5.52364906	3.32E-08	1.87E-06
PITA_000094446	Low quality	AT4G35790	MA_161943g0010	shade<sun	Encodes a protein with phospholipase D a	PHOSPHOLIPASE D DELTA (PLDDEL	calcium ion binding-GO:0005509,m	107.15462	-0.815597828	0.22970887	-3.550571767	0.000384395	0.005523903
PITA_000094605	Low quality	AT1G62020	MA_10429054g0010	shade>sun	Member of the Coat Protein I (COPI) com	α1 COAT PROTEIN (α1-	COPI vesicle coat-GO:0030126,Cul4	78.513461	10.03834398	2.08477203	4.815079944	1.47E-06	3.59E-05
PITA_000094653	High quality	AT5G09590	MA_101964g0010	shade<sun	heat shock protein 70 (Hsc70-5); nuclear	MITOCHONDRIAL HSO70 2 (MTHS(	ATP binding-GO:0005524,ATPase a	60.714318	-22.53151824	4.4082046	-5.11126871	3.20E-07	1.18E-05
PITA_000094894	High quality	AT1G74550	MA_10434848g0020	shade<sun	Encodes a tricoumaroylspermidine meta-	CYTOCHROME P450, FAMILY 98, SI	chloroplast-GO:0009507,dicoumar	235.31982	-1.145415233	0.15473501	-7.402430954	1.34E-13	3.08E-11
PITA_000094953	High quality	AT5G08380	MA_96852g0010	shade<sun	alpha-galactosidase 1;(source:Araport11)	ALPHA-GALACTOSIDASE 1 (AGAL1)	apoplast-GO:0048046,carbohydrat	30.357159	-21.69383454	4.40899933	-4.920353335	8.64E-07	2.39E-05
PITA_000095300	Low quality	AT1G15780	MA_10425757g0010	shade>sun	mediator of RNA polymerase II transcripti	NON-RECOGNITION-OF-BTH 4 (NRI	chromatin DNA binding-GO:003149	25.391708	1.363477304	0.485822	2.806536748	0.00500772	0.049208632
PITA_000095314	High quality	AT3G22060	MA_125752g0010	shade<sun	contains Pfam profile: PF01657 Domain of unknown function that is usually as		cell wall-GO:0005618,cytosol-GO:0	105.13797	-9.964616811	1.6545818	-6.022438288	1.72E-09	1.48E-07
PITA_000095368	Low quality	AT5G57330	MA_10430032g0010	shade>sun	Galactose mutarotase-like superfamily protein;(source:Araport11)		carbohydrate metabolic process-G(	55.006125	1.211700345	0.41305883	2.933481288	0.003351839	0.035481763
PITA_000095672	Low quality	AT5G06990	MA_33507g0010	shade<sun	MIZU-KUSSEI-like protein (Protein of unknown function, DUF617);(source:Ara		chloroplast-GO:0009507,hydrotrop	1170.8362	-1.339321116	0.42536523	-3.148637959	0.001640333	0.01951935
PITA_000095853	High quality	AT4G16730	MA_10196363g0010	shade<sun	In the Col ecotype, no functional protein i	TERPENE SYNTHASE 02 (TPS02)	chloroplast-GO:0009507,magnesiu	40.476212	-21.86659121	4.40860183	-4.959983247	7.05E-07	2.04E-05
PITA_000095920	High quality	AT4G12320	MA_52987g0020	shade<sun	member of CYP706A	CYTOCHROME P450, FAMILY 706, :	chloroplast-GO:0009507,cytosol-G(	530.44603	-2.328342272	0.49182316	-4.734104532	2.20E-06	4.82E-05
PITA_000096044	High quality	AT2G33380	MA_167502g0010	shade<sun	Encodes a calcium binding protein whose	RESPONSIVE TO DESICCATION 20 (I	(R)-2-hydroxy-alpha-linolenic acid b	138.46276	-0.665605282	0.18839946	-3.53294679	0.000410955	0.005876385
PITA_000096241	High quality	AT3G26125	MA_172404g0020	shade<sun	encodes a protein with cytochrome P450	CYTOCHROME P450, FAMILY 86, SI	heme binding-GO:0020037,iron ior	35.358761	-21.90137695	4.40877489	-4.967678662	6.78E-07	2.02E-05
PITA_000096289	High quality	AT4G02290	MA_16152g0010	shade<sun	glycosyl hydrolase 9B13;(source:Araport1	GLYCOSYL HYDROLASE 9B13 (GH9E	cellulase activity-GO:0008810,extra	197.32228	-1.781389957	0.33345013	-5.342297941	9.18E-08	4.48E-06
PITA_000096297	Low quality	AT5G11180	MA_10128576g0010	shade<sun	member of Putative ligand-gated ion char	GLUTAMATE RECEPTOR 2.6 (GLR2.	calcium channel activity-GO:00052	73.992762	-9.45934361	1.87890564	-5.034496361	4.79E-07	1.53E-05
PITA_000096299	High quality	AT4G18910	MA_10990g0010	shade<sun	Encodes an aquaporin homolog. Function	NOD26-LIKE INTRINSIC PROTEIN 1;	arsenite transmembrane transporte	20.238106	-21.14561367	4.4097939	-4.795147836	1.63E-06	3.81E-05
PITA_000096590	High quality	AT5G50260	MA_103463g0010	shade>sun	Encodes a papain-like cysteine protease ir	CYSTEINE ENDOPEPTIDASE 1 (CEP1	anther wall tapetum development-	108.02247	23.75154617	4.32841291	5.487356833	4.08E-08	2.16E-06

Table S2 Gene expression in response to SHADE in Scots pine at latitude 67

PITA_ID	Confidence	Best BLAST Arabidopsis	Best BLAST Conifer spp	Expression	Gene Model Description	Primary Gene Symbol	GO term-GO ID	baseMean	log2FoldChange	lfcSE	stat	pvalue	padj
PITA_000000020	Low quality	AT2G44920	MA_10232509g0010	shade<sun	Encodes a pentapeptide-repeat protein (PRP) composed c		chloroplast-GO:0009507,c	242.9581902	-0.914539891	0.19912554	-4.592780512	4.37E-06	0.000109215
PITA_000000026	Low quality	AT3G63540	MA_132558g0010	shade<sun	Conceptual translation of this open reading frame gave th		calcium ion binding-GO:00	313.7907004	-0.756595214	0.17268155	-4.381447924	1.18E-05	0.00026714
PITA_000000043	Low quality	AT3G54000	MA_213656g0010	shade<sun	TIP41-like protein;(source:Araport11)		biological_process_unkno	510.4300028	-1.329545331	0.21196508	-6.272473536	3.55E-10	1.79E-08
PITA_000000044	Low quality	AT4G01037	MA_423098g0010	shade<sun	Ubiquitin carboxyl-terminal hy	WHAT'S THIS FACTOR? (W	chloroplast-GO:0009507,e	529.6761118	-1.145155467	0.12430032	-9.212812067	3.18E-20	7.86E-18
PITA_000000051	High quality	AT2G44500	MA_10434460g0020	shade>sun	O-fucosyltransferase family protein;(source:Araport11)		cytoplasm-GO:0005737,nu	33.0321813	2.654465954	0.48343502	5.490843337	4.00E-08	1.44E-06
PITA_000000052	High quality	AT1G15670	MA_19365g0010	shade<sun	Encodes a member of a family	KISS ME DEADLY 2 (KMD2	cytosol-GO:0005829,mole	87.04754399	-0.833116868	0.18231885	-4.569559725	4.89E-06	0.000121202
PITA_000000119	Low quality	AT3G12955	MA_667011g0010	shade>sun	SAUR-like auxin-responsive prc	SMALL AUXIN UPREGULAT	mitochondrion-GO:00057:	10.82755514	4.243357362	0.97480916	4.353013405	1.34E-05	0.000297585
PITA_000000122	Low quality	AT1G53800	MA_495939g0010	shade<sun	muscle M-line assembly protein;(source:Araport11)		biological_process_unkno	364.7948035	-0.783618426	0.11069527	-7.07905988	1.45E-12	1.17E-10
PITA_000000194	High quality	AT3G19940	MA_457005g0010	shade<sun	Encodes a hexose-H(+) sympor	SUGAR TRANSPORT PROT	carbohydrate transmembr	172.1148675	-2.244557338	0.15003326	-14.96039883	1.33E-50	3.36E-47
PITA_000000260	High quality	AT2G38540	MA_7195626g0010	shade>sun	Non-specific lipid transfer prot	LIPID TRANSFER PROTEIN	apoplast-GO:0048046,caln	29.59692165	1.253874813	0.41474135	3.023269339	0.002500596	0.026768743
PITA_000000266	Low quality	AT5G42900	MA_48561g0010	shade<sun	Acts with COR28 as a key regul	COLD REGULATED GENE 2	negative regulation of trar	201.1435299	-0.471409507	0.11766744	-4.006286686	6.17E-05	0.001140461
PITA_000000277	High quality	AT4G17500	MA_166248g0010	shade>sun	Encodes a member of the ERF	ETHYLENE RESPONSIVE EL	cell division-GO:0051301,[	37.53655827	1.854876463	0.35045353	5.292788659	1.20E-07	3.95E-06
PITA_000000291	High quality	AT2G31670	MA_10427036g0020	shade<sun	Stress responsive alpha-beta b	UP3 (UP3)	biological_process_unkno	154.6269	-0.584704779	0.20342675	-2.874276748	0.00404954	0.038906433
PITA_000000295	High quality	AT1G01880	MA_10432278g0010	shade<sun	Encodes one of two GEN1 hom	ORTHOLOG OF HSGEN1 (G	5'-flap endonuclease activ	250.3054978	-1.326952466	0.30451261	-4.357627314	1.31E-05	0.000292708
PITA_000000328	High quality	AT5G43940	MA_10427177g0010	shade>sun	Encodes a glutathione-depend	SENSITIVE TO HOT TEMPE	alcohol dehydrogenase ac	32.74582291	0.886771705	0.2721135	3.258830196	0.001118726	0.013859575
PITA_000000345	High quality	AT5G51050	MA_10433727g0010	shade<sun	Encodes an APC isoform in Ara	ATP/PHOSPHATE CARRIER	ADP transmembrane trans	137.9264373	-0.884999056	0.23951801	-3.694916486	0.000219959	0.003471444
PITA_000000405	High quality	AT1G14030	MA_9247415g0010	shade<sun	Encodes a lysine methyltransfe	LYSINE METHYLTRANSFER	[ribulose-bisphosphate car	125.573666	-0.602253048	0.20387906	-2.95397209	0.003137124	0.031949385
PITA_000000524	Low quality	AT4G30080	MA_2237g0020	shade<sun	Involved in root cap cell differe	AUXIN RESPONSE FACTOR	cell division-GO:0051301,[	159.8983223	-10.95173335	1.32543873	-8.262723186	1.42E-16	2.39E-14
PITA_000000568	High quality	AT3G51740	MA_17859g0010	shade>sun	encodes a leucine-repeat recep	INFLORESCENCE MERISTE	cell wall-GO:0005618,kina:	43.6321928	1.159096749	0.26518853	4.370840444	1.24E-05	0.000278453
PITA_000000598	Low quality	AT5G23680	MA_83723g0010	shade>sun	Sterile alpha motif (SAM) domain-containing protein;(sou		biological_process_unkno	51.0079743	1.020827995	0.23775016	4.293700489	1.76E-05	0.000378773
PITA_000000683	Low quality	AT2G41720	MA_959912g0010	shade<sun	Encodes a pentatricopeptide re	EMBRYO DEFECTIVE 2654	chloroplast-GO:0009507,c	163.1612167	-2.097727391	0.56014139	-3.74499624	0.000180396	0.002920154
PITA_000000708	Low quality	AT2G30370	MA_110556g0010	shade<sun	Encodes a small, potentially se	CHALLAH (CHAL)	guard cell differentiation-(	316.1070619	-0.949993355	0.30147552	-3.151145921	0.001626312	0.0189361
PITA_000000711	High quality	AT3G11050	MA_139907g0010	shade>sun	ferritin 2;(source:Araport11)	FERRITIN 2 (FER2)	chloroplast-GO:0009507,c	79.01422547	1.354912261	0.36878705	3.673969152	0.000238812	0.003736246
PITA_000000754	High quality	AT2G47160	MA_103776g0010	shade<sun	Boron transporter. Protein acc	REQUIRES HIGH BORON 1	active borate transmembr	159.7085817	-0.646127911	0.22493523	-2.872506487	0.004072297	0.03903567
PITA_000000791	High quality	AT5G16780	MA_456501g0010	shade>sun	Encodes a protein belonging tc	DEFECTIVELY ORGANIZED	cotyledon vascular tissue p	64.43834928	0.580364862	0.20805175	2.789521658	0.005278597	0.047477249
PITA_000000819	High quality	AT1G18140	MA_75861g0010	shade>sun	putative laccase, a member of	LACCASE 1 (LAC1)	Casparian strip-GO:00482:	166.1321282	1.451288371	0.36773856	3.946522158	7.93E-05	0.001421329
PITA_000000823	High quality	AT3G19990	MA_107468g0010	shade>sun	E3 ubiquitin-protein ligase;(source:Araport11)		biological_process_unkno	34.23074427	0.893424482	0.32238334	2.771310985	0.005583108	0.049353654
PITA_000000847	High quality	AT5G23960	MA_10426561g0010	shade<sun	Encodes a sesquiterpene synth	TERPENE SYNTHASE 21 (T	(-)-E-beta-caryophyllene s	1136.058721	-2.762353981	0.4676725	-5.906599132	3.49E-09	1.47E-07
PITA_000000910	High quality	AT3G58580	MA_10919g0010	shade>sun	Encodes a protein that is involv	(ATCCR4B)	3'-5'-exoribonuclease activ	29.27510133	0.942162905	0.28654834	3.287971898	0.001009119	0.012705933
PITA_000000937	High quality	AT1G64880	MA_955371g0010	shade<sun	Ribosomal protein S5 family protein;(source:Araport11)		chloroplast-GO:0009507,e	204.1356551	-0.91512535	0.16349547	-5.597251961	2.18E-08	8.16E-07
PITA_000001090	High quality	AT2G39510	MA_10432576g0020	shade>sun	Encodes a plasma membrane-l	USUALLY MULTIPLE ACIDS	chloroplast-GO:0009507,L	70.13143772	1.429066619	0.31963391	4.470948082	7.79E-06	0.000183893
PITA_000001100	Low quality	AT2G13600	MA_58337g0010	shade>sun	Encodes a pentatricopeptide re	SLOW GROWTH 2 (SLO2)	chloroplast-GO:0009507,ir	208.622589	11.00080909	3.90694665	2.815704964	0.004867034	0.044929209
PITA_000001105	High quality	AT3G11670	MA_83793g0010	shade<sun	Responsible for the final assem	DIGALACTOSYL DIACYLGLY	chloroplast-GO:0009507,c	145.2272267	-0.772426519	0.16706919	-4.623393073	3.78E-06	9.51E-05
PITA_000001156	High quality	AT5G54530	MA_36861g0010	shade>sun	serine protease, putative (Protein of unknown function, D		biological_process_unkno	32.73223583	0.805858661	0.27025739	2.981819159	0.002865412	0.02985988
PITA_000001173	Low quality	AT5G54930	MA_104066g0010	shade<sun	AT hook motif-containing prot	METABOLIC NETWORK M	biological_process_unkno	162.7870622	-0.426529331	0.14577723	-2.925898083	0.003434634	0.034351601
PITA_000001175	High quality	AT1G64810	MA_931234g0010	shade<sun	Encodes a chloroplast localizec	ACCUMULATION OF PHOT	chloroplast-GO:0009507,c	128.2336374	-0.715322529	0.17226904	-4.152356781	3.29E-05	0.000660757
PITA_000001216	High quality	AT5G41570	MA_10426942g0010	shade>sun	member of WRKY Transcriptio	WRKY DNA-BINDING PROT	DNA-binding transcription	10.58269252	2.102160726	0.63050771	3.334076173	0.000855832	0.011085818
PITA_000001436	High quality	AT5G03860	MA_120902g0010	shade>sun	Encodes a protein with malate	MALATE SYNTHASE (MLS)	cytoplasm-GO:0005737,gly	12.56454433	1.486338257	0.47380585	3.137019616	0.001706747	0.019661002
PITA_000001445	High quality	AT2G05760	MA_185939g0010	shade>sun	Xanthine/uracil permease fami	NUCLEOBASE ASCORBATE	mitochondrion-GO:00057:	63.47837021	0.589190156	0.19667426	2.995766528	0.002737559	0.028779179
PITA_000001454	High quality	AT4G26270	MA_66347g0010	shade<sun	phosphofructokinase 3;(source	PHOSPHOFRUCTOKINASE	6-phosphofructokinase act	96.3666086	-0.769803816	0.19708816	-3.905885671	9.39E-05	0.001651099
PITA_000001609	High quality	AT5G16460	MA_10431190g0010	shade>sun	Membrane protein involved in	SEIPIN1 (SEIPIN1)	endoplasmic reticulum-GC	57.54548122	0.581109796	0.20464814	2.839555661	0.004517641	0.042276406
PITA_000001737	Low quality	AT1G47750	MA_20641g0020	shade<sun	member of the peroxin11 (PEX	PEROXIN 11A (PEX11A)	identical protein binding-(	236.4581056	-0.650428195	0.19373882	-3.357242498	0.00078724	0.01032997
PITA_000001823	High quality	AT5G16715	MA_374514g0010	shade<sun	protein EMBRYO DEFECTIVE 2:	EMBRYO DEFECTIVE 2247	aminoacyl-tRNA editing ac	448.376075	-2.05781686	0.29219692	-7.042568617	1.89E-12	1.48E-10
PITA_000001839	High quality	AT3G51895	MA_19557g0020	shade<sun	Encodes a chloroplast-localizec	SULFATE TRANSPORTER 3	anion:anion antiporter act	529.7333299	-1.313447609	0.39149805	-3.354927573	0.000793858	0.010405972
PITA_000001891	High quality	AT2G44130	MA_10314385g0010	shade>sun	Encodes a member of a family	KISS ME DEADLY 3 (KMD3	cytoplasm-GO:0005737,cy	22.21332523	1.360106814	0.36458895	3.730521227	0.000191084	0.003075392
PITA_000002028	High quality	AT2G43710	MA_10431660g0010	shade<sun	Encodes a stearoyl-ACP desatu	SUPPRESSOR OF SA INSEN	acyl-[acyl-carrier-protein] (	329.085681	-0.576536497	0.18297754	-3.150859427	0.001627908	0.018937198
PITA_000002065	High quality	AT1G32900	MA_10429460g0010	shade<sun	UDP-Glycosyltransferase super	GRANULE BOUND STARCH	ADP-glucose-starch glucos	204.6700459	-0.977378952	0.18902002	-5.170769472	2.33E-07	7.24E-06
PITA_000002080	High quality	AT4G29400	MA_9871172g0010	shade<sun	oxidoreductase/transition metal	ion-binding protein (DUF	biological_process_unkno	120.9551169	-0.746827398	0.23072394	-3.236887273	0.001208411	0.014772726
PITA_000002124	High quality	AT3G26570	MA_6390g0010	shade<sun	low affinity phosphate transpo	PHOSPHATE TRANSPORTE	chloroplast-GO:0009507,c	257.3881217	-0.699062727	0.19214704	-3.638165543	0.000274587	0.004225187
PITA_000002158	Low quality	AT1G80410	MA_10433512g0010	shade>sun	Encodes the catalytic subunit c	NAA15 (NAA15)	cytoplasm-GO:0005737,cy	38.24926166	8.554335187	1.61339241	5.302079717	1.14E-07	3.78E-06
PITA_000002257	High quality	AT3G61590	MA_922832g0010	shade>sun	F-box protein that is involved i	HAWAIIAN SKIRT (HWS)	extracellular region-GO:00	194.9266517	1.298356098	0.27696758	4.687754798	2.76E-06	7.14E-05
PITA_000002281	High quality	AT4G34830	MA_122602g0010	shade<sun	Encodes MRL1, a conserved pe	MATURATION OF RBCL 1 (	chloroplast-GO:0009507,n	365.9677565	-1.561332516	0.23239925	-6.718319979	1.84E-11	1.18E-09
PITA_000002286	High quality	AT3G28345	MA_3437g0010	shade<sun	Encodes an ATP-binding casset	ATP-BINDING CASSETTE B	ATPase activity-GO:00168	365.1881903	-1.565907053	0.19036526	-8.225802793	1.94E-16	3.18E-14
PITA_000002296	High quality	AT1G62300	MA_161426g0010	shade<sun	Encodes a transcription factor	(WRKY6)	cellular response to boron	374.8741281	-0.542547567	0.17773843	-3.052505724	0.002269394	0.024829836
PITA_000002312	Low quality	AT1G62760	MA_10436535g0030	shade>sun	Pectin methylesterase inhibito	(ATPMEI10)	enzyme inhibitor activity-(	22.40696175	1.831018821	0.41804054	4.380003039	1.19E-05	0.000268676
PITA_000002313	High quality	AT4G38620	MA_19163g0010	shade>sun	Encodes a R2R3 MYB protein w	MYB DOMAIN PROTEIN 4	regulation of phenylpropa	46.41651587	0.999422335	0.23467527	4.258745883	2.06E-05	0.000436051
PITA_000002338	High quality	AT2G02500	MA_10436070g0020	shade<sun	Encodes a protein with 4-Diph	(ISPD)	2-C-methyl-D-erythritol 4-p	180.4302765	-1.176528107	0.16757081	-7.021080313	2.20E-12	1.70E-10
PITA_000002414	High quality	AT1G68930	MA_10426261g0020	shade<sun	pentatricopeptide (PPR) repeat-containing protein;(source		intracellular membrane-bc	154.1140934	-1.34947458	0.45526647	-2.964142232	0.00303528	0.031117785
PITA_000002423	Low quality	AT2G39210	MA_116104g0010	shade<sun	Major facilitator superfamily tr	PICLORAM RESISTANT30 (	chloroplast-GO:0009507,ir	64.99501777	-0.778722956	0.24463714	-3.183175466	0.001456693	0.017304663
PITA_000002494	High quality	AT3G63270	MA_10430920g0010	shade<sun	A mutation in ANTAGONIST OI	ANTAGONIST OF LHP1-1 (	chromatin binding-GO:00C	183.8925839	-0.800216629	0.15268443	-5.240983907	1.60E-07	5.13E-06
PITA_000002536	High quality	AT5G08680	MA_11179g0010	shade<sun	Encodes the mitochondrial ATP synthase beta-subunit. Tl		ATPase activity-GO:00168	623.2752992	-1.599914584	0.45884176	-3.486854758	0.000488737	0.006920797
PITA_000002543	High quality	AT5G06530	MA_10428183g0020	shade>sun	Encodes ABCG22, an ABC trans	ATP-BINDING CASSETTE G	ATPase activity-GO:00168	77.51727937	0.64085046	0.2018178	3.175391218	0.001496346	0.017659265
PITA_000002568	Low quality	AT2G01300	MA_120879g0010	shade<sun	mediator of RNA polymerase II transcription subunit;(sou		biological_process_unkno	244.650595	-0.703113675	0.1812744	-3.87872567	0.000105005	0.001823849
PITA_000002706	High quality	AT4G02360	MA_250036g0010	shade<sun	transmembrane protein, putative (Protein of unknown fu		biological_process_unkno	226.0267088	-1.785023459	0.31095561	-5.740444576	9.44E-09	3.70E-07
PITA_000002709	High quality	AT5G42800	MA_81731g0010	shade>sun	dihydroflavonol reductase. Cat	DIHYDROFLAVONOL 4-REI	anthocyanin-containing co	124.7535165	0.808493852	0.2402791	3.364811435	0.00076596	0.010092742
PITA_000002759	Low quality	AT1G65260	MA_572791g0010	shade<sun	Encodes a protein required for	PLASTID TRANSCRIPTION/	chloroplast-GO:0009507,c	203.6759965	-0.620549414	0.1713874	-3.620741183	0.00029376	0.004481932
PITA_000002781	High quality	AT5G07050	MA_71776g0010	shade<sun	nodulin MtN21-like transporte	USUALLY MULTIPLE ACIDS	chloroplast-GO:0009507,p	3524.581777	-3.345245751	0.60343994	-5.543626686	2.96E-08	1.09E-06
PITA_000002868	High quality	AT3G47470	MA_65346g0010	shade<sun	Encodes a chlorophyll a/b-binc	LIGHT-HARVESTING CHLOI	chlorophyll binding-GO:00	783.141792	-0.730475223	0.18977545	-3.849155528	0.000118526	0.002021108
PITA_000002950	High quality	AT4G33170	MA_8868720g0010	shade<sun	Tetratricopeptide repeat (TPR)-like superfamily protein;(s		chloroplast-GO:0009507,zi	626.2283321	-2.023936776	0.25613504	-7.901834952	2.75E-15	3.57E-13
PITA_000002968	High quality	AT2G21940	MA_10427362g0010	shade<sun	Encodes a shikimate kinase. Its	SHIKIMATE KINASE 1 (SK1	chloroplast-GO:0009507,c	567.7466306	-1.372098085	0.31142841	-4.405821767	1.05E-05	0.000241616
PITA_000002978	High quality	AT4G40030	MA_10294952g0020	shade>sun	Histone superfamily protein;(s	HISTONE 3.3 (H3.3)	DNA binding-GO:0003677,	19.71528339	1.881931262	0.42995514	4.377041018	1.20E-05	0.000271377
PITA_000002990	High quality	AT2G46080	MA_72254g0010	shade>sun	Encodes a protein related to B	BYPASS 2 (BPS2)	embryo development end	27.54935061	2.389391326	0.59401196	4.022463297	5.76E-05	0.00107353
PITA_000003022	High quality	AT4G00030	MA_75636g0010	shade<sun	Plastid-lipid associated protein	FIBRILLIN9 (FBN9)	biological_process_unkno	314.4795992	-0.848890318	0.14266994	-5.950029223	2.68E-09	1.16E-07
PITA_000003023	High quality	AT3G13050	MA_552029g0010	shade<sun	Encodes a plant nicotinate trar	NICOTINATE TRANSPORTE	nicotinate transmembrane	186.8545556	-0.740629391	0.24319898	-3.045363929	0.002323989	0.025285162
PITA_000003054	Low quality	AT4G24090	MA_24252g0010	shade<sun	homer protein;(source:Araport11)		biological_process_unkno	301.5389445	-1.3785002	0.11485291	-12.00230934	3.46E-33	2.72E-30
PITA_000003056	Low quality	AT5G55040	MA_10431815g0010	shade>sun	DNA-binding bromodomain-containing protein;(source:Ar		biological_process_unkno	49.3151987	0.802222246	0.2219254	3.614828428	0.000300547	0.00456449
PITA_000003125	High quality	AT1G74960	MA_10428839g0010	shade<sun	Encodes a plastidic beta-ketoa	FATTY ACID BIOSYNTHESI	3-oxoacyl-[acyl-carrier-pro	191.2228509	-0.914813106	0.16982992	-5.386642647	7.18E-08	2.47E-06
PITA_000003130	High quality	AT5G65690	MA_10429698g0010	shade>sun	Encodes a putative phosphoen	PHOSPHOENOLPYRUVATE	cytoplasm-GO:0005737,cy	82.68277734	2.23870883	0.67673996	3.308078369	0.000939385	0.011977399
PITA_000003240	Low quality	AT2G42975	MA_163154g0020	shade<sun	myosin-G heavy chain-like protein;(source:Araport11)		biological_process_unkno	690.8825983	-1.423850545	0.11161424	-12.7568902	2.85E-37	3.43E-34
PITA_000003270	High quality	AT3G10910	MA_124750g0010	shade>sun	RING/U-box superfamily protei	DAF-LIKE GENE 1 (DAFL1)	nucleus-GO:0005634,prote	32.63538936	1.148872567	0.30830353	3.726433349	0.000194208	0.003115735
PITA_000003316	Low quality	AT5G46800	MA_10428907g0020	shade<sun	Seedling lethal mutation; Mito	A BOUT DE SOUFFLE (BOU	chloroplast-GO:0009507,L	231.7387997	-0.843119536	0.11791358	-7.150317592	8.66E-13	7.40E-11
PITA_000003331	High quality	AT1G53830	MA_84680g0010	shade<sun	encodes a pectin methylestera	PECTIN METHYLESTERASE	callus formation-GO:1990:	711.5123633	-1.063926594	0.33242523	-3.200498887	0.001371899	0.01645235
PITA_000003360	Low quality	AT5G11480	MA_311787g0010	shade<sun	P-loop containing nucleoside tı	(ENGB-2)	nucleus-GO:0005634	206.8139981	-0.858403479	0.30725781	-2.793756447	0.00520997	0.047001515
PITA_000003424	High quality	AT1G31930	MA_10432399g0010	shade>sun	Encodes XLG3 (extra-large G pı	EXTRA-LARGE GTP-BINDIN	adenylate cyclase-modulat	27.084314	8.055099311	1.35623266	5.939319661	2.86E-09	1.23E-07
PITA_000003453	High quality	AT2G37220	MA_320479g0010	shade<sun	Encodes a chloroplast RNA binding protein. A substrate o		chloroplast-GO:0009507,c	966.8679825	-1.248709327	0.09661326	-12.92482302	3.26E-38	4.57E-35
PITA_000003520	High quality	AT1G08230	MA_103912g0010	shade<sun	Codes for a H+-driven, high affinity gamma-aminobutyric a		gamma-aminobutyric acid	1531.007285	-1.223688494	0.33014208	-3.706551104	0.000210101	0.003340948
PITA_000003611	High quality	AT3G52960	MA_61158g0010	shade<sun	Thioredoxin superfamily prote	PEROXIREDOXIN-II-E (PRX	cell redox homeostasis-GC	344.3325991	-1.54352146	0.12994318	-11.87843405	1.53E-32	1.17E-29
PITA_000003632	High quality	AT2G32230	MA_10050138g0010	shade<sun	Encodes a protein-only RNase	PROTEINACEOUS RNASE F	chloroplast-GO:0009507,n	460.585009	-1.643153608	0.28227703	-5.821067391	5.85E-09	2.39E-07
PITA_000003671	Low quality	AT3G50170	MA_109716g0010	shade<sun	transmembrane protein, putative (DUF247);(source:Arapc		biological_process_unkno	709.9538402	-2.190778772	0.30228263	-7.24745169	4.25E-13	3.81E-11
PITA_000003817	Low quality	AT3G23700	MA_37826g0010	shade<sun	Encodes a chloroplast-localizec	S1 RNA-BINDING RIBOSON	chloroplast-GO:0009507,c	406.5395825	-1.111140056	0.12497047	-8.891220671	6.04E-19	1.36E-16
PITA_000003860	High quality	AT5G42510	MA_46983g0010	shade<sun	Disease resistance-responsive (dirigent-like protein) famil		extracellular region-GO:00	244.7156112	-0.775629041	0.1905149	-4.071225098	4.68E-05	0.000901722
PITA_000003862	High quality	AT2G39730	MA_10433855g0010	shade<sun	Rubisco activase, a nuclear-enc	RUBISCO ACTIVASE (RCA)	ADP binding-GO:0043531,	553.1949545	-1.139542657	0.13007505	-8.760655191	1.94E-18	4.11E-16
PITA_000003882	High quality	AT1G70940	MA_100472g0010	shade>sun	A regulator of auxin efflux and	PIN-FORMED 3 (PIN3)	auxin efflux-GO:0010315,a	33.0785423	0.758071094	0.26390935	2.872467711	0.004072797	0.03903567
PITA_000003887	High quality	AT5G39530	MA_96307g0010	shade<sun	hypothetical protein (DUF1997);(source:Araport11)		biological_process_unkno	396.9121895	-1.145438268	0.22629153	-5.061781471	4.15E-07	1.24E-05
PITA_000003897	High quality	AT5G33406	MA_9990713g0010	shade<sun	hAT dimerization domain-containing protein / transposas		biological_process_unkno	341.2544723	-1.134272803	0.09623054	-11.78703606	4.55E-32	3.38E-29
PITA_000004029	High quality	AT2G18790	MA_10435530g0010	shade>sun	Red/far-red photoreceptor inv	PHYTOCHROME B (PHYB)	abscisic acid metabolic prc	58.65310565	0.579068371	0.20259182	2.858300819	0.004259164	0.040452002
PITA_000004048	High quality	AT1G22700	MA_133089g0010	shade<sun	Encodes a TPR protein with ho	(PYG7)	chloroplast-GO:0009507,c	346.9664187	-1.218506615	0.1003914	-12.13755939	6.68E-34	5.43E-31
PITA_000004051	High quality	AT1G21710	MA_929218g0010	shade<sun	Encodes 8-oxoguanine-DNA gly	8-OXOGUANINE-DNA GLY(	8-oxo-7,8-dihydroguanine	114.8897655	-0.52953015	0.17954368	-2.949310982	0.003184833	0.032309531
PITA_000004058	High quality	AT4G24380	MA_27893g0010	shade<sun	dihydrofolate reductase;(source:Araport11)		chloroplast-GO:0009507,c	97.27738713	-0.924959046	0.26074977	-3.547305372	0.000389193	0.00570003
PITA_000004067	High quality	AT5G64570	MA_77922g0010	shade<sun	Encodes a beta-d-xylosidase th	BETA-D-XYLOSIDASE 4 (XY	alpha-L-arabinofuranosida	123.2448839	-1.174408446	0.22804803	-5.149829338	2.61E-07	8.04E-06
PITA_000004071	High quality	AT3G59030	MA_45377g0010	shade<sun	Encodes a proton antiporter. Iı	TRANSPARENT TESTA 12 (	antiporter activity-GO:001	772.768581	-2.24179815	0.29071167	-7.711414282	1.24E-14	1.45E-12
PITA_000004076	High quality	AT2G44350	MA_10430772g0010	shade<sun	encodes a mitochrondrion targ	(ATCS)	ATP binding-GO:0005524,(	83.8561878	-0.533285721	0.16922705	-3.151303036	0.001625438	0.0189361
PITA_000004138	High quality	AT5G59480	MA_120403g0010	shade>sun	Haloacid dehalogenase-like hydrolase (HAD) superfamily p		nucleus-GO:0005634	21.44417081	1.824519121	0.4271688	4.271190036	1.94E-05	0.000414855
PITA_000004143	High quality	AT5G59480	MA_120403g0010	shade<sun	Haloacid dehalogenase-like hydrolase (HAD) superfamily p		nucleus-GO:0005634	299.8785758	-0.617642085	0.19407601	-3.182475241	0.00146022	0.017330235
PITA_000004190	High quality	AT1G77120	MA_95933g0010	shade<sun	Catalyzes the reduction of acet	ALCOHOL DEHYDROGENA	alcohol dehydrogenase (N	104.1265233	-2.262475265	0.56107125	-4.032420604	5.52E-05	0.00103592
PITA_000004200	High quality	AT1G11860	MA_10429284g0010	shade<sun	Glycine cleavage T-protein family;(source:Araport11)		aminomethyltransferase a	370.8150199	-1.277038354	0.1315872	-9.704882681	2.87E-22	9.06E-20
PITA_000004207	High quality	AT1G32470	MA_20265g0010	shade<sun	Single hybrid motif superfamily protein;(source:Araport11		cytoplasm-GO:0005737,cy	588.8366115	-1.129678691	0.17846057	-6.330130367	2.45E-10	1.27E-08
PITA_000004261	Low quality	AT3G09050	MA_646962g0010	shade<sun	8-amino-7-oxononanoate synthase;(source:Araport11)		biological_process_unkno	723.88818	-1.228203566	0.25542937	-4.808388255	1.52E-06	4.12E-05
PITA_000004337	High quality	AT5G12860	MA_10428021g0010	shade<sun	AtpOMT1 encodes dicarboxyla	DICARBOXYLATE TRANSP(	alpha-ketoglutarate transr	268.4598037	-0.800415141	0.12777223	-6.264390686	3.74E-10	1.87E-08
PITA_000004362	Low quality	AT3G02010	MA_10432670g0020	shade<sun	Pentatricopeptide repeat (PPR) superfamily protein;(sour		intracellular membrane-bc	295.3790968	-1.183807357	0.31098204	-3.806674329	0.000140848	0.002354001
PITA_000004384	High quality	AT2G24060	MA_18402g0010	shade<sun	SUPPRESSOR OF VARIEGATION	(ATINFC-2)	chloroplast-GO:0009507,c	307.6375167	-0.529567045	0.16951719	-3.123972492	0.001784271	0.02041149
PITA_000004430	High quality	AT2G18710	MA_10429734g0010	shade<sun	Encodes a component of the tl	SECY HOMOLOG 1 (SCY1)	chloroplast-GO:0009507,c	267.5677427	-0.741010245	0.13794564	-5.371755545	7.80E-08	2.66E-06
PITA_000004448	High quality	AT5G09590	MA_101964g0010	shade<sun	heat shock protein 70 (Hsc70-5	MITOCHONDRIAL HSO70 :	ATP binding-GO:0005524,,	208.7234716	-1.136369362	0.33364664	-3.405906781	0.000659447	0.008895796
PITA_000004474	Low quality	AT4G01150	MA_60075g0010	shade<sun	Integral thylakoid membrane p	CURVATURE THYLAKOID 1	chloroplast-GO:0009507,c	952.4888961	-1.04170476	0.13940518	-7.472496699	7.87E-14	7.97E-12
PITA_000004484	Low quality	AT4G13750	MA_113855g0010	shade>sun	Encodes NO VEIN (NOV), a plaı	NO VEIN (NOV)	auxin polar transport-GO:(	75.16922837	0.526745827	0.18901122	2.786849471	0.00532232	0.047785297
PITA_000004499	High quality	AT3G48420	MA_15334g0010	shade<sun	Haloacid dehalogenase-like hydrolase (HAD) superfamily p		chloroplast-GO:0009507,c	884.8549212	-1.054137752	0.18486667	-5.702151467	1.18E-08	4.57E-07
PITA_000004519	High quality	AT5G20190	MA_136177g0010	shade>sun	Tetratricopeptide repeat (TPR)-like superfamily protein;(s		biological_process_unkno	29.65627649	0.876562149	0.30110014	2.911198036	0.003600457	0.035497863
PITA_000004562	High quality	AT4G20960	MA_10437279g0010	shade<sun	encodes diaminohydroxyphosp	PYRIMIDINE DEAMINASE (	chloroplast-GO:0009507,c	322.4365496	-1.104343023	0.20050619	-5.507775322	3.63E-08	1.32E-06
PITA_000004614	High quality	AT4G35000	MA_20242g0010	shade<sun	Encodes a microsomal ascorba	ASCORBATE PEROXIDASE	cellular oxidant detoxificat	129.0760826	-0.414798536	0.14604244	-2.840260263	0.004507674	0.042247406
PITA_000004626	High quality	AT4G36660	MA_9978g0010	shade<sun	polyol transporter, putative (DUF1195);(source:Araport11		biological_process_unkno	172.2058638	-0.647866355	0.21241515	-3.050000709	0.002288408	0.024995087
PITA_000004637	High quality	AT3G24030	MA_136233g0010	shade<sun	hydroxyethylthiazole kinase family protein;(source:Arapor		cellular_component_unkn	191.1506903	-0.805966521	0.17699444	-4.553626181	5.27E-06	0.000129613
PITA_000004638	High quality	AT5G53460	MA_14068g0010	shade<sun	NADH-dependent glutamate sy	NADH-DEPENDENT GLUTA	ammonia assimilation cycl	220.4229033	-1.069847494	0.20056102	-5.334274423	9.59E-08	3.20E-06
PITA_000004674	Low quality	AT3G09040	MA_112849g0010	shade<sun	Pentatricopeptide repeat (PPR	MITOCHONDRIAL RNA ED	intracellular membrane-bc	557.3651617	-0.974709238	0.26930538	-3.619345629	0.000295349	0.004495293
PITA_000004687	High quality	AT5G66460	MA_111906g0010	shade>sun	Encodes a endo-beta-mannana	ENDO-BETA-MANNASE 7 (	cell periphery-GO:007194	43.51385674	0.709971166	0.24470856	2.90129272	0.003716266	0.036486047
PITA_000004767	High quality	AT2G22780	MA_87937g0010	shade<sun	encodes an peroxisomal NAD-r	PEROXISOMAL NAD-MAL/	chloroplast-GO:0009507,c	2537.184999	-1.947096152	0.21606168	-9.011760474	2.03E-19	4.74E-17
PITA_000004768	High quality	AT1G43670	MA_104409g0010	shade<sun	Encodes a fructose-1,6-bisphos	FRUCTOSE-1,6-BISPHOSPH	cytoplasm-GO:0005737,cy	440.6768126	-1.42128836	0.27200938	-5.225144674	1.74E-07	5.50E-06
PITA_000004872	High quality	AT4G36220	MA_19517g0010	shade>sun	encodes ferulate 5-hydroxylase	FERULIC ACID 5-HYDROXY	endoplasmic reticulum-GC	116.4151048	0.932023271	0.23351266	3.991318031	6.57E-05	0.001208704
PITA_000004874	High quality	AT1G47128	MA_9711944g0010	shade>sun	Cysteine proteinase precursor-	RESPONSIVE TO DEHYDRA	apoplast-GO:0048046,cyst	54.49319336	0.643849022	0.22554699	2.854611501	0.00430895	0.040823338
PITA_000004919	High quality	AT1G61110	MA_137415g0010	shade>sun	NAC transcription regulator. R	NAC DOMAIN CONTAININ	DNA-binding transcription	23.92307294	1.048545515	0.34065657	3.078013448	0.002083855	0.023217158
PITA_000004921	High quality	AT2G30020	MA_60279g0010	shade>sun	Encodes AP2C1. Belongs to th	(AP2C1)	abscisic acid-activated sigr	27.94951899	0.86091622	0.29646372	2.903951359	0.003684854	0.036216687
PITA_000005002	High quality	AT2G43980	MA_10431113g0010	shade<sun	inositol 1,3,4-trisphosphate 5/(	INOSITOL 1,3,4-TRISPHOSI	cytoplasm-GO:0005737,cy	119.6958551	-0.771442132	0.1693651	-4.554906057	5.24E-06	0.000129204
PITA_000005018	Low quality	AT4G25170	MA_10427237g0010	shade>sun	Uncharacterized conserved protein (UCP012943);(source:		biological_process_unkno	58.74107575	0.581997796	0.20221605	2.878098955	0.004000796	0.038561622
PITA_000005033	High quality	AT3G08010	MA_10430622g0010	shade<sun	Encodes a chloroplast-localizec	(ATAB2)	chloroplast-GO:0009507,c	301.3007094	-1.239312883	0.20546407	-6.031774315	1.62E-09	7.28E-08
PITA_000005047	High quality	AT4G33300	MA_75297g0010	shade<sun	Encodes a member of the ADR	ADR1-LIKE 1 (ADR1-L1)	ADP binding-GO:0043531,	172.144886	-0.620476938	0.16880031	-3.675804497	0.000237101	0.003714092

PITA_000005048	High quality	AT5G19940	MA_10436223g0010	shade<sun	Enables plants to cope with mo	FIBRILLIN6 (FBN6)	biological_process_unkno	1386.695403	-1.469073531	0.13655778	-10.7578899	5.44E-27	2.80E-24
PITA_000005132	High quality	AT5G50250	MA_10434075g0010	shade<sun	Encodes a RNA binding protein	CHLOROPLAST RNA-BINDI	chloroplast-GO:0009507,c	563.2000087	-1.092914451	0.15230257	-7.175942185	7.18E-13	6.25E-11
PITA_000005150	High quality	AT1G06680	MA_3005g0010	shade<sun	Encodes a 23 kD extrinsic prote	PHOTOSYSTEM II SUBUNI	apoplast-GO:0048046,calc	603.2550305	-0.955575343	0.17001517	-5.620529959	1.90E-08	7.20E-07
PITA_000005177	Low quality	AT1G55670	MA_100910g0020	shade<sun	Encodes subunit G of photosys	PHOTOSYSTEM I SUBUNIT	chloroplast-GO:0009507,c	529.6576279	-0.652503237	0.1289375	-5.060616631	4.18E-07	1.24E-05
PITA_000005196	High quality	AT1G73580	MA_130250g0010	shade>sun	Calcium-dependent lipid-bindi	C2-DOMAIN ABA-RELATED	GTPase activator activity-G	28.2918142	1.124760746	0.31567987	3.562979013	0.00036667	0.005423705
PITA_000005198	High quality	AT4G21160	MA_10430973g0010	shade>sun	ADP-ribosylation factor GTPase	(ZAC)	cytoplasm-GO:0005737,Go	13.52578201	3.810365143	1.20244236	3.16885472	0.001530409	0.01804437
PITA_000005283	High quality	AT4G34200	MA_53302g0010	shade<sun	Encodes a 3-phosphoglycerate	EMBRYO SAC DEVELOPME	ATP binding-GO:0005524,(	490.2266335	-1.48598057	0.1553111	-9.567768038	1.09E-21	3.28E-19
PITA_000005390	Low quality	AT5G13300	MA_10431120g0010	shade>sun	Belongs to 15-member small G	SCARFACE (SFC)	cellular_component_unkn	43.04522989	0.952791114	0.31463666	3.028226591	0.002459936	0.026422307
PITA_000005434	High quality	AT2G36870	MA_75919g0010	shade<sun	Encodes a xyloglucan endotrar	XYLOGLUCAN ENDOTRANS	carbohydrate metabolic pr	862.4741246	-1.78749008	0.23222101	-7.69736594	1.39E-14	1.60E-12
PITA_000005436	High quality	AT5G65620	MA_10434267g0010	shade<sun	Zincin-like metalloproteases fa	ORGANELLAR OLIGOPEPTI	chloroplast-GO:0009507,c	171.2701474	-0.892694372	0.13059439	-6.835625706	8.16E-12	5.67E-10
PITA_000005493	High quality	AT1G35420	MA_10427595g0010	shade<sun	alpha/beta-Hydrolases superfamily protein;(source:Arapo		chloroplast-GO:0009507,c	336.4165899	-0.85664256	0.22049341	-3.8851163	0.000102281	0.00178144
PITA_000005519	High quality	AT3G28860	MA_10431815g0020	shade<sun	Encodes a member of the ATP-	ATP-BINDING CASSETTE B	acropetal auxin transport-	126.156599	-0.634532459	0.17395468	-3.647688413	0.00026461	0.004099183
PITA_000005526	High quality	AT1G06220	MA_931263g0010	shade>sun	Encodes a protein with similari	MATERNAL EFFECT EMBR'	cell wall-GO:0005618,cyto	78.96342216	0.862337218	0.29030964	2.970404999	0.002974074	0.03068991
PITA_000005554	High quality	AT3G61440	MA_6291g0010	shade<sun	Encodes a cysteine synthase is	CYSTEINE SYNTHASE C1 (C	chloroplast-GO:0009507,c	225.0938922	-1.075302674	0.11219785	-9.583986622	9.34E-22	2.87E-19
PITA_000005619	Low quality	AT3G03280	MA_269349g0010	shade>sun	PADRE protein up-regulated after infection by S. sclerotio		cellular response to hypox	45.51777031	0.738304445	0.23887902	3.090704476	0.001996823	0.022452014
PITA_000005665	Low quality	AT5G15280	MA_100612g0010	shade<sun	Pentatricopeptide repeat (PPR) superfamily protein;(sourc		biological_process_unkno	137.846496	-0.883207206	0.15230271	-5.79902474	6.67E-09	2.70E-07
PITA_000005676	High quality	AT3G25920	MA_648g0010	shade<sun	encodes a plastid ribosomal pr	RIBOSOMAL PROTEIN L15	chloroplast-GO:0009507,c	432.2725764	-1.009968071	0.09129437	-11.06276382	1.90E-28	1.07E-25
PITA_000005704	High quality	AT3G10670	MA_10436888g0020	shade<sun	Plastidic SufC-like ATP-binding	NON-INTRINSIC ABC PROT	ATPase activity-GO:00168	160.5568535	-0.842298226	0.19195604	-4.387974607	1.14E-05	0.000260655
PITA_000005716	Low quality	AT2G28200	MA_119483g0010	shade<sun	C2H2-type zinc finger family protein;(source:Araport11)		DNA-binding transcription	175.9686419	-0.938162149	0.18576787	-5.0501853	4.41E-07	1.31E-05
PITA_000005728	High quality	AT5G67290	MA_10429661g0010	shade<sun	FAD-dependent oxidoreductase family protein;(source:Ar		chloroplast-GO:0009507,c	87.23855851	-0.820672799	0.26583399	-3.087162778	0.002020769	0.022650576
PITA_000005742	High quality	AT5G12950	MA_1577g0010	shade<sun	proline-tRNA ligase (DUF1680);(source:Araport11)		carbohydrate metabolic pr	363.7627894	-1.486187435	0.28051849	-5.298001737	1.17E-07	3.85E-06
PITA_000005744	High quality	AT1G67900	MA_42230g0010	shade<sun	Phototropic-responsive NPH3 family protein;(source:Arap		nucleus-GO:0005634,prote	130.6969223	-0.675066247	0.19791252	-3.410932555	0.000647411	0.008768908
PITA_000005779	High quality	AT4G30000	MA_58673g0010	shade<sun	Dihydropterin pyrophosphokinase / Dihydropteroate synt		2-amino-4-hydroxy-6-hydr	268.698336	-11.70064955	1.23538215	-9.47127947	2.76E-21	7.75E-19
PITA_000005786	Low quality	AT1G33990	MA_10429301g0010	shade<sun	Encodes a protein predicted to	METHYL ESTERASE 14 (ME	jasmonic acid metabolic pr	251.7524426	-1.993857599	0.28838473	-6.913880577	4.72E-12	3.40E-10
PITA_000005896	High quality	AT3G29360	MA_422175g0010	shade<sun	Encodes one of four UDP-gluco	UDP-GLUCOSE DEHYDROG	carbohydrate metabolic pr	116.5473323	-0.653762424	0.19212254	-3.402840768	0.000666891	0.008979712
PITA_000005919	High quality	AT4G33510	MA_54864g0010	shade<sun	Enzyme catalyzing the first con	3-DEOXY-D-ARABINO-HEP	3-deoxy-7-phosphoheptul	3706.292934	-1.837631162	0.22723559	-8.08689864	6.12E-16	8.87E-14
PITA_000005943	High quality	AT1G55620	MA_10429113g0010	shade<sun	Encodes a chloride channel pro	CHLORIDE CHANNEL F (CL	chloroplast-GO:0009507,G	536.3629718	-1.441789481	0.32384698	-4.452070108	8.50E-06	0.000198783
PITA_000006000	High quality	AT1G27680	MA_88294g0010	shade<sun	ADP-glucose pyrophosphorylas	ADPGLC-PPASE LARGE SUI	chloroplast-GO:0009507,g	312.109599	-1.476968906	0.24874313	-5.937727375	2.89E-09	1.24E-07
PITA_000006004	High quality	AT1G08830	MA_10431443g0010	shade<sun	Encodes a cytosolic copper/zin	COPPER/ZINC SUPEROXID	cellular response to coppe	325.8787115	-0.686955336	0.14438985	-4.75764289	1.96E-06	5.21E-05
PITA_000006006	High quality	AT4G09620	MA_191994g0010	shade<sun	Mitochondrial transcription ter	(MTERF12)	chloroplast-GO:0009507,c	253.4555568	-0.586297862	0.14115586	-4.153549441	3.27E-05	0.000657845
PITA_000006033	High quality	AT5G01130	MA_72874g0010	shade>sun	hypothetical protein (DUF674);(source:Araport11)		biological_process_unkno	318.1597748	11.60963311	3.90688214	2.971585189	0.002962667	0.030622317
PITA_000006099	Low quality	AT3G55760	MA_10429684g0010	shade<sun	hypothetical protein;(source:A	LIKE EARLY STARVATION (	chloroplast-GO:0009507,c	490.2967063	-1.101795716	0.38164937	-2.886931855	0.003890185	0.037676828
PITA_000006141	High quality	AT1G76570	MA_10435703g0010	shade<sun	Chlorophyll A-B binding family	LIGHT-HARVESTING COMF	chlorophyll binding-GO:00	194.8251937	-1.504664251	0.20093169	-7.488436713	6.97E-14	7.17E-12
PITA_000006164	Low quality	AT2G05710	MA_7469g0010	shade<sun	Encodes an aconitase that can	ACONITASE 3 (ACO3)	4 iron, 4 sulfur cluster binc	113.5238974	-0.890309122	0.27140533	-3.280367169	0.001036721	0.012995078
PITA_000006178	High quality	AT5G48385	MA_58118g0010	shade>sun	FRIGIDA-like protein;(source:Araport11)		nucleus-GO:0005634,plast	59.6033454	3.823353429	1.25491924	3.046692822	0.00231374	0.025206276
PITA_000006274	High quality	AT4G16160	MA_21820g0010	shade<sun	Homologous to pea OEP16 anc	(ATOEP16-2)	amino acid transmembran	140.3478699	-0.512788588	0.1666881	-3.076335986	0.002095616	0.023282566
PITA_000006279	Low quality	AT4G28290	MA_10427809g0010	shade<sun	hypothetical protein;(source:Araport11)		biological_process_unkno	892.2250238	-2.254030259	0.3667285	-6.146318728	7.93E-10	3.71E-08
PITA_000006280	High quality	AT1G76730	MA_25157g0010	shade<sun	Encodes a paralog of ATP-depe	CLUSTERS OF ORTHOLOG(	chloroplast-GO:0009507,c	111.5776991	-0.617763171	0.15110396	-4.088332174	4.34E-05	0.000843551
PITA_000006313	High quality	AT5G53850	MA_156526g0010	shade<sun	Encodes a trifunctional dehydr	DEHYDRATASE-ENOLASE-I	acireductone synthase act	141.5242705	-0.889714215	0.22410237	-3.970124164	7.18E-05	0.001301497
PITA_000006314	High quality	AT4G16745	MA_125709g0010	shade<sun	Exostosin family protein;(source:Araport11)		nucleus-GO:0005634,prote	149.2497676	-0.61878842	0.13078186	-4.731454625	2.23E-06	5.87E-05
PITA_000006352	High quality	AT4G34350	MA_105092g0010	shade<sun	Arabidopsis ISPH is involved in	4-HYDROXY-3-METHYLBU	4-hydroxy-3-methylbut-2-(	762.1700452	-1.048657646	0.32439932	-3.232613611	0.001226633	0.014973716
PITA_000006379	Low quality	AT1G56500	MA_287596g0010	shade<sun	Encodes a thylakoid membrane	SUPPRESSOR OF QUENCH	chloroplast-GO:0009507,c	354.1466383	-1.022536984	0.25647585	-3.98687428	6.69E-05	0.001228869
PITA_000006469	High quality	AT2G38470	MA_10434651g0010	shade>sun	Member of the plant WRKY tra	WRKY DNA-BINDING PRO1	camalexin biosynthetic pro	44.75527608	0.714493355	0.23124038	3.089829566	0.002002714	0.022484775
PITA_000006540	High quality	AT2G05140	MA_10429623g0010	shade<sun	phosphoribosylaminoimidazole carboxylase family protein		5-amino-4-imidazole carbo	106.8491045	-0.765988029	0.21095961	-3.630969992	0.000282358	0.004331551
PITA_000006593	High quality	AT5G44050	MA_94941g0010	shade<sun	MATE efflux family protein;(so	(ATDTX28)	antiporter activity-GO:001	164.34125	-0.59242781	0.1874533	-3.160402119	0.001575515	0.018506985
PITA_000006650	High quality	AT4G17940	MA_13355g0010	shade<sun	Tetratricopeptide repeat (TPR)-like superfamily protein;(s		biological_process_unkno	596.4908721	-0.932082502	0.22913134	-4.067896177	4.74E-05	0.000913301
PITA_000006702	High quality	AT5G06690	MA_102427g0010	shade<sun	Encodes a thioredoxin (WCRKC	WCRKC THIOREDOXIN 1 (\	cell redox homeostasis-GC	313.8227099	-0.890472976	0.17565981	-5.069304045	3.99E-07	1.20E-05
PITA_000006831	High quality	AT1G21350	MA_98916g0010	shade<sun	Thioredoxin superfamily protein;(source:Araport11)		antioxidant activity-GO:00	195.4007026	-0.904248416	0.2383126	-3.794379324	0.000148013	0.002462331
PITA_000006847	Low quality	AT5G56260	MA_12483g0010	shade<sun	Ribonuclease E inhibitor RraA/Dimethylmenaquinone me		4-hydroxy-4-methyl-2-oxo	152.811486	-0.5141485	0.17168253	-2.994763093	0.002746581	0.028849965
PITA_000006881	High quality	AT5G13630	MA_108278g0010	shade<sun	Encodes magnesium chelatase	GENOMES UNCOUPLED 5	chlorophyll biosynthetic p	815.7991593	-1.387883957	0.17633034	-7.870931286	3.52E-15	4.44E-13
PITA_000006901	Low quality	AT1G73500	MA_10194g0020	shade<sun	member of MAP Kinase Kinase	MAP KINASE KINASE 9 (M	camalexin biosynthetic pro	53.56629418	-9.374121016	1.44816102	-6.47312065	9.60E-11	5.30E-09
PITA_000006988	Low quality	AT2G19810	MA_98656g0010	shade>sun	Encodes Oxidation-related Zinc	OXIDATION-RELATED ZINC	defense response to virus-	28.563369	1.139235916	0.32417301	3.514283715	0.000440942	0.006343723
PITA_000007005	High quality	AT5G14370	MA_91504g0010	shade<sun	CCT motif family protein;(source:Araport11)		nucleus-GO:0005634,regu	276.1379726	-0.690262276	0.14373741	-4.802245279	1.57E-06	4.24E-05
PITA_000007015	High quality	AT1G60470	MA_10312128g0010	shade<sun	Predicted to encode a galactin	GALACTINOL SYNTHASE 4	galactose metabolic proce	121.357874	-1.234230367	0.35583449	-3.468551801	0.000523272	0.007352039
PITA_000007016	High quality	AT1G11870	MA_251870g0010	shade<sun	Seryl-tRNA synthetase targeted	SERYL-TRNA SYNTHETASE	chloroplast-GO:0009507,c	399.6275887	-0.756779539	0.21399828	-3.536381386	0.000405649	0.00590078
PITA_000007051	High quality	AT1G02850	MA_10431319g0010	shade>sun	beta glucosidase 11;(source:Ar	BETA GLUCOSIDASE 11 (B	beta-glucosidase activity-G	97.81499868	0.614310343	0.20642267	2.97598299	0.002920511	0.030310814
PITA_000007154	Low quality	AT2G43650	MA_348135g0010	shade>sun	SAS10/C1D family protein. Los	EMBRYO DEFECTIVE 2777	maturation of SSU-rRNA fr	62.65898796	0.839225681	0.23438603	3.580527799	0.000342901	0.005126236
PITA_000007196	High quality	AT1G32100	MA_73179g0010	shade<sun	Encodes a pinoresinol reducta	PINORESINOL REDUCTASE	cytoplasm-GO:0005737,lig	461.2861268	-1.374781412	0.35130117	-3.913398314	9.10E-05	0.001609532
PITA_000007207	Low quality	AT4G33590	MA_58991g0010	shade>sun	transmembrane protein;(source:Araport11)		extracellular region-GO:00	34.96175999	0.891423143	0.26684661	3.340582605	0.000836028	0.01089082
PITA_000007252	High quality	AT1G71695	MA_10275776g0010	shade>sun	Peroxidase superfamily protein;(source:Araport11)		cell wall-GO:0005618,extra	71.12984275	0.848459132	0.20066795	4.228174599	2.36E-05	0.000493907
PITA_000007288	Low quality	AT1G21600	MA_61848g0010	shade<sun	Present in transcriptionally act	PLASTID TRANSCRIPTION/	chloroplast-GO:0009507,c	215.8096477	-0.676571199	0.16039428	-4.218175237	2.46E-05	0.000513509
PITA_000007331	Low quality	AT3G26935	MA_10430260g0010	shade>sun	DHHC-type zinc finger family protein;(source:Araport11)		cytosol-GO:0005829,endo	48.15429673	0.762951241	0.22747606	3.353984748	0.000796568	0.010425238
PITA_000007356	High quality	AT1G03630	MA_91782g0010	shade<sun	Encodes for a protein with pro	PROTOCHLOROPHYLLIDE (	chlorophyll biosynthetic p	256.2433273	-0.869146928	0.20811456	-4.176290925	2.96E-05	0.000604099
PITA_000007357	High quality	AT2G22240	MA_5176g0010	shade<sun	** Referred to as MIPS1 in Mit	MYO-INOSITOL-1-PHOSPH	cellular response to phosp	447.7362128	-1.410407135	0.21206085	-6.650954941	2.91E-11	1.80E-09
PITA_000007397	Low quality	AT2G45120	MA_109421g0010	shade>sun	C2H2-like zinc finger protein;(s	(ZAT4)	DNA-binding transcription	36.21776998	1.123772137	0.34379185	3.268757361	0.001080209	0.013455134
PITA_000007413	High quality	AT5G05580	MA_140292g0010	shade<sun	Encodes a temperature sensitiv	FATTY ACID DESATURASE	1-16:0-2-18:2-digalactosyl	593.8822599	-1.89039925	0.12751189	-14.82527843	1.01E-49	2.31E-46
PITA_000007433	Low quality	AT5G55230	MA_50450g0010	shade<sun	Binds and bundles microtubule	MICROTUBULE-ASSOCIATI	cell growth-GO:0016049,c	1102.693986	-1.113539854	0.34259679	-3.250292708	0.001152863	0.014220387
PITA_000007472	High quality	AT3G53710	MA_46769g0010	shade>sun	A member of ARF GAP domain	ARF-GAP DOMAIN 6 (AGD	cytosol-GO:0005829,Golgi	56.29568879	0.625335187	0.2007042	3.115705502	0.001835054	0.020893937
PITA_000007588	High quality	AT1G61520	MA_815900g0010	shade<sun	PSI type III chlorophyll a/b-bin	PHOTOSYSTEM I LIGHT HA	chlorophyll binding-GO:00	722.6326949	-0.924602026	0.14474961	-6.387595839	1.69E-10	8.93E-09
PITA_000007610	High quality	AT1G77760	MA_16402g0010	shade<sun	Encodes the cytosolic minor is	NITRATE REDUCTASE 1 (N	chloroplast-GO:0009507,c	219.8445687	-1.403386277	0.41625677	-3.371443748	0.000747753	0.009894193
PITA_000007622	Low quality	AT4G02420	MA_10213388g0010	shade>sun	Concanavalin A-like lectin prot	L-TYPE LECTIN RECEPTOR	defense response to bacte	9.656725443	6.569630675	1.41535021	4.641699724	3.46E-06	8.79E-05
PITA_000007776	High quality	AT4G22530	MA_34035g0010	shade<sun	S-adenosyl-L-methionine-dependent methyltransferases s		cytoplasm-GO:0005737,cy	621.9210881	-1.028925493	0.36666623	-2.806163761	0.005013521	0.045828557
PITA_000007785	High quality	AT1G69530	MA_114349g0010	shade<sun	Member of Alpha-Expansin Ge	EXPANSIN A1 (EXPA1)	extracellular region-GO:00	169.7346183	-2.404433253	0.34838514	-6.901652678	5.14E-12	3.67E-10
PITA_000007793	High quality	AT1G26770	MA_10020g0010	shade>sun	Encodes an expansin. Naming	EXPANSIN A10 (EXPA10)	extracellular region-GO:00	30.34216647	0.853921797	0.28084852	3.040506624	0.002361805	0.025597216
PITA_000007852	High quality	AT3G05390	MA_6067g0010	shade<sun	S-adenosyl-L-methionine-dependent methyltransferase;(s		root development-GO:004	218.3230029	-0.781020499	0.16216575	-4.816186495	1.46E-06	3.98E-05
PITA_000007951	High quality	AT3G48700	MA_10429351g0010	shade>sun	carboxyesterase 13;(source:Ar	CARBOXYESTERASE 13 (C)	cytosol-GO:0005829,hydro	36.23154541	0.930202145	0.27896536	3.334471926	0.000854615	0.011081438
PITA_000008013	High quality	AT5G09650	MA_10426244g0010	shade<sun	Encodes a protein with inorgar	PYROPHOSPHORYLASE 6 (	chloroplast-GO:0009507,c	189.7141341	-0.724027114	0.1915251	-3.780325	0.000156624	0.0025885
PITA_000008017	High quality	AT1G55850	MA_10436687g0010	shade>sun	encodes a protein similar to ce	CELLULOSE SYNTHASE LIK	cellulose biosynthetic proc	42.03309145	0.811454339	0.24571265	3.302452429	0.000958433	0.012195606
PITA_000008068	High quality	AT1G23740	MA_10433694g0010	shade<sun	AOR is an alkenal/one oxidore	ALKENAL/ONE OXIDORED	2-alkenal reductase (NADF	500.0823514	-1.017007343	0.15069898	-6.748601259	1.49E-11	9.78E-10
PITA_000008130	High quality	AT2G33450	MA_10426581g0010	shade<sun	Ribosomal L28 family;(source:/	PLASTID RIBOSOMAL PRO	chloroplast-GO:0009507,c	289.5974799	-0.71538772	0.13575558	-5.269674669	1.37E-07	4.44E-06
PITA_000008146	Low quality	AT2G46690	MA_138556g0010	shade>sun	SAUR-like auxin-responsive pro	SMALL AUXIN UPREGULAT	molecular_function_unkn	48.90826629	1.267184552	0.41916729	3.023099778	0.002501997	0.026771478
PITA_000008149	High quality	AT5G20680	MA_430863g0010	shade<sun	Encodes a member of the TBL	TRICHOME BIREFRINGENC	endosome-GO:0005768,G	366.4319947	-0.887994682	0.21075018	-4.213494261	2.51E-05	0.00052237
PITA_000008184	High quality	AT5G54390	MA_10433905g0010	shade<sun	Encodes a 3'-phosphoadenosin	HAL2-LIKE (HL)	3'(2'),5'-bisphosphate nucl	161.1965007	-0.542217106	0.14114398	-3.841588645	0.000122241	0.002074635
PITA_000008261	High quality	AT5G52390	MA_35310g0010	shade<sun	PAR1 protein;(source:Araport11)		biological_process_unkno	5275.850971	-5.787604372	2.02307708	-2.86079281	0.004225831	0.040217156
PITA_000008353	Low quality	AT1G64940	MA_89703g0010	shade<sun	member of CYP89A	CYTOCHROME P450, FAM	heme binding-GO:002003	1189.432757	-2.171182268	0.53611971	-4.049808726	5.13E-05	0.000977793
PITA_000008367	Low quality	AT3G19640	MA_10433792g0010	shade<sun	Transmembrane magnesium tr	MAGNESIUM TRANSPORT	chloroplast-GO:0009507,m	95.67056375	-0.702236891	0.24867957	-2.823862412	0.004744875	0.044059552
PITA_000008377	High quality	AT4G38620	MA_22140g0010	shade>sun	Encodes a R2R3 MYB protein w	MYB DOMAIN PROTEIN 4	regulation of phenylpropa	25.84059091	1.203693603	0.32336506	3.722398468	0.000197339	0.003157929
PITA_000008420	High quality	AT5G08050	MA_114154g0010	shade<sun	Encodes a grana core localized	(RIQ1)	chloroplast-GO:0009507,c	2006.004784	-1.353559139	0.22538794	-6.005463988	1.91E-09	8.44E-08
PITA_000008498	High quality	AT3G17611	MA_10426917g0010	shade>sun	RHOMBOID-like protein 14;(so	RHOMBOID-LIKE PROTEIN	plasma membrane-GO:00	7.027480037	6.113119018	1.59569746	3.831001272	0.000127623	0.002155826
PITA_000008503	Low quality	AT2G34930	MA_19613g0010	shade>sun	disease resistance family protein / LRR family protein;(sou		cell wall-GO:0005618,defe	40.38902791	1.699237385	0.38404214	4.424611758	9.66E-06	0.000222933
PITA_000008513	High quality	AT5G44370	MA_68437g0010	shade<sun	Encodes an inorganic phospha	PHOSPHATE TRANSPORTE	Golgi apparatus-GO:00057	112.4728751	-0.984568235	0.15690569	-6.274904598	3.50E-10	1.76E-08
PITA_000008574	Low quality	AT5G64950	MA_804585g0010	shade>sun	mTERF family protein which fu	MITOCHONDRIAL TRANSC	chloroplast-GO:0009507,c	106.1068249	0.658525575	0.17472407	3.768946022	0.000163938	0.002686621
PITA_000008605	High quality	AT5G22620	MA_10436249g0010	shade<sun	encodes a putative 2-carboxy-D-arabinitol 1-phosphate ph		2-carboxy-D-arabinitol-1-p	472.5350588	-0.75401818	0.26106456	-2.888244105	0.003873991	0.037534404
PITA_000008606	High quality	AT5G38520	MA_10433730g0010	shade<sun	CLD1 is involved in steady-state	CHLOROPHYLL DEPHYTYL/	chlorophyll metabolic proc	1951.465524	-1.650808156	0.28501955	-5.791912022	6.96E-09	2.81E-07
PITA_000008626	Low quality	AT4G30490	MA_80622g0010	shade>sun	AFG1-like ATPase family protein;(source:Araport11)		ATPase activity-GO:00168	33.03465776	1.083436964	0.29612268	3.658743599	0.000253455	0.003940893
PITA_000008660	High quality	AT5G57620	MA_14061g0010	shade>sun	MYB36 is a transcriptional regu	MYB DOMAIN PROTEIN 36	Casparian strip-GO:00482	85.92383168	1.326931404	0.43578562	3.044917858	0.002327439	0.025311774
PITA_000008672	Low quality	AT4G00755	MA_10431612g0010	shade>sun	F-box family protein;(source:Araport11)		biological_process_unkno	40.19269773	1.068365267	0.33333007	3.205127169	0.001350028	0.016259652
PITA_000008674	High quality	AT4G13650	MA_9258g0010	shade<sun	Pentatricopeptide repeat (PPR) superfamily protein;(sourc		mitochondrion-GO:00057	170.7881339	-2.194017626	0.33664317	-6.517338829	7.16E-11	4.06E-09
PITA_000008676	High quality	AT5G38430	MA_47626g0010	shade<sun	Encodes a member of the Rubi	RUBISCO SMALL SUBUNIT	apoplast-GO:0048046,chlo	1434.732463	-1.175046593	0.16050288	-7.321031344	2.46E-13	2.27E-11
PITA_000008702	High quality	AT1G60470	MA_3384g0010	shade>sun	Predicted to encode a galactin	GALACTINOL SYNTHASE 4	galactose metabolic proce	22.16474959	1.042218388	0.37589013	2.772667575	0.005559888	0.049182879
PITA_000008771	High quality	AT4G13670	MA_692g0010	shade<sun	plastid transcriptionally active	PLASTID TRANSCRIPTION/	chloroplast-GO:0009507,c	162.3273908	-1.09082306	0.25849994	-4.219819458	2.44E-05	0.00051045
PITA_000008784	High quality	AT2G30780	MA_898819g0010	shade<sun	Tetratricopeptide repeat (TPR)-like superfamily protein;(s		biological_process_unkno	529.5843347	-1.430709117	0.21182796	-6.754108875	1.44E-11	9.46E-10
PITA_000008875	High quality	AT3G13790	MA_166367g0020	shade>sun	Encodes a protein with inverta	(ATBFRUCT1)	beta-fructofuranosidase ac	31.56335434	2.187836494	0.75613555	2.893444812	0.003810412	0.037161099
PITA_000008977	Low quality	AT4G01935	MA_10429903g0010	shade<sun	insulin-induced protein;(source:Araport11)		biological_process_unkno	411.1056853	-0.797543509	0.18267171	-4.365993562	1.27E-05	0.000283688
PITA_000009088	High quality	AT2G39510	MA_10430121g0020	shade<sun	Encodes a plasma membrane-l	USUALLY MULTIPLE ACIDS	chloroplast-GO:0009507,L	184.6753344	-1.33613476	0.36085774	-3.702663476	0.000213348	0.003381918
PITA_000009171	Low quality	AT5G15600	MA_110382g0010	shade<sun	SPIRAL1-LIKE4 belongs to a six-	SPIRAL1-LIKE4 (SP1L4)	cortical microtubule organ	3434.801158	-1.718521651	0.43848175	-3.919254705	8.88E-05	0.001575331
PITA_000009191	High quality	AT4G31940	MA_10055697g0010	shade>sun	The gene encodes a cytochrom	CYTOCHROME P450, FAM	cellular response to iron ic	22.80397701	1.239981132	0.43194551	2.870688795	0.004095785	0.039216289
PITA_000009232	High quality	AT5G04590	MA_94437g0010	shade<sun	A.thaliana gene encoding sulfit	SULFITE REDUCTASE (SIR)	apoplast-GO:0048046,chlo	119.9763504	-0.597930433	0.15542088	-3.84716921	0.00011949	0.002033434
PITA_000009360	High quality	AT3G63490	MA_10002614g0010	shade<sun	Ribosomal protein L1p/L10e fa	EMBRYO DEFECTIVE 3126	chloroplast-GO:0009507,c	367.6529416	-1.117177765	0.12380207	-9.023902187	1.82E-19	4.32E-17
PITA_000009366	High quality	AT2G42750	MA_8214778g0010	shade<sun	DNAJ heat shock N-terminal dc	DNA J PROTEIN C77 (DJC7	chloroplast-GO:0009507,c	505.0290252	-0.980568321	0.34686296	-2.826961738	0.004699194	0.043683626
PITA_000009417	High quality	AT1G54690	MA_94328g0020	shade>sun	Encodes HTA3, a histone H2A p	GAMMA HISTONE VARIAN	chromatin silencing-GO:00	78.9467475	1.507660609	0.2446645	6.162155235	7.18E-10	3.37E-08
PITA_000009464	High quality	AT1G79320	MA_10433349g0010	shade>sun	Encodes a putative metacaspas	METACASPASE 6 (MC6)	cellular_component_unkn	103.8510291	1.076056371	0.2654147	4.054245545	5.03E-05	0.000963886
PITA_000009549	High quality	AT3G57680	MA_74926g0010	shade<sun	Peptidase S41 family protein;(source:Araport11)		endopeptidase activity-GC	1047.516442	-1.117172877	0.19290011	-5.791458093	6.98E-09	2.81E-07
PITA_000009570	High quality	AT3G26618	MA_15391g0010	shade>sun	eukaryotic release factor 1-3;(s	EUKARYOTIC RELEASE FAC	cytoplasm-GO:0005737,cy	43.58745119	0.797091749	0.25294799	3.151208083	0.001625966	0.0189361
PITA_000009619	Low quality	AT3G52300	MA_100379g0010	shade<sun	ATP synthase D chain;(source:/	ATP SYNTHASE D CHAIN, I	ATP synthesis coupled pro	125.4698355	-0.463412937	0.16478956	-2.812149772	0.004921158	0.045329296
PITA_000009637	High quality	AT5G03820	MA_10224291g0010	shade<sun	GDSL-motif esterase/acyltransferase/lipase. Enzyme grou		extracellular region-GO:00	110.9181405	-0.948890808	0.29230207	-3.246267871	0.001169288	0.014364069
PITA_000009665	High quality	AT1G09530	MA_26114g0010	shade>sun	Transcription factor interacting	PHYTOCHROME INTERACT	de-etiolation-GO:0009704	66.90098629	0.630021489	0.20090206	3.135963367	0.001712906	0.019698809
PITA_000009668	High quality	AT3G27180	MA_10426931g0010	shade<sun	S-adenosyl-L-methionine-dependent methyltransferases s		chloroplast-GO:0009507,R	200.4447843	-1.152323873	0.17124317	-6.729167084	1.71E-11	1.11E-09
PITA_000009808	High quality	AT5G62790	MA_8158336g0010	shade<sun	1-Deoxy-d-xylulose 5-phosphat	1-DEOXY-D-XYLULOSE 5-P	1-deoxy-D-xylulose-5-phos	234.2564789	-0.883687048	0.14454429	-6.113607504	9.74E-10	4.51E-08
PITA_000009827	High quality	AT4G17370	MA_10436710g0010	shade<sun	Oxidoreductase family protein;(source:Araport11)		chloroplast-GO:0009507,e	150.6306041	-1.25115924	0.25961568	-4.819274481	1.44E-06	3.93E-05
PITA_000009924	Low quality	AT5G46110	MA_10428353g0020	shade<sun	Encodes a chloroplast triose ph	ACCLIMATION OF PHOTOS	antiporter activity-GO:001	1086.749769	-0.990660492	0.10447059	-9.482673387	2.48E-21	7.10E-19
PITA_000009955	Low quality	AT3G02330	MA_10138132g0010	shade<sun	Involved in cytidine to uridine	MITOCHONDRIAL EDITING	cytidine to uridine editing-	240.6955693	-2.766358208	0.83902918	-3.297094172	0.000976907	0.012383722
PITA_000009967	High quality	AT2G39770	MA_17910g0010	shade<sun	Encodes a GDP-mannose pyrop	CYTOKINESIS DEFECTIVE 1	cellulose biosynthetic proc	258.7430131	-0.727047137	0.18227991	-3.988630094	6.65E-05	0.001221588
PITA_000010054	Low quality	AT1G75460	MA_10436586g0030	shade<sun	ATP-dependent protease La (LON) domain protein;(source		chloroplast-GO:0009507	458.5481259	-0.783441776	0.18380633	-4.26232212	2.02E-05	0.000430215
PITA_000010057	Low quality	AT2G31040	MA_6812047g0010	shade<sun	Encodes an integral thylakoid p	CONSERVED ONLY IN THE	chloroplast proton-transpo	238.7456566	-0.837737381	0.13494965	-6.207777292	5.37E-10	2.60E-08
PITA_000010147	High quality	AT3G13224	MA_125759g0010	shade<sun	Belongs to a member of the RN	RNA-BINDING GLYCINE-RI	extracellular region-GO:00	557.218741	-1.401883698	0.25522682	-5.492697389	3.96E-08	1.43E-06
PITA_000010201	High quality	AT5G05270	MA_502949g0010	shade<sun	Chalcone-flavanone isomerase	CHALCONE ISOMERASE LI	chalcone isomerase activit	235.3962644	-1.255053369	0.25857282	-4.853771464	1.21E-06	3.36E-05
PITA_000010230	High quality	AT5G64330	MA_52004g0010	shade>sun	Involved in blue light response	NON-PHOTOTROPIC HYPC	blue light signaling pathwa	15.36684623	1.397739863	0.43621016	3.204280864	0.001354003	0.016291966
PITA_000010241	High quality	AT2G32020	MA_100836g0010	shade>sun	Acyl-CoA N-acyltransferases (NAT) superfamily protein;(sc		cytoplasm-GO:0005737,N-	69.3333478	0.701148367	0.25265079	2.775167935	0.005517321	0.048926454
PITA_000010271	High quality	AT4G36360	MA_10427427g0010	shade<sun	putative beta-galactosidase (B(	BETA-GALACTOSIDASE 3 (	beta-galactosidase activity	303.6177013	-1.267900067	0.20725971	-6.117445839	9.51E-10	4.41E-08
PITA_000010337	Low quality	AT4G26850	MA_56912g0020	shade<sun	Encodes a novel protein involv	VITAMIN C DEFECTIVE 2 (\	cytoplasm-GO:0005737,de	198.1977766	-0.930479401	0.17436909	-5.336263379	9.49E-08	3.17E-06
PITA_000010362	High quality	AT5G04330	MA_7247276g0010	shade>sun	Cytochrome P450 superfamily	CYTOCHROME P450 84A4	heme binding-GO:002003	39.71643422	4.802590603	1.59844488	3.004539384	0.002659833	0.028043894
PITA_000010397	Low quality	AT1G64080	MA_444980g0010	shade>sun	Encodes a member of the MAK	MEMBRANE-ASSOCIATED	biological_process_unkno	58.82775266	1.456643016	0.51278448	2.840653483	0.00450212	0.042247406
PITA_000010409	High quality	AT3G48310	MA_10436871g0020	shade>sun	putative cytochrome P450	CYTOCHROME P450, FAM	heme binding-GO:002003	7.283368257	2.810460626	0.78957548	3.55945785	0.000371621	0.005480869
PITA_000010423	Low quality	AT5G16760	MA_41802g0010	shade<sun	Encodes a inositol 1,3,4-trisphc	INOSITOL 1,3,4-TRISPHOSI	cytoplasm-GO:0005737,cy	164.1005418	-0.970348339	0.16259484	-5.967891282	2.40E-09	1.04E-07

PITA_000010440	High quality	AT1G66670	MA_952592g0010	shade<sun	One of several nuclear-encode	CLP PROTEASE PROTEOLY	ATPase binding-GO:00511	336.0823666	-1.012061885	0.30920715	-3.273086975	0.001063797	0.013281668
PITA_000010453	High quality	AT2G29630	MA_10434897g0010	shade<sun	Encodes a protein involved in t	THIAMINC (THIC)	ADP-ribose pyrophosphoh	152.1573449	-1.039336128	0.21086606	-4.928892423	8.27E-07	2.35E-05
PITA_000010567	High quality	AT1G17050	MA_17853g0010	shade<sun	Encodes one of the two paralo	SOLANESYL DIPHOSPHATE	all-trans-nonaprenyl-diphc	417.0993364	-1.406646111	0.17404352	-8.082151612	6.36E-16	9.17E-14
PITA_000010697	High quality	AT4G37200	MA_10429518g0010	shade<sun	Encodes thioredoxin-like prote	HIGH CHLOROPHYLL FLUO	chloroplast-GO:0009507,c	307.5527631	-0.478992632	0.1326098	-3.612045568	0.000303791	0.004607104
PITA_000010781	Low quality	AT3G06035	MA_31797g0010	shade<sun	Glycoprotein membrane precursor	GPI-anchored;(source:	anchored component of m	155.6425568	-0.609185066	0.16707934	-3.646082561	0.000266268	0.00411981
PITA_000010799	High quality	AT1G73340	MA_110777g0010	shade<sun	ADTO1 is required for the activ	ABIETANE DITERPENE OXI	brassinosteroid biosynthet	255.4877494	-1.745389388	0.26729537	-6.5298152	6.59E-11	3.76E-09
PITA_000010811	High quality	AT3G21190	MA_24566g0010	shade<sun	O-fucosyltransferase family prc	MANNAN SYNTHESIS RELA	cell wall pectin biosynthet	234.6428371	-1.196227465	0.24258503	-4.931167783	8.17E-07	2.33E-05
PITA_000010907	High quality	AT3G24590	MA_10430181g0010	shade<sun	Encodes a signal peptidase Plsp	PLASTIDIC TYPE I SIGNAL I	chloroplast-GO:0009507,c	567.432105	-1.529545235	0.21319436	-7.174417037	7.26E-13	6.29E-11
PITA_000010949	Low quality	AT3G06790	MA_123833g0010	shade<sun	Encodes a protein involved in I	MULTIPLE ORGANELLAR R	chloroplast-GO:0009507,c	134.8249604	-0.644910322	0.18407736	-3.50347449	0.000459231	0.006569046
PITA_000010971	High quality	AT5G49900	MA_10430605g0020	shade<sun	Beta-glucosidase, GBA2 type family protein;(source:Arapc		beta-glucosidase activity-G	166.5727308	-0.809059142	0.22933728	-3.527813426	0.000419007	0.006069711
PITA_000010985	High quality	AT1G72210	MA_686524g0010	shade<sun	basic helix-loop-helix (bHLH) D	(BHLH096)	DNA-binding transcription	107.2139226	-0.687041398	0.18865522	-3.641783136	0.000270756	0.00417899
PITA_000011054	High quality	AT3G56430	MA_206524g0010	shade<sun	TIM domain protein. Associate	ATTIM21-LIKE 2 (ATTIM21	biological_process_unkno	114.1744369	-0.493526432	0.14544612	-3.393190826	0.000690835	0.00926255
PITA_000011146	High quality	AT5G48250	MA_90307g0010	shade>sun	B-box type zinc finger protein v	B-BOX DOMAIN PROTEIN	DNA-binding transcription	58.48611903	0.578655144	0.20585382	2.811000282	0.004938774	0.045408635
PITA_000011154	Low quality	AT5G21940	MA_102706g0010	shade>sun	hybrid signal transduction histidine kinase M-like protein;		nucleus-GO:0005634,prote	35.04542432	1.24191553	0.28046313	4.428088377	9.51E-06	0.000219772
PITA_000011252	High quality	AT1G10510	MA_19479g0010	shade<sun	RNI-like superfamily protein;(s	EMBRYO DEFECTIVE 2004	chloroplast-GO:0009507,c	120.1251212	-0.669336835	0.21838466	-3.06494443	0.002177104	0.024039656
PITA_000011253	High quality	AT5G23090	MA_152275g0010	shade<sun	nuclear factor Y, subunit B13;(	NUCLEAR FACTOR Y, SUBL	DNA-binding transcription	114.2901978	-0.64856673	0.22215334	-2.919455183	0.003506438	0.034766771
PITA_000011255	High quality	AT4G15630	MA_10431939g0010	shade>sun	Uncharacterized protein family	CASP-LIKE PROTEIN 1E1 ((	4 iron, 4 sulfur cluster binc	16.64711684	1.753018417	0.47623721	3.680977396	0.000232342	0.003644064
PITA_000011262	High quality	AT2G45030	MA_10435583g0010	shade<sun	Translation elongation factor EFG/EF2 protein;(source:Ara		chloroplast-GO:0009507,G	226.8695009	-1.241098655	0.22896277	-5.420525963	5.94E-08	2.08E-06
PITA_000011445	High quality	AT5G17540	MA_457000g0010	shade>sun	HXXXD-type acyl-transferase family protein;(source:Arapo		cytoplasm-GO:0005737,tra	29.08464669	1.179303527	0.36485721	3.23223299	0.001228269	0.014986422
PITA_000011481	High quality	AT4G39470	MA_10426317g0010	shade<sun	Tetratricopeptide repeat (TPR)-like superfamily protein;(s		biological_process_unkno	283.6959713	-1.423147853	0.20684776	-6.880170414	5.98E-12	4.24E-10
PITA_000011540	High quality	AT3G07370	MA_10428939g0020	shade<sun	Encodes AtCHIP, a new class of	CARBOXYL TERMINUS OF	cellular response to misfol	237.9417432	-0.872682314	0.16579455	-5.263636783	1.41E-07	4.57E-06
PITA_000011568	Low quality	AT3G55250	MA_11912g0010	shade<sun	Encodes a nucleus-encoded pr	PIGMENT DEFECTIVE 329	chloroplast-GO:0009507,c	265.4204581	-1.197184499	0.17480377	-6.848734004	7.45E-12	5.23E-10
PITA_000011579	High quality	AT4G25610	MA_3965g0010	shade<sun	C2H2-like zinc finger protein;(source:Araport11)		biological_process_unkno	94.56447328	-0.746488518	0.26359307	-2.831973181	0.004626173	0.043084224
PITA_000011629	High quality	AT5G17710	MA_10432570g0020	shade<sun	Chloroplast GrpE protein invol	EMBRYO DEFECTIVE 1241	adenyl-nucleotide exchang	244.6584626	-1.122171877	0.16479708	-6.809416165	9.80E-12	6.66E-10
PITA_000011646	High quality	AT5G05700	MA_10352794g0010	shade>sun	Encodes an arginyl-tRNA:prote	ARGININE-TRNA PROTEIN	arginyltransferase activity-	40.42009988	1.347003058	0.34253284	3.932478553	8.41E-05	0.001499546
PITA_000011648	Low quality	AT5G17520	MA_114460g0010	shade<sun	Encodes a maltose transporter	ROOT CAP 1 (RCP1)	carbohydrate metabolic pr	214.8212533	-0.700237409	0.19630896	-3.567017061	0.000361068	0.00535655
PITA_000011650	High quality	AT1G23180	MA_10426151g0010	shade<sun	ARM repeat superfamily protein;(source:Araport11)		biological_process_unkno	165.0990339	-0.845839799	0.26113467	-3.239094167	0.0011991	0.014673118
PITA_000011693	High quality	AT2G36770	MA_10427810g0010	shade<sun	UDP-Glycosyltransferase superfamily protein;(source:Arap		chloroplast-GO:0009507,tr	3590.626658	-1.927242099	0.40894947	-4.712665545	2.44E-06	6.40E-05
PITA_000011723	High quality	AT2G28470	MA_10428400g0010	shade>sun	putative beta-galactosidase (B(	BETA-GALACTOSIDASE 8 (	beta-galactosidase activity	714.7008091	1.998645342	0.71326267	2.802116833	0.005076848	0.046206466
PITA_000011808	High quality	AT1G11530	MA_164160g0010	shade>sun	Encodes a monocysteinic thior	C-TERMINAL CYSTEINE RE	cytoplasm-GO:0005737,cy	37.41205991	1.33479695	0.39569263	3.373317706	0.000742682	0.009832253
PITA_000011863	High quality	AT5G64040	MA_348255g0010	shade<sun	Encodes the only subunit of ph	(PSAN)	calmodulin binding-GO:00	585.2709698	-0.592693519	0.15773302	-3.757574234	0.000171568	0.002798808
PITA_000012081	Low quality	AT1G64660	MA_611684g0010	shade>sun	Encodes a functional methionii	METHIONINE GAMMA-LYA	carbon-sulfur lyase activity	31.8080563	1.395097725	0.31638373	4.409511552	1.04E-05	0.000238185
PITA_000012112	High quality	AT5G09970	MA_137345g0010	shade>sun	Member of CYP78A family. Par	CYTOCHROME P450, FAM	heme binding-GO:002003	24.19535499	1.861165039	0.64915381	2.867063266	0.004143002	0.039548261
PITA_000012120	High quality	AT3G23330	MA_175688g0010	shade<sun	Tetratricopeptide repeat (TPR)-like superfamily protein;(s		intracellular membrane-bo	451.1753356	-1.729204778	0.13456887	-12.84996089	8.61E-38	1.09E-34
PITA_000012121	High quality	AT3G12770	MA_10287590g0010	shade<sun	Encodes a pentatricopeptide re	MITOCHONDRIAL EDITING	chloroplast-GO:0009507,m	325.5302906	-1.339899073	0.1709015	-7.840183021	4.50E-15	5.59E-13
PITA_000012128	High quality	AT3G01480	MA_7732g0010	shade<sun	Encodes a chloroplast cyclophi	CYCLOPHILIN 38 (CYP38)	chloroplast-GO:0009507,c	383.8643645	-1.130501863	0.17445955	-6.480022803	9.17E-11	5.08E-09
PITA_000012130	Low quality	AT3G56570	MA_10435993g0010	shade<sun	SET domain-containing protein;(source:Araport11)		nucleus-GO:0005634,pept	157.4602134	-1.109138923	0.32420094	-3.421146584	0.000623577	0.008500873
PITA_000012211	High quality	AT3G22550	MA_10432609g0010	shade<sun	NAD(P)H-quinone oxidoreductase subunit, putative (DUF5		cellular response to hypox	295.0569758	-0.648456065	0.19177024	-3.381421809	0.000721118	0.00959208
PITA_000012239	Low quality	AT5G08390	MA_97731g0010	shade>sun	One of four katanin p80 subun	KATANIN P80 SUBUNIT 3 (	cortical microtubule organ	184.4917858	10.82344117	3.90697117	2.770289491	0.005600649	0.049439401
PITA_000012279	High quality	AT1G25210	MA_10433938g0010	shade<sun	UDP-3-O-acyl N-acetylglycosam	LIPID X C5 (LPXC5)	chloroplast-GO:0009507,li	100.0072413	-0.921166005	0.32119007	-2.867977837	0.004131045	0.039480235
PITA_000012316	Low quality	AT3G04930	MA_203641g0010	shade>sun	DNA-binding storekeeper protein-related transcriptional r		nucleus-GO:0005634,prote	70.74035852	0.787054352	0.27022324	2.912607977	0.003584243	0.03537949
PITA_000012416	High quality	AT5G66390	MA_140596g0010	shade<sun	Encodes a peroxidase that is in	PEROXIDASE 72 (PRX72)	extracellular region-GO:00	156.6222724	-1.146695073	0.19716688	-5.815860585	6.03E-09	2.46E-07
PITA_000012455	Low quality	AT1G63610	MA_106317g0010	shade<sun	hypothetical protein;(source:Araport11)		chloroplast-GO:0009507,c	144.2970789	-1.117926401	0.35858263	-3.11762563	0.001823142	0.020786459
PITA_000012484	High quality	AT1G11080	MA_769g0010	shade<sun	serine carboxypeptidase-like 3	SERINE CARBOXYPEPTIDA	extracellular region-GO:00	233.784096	-0.913633119	0.30298603	-3.015429898	0.002566153	0.027249838
PITA_000012496	High quality	AT3G23400	MA_10429604g0010	shade<sun	Encodes FIBRILLIN 4 (FIB4). Th	FIBRILLIN 4 (FIB4)	chloroplast-GO:0009507,c	449.9777779	-1.35987935	0.23757258	-5.724058523	1.04E-08	4.05E-07
PITA_000012498	High quality	AT2G34590	MA_118815g0010	shade<sun	Transketolase family protein;(source:Araport11)		acetyl-CoA biosynthetic pr	88.48091895	-0.58749649	0.20413229	-2.878018473	0.004001817	0.038561622
PITA_000012501	High quality	AT1G75620	MA_812690g0010	shade<sun	glyoxal oxidase-related protein;(source:Araport11)		biological_process_unkno	819.9675734	-0.460345162	0.08275557	-5.56270928	2.66E-08	9.81E-07
PITA_000012530	High quality	AT1G06820	MA_10436461g0010	shade<sun	Encodes carotenoid isomerase	CAROTENOID ISOMERASE	carotenoid biosynthetic pr	919.6794011	-1.018897902	0.20798775	-4.898836219	9.64E-07	2.72E-05
PITA_000012619	Low quality	AT1G35180	MA_10436507g0010	shade>sun	TRAM, LAG1 and CLN8 (TLC) lipid-sensing domain contain		biological_process_unkno	114.5395928	0.563869114	0.19168385	2.941662068	0.00326456	0.032971155
PITA_000012622	Low quality	AT3G60370	MA_170550g0010	shade<sun	Encodes an immunophilin, FKBP20-2, that belongs to the		chloroplast-GO:0009507,c	771.5040141	-1.569269677	0.08532757	-18.39112067	1.55E-75	9.76E-72
PITA_000012682	High quality	AT5G15180	MA_183305g0010	shade>sun	Peroxidase superfamily proteir	PEROXIDASE 56 (PRX56)	extracellular region-GO:00	46.84222304	0.840499599	0.27514438	3.054758394	0.002252419	0.024719759
PITA_000012746	High quality	AT3G43540	MA_10098847g0010	shade<sun	initiation factor 4F subunit (DUF1350);(source:Araport11)		chloroplast-GO:0009507,e	1431.258089	-1.750070958	0.07624915	-22.95200491	1.41E-116	3.55E-112
PITA_000012747	High quality	AT3G08900	MA_162652g0010	shade<sun	RGP3 is a UDP-arabinose muta	REVERSIBLY GLYCOSYLATE	cytoplasm-GO:0005737,cy	99.44776461	-0.481685043	0.16463814	-2.925719576	0.003436606	0.034351601
PITA_000012766	High quality	AT1G79460	MA_57103g0010	shade<sun	Encodes for a protein with ent	GA REQUIRING 2 (GA2)	chloroplast-GO:0009507,c	713.4087148	-1.671567587	0.29297118	-5.705570095	1.16E-08	4.49E-07
PITA_000012787	High quality	AT4G27990	MA_961483g0010	shade<sun	One of four Arabidopsis homol	(YLMG1-2)	chloroplast-GO:0009507,c	156.6838747	-0.676634358	0.157402	-4.298765936	1.72E-05	0.000371172
PITA_000012805	High quality	AT1G60710	MA_40561g0010	shade<sun	Encodes ATB2.	(ATB2)	cytoplasm-GO:0005737,cy	302.4906337	-1.269592545	0.14731258	-8.618357947	6.79E-18	1.36E-15
PITA_000012959	High quality	AT3G15810	MA_10431743g0010	shade>sun	LURP-one-like protein (DUF567);(source:Araport11)		cytoplasm-GO:0005737,cy	34.31891262	2.368136257	0.77663044	3.049244709	0.002294175	0.025014879
PITA_000012964	High quality	AT2G38640	MA_10428043g0010	shade>sun	LURP-one-like protein (DUF567);(source:Araport11)		molecular_function_unkno	46.21100394	1.385818556	0.30974684	4.474036184	7.68E-06	0.000181766
PITA_000013003	High quality	AT2G36970	MA_111005g0010	shade>sun	UDP-Glycosyltransferase superfamily protein;(source:Arap		transferase activity, transfe	179.7822762	10.78605233	1.36991844	7.873499649	3.45E-15	4.37E-13
PITA_000013005	Low quality	AT2G29180	MA_20436g0010	shade<sun	transmembrane protein;(source:Araport11)		biological_process_unkno	181.2657391	-0.520470784	0.17154179	-3.034075758	0.002412738	0.02599285
PITA_000013038	High quality	AT1G33970	MA_153116g0010	shade<sun	IAN9 is a member of a small fa	IMMUNE ASSOCIATED NU	mitochondrion-GO:00057	102.4151539	-1.013211465	0.32636154	-3.104567572	0.001905574	0.021579962
PITA_000013063	High quality	AT4G33150	MA_394353g0010	shade>sun	This is a splice variant of the Lk	SACCHAROPINE DEHYDRO	cytoplasm-GO:0005737,cy	38.11027506	0.744245758	0.25693483	2.896632422	0.003771914	0.036899796
PITA_000013170	Low quality	AT2G18160	MA_35014g0010	shade>sun	Encodes a b-ZIP transcription f	BASIC LEUCINE-ZIPPER 2 (	DNA-binding transcription	26.95619332	0.87828582	0.29518079	2.975416638	0.002925909	0.030354346
PITA_000013260	Low quality	AT1G69390	MA_10433395g0010	shade<sun	Encodes an Arabidopsis homol	HOMOLOGUE OF BACTERI	ATPase binding-GO:00511	190.2845353	-0.506756239	0.159294	-3.181263887	0.00146634	0.017378334
PITA_000013278	High quality	AT3G63310	MA_117923g0010	shade>sun	Mediates cell elongation in bra	BRZ-INSENSITIVE-LONG H	brassinosteroid mediated	46.4523569	0.904794949	0.29778259	3.038441367	0.002378054	0.025696022
PITA_000013281	High quality	AT4G29080	MA_62134g0010	shade>sun	phytochrome-associated prote	PHYTOCHROME-ASSOCIAT	DNA-binding transcription	45.56830866	0.639997258	0.22495131	2.845047957	0.004440474	0.04177126
PITA_000013363	High quality	AT2G03890	MA_2516g0010	shade>sun	Phosphoinositide kinase which	PHOSPHOINOSITIDE 4-KIN	1-phosphatidylinositol 4-k	24.02287334	1.032102805	0.32081948	3.217082748	0.001295012	0.01569448
PITA_000013390	High quality	AT1G55850	MA_10436687g0010	shade<sun	encodes a protein similar to ce	CELLULOSE SYNTHASE LIK	cellulose biosynthetic proc	185.8677626	-1.165778335	0.16424289	-7.097892389	1.27E-12	1.05E-10
PITA_000013409	High quality	AT4G28660	MA_18349g0010	shade<sun	Similar to PsbW subunit of pho	PHOTOSYSTEM II REACTIC	chloroplast-GO:0009507,c	771.8944571	-1.183310902	0.16058171	-7.368901945	1.72E-13	1.62E-11
PITA_000013423	High quality	AT4G23930	MA_254400g0010	shade>sun	Late embryogenesis abundant (LEA) hydroxyproline-rich g		biological_process_unkno	39.11979707	1.28829105	0.29255234	4.403625926	1.06E-05	0.000243633
PITA_000013474	High quality	AT3G15680	MA_101037g0010	shade>sun	Ran BP2/NZF zinc finger-like superfamily protein;(source:/		mRNA binding-GO:000372	40.74036603	1.270473471	0.2912577	4.3620254	1.29E-05	0.000287861
PITA_000013640	High quality	AT1G02660	MA_75319g0010	shade>sun	PLIP2 is a glycerolipid A1 lipas	PLASTID LIPASE2 (PLIP2)	1-18:1-2-16:0-monogalact	46.29573576	0.786480465	0.25621309	3.069634232	0.002143211	0.023717321
PITA_000013689	High quality	AT1G19230	MA_5424284g0010	shade>sun	Riboflavin synthase-like superf	(ATRBOHE)	calcium ion binding-GO:00	85.66387492	1.592511234	0.5386466	2.956504734	0.003111475	0.031756936
PITA_000013692	Low quality	AT1G64430	MA_626674g0010	shade<sun	Pentatricopeptide repeat (PPR) superfamily protein;(sour		biological_process_unkno	298.1437272	-0.864689252	0.21496087	-4.02254258	5.76E-05	0.00107353
PITA_000013730	High quality	AT4G16260	MA_100289g0010	shade<sun	Encodes a putative beta-1,3-endoglucanase that interacts		anchored component of p	117.6521465	-2.84043299	0.91068872	-3.118994357	0.001814694	0.020708865
PITA_000013737	High quality	AT4G35250	MA_10437102g0010	shade<sun	HCF244 is a member of the aty	HIGH CHLOROPHYLL FLUO	chloroplast-GO:0009507,c	845.54053	-1.026295069	0.19169461	-5.353802515	8.61E-08	2.90E-06
PITA_000013860	High quality	AT5G42130	MA_10426415g0010	shade<sun	Encodes a protein belonging tc	MITOFERRINLIKE1 (Mfl1)	chloroplast-GO:0009507,c	122.5655018	-0.611930374	0.19614505	-3.11978498	0.001809831	0.020662715
PITA_000013954	Low quality	AT3G02660	MA_766311g0010	shade<sun	Tyrosyl-tRNA synthetase, class	EMBRYO DEFECTIVE 2768	chloroplast-GO:0009507,c	341.0503768	-1.104302161	0.15625285	-7.067404714	1.58E-12	1.26E-10
PITA_000013956	High quality	AT5G51890	MA_750042g0010	shade<sun	encodes peroxidase involved ir	PEROXIDASE 66 (PRX66)	cellular oxidant detoxificat	156.4848458	-2.767095021	0.76579523	-3.613361511	0.000302253	0.004586532
PITA_000014004	High quality	AT2G41790	MA_7967391g0010	shade<sun	Insulinase (Peptidase family M16) family protein;(source:/		cytoplasm-GO:0005737,cy	48.3145984	-9.223932719	1.2334423	-7.478203651	7.53E-14	7.72E-12
PITA_000014083	High quality	AT4G03200	MA_10429547g0010	shade<sun	catalytics;(source:Araport11)		carbohydrate metabolic pr	104.3548442	-0.710543126	0.16648596	-4.267886254	1.97E-05	0.000420335
PITA_000014115	High quality	AT5G67030	MA_748776g0010	shade<sun	Encodes a single copy zeaxanth	ABA DEFICIENT 1 (ABA1)	abscisic acid biosynthetic p	336.9595105	-0.618432762	0.1505008	-4.10916584	3.97E-05	0.000779349
PITA_000014201	Low quality	AT1G78995	MA_10426769g0020	shade<sun	hypothetical protein;(source:Araport11)		biological_process_unkno	261.5845993	-1.330822471	0.22390921	-5.943580719	2.79E-09	1.20E-07
PITA_000014294	High quality	AT2G13600	MA_164547g0010	shade<sun	Encodes a pentatricopeptide re	SLOW GROWTH 2 (SLO2)	chloroplast-GO:0009507,ir	222.7321012	-1.122592087	0.2111264	-5.31715646	1.05E-07	3.49E-06
PITA_000014365	High quality	AT2G20920	MA_10437095g0010	shade<sun	chaperone (DUF3353);(source:Araport11)		chloroplast-GO:0009507	251.3029629	-0.77192939	0.16351375	-4.720883532	2.35E-06	6.18E-05
PITA_000014421	High quality	AT4G16442	MA_8167318g0010	shade>sun	Uncharacterized protein family	CASP-LIKE PROTEIN 2B1 ((	plasma membrane-GO:00(	57.2115606	1.373696909	0.34063143	4.032795485	5.51E-05	0.001035441
PITA_000014450	High quality	AT4G09650	MA_657g0010	shade<sun	Encodes the chloroplast ATPas	ATP SYNTHASE DELTA-SUI	ATP synthesis coupled pro	435.1657157	-1.001680048	0.18233454	-5.49363857	3.94E-08	1.42E-06
PITA_000014471	High quality	AT1G53210	MA_10281743g0010	shade>sun	Encodes a Na+/Ca 2+ exchange	NA+/CA2+ EXCHANGER (N	calcium ion binding-GO:00	22.99668065	1.608551176	0.44196975	3.639505163	0.000273162	0.004208404
PITA_000014485	Low quality	AT3G54500	MA_10428838g0010	shade<sun	Member of a small family (4 pr	NIGHT LIGHT-INDUCIBLE A	circadian regulation of ger	345.9347443	-0.870943311	0.1227651	-7.094388407	1.30E-12	1.07E-10
PITA_000014554	High quality	AT4G18210	MA_9508421g0010	shade>sun	Member of a family of proteins	PURINE PERMEASE 10 (PU	cytoplasm-GO:0005737,m	113.7389728	10.12550048	1.55782572	6.499764599	8.04E-11	4.51E-09
PITA_000014574	Low quality	AT2G15490	MA_10426914g0010	shade<sun	UDP-glycosyltransferase 73B4;	UDP-GLYCOSYLTRANSFER/	chloroplast-GO:0009507,c	1759.676953	-5.935525965	0.80504525	-7.372909765	1.67E-13	1.58E-11
PITA_000014645	High quality	AT4G18240	MA_10428935g0010	shade<sun	Encodes a starch synthase. In	STARCH SYNTHASE 4 (SS4)	alpha-1,4-glucan synthase	123.8590487	-0.941614849	0.27905847	-3.374256434	0.000740154	0.009803928
PITA_000014815	High quality	AT4G34860	MA_7394g0010	shade<sun	Plant neutral invertase family p	ALKALINE/NEUTRAL INVEF	cytosol-GO:0005829,glyco	174.121736	-0.82491342	0.15412504	-5.352235051	8.69E-08	2.93E-06
PITA_000014878	Low quality	AT1G74670	MA_7575249g0010	shade>sun	Gibberellin-regulated family pr	GA-STIMULATED ARABIDC	extracellular region-GO:00	40.02919154	1.441889829	0.32336324	4.459040659	8.23E-06	0.000192965
PITA_000014959	Low quality	AT4G24210	MA_233275g0010	shade>sun	F-box protein that is involved i	SLEEPY1 (SLY1)	gibberellic acid mediated s	35.26622744	1.89688648	0.33598918	5.645677346	1.65E-08	6.25E-07
PITA_000015004	High quality	AT2G24840	MA_195511g0010	shade<sun	Encodes a member of the Agar	AGAMOUS-LIKE 61 (AGL61	DNA-binding transcription	416.5163383	-2.432739063	0.74121566	-3.282093438	0.001030395	0.012935068
PITA_000015048	Low quality	AT3G62910	MA_389025g0010	shade<sun	Encodes a plastid-localized ribc	ALBINO AND PALE GREEN	chloroplast-GO:0009507,c	383.2003746	-0.775055093	0.25154353	-3.081196729	0.002061704	0.02301734
PITA_000015134	High quality	AT2G32830	MA_10434434g0020	shade<sun	Encodes Pht1;5, a member of t	PHOSPHATE TRANSPORTE	cytoplasm-GO:0005737,in	2244.71253	-1.929194206	0.41951194	-4.598663382	4.25E-06	0.000106387
PITA_000015178	High quality	AT4G15510	MA_81231g0010	shade<sun	A nuclear-encoded thylakoid lu	PSBP-DOMAIN PROTEIN1	calcium ion binding-GO:00	1211.55113	-1.574771499	0.18298512	-8.606008304	7.56E-18	1.49E-15
PITA_000015241	High quality	AT5G13650	MA_138810g0010	shade<sun	Encodes SVR3, a putative chlor	SUPPRESSOR OF VARIEGA	chloroplast-GO:0009507,c	440.7317979	-1.422151866	0.10887133	-13.062685	5.38E-39	8.48E-36
PITA_000015247	High quality	AT3G29575	MA_2575g0010	shade>sun	ABI five binding protein 3;(sou	ABI FIVE BINDING PROTEI	negative regulation of trar	11.52144142	2.417620828	0.56085559	4.310594127	1.63E-05	0.000353376
PITA_000015282	Low quality	AT1G19800	MA_195852g0010	shade<sun	Encodes a permease-like prote	ATP-BINDING CASSETTE I1	ATP-binding cassette (ABC	107.8317837	-0.567008745	0.14682893	-3.861696383	0.000112602	0.001936012
PITA_000015343	Low quality	AT1G79850	MA_10123473g0010	shade<sun	nuclear-encoded 30S chloropla	RIBOSOMAL PROTEIN S17	chloroplast-GO:0009507,c	355.1296468	-0.838696704	0.16292555	-5.147729705	2.64E-07	8.12E-06
PITA_000015357	Low quality	AT5G13510	MA_483364g0010	shade<sun	Ribosomal protein L10 family p	EMBRYO DEFECTIVE 3136	chloroplast-GO:0009507,c	474.1748634	-0.958552589	0.18680507	-5.131298573	2.88E-07	8.82E-06
PITA_000015366	Low quality	AT3G46640	MA_303578g0010	shade>sun	Encodes a myb family transcrip	PHYTOCLOCK 1 (PCL1)	circadian rhythm-GO:0007	43.78513661	1.124084389	0.28034567	4.00963704	6.08E-05	0.001126879
PITA_000015384	Low quality	AT1G06550	MA_105804g0010	shade<sun	ATP-dependent caseinolytic (Clp) protease/crotonase fam		3-hydroxyisobutyryl-CoA h	145.11395	-0.835498271	0.22254977	-3.754208566	0.00017389	0.002833015
PITA_000015388	Low quality	AT2G36320	MA_91228g0010	shade>sun	A20/AN1-like zinc finger family protein;(source:Araport11		biological_process_unkno	36.73895037	8.496694381	1.36206901	6.238079212	4.43E-10	2.16E-08
PITA_000015402	High quality	AT4G28250	MA_21214g0010	shade>sun	putative beta-expansin/allerge	EXPANSIN B3 (EXPB3)	extracellular region-GO:00	31.58613593	0.932526365	0.2809172	3.319577367	0.000901538	0.011594489
PITA_000015521	High quality	AT1G50170	MA_10432910g0010	shade<sun	encodes sirohydrochlorin ferrc	SIROHYDROCHLORIN FERF	chloroplast-GO:0009507,e	232.885015	-0.802360054	0.18569521	-4.320844198	1.55E-05	0.000339421
PITA_000015551	Low quality	AT5G14170	MA_10426077g0010	shade>sun	CHC1 is predicted to encode a	(CHC1)	DNA repair-GO:0006281,D	50.75587075	0.618973769	0.22157188	2.793557432	0.005213177	0.047006195
PITA_000015574	Low quality	AT4G32870	MA_37077g0020	shade>sun	Polyketide cyclase/dehydrase and lipid transport superfar		abscisic acid binding-GO:0	30.19043854	1.036248265	0.34761027	2.981063462	0.002872493	0.029910926
PITA_000015580	Low quality	AT3G14920	MA_10379445g0010	shade<sun	Peptide-N4-(N-acetyl-beta-glucosaminyl)asparagine amida		biological_process_unkno	118.8407302	-0.501387915	0.14855923	-3.375003434	0.000738148	0.009787637
PITA_000015598	High quality	AT3G21200	MA_386726g0010	shade<sun	Encodes a soluble glutamyl-tRI	PROTON GRADIENT REGU	chloroplast-GO:0009507,c	253.3016653	-0.541103911	0.17745516	-3.04924307	0.002294188	0.025014879
PITA_000015709	High quality	AT5G27390	MA_811505g0010	shade<sun	tagatose-6-phosphate ketose/aldose isomerase, putative (		calcium ion binding-GO:00	270.3679976	-0.476188327	0.1547089	-3.077963442	0.002084205	0.023217158
PITA_000015787	Low quality	AT5G36110	MA_896041g0010	shade<sun	Encodes a member of the CYP7	CYTOCHROME P450, FAM	extracellular region-GO:00	184.7347271	-0.672190527	0.16195928	-4.15036747	3.32E-05	0.000665996
PITA_000015794	High quality	AT3G17970	MA_10432470g0010	shade<sun	Integral chloroplast outer mem	TRANSLOCON AT THE OUT	chloroplast-GO:0009507,c	132.9573264	-1.08033577	0.19600321	-5.511827022	3.55E-08	1.29E-06
PITA_000015880	High quality	AT1G36370	MA_10428694g0010	shade<sun	Encodes a nuclear localised prc	MORE SULPHUR ACCUMU	chloroplast-GO:0009507,c	714.8830919	-1.675589352	0.20580616	-8.141589699	3.90E-16	6.11E-14
PITA_000016180	High quality	AT3G06060	MA_29607g0010	shade>sun	Encodes one of the Arabidopsi	TSC10A (TSC10A)	3-dehydrosphinganine red	84.05192082	1.030379764	0.28345217	3.635109761	0.000277862	0.004270376
PITA_000016220	Low quality	AT5G22870	MA_27406g0010	shade>sun	Late embryogenesis abundant (LEA) hydroxyproline-rich g		biological_process_unkno	67.6135795	0.832265605	0.28936379	2.876191299	0.004025057	0.03871546
PITA_000016229	Low quality	AT5G52970	MA_37709g0010	shade<sun	thylakoid lumen 15.0 kDa protein;(source:Araport11)		biological_process_unkno	367.1022564	-0.72333424	0.1269447	-5.698026373	1.21E-08	4.67E-07
PITA_000016305	High quality	AT1G80920	MA_10431645g0020	shade<sun	A nuclear encoded soluble pro	(J8)	chloroplast-GO:0009507,c	397.2591391	-0.826232781	0.25506705	-3.239276875	0.001198332	0.014670839
PITA_000016338	High quality	AT4G35760	MA_10432388g0010	shade<sun	Encodes a bimodular enzyme c	LUMEN THIOL OXIDOREDU	chloroplast-GO:0009507,N	228.9279639	-0.942679656	0.24962332	-3.776408622	0.000159106	0.0026278
PITA_000016482	High quality	AT1G65450	MA_56269g0010	shade>sun	Contains dual transcription uni	GLAUCE (GLC)	cytoplasm-GO:0005737,dc	52.34420825	1.37764397	0.38289272	3.597989487	0.000320687	0.00483715
PITA_000016508	High quality	AT1G78530	MA_10435581g0010	shade>sun	Protein kinase superfamily protein;(source:Araport11)		phosphorylation-GO:0016	63.91991536	0.807314255	0.25117603	3.214137282	0.001308371	0.015841151
PITA_000016536	High quality	AT2G28470	MA_87238g0010	shade<sun	putative beta-galactosidase (B(	BETA-GALACTOSIDASE 8 (	beta-galactosidase activity	505.6130845	-1.949862115	0.12099837	-16.11478046	2.01E-58	6.33E-55
PITA_000016538	High quality	AT3G10420	MA_10427944g0010	shade<sun	P-loop containing nucleoside tı	SEEDLING PLASTID DEVELI	ATPase activity-GO:00168	433.5879046	-1.121276916	0.11082978	-10.11710834	4.64E-24	1.83E-21
PITA_000016543	Low quality	AT1G06440	MA_118270g0010	shade<sun	Ubiquitin carboxyl-terminal hydrolase family protein;(sou		chloroplast-GO:0009507	97.78242201	-1.06764244	0.2575312	-4.145681934	3.39E-05	0.000678684
PITA_000016546	High quality	AT5G05390	MA_10191230g0010	shade>sun	putative laccase, a member of	LACCASE 12 (LAC12)	copper ion binding-GO:00	38.14623096	2.507900073	0.5954937	4.211463627	2.54E-05	0.000526222
PITA_000016602	High quality	AT1G17840	MA_10436774g0010	shade>sun	Encodes a plasma membrane-l	ATP-BINDING CASSETTE G	ATPase activity-GO:00168	14.53569062	2.377319142	0.57352313	4.145114708	3.40E-05	0.000679826
PITA_000016722	High quality	AT1G65060	MA_109119g0010	shade<sun	encodes an isoform of 4-coumi	4-COUMARATE:COA LIGAS	4-coumarate-CoA ligase ac	159.2305664	-1.183246059	0.17855507	-6.626785111	3.43E-11	2.08E-09
PITA_000016742	High quality	AT3G13890	MA_10431212g0010	shade>sun	Encodes a putative transcriptic	MYB DOMAIN PROTEIN 26	anther dehiscence-GO:000	133.7405528	0.78164169	0.13933889	5.609645008	2.03E-08	7.63E-07
PITA_000016872	High quality	AT1G08550	MA_10426812g0010	shade<sun	Violaxanthin deepoxidase invo	NON-PHOTOCHEMICAL Q	chlorophyll metabolic proc	796.8106125	-1.422236164	0.17484295	-8.134363612	4.14E-16	6.33E-14
PITA_000016891	High quality	AT4G09750	MA_8052156g0010	shade<sun	NAD(P)-binding Rossmann-fold superfamily protein;(sourc		chloroplast-GO:0009507,c	296.458906	-0.741180261	0.16419311	-4.514076594	6.36E-06	0.00015289
PITA_000016905	High quality	AT4G02750	MA_10431508g0010	shade<sun	Tetratricopeptide repeat (TPR)	(SSTPR)	acquisition of seed longev	269.2568782	-1.805724546	0.33954192	-5.318119662	1.05E-07	3.48E-06
PITA_000016907	High quality	AT2G29760	MA_8607116g0010	shade<sun	Encodes a chloroplast RNA edi	ORGANELLE TRANSCRIPT I	chloroplast-GO:0009507,c	426.5352977	-0.866013331	0.28541606	-3.034213773	0.002411635	0.025992064

PITA_000016994	High quality	AT3G14360	MA_208494g0010	shade>sun	alpha/beta-Hydrolases superfa	OIL BODY LIPASE 1 (ATOB	extracellular region-GO:00	175.6115636	1.075430062	0.1924097	5.589271465	2.28E-08	8.51E-07
PITA_000017013	High quality	AT1G77280	MA_121123g0010	shade>sun	kinase with adenine nucleotide	alpha hydrolases-like dom	cytoplasm-GO:0005737,ph	60.94720017	0.731285195	0.22934589	3.188568935	0.001429789	0.017049306
PITA_000017028	High quality	AT2G20860	MA_10428008g0010	shade<sun	LIP1,Lipoic acid synthase,	LIPOIC ACID SYNTHASE 1 (	chloroplast-GO:0009507,g	136.2575525	-0.647975239	0.14272311	-4.540086426	5.62E-06	0.000137152
PITA_000017071	High quality	AT4G01650	MA_202526g0010	shade<sun	Polyketide cyclase / dehydrase	and lipid transport protein	biological_process_unkno	186.9973563	-0.901562633	0.16445721	-5.482049953	4.20E-08	1.51E-06
PITA_000017080	High quality	AT3G54890	MA_10431493g0010	shade<sun	Encodes a component of the li	PHOTOSYSTEM I LIGHT HA	chlorophyll binding-GO:00	1750.271395	-0.883027688	0.29562081	-2.987028129	0.002817038	0.029455099
PITA_000017122	High quality	AT5G10460	MA_204656g0010	shade<sun	Haloacid dehalogenase-like hydrolase (HAD) superfamily p		chloroplast-GO:0009507,c	209.2289443	-0.894303747	0.24869138	-3.596038337	0.0003231	0.004867734
PITA_000017127	High quality	AT5G13930	MA_10426264g0020	shade<sun	Encodes chalcone synthase (CH	TRANSPARENT TESTA 4 (T	auxin polar transport-GO:(	119.8259105	-1.224899167	0.24740718	-4.950944232	7.39E-07	2.12E-05
PITA_000017131	High quality	AT4G25050	MA_10429573g0020	shade<sun	encodes an acyl carrier protein	ACYL CARRIER PROTEIN 4	acyl carrier activity-GO:00(	183.3143208	-0.678917872	0.18218061	-3.726619893	0.000194065	0.003115413
PITA_000017212	High quality	AT2G15970	MA_95456g0010	shade>sun	encodes an alpha form of a pro	COLD REGULATED 413 PLA	cellular response to water	58.29016782	0.655712007	0.21141103	3.101597897	0.001924792	0.021774058
PITA_000017254	High quality	AT3G51240	MA_10435304g0010	shade<sun	Encodes flavanone 3-hydroxyla	FLAVANONE 3-HYDROXYL	cytoplasm-GO:0005737,di	158.5980246	-10.9391386	3.90707702	-2.799826712	0.005113005	0.046404367
PITA_000017336	Low quality	AT5G63040	MA_10435802g0010	shade<sun	transmembrane protein;(source:Araport11)		biological_process_unkno	185.6015656	-0.925138552	0.27147186	-3.40786165	0.000654741	0.008849177
PITA_000017502	High quality	AT5G60540	MA_10432986g0010	shade<sun	Encodes a protein predicted to	PYRIDOXINE BIOSYNTHESI	cytoplasm-GO:0005737,cy	144.0559919	-0.91761948	0.19137019	-4.794996964	1.63E-06	4.38E-05
PITA_000017576	Low quality	AT2G21970	MA_118719g0010	shade<sun	stress enhanced protein 2 (SEP	STRESS ENHANCED PROTE	cellular response to high li	334.9662216	-0.955500659	0.19097749	-5.003210811	5.64E-07	1.65E-05
PITA_000017588	High quality	AT1G15520	MA_10425791g0020	shade<sun	ABC transporter family involve	ATP-BINDING CASSETTE G	abscisic acid transport-GO	158.5980246	-10.9391386	3.90707702	-2.799826712	0.005113005	0.046404367
PITA_000017693	High quality	AT2G03200	MA_10431950g0020	shade<sun	Atypical aspartic protease whic	ATYPICAL ASPARTIC PROT	extracellular region-GO:00	243.5826143	-11.55894399	1.23031131	-9.395137581	5.71E-21	1.57E-18
PITA_000017769	High quality	AT1G09850	MA_22695g0010	shade<sun	Arabidopsis thaliana papain-lik	XYLEM BARK CYSTEINE PE	cysteine-type endopeptida	296.5653092	-1.657441601	0.22949939	-7.221986915	5.12E-13	4.55E-11
PITA_000017848	High quality	AT4G13420	MA_6064g0010	shade<sun	Encodes a protein of the KUP/I	HIGH AFFINITY K+ TRANSF	membrane-GO:0016020,n	53.15496325	-9.364186492	1.32813919	-7.050606281	1.78E-12	1.40E-10
PITA_000017885	High quality	AT1G10020	MA_25897g0010	shade>sun	formin-like protein (DUF1005);(source:Araport11)		biological_process_unkno	35.63777776	1.011883316	0.26375745	3.836416017	0.000124843	0.002111695
PITA_000017976	Low quality	AT1G11050	MA_10435959g0010	shade>sun	Protein kinase superfamily protein;(source:Araport11)		chloroplast-GO:0009507,k	83.86958295	9.68668751	1.57100596	6.165913903	7.01E-10	3.31E-08
PITA_000018010	High quality	AT3G25560	MA_20016g0010	shade<sun	NSP-interacting kinase 2;(sourc	NSP-INTERACTING KINASE	plasma membrane-GO:00(	252.0040568	-1.035999128	0.15714102	-6.592798927	4.32E-11	2.57E-09
PITA_000018011	High quality	AT1G15110	MA_459670g0010	shade>sun	PSS1 encodes a base-exchange	PHOSPHATIDYLSERINE SYI	CDP-diacylglycerol-serine (	28.29231867	1.185150859	0.30337797	3.906515896	9.36E-05	0.00164795
PITA_000018047	High quality	AT1G48420	MA_9349051g0010	shade<sun	Encodes an enzyme that decor	D-CYSTEINE DESULFHYDR/	1-aminocyclopropane-1-ca	104.2450027	-0.576606636	0.17191675	-3.35398759	0.000796559	0.010425238
PITA_000018068	High quality	AT1G12900	MA_63231g0010	shade>sun	glyceraldehyde 3-phosphate de	GLYCERALDEHYDE 3-PHOS	apoplast-GO:0048046,chlc	61.87004186	0.688722185	0.23450792	2.936882433	0.003315298	0.033364647
PITA_000018086	High quality	AT3G55740	MA_184924g0010	shade>sun	Encodes a proline transporter	PROLINE TRANSPORTER 2	amino acid transmembran	75.86169084	0.768170382	0.22373564	3.433384077	0.000596097	0.008197147
PITA_000018156	Low quality	AT1G30450	MA_843476g0010	shade<sun	member of Cation-chloride co-	CATION-CHLORIDE CO-TR/	cation:chloride symporter	118.4272487	-0.654392087	0.20147218	-3.248051858	0.001161981	0.014302176
PITA_000018168	Low quality	AT2G28720	MA_10428006g0010	shade<sun	Histone superfamily protein;(s	(HTB3)	DNA binding-GO:0003677,	170.7848929	-0.947402949	0.30201023	-3.136989563	0.001706922	0.019661002
PITA_000018204	High quality	AT1G11290	MA_106123g0010	shade<sun	Pentatricopeptide Repeat Prot	CHLORORESPIRATORY REI	chloroplast-GO:0009507,e	992.7372768	-1.935885922	0.48019074	-4.031493688	5.54E-05	0.00103847
PITA_000018272	High quality	AT1G69500	MA_10428090g0010	shade>sun	Encodes a cytochrome P450, d	CYTOCHROME P450, FAM	alkane 1-monooxygenase	187.6372316	10.84782526	3.90696762	2.776533191	0.005494203	0.048755731
PITA_000018451	High quality	AT2G47850	MA_10435282g0010	shade<sun	Zinc finger C-x8-C-x5-C-x3-H type family protein;(source:A		biological_process_unkno	139.2436555	-0.972704786	0.22424063	-4.337772287	1.44E-05	0.000317309
PITA_000018476	High quality	AT4G03280	MA_733845g0010	shade<sun	Encodes the Rieske FeS center	PHOTOSYNTHETIC ELECTR	chloroplast-GO:0009507,c	921.7183512	-1.281808414	0.16665935	-7.691188156	1.46E-14	1.66E-12
PITA_000018489	Low quality	AT2G25680	MA_155905g0010	shade<sun	Encodes a high-affinity molybd	MOLYBDATE TRANSPORTI	chloroplast-GO:0009507,n	179.8652718	-0.955476304	0.2937295	-3.252912259	0.001142288	0.014121811
PITA_000018490	High quality	AT2G38740	MA_10430307g0010	shade<sun	HAD-type phosphosugar phosp	BROAD-RANGE SUGAR PH	cytoplasm-GO:0005737,cy	132.8792773	-1.712422361	0.17959702	-9.534803641	1.50E-21	4.46E-19
PITA_000018663	High quality	AT4G30110	MA_10434428g0010	shade>sun	encodes a protein similar to Zn	HEAVY METAL ATPASE 2 (	ABC-type cadmium transp	65.98371917	0.702791023	0.21071647	3.335244896	0.000852243	0.011056364
PITA_000018697	High quality	AT2G16630	MA_74117g0010	shade>sun	Pollen Ole e 1 allergen and ext	FUSED OUTER CUTICULAR	extracellular region-GO:00	52.86891489	1.070798112	0.25192088	4.250533443	2.13E-05	0.000449705
PITA_000018731	Low quality	AT2G35260	MA_10435404g0010	shade<sun	CAAX protease self-immunity p	BALANCE OF CHLOROPHY	CAAX-box protein process	188.6446939	-0.445589758	0.13757839	-3.238806346	0.00120031	0.014680809
PITA_000018786	High quality	AT2G37240	MA_10426424g0010	shade<sun	Thioredoxin superfamily protein;(source:Araport11)		antioxidant activity-GO:00	980.0953393	-1.184079419	0.20899268	-5.665650325	1.46E-08	5.60E-07
PITA_000018796	High quality	AT4G26180	MA_10433305g0010	shade<sun	Encodes a mitochondrial CoA t	COA CARRIER 2 (COAC2)	coenzyme A transmembra	543.6617777	-1.3519123	0.21735698	-6.21977866	4.98E-10	2.41E-08
PITA_000018799	Low quality	AT3G07270	MA_102960g0010	shade<sun	GTP cyclohydrolase I;(source:Araport11)		7,8-dihydroneopterin 3'-tr	180.4712721	-0.736417827	0.14956823	-4.923624746	8.50E-07	2.42E-05
PITA_000018800	High quality	AT5G11420	MA_9589g0010	shade<sun	Encodes a DUF642 cell wall protein.		biological_process_unkno	424.202388	-2.123047767	0.59805437	-3.549924372	0.000385342	0.005653474
PITA_000018802	High quality	AT5G25460	MA_54830g0010	shade>sun	Encodes a DUF642 cell wall pro	DUF642 L-GALL RESPONSI	extracellular region-GO:00	34.77130277	1.348701543	0.34611222	3.896717498	9.75E-05	0.001710071
PITA_000018803	High quality	AT2G34510	MA_44504g0020	shade>sun	Protein of unknown function, I	(ATHB-1)	anchored component of m	14.09035254	1.862537525	0.66610834	2.796148016	0.005171571	0.046764797
PITA_000018804	High quality	AT2G35530	MA_30617g0010	shade>sun	Encodes a G group bZIP transc	BASIC REGION/LEUCINE ZI	DNA-binding transcription	28.14984791	1.119907073	0.31189397	3.590665984	0.000329834	0.004948493
PITA_000018900	High quality	AT5G02540	MA_137121g0010	shade>sun	NAD(P)-binding Rossmann-fold	superfamily protein;(sourc	plant organ morphogenes	73.84803824	0.595211239	0.19421468	3.064707819	0.002178827	0.024048152
PITA_000018953	Low quality	AT2G38780	MA_10435866g0010	shade<sun	cytochrome C oxidase subunit;(source:Araport11)		biological_process_unkno	646.5490007	-1.428348645	0.2552194	-5.596551978	2.19E-08	8.18E-07
PITA_000018993	High quality	AT5G45910	MA_76943g0010	shade<sun	GDSL-motif esterase/acyltransferase/lipase. Enzyme grou		hydrolase activity, acting c	1233.072924	-1.845111871	0.32878593	-5.611894174	2.00E-08	7.54E-07
PITA_000019022	High quality	AT5G44000	MA_10433329g0010	shade<sun	Glutathione S-transferase family protein;(source:Araport1		chloroplast-GO:0009507,c	121.494526	-0.867485483	0.22719453	-3.818249855	0.000134402	0.002255231
PITA_000019051	Low quality	AT1G52620	MA_957303g0010	shade<sun	Mitochondrial pentatricopepti	PENTATRICOPEPTIDE REPI	chloroplast-GO:0009507,n	105.0529655	-0.984300066	0.29563261	-3.32947056	0.000870113	0.011253457
PITA_000019085	High quality	AT2G45270	MA_714494g0010	shade<sun	Mitochondrial protein essentia	GLYCOPROTEASE 1 (GCP1)	chloroplast-GO:0009507,E	211.1406056	-0.889570553	0.24592713	-3.617211901	0.000297794	0.004529766
PITA_000019159	High quality	AT1G75450	MA_10433485g0010	shade>sun	This gene used to be called At(	CYTOKININ OXIDASE 5 (CK	cytokinin catabolic process	25.42664633	3.247690579	0.98448343	3.298877852	0.000970721	0.012339513
PITA_000019164	Low quality	AT3G04910	MA_16984g0010	shade<sun	Serine/threonine protein kinas	WITH NO LYSINE (K) KINA	ATP binding-GO:0005524,(	71.00610427	-1.404346477	0.46055259	-3.049264073	0.002294027	0.025014879
PITA_000019252	High quality	AT4G38620	MA_55123g0010	shade>sun	Encodes a R2R3 MYB protein w	MYB DOMAIN PROTEIN 4	regulation of phenylpropa	23.19394518	1.165607008	0.39664977	2.938630206	0.003296662	0.033203596
PITA_000019288	High quality	AT4G19810	MA_126635g0010	shade<sun	ChiC encodes a Class V chitinas	CLASS V CHITINASE (ChiC)	cell wall-GO:0005618,chiti	1291.119173	-1.361415118	0.19134116	-7.115118859	1.12E-12	9.33E-11
PITA_000019302	High quality	AT5G16890	MA_168957g0010	shade<sun	Exostosin family protein;(source:Araport11)		Golgi apparatus-GO:00057	85.9652276	-0.685065632	0.17552285	-3.902999746	9.50E-05	0.001668591
PITA_000019340	High quality	AT5G54250	MA_175873g0010	shade>sun	member of Cyclic nucleotide ga	CYCLIC NUCLEOTIDE-GATE	calmodulin binding-GO:00	32.42818859	1.936302307	0.67302171	2.877028025	0.004014399	0.038639094
PITA_000019422	High quality	AT4G29270	MA_10435570g0010	shade<sun	HAD superfamily, subfamily IIIB	acid phosphatase;(source	acid phosphatase activity-(	5556.072503	-1.038793863	0.20104793	-5.166896532	2.38E-07	7.38E-06
PITA_000019484	High quality	AT1G57720	MA_45916g0010	shade<sun	Translation elongation factor EF1B, gamma chain;(source:		cell wall-GO:0005618,copp	117.8781967	-0.408995404	0.1458375	-2.804459816	0.005040097	0.045968891
PITA_000019549	High quality	AT1G34060	MA_10435420g0040	shade<sun	Pyridoxal phosphate (PLP)-dep	TRYPTOPHAN AMINOTRAI	carbon-sulfur lyase activity	87.19379944	-0.523384192	0.17670757	-2.961866249	0.003057806	0.031318946
PITA_000019626	Low quality	AT4G11910	MA_10433870g0010	shade<sun	Acts antagonistically with SGR1	NONYELLOWING 2 (NYE2)	chloroplast-GO:0009507,n	231.9639167	-0.885527366	0.16329958	-5.422716675	5.87E-08	2.06E-06
PITA_000019629	High quality	AT5G63290	MA_28151g0010	shade<sun	CPO3 (At5g63290) has not bee	(ATHEMN1)	4 iron, 4 sulfur cluster binc	158.9339105	-0.712983656	0.14226853	-5.011534712	5.40E-07	1.59E-05
PITA_000019741	Low quality	AT5G08139	MA_281830g0010	shade>sun	RING/U-box superfamily protein;(source:Araport11)		nucleus-GO:0005634,ubiq	36.57064263	0.83645529	0.25678051	3.257471905	0.001124094	0.013917352
PITA_000019785	High quality	AT2G43760	MA_10201271g0010	shade<sun	molybdopterin biosynthesis M	COFACTOR OF NITRATE RI	cytosol-GO:0005829,molyl	352.2342829	-1.701500932	0.55913397	-3.043100633	0.00234154	0.025443189
PITA_000019802	High quality	AT2G16430	MA_10430628g0010	shade<sun	Encodes an acid phosphatase i	PURPLE ACID PHOSPHATA	acid phosphatase activity-(	213.5131299	-0.927009175	0.15305341	-6.056769106	1.39E-09	6.31E-08
PITA_000019943	Low quality	AT2G03430	MA_64903g0010	shade>sun	Ankyrin repeat family protein;(source:Araport11)		biological_process_unkno	29.91186915	0.94677385	0.31210789	3.033482603	0.002417486	0.026032882
PITA_000020011	High quality	AT5G45930	MA_48250g0010	shade<sun	encodes a second Chl I gene (C	MAGNESIUM CHELATASE	ATPase activity-GO:00168	499.7948074	-0.971921607	0.19261003	-5.04605908	4.51E-07	1.34E-05
PITA_000020037	Low quality	AT2G32850	MA_10428537g0030	shade<sun	Protein kinase superfamily protein;(source:Araport11)		ATP binding-GO:0005524,(	75.02342968	-9.858956513	1.54507076	-6.380909371	1.76E-10	9.29E-09
PITA_000020140	High quality	AT1G23460	MA_21072g0010	shade<sun	Pectin lyase-like superfamily protein;(source:Araport11)		carbohydrate metabolic pı	340.6612246	-1.133634255	0.29003083	-3.908668173	9.28E-05	0.001637912
PITA_000020224	Low quality	AT5G06240	MA_321346g0010	shade>sun	embryo defective 2735;(source	EMBRYO DEFECTIVE 2735	cellular_component_unkn	94.83026494	0.51527611	0.17779197	2.8981968	0.00375315	0.036744737
PITA_000020313	High quality	AT3G07130	MA_618219g0010	shade<sun	Encodes PAP15, a purple acid p	PURPLE ACID PHOSPHATA	acid phosphatase activity-(	334.9474341	-1.20915061	0.25181471	-4.801747418	1.57E-06	4.24E-05
PITA_000020478	High quality	AT3G62240	MA_6264656g0010	shade>sun	RING/U-box superfamily protein;(source:Araport11)		mRNA binding-GO:000372	48.23619313	0.789021271	0.27425761	2.87693481	0.004015585	0.038639094
PITA_000020535	High quality	AT3G20320	MA_10434063g0010	shade<sun	Encodes a permease-like comp	ATP-BINDING CASSETTE I1	chloroplast-GO:0009507,c	124.0918701	-0.612094232	0.17704273	-3.457324869	0.000545567	0.007610173
PITA_000020572	High quality	AT5G21920	MA_9458287g0010	shade<sun	One of four Arabidopsis homol	(YLMG2)	chloroplast fission-GO:001	539.1406191	-0.960947028	0.13916419	-6.905131606	5.02E-12	3.59E-10
PITA_000020728	High quality	AT1G55610	MA_170g0010	shade>sun	mutant has Altered vascular ce	BRI1 LIKE (BRL1)	hormone-mediated signali	25.04274373	1.035159823	0.33193768	3.118536697	0.001817515	0.020731674
PITA_000020909	Low quality	AT1G73885	MA_181270g0010	shade<sun	AT-rich interactive domain protein;(source:Araport11)		biological_process_unkno	307.6968555	-0.96368245	0.13590022	-7.091103108	1.33E-12	1.09E-10
PITA_000021024	High quality	AT1G11080	MA_118714g0010	shade>sun	serine carboxypeptidase-like 3	SERINE CARBOXYPEPTIDA	extracellular region-GO:00	27.65322247	2.838462492	0.65556968	4.329764778	1.49E-05	0.000327638
PITA_000021038	High quality	AT1G54780	MA_10433010g0010	shade<sun	Encodes a thylakoid lumen pro	THYLAKOID LUMEN PROTI	acid phosphatase activity-(	625.4740355	-1.007526467	0.17320402	-5.816992309	5.99E-09	2.45E-07
PITA_000021124	Low quality	AT5G35970	MA_10435849g0030	shade<sun	P-loop containing nucleoside triphosphate hydrolases sup		5'-3' DNA helicase activity-	387.5034908	-1.052523364	0.30112302	-3.495326817	0.000473482	0.006729661
PITA_000021147	High quality	AT2G35840	MA_10432402g0010	shade<sun	Sucrose-6F-phosphate phosphohydrolase family protein;(		cytoplasm-GO:0005737,cy	199.5100897	-0.855128556	0.17436341	-4.904288974	9.38E-07	2.65E-05
PITA_000021151	High quality	AT1G22990	MA_10428986g0010	shade<sun	Heavy metal transport/detoxifi	HEAVY METAL ASSOCIATE	cadmium ion homeostasis	1263.775854	-2.069525929	0.22297464	-9.281440706	1.67E-20	4.30E-18
PITA_000021184	High quality	AT5G42390	MA_298732g0010	shade<sun	Encodes a chloroplast-localizec	STROMAL PROCESSING PE	chloroplast-GO:0009507,c	192.2468159	-1.286037936	0.43984408	-2.923849605	0.003457317	0.034504767
PITA_000021229	Low quality	AT4G26500	MA_181487g0010	shade<sun	Sulfur acceptor that interacts v	CHLOROPLAST SULFUR E (	chloroplast-GO:0009507,c	404.6399273	-1.025911831	0.08776361	-11.68948946	1.44E-31	9.57E-29
PITA_000021253	High quality	AT4G32520	MA_169071g0010	shade<sun	Encodes a serine hydroxymeth	SERINE HYDROXYMETHYL	chloroplast-GO:0009507,c	729.2557685	-1.326441188	0.1579453	-8.398105053	4.54E-17	7.84E-15
PITA_000021255	High quality	AT4G13930	MA_11357g0010	shade<sun	Encodes a serine hydroxymeth	SERINE HYDROXYMETHYL	circadian rhythm-GO:0007	576.2378287	-0.888809904	0.21061977	-4.219973737	2.44E-05	0.00051045
PITA_000021275	High quality	AT1G68650	MA_22128g0010	shade<sun	Member of the UPF0016 famil	PHOTOSYNTHESIS-AFFECT	calcium ion transmembrar	147.1843716	-0.600930511	0.20753513	-2.895560491	0.003784821	0.036988824
PITA_000021328	Low quality	AT5G17540	MA_189139g0010	shade<sun	HXXXD-type acyl-transferase family protein;(source:Arapo		cytoplasm-GO:0005737,tra	200.3879998	-11.27601234	1.31308549	-8.587416785	8.89E-18	1.73E-15
PITA_000021414	Low quality	AT3G22470	MA_10432772g0010	shade<sun	Pentatricopeptide repeat (PPR	PENTATRICOPEPTIDE REPI	chloroplast-GO:0009507	182.9664666	-0.934656926	0.25465481	-3.670289643	0.000242276	0.003781061
PITA_000021420	High quality	AT2G06050	MA_10427053g0010	shade<sun	Encodes a 12-oxophytodienoat	OXOPHYTODIENOATE-REI	12-oxophytodienoate redu	7495.679743	-3.582325185	0.44920235	-7.974858568	1.53E-15	2.07E-13
PITA_000021458	Low quality	AT4G35490	MA_24522g0020	shade<sun	mitochondrial ribosomal prote	MITOCHONDRIAL RIBOSO	large ribosomal subunit-G	203.5263693	-11.29898569	3.90700807	-2.891979101	0.003828234	0.037262858
PITA_000021460	High quality	AT4G30580	MA_141060g0010	shade<sun	Encodes a plastidic lysophosph	(ATS2)	1-acylglycerol-3-phosphate	120.2798217	-0.537255198	0.18159537	-2.958529116	0.00309111	0.031613059
PITA_000021466	High quality	AT4G27410	MA_75192g0010	shade>sun	Encodes a NAC transcription fa	RESPONSIVE TO DESICCAT	DNA binding-GO:0003677,	14.86632324	3.005333573	0.57348901	5.240437976	1.60E-07	5.13E-06
PITA_000021520	High quality	AT5G06700	MA_102045g0010	shade<sun	Encodes a member of the TBL	TRICHOME BIREFRINGENC	cellulose biosynthetic proc	400.7759997	-12.27636939	1.3112187	-9.362564271	7.78E-21	2.09E-18
PITA_000021594	High quality	AT2G27290	MA_121617g0010	shade<sun	FAM210B-like protein, putative	(DUF1279);(source:Arapo	biological_process_unkno	259.4474487	-1.027654157	0.22100917	-4.649825785	3.32E-06	8.46E-05
PITA_000021648	High quality	AT3G09640	MA_177059g0010	shade<sun	Encodes a cytosolic ascorbate p	ASCORBATE PEROXIDASE	cellular response to oxidat	604.5062368	-1.651420172	0.21439103	-7.702841662	1.33E-14	1.55E-12
PITA_000021680	High quality	AT3G12810	MA_424432g0010	shade<sun	Encodes a protein similar to AT	PHOTOPERIOD-INDEPEND	ATPase activity-GO:00168	336.470898	-12.02419582	3.90691191	-3.077672617	0.00208624	0.023219314
PITA_000021695	High quality	AT5G62910	MA_102797g0010	shade<sun	RING/U-box superfamily protein;(source:Araport11)		CCR4-NOT complex-GO:00	508.7959496	-0.872074333	0.20907726	-4.171062469	3.03E-05	0.000615121
PITA_000021709	High quality	AT5G40810	MA_10429544g0010	shade<sun	Cytochrome C1 family;(source:Araport11)		heme binding-GO:002003	105.4291397	-0.454887761	0.15525	-2.930033851	0.003389251	0.033952962
PITA_000021755	High quality	AT5G59610	MA_10428997g0010	shade<sun	Chaperone DnaJ-domain super	DNA J PROTEIN C73 (DJC7	chaperone cofactor-deper	156.7904686	-0.614525355	0.21522535	-2.855264747	0.004300097	0.040754768
PITA_000021812	High quality	AT5G42180	MA_10428759g0010	shade<sun	Peroxidase required for caspar	PEROXIDASE 64 (PER64)	extracellular region-GO:00	165.4497152	-1.519595088	0.52533743	-2.892607679	0.003820582	0.037245874
PITA_000021973	High quality	AT5G28840	MA_141445g0010	shade<sun	Encodes a protein with GDP-D-	GDP-D-MANNOSE 3',5'-EP	cytosol-GO:0005829,GDP-ı	135.5646837	-0.524969974	0.13999194	-3.750001542	0.000176833	0.002868
PITA_000022060	High quality	AT1G26945	MA_148495g0010	shade>sun	Encodes a basic helix-loop-heli	KIDARI (KDR)	nucleus-GO:0005634,prote	8.997840948	1.833326931	0.64278872	2.852145484	0.004342522	0.041048878
PITA_000022063	High quality	AT1G75500	MA_115880g0010	shade>sun	An Arabidopsis thaliana homol	WALLS ARE THIN 1 (WAT1	auxin efflux-GO:0010315,r	28.24285591	0.88970372	0.31204338	2.851218067	0.004355209	0.041091796
PITA_000022122	Low quality	AT1G19440	MA_56709g0010	shade<sun	Encodes KCS4, a member of th	3-KETOACYL-COA SYNTHA	3-oxo-arachidoyl-CoA synt	1982.64826	-1.660006567	0.5099744	-3.255078262	0.001133611	0.0140283
PITA_000022156	Low quality	AT1G69420	MA_10426674g0010	shade<sun	DHHC-type zinc finger family protein;(source:Araport11)		endoplasmic reticulum-GC	191.3471926	-0.54135011	0.15321684	-3.533228497	0.000410517	0.005959175
PITA_000022232	High quality	AT5G07990	MA_109033g0010	shade>sun	Required for flavonoid 3' hydro	TRANSPARENT TESTA 7 (T	extracellular region-GO:00	24.57025676	3.140668372	0.83733618	3.75078548	0.000176281	0.002862729
PITA_000022296	High quality	AT1G03475	MA_73740g0010	shade<sun	Encodes coproporphyrinogen I	LESION INITIATION 2 (LIN2	apoplast-GO:0048046,chlc	373.8469299	-1.05895547	0.09244327	-11.45519316	2.21E-30	1.36E-27
PITA_000022336	High quality	AT5G01920	MA_10428389g0010	shade<sun	Chloroplast thylakoid protein k	STATE TRANSITION 8 (STN	chloroplast-GO:0009507,n	196.3522306	-0.471747105	0.12918674	-3.651668162	0.000260542	0.004041424
PITA_000022432	High quality	AT5G23940	MA_10272508g0010	shade>sun	Encodes PERMEABLE LEAVES3	PERMEABLE LEAVES3 (PEL	cutin biosynthetic process	111.0899577	0.627574852	0.20796747	3.017658724	0.002547356	0.02711875
PITA_000022498	High quality	AT3G14930	MA_10435805g0010	shade<sun	Uroporphyrinogen decarboxyla	(HEME1)	chlorophyll biosynthetic p	1299.062387	-1.990324897	0.15804413	-12.59347578	2.29E-36	2.41E-33
PITA_000022505	High quality	AT1G14190	MA_137064g0010	shade<sun	Glucose-methanol-choline (GMC) oxidoreductase family p		chloroplast-GO:0009507,fl	126.8490814	-1.589560444	0.20879234	-7.613116531	2.68E-14	2.91E-12
PITA_000022525	High quality	AT5G54080	MA_44565g0010	shade>sun	Encodes a homogentisate 1,2-(	HOMOGENTISATE 1,2-DIO	cytoplasm-GO:0005737,cy	34.99385569	0.831375027	0.25881129	3.212282645	0.001316848	0.015913222
PITA_000022529	Low quality	AT5G59900	MA_10430109g0010	shade<sun	Pentatricopeptide repeat (PPR)	superfamily protein;(sourc	biological_process_unkno	146.2411579	-0.828439871	0.23875064	-3.469895859	0.00052066	0.007323508
PITA_000022582	High quality	AT3G62110	MA_88519g0010	shade<sun	Pectin lyase-like superfamily protein;(source:Araport11)		carbohydrate metabolic pı	117.3205358	-0.753026094	0.24692522	-3.049611938	0.002291372	0.025014879
PITA_000022622	High quality	AT3G53900	MA_6556215g0010	shade<sun	Encodes UPP, a plastidial uraci	URACIL PHOSPHORIBOSYL	cellular response to phosp	155.7003484	-0.428454556	0.14623156	-2.929973318	0.003389911	0.033952962
PITA_000022625	High quality	AT3G63410	MA_215523g0010	shade<sun	Encodes a MPBQ/MSBQ methy	ALBINO OR PALE GREEN N	2-methyl-6-geranylgeranyl	356.5907859	-1.532822322	0.177389	-8.64102261	5.57E-18	1.13E-15
PITA_000022645	High quality	AT4G00660	MA_7069g0010	shade<sun	RNAhelicase-like 8;(source:Ara	RNAHELICASE-LIKE 8 (RH8	cytoplasm-GO:0005737,cy	235.4706995	-1.153098441	0.28448749	-4.053248333	5.05E-05	0.000966537
PITA_000022694	High quality	AT2G39650	MA_659011g0010	shade>sun	cruciferin (DUF506);(source:Araport11)		biological_process_unkno	32.85669751	1.544757119	0.29000081	5.326733732	1.00E-07	3.33E-06
PITA_000022709	Low quality	AT2G19730	MA_231973g0010	shade>sun	Ribosomal L28e protein family;(source:Araport11)		adaxial/abaxial pattern sp	36.33562168	1.730306931	0.29930277	5.781125611	7.42E-09	2.97E-07
PITA_000022742	High quality	AT4G24800	MA_10693g0010	shade>sun	MA3 domain-containing protei	EIN2 C-TERMINUS INTERA	cytoplasm-GO:0005737,cy	34.08600038	1.111317266	0.26849594	4.139046754	3.49E-05	0.000693107
PITA_000022751	High quality	AT5G11520	MA_72677g0010	shade<sun	Encodes the chloroplastic isozy	ASPARTATE AMINOTRANS	2-oxoglutarate metabolic p	253.9280134	-1.369586393	0.11672794	-11.73315013	8.62E-32	5.87E-29
PITA_000022755	High quality	AT1G56310	MA_210052g0010	shade<sun	DEDDy-type 3′ -> 5&#8	ATRIMMER2 (ATRM2)	3'-5' exonuclease activity-(	111.8881203	-0.651352035	0.17041985	-3.822043144	0.000132351	0.002225254
PITA_000022758	High quality	AT1G10940	MA_19316g0010	shade>sun	Encodes a plant protein kinase	SNF1-RELATED PROTEIN K	cytoplasm-GO:0005737,cy	32.93103754	0.757146769	0.26781808	2.827093592	0.00469726	0.043681744
PITA_000022826	High quality	AT2G44850	MA_114193g0010	shade<sun	hypothetical protein;(source:Araport11)		biological_process_unkno	136.431951	-0.696237834	0.21618127	-3.220620462	0.001279134	0.015531904
PITA_000022839	High quality	AT3G19553	MA_99177g0010	shade<sun	Encodes POLYAMINE UPTAKE	POLYAMINE UPTAKE TRAI	basic amino acid transmer	129.8308202	-0.632901019	0.21216895	-2.983004851	0.002854335	0.029783335
PITA_000022962	Low quality	AT5G46580	MA_283144g0010	shade<sun	pentatricopeptide (PPR) repea	SUPPRESSOR OF THYLAKC	chloroplast-GO:0009507	184.0982023	-0.844935541	0.13235318	-6.383945856	1.73E-10	9.12E-09
PITA_000022967	High quality	AT1G23820	MA_17812g0010	shade<sun	Spermidine synthase.	SPERMIDINE SYNTHASE 1	chloroplast-GO:0009507,c	155.9171735	-0.773475914	0.15487973	-4.994042136	5.91E-07	1.73E-05
PITA_000022991	Low quality	AT3G52430	MA_10428089g0010	shade<sun	Encodes a lipase-like gene that	PHYTOALEXIN DEFICIENT	aerenchyma formation-GC	272.0038353	-2.609400082	0.68210929	-3.825486804	0.000130514	0.002201713
PITA_000023013	Low quality	AT2G43950	MA_104060g0010	shade<sun	Constitutes a peptide sensitive	CHLOROPLAST OUTER EN\	cation transport-GO:00068	157.9960172	-0.759619936	0.14791875	-5.135386514	2.82E-07	8.64E-06
PITA_000023021	High quality	AT3G02690	MA_8499291g0010	shade<sun	Nucleotide/sugar transporter family protein		chloroplast-GO:0009507,ir	433.0184482	-1.170758773	0.26177665	-4.472357475	7.74E-06	0.000183027
PITA_000023126	High quality	AT1G27510	MA_10431343g0010	shade<sun	Encodes one of the two plastid	EXECUTER 2 (EX2)	chloroplast-GO:0009507,r	127.7345477	-0.676905155	0.16903079	-4.004626252	6.21E-05	0.00114682
PITA_000023131	Low quality	AT2G31840	MA_875929g0010	shade<sun	Thioredoxin superfamily prote	MESOPHYLL-CELL RNAI LIE	chloroplast-GO:0009507,c	310.0230394	-0.975556658	0.15620728	-6.245270091	4.23E-10	2.08E-08
PITA_000023226	High quality	AT1G12520	MA_10437032g0010	shade<sun	Copper-zinc superoxide dismut	COPPER CHAPERONE FOR	cellular copper ion homeo	100.4192822	-0.776348666	0.20103957	-3.861670963	0.000112614	0.001936012
PITA_000023362	Low quality	AT4G37850	MA_14104g0010	shade>sun	basic helix-loop-helix (bHLH) DNA-binding superfamily pro		DNA-binding transcription	293.0135266	11.49086849	3.90689269	2.941178428	0.003269661	0.032971155
PITA_000023415	High quality	AT3G02450	MA_10436247g0010	shade<sun	Proteolytically inactive membe	FTSH (FILAMENTATION-TE	ATP-dependent peptidase	190.7966231	-1.012086265	0.13654844	-7.411920876	1.24E-13	1.22E-11
PITA_000023465	High quality	AT1G08465	MA_112273g0010	shade>sun	Member of the YABBY family c	YABBY2 (YAB2)	abaxial cell fate specificati	63.00357664	0.828708105	0.24053445	3.445278241	0.000570472	0.007900769
PITA_000023516	High quality	AT2G30390	MA_10436993g0010	shade<sun	Encodes one of two ferrochela	FERROCHELATASE 2 (FC2)	chloroplast-GO:0009507,fe	219.6327824	-2.915328858	0.8397949	-3.471477212	0.000517603	0.00728864
PITA_000023546	Low quality	AT4G26090	MA_10265639g0010	shade>sun	Encodes a plasma membrane p	RESISTANT TO P. SYRINGA	ADP binding-GO:0043531,	25.31413105	7.956810503	1.54487021	5.150471847	2.60E-07	8.02E-06

PITA_000023600	High quality	AT2G02040	MA_10428144g0010	shade<sun	Encodes a di- and tri-peptide t	NRT1/ PTR FAMILY 8.3 (NF	dipeptide transport-GO:00	166.6559179	-1.224347179	0.29086474	-4.20933515	2.56E-05	0.000530331
PITA_000023661	Low quality	AT1G62670	MA_132853g0030	shade<sun	Encodes a pentatricopeptide r	RNA PROCESSING FACTOR	mitochondrion-GO:00057:	407.6875484	-2.197062748	0.51224529	-4.289083384	1.79E-05	0.000385743
PITA_000023764	High quality	AT2G35770	MA_8233995g0010	shade<sun	serine carboxypeptidase-like 2:	SERINE CARBOXYPEPTIDA	extracellular region-GO:00	1729.550698	-1.115606295	0.26594582	-4.194863027	2.73E-05	0.000562121
PITA_000024011	Low quality	AT2G26250	MA_309409g0010	shade>sun	epidermis-specific, encodes KC	3-KETOACYL-COA SYNTHA	3-oxo-arachidoyl-CoA synt	49.05146367	1.241877261	0.2374714	5.229586706	1.70E-07	5.40E-06
PITA_000024052	Low quality	AT3G54000	MA_213656g0010	shade>sun	TIP41-like protein;(source:Araport11)		biological_process_unkno	29.72416361	1.319187599	0.32825652	4.0187705	5.85E-05	0.001089687
PITA_000024055	High quality	AT2G23350	MA_36585g0020	shade<sun	polyadenylate-binding protein,	POLY(A) BINDING PROTEII	cytosol-GO:0005829,mRN/	111.7951301	-0.574150077	0.16433287	-3.493823634	0.000476155	0.006757817
PITA_000024057	High quality	AT1G08460	MA_121026g0010	shade<sun	histone deacetylase 8;(source:/	HISTONE DEACETYLASE 8	histone deacetylase activit	153.9029543	-0.59362392	0.14472675	-4.101687532	4.10E-05	0.000800614
PITA_000024144	High quality	AT5G54570	MA_484764g0010	shade<sun	beta glucosidase 41;(source:Ar	BETA GLUCOSIDASE 41 (B	beta-glucosidase activity-G	406.9421235	-1.255067656	0.44229062	-2.837653815	0.004544644	0.042466073
PITA_000024145	High quality	AT4G21770	MA_117552g0010	shade<sun	Pseudouridine synthase family protein;(source:Araport11		chloroplast-GO:0009507,e	250.7172376	-0.917573285	0.2122161	-4.32376841	1.53E-05	0.000335508
PITA_000024159	High quality	AT5G56090	MA_9897801g0010	shade<sun	Encodes a homolog of COX15.	CYTOCHROME C OXIDASE	heme A biosynthetic proce	216.7686152	-1.195092766	0.22713018	-5.261708467	1.43E-07	4.61E-06
PITA_000024242	Low quality	AT2G32980	MA_10427389g0010	shade<sun	HAUS augmin-like complex sul	AUGMIN SUBUNIT 2 (AUG	cytokinesis by cell plate fo	597.5960626	-2.35956438	0.51020881	-4.624703337	3.75E-06	9.46E-05
PITA_000024245	High quality	AT4G17560	MA_947876g0010	shade<sun	Ribosomal protein L19 family protein;(source:Araport11)		chloroplast-GO:0009507,c	551.9475012	-1.259162728	0.19344915	-6.509011369	7.56E-11	4.28E-09
PITA_000024269	High quality	AT3G13120	MA_10432148g0010	shade<sun	Ribosomal protein S10p/S20e f	PLASTID RIBOSOMAL PRO	chloroplast-GO:0009507,c	583.6958601	-0.914397752	0.20168457	-4.533801337	5.79E-06	0.00014063
PITA_000024316	High quality	AT1G73880	MA_57964g0010	shade<sun	UDP-glucosyl transferase 89B1	UDP-GLUCOSYL TRANSFEF	daphnetin 3-O-glucosyltra	197.5754798	-0.938480582	0.19130544	-4.90566593	9.31E-07	2.64E-05
PITA_000024471	High quality	AT5G42480	MA_50216g0010	shade<sun	Shows homology to the cyanol	ACCUMULATION AND REF	chloroplast-GO:0009507,c	160.5991489	-0.93970598	0.15290412	-6.145720478	7.96E-10	3.72E-08
PITA_000024618	High quality	AT1G14350	MA_10431933g0010	shade>sun	Encodes a putative MYB transc	FOUR LIPS (FLP)	auxin polar transport-GO:(	48.8754955	1.08146453	0.27671786	3.908184801	9.30E-05	0.001638898
PITA_000024693	High quality	AT4G36810	MA_1011718g0010	shade>sun	Encodes a protein with geranyl	GERANYLGERANYL PYROP	chloroplast-GO:0009507,c	203.9130795	10.96787582	3.90695098	2.807272443	0.004996297	0.045720827
PITA_000024716	High quality	AT5G24760	MA_409626g0010	shade>sun	GroES-like zinc-binding dehydrogenase family protein;(sou		alcohol dehydrogenase ac	68.27401995	1.116519506	0.24909005	4.482393107	7.38E-06	0.000175614
PITA_000024795	Low quality	AT1G18900	MA_10428189g0010	shade<sun	Pentatricopeptide repeat (PPR) superfamily protein;(sour		mitochondrion-GO:00057:	188.3499285	-0.819880335	0.14138726	-5.798827461	6.68E-09	2.70E-07
PITA_000024824	Low quality	AT2G34640	MA_8815984g0010	shade<sun	Present in transcriptionally act	PLASTID TRANSCRIPTION/	chloroplast-GO:0009507,c	241.2573992	-1.245862432	0.19956229	-6.242975367	4.29E-10	2.10E-08
PITA_000024853	Low quality	AT1G80710	MA_42386g0010	shade>sun	Encodes a WD-40 repeat famil	DROUGHT SENSITIVE 1 (D	cellular response to DNA c	43.04519116	1.289452779	0.40762007	3.163369213	0.001559544	0.018353571
PITA_000024916	Low quality	AT4G39230	MA_10427035g0010	shade<sun	encodes a protein whose sequ	PHENYLCOUMARAN BENZ	cytoplasm-GO:0005737,lig	833.8550088	-1.482051186	0.20714151	-7.154776321	8.38E-13	7.19E-11
PITA_000025023	High quality	AT1G32360	MA_324587g0010	shade>sun	Zinc finger (CCCH-type) family protein;(source:Araport11)		cytoplasm-GO:0005737,DN	57.16433169	1.341914782	0.28428865	4.720254528	2.36E-06	6.19E-05
PITA_000025035	High quality	AT5G13520	MA_42276g0020	shade<sun	peptidase M1 family protein;(source:Araport11)		chloroplast-GO:0009507,c	135.1479365	-0.753209372	0.1663788	-4.527075354	5.98E-06	0.000144609
PITA_000025185	High quality	AT1G47260	MA_10435782g0010	shade<sun	Encodes mitochondrial gamma	GAMMA CARBONIC ANHY	anther dehiscence-GO:00(	109.5068514	-0.5730213	0.16651822	-3.441192889	0.000579156	0.00799907
PITA_000025316	High quality	AT4G00730	MA_93471g0010	shade>sun	Encodes a homeodomain prot	ANTHOCYANINLESS 2 (AN	anthocyanin accumulation	35.38261859	1.471535538	0.28897341	5.092287059	3.54E-07	1.07E-05
PITA_000025330	Low quality	AT5G45040	MA_122378g0010	shade<sun	Encodes a Class I cytochrome c	CYTOCHROME C6A (CYTC6	chloroplast-GO:0009507,e	153.4041591	-0.783382284	0.19776672	-3.961143191	7.46E-05	0.001347568
PITA_000025436	High quality	AT2G23620	MA_26076g0010	shade>sun	Encodes a protein shown to ha	METHYL ESTERASE 1 (MES	hydrolase activity, acting c	33.94290737	1.563561051	0.47272811	3.307527108	0.000941236	0.011994933
PITA_000025448	High quality	AT5G23400	MA_100087g0010	shade<sun	Leucine-rich repeat (LRR) family protein;(source:Araport1:		cell wall-GO:0005618,extr	52.20483456	-1.445312432	0.39348055	-3.673148303	0.00023958	0.003745949
PITA_000025543	High quality	AT4G27600	MA_159115g0010	shade<sun	Encodes a phosphofructokinas	GENES NECESSARY FOR TI	chloroplast-GO:0009507,p	3389.722949	-1.953132334	0.44147408	-4.424115546	9.68E-06	0.000223093
PITA_000025610	High quality	AT2G22070	MA_3449g0010	shade<sun	pentatricopeptide (PPR) repeat-containing protein;(source		intracellular membrane-bo	281.4630361	-1.849522446	0.20835905	-8.876612184	6.89E-19	1.52E-16
PITA_000025634	High quality	AT1G74970	MA_18349g0020	shade<sun	ribosomal protein S9, nuclear e	RIBOSOMAL PROTEIN S9 (	chloroplast-GO:0009507,c	418.8162289	-1.092263126	0.2720029	-4.015630452	5.93E-05	0.001103484
PITA_000025647	High quality	AT3G19940	MA_457005g0010	shade>sun	Encodes a hexose-H(+) sympor	SUGAR TRANSPORT PROT	carbohydrate transmembr	20.59935561	0.989355922	0.35349242	2.798803749	0.00512923	0.04648192
PITA_000025670	High quality	AT3G48730	MA_10085364g0010	shade<sun	glutamate-1-semialdehyde 2,1-	GLUTAMATE-1-SEMIALDEI	chlorophyll biosynthetic p	413.3588412	-0.916376971	0.14994887	-6.111263033	9.88E-10	4.57E-08
PITA_000025840	Low quality	AT3G06840	MA_19015g0020	shade>sun	hypothetical protein;(source:Araport11)		biological_process_unkno	25.16841237	1.396565933	0.34815701	4.011310632	6.04E-05	0.001122218
PITA_000025902	Low quality	AT1G16520	MA_186538g0010	shade<sun	NAI1 interacting protein, invol	NAI2-INTERACTING PROTE	integral component of me	261.7463584	-0.733569322	0.13891687	-5.280635114	1.29E-07	4.21E-06
PITA_000025912	Low quality	AT1G16880	MA_853979g0010	shade<sun	Encodes a ACT domain-contair	ACT DOMAIN REPEATS 11	chloroplast-GO:0009507,c	185.6856729	-0.88388219	0.29600788	-2.986008976	0.002826443	0.029541195
PITA_000026020	High quality	AT5G15780	MA_73048g0010	shade<sun	Pollen Ole e 1 allergen and extensin family protein;(sourc		biological_process_unkno	231.2440002	-0.442700479	0.14306209	-3.094463879	0.001971689	0.022218701
PITA_000026026	High quality	AT4G30620	MA_165748g0020	shade<sun	Homolog of STIC2, recent dupl	STIC2 LIKE (STCL)	biological_process_unkno	167.1895568	-0.669680541	0.16986853	-3.942346228	8.07E-05	0.001445285
PITA_000026050	High quality	AT1G49160	MA_10299278g0010	shade>sun	Encodes a member of the WNI	(WNK7)	ATP binding-GO:0005524,(	54.11079618	1.942157905	0.65759951	2.953405356	0.00314289	0.031986956
PITA_000026058	Low quality	AT4G33590	MA_10433724g0010	shade<sun	transmembrane protein;(source:Araport11)		extracellular region-GO:00	528.7383279	-0.959879951	0.24118775	-3.979803929	6.90E-05	0.001258667
PITA_000026064	High quality	AT1G74690	MA_1548g0010	shade<sun	Encodes a microtubule-associa	IQ-DOMAIN 31 (IQD31)	biological_process_unkno	160.5647647	-0.405800892	0.12828687	-3.163230047	0.00156029	0.018353782
PITA_000026084	Low quality	AT5G08410	MA_925722g0010	shade<sun	ferredoxin/thioredoxin reducta	FERREDOXIN/THIOREDOX	chloroplast-GO:0009507,p	246.9947298	-0.58088217	0.19408518	-2.992923938	0.002763186	0.029012303
PITA_000026146	Low quality	AT3G15520	MA_10432513g0010	shade<sun	Cyclophilin-like peptidyl-prolyl	cis-trans isomerase family		chloroplast-GO:0009507,c	897.0832976	-1.492907788	0.08674788	-17.20973252	2.24E-66	1.13E-62
PITA_000026249	Low quality	AT3G60520	MA_322880g0010	shade>sun	zinc ion-binding protein;(source:Araport11)		cellular response to hypox	219.6229902	11.07504069	1.30417611	8.491982473	2.03E-17	3.77E-15
PITA_000026312	High quality	AT4G21990	MA_16080g0010	shade<sun	Encodes a protein disulfide iso	APS REDUCTASE 3 (APR3)	adenylyl-sulfate reductase	682.157698	-2.039984065	0.25062724	-8.139514408	3.97E-16	6.18E-14
PITA_000026443	High quality	AT1G72710	MA_6447g0010	shade>sun	Encodes a member of the case	CASEIN KINASE 1-LIKE PRC	cytoplasm-GO:0005737,en	39.86994439	0.701515068	0.24098756	2.911001186	0.003602727	0.035506355
PITA_000026472	High quality	AT4G27670	MA_8682g0010	shade>sun	Encodes Hsp21, a chloroplast l	HEAT SHOCK PROTEIN 21	chaperone complex-GO:0:	171.4001935	10.71751462	1.62858369	6.580880504	4.68E-11	2.77E-09
PITA_000026507	High quality	AT5G34940	MA_77698g0010	shade>sun	The protein is predicted (WoL	GLUCURONIDASE 3 (GUS3	beta-glucuronidase activity	49.39470219	1.560924978	0.34319115	4.548266985	5.41E-06	0.000132312
PITA_000026545	High quality	AT4G29100	MA_114573g0010	shade>sun	basic helix-loop-helix (bHLH) D	(BHLH68)	DNA-binding transcription	45.49806572	0.656593331	0.22817781	2.877551257	0.004007748	0.038593128
PITA_000026574	High quality	AT1G44575	MA_575444g0010	shade<sun	Encoding PSII-S (CP22), a ubiqu	NONPHOTOCHEMICAL QU	chlorophyll binding-GO:00	1623.402613	-2.074535293	0.46789079	-4.433802383	9.26E-06	0.000214615
PITA_000026589	High quality	AT1G72320	MA_10434208g0010	shade>sun	Encodes a member of the Arab	PUMILIO 23 (PUM23)	90S preribosome-GO:003C	19.04480768	7.550113981	1.30525085	5.784416073	7.28E-09	2.92E-07
PITA_000026646	High quality	AT5G23535	MA_10134g0010	shade<sun	KOW domain-containing protein;(source:Araport11)		mitochondrion-GO:00057:	607.2482953	-0.762532974	0.15330572	-4.973937012	6.56E-07	1.90E-05
PITA_000026647	Low quality	AT4G18390	MA_4774g0010	shade<sun	TEOSINTE BRANCHED 1, cycloi	TEOSINTE BRANCHED 1, C	cell differentiation-GO:003	112.8599618	-0.536260113	0.19047367	-2.815402793	0.004871613	0.044955026
PITA_000026658	High quality	AT3G57670	MA_879g0010	shade>sun	Encodes a a C2H2/C2HC zinc fi	NO TRANSMITTING TRACT	anatomical structure deve	77.86517192	0.949157289	0.21822403	4.349462704	1.36E-05	0.000302178
PITA_000026702	High quality	AT5G19420	MA_10435870g0010	shade>sun	Regulator of chromosome condensation (RCC1) family wit		chloroplast-GO:0009507,c	50.25011022	0.750366476	0.25507278	2.941773976	0.00326338	0.032971155
PITA_000026722	High quality	AT1G02000	MA_52380g0010	shade<sun	UDP-D-glucuronate 4-epimera:	UDP-D-GLUCURONATE 4-E	UDP-glucuronate 4-epime	190.5425493	-0.711110472	0.20595874	-3.4526841	0.000555039	0.007725206
PITA_000026739	High quality	AT1G55140	MA_10430520g0010	shade<sun	Encodes one of two chloroplas	(RNC3)	chloroplast-GO:0009507,m	128.2067906	-0.472892897	0.1537685	-3.075356201	0.002102513	0.023338633
PITA_000026798	High quality	AT2G46950	MA_102685g0020	shade<sun	cytochrome P450, family 709, s	CYTOCHROME P450, FAM	chloroplast-GO:0009507,c	173.8503709	-0.987240665	0.30231239	-3.265630881	0.001092205	0.013589253
PITA_000026799	High quality	AT5G14100	MA_890463g0010	shade<sun	Member of NAP subfamily. Pu	ATP-BINDING CASSETTE I1	ATP binding-GO:0005524,/	118.5533863	-0.486340558	0.16698712	-2.912443501	0.003586131	0.035384278
PITA_000027023	High quality	AT3G44200	MA_1191g0010	shade>sun	Encodes AtNek5, a member of	NIMA (NEVER IN MITOSIS,	cortical microtubule-GO:0	70.52927248	0.709681425	0.22804715	3.111994318	0.001858281	0.021110739
PITA_000027039	High quality	AT1G67100	MA_70989g0010	shade>sun	LOB domain-containing protei	LOB DOMAIN-CONTAININ	regulation of gene express	50.84669598	1.815070237	0.56304242	3.223682914	0.001265534	0.01538158
PITA_000027095	Low quality	AT1G56180	MA_9271584g0010	shade<sun	VIR3 encodes a putative chlorc	VIRESCENT3-1 (VIR3)	ATP-dependent peptidase	240.0838207	-1.110570017	0.19267686	-5.763899419	8.22E-09	3.27E-07
PITA_000027260	High quality	AT5G66390	MA_109058g0010	shade>sun	Encodes a peroxidase that is in	PEROXIDASE 72 (PRX72)	extracellular region-GO:00	33.57782553	4.331965802	1.2541019	3.454237494	0.000551851	0.00768933
PITA_000027348	High quality	AT4G39970	MA_137202g0010	shade<sun	Haloacid dehalogenase-like hydrolase (HAD) superfamily		chloroplast-GO:0009507,c	999.3041206	-1.169654878	0.17301895	-6.760270526	1.38E-11	9.17E-10
PITA_000027358	High quality	AT3G27925	MA_10431940g0010	shade<sun	Encodes a DegP protease; nucl	DEGRADATION OF PERIPL	chloroplast-GO:0009507,c	505.7355309	-1.087887072	0.25479858	-4.269596271	1.96E-05	0.000417478
PITA_000027373	High quality	AT3G59890	MA_122229g0010	shade<sun	Dihydrodipicolinate reductase, bacterial/plant;(source:Ar		4-hydroxy-tetrahydrodipic	147.5650179	-0.540720218	0.17808063	-3.036378538	0.002394386	0.025839283
PITA_000027404	Low quality	AT3G22530	MA_102330g0020	shade>sun	heat shock protein;(source:Araport11)		biological_process_unkno	32.93407425	0.990608831	0.28986905	3.417435708	0.00063214	0.008599016
PITA_000027481	Low quality	AT1G72480	MA_39312g0010	shade>sun	Lung seven transmembrane receptor family protein;(sour		Golgi apparatus-GO:00057	98.4386655	0.587560826	0.16203452	3.626146084	0.000287683	0.004402522
PITA_000027558	High quality	AT2G33580	MA_892990g0010	shade<sun	Encodes a putative LysM-conta	LYSM-CONTAINING RECEP	cellular response to chitin-	904.0029273	-2.036296646	0.16499015	-12.34192823	5.39E-35	4.68E-32
PITA_000027571	Low quality	AT3G56580	MA_76949g0010	shade<sun	Encodes a functional E3 ubiqui	RING ZINC FINGER 1 (RZF1	negative regulation of pro	474.4754957	-0.979014798	0.19820779	-4.939335619	7.84E-07	2.24E-05
PITA_000027635	Low quality	AT2G14680	MA_9482453g0010	shade<sun	myosin heavy chain-like protei	MATERNAL EFFECT EMBR'	cytoplasm-GO:0005737,en	88.65508679	-0.48611838	0.16991992	-2.860867543	0.004224835	0.040217156
PITA_000027651	High quality	AT5G53490	MA_10429821g0010	shade<sun	thylakoid lumenal 17.4 kDa prc	THYLAKOID LUMENAL 17.4	biological_process_unkno	175.8802009	-0.602881731	0.15131655	-3.984241896	6.77E-05	0.001239862
PITA_000027763	High quality	AT1G78570	MA_10431083g0010	shade<sun	Encodes a UDP-L-Rhamnose sy	RHAMNOSE BIOSYNTHESI	auxin efflux-GO:0010315,c	125.8074571	-0.486329544	0.1466522	-3.316210342	0.000912471	0.011699301
PITA_000027835	Low quality	AT1G80380	MA_9944g0020	shade<sun	encodes a glycerate kinase whi	GLYCERATE KINASE (GLYK)	ATP binding-GO:0005524,(	244.955056	-0.964843384	0.10862141	-8.882626162	6.53E-19	1.46E-16
PITA_000027842	High quality	AT4G22010	MA_10236360g0010	shade<sun	SKU5 similar 4;(source:Araport	SKU5 SIMILAR 4 (sks4)	copper ion binding-GO:00	377.8534077	-0.883942542	0.31483967	-2.807595833	0.004991283	0.045720827
PITA_000027851	High quality	AT1G14790	MA_10436625g0010	shade>sun	Encodes RNA-dependent RNA	RNA-DEPENDENT RNA PO	nuclear RNA-directed RNA	43.30716232	1.15957096	0.30102847	3.852030821	0.000117142	0.002002934
PITA_000027873	Low quality	AT4G35220	MA_10064667g0010	shade<sun	Cyclase family protein;(source:	CYCLASE2 (CYCLASE2)	arylformamidase activity-G	105.85943	-0.732485842	0.25085739	-2.919929249	0.003501109	0.034766771
PITA_000027912	High quality	AT3G12120	MA_203704g0010	shade<sun	Major enzyme responsible for	FATTY ACID DESATURASE	1-16:0-2-18:1-phosphatidy	270.5356173	-1.253233642	0.32388917	-3.869328674	0.000109135	0.001882624
PITA_000027950	High quality	AT5G11880	MA_10426527g0020	shade<sun	Meso-diaminopimelate decarb	MESO-DIAMINOPIMELATE	chloroplast-GO:0009507,c	292.0784286	-0.769831247	0.18389417	-4.186273207	2.84E-05	0.000581986
PITA_000027981	High quality	AT3G26380	MA_10434513g0010	shade<sun	APSE is a member of the Glyco	ARAPASE (APSE)	arabinogalactan protein m	2957.246067	-1.855973785	0.08995976	-20.63115458	1.44E-94	1.21E-90
PITA_000028024	High quality	AT1G10950	MA_10431134g0010	shade<sun	Encodes an Arabidopsis Transr	TRANSMEMBRANE NINE 1	cis-Golgi network-GO:000!	114.107412	-0.6739467	0.22602669	-2.981712931	0.002866406	0.02985988
PITA_000028058	High quality	AT1G20230	MA_237076g0010	shade<sun	Pentatricopeptide repeat (PPR) superfamily protein;(sour		mitochondrion-GO:00057:	177.7091446	-1.130511252	0.32630701	-3.464563229	0.000531093	0.007445343
PITA_000028119	High quality	AT3G01930	MA_40195g0010	shade>sun	Major facilitator superfamily protein;(source:Araport11)		membrane-GO:0016020,m	16.14940668	1.948129701	0.51422935	3.788445213	0.000151593	0.002513594
PITA_000028190	High quality	AT5G05590	MA_10433593g0010	shade<sun	Encodes phosphoribosylanthra	PHOSPHORIBOSYLANTHR/	chloroplast-GO:0009507,p	187.3508326	-0.835916413	0.20379469	-4.101757587	4.10E-05	0.000800614
PITA_000028233	High quality	AT4G12910	MA_8787g0010	shade<sun	serine carboxypeptidase-like 2(	SERINE CARBOXYPEPTIDA	extracellular region-GO:00	2832.257696	-2.432515654	0.41019897	-5.930087191	3.03E-09	1.30E-07
PITA_000028289	Low quality	AT3G15630	MA_69177g0010	shade>sun	plant/protein;(source:Araport11)		biological_process_unkno	302.4325457	11.53650736	3.90688853	2.952863196	0.003148415	0.032017345
PITA_000028349	High quality	AT5G50400	MA_133060g0010	shade<sun	purple acid phosphatase 27;(sc	PURPLE ACID PHOSPHATA	acid phosphatase activity-(	254.6246778	-11.62212805	3.90695923	-2.974724681	0.002932516	0.030397886
PITA_000028402	Low quality	AT4G34760	MA_10901g0010	shade>sun	SAUR-like auxin-responsive prc	SMALL AUXIN UPREGULAT	mitochondrion-GO:00057:	7.582986284	6.223509025	1.35757633	4.584279273	4.56E-06	0.000113529
PITA_000028414	High quality	AT1G29880	MA_10433789g0010	shade<sun	glycyl-tRNA synthetase / glycine-tRNA ligase;(source:Arap		chloroplast-GO:0009507,c	126.6976243	-0.623797637	0.14927299	-4.178904946	2.93E-05	0.000597681
PITA_000028430	High quality	AT2G47450	MA_845170g0010	shade<sun	A component of the chloroplas	CHAOS (CAO)	chloroplast-GO:0009507,c	272.4006723	-0.627122412	0.09784948	-6.409052363	1.46E-10	7.81E-09
PITA_000028434	High quality	AT2G42990	MA_58098g0010	shade<sun	GDSL-motif esterase/acyltransferase/lipase. Enzyme grou		hydrolase activity, acting c	1630.906822	-1.23408757	0.41351404	-2.98439098	0.002841434	0.029661
PITA_000028445	Low quality	AT5G57625	MA_907632g0010	shade<sun	CAP (Cysteine-rich secretory pr	(ATCAPE5)	biological_process_unkno	231.9586176	-1.270980794	0.30743609	-4.134130098	3.56E-05	0.000705886
PITA_000028738	Low quality	AT5G44210	MA_10426586g0010	shade>sun	encodes a member of the ERF	ERF DOMAIN PROTEIN 9 (	DNA binding-GO:0003677,	41.13732689	0.723954371	0.23881222	3.03147955	0.002433584	0.026180727
PITA_000028919	Low quality	AT2G32480	MA_117901g0010	shade<sun	Metalloprotease essential for p	ARABIDOPSIS SERIN PROT	chloroplast-GO:0009507,c	166.3335745	-1.094700518	0.14231797	-7.691934735	1.45E-14	1.66E-12
PITA_000029019	High quality	AT3G10840	MA_174635g0010	shade<sun	alpha/beta-Hydrolases superfamily protein;(source:Arapo		chloroplast envelope-GO:(	117.1935899	-0.753867324	0.21470292	-3.511211258	0.00044607	0.006399249
PITA_000029100	Low quality	AT1G48590	MA_140058g0010	shade>sun	Calcium-dependent lipid-bindi	C2-DOMAIN ABA-RELATED	chloroplast-GO:0009507,G	58.19221695	1.772734681	0.47909951	3.700138803	0.000215482	0.003408027
PITA_000029184	High quality	AT3G17430	MA_42261g0010	shade<sun	Nucleotide/sugar transporter family protein The mRNA is		antiporter activity-GO:001	129.1322938	-0.562191027	0.15104054	-3.722120071	0.000197557	0.003159409
PITA_000029207	High quality	AT3G30390	MA_8172061g0010	shade>sun	Encodes a putative amino acid transporter.		amino acid transmembran	71.40173124	1.078683489	0.38739153	2.784478738	0.005361384	0.047999324
PITA_000029236	High quality	AT3G21420	MA_10426390g0020	shade<sun	LATERAL BRANCHING OXIDORI	LATERAL BRANCHING OXII	cytoplasm-GO:0005737,di	600.3756323	-2.578820415	0.80864195	-3.189075725	0.001427285	0.017035553
PITA_000029245	High quality	AT5G38530	MA_10433022g0010	shade<sun	TSBtype2 encodes a type 2 tryp	TRYPTOPHAN SYNTHASE B	chloroplast-GO:0009507,c	117.760864	-0.603847214	0.18013994	-3.352100741	0.000802008	0.010485562
PITA_000029269	Low quality	AT5G64320	MA_105495g0010	shade<sun	MTL1 is a mitochondria localiz	MITOCHONDRIAL TRANSL	mitochondrial translation-	899.7071718	-1.527123046	0.25122192	-6.078781004	1.21E-09	5.56E-08
PITA_000029278	Low quality	AT4G23440	MA_115260g0010	shade>sun	Disease resistance protein (TIR-NBS class);(source:Araport		nucleus-GO:0005634,plasr	366.4213812	11.81340243	3.90686596	3.023754219	0.002496592	0.026747682
PITA_000029315	High quality	AT2G41640	MA_119799g0010	shade<sun	Glycosyltransferase family 61 protein;(source:Araport11)		extracellular region-GO:00	118.6233622	-0.440790689	0.14502882	-3.039331758	0.002371036	0.025653164
PITA_000029507	High quality	AT5G57540	MA_8261658g0010	shade>sun	Encodes a xyloglucan endotrar	XYLOGLUCAN ENDOTRAN!	cell wall biogenesis-GO:00	108.4426182	1.559041678	0.3872094	4.026352818	5.66E-05	0.001059851
PITA_000029617	High quality	AT2G31750	MA_10428919g0010	shade<sun	Encodes an auxin glycosyltrans	UDP-GLUCOSYL TRANSFEF	auxin metabolic process-G	456.7243603	-2.397645547	0.75756909	-3.164919956	0.001551256	0.018273088
PITA_000029621	High quality	AT1G76460	MA_18853g0020	shade>sun	RNA-binding (RRM/RBD/RNP motifs) family protein;(sourc		biological_process_unkno	48.1668264	0.635178591	0.2256826	2.814477422	0.004885661	0.045051689
PITA_000029627	High quality	AT5G61400	MA_69436g0010	shade<sun	Pentatricopeptide repeat (PPR) superfamily protein;(sour		biological_process_unkno	146.965037	-0.892116887	0.19040551	-4.685352184	2.79E-06	7.21E-05
PITA_000029644	High quality	AT5G51600	MA_10426277g0020	shade<sun	Mutant has defective roots. Es	PLEIADE (PLE)	cortical microtubule-GO:0	173.9222439	-0.559974614	0.1907777	-2.935220537	0.003333107	0.033506586
PITA_000029771	High quality	AT5G08370	MA_92663g0010	shade<sun	Member of Glycoside Hydrolas	ALPHA-GALACTOSIDASE 2	alpha-galactosidase activit	144.7111168	-0.715934712	0.18962315	-3.77556604	0.000159645	0.00263325
PITA_000029837	High quality	AT4G36850	MA_87001g0010	shade>sun	PQ-loop repeat family protein / transmembrane family pr		biological_process_unkno	38.66104402	1.383905673	0.36283457	3.814150584	0.000136652	0.002288424
PITA_000029856	High quality	AT5G05600	MA_10435395g0010	shade<sun	Encodes a protein with similari	JASMONIC ACID OXIDASE	cellular response to toxic s	74.61619101	-0.679989562	0.23800801	-2.857002791	0.004276621	0.040559307
PITA_000030002	High quality	AT5G66870	MA_290245g0010	shade>sun	Encodes LOB domain protein v	ASYMMETRIC LEAVES 2-LI	leaf morphogenesis-GO:00	22.2474266	1.318351607	0.3546931	3.716879739	0.000201698	0.003215445
PITA_000030110	High quality	AT3G24140	MA_57244g0010	shade>sun	Encodes a basic helix-loop-heli	FAMA (FMA)	DNA-binding transcription	63.37158872	0.593551781	0.20741775	2.861624802	0.004214755	0.040157208
PITA_000030345	High quality	AT5G53390	MA_10432972g0020	shade>sun	Encodes a member of the bifu	FOLDED PETAL 1 (FOP1)	diacylglycerol O-acyltransf	53.00223377	3.081094763	1.05113673	2.931202651	0.003376524	0.033851501
PITA_000030346	High quality	AT5G53390	MA_297665g0010	shade<sun	Encodes a member of the bifu	FOLDED PETAL 1 (FOP1)	diacylglycerol O-acyltransf	201.4730154	-1.573963799	0.51016038	-3.085233337	0.002033925	0.022777794
PITA_000030347	Low quality	AT1G66160	MA_17715g0010	shade>sun	CYS, MET, PRO, and GLY protei	CYS, MET, PRO, AND GLY F	protein binding-GO:00055	68.00704229	9.383260499	1.47282517	6.370926242	1.88E-10	9.87E-09
PITA_000030441	Low quality	AT4G32300	MA_33934g0020	shade>sun	S-domain-2 5;(source:Araport1	S-DOMAIN-2 5 (SD2-5)	calmodulin binding-GO:00	28.65455348	8.138814769	1.29065491	6.305957317	2.86E-10	1.46E-08
PITA_000030442	High quality	AT5G52440	MA_919815g0010	shade<sun	HCF106; nuclear gene for chlor	HIGH CHLOROPHYLL FLUO	chloroplast-GO:0009507,c	233.2844237	-0.834459946	0.1234744	-6.758161548	1.40E-11	9.28E-10
PITA_000030464	High quality	AT1G09230	MA_181629g0010	shade<sun	Encodes a U12-type spliceoson	U11/U12-65K (U11/U12-6!	inflorescence developmen	162.1222967	-0.994506781	0.21760575	-4.570222992	4.87E-06	0.000120938
PITA_000030495	Low quality	AT2G47840	MA_24093g0010	shade<sun	Encodes a component of the T	TRANSLOCON AT THE INN	chloroplast-GO:0009507,c	214.2764294	-0.490859377	0.16528588	-2.969759894	0.002980326	0.030693734
PITA_000030522	High quality	AT4G34730	MA_7478542g0010	shade<sun	ribosome-binding factor A fam	RBFA DOMAIN-CONTAINII	chloroplast-GO:0009507,ri	435.9148208	-1.178997461	0.20356531	-5.79174042	6.97E-09	2.81E-07
PITA_000030532	Low quality	AT5G63450	MA_18375g0010	shade>sun	AtWRKY33 regulates root apop	CYTOCHROME P450, FAM	chloroplast-GO:0009507,h	17.99839004	1.993345756	0.49036232	4.065046793	4.80E-05	0.000923826
PITA_000030561	High quality	AT5G39660	MA_175298g0010	shade>sun	Dof-type zinc finger domain-co	CYCLING DOF FACTOR 2 (C	DNA binding-GO:0003677,	52.71332081	0.635263028	0.22825207	2.78316433	0.005383153	0.04807225
PITA_000030658	High quality	AT5G16710	MA_86450g0010	shade<sun	DHAR3 protein undergoes thio	DEHYDROASCORBATE REL	acquisition of seed longev	404.4521432	-12.28970089	3.90688717	-3.145650325	0.00165718	0.019180395
PITA_000030667	High quality	AT1G01090	MA_10426096g0010	shade<sun	pyruvate dehydrogenase E1 al	PYRUVATE DEHYDROGEN/	acetyl-CoA biosynthetic pr	95.2026554	-0.509381	0.17133204	-2.973063377	0.002948436	0.030524986
PITA_000030884	High quality	AT5G15220	MA_80391g0020	shade<sun	Ribosomal protein L27 family protein;(source:Araport11)		chloroplast-GO:0009507,s	272.3501129	-0.758233911	0.18351469	-4.131734063	3.60E-05	0.000710495
PITA_000030909	High quality	AT2G23810	MA_24323g0010	shade<sun	Member of TETRASPANIN fam	TETRASPANIN8 (TET8)	defense response to Gram	430.9148101	-0.887732189	0.2271839	-3.907548869	9.32E-05	0.001642068
PITA_000030941	Low quality	AT2G36530	MA_10428568g0010	shade<sun	Involved in light-dependent co	LOW EXPRESSION OF OSM	apoplast-GO:0048046,chlc	64.77271212	-1.009035144	0.28329821	-3.56174202	0.000368402	0.005442944
PITA_000031075	High quality	AT1G47710	MA_2450g0010	shade<sun	Serine protease inhibitor (SERI	(SERPIN1)	apoplast-GO:0048046,cyst	85.8417577	-1.400670051	0.27566707	-5.081020654	3.75E-07	1.13E-05
PITA_000031116	High quality	AT5G57870	MA_179075g0010	shade<sun	Encodes a putative eukaryotic	EUKARYOTIC TRANSLATIO	cytoplasm-GO:0005737,cy	266.9646058	-11.69038391	3.90695024	-2.992201892	0.00276973	0.029056821
PITA_000031137	Low quality	AT3G61870	MA_80427g0010	shade<sun	plant/protein;(source:Araport11)		biological_process_unkno	1172.793614	-1.454977802	0.21806022	-6.672366988	2.52E-11	1.56E-09
PITA_000031224	Low quality	AT1G21910	MA_10020434g0010	shade>sun	encodes a member of the DREI	DEHYDRATION RESPONSE	cellular response to freezi	19.75679402	7.597921485	1.2909713	5.885430204	3.97E-09	1.67E-07
PITA_000031250	Low quality	AT3G03280	MA_10427211g0020	shade<sun	PADRE protein up-regulated after infection by S. sclerotio		cellular response to hypox	47.25025359	-2.408797221	0.72857951	-3.306155614	0.000945855	0.012047714
PITA_000031332	Low quality	AT1G32520	MA_93555g0010	shade<sun	TLDc domain protein;(source:A	OXIDATION RESISTANCE 4	biological_process_unkno	461.1461225	-1.200308053	0.22393733	-5.360017742	8.32E-08	2.82E-06

PITA_000031421	Low quality	AT1G64850	MA_10132073g0010	shade>sun	Calcium-binding EF hand family	protein;(source:Araport11)	biological_process_unkno	77.94156024	0.564984066	0.17186231	3.28742268	0.00101109	0.012718043
PITA_000031445	High quality	AT5G10020	MA_6221g0010	shade<sun	Leucine-rich receptor-like prot	SUCROSE-INDUCED RECEP	cytosol-GO:0005829,plasm	118.014331	0.898879658	0.28169774	3.190936687	0.001418124	0.016947579
PITA_000031479	High quality	AT4G28740	MA_173127g0010	shade<sun	LOW PSII ACCUMULATION-like	protein;(source:Araport11	chloroplast-GO:0009507,c	669.3618598	-1.655684978	0.27521121	-6.016052169	1.79E-09	7.98E-08
PITA_000031517	High quality	AT1G50260	MA_10300g0030	shade<sun	N-terminal-transmembrane-C2	N-TERMINAL-TRANSMEMI	plasma membrane-GO:000	144.0360593	-1.053412745	0.17620931	-5.978190077	2.26E-09	9.86E-08
PITA_000031565	Low quality	AT5G50200	MA_108756g0010	shade<sun	Wound-responsive gene 3 (WF	WOUND-RESPONSIVE 3 (V	nitrate transmembrane tra	335.4365037	-2.138761654	0.39670359	-5.391334244	6.99E-08	2.41E-06
PITA_000031614	High quality	AT1G23400	MA_757065g0010	shade<sun	Promotes the splicing of chlorc	CHLOROPLAST RNA SPLICI	chloroplast-GO:0009507,G	295.1445094	-1.125156256	0.2308905	-4.873117629	1.10E-06	3.07E-05
PITA_000031624	High quality	AT1G32240	MA_138039g0010	shade>sun	Encodes a member of the KAN	KANADI 2 (KAN2)	carpel development-GO:0	57.98885637	0.594889747	0.2116539	2.81067233	0.004943811	0.045438376
PITA_000031694	High quality	AT1G64510	MA_10429326g0010	shade<sun	Translation elongation factor E	PLASTID RIBOSOMAL PRO	chloroplast-GO:0009507,c	302.8686059	-1.06099073	0.11429665	-9.282780769	1.65E-20	4.29E-18
PITA_000031809	Low quality	AT4G28100	MA_141029g0010	shade<sun	transmembrane protein;(source:Araport11)		anchored component of m	298.5138135	-1.069211279	0.11225413	-9.524916738	1.65E-21	4.79E-19
PITA_000031810	High quality	AT5G03300	MA_255g0010	shade<sun	Encodes adenosine kinase 2 (A	ADENOSINE KINASE 2 (AD	adenosine kinase activity-(	127.8411391	-0.495733413	0.16473473	-3.009282948	0.002618651	0.027644362
PITA_000031874	High quality	AT5G48460	MA_10429910g0010	shade<sun	Encodes a member of the fimb	(ATFIM2)	actin filament-GO:000588	119.7887745	-1.14303925	0.31679279	-3.6081606	0.000308376	0.004665406
PITA_000031878	High quality	AT1G70520	MA_101258g0010	shade>sun	Encodes a cysteine-rich receptor-like protein kinase. Invol		kinase activity-GO:001630	119.681759	0.933787222	0.31014405	3.010817817	0.002605451	0.027539598
PITA_000031884	High quality	AT4G18750	MA_10257685g0010	shade<sun	Encodes a pentatricopeptide (I	DEFECTIVELY ORGANIZED	chloroplast-GO:0009507,c	328.19236	-1.809820526	0.41899896	-4.319391485	1.56E-05	0.000341048
PITA_000031933	High quality	AT5G26340	MA_130810g0010	shade<sun	Encodes a protein with high af	(MSS1)	carbohydrate transmemb	162.6399848	-0.802024118	0.27014526	-2.968862415	0.002989044	0.030768853
PITA_000031953	High quality	AT2G01170	MA_10435539g0010	shade<sun	Encodes a bidirectional amino	BIDIRECTIONAL AMINO A	arginine transmembrane t	114.7831475	-0.67186518	0.22313717	-3.010996304	0.00260392	0.027539598
PITA_000031970	Low quality	AT2G26975	MA_10434905g0010	shade>sun	Ctr copper transporter family;(	COPPER TRANSPORTER 6	cellular copper ion homeo	41.84865416	2.894975766	0.37738765	7.671092944	1.71E-14	1.93E-12
PITA_000031992	High quality	AT2G22900	MA_48841g0010	shade<sun	Encodes MUCI10, a galactomal	MUCILAGE-RELATED10 (M	endosome-GO:0005768,ga	90.62112102	-0.680004725	0.19229039	-3.536342793	0.000405708	0.00590078
PITA_000032112	High quality	AT4G17090	MA_3193g0010	shade<sun	Encodes a beta-amylase target	CHLOROPLAST BETA-AMY	amylopectin maltohydrola	2725.974386	-1.885124506	0.26825939	-7.027245063	2.11E-12	1.64E-10
PITA_000032214	High quality	AT1G62350	MA_10430982g0010	shade<sun	Pentatricopeptide repeat (PPR)	superfamily protein;(sour	biological_process_unkno	90.00918142	-1.236057545	0.39273089	-3.14733978	0.001647634	0.0191045
PITA_000032339	High quality	AT2G36460	MA_386405g0010	shade<sun	Aldolase superfamily protein;(s	FRUCTOSE-BISPHOSPHATI	cellular response to hypox	383.3131809	-1.284133804	0.15948227	-8.051890602	8.15E-16	1.16E-13
PITA_000032414	High quality	AT3G03990	MA_10427409g0020	shade>sun	Encodes an alpha/beta hydrola	DWARF 14 (D14)	cellular response to strigo	82.79317568	0.96167738	0.22032472	4.364818426	1.27E-05	0.000284459
PITA_000032437	Low quality	AT1G03230	MA_10135085g0010	shade>sun	Eukaryotic aspartyl protease fa	SECRETED ASPARTIC PRO1	aspartic-type endopeptida	28.75210019	8.141260643	1.73127953	4.702453016	2.57E-06	6.68E-05
PITA_000032471	High quality	AT4G39370	MA_47879g0010	shade<sun	Encodes a ubiquitin-specific pr	UBIQUITIN-SPECIFIC PROT	cysteine-type endopeptida	89.35969792	-0.544451801	0.18312243	-2.97315743	0.002947532	0.030524986
PITA_000032473	High quality	AT1G22640	MA_64405g0010	shade>sun	MYB-type transcription factor (	MYB DOMAIN PROTEIN 3	response to wounding-GO	32.60550225	0.974678088	0.2705652	3.602377813	0.00031532	0.004759043
PITA_000032475	High quality	AT1G09155	MA_6489g0010	shade<sun	phloem protein 2-B15;(source:	PHLOEM PROTEIN 2-B15 (	carbohydrate binding-GO:	104.4876716	-0.761048573	0.27103469	-2.807937849	0.004985985	0.045709392
PITA_000032550	High quality	AT2G44050	MA_10425797g0010	shade<sun	6,7-dimethyl-8-ribityllumazine	COI1 SUPPRESSOR1 (COS1	6,7-dimethyl-8-ribityllum	103.7404184	-0.456991028	0.14929427	-3.061008436	0.002205929	0.024278671
PITA_000032638	Low quality	AT5G40500	MA_10431239g0010	shade<sun	hypothetical protein;(source:Araport11)		biological_process_unkno	349.2860262	-0.634709259	0.13476331	-4.709807682	2.48E-06	6.48E-05
PITA_000032639	High quality	AT1G22340	MA_10293821g0010	shade<sun	UDP-glucosyl transferase 85A7	UDP-GLUCOSYL TRANSFEF	cytosol-GO:0005829,glucu	941.8599273	-1.544551901	0.27144143	-5.690184827	1.27E-08	4.87E-07
PITA_000032653	High quality	AT1G80830	MA_100211g0010	shade<sun	Thought to be involved in iron	NATURAL RESISTANCE-AS	cadmium ion transmembr	219.6872002	-11.40954103	1.57486096	-7.244792597	4.33E-13	3.87E-11
PITA_000032765	High quality	AT3G07990	MA_10428464g0010	shade<sun	serine carboxypeptidase-like 2	SERINE CARBOXYPEPTIDA	extracellular region-GO:0	134.8482112	-0.700986207	0.14010038	-5.003456794	5.63E-07	1.65E-05
PITA_000032817	Low quality	AT1G69380	MA_10429629g0010	shade<sun	Encodes a mitochondria-locali	RETARDED ROOT GROWTI	mitochondrion-GO:00057	119.5780992	-1.0640809	0.25957333	-4.099345973	4.14E-05	0.000808131
PITA_000032858	High quality	AT3G04070	MA_10436448g0010	shade>sun	NAC domain containing protei	NAC DOMAIN CONTAININ	DNA binding-GO:0003677,	8.961547724	6.460702949	1.56227083	4.135456433	3.54E-05	0.000702925
PITA_000032921	High quality	AT2G37050	MA_101110g0010	shade<sun	Leucine-rich repeat protein kinase family protein;(source:		ATP binding-GO:0005524,(	238.2482231	-0.70120185	0.16059946	-4.366153166	1.26E-05	0.000283688
PITA_000032959	Low quality	AT4G11960	MA_117727g0010	shade<sun	Encodes PGRL1B, a transmemb	PGR5-LIKE B (PGRL1B)	chloroplast-GO:0009507,c	351.5386494	-0.962821477	0.14086009	-6.835303626	8.18E-12	5.67E-10
PITA_000033097	High quality	AT2G37130	MA_10432809g0020	shade<sun	Peroxidase superfamily protein;(source:Araport11)		cytosol-GO:0005829,defer	284.3600461	-2.442167994	0.54049683	-4.518376145	6.23E-06	0.000150105
PITA_000033204	High quality	AT2G35720	MA_10433567g0010	shade<sun	Encodes OWL1, a J-domain pr	ORIENTATION UNDER VEF	chloroplast-GO:0009507,e	97.36244052	-0.847562423	0.27384454	-3.09504951	0.0019678	0.022185037
PITA_000033273	High quality	AT5G52450	MA_10434642g0010	shade>sun	MATE efflux family protein;(source:Araport11)		antiporter activity-GO:001	16.96613843	1.598251428	0.4258339	3.75322728	0.000174572	0.002840463
PITA_000033329	High quality	AT1G19715	MA_1015570g0010	shade<sun	Mannose-binding lectin superfamily protein;(source:Arap		extracellular region-GO:0	215.1750728	-0.82525945	0.2948752	-2.798673622	0.005131297	0.046483952
PITA_000033347	High quality	AT4G15920	MA_930638g0010	shade>sun	Encodes a vacuolar fructose tr	(SWEET17)	carbohydrate export-GO:0	41.28057167	0.848685863	0.24627095	3.446146813	0.000568641	0.007892754
PITA_000033409	High quality	AT4G29670	MA_10436837g0010	shade<sun	Encodes a member of the thior	ATYPICAL CYS HIS RICH TI	chloroplast-GO:0009507,o	144.869526	-0.632264658	0.16842545	-3.753973443	0.000174053	0.002833844
PITA_000033440	High quality	AT2G04850	MA_10431938g0010	shade<sun	Auxin-responsive family protein;(source:Araport11)		extracellular region-GO:0	395.0209257	-1.456346948	0.26988208	-5.396234311	6.81E-08	2.35E-06
PITA_000033503	High quality	AT1G11545	MA_10436348g0010	shade>sun	xyloglucan endotransglucosyla	XYLOGLUCAN ENDOTRAN:	cell wall biogenesis-GO:00	30.4652795	1.301030013	0.29226782	4.451499417	8.53E-06	0.000199128
PITA_000033577	High quality	AT2G46080	MA_7578826g0010	shade<sun	Encodes a protein related to B	BYPASS 2 (BPS2)	embryo development end	49.24995917	-9.252776687	1.45975043	-6.338601777	2.32E-10	1.21E-08
PITA_000033647	High quality	AT3G14200	MA_204527g0010	shade<sun	Chaperone DnaJ-domain superfamily protein;(source:Araj		biological_process_unkno	1760.00763	-1.696010258	0.31391046	-5.402847302	6.56E-08	2.28E-06
PITA_000033657	High quality	AT5G07280	MA_177039g0010	shade>sun	Encodes EMS1 (EXCESS MICRO	EXCESS MICROSPOROCYTI	anther wall tapetum cell fa	36.74195079	8.494827337	1.70721775	4.975831204	6.50E-07	1.88E-05
PITA_000033675	High quality	AT1G53280	MA_10433067g0010	shade<sun	Encodes a homolog of animal I	DJ-1 HOMOLOG B (DJ1B)	chloroplast-GO:0009507,c	359.9653482	-0.895737976	0.16209087	-5.526146975	3.27E-08	1.20E-06
PITA_000033783	High quality	AT2G46510	MA_10436183g0010	shade>sun	Encodes a nuclear localized BL	ABA-INDUCIBLE BHLH-TYP	DNA-binding transcription	106.9530297	1.771640678	0.39136051	4.526876432	5.99E-06	0.000144609
PITA_000033943	Low quality	AT3G03580	MA_519985g0010	shade<sun	Tetratricopeptide repeat (TPR)	(MEF26)	intracellular membrane-bc	295.2311221	-1.659516828	0.35864016	-4.627247281	3.71E-06	9.36E-05
PITA_000033967	High quality	AT2G31170	MA_10431763g0010	shade<sun	Encodes the cysteinyl t-RNA sy	CYSTEINYL T-RNA SYNTHE	ATP binding-GO:0005524,(	258.5373234	-0.852446995	0.1031388	-8.26504697	1.40E-16	2.36E-14
PITA_000033974	High quality	AT2G12646	MA_18612g0010	shade>sun	Plant AT-rich sequence and zin	RGF1-INDUCIBLE TRANSCF	maintenance of root meris	20.50330553	0.965250622	0.33661052	2.867559256	0.004136513	0.039501275
PITA_000034022	High quality	AT5G52060	MA_10425833g0010	shade<sun	A member of Arabidopsis BAG	BCL-2-ASSOCIATED ATHAN	adenyl-nucleotide exchang	153.9333132	-0.601598937	0.15460273	-3.891256984	9.97E-05	0.001741799
PITA_000034197	High quality	AT5G37810	MA_10426635g0010	shade<sun	NOD26-like intrinsic protein 4;	NOD26-LIKE INTRINSIC PR	channel activity-GO:00152	192.6406241	-0.900963605	0.21151294	-4.259614618	2.05E-05	0.000434726
PITA_000034223	High quality	AT3G08500	MA_137886g0010	shade<sun	Encodes a putative R2R3-type I	MYB DOMAIN PROTEIN 83	DNA-binding transcription	506.652477	-2.45619814	0.80791056	-3.040185717	0.002364323	0.025602907
PITA_000034245	High quality	AT4G04930	MA_469702g0010	shade<sun	Encodes a sphingolipid delta4-	(DES-1-LIKE)	ceramide biosynthetic pro	120.8203621	-0.853204783	0.2383033	-3.580331331	0.000343159	0.005127053
PITA_000034273	High quality	AT3G53000	MA_4735g0010	shade>sun	phloem protein 2-A15;(source:	PHLOEM PROTEIN 2-A15 (	carbohydrate binding-GO:	62.51983891	3.149126127	1.03884002	3.031387009	0.00243433	0.026180727
PITA_000034298	Low quality	AT1G26220	MA_460253g0010	shade<sun	Catalyzes formation of N-acety	SEROTONIN N-ACETYLTRA	chloroplast-GO:0009507,N	156.6900127	-0.611048826	0.15330114	-3.985937939	6.72E-05	0.001232828
PITA_000034350	High quality	AT5G18610	MA_10430833g0010	shade>sun	Encodes a receptor-like cytopl	(PBL27)	activation of MAPK activity	54.90574756	9.074905428	1.31241992	6.914635535	4.69E-12	3.39E-10
PITA_000034362	High quality	AT2G21860	MA_89112g0010	shade<sun	violaxanthin de-epoxidase-like	protein;(source:Araport11)	chloroplast-GO:0009507,m	389.64183	-1.591136085	0.1898932	-8.379110423	5.33E-17	9.15E-15
PITA_000034403	Low quality	AT5G62140	MA_422522g0010	shade<sun	ATP-dependent Clp protease	ATP-binding subunit;(sourc	chloroplast-GO:0009507,p	665.0255319	-1.318663025	0.25000911	-5.274459821	1.33E-07	4.33E-06
PITA_000034420	High quality	AT2G36690	MA_480690g0010	shade<sun	Protein belonging to the Fe-de	GERMINATION INSENSITIV	2-oxoglutarate-dependent	303.9892562	-11.87776699	3.90692764	-3.040180951	0.00236436	0.025602907
PITA_000034498	High quality	AT3G17390	MA_141759g0010	shade<sun	S-adenosylmethionine synthet	METHIONINE OVER-ACCU	cell wall-GO:0005618,cyto	367.4274928	-12.15118855	3.90689951	-3.110186612	0.001869692	0.021221254
PITA_000034670	Low quality	AT4G24190	MA_10431031g0010	shade<sun	encodes an ortholog of GRP94,	SHEPHERD (SHD)	ATPase activity-GO:00168	91.46486634	-0.759135124	0.19508768	-3.891251024	9.97E-05	0.001741799
PITA_000034721	High quality	AT4G39350	MA_82434g0010	shade<sun	Encodes a cellulose synthase is	CELLULOSE SYNTHASE A2	cellulose biosynthetic pro	147.6815218	-3.541688057	0.43639458	-8.115793018	4.83E-16	7.15E-14
PITA_000034765	High quality	AT5G62930	MA_3400914g0010	shade<sun	SGNH hydrolase-type esterase	superfamily protein;(sourc	cytoplasm-GO:0005737,cy	88.69316172	-0.473732897	0.16889793	-2.804847323	0.005034042	0.045949524
PITA_000034924	High quality	AT4G37970	MA_10253390g0010	shade>sun	cinnamyl alcohol dehydrogena	CINNAMYL ALCOHOL DEH	cinnamyl-alcohol dehydrog	89.08424794	0.782757741	0.25271536	3.097388862	0.001952336	0.022030385
PITA_000035179	High quality	AT2G17880	MA_56308g0010	shade>sun	Chaperone DnaJ-domain super	DNA J PROTEIN C24 (DJC2	chloroplast-GO:0009507	33.86288787	1.647736165	0.30732825	5.361486093	8.25E-08	2.80E-06
PITA_000035220	Low quality	AT1G24620	MA_8414519g0010	shade>sun	Encodes a EF-hand calcium-bin	CALMODULIN LIKE 25 (CM	calcium ion binding-GO:00	15.24049925	1.678167971	0.42936585	3.908480326	9.29E-05	0.001638039
PITA_000035221	Low quality	AT4G08250	MA_73173g0010	shade<sun	GRAS family transcription factor;(source:Araport11)		DNA-binding transcription	172.8659481	-1.38292412	0.39949973	-3.461639709	0.000536895	0.007514152
PITA_000035227	High quality	AT3G27740	MA_135287g0010	shade<sun	Encodes carbamoyl phosphate	CARBAMOYL PHOSPHATE	arginine biosynthetic proc	126.9016217	-0.551457537	0.16014064	-3.443332891	0.000574592	0.007949095
PITA_000035352	High quality	AT5G24930	MA_54929g0010	shade<sun	Flowering repressor in long da	CONSTANS-LIKE 4 (COL4)	circadian regulation of ger	689.7082653	-0.561575261	0.12927711	-4.343965002	1.40E-05	0.000309302
PITA_000035495	High quality	AT2G46410	MA_9991g0010	shade<sun	Nuclear-localized R3-type MYB	CAPRICE (CPC)	cell differentiation-GO:003	55.59552822	-1.223084112	0.37462272	-3.264842311	0.00109525	0.013607002
PITA_000035534	High quality	AT5G04770	MA_84286g0010	shade>sun	Encodes a member of the catic	CATIONIC AMINO ACID TF	amino acid transmembran	272.0281691	11.38366644	1.45958467	7.799250463	6.23E-15	7.59E-13
PITA_000035566	Low quality	AT1G25360	MA_622231g0010	shade<sun	DYW-type pentatricopeptide re	D ORGANELLAR TRANSCRI	intracellular membrane-bc	242.6912424	-1.335048689	0.38077397	-3.506144841	0.000454648	0.006511196
PITA_000035567	Low quality	AT5G02120	MA_10117352g0010	shade<sun	Encodes a one helix protein hc	ONE-HELIX LHC-LIKE PROT	chloroplast-GO:0009507,c	225.9276831	-0.925350108	0.13579325	-6.814404208	9.47E-12	6.47E-10
PITA_000035798	High quality	AT4G11240	MA_10430886g0020	shade<sun	encodes a type I serine/threon	(TOPP7)	cytoplasm-GO:0005737,mi	121.5521504	-0.474983937	0.14841828	-3.200306199	0.001372817	0.01645553
PITA_000035848	Low quality	AT4G27030	MA_368556g0010	shade<sun	Encodes an unusual palmitate	FATTY ACID DESATURASE	chloroplast-GO:0009507,c	14543.99953	-3.009812824	0.28214194	-10.66772583	1.44E-26	6.99E-24
PITA_000035943	High quality	AT5G65030	MA_8319422g0010	shade>sun	nitric oxide synthase-interacting	protein;(source:Araport1	biological_process_unkno	20.63200067	7.661678394	1.61219423	4.752329632	2.01E-06	5.33E-05
PITA_000035972	High quality	AT4G23160	MA_10604g0010	shade>sun	Encodes a cysteine-rich recept	CYSTEINE-RICH RLK (RECEI	ATP binding-GO:0005524,(	45.29030926	8.797813842	1.84842896	4.759616966	1.94E-06	5.17E-05
PITA_000036005	High quality	AT4G21970	MA_737152g0010	shade>sun	senescence regulator (Protein	of unknown function, DUF5	biological_process_unkno	29.63255725	1.753841581	0.36802999	4.765485492	1.88E-06	5.03E-05
PITA_000036022	High quality	AT4G29070	MA_99242g0010	shade<sun	Phospholipase A2 family protein;(source:Araport11)		arachidonic acid secretion	186.7148696	-0.629450188	0.21561676	-2.919301779	0.003508164	0.034766771
PITA_000036052	High quality	AT2G28720	MA_10428006g0010	shade>sun	Histone superfamily protein;(s	(HTB3)	DNA binding-GO:0003677,	90.69318658	1.456278313	0.42700405	3.410455523	0.000648545	0.008779546
PITA_000036084	High quality	AT5G55580	MA_161180g0010	shade<sun	Encodes a mitochondrial transc	(MTERF9)	chloroplast-GO:0009507,c	667.2828676	-1.545245445	0.25373891	-6.089903274	1.13E-09	5.20E-08
PITA_000036106	High quality	AT5G13870	MA_10428415g0010	shade>sun	EXGT-A4, endoxyloglucan tran:	XYLOGLUCAN ENDOTRAN:	cell wall biogenesis-GO:00	42.05713242	2.388808323	0.72568014	3.29181989	0.000995413	0.012556297
PITA_000036151	High quality	AT1G02850	MA_139193g0010	shade<sun	beta glucosidase 11;(source:Ar	BETA GLUCOSIDASE 11 (B	beta-glucosidase activity-C	249.7084923	-1.207745813	0.22492641	-5.369515279	7.89E-08	2.69E-06
PITA_000036164	High quality	AT2G19580	MA_391725g0010	shade<sun	Member of TETRASPANIN fam	TETRASPANIN2 (TET2)	chloroplast-GO:0009507,m	164.7064786	-0.676129194	0.22698577	-2.978729445	0.002894462	0.030068835
PITA_000036169	High quality	AT5G20810	MA_132100g0010	shade<sun	SAUR-like auxin-responsive prc	SMALL AUXIN UPREGULAT	nucleus-GO:0005634,pero	408.9492426	-2.286655832	0.31855284	-7.178262267	7.06E-13	6.18E-11
PITA_000036224	High quality	AT2G28150	MA_10435302g0010	shade>sun	DUF966 domain containing pro	SOSEKI3 (SOK3)	extrinsic component of cyt	55.20924881	0.720005541	0.22892858	3.145109826	0.001660245	0.019206934
PITA_000036228	High quality	AT2G01860	MA_10281691g0010	shade<sun	Tetratricopeptide repeat (TPR)	EMBRYO DEFECTIVE 975 (	chloroplast-GO:0009507,m	292.1132996	-1.071074481	0.16181716	-6.619041597	3.62E-11	2.19E-09
PITA_000036283	High quality	AT4G00350	MA_123647g0010	shade<sun	MATE efflux family protein;(source:Araport11)		antiporter activity-GO:001	390.7204822	-1.72228512	0.34007533	-5.064422396	4.10E-07	1.22E-05
PITA_000036284	High quality	AT1G15410	MA_9130g0010	shade<sun	aspartate-glutamate racemase family;(source:Araport11)		amino-acid racemase activ	633.9351248	-0.981773543	0.26530839	-3.700499426	0.000215176	0.003406609
PITA_000036343	High quality	AT5G10970	MA_48488g0010	shade<sun	C2H2 and C2HC zinc fingers superfamily protein;(source:A		chloroplast-GO:0009507,D	299.444724	-0.702727553	0.18311864	-3.837553297	0.000124266	0.002103353
PITA_000036379	High quality	AT1G28140	MA_10430187g0010	shade<sun	integral membrane family protein;(source:Araport11)		biological_process_unkno	146.8757062	-0.448672045	0.15496085	-2.895389673	0.003786881	0.036988824
PITA_000036388	Low quality	AT3G01300	MA_120879g0010	shade>sun	mediator of RNA polymerase II	transcription subunit;(sou	biological_process_unkno	51.86453599	2.398761485	0.68291233	3.512546726	0.000443834	0.006374936
PITA_000036407	High quality	AT3G01120	MA_2215g0010	shade<sun	encodes a cystathionine gamm	METHIONINE OVERACCUM	chloroplast-GO:0009507,c	167.8222745	-0.822417126	0.12431304	-6.615694596	3.70E-11	2.22E-09
PITA_000036512	High quality	AT5G44650	MA_7653147g0010	shade<sun	Encodes a nucleus-encoded thr	CHLOROPLAST PROTEIN-E	chloroplast-GO:0009507,c	239.9462486	-0.627333085	0.17297823	-3.626659093	0.000287112	0.004396456
PITA_000036543	Low quality	AT1G20140	MA_165814g0010	shade>sun	SKP1-like 4;(source:Araport11)	SKP1-LIKE 4 (SK4)	cullin family protein bindir	87.87778529	9.753591687	1.50001644	6.502323182	7.91E-11	4.45E-09
PITA_000036642	High quality	AT3G16770	MA_19532g0010	shade>sun	Encodes a member of the ERF	ETHYLENE-RESPONSIVE EL	cell death-GO:0008219,cyt	34.06462841	1.125720562	0.33762599	3.33422366	0.000855378	0.011085635
PITA_000036786	High quality	AT5G13800	MA_69602g0010	shade<sun	Encodes a pheophytinase that	PHEOPHYTINASE (PPH)	chlorophyll catabolic proce	256.8553824	-0.763181389	0.21507676	-3.548414016	0.000387559	0.005682689
PITA_000036823	High quality	AT3G06210	MA_10434888g0020	shade>sun	ARM repeat superfamily protein;(source:Araport11)		biological_process_unkno	8.965490001	3.998945272	1.41959546	2.816961169	0.004848039	0.044813489
PITA_000036832	High quality	AT5G53090	MA_90899g0010	shade<sun	NAD(P)-binding Rossmann-fold	superfamily protein;(sourc	cellular_component_unkn	69.14788149	-1.174025771	0.35845379	-3.27524997	0.001055685	0.013193449
PITA_000036860	Low quality	AT4G17600	MA_10427346g0010	shade<sun	Encodes Lil3:1 (light-harvesting	LIGHT-HARVESTING-LIKE 3	chloroplast-GO:0009507,c	236.2881018	-0.977238161	0.11760484	-8.309506007	9.61E-17	1.64E-14
PITA_000036885	High quality	AT1G68920	MA_96671g0010	shade>sun	basic helix-loop-helix (bHLH) D	CIB1 LIKE PROTEIN 1 (CIL1	DNA-binding transcription	15.50637102	1.980773817	0.57970576	3.416860695	0.000633477	0.008612553
PITA_000037012	Low quality	AT1G20830	MA_166041g0010	shade<sun	Encodes MCD1 (MULTIPLE CHL	MULTIPLE CHLOROPLAST	chloroplast-GO:0009507,c	180.6308602	-0.675031143	0.12921756	-5.223989189	1.75E-07	5.51E-06
PITA_000037098	High quality	AT5G24120	MA_10429477g0010	shade<sun	Encodes a specialized sigma fa	SIGMA FACTOR E (SIGE)	cellular response to blue li	333.5655288	-0.89922736	0.26178835	-3.43494033	0.000592684	0.008154664
PITA_000037246	High quality	AT2G39190	MA_10427105g0010	shade<sun	member of ATH subfamily	(ATATH8)	chloroplast-GO:0009507,p	178.1482064	-1.063936503	0.36520185	-2.913283434	0.003576498	0.035316869
PITA_000037427	High quality	AT3G54090	MA_10430836g0020	shade<sun	Encodes a fructokinase-like pro	FRUCTOKINASE-LIKE 1 (FL	chloroplast nucleoid-GO:0	754.3579236	-1.457437083	0.15075877	-9.667345446	4.15E-22	1.29E-19
PITA_000037445	High quality	AT1G76880	MA_79519g0010	shade>sun	DF1 is a putative transcription	(DF1)	DNA-binding transcription	43.49474375	0.65599136	0.2288924	2.86593772	0.00415776	0.039659121
PITA_000037508	High quality	AT2G26930	MA_10430180g0010	shade<sun	Encodes a 4-(cytidine 5'-phosp	4-(CYTIDINE 5'-PHOSPHO)	4-(cytidine 5'-diphospho)-2	177.6684623	-0.65388936	0.15738555	-4.154697428	3.26E-05	0.000655073
PITA_000037580	High quality	AT1G08250	MA_7947g0030	shade<sun	Encodes a plastid-localized aro	AROGENATE DEHYDRATAS	arogenate dehydratase act	477.355487	-1.320046361	0.21301636	-6.196924705	5.76E-10	2.77E-08
PITA_000037608	High quality	AT1G10750	MA_1134g0010	shade>sun	carboxyl-terminal peptidase, putative (DUF239);(source:A		mitochondrion-GO:00057	40.77986428	1.17478908	0.32370549	3.62919105	0.000284311	0.004356208
PITA_000037671	High quality	AT2G18950	MA_10436616g0010	shade<sun	Encodes homogentisate phytyl	HOMOGENTISATE PHYTYL	cell wall organization-GO:(	398.0310028	-0.926782395	0.15513203	-5.974152543	2.31E-09	1.01E-07
PITA_000037690	High quality	AT1G02180	MA_15462g0010	shade>sun	ferredoxin-like protein;(source:Araport11)		biological_process_unkno	88.17092128	0.692462661	0.20713584	3.343036385	0.00082867	0.010806135
PITA_000037787	Low quality	AT5G67620	MA_76415g0010	shade>sun	PADRE protein up-regulated after infection by S. sclerotio		cytoplasm-GO:0005737,mi	43.77411454	0.772280322	0.25563735	3.020999543	0.002519417	0.026900807
PITA_000037825	High quality	AT1G70000	MA_79012g0010	shade<sun	Encodes a MYB-like Domain tra	(MYBD)	chromatin binding-GO:000	120.8889223	-0.522880853	0.1437326	-3.637872423	0.0002749	0.004227418
PITA_000037954	Low quality	AT5G16080	MA_9392760g0010	shade<sun	carboxyesterase 17;(source:Ar	CARBOXYESTERASE 17 (C)	cellular_component_unkn	181.5236263	-0.874643311	0.27322018	-3.201239813	0.001368376	0.016417907
PITA_000038039	High quality	AT3G18890	MA_10429419g0010	shade<sun	NAD(P)-binding Rossmann-fold	TRANSLOCON AT THE INN	chloroplast-GO:0009507,c	922.3988908	-1.576533031	0.09705158	-16.24428055	2.45E-59	8.83E-56
PITA_000038071	Low quality	AT3G50180	MA_10426384g0010	shade<sun	transmembrane protein, putative (DUF247);(source:Arapc		biological_process_unkno	1394.264476	-1.532089151	0.43574461	-3.516025453	0.000438059	0.006305849
PITA_000038176	High quality	AT5G22800	MA_10436405g0010	shade<sun	A locus involved in embryogen	EMBRYO DEFECTIVE 1030	alanine-tRNA ligase activit	288.1705033	-0.917047212	0.24586632	-3.729861007	0.000191585	0.003081495
PITA_000038178	High quality	AT4G38620	MA_2026g0010	shade>sun	Encodes a R2R3 MYB protein w	MYB DOMAIN PROTEIN 4	regulation of phenylpropa	62.68802289	1.417092907	0.49019649	2.890867127	0.003841805	0.037359685
PITA_000038336	High quality	AT5G67060	MA_83500g0010	shade>sun	Encodes a bHLH transcription f	HECATE 1 (HEC1)	carpel formation-GO:0048	23.17120042	1.876258834	0.45071714	4.162830024	3.14E-05	0.000634695
PITA_000038337	High quality	AT2G30020	MA_182759g0010	shade<sun	Encodes AP2C1. Belongs to th	(AP2C1)	abscisic acid-activated sigr	632.799759	-0.970825461	0.23963458	-4.05127446	5.09E-05	0.000973252
PITA_000038419	High quality	AT5G08740	MA_910768g0010	shade<sun	Encodes a dedicated Type II N/	NAD(P)H DEHYDROGENAS	cell wall-GO:0005618,chlo	370.5760192	-0.980309868	0.15680765	-6.251671303	4.06E-10	2.00E-08
PITA_000038430	High quality	AT2G18790	MA_10435530g0010	shade<sun	Red/far-red photoreceptor inv	PHYTOCHROME B (PHYB)	abscisic acid metabolic pro	663.6430997	-0.960534329	0.23382681	-4.107887835	3.99E-05	0.000783063
PITA_000038470	High quality	AT1G32060	MA_210555g0010	shade<sun	phosphoribulokinase;(source:A	PHOSPHORIBULOKINASE (	apoplast-GO:0048046,chlc	698.3221249	-1.294578757	0.11008773	-11.75951955	6.31E-32	4.42E-29
PITA_000038580	High quality	AT4G32600	MA_105489g0010	shade<sun	C3H4 type zinc finger protein;(source:Araport11)		nucleus-GO:0005634,plasr	180.5581273	-0.541650892	0.19233876	-2.816129748	0.004860603	0.044902716
PITA_000038640	High quality	AT2G39210	MA_207936g0010	shade>sun	Major facilitator superfamily in	PICLORAM RESISTANT30 (	chloroplast-GO:0009507,ir	25.84189855	1.415535871	0.49233195	2.875165568	0.004038157	0.038826655
PITA_000038786	High quality	AT1G76590	MA_14971g0010	shade>sun	PLATZ transcription factor fam	(PLATZ2)	biological_process_unkno	34.77986605	1.700200457	0.28910011	5.881009425	4.08E-09	1.71E-07
PITA_000038936	High quality	AT3G02820	MA_10427230g0010	shade>sun	zinc knuckle (CCHC-type) family protein;(source:Araport11)		DNA binding-GO:0003677,	161.4209899	0.531950214	0.15662374	3.396357485	0.000682891	0.009165787
PITA_000038946	High quality	AT1G74960	MA_10428839g0010	shade>sun	Encodes a plastidic beta-ketoa	FATTY ACID BIOSYNTHESI	3-oxoacyl-[acyl-carrier-pro	77.44165258	1.332285334	0.33893494	3.930799639	8.47E-05	0.00150899
PITA_000038969	High quality	AT5G61850	MA_108198g0010	shade>sun	Encodes transcriptional regulat	LEAFY (LFY)	chromatin DNA binding-G	55.04106108	1.061783934	0.28241939	3.759600043	0.000170185	0.002779839
PITA_000038978	High quality	AT3G25500	MA_10431990g0020	shade<sun	Poly-L-proline-containing (PLP)	FORMIN HOMOLOGY 1 (A	actin binding-GO:000377	247.5132903	-0.399971332	0.13819942	-2.894160767	0.003801735	0.037105165
PITA_000039004	Low quality	AT3G13882	MA_4464g0010	shade<sun	Ribosomal protein L34;(source:Araport11)		mitochondrial large ribosc	27.89476399	-8.426384727	1.22144141	-6.898722001	5.25E-12	3.74E-10

PITA_000039014	High quality	AT3G02645	MA_10435738g0010	shade<sun	transmembrane protein,<br>putative<br>(DUF247);(source:Araport11)		biological_process_unkno<br>wn-<br>GO:0008150,molecular_f<br>unction_unknown-<br>GO:0003674,cellular_com<br>ponent_unknown-<br>GO:0005575	252.8909476	-11.61227336	3.90696057	-2.972201322	0.002956727	0.030573458
PITA_000039112	High quality	AT4G20360	MA_942991g0010	shade<sun	Nuclear transcribed, plastid loc	RAB GTPASE HOMOLOG E	apoplast-GO:0048046,chlo	245.3509106	-1.926745929	0.57379937	-3.35787388	0.000785444	0.01031714
PITA_000039123	Low quality	AT2G24395	MA_138264g0010	shade<sun	chaperone protein dnaJ-like protein;(source:Araport11)		biological_process_unknov	243.1429482	-0.774737162	0.12921369	-5.995782176	2.03E-09	8.91E-08
PITA_000039134	High quality	AT4G10300	MA_166295g0010	shade<sun	RmlC-like cupins superfamily p	(TRR14)	chloroplast-GO:0009507,c	333.7278071	-0.562680417	0.13560077	-4.149536978	3.33E-05	0.000667886
PITA_000039265	High quality	AT2G30570	MA_10431532g0010	shade<sun	Encodes PsbW, a protein simila	PHOTOSYSTEM II REACTIC	chloroplast-GO:0009507,c	526.2630121	-0.817494641	0.21288449	-3.840085408	0.000122992	0.002084574
PITA_000039290	High quality	AT1G04690	MA_10425964g0010	shade<sun	potassium channel beta subun	POTASSIUM CHANNEL BET	cytoplasm-GO:0005737,ex	200.4312574	-0.855219516	0.18293661	-4.674950127	2.94E-06	7.54E-05
PITA_000039306	Low quality	AT4G32260	MA_128169g0010	shade<sun	ATPase, F0 complex, subunit B	PIGMENT DEFECTIVE 334	ATP synthesis coupled pro	667.4305132	-0.999978582	0.28612623	-3.49488609	0.000474264	0.006734764
PITA_000039442	High quality	AT4G17910	MA_10434559g0010	shade<sun	transferases, transferring acyl groups;(source:Araport11)		glucosaminyl-phosphatidy	45.79144203	-9.147730146	1.60141622	-5.712275205	1.11E-08	4.32E-07
PITA_000039485	High quality	AT1G42970	MA_161458g0010	shade<sun	Encodes chloroplast localized g	GLYCERALDEHYDE-3-PHOS	apoplast-GO:0048046,chlo	1150.329563	-1.357135887	0.09255473	-14.66306345	1.11E-48	2.34E-45
PITA_000039609	Low quality	AT3G24300	MA_469277g0010	shade<sun	Encodes a plasma membrane l	AMMONIUM TRANSPORT	ammonium transmembrar	689.7504501	-1.543133485	0.24348301	-6.33774604	2.33E-10	1.21E-08
PITA_000039706	High quality	AT5G16010	MA_8985g0010	shade<sun	3-oxo-5-alpha-steroid 4-dehydrogenase family protein;(so		chloroplast-GO:0009507,c	9081.351241	-2.522053732	0.23652205	-10.66308059	1.51E-26	7.21E-24
PITA_000039982	Low quality	AT5G51300	MA_11962g0020	shade<sun	Encodes a nuclear localized spl	ARABIDOPSIS SF1 HOMOL	mRNA binding-GO:000372	89.69941208	-1.862546682	0.66311941	-2.808765127	0.004973191	0.045634824
PITA_000039984	High quality	AT1G71500	MA_9287008g0010	shade<sun	Encodes PSB33, a protein cons	PHOTOSYSTEM B PROTEIN	chloroplast-GO:0009507,c	663.414141	-1.054108137	0.12203693	-8.637616151	5.74E-18	1.16E-15
PITA_000039996	High quality	AT1G68840	MA_178646g0010	shade>sun	Rav2 is part of a complex that l	RELATED TO ABI3/VP1 2 (I	cellular response to hypox	4.953179598	5.599708724	1.53307099	3.652608891	0.000259589	0.004031309
PITA_000040012	High quality	AT1G13260	MA_17571g0010	shade>sun	Encodes an AP2/B3 domain tra	RELATED TO ABI3/VP1 1 (I	cellular response to hypox	26.80043686	1.617677767	0.38642702	4.186243908	2.84E-05	0.000581986
PITA_000040017	High quality	AT5G24650	MA_10433953g0010	shade<sun	HP30/Tric1 is a component of t	HYPOTHETICAL PROTEIN 3	chloroplast-GO:0009507,c	123.8951944	-0.663575694	0.14779598	-4.489808815	7.13E-06	0.00016993
PITA_000040032	High quality	AT4G36810	MA_1011718g0010	shade<sun	Encodes a protein with geranyl	GERANYLGERANYL PYROP	chloroplast-GO:0009507,c	179.5156851	-0.45896948	0.13942324	-3.291915283	0.000995076	0.012556297
PITA_000040044	High quality	AT2G47420	MA_40677g0020	shade<sun	Encodes a putative rRNA dimet	ADENOSINE DIMETHYL TR	cell division-GO:0051301,c	138.0722749	-0.62324914	0.1936739	-3.218033739	0.001290726	0.015657103
PITA_000040066	High quality	AT2G45770	MA_375589g0010	shade<sun	Cell division		protein import into<br>chloroplast thylakoid<br>membrane-<br>GO:0045038,SRP-<br>dependent<br>cotranslational protein<br>targeting to membrane-<br>GO:0006614,<br>membrane-<br>GO:0016020,thylakoid<br>membrane organization-<br>GO:0010027,signal<br>recognition particle<br>binding-<br>GO:0005047,chloroplast-<br>GO:0009507,<br>chloroplast thylakoid-<br>GO:0009534,GTPase<br>activity-<br>GO:0003924,photosynthe<br>tic electron transport in<br>photosystem II-<br>GO:0009772,protein<br>binding-<br>GO:0005515,cytosol-<br>GO:0005829,protein<br>targeting-GO:0006605	292.0009087	-0.623620204	0.20768542	-3.002715324	0.002675826	0.028200724
PITA_000040067	High quality	AT1G25560	MA_23673g0010	shade>sun	Encodes a member of the RAV	TEMPRANILLO 1 (TEM1)	cellular response to hypox	38.42560492	0.948377542	0.2747472	3.451818771	0.000556822	0.007745748
PITA_000040112	High quality	AT3G13080	MA_8849g0010	shade>sun	encodes an ATP-dependent MI	ATP-BINDING CASSETTE C	ABC-type glutathione S-co	12.8326839	1.311029196	0.46838887	2.799018684	0.005125817	0.04648192
PITA_000040134	Low quality	AT3G14200	MA_5156g0010	shade>sun	Chaperone DnaJ-domain superfamily protein;(source:Arap		biological_process_unknov	39.44611596	0.900516367	0.24874876	3.620184337	0.000294393	0.004488873
PITA_000040167	Low quality	AT5G20820	MA_245557g0010	shade<sun	SAUR-like auxin-responsive prc	SMALL AUXIN UPREGULAT	cytoplasm-GO:0005737,ex	144.0425248	-2.063036616	0.63855534	-3.230787524	0.001234497	0.015047853
PITA_000040191	Low quality	AT1G79600	MA_10427314g0010	shade<sun	Encodes a chloroplast ABC1-lik	ABC1-LIKE KINASE 3 (ABC1	cellular response to nitrog	448.15761	-1.272899886	0.20652671	-6.163366953	7.12E-10	3.35E-08
PITA_000040251	Low quality	AT2G46225	MA_141074g0010	shade<sun	Encodes a subunit of the WAVI	ABI-1-LIKE 1 (ABIL1)	actin nucleation-GO:0045(	197.4632548	-0.543753316	0.15417931	-3.526759218	0.000420679	0.006083445
PITA_000040371	Low quality	AT2G46420	MA_115256g0010	shade<sun	helicase with zinc finger protein;(source:Araport11)		biological_process_unknov	155.3099147	-0.537712233	0.16441533	-3.270450743	0.001073762	0.013392823
PITA_000040386	High quality	AT3G01470	MA_19453g0040	shade<sun	Encodes a homeodomain leuci	HOMEOBOX 1 (HB-1)	DNA binding-GO:0003677,	142.5577971	-0.417858926	0.13266686	-3.149685825	0.001634461	0.018995905
PITA_000040447	High quality	AT5G03760	MA_96942g0010	shade<sun	encodes a beta-mannan synthe	(ATCSLA09)	DNA mediated transforma	461.3283249	-0.698218535	0.16127245	-4.329434597	1.49E-05	0.000327844
PITA_000040635	High quality	AT5G49300	MA_10436047g0010	shade<sun	Encodes a member of the GAT.	GATA TRANSCRIPTION FA(	DNA-binding transcription	149.805358	-0.76307692	0.21437664	-3.559515248	0.00037154	0.005480869
PITA_000040661	Low quality	AT2G27680	MA_31479g0010	shade<sun	NAD(P)-linked oxidoreductase superfamily protein;(source		chloroplast-GO:0009507,c	274.8418864	-1.605308896	0.2148882	-7.470437518	7.99E-14	8.06E-12
PITA_000040698	High quality	AT3G13540	MA_53373g0010	shade<sun	Encodes a member of the MYB	MYB DOMAIN PROTEIN 5	cell differentiation-GO:003	2421.793665	-2.964663592	0.42019311	-7.055478908	1.72E-12	1.36E-10
PITA_000040853	High quality	AT5G14320	MA_10431241g0010	shade<sun	Ribosomal protein S13/S18 fan	EMBRYO DEFECTIVE 3137	chloroplast-GO:0009507,c	380.7809568	-0.945087493	0.14377296	-6.573471554	4.92E-11	2.89E-09
PITA_000040884	High quality	AT3G51030	MA_836269g0010	shade<sun	encodes a cytosolic thioredoxin	THIOREDOXIN H-TYPE 1 (T	cell redox homeostasis-GC	49.21003725	-1.039985467	0.26300596	-3.954227808	7.68E-05	0.00138219
PITA_000040956	Low quality	AT3G22850	MA_10432644g0010	shade>sun	aluminum induced protein with YGL and LRDR motifs;(sou		cytoplasm-GO:0005737,cy	27.50824033	2.274385104	0.39478974	5.761003577	8.36E-09	3.32E-07
PITA_000040962	High quality	AT1G17220	MA_4340g0010	shade<sun	Encodes a chloroplast localizec	FU-GAERI1 (FUG1)	chloroplast-GO:0009507,c	348.3567256	-1.040007726	0.10519205	-9.886751915	4.75E-23	1.71E-20
PITA_000040976	High quality	AT5G01190	MA_10066171g0010	shade>sun	putative laccase, a member of	LACCASE 10 (LAC10)	copper ion binding-GO:00(	58.89246819	0.928229755	0.29502098	3.146317766	0.001653403	0.019145461
PITA_000040998	High quality	AT5G35770	MA_82060g0010	shade>sun	A recessive mutation in the Ara	STERILE APETALA (SAP)	DNA-binding transcription	33.12163243	1.944785185	0.55175799	3.524706861	0.000423952	0.006123746
PITA_000041010	Low quality	AT5G14210	MA_10432546g0010	shade>sun	Leucine-rich repeat protein kinase family protein;(source:		ATP binding-GO:0005524,r	48.9081421	0.994679812	0.31284224	3.179493338	0.001475328	0.017452046
PITA_000041070	High quality	AT1G01060	MA_102199g0010	shade<sun	LHY encodes a myb-related pu	LATE ELONGATED HYPOC(	circadian rhythm-GO:0007	205.6168859	-0.644524635	0.15145538	-4.255541481	2.09E-05	0.000441972
PITA_000041139	Low quality	AT1G74910	MA_1324g0010	shade<sun	KONJAC1 is imilar to sugar pyro	KONJAC 1 (KJC1)	biosynthetic process-GO:0	143.1247791	-0.696444166	0.24879395	-2.799280938	0.005121656	0.046463364
PITA_000041147	High quality	AT1G69040	MA_10429375g0010	shade>sun	ACT-domain containing proteir	ACT DOMAIN REPEAT 4 (A	chloroplast-GO:0009507,c	35.42910012	2.514496089	0.69974259	3.593458693	0.000326317	0.004901563
PITA_000041326	High quality	AT1G77000	MA_107288g0010	shade>sun	AtSKP2;2 is a homolog of huma	(SKP2B)	heat acclimation-GO:0010	53.76516349	0.804522953	0.21877137	3.677459882	0.000235568	0.003692373
PITA_000041361	Low quality	AT1G01500	MA_9195168g0010	shade<sun	Erythronate-4-phosphate dehydrogenase family protein;(		phosphoglycerate dehydro	139.6670279	-1.0102676	0.1882639	-5.366231214	8.04E-08	2.74E-06
PITA_000041418	High quality	AT1G12480	MA_175194g0010	shade<sun	Encodes a membrane protein v	OZONE-SENSITIVE 1 (OZS1	anion transmembrane trar	307.5554784	-4.381962347	1.45527314	-3.011092705	0.002603094	0.027539598
PITA_000041425	Low quality	AT3G01480	MA_7732g0010	shade>sun	Encodes a chloroplast cyclophi	CYCLOPHILIN 38 (CYP38)	chloroplast-GO:0009507,c	127.3736337	10.28943727	1.44089858	7.140986471	9.27E-13	7.90E-11
PITA_000041456	High quality	AT3G11520	MA_10431608g0020	shade<sun	Encodes a B-type mitotic cyclin	CYCLIN B1;3 (CYCB1;3)	centrosome-GO:0005813,c	211.9578429	-1.131179226	0.38347142	-2.949839723	0.003179388	0.032280262
PITA_000041609	High quality	AT1G08380	MA_893g0010	shade<sun	Encodes subunit O of photosys	PHOTOSYSTEM I SUBUNIT	chloroplast-GO:0009507,e	594.577736	-0.724955254	0.14631199	-4.954858952	7.24E-07	2.08E-05
PITA_000041718	High quality	AT1G27620	MA_10392111g0010	shade<sun	HXXXD-type acyl-transferase family protein;(source:Arapo		nucleus-GO:0005634,trans	152.4280607	-10.88192005	3.90708965	-2.785172857	0.005349919	0.047930717
PITA_000041760	High quality	AT1G62850	MA_14958g0010	shade<sun	Class I peptide chain release factor;(source:Araport11)		mitochondrion-GO:00057:	278.763005	-1.169094113	0.37815677	-3.091559419	0.001991081	0.022397444
PITA_000041803	High quality	AT3G15850	MA_112563g0010	shade<sun	Chloroplastic enzyme responsil	FATTY ACID DESATURASE	1-18:1-2-16:0-monogalact	2019.441342	-1.851697161	0.25455527	-7.274244058	3.48E-13	3.15E-11
PITA_000041876	High quality	AT4G03250	MA_38676g0010	shade>sun	Homeodomain-like superfamily protein;(source:Araport11		DNA-binding transcription	29.70843935	0.847719837	0.27722718	3.057852522	0.002229293	0.024487264
PITA_000041959	High quality	AT2G19170	MA_9342539g0010	shade>sun	Encodes a novel subtilisin-like	SUBTILISIN-LIKE SERINE PI	extracellular region-GO:00	26.37656594	0.973398087	0.32468047	2.998018562	0.002717411	0.028591202
PITA_000041975	High quality	AT3G04120	MA_10426544g0010	shade<sun	encodes cytosolic GADPH (C su	GLYCERALDEHYDE-3-PHOS	apoplast-GO:0048046,chlo	383.8031633	-12.21406242	3.90689376	-3.126284761	0.0017703	0.02029408
PITA_000041996	High quality	AT3G18030	MA_329767g0010	shade<sun	flavin mononucleotide flavopro	HAL3-LIKE PROTEIN A (HA	coenzyme A biosynthetic p	105.6642161	-0.470410756	0.1696321	-2.773123451	0.005552105	0.049131262
PITA_000042083	High quality	AT5G17680	MA_164186g0010	shade>sun	disease resistance protein (TIR-NBS-LRR class);(source:Ara		ADP binding-GO:0043531,	48.47946927	1.278986879	0.28945868	4.418547294	9.94E-06	0.000228652
PITA_000042174	Low quality	AT1G04120	MA_281476g0010	shade<sun	encodes a high-affinity inositol	ATP-BINDING CASSETTE C	ABC-type xenobiotic trans	702.3893283	-3.782273968	0.56557806	-6.687448168	2.27E-11	1.42E-09
PITA_000042229	High quality	AT2G47000	MA_138894g0010	shade<sun	Encodes an auxin efflux transm	ATP-BINDING CASSETTE B	ABC-type xenobiotic trans	449.7066699	-1.733915747	0.22670739	-7.648254083	2.04E-14	2.24E-12
PITA_000042288	High quality	AT3G46440	MA_174294g0010	shade>sun	encodes a protein similar to UI	UDP-XYL SYNTHASE 5 (UX!	chloroplast-GO:0009507,c	29.60319035	1.639419853	0.43574289	3.762355985	0.00016832	0.002754728
PITA_000042290	High quality	AT5G05350	MA_10428976g0010	shade<sun	PLAC8 family protein;(source:Araport11)		cyclase activity-GO:000997	312.7916068	-0.825500003	0.28284643	-2.918544864	0.003516693	0.034835424
PITA_000042310	Low quality	AT4G01400	MA_10316984g0010	shade<sun	pentatricopeptide repeat-containing mitochondrial		Group II intron splicing-<br>GO:0000373,mitochondri<br>on-<br>GO:0005739,molecular_f<br>unction_unknown-<br>GO:0003674,cytosol-<br>GO:0005829,mitochondri<br>al respiratory chain<br>complex I assembly-<br>GO:0032981,mitochondri<br>al RNA processing-<br>GO:0000963	218.587616	-0.441031606	0.12809948	-3.442883728	0.000575547	0.007953582
PITA_000042336	Low quality	AT1G72100	MA_29426g0010	shade>sun	late embryogenesis abundant domain-containing protein		chloroplast-GO:0009507,p	32.58401617	0.915601889	0.28796887	3.179516876	0.001475208	0.017452046
PITA_000042420	High quality	AT4G03520	MA_474303g0010	shade<sun	Encodes a redox activated co-c	(ATHM2)	cell redox homeostasis-GC	440.267178	-0.850251451	0.1648452	-5.15787813	2.50E-07	7.72E-06
PITA_000042465	High quality	AT5G53730	MA_696598g0010	shade>sun	Late embryogenesis abundant	NDR1/HIN1-LIKE 26 (NHL2	anchored component of p	149.1379216	1.436315369	0.49368437	2.909379898	0.003621465	0.035663158
PITA_000042488	Low quality	AT4G34090	MA_184970g0010	shade<sun	cyclin delta-3;(source:Araport1	PROTEIN IN CHLOROPLAST	chloroplast-GO:0009507,c	465.043516	-1.187111345	0.24478291	-4.849649599	1.24E-06	3.43E-05
PITA_000042654	Low quality	AT2G28160	MA_833677g0010	shade>sun	Encodes a putative transcriptic	FER-LIKE IRON DEFICIENCY	DNA-binding transcription	56.05281503	1.11073762	0.33722804	3.29372853	0.000988679	0.012510608
PITA_000042658	High quality	AT5G11310	MA_10434663g0010	shade<sun	The SOAR1 gene encodes a pe	SUPPRESSOR OF THE ABA	cytosol-GO:0005829,mitoc	198.059453	-0.849441899	0.29471655	-2.882233469	0.00394867	0.038184612
PITA_000042720	High quality	AT3G06060	MA_172106g0010	shade<sun	Encodes one of the Arabidopsi	TSC10A (TSC10A)	3-dehydrosphinganine red	141.4848195	-0.57778857	0.19159183	-3.015726581	0.002563643	0.027234659
PITA_000042741	High quality	AT4G01800	MA_8475350g0010	shade<sun	Encodes the ATPase subunit of	ALBINO OR GLASSY YELLO	ABC-type protein transpor	341.1035365	-1.271834843	0.18896501	-6.730530852	1.69E-11	1.10E-09
PITA_000042750	High quality	AT5G43810	MA_315811g0010	shade<sun	Encodes Argonaute10, a meml	ARGONAUTE 10 (AGO10)	cytoplasm-GO:0005737,de	212.9235091	-2.080881134	0.68482257	-3.038569733	0.002377041	0.025696022
PITA_000042827	Low quality	AT1G64980	MA_10436855g0020	shade<sun	Encodes a putative nucleotide-	CADMIUM 2+ INDUCED (C	chloroplast-GO:0009507,c	133.8635972	-0.954784005	0.15555049	-6.13809701	8.35E-10	3.89E-08
PITA_000042842	High quality	AT3G27200	MA_76825g0010	shade<sun	Cupredoxin superfamily protein;(source:Araport11)		anchored component of m	139.1321252	-1.037910906	0.20824574	-4.984067828	6.23E-07	1.81E-05
PITA_000042890	High quality	AT2G46160	MA_9764263g0010	shade>sun	RING/U-box superfamily protei	ARABIDOPSIS T??XICOS EI	chloroplast-GO:0009507,p	100.1141286	2.019727939	0.36135221	5.589361016	2.28E-08	8.51E-07
PITA_000042898	Low quality	AT1G34000	MA_17658g0010	shade<sun	Encodes a novel member of th	ONE-HELIX PROTEIN 2 (OH	chloroplast-GO:0009507,c	281.406875	-0.749304811	0.13052577	-5.740665743	9.43E-09	3.70E-07
PITA_000042976	High quality	AT2G18420	MA_23745g0010	shade>sun	Encodes a Gibberellin-regulated	GASA/GAST/Snakin famil	extracellular region-GO:00	37.06372436	1.590751274	0.48546183	3.276779288	0.001049984	0.013135218
PITA_000042991	High quality	AT3G28910	MA_355131g0010	shade>sun	Encodes a MYB family transcrip	MYB DOMAIN PROTEIN 3(	DNA-binding transcription	28.87052412	1.565186769	0.4241668	3.690026615	0.000224231	0.003532227
PITA_000043170	Low quality	AT5G01880	MA_41514g0010	shade>sun	RING/U-box superfamily protei	DAF-LIKE GENE 2 (DAFL2)	protein ubiquitination-GO	72.24152928	1.490672729	0.4288716	3.475801893	0.000509328	0.007184151
PITA_000043338	High quality	AT1G32780	MA_173093g0010	shade<sun	GroES-like zinc-binding dehydrogenase family protein;(sou		alcohol dehydrogenase ac	110.7137715	-0.474550643	0.17125958	-2.770943607	0.005589411	0.049392059
PITA_000043567	High quality	AT5G46220	MA_33920g0010	shade>sun	Encodes a protein with alkaline	TURGOR REGULATION DEI	chloroplast-GO:0009507,d	326.0147302	11.64481401	3.90687918	2.980592299	0.002876915	0.029944615
PITA_000043678	High quality	AT3G51990	MA_9461995g0010	shade<sun	Protein kinase superfamily protein;(source:Araport11)		phosphorylation-GO:0016:	126.6748445	-0.504258483	0.17101139	-2.948683688	0.003191304	0.032362161
PITA_000043744	High quality	AT1G22160	MA_6300g0010	shade<sun	senescence-associated family protein (DUF581);(source:Ar		cytosol-GO:0005829,kinas	213.4300213	-0.808463116	0.15474844	-5.224369974	1.75E-07	5.51E-06
PITA_000043841	High quality	AT3G07880	MA_10430494g0010	shade<sun	RhoGTPase GDP dissociation ir	SUPERCENTIPEDE1 (SCN1)	cell tip growth-GO:000993	324.9275943	-1.402540616	0.25514207	-5.49709656	3.86E-08	1.40E-06
PITA_000043911	Low quality	AT4G24130	MA_4522g0020	shade>sun	DUF538 family protein (Protein of unknown function, DUF		biological_process_unknov	84.23276481	1.852756496	0.59360088	3.121215899	0.001801059	0.020571883
PITA_000043927	High quality	AT1G70300	MA_103581g0010	shade<sun	potassium transporter	K+ UPTAKE PERMEASE 6 (	membrane-GO:0016020,m	95.934325	-0.762496375	0.18621015	-4.09481647	4.23E-05	0.000821552
PITA_000043931	High quality	AT5G39530	MA_96307g0010	shade>sun	hypothetical protein (DUF1997);(source:Araport11)		biological_process_unknov	74.77749993	9.520139889	1.3563477	7.018952356	2.24E-12	1.71E-10
PITA_000044046	High quality	AT1G28440	MA_10426680g0010	shade>sun	HAESA-like 1;(source:Araport1	HAESA-LIKE 1 (HSL1)	kinase activity-GO:001630	34.11229582	0.801402752	0.28514362	2.810523192	0.004946102	0.045442879
PITA_000044050	High quality	AT2G27610	MA_252238g0010	shade<sun	Tetratricopeptide repeat (TPR)-like superfamily protein;(s		intracellular membrane-bo	148.2874113	-2.03301776	0.47116631	-4.314862287	1.60E-05	0.000347215
PITA_000044056	High quality	AT1G32990	MA_26087g0010	shade<sun	mutant has Decreased effective	PLASTID RIBOSOMAL PRO	chloroplast-GO:0009507,c	683.9408721	-0.795938826	0.26063752	-3.053815191	0.002259512	0.024765276
PITA_000044086	Low quality	AT5G48485	MA_106330g0010	shade<sun	Encodes a putative apoplastic l	DEFECTIVE IN INDUCED RI	endoplasmic reticulum-GC	470.9447224	-1.272228557	0.16830895	-7.558888231	4.07E-14	4.29E-12
PITA_000044207	High quality	AT1G26830	MA_507769g0010	shade<sun	Cullin, putative, similar to Culli	CULLIN 3 (CUL3)	cullin-RING ubiquitin ligase	607.0894124	-0.842662288	0.12513915	-6.733802468	1.65E-11	1.08E-09
PITA_000044269	High quality	AT1G33475	MA_40583g0020	shade<sun	SNARE-like superfamily protein;(source:Araport11)		nucleus-GO:0005634	115.72547	-0.493227278	0.17126583	-2.879893115	0.0039781	0.038410293
PITA_000044382	High quality	AT5G02070	MA_418666g0010	shade>sun	Protein kinase family protein;(source:Araport11)		cell surface receptor signa	23.93729119	3.040201311	0.57922289	5.248758896	1.53E-07	4.94E-06
PITA_000044433	High quality	AT4G25400	MA_384190g0010	shade<sun	basic helix-loop-helix (bHLH) DNA-binding superfamily pro		DNA-binding transcription	183.3210843	-1.286135554	0.31270674	-4.112912824	3.91E-05	0.000767399

PITA_000044624	High quality	AT1G63220	MA_108928g0010	shade>sun	Calcium-dependent lipid-binding (CaLB domain) family pro		cytoplasm-GO:0005737	37.76174712	1.491945078	0.50304981	2.965799922	0.003018968	0.030988351
PITA_000044773	High quality	AT3G19380	MA_83739g0020	shade>sun	PUB25 and PUB26 are closely r	PLANT U-BOX 25 (PUB25)	defense response to other	40.73277844	1.023863858	0.30056508	3.40646312	0.000658104	0.008889873
PITA_000044810	Low quality	AT5G36970	MA_114716g0010	shade>sun	NDR1/HIN1-like protein, expre	NDR1/HIN1-LIKE 25 (NHL2	defense response to other	66.21472434	0.793042349	0.21760082	3.644482288	0.000267931	0.004142986
PITA_000044814	High quality	AT5G54160	MA_630428g0010	shade<sun	A caffeic acid/5-hydroxyferulic	O-METHYLTRANSFERASE 1	acetylserotonin O-methylt	152.4280607	-10.88192005	3.90708965	-2.785172857	0.005349919	0.047930717
PITA_000045073	Low quality	AT4G37870	MA_101646g0010	shade>sun	Encodes a phosphoenolpyruva	PHOSPHOENOLPYRUVATE	cellular response to phosp	38.30913325	1.340625091	0.42705499	3.139232901	0.001693908	0.019541131
PITA_000045116	Low quality	AT5G64260	MA_10425915g0010	shade>sun	EXORDIUM like 2;(source:Arap	EXORDIUM LIKE 2 (EXL2)	cell wall-GO:0005618,cellu	26.46943655	1.567105209	0.3883789	4.034990597	5.46E-05	0.001026939
PITA_000045149	High quality	AT2G41510	MA_17212g0010	shade<sun	It encodes a protein whose sec	CYTOKININ OXIDASE/DEH	cytokinin catabolic process	320.2369803	-0.419028563	0.12912934	-3.245029762	0.001174383	0.014419645
PITA_000045482	High quality	AT5G54130	MA_10437257g0040	shade<sun	Calcium-binding endonuclease/exonuclease/phosphatase		3'-5'-exoribonuclease activ	553.3495782	-1.460401005	0.17738764	-8.232822869	1.83E-16	3.03E-14
PITA_000045571	Low quality	AT2G38150	MA_10436829g0020	shade>sun	alpha 1,4-glycosyltransferase family protein;(source:Arapo		Golgi apparatus-GO:00057	23.11085802	2.191592267	0.41460364	5.285993813	1.25E-07	4.09E-06
PITA_000045591	High quality	AT3G57560	MA_623831g0010	shade<sun	encodes a N-acetylglutamate k	N-ACETYL-L-GLUTAMATE K	acetylglutamate kinase act	158.3184131	-0.773889716	0.18446392	-4.195344522	2.72E-05	0.000561845
PITA_000045597	Low quality	AT5G04820	MA_61037g0010	shade>sun	ovate family protein 13;(source	OVATE FAMILY PROTEIN 1	negative regulation of tran	48.28374868	1.190470359	0.27985528	4.253878555	2.10E-05	0.000444522
PITA_000045680	High quality	AT2G25140	MA_763g0010	shade<sun	Encodes ClpB4, which belongs	CASEIN LYTIC PROTEINASE	ATPase activity-GO:00168	182.9524647	-0.505523177	0.14199757	-3.560083379	0.000370737	0.005474232
PITA_000045696	High quality	AT1G60710	MA_48532g0010	shade>sun	Encodes ATB2.	(ATB2)	cytoplasm-GO:0005737,cy	52.23882448	9.002933621	1.56097014	5.76752457	8.04E-09	3.21E-07
PITA_000045788	High quality	AT2G43030	MA_10432831g0010	shade<sun	Ribosomal protein L3 family pr	PLASTID RIBOSOMAL PRO	chloroplast-GO:0009507,c	317.776263	-0.724356465	0.10228889	-7.081477362	1.43E-12	1.16E-10
PITA_000045818	Low quality	AT2G34730	MA_36342g0010	shade<sun	myosin heavy chain-like protein;(source:Araport11)		mitochondrion-GO:00057	201.0825876	-0.870500642	0.19759552	-4.405467484	1.06E-05	0.000241791
PITA_000045882	Low quality	AT1G80870	MA_17059g0010	shade>sun	Protein kinase superfamily protein;(source:Araport11)		chloroplast-GO:0009507,p	42.51786679	0.782310601	0.23947136	3.266823267	0.001087616	0.013538827
PITA_000045996	High quality	AT1G28590	MA_60155g0010	shade<sun	GDSL-motif esterase/acyltransferase/lipase. Enzyme grou		extracellular region-GO:00	276.7510867	-1.976372635	0.27778152	-7.114845565	1.12E-12	9.33E-11
PITA_000046189	High quality	AT2G28380	MA_15142g0010	shade>sun	Encodes a cytoplasmic dsRNA-l	DSRNA-BINDING PROTEIN	cytoplasm-GO:0005737,dc	81.5466361	0.58779268	0.20123741	2.920891627	0.003490312	0.034711449
PITA_000046222	High quality	AT1G30440	MA_10430653g0010	shade<sun	Phototropic-responsive NPH3 family protein;(source:Arap		nucleus-GO:0005634,plasr	254.1566477	-0.764325496	0.27254883	-2.804361665	0.005041632	0.045968891
PITA_000046256	High quality	AT5G49940	MA_10436193g0010	shade<sun	Encodes a protein containing t	NIFU-LIKE PROTEIN 2 (NFU	4 iron, 4 sulfur cluster binc	123.2166231	-0.906426582	0.25564522	-3.545642583	0.000391657	0.005729461
PITA_000046290	High quality	AT1G48900	MA_10429875g0010	shade<sun	Signal recognition particle, SRP54 subunit protein;(source		7S RNA binding-GO:00083	287.0822694	-1.531961727	0.15312152	-10.00487528	1.45E-23	5.38E-21
PITA_000046293	High quality	AT5G60530	MA_182721g0010	shade<sun	late embryogenesis abundant protein-related / LEA prote		cytoplasm-GO:0005737,m	426.1612323	-1.312642184	0.12253616	-10.71228389	8.91E-27	4.50E-24
PITA_000046316	High quality	AT5G15710	MA_10371740g0010	shade>sun	Galactose oxidase/kelch repeat superfamily protein;(sour		nucleus-GO:0005634,prote	35.23000454	0.89929716	0.30406903	2.957542736	0.003101018	0.031681875
PITA_000046329	High quality	AT5G06570	MA_5588g0010	shade>sun	alpha/beta-Hydrolases superfamily protein;(source:Arapo		hydrolase activity-GO:001	34.92983806	0.949516711	0.2946638	3.222373175	0.001271334	0.01544463
PITA_000046386	Low quality	AT4G30780	MA_132750g0010	shade>sun	ATP-dependent DNA helicase;(source:Araport11)		biological_process_unknow	44.3485039	0.772398176	0.23607745	3.271799855	0.001068652	0.013335677
PITA_000046397	High quality	AT5G57040	MA_10432583g0010	shade<sun	Vicinal oxygen chelate (VOC) su	GLYOXALASE I-LIKE;11 (GL	chloroplast-GO:0009507,re	159.5626641	-0.600125088	0.18498782	-3.244133011	0.001178087	0.014458082
PITA_000046415	Low quality	AT3G52790	MA_106046g0010	shade>sun	peptidoglycan-binding LysM domain-containing protein;(s		molecular_function_unkno	32.73020823	1.079575559	0.33864153	3.187959753	0.001432805	0.017077191
PITA_000046446	High quality	AT2G38600	MA_10430401g0020	shade<sun	HAD superfamily, subfamily IIIB acid phosphatase;(source		acid phosphatase activity-	171.0695229	-1.635111521	0.58302755	-2.804518469	0.00503918	0.045968891
PITA_000046518	Low quality	AT1G61190	MA_10430184g0010	shade<sun	Putative CC-NB-LRR resistance gene. It's ortholog in ecoty		ADP binding-GO:0043531,	55.78952798	-9.428251272	1.21224637	-7.777504251	7.40E-15	8.88E-13
PITA_000046543	High quality	AT5G25752	MA_116382g0010	shade<sun	Chloroplast-localized rhomboic	RHOMBOID-LIKE PROTEIN	chloroplast-GO:0009507,c	146.6070251	-0.863678991	0.14597425	-5.916653223	3.29E-09	1.39E-07
PITA_000046577	High quality	AT1G75690	MA_122077g0010	shade<sun	Thylakoid Thiol/Disulfide-Modi	LOW QUANTUM YIELD OF	chloroplast-GO:0009507,c	562.5643628	-0.868549209	0.12810499	-6.779979758	1.20E-11	8.06E-10
PITA_000046689	Low quality	AT1G32080	MA_185619g0010	shade<sun	Encodes a plant LrgAB/CidAB p	(LrgB)	cellular response to water	419.6344661	-0.645408343	0.22703145	-2.842814653	0.004471707	0.04203371
PITA_000046724	High quality	AT5G56300	MA_128083g0020	shade>sun	A member of the Arabidopsis S	GIBBERELLIC ACID METHY	cellular_component_unkn	67.95864745	1.971286271	0.35938695	5.485135933	4.13E-08	1.48E-06
PITA_000046733	Low quality	AT1G45170	MA_69201g0010	shade<sun	outer envelope pore 24B-like protein;(source:Araport11)		etioplast membrane-GO:0	119.6232443	-0.587816631	0.17759311	-3.309906705	0.000933271	0.011917513
PITA_000046819	High quality	AT1G69870	MA_408691g0010	shade>sun	Encodes a low affinity nitrate t	NRT1/ PTR FAMILY 2.13 (N	chloroplast-GO:0009507,lc	173.1986121	2.510301148	0.32340502	7.762097148	8.35E-15	9.89E-13
PITA_000046841	High quality	AT5G11950	MA_47673g0010	shade>sun	Encodes a protein of unknown	LONELY GUY 8 (LOG8)	cytokinin biosynthetic proc	39.94206648	0.768196351	0.24121981	3.184632063	0.001449382	0.017250309
PITA_000046905	High quality	AT5G65270	MA_10428137g0010	shade<sun	RAB GTPase homolog A4A;(sou	RAB GTPASE HOMOLOG A	cell wall biogenesis-GO:00	98.9531317	-0.554612713	0.19602992	-2.829224824	0.004666091	0.043423914
PITA_000046946	High quality	AT4G28780	MA_15196g0010	shade>sun	GDSL-motif esterase/acyltransferase/lipase. Enzyme grou		chloroplast-GO:0009507,h	114.7520609	0.671558913	0.18373645	3.655011873	0.00025717	0.003996198
PITA_000047059	High quality	AT5G43290	MA_10434450g0010	shade>sun	member of WRKY Transcriptio	WRKY DNA-BINDING PROT	DNA-binding transcription	96.24647632	0.79831627	0.27293242	2.924959524	0.00344501	0.03439555
PITA_000047083	High quality	AT3G23920	MA_10432630g0020	shade<sun	Encodes a chloroplast beta-am	BETA-AMYLASE 1 (BAM1)	amylopectin maltohydrola	289.6732837	-1.408197038	0.29604899	-4.756635209	1.97E-06	5.23E-05
PITA_000047097	Low quality	AT1G56580	MA_920567g0010	shade>sun	Encodes SMALLER WITH VARIA	SMALLER WITH VARIABLE	cytoplasm-GO:0005737,tri	108.1697517	1.20740072	0.18179372	6.641597382	3.10E-11	1.89E-09
PITA_000047120	High quality	AT2G19870	MA_123340g0010	shade<sun	tRNA/rRNA methyltransferase (SpoU) family protein;(sour		chloroplast-GO:0009507,m	244.8979908	-0.849845557	0.25636036	-3.315042745	0.000916291	0.011733303
PITA_000047186	High quality	AT1G12280	MA_10435336g0010	shade>sun	Encodes a NB-LRR protein SUM	SUPPRESSOR OF MKK1 MI	ADP binding-GO:0043531,	19.93426631	2.617757414	0.60878915	4.299940972	1.71E-05	0.000369526
PITA_000047239	Low quality	AT4G34740	MA_70676g0010	shade<sun	Encodes glutamine 5-phosphoi	GLN PHOSPHORIBOSYL PY	amidophosphoribosyltrans	413.9103848	-1.658907346	0.38547637	-4.303525345	1.68E-05	0.000363908
PITA_000047325	Low quality	AT1G07990	MA_10435320g0020	shade>sun	SIT4 phosphatase-associated family protein;(source:Arapo		chloroplast-GO:0009507,c	58.45926452	1.665594461	0.4130778	4.032156764	5.53E-05	0.001036313
PITA_000047332	High quality	AT3G25250	MA_55799g0010	shade<sun	Arabidopsis protein kinase The	AGC2 KINASE 1 (AGC2-1)	cellular response to hypox	235.176918	-1.193544747	0.27216516	-4.385369359	1.16E-05	0.000263318
PITA_000047411	High quality	AT5G50920	MA_10427039g0010	shade<sun	Encodes a protein that is simila	CLPC HOMOLOGUE 1 (CLP	ATP-dependent peptidase	208.5742611	-0.955458661	0.12278097	-7.781814073	7.15E-15	8.63E-13
PITA_000047476	High quality	AT4G02280	MA_113423g0010	shade>sun	Encodes a protein with sucrose	SUCROSE SYNTHASE 3 (SU	chloroplast-GO:0009507,re	318.9418066	11.61326056	1.65292424	7.025887999	2.13E-12	1.65E-10
PITA_000047481	Low quality	AT3G10870	MA_10772g0010	shade<sun	Encodes a methyl IAA esterase	METHYL ESTERASE 17 (ME	hydrolase activity, acting c	266.020455	-0.989537722	0.12164556	-8.134598147	4.13E-16	6.33E-14
PITA_000047526	High quality	AT5G08250	MA_215531g0010	shade<sun	Cytochrome P450 superfamily protein;(source:Araport11)		cellular_component_unkn	183.1759723	-11.14692096	3.90703512	-2.853038328	0.00433034	0.040979801
PITA_000047545	High quality	AT4G32850	MA_136671g0010	shade>sun	Encodes a nuclear poly(A) poly	NUCLEAR POLY(A) POLYM	mRNA polyadenylation-GC	187.6372316	10.84782526	3.90696762	2.776533191	0.005494203	0.048755731
PITA_000047558	High quality	AT5G23140	MA_207420g0010	shade<sun	One of several nuclear-encode	NUCLEAR-ENCODED CLP P	ATPase binding-GO:00511	302.8012671	-0.88737713	0.11428283	-7.764745806	8.18E-15	9.73E-13
PITA_000047565	Low quality	AT4G24310	MA_461971g0010	shade>sun	transmembrane protein, putat	DUF679 DOMAIN MEMBR	endomembrane system or	203.657389	0.942499727	0.29817196	3.160926776	0.00157268	0.018482291
PITA_000047621	Low quality	AT5G26600	MA_498229g0010	shade<sun	Pyridoxal phosphate (PLP)-dependent transferases superf		chloroplast-GO:0009507	142.3730075	-0.507484766	0.17514629	-2.897490767	0.003761608	0.036813254
PITA_000047727	High quality	AT1G13130	MA_10432675g0010	shade<sun	Cellulase (glycosyl hydrolase family 5) protein;(source:Ara		chloroplast-GO:0009507,m	96.52719664	-2.121911343	0.60833507	-3.488063502	0.000486533	0.006893455
PITA_000047730	High quality	AT3G09140	MA_58518g0010	shade>sun	hypothetical protein (DUF674);(source:Araport11)		biological_process_unknow	36.70028663	2.404258125	0.62896319	3.822573688	0.000132066	0.002221951
PITA_000047749	High quality	AT4G22240	MA_28715g0010	shade<sun	Involved in photoprotection of	FIBRILLIN 1B (FBN1B)	biological_process_unknow	257.8311355	-0.623900689	0.13625162	-4.579033339	4.67E-06	0.000116298
PITA_000047765	High quality	AT5G18670	MA_21789g0010	shade>sun	putative beta-amylase BMY3 (E	BETA-AMYLASE 3 (BMY3)	beta-amylase activity-GO:(	49.25782856	0.820157039	0.27807551	2.949404099	0.003183874	0.032309531
PITA_000047798	High quality	AT5G58440	MA_42929g0010	shade<sun	sorting nexin 2A;(source:Arapc	SORTING NEXIN 2A (SNX2	cytosol-GO:0005829,devel	206.1288779	-1.823113513	0.18542019	-9.832335614	8.17E-23	2.75E-20
PITA_000047808	High quality	AT4G19390	MA_34822g0010	shade<sun	Uncharacterized protein family (UPF0114);(source:Arapor		biological_process_unknow	332.3358714	-0.82301763	0.13693632	-6.010221542	1.85E-09	8.23E-08
PITA_000047812	Low quality	AT5G16150	MA_10434103g0020	shade<sun	Encodes a putative plastidic glu	PLASTIDIC GLC TRANSLOC	carbohydrate transmembr	173.6740258	-1.892838386	0.47922055	-3.949827277	7.82E-05	0.001405842
PITA_000047860	High quality	AT2G02040	MA_10431577g0010	shade>sun	Encodes a di- and tri-peptide tr	NRT1/ PTR FAMILY 8.3 (NF	dipeptide transport-GO:00	15.73793032	1.743220109	0.44146386	3.948726675	7.86E-05	0.00141031
PITA_000047880	Low quality	AT5G23730	MA_23011g0010	shade<sun	Encodes REPRESSOR OF UV-B I	REPRESSOR OF UV-B PHO	chloroplast organization-G	718.8445906	-1.032298377	0.21888521	-4.716163156	2.40E-06	6.29E-05
PITA_000048002	High quality	AT3G07470	MA_10434011g0010	shade<sun	transmembrane protein, putative (Protein of unknown fu		biological_process_unknow	796.1934767	-1.963390132	0.31306517	-6.2715061	3.58E-10	1.79E-08
PITA_000048053	Low quality	AT3G09650	MA_11675g0010	shade<sun	RNA binding protein involved i	HIGH CHLOROPHYLL FLUO	chloroplast-GO:0009507,c	308.5865264	-0.954295258	0.15944017	-5.985287666	2.16E-09	9.46E-08
PITA_000048063	Low quality	AT3G25700	MA_122699g0020	shade<sun	Eukaryotic aspartyl protease family protein;(source:Arapo		aspartic-type endopeptida	242.8714494	-1.271756753	0.14676572	-8.665216636	4.51E-18	9.32E-16
PITA_000048102	High quality	AT5G18670	MA_21789g0010	shade<sun	putative beta-amylase BMY3 (E	BETA-AMYLASE 3 (BMY3)	beta-amylase activity-GO:(	812.47553	-1.496915894	0.30158142	-4.963554703	6.92E-07	2.00E-05
PITA_000048124	High quality	AT2G41250	MA_16075g0010	shade<sun	Haloacid dehalogenase-like hydrolase (HAD) superfamily p		oxoacid metabolic process	92.65766263	-0.528833162	0.17115769	-3.089742264	0.002003303	0.022484775
PITA_000048165	High quality	AT3G03760	MA_962403g0010	shade>sun	LOB domain-containing protei	LOB DOMAIN-CONTAININ	hormone-mediated signali	53.21301193	2.019732604	0.41503657	4.866396698	1.14E-06	3.17E-05
PITA_000048290	High quality	AT1G73340	MA_266173g0010	shade<sun	ADTO1 is required for the activ	ABIETANE DITERPENE OXI	brassinosteroid biosynthe	150.2417271	-0.630287821	0.17546442	-3.592111785	0.000328009	0.004924041
PITA_000048396	Low quality	AT1G07160	MA_10429333g0010	shade>sun	Protein phosphatase 2C family protein;(source:Araport11)		chloroplast-GO:0009507,m	4.111041224	5.33483985	1.55046419	3.440801719	0.000579993	0.008006258
PITA_000048700	High quality	AT4G13660	MA_43637g0010	shade<sun	Encodes a pinoresinol reducta	PINORESINOL REDUCTASE	cytoplasm-GO:0005737,lig	164.2691538	-2.139932103	0.69313589	-3.087319737	0.002019702	0.022648684
PITA_000048731	Low quality	AT4G35800	MA_8544g0010	shade<sun	Encodes the unique largest sub	RNA POLYMERASE II LARG	DNA binding-GO:0003677,	126.7976786	-0.59281681	0.15426842	-3.842761929	0.000121657	0.002066129
PITA_000048812	High quality	AT3G09930	MA_5971026g0020	shade>sun	GDSL-motif esterase/acyltransferase/lipase. Enzyme grou		hydrolase activity, acting c	61.39453933	1.267271253	0.27974705	4.530061154	5.90E-06	0.00014272
PITA_000048869	High quality	AT5G09430	MA_182752g0010	shade<sun	alpha/beta-Hydrolases superfamily protein;(source:Arapo		cytoplasm-GO:0005737	209.7905796	-1.082146542	0.31769696	-3.406222555	0.000658685	0.008892947
PITA_000048882	High quality	AT3G54950	MA_10431235g0020	shade<sun	Encodes pPLAIIIbeta, a membe	PATATIN-RELATED PHOSP	acyl-CoA hydrolase activity	417.6589363	-12.33604806	3.9068833	-3.157516397	0.001591193	0.018639055
PITA_000048921	High quality	AT4G10180	MA_10292334g0010	shade<sun	Encodes a nuclear-localized pro	DE-ETIOLATED 1 (DET1)	carbohydrate metabolic pr	128.0347101	-0.929884749	0.24630384	-3.77535635	0.000159779	0.002633744
PITA_000048925	Low quality	AT5G17870	MA_232471g0010	shade<sun	plastid-specific ribosomal prote	PLASTID-SPECIFIC 50S RIB	chloroplast-GO:0009507,m	488.2998336	-1.025710063	0.1186434	-8.645319444	5.37E-18	1.10E-15
PITA_000048928	High quality	AT5G56150	MA_612949g0010	shade>sun	ubiquitin-conjugating enzyme :	UBIQUITIN-CONJUGATING	nucleus-GO:0005634,prote	54.69071342	0.986343822	0.22496758	4.384382128	1.16E-05	0.000264277
PITA_000048951	High quality	AT2G40780	MA_17139g0010	shade<sun	Nucleic acid-binding, OB-fold-like protein;(source:Araport		nucleus-GO:0005634,trans	112.4526691	-0.520358386	0.17200537	-3.025245012	0.002484318	0.026650147
PITA_000049050	High quality	AT1G68400	MA_136282g0010	shade>sun	leucine-rich repeat transmembrane protein kinase family		kinase activity-GO:001630	34.57572928	1.117127285	0.3320265	3.364572683	0.000766623	0.010096203
PITA_000049063	High quality	AT4G01310	MA_8012412g0010	shade<sun	Ribosomal L5P family protein;(	PLASTID RIBOSOMAL PRO	chloroplast-GO:0009507,c	533.8495105	-0.896772527	0.13043326	-6.875336372	6.18E-12	4.37E-10
PITA_000049074	High quality	AT5G14660	MA_10434550g0010	shade<sun	Encodes a peptide deformylase	PEPTIDE DEFORMYLASE 1	chloroplast-GO:0009507,m	120.3197642	-0.796270513	0.1972013	-4.037856256	5.39E-05	0.001019205
PITA_000049143	High quality	AT5G22920	MA_10436725g0010	shade>sun	Encodes a protein with sequen	RING ZINC-FINGER PROTEI	nucleus-GO:0005634,prote	52.98241772	0.602822828	0.21745837	2.77212986	0.005569081	0.049246927
PITA_000049150	High quality	AT4G02450	MA_74917g0010	shade<sun	Encodes one of two isoforms o	(P23-1)	chaperone binding-GO:00	252.0240825	-11.60732078	3.90696124	-2.970933181	0.002968964	0.03066227
PITA_000049159	High quality	AT3G50750	MA_195595g0010	shade<sun	BES1/BZR1 homolog 1;(source:	BES1/BZR1 HOMOLOG 1 (I	brassinosteroid mediated	131.6108912	-0.548522295	0.15823907	-3.466415134	0.000527448	0.007398356
PITA_000049161	Low quality	AT2G17525	MA_17644g0010	shade<sun	Pentatricopeptide repeat (PPR) superfamily protein;(sour		biological_process_unknow	229.3729616	-1.575858961	0.44907627	-3.509112059	0.000449605	0.006446304
PITA_000049280	High quality	AT1G09740	MA_100052g0010	shade<sun	Adenine nucleotide alpha hydrolases-like superfamily pro		cytoplasm-GO:0005737	377.6804289	-1.435655646	0.31595272	-4.543893905	5.52E-06	0.000134827
PITA_000049308	High quality	AT1G14850	MA_8686909g0010	shade>sun	Encodes a protein similar to nu	NUCLEOPORIN 155 (NUP1	nuclear pore-GO:0005643,	67.31354822	0.610880033	0.19769366	3.090033441	0.00200134	0.022484775
PITA_000049383	High quality	AT1G27340	MA_10436145g0010	shade<sun	Encodes a putative F-box prote	LEAF CURLING RESPONSIV	leaf vascular tissue patterr	111.2552172	-0.760030428	0.25219458	-3.013666818	0.002581111	0.027374104
PITA_000049396	Low quality	AT3G57430	MA_96662g0010	shade<sun	Encodes a chloroplast RNA edi	ORGANELLE TRANSCRIPT I	chloroplast-GO:0009507,c	142.8663556	-1.213980341	0.30549766	-3.973779504	7.07E-05	0.001286294
PITA_000049419	High quality	AT3G22370	MA_10430050g0010	shade>sun	Encodes AOX1a, an isoform of	ALTERNATIVE OXIDASE 1A	alternative oxidase activity	18.04593334	1.628630451	0.41401999	3.933700014	8.36E-05	0.001494067
PITA_000049461	Low quality	AT2G15890	MA_123309g0010	shade>sun	Encodes CBP1, a regulator of t	MATERNAL EFFECT EMBRY	cellular response to hypox	11.83611053	2.138497371	0.56279229	3.799798628	0.000144814	0.002418677
PITA_000049509	High quality	AT5G62890	MA_76962g0010	shade>sun	Xanthine/uracil permease family protein;(source:Araport1		cell wall-GO:0005618,endc	28.00912121	8.102519869	1.21562598	6.66530658	2.64E-11	1.64E-09
PITA_000049548	High quality	AT4G10250	MA_10243352g0010	shade>sun	Columbia endomembrane-loca	(ATHSP22.0)	cellular response to hypox	30.70470313	1.076304356	0.28089057	3.831756815	0.000127231	0.002150655
PITA_000049588	High quality	AT5G03795	MA_61350g0010	shade<sun	Exostosin family protein;(source:Araport11)		nucleus-GO:0005634,prote	317.1960493	-11.93910615	3.90692086	-3.055886357	0.002243963	0.024637677
PITA_000049591	High quality	AT5G54180	MA_926365g0010	shade<sun	Encodes a member of the Mitc	PLASTID TRANSCRIPTIONA	chloroplast-GO:0009507,c	274.1163995	-0.883534432	0.17254342	-5.120649744	3.04E-07	9.30E-06
PITA_000049655	Low quality	AT4G25130	MA_10430139g0010	shade<sun	Encodes a chloroplast-localized	PEPTIDE MET SULFOXIDE	cellular response to oxidat	224.0252447	-0.874813816	0.22610159	-3.869118407	0.00010923	0.00188296
PITA_000049661	Low quality	AT2G26280	MA_10429553g0010	shade>sun	smr (Small MutS Related) dom	CTC-INTERACTING DOMAI	chloroplast-GO:0009507,m	28.9956877	1.819978396	0.49187603	3.700075426	0.000215535	0.003408027
PITA_000049753	High quality	AT1G03080	MA_102054g0010	shade>sun	kinase interacting (KIP1-like) fa	NETWORKED 1D (NET1D)	actin filament binding-GO:	44.19103796	0.843934635	0.26355461	3.202124362	0.001364181	0.016390968
PITA_000049978	Low quality	AT5G23120	MA_10433328g0010	shade<sun	encodes a stability and/or asse	HIGH CHLOROPHYLL FLUO	chloroplast-GO:0009507,c	189.5314575	-0.99398425	0.13379722	-7.429035159	1.09E-13	1.08E-11
PITA_000050009	Low quality	AT5G46160	MA_106890g0010	shade<sun	Ribosomal protein L14p/L23e family protein;(source:Arap		chloroplast-GO:0009507,la	111.676418	-0.779227063	0.19253644	-4.047166616	5.18E-05	0.000987491
PITA_000050055	High quality	AT1G26560	MA_48585g0010	shade<sun	beta glucosidase 40;(source:Ar	BETA GLUCOSIDASE 40 (B	apoplast-GO:0048046,beta	320.7497166	-1.319791561	0.37425759	-3.526425655	0.000421209	0.006087622
PITA_000050098	High quality	AT5G63960	MA_36703g0010	shade<sun	Encodes the catalytic subunits	GIGANTEA SUPPRESSOR5	3'-5'-exodeoxyribonucleas	243.8628136	-0.662548538	0.18552306	-3.571246327	0.000355287	0.005283212
PITA_000050116	High quality	AT5G17230	MA_86494g0010	shade<sun	Encodes phytoene synthase th	PHYTOENE SYNTHASE (PS	carotenoid biosynthetic pr	605.5051919	-1.022043714	0.11491226	-8.894122423	5.89E-19	1.34E-16
PITA_000050156	High quality	AT5G67500	MA_28816g0010	shade<sun	Encodes a voltage-dependent a	VOLTAGE DEPENDENT AN	anion transmembrane tran	279.6893998	-1.032679326	0.25550809	-4.041669803	5.31E-05	0.00100486
PITA_000050216	High quality	AT5G02600	MA_131067g0010	shade<sun	Encodes a phloem mobile met	SODIUM POTASSIUM ROC	chloroplast-GO:0009507,c	559.9632658	-0.733728028	0.16578299	-4.425834234	9.61E-06	0.000221877
PITA_000050263	Low quality	AT5G65660	MA_940045g0010	shade>sun	hydroxyproline-rich glycoprotein family protein;(source:A		biological_process_unknow	64.23320992	0.665417696	0.21916551	3.036142408	0.002396262	0.025848468
PITA_000050368	High quality	AT1G66150	MA_8628986g0010	shade>sun	receptor-like transmembrane k	TRANSMEMBRANE KINASE	extracellular region-GO:00	288.304017	11.46749615	3.90689487	2.935194457	0.003333387	0.033506586
PITA_000050409	High quality	AT4G36430	MA_10227622g0010	shade<sun	Peroxidase superfamily protein;(source:Araport11)		cell wall-GO:0005618,extra	273.7379529	-2.115812476	0.56384961	-3.752441172	0.000175121	0.002845715
PITA_000050461	High quality	AT1G30220	MA_10276622g0010	shade>sun	Inositol transporter presenting	INOSITOL TRANSPORTER 2	carbohydrate transmembr	12.44533892	4.039954452	1.09617367	3.685505821	0.000228249	0.003584331
PITA_000050463	High quality	AT5G46330	MA_85663g0010	shade>sun	Encodes a leucine-rich repeat s	FLAGELLIN-SENSITIVE 2 (F	defense response by callo	185.748446	1.236585057	0.38836347	3.184092104	0.001452088	0.017266223
PITA_000050612	High quality	AT3G12670	MA_1481g0010	shade>sun	Cytidine triphosphate synthase	EMBRYO DEFECTIVE 2742	CTP biosynthetic process-(	44.28151789	0.88950528	0.24573155	3.619825232	0.000294802	0.004490032
PITA_000050631	High quality	AT1G71400	MA_96665g0030	shade<sun	Encodes a CLAVATA2 (CLV2)-re	RECEPTOR LIKE PROTEIN 1	extracellular region-GO:00	293.5194725	-1.751784909	0.30655781	-5.714370482	1.10E-08	4.27E-07
PITA_000050702	Low quality	AT5G52520	MA_10436163g0010	shade<sun	Encodes a chloroplast and mitc	OVULE ABORTION 6 (OVA	aminoacyl-tRNA synthetas	330.4058011	-1.186728767	0.16787946	-7.068933772	1.56E-12	1.25E-10
PITA_000050749	High quality	AT4G30410	MA_136773g0010	shade>sun	sequence-specific DNA binding	IBH1-LIKE 1 (IBL1)	brassinosteroid mediated	47.49713654	1.126182577	0.32444833	3.471069115	0.000518391	0.007295653
PITA_000050754	High quality	AT5G36120	MA_27435g0010	shade<sun	One of four Arabidopsis homol	COFACTOR ASSEMBLY, CO	chloroplast-GO:0009507,c	420.4699888	-1.009405381	0.15976661	-6.317999432	2.65E-10	1.37E-08
PITA_000050763	High quality	AT2G16500	MA_8291837g0010	shade<sun	Encodes a arginine decarboxyla	ARGININE DECARBOXYLAS	arginine catabolic process-	173.9638808	-0.817399452	0.25867331	-3.159968323	0.001577863	0.018525933
PITA_000050838	High quality	AT3G18680	MA_245601g0010	shade<sun	Encodes a functional UMP Kina	PLASTID 55 UMP KINASE (	chloroplast-GO:0009507,c	157.4489301	-0.638374703	0.18558094	-3.439872104	0.000581989	0.008029413
PITA_000050884	Low quality	AT1G28100	MA_42281g0010	shade<sun	hypothetical protein;(source:Araport11)		molecular_function_unkno	217.1649204	-0.669412485	0.2018913	-3.315707468	0.000914115	0.011714416
PITA_000050967	High quality	AT2G25870	MA_10429896g0010	shade<sun	Encodes an endoribonuclease	YBEY ENDORIBONUCLEAS	chloroplast-GO:0009507,c	162.581034	-1.134751008	0.18419875	-6.160470757	7.25E-10	3.40E-08
PITA_000051145	High quality	AT5G57480	MA_10067279g0010	shade>sun	P-loop containing nucleoside triphosphate hydrolases sup		ATPase activity-GO:00168	66.04330344	9.340010695	1.19569065	7.811393934	5.66E-15	6.92E-13
PITA_000051147	High quality	AT5G48730	MA_10431478g0010	shade<sun	Pentatricopeptide repeat (PPR) superfamily protein;(sour		biological_process_unknow	174.9283736	-0.800418057	0.18683385	-4.284116857	1.83E-05	0.000393789
PITA_000051233	High quality	AT1G08510	MA_191613g0010	shade<sun	Encodes an acyl-acyl carrier pro	FATTY ACYL-ACP THIOEST	acyl carrier activity-GO:00	96.71286259	-0.55139393	0.17512846	-3.148511325	0.001641044	0.019046075
PITA_000051313	High quality	AT5G55840	MA_12730g0010	shade<sun	PPR superfamily protein;(source:Araport11)		mitochondrion-GO:00057	326.4446207	-1.017710729	0.25689802	-3.961535931	7.45E-05	0.001346317
PITA_000051337	High quality	AT1G01820	MA_5289g0010	shade<sun	member of the peroxin11 (PEX	PEROXIN 11C (PEX11C)	extracellular region-GO:00	123.8117157	-0.647114199	0.14509894	-4.459813514	8.20E-06	0.000192628
PITA_000051350	High quality	AT5G54570	MA_952732g0010	shade>sun	beta glucosidase 41;(source:Ar	BETA GLUCOSIDASE 41 (B	beta-glucosidase activity-G	55.10489777	1.878328088	0.41739439	4.500127726	6.79E-06	0.000162655
PITA_000051456	Low quality	AT5G60600	MA_88006g0010	shade<sun	Encodes a chloroplast-localized	4-HYDROXY-3-METHYLBU	4 iron, 4 sulfur cluster binc	203.0397213	-2.30431315	0.57466386	-4.009845241	6.08E-05	0.001126879
PITA_000051501	High quality	AT1G27170	MA_4549g0010	shade>sun	transmembrane receptors / ATP binding protein;(source:A		ADP binding-GO:0043531,	69.52828052	9.416914753	1.22891436	7.662791713	1.82E-14	2.04E-12
PITA_000051520	Low quality	AT1G50660	MA_10429999g0020	shade>sun	actin cytoskeleton-regulatory complex pan-like protein;(sc		cytoplasm-GO:0005737	49.31192125	0.734677047	0.22649315	3.243705408	0.001179857	0.01447276
PITA_000051708	High quality	AT5G02860	MA_71865g0010	shade<sun	Pentatricopeptide repeat (PPR) superfamily protein;(sour		biological_process_unknow	250.5865127	-1.050929792	0.30344151	-3.46336858	0.000533457	0.007470177
PITA_000051785	High quality	AT5G46050	MA_890631g0010	shade<sun	Encodes a di- and tri-peptide tr	NRT1/ PTR FAMILY 5.2 (NF	defense response to bacte	541.5813262	-1.324170547	0.46214886	-2.865246828	0.004166843	0.039730731
PITA_000051869	High quality	AT5G07990	MA_20408g0020	shade<sun	Required for flavonoid 3' hydro	TRANSPARENT TESTA 7 (T	extracellular region-GO:00	263.6751934	-1.682793294	0.47713183	-3.526893825	0.000420465	0.006083445
PITA_000051918	High quality	AT4G25080	MA_10426641g0010	shade<sun	Encodes a protein with methyl	MAGNESIUM-PROTOPORI	chlorophyll biosynthetic p	438.9728402	-1.078301201	0.10518512	-10.25146179	1.17E-24	4.98E-22
PITA_000051936	Low quality	AT2G36770	MA_103636g0020	shade>sun	UDP-Glycosyltransferase superfamily protein;(source:Arap		chloroplast-GO:0009507,t	25.13738493	1.859951776	0.62630145	2.969738903	0.00298053	0.030693734

PITA_000051999	High quality	AT3G27650	MA_10429262g0010	shade>sun	LOB domain-containing protei	LOB DOMAIN-CONTAININ	biological_process_unknov	515.7646443	12.30659498	3.90683507	3.150016514	0.001632612	0.018983164
PITA_000052141	High quality	AT1G30510	MA_10435561g0010	shade<sun	Encodes a root-type ferredoxir	ROOT FNR 2 (RFNR2)	chloroplast-GO:0009507,c	138.4268735	-0.429300773	0.1371775	-3.129527491	0.001750877	0.020107976
PITA_000052243	Low quality	AT5G57625	MA_79998g0010	shade>sun	CAP (Cysteine-rich secretory pr	(ATCAPE5)	biological_process_unknov	28.3241393	1.615228171	0.35501014	4.549808575	5.37E-06	0.00013173
PITA_000052285	High quality	AT5G23850	MA_10425862g0010	shade>sun	O-glucosyltransferase rumi-like	protein (DUF821);(source:	endosome-GO:0005768,e›	253.0475283	2.071425506	0.53787871	3.851101497	0.000117588	0.002007829
PITA_000052296	High quality	AT1G22640	MA_130918g0010	shade<sun	MYB-type transcription factor (	MYB DOMAIN PROTEIN 3	response to wounding-GO	459.0665728	-0.751157013	0.22152198	-3.390891491	0.000696657	0.009315845
PITA_000052326	High quality	AT4G15530	MA_584406g0010	shade<sun	Encodes a dual-targeted protei	PYRUVATE ORTHOPHOSPI	chloroplast-GO:0009507,c	792.6924652	-1.142618672	0.15099392	-7.567316011	3.81E-14	4.04E-12
PITA_000052348	High quality	AT2G44020	MA_78196g0010	shade<sun	Mitochondrial transcription termination factor family prot		developmental process-G(	341.1078343	-1.020964939	0.12861565	-7.93810813	2.05E-15	2.70E-13
PITA_000052361	High quality	AT2G40490	MA_8643028g0010	shade<sun	Uroporphyrinogen decarboxyla	(HEME2)	chlorophyll biosynthetic p	347.5735277	-0.824194224	0.2637268	-3.125181853	0.001776952	0.02035144
PITA_000052367	High quality	AT2G03430	MA_10430558g0020	shade<sun	Ankyrin repeat family protein;(source:Araport11)		biological_process_unknov	280.0993407	-1.807445662	0.50605421	-3.571644353	0.000354747	0.005281057
PITA_000052444	High quality	AT3G14940	MA_20154g0020	shade<sun	Encodes a cytosolic phosphoer	PHOSPHOENOLPYRUVATE	apoplast-GO:0048046,chlc	212.4341642	-0.690053761	0.15372903	-4.488766907	7.16E-06	0.000170602
PITA_000052512	High quality	AT1G06430	MA_188257g0010	shade<sun	encodes a FtsH protease that is	FTSH PROTEASE 8 (ftsh8)	ATP-dependent peptidase	651.9331137	-0.996291051	0.12440063	-8.008730017	1.16E-15	1.61E-13
PITA_000052589	Low quality	AT4G33110	MA_54449g0010	shade<sun	S-adenosyl-L-methionine-dependent methyltransferases s		cytoplasm-GO:0005737,pla	310.1112791	-1.132572216	0.36563405	-3.097556742	0.00195123	0.022027766
PITA_000052658	High quality	AT4G36530	MA_196209g0010	shade<sun	alpha/beta-Hydrolases superfamily protein;(source:Arapo		chloroplast-GO:0009507,c	863.9431705	-1.611973232	0.22149607	-7.277660662	3.40E-13	3.09E-11
PITA_000052692	High quality	AT5G20270	MA_16904g0010	shade>sun	heptahelical transmembrane p	HEPTAHELICAL TRANSMEI	negative regulation of abs	85.06628243	0.595505054	0.17582168	3.386983084	0.000706657	0.009419609
PITA_000052706	Low quality	AT2G48070	MA_122925g0010	shade<sun	Encodes a chloroplast protein I	RESISTANCE TO PHYTOPH	chloroplast-GO:0009507,d	373.5543621	-0.733722456	0.18361941	-3.995887205	6.45E-05	0.001188224
PITA_000052916	High quality	AT2G35730	MA_801600g0010	shade>sun	Heavy metal transport/detoxifi	HEAVY METAL ASSOCIATE	metal ion transport-GO:00	81.92678222	1.602430272	0.5302695	3.021916736	0.002511796	0.026830793
PITA_000052925	High quality	AT5G47520	MA_113056g0010	shade<sun	RAB GTPase homolog A5A;(sou	RAB GTPASE HOMOLOG A	endomembrane system-G	164.4210013	-0.873629155	0.27210238	-3.210663458	0.001324289	0.015995486
PITA_000053079	High quality	AT1G58340	MA_2674g0010	shade>sun	Encodes a plant MATE (multidı	(ZF14)	antiporter activity-GO:001	64.11848567	1.219500484	0.39327428	3.100890482	0.001929396	0.021810568
PITA_000053146	High quality	AT2G13360	MA_18142g0010	shade<sun	Encodes a peroxisomal photor	ALANINE:GLYOXYLATE AM	alanine-glyoxylate transam	443.7672739	-1.145930515	0.15470696	-7.40710359	1.29E-13	1.26E-11
PITA_000053170	High quality	AT5G09850	MA_23914g0010	shade>sun	Transcription elongation factor	MEDIATOR 26C (MED26C)	cytosol-GO:0005829,nucle	41.86847741	0.84202023	0.25093942	3.355472106	0.000792296	0.010390908
PITA_000053298	Low quality	AT3G54220	MA_18234g0010	shade<sun	Encodes a member of a novel f	SCARECROW (SCR)	asymmetric cell division-G	125.5150161	-0.563618832	0.15843697	-3.557369442	0.000374587	0.00551172
PITA_000053311	High quality	AT5G65020	MA_9033347g0010	shade<sun	Annexins are calcium binding p	ANNEXIN 2 (ANNAT2)	calcium ion binding-GO:00	234.8110287	-0.922272397	0.32475604	-2.839892967	0.004512867	0.042247406
PITA_000053344	High quality	AT1G17100	MA_72682g0010	shade<sun	SOUL heme-binding family pro	HAEM-BINDING PROTEIN	biological_process_unknov	2702.56256	-2.486962364	0.85010387	-2.925480588	0.003439246	0.034351601
PITA_000053360	High quality	AT1G58370	MA_476611g0010	shade<sun	Encodes a protein with xylanas	(RXF12)	cell wall-GO:0005618,endc	259.1085817	-1.22392575	0.31002745	-3.947797978	7.89E-05	0.001414783
PITA_000053531	High quality	AT2G39890	MA_10433063g0010	shade>sun	Encodes a proline transporter '	PROLINE TRANSPORTER 1	amino acid transmembran	78.01438221	9.582547908	1.36317248	7.029593135	2.07E-12	1.62E-10
PITA_000053586	High quality	AT4G38250	MA_10433138g0010	shade>sun	Transmembrane amino acid tra	(AT4G38250)	amino acid transmembran	14.52952676	7.154573599	1.22272404	5.851339601	4.88E-09	2.02E-07
PITA_000053682	High quality	AT1G75290	MA_176417g0010	shade<sun	encodes a protein whose sequence is similar to an isoflavo		chloroplast-GO:0009507,c'	667.9863852	-2.966868477	0.2128849	-13.93649118	3.80E-44	7.38E-41
PITA_000053721	High quality	AT1G11290	MA_109016g0020	shade>sun	Pentatricopeptide Repeat Prot	CHLORORESPIRATORY REI	chloroplast-GO:0009507,e	42.8468381	1.267336029	0.29567594	4.286233193	1.82E-05	0.000390391
PITA_000053765	Low quality	AT5G63930	MA_43145g0010	shade>sun	Leucine-rich repeat protein kinase family protein;(source:		ATP binding-GO:0005524,(	197.2020646	10.91955683	3.90695751	2.794900329	0.005191572	0.046912018
PITA_000053852	High quality	AT1G56280	MA_141147g0010	shade<sun	Encodes a gene whose transcri	DROUGHT-INDUCED 19 (D	DNA-binding transcription	42.60412449	-2.02799152	0.56370491	-3.597611962	0.000321152	0.004841279
PITA_000053952	High quality	AT1G01280	MA_54958g0010	shade>sun	member of CYP703A CYP703A	CYTOCHROME P450, FAM	heme binding-GO:002003	29.100119	1.197101531	0.37652765	3.179319015	0.001476215	0.017454359
PITA_000053966	Low quality	AT4G36040	MA_116117g0010	shade>sun	Chaperone DnaJ-domain super	DNAJ11 (J11)	chloroplast-GO:0009507,n	25.96386577	2.385410167	0.54261709	4.396120622	1.10E-05	0.000251521
PITA_000053999	High quality	AT1G12680	MA_8587931g0010	shade>sun	phosphoenolpyruvate carboxyl	PHOSPHOENOLPYRUVATE	calcium-dependent protei	66.95080977	1.404044741	0.45731491	3.070192387	0.002139209	0.023683432
PITA_000054086	High quality	AT4G04950	MA_85544g0010	shade<sun	Encodes a monothiol glutaredc	MONOTHIOL GLUTAREDO	auxin polar transport-GO:(	165.0045771	-0.747755183	0.12996233	-5.75363014	8.73E-09	3.45E-07
PITA_000054122	High quality	AT5G53750	MA_20447g0010	shade>sun	CBS domain-containing protein;(source:Araport11)		AMP binding-GO:0016208	43.0410417	0.738582516	0.25465416	2.900335618	0.003727633	0.036537468
PITA_000054129	Low quality	AT1G70200	MA_10435608g0010	shade<sun	Encodes a RNA-Binding Proteir	(RBD1)	chloroplast-GO:0009507,p	244.6145701	-0.845512167	0.14958503	-5.652385005	1.58E-08	6.03E-07
PITA_000054151	High quality	AT3G26070	MA_10437185g0010	shade<sun	localized to chloroplasts	FIBRILLIN3A (FBN3A)	biological_process_unknov	132.4484808	-0.503518406	0.17976679	-2.800953497	0.005095186	0.046326942
PITA_000054188	High quality	AT3G18110	MA_712594g0010	shade<sun	Pentatricopeptide repeat (PPR	EMBRYO DEFECTIVE 1270	embryo development end	234.9545461	-0.991367792	0.34398468	-2.88201144	0.003951454	0.038196883
PITA_000054205	Low quality	AT1G68760	MA_11165g0010	shade>sun	Encodes a cytosol-localized nu	NUDIX HYDROLASE 1 (NU	8-oxo-7,8-dihydrodeoxygu	50.73610701	8.96066437	1.80014472	4.977746673	6.43E-07	1.87E-05
PITA_000054251	High quality	AT4G35290	MA_391859g0010	shade>sun	Encodes a putative glutamate ı	GLUTAMATE RECEPTOR 2	calcium channel activity-G	87.56454963	0.900947166	0.31055276	2.901108256	0.003718454	0.036486047
PITA_000054362	High quality	AT1G79220	MA_104598g0010	shade>sun	Mitochondrial transcription ter	(MTERF17)	chloroplast-GO:0009507,c	69.75226238	1.02590679	0.32406478	3.165746064	0.001546858	0.01822979
PITA_000054366	High quality	AT5G49030	MA_10436718g0010	shade<sun	tRNA synthetase class I (I, L, M	OVULE ABORTION 2 (OVA	aminoacyl-tRNA editing ac	315.7944433	-1.376724873	0.33891817	-4.062115865	4.86E-05	0.00093408
PITA_000054474	High quality	AT2G20780	MA_41476g0010	shade>sun	Major facilitator superfamily protein;(source:Araport11)		carbohydrate transmembr	96.27721077	1.76123538	0.57401983	3.068248313	0.002153176	0.023796277
PITA_000054480	Low quality	AT1G26650	MA_8219g0010	shade<sun	Son of sevenless protein;(source:Araport11)		biological_process_unknov	135.7238579	-1.301817656	0.19714183	-6.603457418	4.02E-11	2.41E-09
PITA_000054563	High quality	AT1G26560	MA_8591669g0010	shade>sun	beta glucosidase 40;(source:Ar	BETA GLUCOSIDASE 40 (B(	apoplast-GO:0048046,beta	77.87322738	0.552244025	0.19581776	2.820193726	0.004799466	0.044451908
PITA_000054576	High quality	AT1G48130	MA_92424g0010	shade<sun	encodes a protein similar to th	1-CYSTEINE PEROXIREDO›	cell redox homeostasis-GC	488.0007996	-1.083178053	0.22394984	-4.836699333	1.32E-06	3.64E-05
PITA_000054582	Low quality	AT1G60900	MA_96898g0010	shade>sun	Putative U2A65 splicing factor	(U2AF65B)	commitment complex-GO	107.7529485	1.431015	0.37390647	3.827200435	0.000129609	0.002187911
PITA_000054673	High quality	AT5G01130	MA_72874g0010	shade<sun	hypothetical protein (DUF674);(source:Araport11)		biological_process_unknov	112.6612395	-1.263702793	0.38449688	-3.286639887	0.001013904	0.012747088
PITA_000054725	High quality	AT2G15620	MA_142030g0010	shade<sun	Involved in the second step of	NITRITE REDUCTASE 1 (NII	apoplast-GO:0048046,chlc	87.09716829	-0.544971448	0.17316003	-3.147212673	0.00164835	0.0191045
PITA_000054827	Low quality	AT5G65780	MA_3958789g0010	shade>sun	Encodes a chloroplast branche	(ATBCAT-5)	branched-chain-amino-aci	37.06155863	1.262048347	0.38318237	3.293597109	0.000989142	0.012510608
PITA_000054955	High quality	AT3G14440	MA_10428505g0020	shade>sun	Encodes 9-<i>cis</i>-epoxycar	NINE-CIS-EPOXYCAROTEN	9-cis-epoxycarotenoid dio›	49.24190261	2.512788515	0.86617395	2.901020657	0.003719494	0.036486047
PITA_000055028	High quality	AT1G62020	MA_10428405g0010	shade>sun	Member of the Coat Protein I (	α1 COAT PROTEIN (8	COPI vesicle coat-GO:0030	248.463303	11.25294211	3.90691661	2.880261659	0.003973453	0.038380112
PITA_000055117	High quality	AT1G27170	MA_18929g0020	shade<sun	transmembrane receptors / ATP binding protein;(source:A		ADP binding-GO:0043531,	360.1755563	-2.861282078	0.26785705	-10.68212329	1.23E-26	6.10E-24
PITA_000055191	Low quality	AT5G67360	MA_10435769g0010	shade<sun	Encodes a subtilisin-like serine	(ARA12)	apoplast-GO:0048046,cell	145.6639659	-0.791985329	0.20111066	-3.938057471	8.21E-05	0.001469269
PITA_000055376	Low quality	AT4G10780	MA_733g0010	shade>sun	LRR and NB-ARC domains-containing disease resistance pr		ADP binding-GO:0043531,	94.18786582	0.966724686	0.18466648	5.234976652	1.65E-07	5.27E-06
PITA_000055435	High quality	AT1G07890	MA_79741g0010	shade<sun	Encodes a cytosolic ascorbate ı	ASCORBATE PEROXIDASE	cell wall-GO:0005618,cellu	664.5960758	-1.434387405	0.20308926	-7.062842206	1.63E-12	1.30E-10
PITA_000055438	High quality	AT2G30950	MA_14707g0010	shade<sun	Metalloprotease that functions	VARIEGATED 2 (VAR2)	ATP-dependent peptidase	5999.127405	-1.086477063	0.18096982	-6.003636833	1.93E-09	8.52E-08
PITA_000055513	Low quality	AT3G16890	MA_29431g0010	shade<sun	Encodes a mitochondrial penta	PENTATRICOPEPTIDE (PPF	mitochondrial ATP synthes	106.830783	-0.586990043	0.18712257	-3.136928135	0.00170728	0.019661002
PITA_000055578	High quality	AT1G16720	MA_21385g0010	shade<sun	Encodes HCF173, a protein wit	HIGH CHLOROPHYLL FLUO	chloroplast-GO:0009507,c	589.2011902	-1.332863858	0.21134181	-6.306673752	2.85E-10	1.46E-08
PITA_000055688	High quality	AT2G30695	MA_62309g0010	shade<sun	bacterial trigger factor;(source:Araport11)		chaperone-mediated prot(	1199.925597	-1.243842908	0.27438473	-4.533207477	5.81E-06	0.00014088
PITA_000055944	High quality	AT1G76890	MA_79619g0010	shade<sun	encodes a plant trihelix DNA-b	(GT2)	DNA-binding transcription	109.5822279	-0.505530742	0.18039743	-2.80231672	0.005073704	0.046194515
PITA_000055968	High quality	AT5G53350	MA_425966g0010	shade<sun	CLP protease regulatory subun	CLP PROTEASE REGULATO	ATP binding-GO:0005524,	128.5277542	-0.946906373	0.28299061	-3.346069962	0.000819657	0.010699665
PITA_000056002	High quality	AT1G48520	MA_889802g0010	shade<sun	Encodes Glu-tRNA(Gln) amidot	GLU-ADT SUBUNIT B (GAT	chloroplast-GO:0009507,c	348.1130393	-1.471998458	0.1183413	-12.43858569	1.61E-35	1.51E-32
PITA_000056048	High quality	AT1G67480	MA_10051g0010	shade<sun	Galactose oxidase/kelch repeat	superfamily protein;(sourc	cytoplasm-GO:0005737	88.91693831	-0.948487132	0.28395554	-3.340266334	0.000836981	0.010891979
PITA_000056112	High quality	AT1G17650	MA_10048552g0010	shade<sun	Glyoxylate reductase located ir	GLYOXYLATE REDUCTASE	chloroplast-GO:0009507,c	138.6034897	-0.663566169	0.22322898	-2.972580693	0.002953076	0.030548224
PITA_000056154	High quality	AT2G40000	MA_10429454g0010	shade>sun	ortholog of sugar beet HS1 PR(	ORTHOLOG OF SUGAR BEI	cellular response to hypox	16.83862594	7.369384387	1.77888638	4.142695373	3.43E-05	0.000684866
PITA_000056248	High quality	AT5G41000	MA_483137g0010	shade<sun	Arabidopsis thaliana metal-nic(	YELLOW STRIPE LIKE 4 (YS	chloroplast-GO:0009507,m	86.87824519	-1.681963454	0.40205996	-4.183364726	2.87E-05	0.000587974
PITA_000056335	Low quality	AT5G12020	MA_27119g0010	shade>sun	17.6 kDa class II heat shock prc	17.6 KDA CLASS II HEAT SH	cellular response to hypox	11.98548508	6.876587204	1.42741491	4.817511109	1.45E-06	3.96E-05
PITA_000056365	Low quality	AT4G27450	MA_109804g0010	shade>sun	aluminum induced protein witl	HYPOXIA RESPONSE UNK	cellular response to hypox	21.13611688	2.270121739	0.41930099	5.41406248	6.16E-08	2.15E-06
PITA_000056372	High quality	AT4G37560	MA_16526g0010	shade<sun	Indole-3-acetamide (IAM) hydr	IAMHYDROLASE12 (IAMH:	cytosol-GO:0005829,hydrc	181.0819365	-0.842278111	0.25587725	-3.291727272	0.000995741	0.012556297
PITA_000056392	High quality	AT1G26260	MA_42080g0010	shade>sun	Encodes CIB5 (cryptochrome-ir	CRYPTOCHROME-INTERAC	DNA-binding transcription	30.04446076	0.786814295	0.27876287	2.822521866	0.004764757	0.044211618
PITA_000056397	High quality	AT2G01820	MA_10431591g0010	shade<sun	Transmembrane kinase (TMK),	TRANSMEMBRANE KINASI	plant-type cell wall-GO:00(	283.2538374	-1.14877691	0.35756657	-3.212763782	0.001314644	0.015901829
PITA_000056400	High quality	AT1G08080	MA_10432594g0010	shade>sun	alpha carbonic anhydrase 7;(sc	ALPHA CARBONIC ANHYDI	carbonate dehydratase act	216.1242216	2.065883759	0.2371241	8.712247024	2.98E-18	6.21E-16
PITA_000056587	High quality	AT1G14590	MA_386090g0010	shade<sun	Nucleotide-diphospho-sugar transferase family protein;(sc		biological_process_unknov	126.6989735	-2.12166065	0.59486793	-3.566607865	0.000361632	0.005361622
PITA_000056746	Low quality	AT2G33680	MA_935792g0010	shade<sun	Tetratricopeptide repeat (TPR)	(RID4)	chloroplast-GO:0009507,ir	254.4619134	-1.255119701	0.4084731	-3.072710812	0.002121239	0.023505122
PITA_000056904	High quality	AT4G17900	MA_8773709g0010	shade>sun	PLATZ transcription factor fami	(PLATZ11)	biological_process_unknov	219.3337576	-11.40686264	3.90699054	-2.919603346	0.003504772	0.034766771
PITA_000056915	Low quality	AT1G72060	MA_7906475g0010	shade>sun	serine-type endopeptidase inhibitor;(source:Araport11)		extracellular region-GO:00	4.556570861	4.473751939	1.54861869	2.888866043	0.003866337	0.037474646
PITA_000056970	High quality	AT4G14050	MA_38687g0010	shade<sun	Pentatricopeptide repeat (PPR	MITOCHONDRIAL EDITING	chloroplast-GO:0009507,c	242.955938	-1.35778247	0.34285311	-3.960245506	7.49E-05	0.001351676
PITA_000057171	High quality	AT4G37000	MA_10430866g0010	shade<sun	Mutants have spontaneous spr	ACCELERATED CELL DEATH	chlorophyll catabolic proc(	239.7538734	-0.774688132	0.23321619	-3.321759632	0.000894517	0.01152182
PITA_000057185	High quality	AT3G01790	MA_109054g0010	shade<sun	Ribosomal protein L13 family protein;(source:Araport11)		chloroplast-GO:0009507,m	482.2457812	-1.043690402	0.16004861	-6.521083929	6.98E-11	3.96E-09
PITA_000057319	High quality	AT4G21210	MA_17639g0020	shade<sun	Encodes a PPDK regulatory prc	PPDK REGULATORY PROTI	ATP binding-GO:0005524,(	264.4025105	-0.960934281	0.15581717	-6.167062865	6.96E-10	3.30E-08
PITA_000057367	High quality	AT3G54050	MA_41114g0010	shade<sun	Encodes a chloroplastic fructos	HIGH CYCLIC ELECTRON FL	apoplast-GO:0048046,chlc	1173.663595	-1.455881931	0.19982893	-7.285641383	3.20E-13	2.93E-11
PITA_000057448	Low quality	AT3G24000	MA_103380g0010	shade<sun	Tetratricopeptide repeat (TPR)-like superfamily protein;(s(		mitochondrion-GO:000573	246.5632828	-1.003404739	0.31304684	-3.205286311	0.001349282	0.016258428
PITA_000057619	Low quality	AT1G63300	MA_180303g0010	shade<sun	Myosin heavy chain-related protein;(source:Araport11)		biological_process_unknov	155.2107569	-0.860257568	0.27537758	-3.123920113	0.001784589	0.02041149
PITA_000057660	High quality	AT5G62940	MA_52474g0010	shade>sun	HCA2 induces the formation of	HIGH CAMBIAL ACTIVITY2	DNA-binding transcription	43.53385886	0.814689326	0.25483882	3.196880776	0.001389223	0.016628482
PITA_000057665	High quality	AT2G39540	MA_25298g0010	shade>sun	Gibberellin-regulated family protein;(source:Araport11)		extracellular region-GO:00	59.00234605	1.33998937	0.26137013	5.12678847	2.95E-07	9.01E-06
PITA_000057690	High quality	AT5G54510	MA_10432413g0020	shade>sun	Encodes an IAA-amido synthas	DWARF IN LIGHT 1 (DFL1)	acid-amino acid ligase acti	32.62218313	1.847516197	0.66587238	2.774580024	0.005527303	0.048980531
PITA_000057797	Low quality	AT3G23760	MA_36893g0010	shade<sun	transferring glycosyl group transferase;(source:Araport11)		biological_process_unknov	238.3901096	-1.529621449	0.31421835	-4.868020781	1.13E-06	3.14E-05
PITA_000057878	High quality	AT1G05675	MA_732668g0010	shade<sun	UDP-Glycosyltransferase superfamily protein;(source:Arap		quercetin 3-O-glucosyltrar	217.8698135	-2.106788059	0.33019867	-6.380364994	1.77E-10	9.30E-09
PITA_000057946	High quality	AT3G57520	MA_139754g0010	shade<sun	SIP2 encodes a raffinose-specil	SEED IMBIBITION 2 (SIP2)	cytoplasm-GO:0005737,ga	5390.081437	-2.133339234	0.16998528	-12.55014092	3.97E-36	4.00E-33
PITA_000058048	High quality	AT2G24090	MA_181338g0010	shade<sun	Ribosomal protein L35;(source	PLASTID RIBOSOMAL PRO	chloroplast-GO:0009507,la	388.4078888	-0.924153859	0.1525057	-6.059798953	1.36E-09	6.22E-08
PITA_000058078	High quality	AT2G18990	MA_10435712g0010	shade<sun	thioredoxin-like/ATP-binding p	THIOREDOXIN DOMAIN-C(	cytoplasm-GO:0005737	84.66446251	-0.73097009	0.20769006	-3.519523774	0.000432322	0.00623751
PITA_000058205	High quality	AT5G06720	MA_111431g0010	shade>sun	Encodes a peroxidase with div(	PEROXIDASE 2 (PA2)	defense response to nema	57.78313998	3.157692688	0.42524096	7.425655012	1.12E-13	1.10E-11
PITA_000058210	High quality	AT5G20950	MA_41345g0010	shade>sun	Encodes a beta-glucosidase inv	(BGLC1)	apoplast-GO:0048046,beta	20.88414628	4.520865104	1.5546971	2.907875168	0.003638936	0.035793275
PITA_000058250	Low quality	AT3G25690	MA_211108g0010	shade<sun	actin binding protein required	CHLOROPLAST UNUSUAL I	chloroplast-GO:0009507,c	563.9712435	-1.219359344	0.17135448	-7.116005126	1.11E-12	9.31E-11
PITA_000058378	Low quality	AT5G43830	MA_12061g0010	shade>sun	aluminum induced protein with	YGL and LRDR motifs;(sou	chloroplast-GO:0009507,c'	14.66186731	7.170157439	1.92874772	3.717519586	0.000201188	0.003209343
PITA_000058387	High quality	AT1G77490	MA_119996g0010	shade<sun	Encodes a chloroplastic thylakc	THYLAKOIDAL ASCORBATE	cellular response to oxidat	126.4359827	-0.784196553	0.23507216	-3.335982203	0.000849986	0.011032758
PITA_000058463	High quality	AT5G43860	MA_337265g0010	shade>sun	Encodes a chlorophyllase, the f	CHLOROPHYLLASE 2 (CLH2	chlorophyll catabolic proc(	39.26468922	0.848083161	0.28634204	2.961783609	0.003058627	0.031318946
PITA_000058475	High quality	AT1G61560	MA_40975g0010	shade>sun	A member of a large family of :	MILDEW RESISTANCE LOC	mitochondrion-GO:000573	39.22721969	0.934357236	0.27223798	3.432134022	0.000598852	0.008230537
PITA_000058518	High quality	AT1G48100	MA_101764g0010	shade<sun	Pectin lyase-like superfamily pı	POLYGALACTURONASE IN'	carbohydrate metabolic pı	280.2054859	-0.950066464	0.16858044	-5.63568617	1.74E-08	6.60E-07
PITA_000058547	Low quality	AT5G23530	MA_131537g0010	shade>sun	carboxyesterase 18;(source:Ara	CARBOXYESTERASE 18 (C›	carboxylic ester hydrolase	44.07262025	1.501041792	0.46423131	3.233391964	0.001223296	0.014940205
PITA_000058552	High quality	AT2G28080	MA_173000g0010	shade<sun	UDP-Glycosyltransferase superfamily protein;(source:Arap		transferase activity, transf(	793.7649855	-0.916471294	0.21503595	-4.261944458	2.03E-05	0.00043058
PITA_000058555	Low quality	AT4G10840	MA_82562g0010	shade<sun	CMU1 and CMU2 along with FI	KINESIN LIGHT CHAIN-REL	cortical microtubule-GO:0(	195.8760385	-11.24464201	1.27140912	-8.844235735	9.22E-19	2.00E-16
PITA_000058627	Low quality	AT3G06210	MA_10434888g0020	shade<sun	ARM repeat superfamily protein;(source:Araport11)		biological_process_unknov	61.40454676	-2.170252651	0.54510718	-3.981331971	6.85E-05	0.001253322
PITA_000058678	High quality	AT3G48770	MA_10426213g0030	shade>sun	ATP/DNA binding protein;(source:Araport11)		biological_process_unknov	45.67490932	8.808895675	1.58492043	5.557941896	2.73E-08	1.01E-06
PITA_000058730	High quality	AT4G01050	MA_157164g0010	shade<sun	hydroxyproline-rich glycoprote	THYLAKOID RHODANESE-L	chloroplast-GO:0009507,c	981.1634067	-1.892702961	0.22925944	-8.255725344	1.51E-16	2.52E-14
PITA_000058755	Low quality	AT2G38140	MA_202598g0010	shade<sun	plastid-specific ribosomal prot(	PLASTID-SPECIFIC RIBOSO	chloroplast-GO:0009507,c	267.3185335	-0.954389029	0.16994449	-5.615886728	1.96E-08	7.38E-07
PITA_000058780	High quality	AT5G19730	MA_20853g0010	shade<sun	Pectin lyase-like superfamily protein;(source:Araport11)		carboxylic ester hydrolase	497.3485009	-0.781556173	0.25071525	-3.117306107	0.00182512	0.0207996
PITA_000058792	High quality	AT3G17860	MA_6326g0010	shade<sun	JAZs are direct targets of the S	JASMONATE-ZIM-DOMAIN	identical protein binding-(	97.65579217	-0.595796528	0.17644802	-3.376612058	0.000733845	0.009735699
PITA_000058807	High quality	AT3G49170	MA_27147g0010	shade<sun	Tetratricopeptide repeat (TPR)	EMBRYO DEFECTIVE 2261	chloroplast-GO:0009507,e	140.4033946	-1.616353069	0.574291	-2.81451922	0.004885026	0.045051689
PITA_000058842	High quality	AT2G20830	MA_206805g0010	shade<sun	folic acid binding / transferase;(source:Araport11)		chloroplast-GO:0009507,f(	268.696186	-1.529461878	0.23530503	-6.499911653	8.04E-11	4.51E-09
PITA_000058854	High quality	AT1G13380	MA_10435104g0010	shade<sun	sodium/hydrogen exchanger (DUF1218);(source:Araport1		biological_process_unknov	77.22786812	-1.119539219	0.22928366	-4.882769329	1.05E-06	2.93E-05
PITA_000059094	High quality	AT3G08760	MA_46136g0010	shade>sun	Encodes an osmotic stress-indu	(ATSIK)	ATP binding-GO:0005524,ı	55.8202915	0.701020302	0.23838814	2.94066774	0.003275056	0.033012357
PITA_000059118	Low quality	AT4G33300	MA_99935g0010	shade>sun	Encodes a member of the ADR	ADR1-LIKE 1 (ADR1-L1)	ADP binding-GO:0043531,	9.584772339	6.556338573	1.60280834	4.090531861	4.30E-05	0.000836234
PITA_000059173	Low quality	AT5G23190	MA_10428260g0010	shade<sun	cytochrome P450 CYP86B1, nu	CYTOCHROME P450, FAM	chloroplast-GO:0009507,e	416.8560941	-0.800010723	0.15094865	-5.299886677	1.16E-07	3.82E-06
PITA_000059236	Low quality	AT2G21100	MA_10068901g0010	shade<sun	Disease resistance-responsive (dirigent-like protein) famil		cell differentiation-<br>GO:0030154,tissue<br>development-<br>GO:0009888,root<br>development-<br>GO:0048364	240.642197	-1.243490011	0.42621177	-2.917540281	0.003528041	0.034920403
PITA_000059264	High quality	AT1G47740	MA_10431766g0010	shade<sun	PPPDE putative thiol peptidase family protein;(source:Ara		cytoplasm-GO:0005737,pr	281.0280587	-0.814858277	0.23154291	-3.519253824	0.000432762	0.006240291
PITA_000059270	High quality	AT1G65730	MA_46073g0010	shade<sun	Arabidopsis thaliana metal-nic(	YELLOW STRIPE LIKE 7 (YS	membrane-GO:0016020,n	207.8611781	-2.493030822	0.3988458	-6.250613257	4.09E-10	2.01E-08
PITA_000059284	High quality	AT4G11400	MA_10429133g0010	shade>sun	ARID/BRIGHT DNA-binding , ELM2 domain and myb-like D		nucleus-GO:0005634,prot(	39.26548095	8.592232443	1.32242259	6.497342448	8.18E-11	4.56E-09
PITA_000059389	High quality	AT1G56220	MA_15605g0010	shade>sun	Dormancy/auxin associated family protein;(source:Arapor		molecular_function_unkn(	12.53879812	2.531812631	0.56706518	4.464764768	8.02E-06	0.000188754
PITA_000059400	High quality	AT1G69310	MA_120697g0010	shade>sun	Encodes WRKY57, a member o	WRKY DNA-BINDING PROT	DNA-binding transcription	50.13606614	0.798860524	0.23396247	3.41448153	0.000639035	0.008674095
PITA_000059482	Low quality	AT3G57360	MA_10437030g0010	shade<sun	tRNA-splicing endonuclease subunit;(source:Araport11)		nucleus-GO:0005634,tRNA	74.79035925	-0.594011658	0.21131051	-2.81108432	0.004937484	0.045408635
PITA_000059623	High quality	AT1G02140	MA_10426266g0010	shade>sun	Encodes a homologue of the e:	MAGO NASHI (MAGO)	embryo development end	182.9277221	10.81115986	3.90697298	2.767144773	0.005654964	0.049796852
PITA_000059870	High quality	AT4G02780	MA_10433916g0010	shade<sun	Catalyzes the conversion of ger	GA REQUIRING 1 (GA1)	chloroplast-GO:0009507,d	112.9927078	-2.074891789	0.49756153	-4.170120991	3.04E-05	0.000616206
PITA_000059899	High quality	AT3G30180	MA_31668g0010	shade<sun	Encodes a cytochrome p450 er	BRASSINOSTEROID-6-OXII	brassinosteroid biosynthet	323.8381208	-0.99057387	0.16871185	-5.871394764	4.32E-09	1.80E-07
PITA_000059957	Low quality	AT5G19140	MA_762051g0010	shade>sun	aluminum induced protein witl	(AILP1)	chloroplast-GO:0009507,c'	32.00143343	2.231543607	0.35679004	6.254500865	3.99E-10	1.98E-08
PITA_000059969	Low quality	AT2G42320	MA_10083347g0010	shade>sun	nucleolar protein gar2-like protein;(source:Araport11)		nuclear pore-GO:0005643,	119.3739551	0.9076334	0.27160948	3.34168526	0.000832714	0.010853258
PITA_000059996	Low quality	AT3G27160	MA_249305g0010	shade<sun	GHS1 encodes plastid ribosom:	GLUCOSE HYPERSENSITIVI	chloroplast-GO:0009507,c	507.4419984	-1.085089341	0.16521965	-6.567556235	5.11E-11	2.99E-09
PITA_000060010	High quality	AT5G07050	MA_71776g0010	shade>sun	nodulin MtN21-like transporte	USUALLY MULTIPLE ACIDS	chloroplast-GO:0009507,p	99.48442556	2.61879636	0.31991865	8.185819547	2.70E-16	4.30E-14
PITA_000060012	Low quality	AT1G15550	MA_666770g0010	shade>sun	Involved in later steps of the gi	GIBBERELLIN 3-OXIDASE 1	cytoplasm-GO:0005737,di(	17.89254118	1.121176342	0.3930433	2.852551741	0.004336975	0.041011816

PITA_000060035	Low quality	AT2G46600	MA_1552801g0010	shade>sun	Calcium-binding EF-hand family protein;(source:Araport11		calcium ion binding-GO:00	26.99643856	1.686881507	0.34641758	4.869503191	1.12E-06	3.12E-05
PITA_000060044	High quality	AT5G65380	MA_9267g0020	shade<sun	MATE efflux family protein;(source:Araport11)		antiporter activity-GO:001	242.0006383	-1.136957489	0.40585689	-2.801375349	0.00508853	0.046296073
PITA_000060045	High quality	AT1G50940	MA_6571812g0010	shade<sun	Encodes the electron transfer f	ELECTRON TRANSFER FLAV	copper ion binding-GO:00	113.4042306	-0.695448236	0.21645376	-3.212918255	0.001313937	0.015900905
PITA_000060081	Low quality	AT5G05280	MA_10436028g0020	shade>sun	Encodes a RING-finger E3 ligase	DEFECTIVE IN ANTHER DE	anther dehiscence-GO:000	265.7372775	11.35002918	1.64246223	6.910374523	4.83E-12	3.47E-10
PITA_000060121	Low quality	AT5G12180	MA_18543g0010	shade>sun	member of Calcium Dependen	CALCIUM-DEPENDENT PR	calcium ion binding-GO:00	23.93574792	1.298616642	0.32718141	3.969102818	7.21E-05	0.001306149
PITA_000060278	Low quality	AT2G21220	MA_95603g0010	shade<sun	SAUR-like auxin-responsive pro	SMALL AUXIN UPREGULAT	mitochondrion-GO:00057	124.2138474	-1.724229388	0.38376122	-4.492974491	7.02E-06	0.000167581
PITA_000060349	High quality	AT2G28840	MA_10426600g0010	shade>sun	hypothetical protein;(source:A	XB3 ORTHOLOG 1 IN ARAE	chloroplast-GO:0009507,n	222.7684361	11.09543652	3.90693476	2.839933913	0.004512288	0.042247406
PITA_000060447	High quality	AT5G09320	MA_10432680g0020	shade<sun	vacuolar protein sorting-associ	(VPS9B)	cytoplasm-GO:0005737,m	127.476876	-0.786153154	0.25486896	-3.084538597	0.002038682	0.022810802
PITA_000060555	High quality	AT1G72470	MA_89549g0010	shade>sun	A member of EXO70 gene fami	EXOCYST SUBUNIT EXO70	cytosol-GO:0005829,exocy	52.72433259	0.799999108	0.25626583	3.121754878	0.001797766	0.020543565
PITA_000060576	High quality	AT5G46220	MA_33920g0010	shade<sun	Encodes a protein with alkaline	TURGOR REGULATION DEI	chloroplast-GO:0009507,d	111.1623953	-1.682421031	0.28986204	-5.804213134	6.47E-09	2.63E-07
PITA_000060617	High quality	AT3G22960	MA_3663g0010	shade<sun	encodes a chloroplast pyruvate	(PKP-ALPHA)	chloroplast-GO:0009507,c	191.1461983	-0.783765901	0.20327843	-3.855627406	0.000115433	0.001977734
PITA_000060758	Low quality	AT5G22640	MA_10427295g0010	shade<sun	EMB1211 is a MORN (multiple	EMBRYO DEFECTIVE 1211	chloroplast-GO:0009507,c	197.9838331	-0.837962419	0.18172024	-4.611277262	4.00E-06	0.000100329
PITA_000060772	High quality	AT5G58250	MA_46214g0010	shade<sun	Involved in tetrapyrrole biosyn	EMBRYO DEFECTIVE 3143	chlorophyll biosynthetic p	432.7951888	-1.085308759	0.11034119	-9.83593526	7.88E-23	2.72E-20
PITA_000060797	High quality	AT2G33470	MA_403012g0010	shade>sun	glycolipid transfer protein 1;(sc	GLYCOLIPID TRANSFER PR	ceramide 1-phosphate bin	25.93603792	1.141643052	0.34626452	3.297025761	0.000977145	0.012383722
PITA_000060813	High quality	AT5G56190	MA_57255g0010	shade>sun	Transducin/WD40 repeat-like superfamily protein;(source		biological_process_unkno	56.78486599	9.124214987	1.46399127	6.232424454	4.59E-10	2.23E-08
PITA_000060831	High quality	AT2G32440	MA_107159g0010	shade<sun	ent-kaurenoic acid hydroxylase	ENT-KAURENOIC ACID HYI	brassinosteroid biosynthet	153.5326896	-0.903441085	0.20671377	-4.370492979	1.24E-05	0.000278648
PITA_000060907	Low quality	AT5G06570	MA_10207543g0010	shade<sun	alpha/beta-Hydrolases superfamily protein;(source:Arapo		hydrolase activity-GO:001(	115.4437625	-4.260810269	0.90550456	-4.705454214	2.53E-06	6.61E-05
PITA_000061023	Low quality	AT1G53820	MA_9143538g0010	shade>sun	RING/U-box superfamily protei	ARABIDOPSIS T??XICOS EI	nucleus-GO:0005634,prote	59.2072411	9.184426238	1.41495194	6.490981071	8.53E-11	4.74E-09
PITA_000061058	Low quality	AT4G28890	MA_179334g0010	shade>sun	RING/U-box superfamily protei	ARABIDOPSIS T??XICOS EI	nucleus-GO:0005634,prote	38.60879845	1.061837352	0.37948705	2.798085845	0.005140645	0.046535198
PITA_000061115	Low quality	AT1G05835	MA_7163462g0010	shade>sun	PhD finger protein.		cell fate determination-GC	10.32497763	1.896515019	0.61631211	3.077198992	0.002089558	0.023235733
PITA_000061126	High quality	AT4G21960	MA_10427936g0010	shade<sun	Encodes AT4g21960 (AT4g219(	(PRXR1)	heme binding-GO:002003	106.8704033	-0.910288785	0.24988385	-3.642847658	0.000269638	0.004166839
PITA_000061160	High quality	AT5G21326	MA_819000g0010	shade<sun	Ca2+regulated serine-threonin	CALCINEURIN B-LIKE PROT	cytoplasm-GO:0005737,cy	108.9409356	-1.99566594	0.57959938	-3.44318163	0.000574913	0.007949182
PITA_000061384	High quality	AT3G62550	MA_111185g0010	shade>sun	Adenine nucleotide alpha hydrolases-like superfamily pro		nucleus-GO:0005634,vacu	54.05772206	0.73774041	0.25806179	2.858774315	0.004252812	0.040437292
PITA_000061549	Low quality	AT1G05385	MA_341577g0010	shade<sun	Encodes a Psb27 homolog invc	LOW PSII ACCUMULATION	chloroplast-GO:0009507,c	126.0467252	-0.677934898	0.22411943	-3.024882329	0.002487299	0.02667078
PITA_000061745	High quality	AT2G42040	MA_169331g0010	shade<sun	WRC protein;(source:Araport11)		developmental process-GC	208.3311783	-1.124619764	0.27312286	-4.117633166	3.83E-05	0.000752439
PITA_000061896	Low quality	AT3G19920	MA_10434946g0010	shade<sun	BTB/POZ domain protein;(source:Araport11)		molecular_function_unkn	137.2357824	-0.757999273	0.24875284	-3.047198438	0.002309852	0.025174788
PITA_000061992	High quality	AT3G51680	MA_10436701g0010	shade<sun	NAD(P)-binding Rossmann-folc	SHORT-CHAIN DEHYDROG	cytoplasm-GO:0005737	510.6531173	-1.010279023	0.34175446	-2.956154637	0.003115009	0.031780145
PITA_000062058	High quality	AT2G02990	MA_95383g0020	shade<sun	Encodes a member of the ribo	RIBONUCLEASE 1 (RNS1)	aging-GO:0007568,anthoc	178.708634	-2.295612626	0.76356107	-3.006455807	0.002643125	0.027879383
PITA_000062171	High quality	AT4G39230	MA_419572g0010	shade>sun	encodes a protein whose sequ	PHENYLCOUMARAN BENZ	cytoplasm-GO:0005737,lig	33.98534698	1.436755831	0.42151781	3.40852934	0.000653141	0.008832282
PITA_000062183	High quality	AT1G30380	MA_16054g0010	shade<sun	Encodes subunit K of photosys	PHOTOSYSTEM I SUBUNIT	chloroplast-GO:0009507,c	299.8524311	-0.681063024	0.23295783	-2.923546449	0.003460686	0.034524722
PITA_000062213	High quality	AT3G05950	MA_160196g0010	shade>sun	RmlC-like cupins superfamily protein;(source:Araport11)		cell wall-GO:0005618,extr	165.6450884	10.66831235	1.76433856	6.046635608	1.48E-09	6.66E-08
PITA_000062225	Low quality	AT1G68490	MA_8052g0010	shade>sun	translocase subunit seca;(source:Araport11)		biological_process_unkno	39.86541575	0.844990097	0.25845522	3.26938687	0.001077808	0.013436641
PITA_000062337	High quality	AT5G14780	MA_10431917g0020	shade<sun	Encodes a NAD-dependent for	FORMATE DEHYDROGENA	chloroplast-GO:0009507,c	234.4016229	-1.875803456	0.46396553	-4.042980253	5.28E-05	0.001002268
PITA_000062339	High quality	AT2G15580	MA_13397g0020	shade>sun	RING/U-box superfamily protein;(source:Araport11)		cytoplasm-GO:0005737,mi	27.73403121	1.160062742	0.36886202	3.144977479	0.001660996	0.019206934
PITA_000062340	High quality	AT1G68020	MA_48114g0010	shade>sun	Encodes an enzyme putatively	(ATTPS6)	alpha,alpha-trehalose-pho	32.70632024	0.886851161	0.29763453	2.979664893	0.002885639	0.029998272
PITA_000062383	High quality	AT4G36870	MA_6763g0010	shade<sun	Encodes a member of the BEL	BEL1-LIKE HOMEODOMAI	DNA-binding transcription	261.5487936	-0.609289909	0.10463827	-5.822820794	5.79E-09	2.37E-07
PITA_000062453	High quality	AT5G38450	MA_35890g0010	shade<sun	cytochrome P450, family 735, s	CYTOCHROME P450, FAM	heme binding-GO:002003	202.0778907	-1.143767697	0.26538361	-4.30986559	1.63E-05	0.000354238
PITA_000062492	Low quality	AT5G15280	MA_100612g0010	shade>sun	Pentatricopeptide repeat (PPR) superfamily protein;(sour		biological_process_unkno	44.15920761	8.760526892	1.34654317	6.505938381	7.72E-11	4.36E-09
PITA_000062495	Low quality	AT3G30530	MA_38931g0010	shade>sun	basic leucine-zipper 42;(source	BASIC LEUCINE-ZIPPER 42	DNA-binding transcription	56.39632936	2.401679979	0.69913224	3.435229924	0.000592051	0.0081504
PITA_000062530	High quality	AT3G61250	MA_128244g0010	shade>sun	LATE MERISTEM IDENTITY2 (LI	MYB DOMAIN PROTEIN 1	DNA-binding transcription	9.258224804	5.505383622	1.28191403	4.294658995	1.75E-05	0.000377463
PITA_000062536	High quality	AT5G14040	MA_89717g0010	shade<sun	Encodes a mitochondrial phos	PHOSPHATE TRANSPORTE	cell wall-GO:0005618,chlo	85.05465716	-1.372530615	0.49329562	-2.782369348	0.005396359	0.048145699
PITA_000062584	Low quality	AT3G51780	MA_728187g0010	shade>sun	A member of Arabidopsis BAG	BCL-2-ASSOCIATED ATHAN	adenyl-nucleotide exchang	67.71768738	2.12857965	0.59711207	3.564790867	0.000364146	0.005392703
PITA_000062645	High quality	AT2G46750	MA_9092998g0010	shade<sun	Encodes a homolog of rat L-gul	L -GULONO-1,4-LACTONE	D-arabinono-1,4-lactone o	129.2389392	-1.284471155	0.29551441	-4.346560086	1.38E-05	0.000305934
PITA_000062679	Low quality	AT4G19100	MA_268175g0010	shade<sun	Protein of unknown function (I	PHOTOSYNTHESIS AFFECT	chloroplast-GO:0009507,m	364.4511813	-1.15131794	0.13692627	-8.408305536	4.16E-17	7.24E-15
PITA_000062737	High quality	AT1G14590	MA_10434802g0010	shade>sun	Nucleotide-diphospho-sugar transferase family protein;(s		biological_process_unkno	75.68645381	0.915768157	0.2860469	3.201461575	0.001367323	0.016413084
PITA_000062762	Low quality	AT5G08650	MA_742675g0010	shade<sun	Critical for chloroplast protein	(CPLEPA)	chloroplast-GO:0009507,m	211.1620937	-1.046570685	0.2817147	-3.715002074	0.000203202	0.003237369
PITA_000062909	Low quality	AT4G24130	MA_5629g0010	shade<sun	DUF538 family protein (Protein of unknown function, DUF		biological_process_unkno	188.622005	-0.830365281	0.23451287	-3.540808934	0.000398902	0.005818575
PITA_000062913	Low quality	AT1G19400	MA_401170g0010	shade<sun	Tail-anchored (TA) OEP membr	ERYTHRONATE-4PHOSPHA	nucleus-GO:0005634,phos	116.5268094	-1.698032052	0.25393123	-6.686976007	2.28E-11	1.42E-09
PITA_000062953	High quality	AT5G60700	MA_875703g0010	shade<sun	glycosyltransferase family protein 2;(source:Araport11)		mitochondrion-GO:00057	641.9313372	-1.182477203	0.19723007	-5.995420619	2.03E-09	8.92E-08
PITA_000063012	High quality	AT5G65360	MA_10432805g0020	shade>sun	Histone superfamily protein;(s	HISTONE 3.1 (H3.1)	chromocenter-GO:001036	228.2599775	11.13061353	1.65013059	6.745292516	1.53E-11	9.98E-10
PITA_000063013	High quality	AT4G00730	MA_93471g0010	shade<sun	Encodes a homeodomain prot	ANTHOCYANINLESS 2 (AN	anthocyanin accumulation	248.347939	-11.58605066	3.90696416	-2.965486804	0.003022043	0.031007293
PITA_000063210	Low quality	AT2G01610	MA_8433628g0010	shade<sun	Plant invertase/pectin methylesterase inhibitor superfami		enzyme inhibitor activity-(	189.4664651	-2.745630459	0.26780103	-10.25250151	1.15E-24	4.98E-22
PITA_000063248	Low quality	AT2G38500	MA_24218g0010	shade>sun	2-oxoglutarate (2OG) and Fe(II)-dependent oxygenase sup		biological_process_unkno	25.5014773	1.236058998	0.34147117	3.619804845	0.000294825	0.004490032
PITA_000063502	High quality	AT5G46240	MA_4531g0010	shade<sun	Encodes a potassium channel p	POTASSIUM CHANNEL IN	identical protein binding-(	141.6429746	-0.718352661	0.24855403	-2.890126757	0.003850865	0.037396542
PITA_000063520	Low quality	AT1G03220	MA_10426833g0010	shade>sun	Eukaryotic aspartyl protease fa	SECRETED ASPARTIC PROT	aspartic-type endopeptida	37.37407101	8.521084336	1.56580936	5.44196794	5.27E-08	1.86E-06
PITA_000063628	High quality	AT1G55940	MA_10110965g0010	shade<sun	cytochrome P450 family protei	CYTOCHROME P450, FAM	brassinosteroid biosynthet	866.1915403	-0.747740361	0.24423792	-3.061524407	0.00220213	0.024254993
PITA_000063672	High quality	AT1G54520	MA_10432911g0010	shade<sun	FLAP1 is a chloroplast membra	FLUCTUATING-LIGHT- ACC	chloroplast-GO:0009507,c	580.5750905	-2.152101202	0.16880466	-12.7490631	3.16E-37	3.62E-34
PITA_000063673	High quality	AT5G03650	MA_10430611g0010	shade<sun	Encodes starch branching enzy	STARCH BRANCHING ENZY	1,4-alpha-glucan branchin	547.9426067	-0.946646483	0.19490716	-4.856909632	1.19E-06	3.32E-05
PITA_000063732	High quality	AT1G80160	MA_193904g0010	shade>sun	Vicinal oxygen chelate (VOC) su	GLYOXYLASE I 7 (GLYI7)	cytoplasm-GO:0005737	10.38813313	1.769690952	0.58604882	3.019698876	0.002530261	0.027005157
PITA_000063822	High quality	AT2G04570	MA_166543g0010	shade<sun	GDSL-motif esterase/acyltrans	OCCLUDED STOMATAL PC	acyl-[acyl-carrier-protein] l	137.6994088	-1.080206407	0.31780543	-3.398955143	0.000676438	0.009088849
PITA_000063850	Low quality	AT4G37300	MA_10429738g0010	shade>sun	maternal effect embryo arrest	MATERNAL EFFECT EMBR	cytosol-GO:0005829,embr	34.90912787	8.422244595	1.2350572	6.81931543	9.15E-12	6.27E-10
PITA_000064023	High quality	AT4G37850	MA_50811g0010	shade<sun	basic helix-loop-helix (bHLH) DNA-binding superfamily pro		DNA-binding transcription	1106.264329	-1.507144542	0.27182842	-5.544470102	2.95E-08	1.08E-06
PITA_000064096	Low quality	AT5G59500	MA_842948g0010	shade<sun	protein C-terminal S-isoprenylcysteine carboxyl O-methylt		extracellular region-GO:00	363.0764974	-1.309592689	0.18396574	-7.118676916	1.09E-12	9.16E-11
PITA_000064123	Low quality	AT1G75250	MA_254136g0010	shade<sun	RAD-like 6;(source:Araport11)	RAD-LIKE 6 (RL6)	DNA-binding transcription	610.7538441	-1.56410973	0.31291617	-4.998494438	5.78E-07	1.69E-05
PITA_000064168	High quality	AT5G06710	MA_194299g0010	shade>sun	Homeobox-leucine zipper prot	HOMEOBOX FROM ARABI	DNA-binding transcription	73.2243117	1.006362836	0.20279071	4.96256874	6.96E-07	2.01E-05
PITA_000064226	High quality	AT2G21090	MA_251664g0010	shade<sun	Pentatricopeptide repeat (PPR-like) superfamily protein;(s		mitochondrion-GO:00057	219.9198905	-0.676336455	0.15699173	-4.308102492	1.65E-05	0.000356766
PITA_000064250	High quality	AT4G19860	MA_102148g0010	shade<sun	Encodes a cytosolic calcium-independent phospholipase A		cytoplasm-GO:0005737,cy	178.3751186	-0.835615163	0.22551891	-3.705299742	0.000211141	0.003353258
PITA_000064296	Low quality	AT2G46690	MA_40440g0010	shade<sun	SAUR-like auxin-responsive pro	SMALL AUXIN UPREGULAT	molecular_function_unkn	216.7331623	-11.38965832	3.90699325	-2.915197851	0.003554631	0.035141859
PITA_000064299	High quality	AT4G22140	MA_14897g0020	shade>sun	Encoding a chromatin remodel	EARLY BOLTING IN SHORT	chromatin binding-GO:000	51.16191713	1.718993155	0.60260332	2.852611471	0.00433616	0.041011816
PITA_000064414	High quality	AT1G50320	MA_10433314g0010	shade<sun	encodes a prokaryotic thioredo	THIOREDOXIN X (THX)	cell redox homeostasis-GC	185.15521	-1.066315126	0.1931536	-5.520555349	3.38E-08	1.23E-06
PITA_000064471	Low quality	AT5G01880	MA_41514g0010	shade<sun	RING/U-box superfamily protei	DAF-LIKE GENE 2 (DAFL2)	protein ubiquitination-GO	241.8488841	-11.54784297	3.90696951	-2.955703374	0.00311957	0.031813807
PITA_000064481	High quality	AT5G20250	MA_18317g0010	shade>sun	encodes a member of glycosyl	DARK INDUCIBLE 10 (DIN1	cellular response to hypox	9.592196771	1.58914298	0.56527053	2.811296367	0.004934231	0.045408635
PITA_000064503	Low quality	AT1G80490	MA_10436445g0020	shade>sun	Encodes a protein with a Lisser	TOPLESS-RELATED 1 (TPR1	cytoplasm-GO:0005737,cy	125.9547677	1.24890663	0.2841319	4.395517084	1.11E-05	0.000251993
PITA_000064560	High quality	AT4G27590	MA_64950g0010	shade>sun	Heavy metal transport/detoxifi	HEAVY METAL ASSOCIATE	metal ion binding-GO:004	32.64785171	1.2905542	0.46440892	2.778917747	0.005454033	0.04857017
PITA_000064624	High quality	AT5G23190	MA_10250029g0020	shade>sun	cytochrome P450 CYP86B1, nu	CYTOCHROME P450, FAM	chloroplast-GO:0009507,e	115.0180187	1.005167087	0.28893979	3.478811555	0.000503643	0.007115892
PITA_000064642	High quality	AT3G03270	MA_10434855g0010	shade>sun	HRU1 is a hypoxia induced univ	HYPOXIA RESPONSIVE UN	cellular response to hypox	46.42696562	0.867662147	0.22866444	3.794477814	0.000147955	0.002462331
PITA_000064696	Low quality	AT1G19530	MA_79150g0010	shade>sun	DNA polymerase epsilon catalytic subunit A;(source:Arapc		anaerobic respiration-GO:	18.82576604	1.090774364	0.35977177	3.0318509	0.002430592	0.026162842
PITA_000064698	Low quality	AT3G15110	MA_837813g0010	shade<sun	transmembrane protein;(source:Araport11)		biological_process_unkno	256.2909622	-1.501169019	0.12761478	-11.76328479	6.03E-32	4.35E-29
PITA_000064758	High quality	AT3G55240	MA_47319g0010	shade>sun	Overexpression leads to PEL (Pseudo-Etiolation in Light) p		chloroplast-GO:0009507,d	10.7428496	5.734064907	1.37373586	4.174066558	2.99E-05	0.00060884
PITA_000064895	Low quality	AT1G48030	MA_4137775g0010	shade<sun	Encodes a mitochondrial lipoa	MITOCHONDRIAL LIPOAM	apoplast-GO:0048046,ATP	153.4053487	-1.01024723	0.15697841	-6.435580714	1.23E-10	6.63E-09
PITA_000065018	High quality	AT1G08510	MA_10197220g0010	shade>sun	Encodes an acyl-acyl carrier pr	FATTY ACYL-ACP THIOEST	acyl carrier activity-GO:00	67.84791435	0.562520238	0.19217442	2.927133791	0.003421017	0.034237319
PITA_000065032	Low quality	AT5G07330	MA_10337581g0010	shade>sun	NFU1 iron-sulfur cluster protein;(source:Araport11)		biological_process_unkno	47.78388957	1.147674992	0.25303132	4.535703383	5.74E-06	0.000139626
PITA_000065306	Low quality	AT1G06980	MA_10425793g0010	shade>sun	PADRE protein		biological_process_unkno	25.75997595	7.98454488	1.57544765	5.068111838	4.02E-07	1.20E-05
PITA_000065339	High quality	AT3G20330	MA_1829g0010	shade<sun	encodes aspartate carbamoyltr	PYRIMIDINE B (PYRB)	amino acid binding-GO:00	165.3897475	-0.834633323	0.23525533	-3.547776533	0.000388498	0.00569315
PITA_000065353	High quality	AT3G22970	MA_10428193g0010	shade>sun	hypothetical protein (DUF506);(source:Araport11)		biological_process_unkno	21.03485382	1.638566836	0.36946483	4.434973771	9.21E-06	0.000213647
PITA_000065355	High quality	AT5G20480	MA_161278g0010	shade<sun	Encodes a predicted leucine-ri	EF-TU RECEPTOR (EFR)	detection of bacterium-GC	191.5417464	-1.226077385	0.38533198	-3.181872865	0.00146326	0.017349989
PITA_000065409	High quality	AT1G22400	MA_55344g0010	shade<sun	UDP-Glycosyltransferase super	(UGT85A1)	cis-zeatin O-beta-D-glucos	734.8270017	-2.068598974	0.73189682	-2.826353293	0.004708131	0.043734457
PITA_000065445	High quality	AT2G38600	MA_10430401g0020	shade>sun	HAD superfamily, subfamily IIIB	acid phosphatase;(source	acid phosphatase activity-(	20.05278409	1.357920991	0.44464796	3.053923784	0.002258695	0.024765276
PITA_000065492	Low quality	AT5G08530	MA_95955g0010	shade<sun	51 kDa subunit of complex I;(sc	51 KDA SUBUNIT OF COM	FMN binding-GO:0010181	121.0904088	-0.489219791	0.14144992	-3.458607714	0.000542975	0.007584284
PITA_000065640	High quality	AT3G04300	MA_2485g0010	shade>sun	RmlC-like cupins superfamily protein;(source:Araport11)		biological_process_unkno	75.63184777	0.814920539	0.24959306	3.264996794	0.001094653	0.013606285
PITA_000065658	High quality	AT4G37510	MA_10436489g0010	shade<sun	Ribonuclease III family protein;(source:Araport11)		chloroplast-GO:0009507,d	226.551705	-1.027772554	0.12069986	-8.51510977	1.66E-17	3.16E-15
PITA_000065758	High quality	AT5G01920	MA_10428389g0010	shade>sun	Chloroplast thylakoid protein k	STATE TRANSITION 8 (STN	chloroplast-GO:0009507,n	53.46373192	9.036267593	1.44319538	6.261291925	3.82E-10	1.90E-08
PITA_000065768	High quality	AT4G37080	MA_90792g0010	shade<sun	ternary complex factor MIP1 leucine-zipper protein (Prote		biological_process_unkno	174.8263727	-0.922858492	0.14658146	-6.295874577	3.06E-10	1.55E-08
PITA_000065794	High quality	AT2G44740	MA_80306g0010	shade<sun	cyclin p4;(source:Araport11)	CYCLIN P4;1 (CYCP4;1)	protein binding-GO:00055	113.6513246	-0.60117837	0.20239848	-2.970271101	0.00297537	0.030690733
PITA_000065848	Low quality	AT5G52960	MA_10017907g0010	shade<sun	tRNA dimethylallyltransferase;(source:Araport11)		biological_process_unkno	367.873058	-1.257845081	0.14340422	-8.771325489	1.77E-18	3.77E-16
PITA_000065853	High quality	AT3G57620	MA_10437233g0020	shade<sun	glyoxal oxidase-related protein;(source:Araport11)		biological_process_unkno	1034.723676	-2.060527506	0.49733503	-4.143137659	3.43E-05	0.000684282
PITA_000065941	High quality	AT3G04870	MA_922179g0010	shade<sun	Involved in the biosynthesis of	ZETA-CAROTENE DESATUI	7,9,9'-tricis-neurosporene	101.6686165	-0.702926717	0.15195498	-4.625887908	3.73E-06	9.42E-05
PITA_000065942	High quality	AT2G46410	MA_9544g0010	shade>sun	Nuclear-localized R3-type MYB	CAPRICE (CPC)	cell differentiation-GO:00	26.33015652	1.797250407	0.53387233	3.366442338	0.000761445	0.010054263
PITA_000066016	High quality	AT4G17050	MA_485463g0010	shade<sun	Encodes a protein with ureido	UREIDOGLYCINE AMINOH	allantoin catabolic process	99.47881046	-0.533430528	0.15761575	-3.384373253	0.000713409	0.009494558
PITA_000066052	High quality	AT2G28500	MA_328535g0010	shade>sun	LOB domain-containing protei	LOB DOMAIN-CONTAININ	biological_process_unkno	302.449864	11.53658875	3.90688853	2.952884035	0.003148202	0.032017345
PITA_000066241	High quality	AT1G19835	MA_10429332g0010	shade<sun	TCS1 encodes a coiled-coil don	TRICHOME CELL SHAPE 1 (	cortical microtubule-GO:0	302.255526	-11.86951709	3.90692858	-3.038068615	0.002380997	0.025716811
PITA_000066316	High quality	AT5G62850	MA_118539g0010	shade>sun	Encodes a protein that is expre	VEGETATIVE CELL EXPRES	carbohydrate transport-G(	22.23767181	2.08515137	0.40170392	5.190766808	2.09E-07	6.54E-06
PITA_000066404	High quality	AT1G53670	MA_10433652g0010	shade<sun	methionine sulfoxide reductas	METHIONINE SULFOXIDE	chloroplast-GO:0009507,c	122.5411477	-0.562106449	0.17220193	-3.264228583	0.001097626	0.013629798
PITA_000066468	Low quality	AT4G02920	MA_10430693g0010	shade<sun	hypothetical protein;(source:Araport11)		biological_process_unkno	160.1263165	-0.588202728	0.16315205	-3.605242549	0.000311862	0.004712491
PITA_000066650	High quality	AT5G51010	MA_10432860g0010	shade<sun	Rubredoxin-like superfamily protein;(source:Araport11)		chloroplast-GO:0009507,ir	488.4547705	-1.380806541	0.20186967	-6.840089189	7.91E-12	5.51E-10
PITA_000066684	High quality	AT3G03070	MA_7862g0010	shade<sun	NADH-ubiquinone oxidoreductase-like protein;(source:Ar		mitochondrial electron tra	113.6007927	-0.603618183	0.16185724	-3.729324692	0.000191994	0.003086093
PITA_000066922	High quality	AT5G36930	MA_10024934g0010	shade>sun	Disease resistance protein (TIR-NBS-LRR class) family;(sou		ADP binding-GO:0043531,	77.41072554	1.432036359	0.46375328	3.087927157	0.002015579	0.022612498
PITA_000066947	High quality	AT4G31850	MA_103898g0010	shade>sun	encodes a protein containing 2	PROTON GRADIENT REGU	mRNA binding-GO:000372	62.50774448	0.941560099	0.22561208	4.17335852	3.00E-05	0.000609958
PITA_000066986	Low quality	AT5G12080	MA_10428116g0010	shade<sun	Encodes a mechanosensitive (c	MECHANOSENSITIVE CHA	anion transport-GO:00068	203.3334412	-1.328141234	0.24888938	-5.336271227	9.49E-08	3.17E-06
PITA_000067002	High quality	AT2G36780	MA_10426944g0020	shade<sun	UDP-Glycosyltransferase superfamily protein;(source:Arap		chloroplast-GO:0009507,t	386.9445101	-1.970888439	0.29349148	-6.715317429	1.88E-11	1.20E-09
PITA_000067048	Low quality	AT1G72200	MA_10436208g0010	shade>sun	RING/U-box superfamily protei	ARABIDOPSIS T??XICOS EI	mitochondrion-GO:00057	22.73149503	3.225013923	0.49651559	6.495292318	8.29E-11	4.61E-09
PITA_000067262	High quality	AT5G52010	MA_33034g0010	shade<sun	C2H2-like zinc finger protein;(source:Araport11)		chloroplast-GO:0009507,D	237.7289615	-0.889092378	0.10472582	-8.489715248	2.07E-17	3.81E-15
PITA_000067268	High quality	AT1G56120	MA_954053g0010	shade<sun	Leucine-rich repeat transmembrane protein kinase;(sourc		kinase activity-GO:001630	107.2558707	-0.451720972	0.15347307	-2.943324092	0.003247083	0.03286173
PITA_000067486	High quality	AT5G57170	MA_449338g0010	shade<sun	Tautomerase/MIF superfamily protein;(source:Araport11)		chloroplast-GO:0009507,e	175.0934047	-0.707400879	0.17006948	-4.159481514	3.19E-05	0.000642528
PITA_000067494	High quality	AT1G56170	MA_33970g0010	shade>sun	Encodes a protein with similari	NUCLEAR FACTOR Y, SUBU	CCAAT-binding factor com	332.6558265	11.67405373	2.27036176	5.141935495	2.72E-07	8.36E-06
PITA_000067504	High quality	AT1G18010	MA_140137g0010	shade<sun	Major facilitator superfamily protein;(source:Araport11)		molecular_function_unkn	152.0264608	-1.945255977	0.30081106	-6.466703625	1.00E-10	5.50E-09
PITA_000067510	High quality	AT5G61190	MA_56313g0010	shade>sun	putative endonuclease or glycosyl hydrolase with C2H2-ty		chloroplast-GO:0009507,n	43.81560443	0.67229972	0.22833813	2.944316521	0.003236688	0.032769679
PITA_000067679	Low quality	AT3G56290	MA_55006g0010	shade<sun	potassium transporter;(source:Araport11)		chloroplast organization-G	1585.150985	-2.163663146	0.75328722	-2.872295044	0.004075023	0.03903567
PITA_000067719	High quality	AT1G64970	MA_53835g0010	shade<sun	gamma-tocopherol methyltran	GAMMA-TOCOPHEROL M	chloroplast-GO:0009507,m	220.3935414	-0.815308817	0.15084316	-5.405010074	6.48E-08	2.26E-06
PITA_000067721	High quality	AT1G15500	MA_10428243g0010	shade<sun	TLC ATP/ADP transporter;(sou	(ATNTT2)	ATP:ADP antiporter activit	165.7801114	-0.866551002	0.21123302	-4.102346308	4.09E-05	0.000799575
PITA_000067829	High quality	AT1G59700	MA_57437g0010	shade>sun	Encodes glutathione transfera	GLUTATHIONE S-TRANSFE	cytoplasm-GO:0005737,cy	41.43840294	1.046813509	0.24651496	4.246450248	2.17E-05	0.000456829
PITA_000067857	High quality	AT2G38940	MA_366260g0010	shade<sun	Encodes Pht1;4, a member of t	PHOSPHATE TRANSPORTE	cytoplasm-GO:0005737,Go	56.70237037	-2.431034304	0.82839192	-2.934642715	0.00333932	0.033552844
PITA_000067916	High quality	AT5G51110	MA_167002g0010	shade<sun	Encodes a protein involved in I	SDIR1-INTERACTING PROT	4-alpha-hydroxytetrahydr	741.8225839	-1.257956795	0.17750612	-7.086835988	1.37E-12	1.12E-10
PITA_000067926	High quality	AT5G05340	MA_10432379g0020	shade>sun	Encodes a protein with sequen	PEROXIDASE 52 (PRX52)	apoplast-GO:0048046,cell	272.5133598	2.317475603	0.7107484	3.260613179	0.001111716	0.013784403
PITA_000068029	High quality	AT5G06839	MA_130907g0010	shade>sun	bZIP transcription factor family	TGACG (TGA) MOTIF-BIND	anther development-GO:0	27.16459797	0.892991732	0.29545776	3.022400644	0.002507784	0.026817051
PITA_000068060	Low quality	AT1G51420	MA_10436487g0010	shade<sun	sucrose-phosphatase 1;(source	SUCROSE-PHOSPHATASE	magnesium ion binding-G(	487.0357435	-1.76902	0.30301507	-5.838059496	5.28E-09	2.17E-07
PITA_000068091	Low quality	AT4G38160	MA_453913g0010	shade<sun	Encodes a member of the mitc	PIGMENT DEFECTIVE 191	chloroplast-GO:0009507,c	311.1384294	-0.507121192	0.15284107	-3.317964203	0.000906761	0.011643846
PITA_000068207	High quality	AT2G39050	MA_10435501g0030	shade>sun	Encodes a nucleocytoplasmic l	EUONYMUS LECTIN S3 (EL	carbohydrate binding-GO:	44.61024146	1.887311325	0.34453964	5.477776995	4.31E-08	1.54E-06
PITA_000068234	Low quality	AT5G11090	MA_10432433g0020	shade>sun	serine-rich protein-like protein;(source:Araport11)		biological_process_unkno	22.29274017	1.84115324	0.46884022	3.927037721	8.60E-05	0.001530615
PITA_000068288	High quality	AT3G14420	MA_15820g0010	shade<sun	Encodes a glycolate oxidase th	GLYCOLATE OXIDASE 1 (G	apoplast-GO:0048046,chlc	477.8944655	-1.20575743	0.42482891	-2.838218886	0.004536606	0.042406673
PITA_000068540	High quality	AT3G08490	MA_10227167g0010	shade<sun	delta-latroinsectotoxin-Lt1a protein;(source:Araport11)		biological_process_unkno	209.9534673	-0.847442019	0.20104811	-4.215120564	2.50E-05	0.000519904
PITA_000068560	High quality	AT1G05675	MA_10433477g0020	shade>sun	UDP-Glycosyltransferase superfamily protein;(source:Arap		quercetin 3-O-glucosyltrar	12.41203683	1.262003605	0.44319026	2.847543638	0.004405805	0.041507064
PITA_000068610	High quality	AT1G18170	MA_10436268g0010	shade<sun	FKBP-like peptidyl-prolyl cis-trans isomerase family protei		chloroplast-GO:0009507,c	1013.010955	-1.117245674	0.14693443	-7.603702365	2.88E-14	3.09E-12
PITA_000068628	High quality	AT1G69550	MA_10146548g0020	shade<sun	disease resistance protein (TIR-NBS-LRR class);(source:Ara		ADP binding-GO:0043531,	121.0157992	-0.854511998	0.30743064	-2.779527755	0.0054438	0.048496164
PITA_000068685	High quality	AT2G40460	MA_89317g0010	shade<sun	Major facilitator superfamily protein;(source:Araport11)		response to nematode-GC	103.9725139	-1.30341499	0.24372287	-5.347938802	8.90E-08	2.98E-06

PITA_000068799	High quality	AT1G52240	MA_10199247g0010	shade<sun	dynein light chain flagellar outer arm-like		protein binding-GO:00055	112.0113053	-0.688493436	0.22347425	-3.080862549	0.002064019	0.02302496
PITA_000068921	Low quality	AT5G04550	MA_55500g0010	shade>sun	type-1 restriction enzyme mjaxp r protein (DUF668);(sour		molecular_function_unkn	48.26219871	0.704227487	0.24354802	2.891534469	0.003833655	0.03730123
PITA_000068957	High quality	AT4G38620	MA_2026g0010	shade>sun	Encodes a R2R3 MYB protein w	MYB DOMAIN PROTEIN 4	regulation of phenylpropa	55.03472299	9.077999543	1.71794632	5.284216063	1.26E-07	4.13E-06
PITA_000069214	Low quality	AT1G74930	MA_10062104g0010	shade>sun	encodes a member of the DREI	(ORA47)	cell division-GO:0051301,c	33.02165172	8.33945414	1.20294584	6.932526693	4.13E-12	3.01E-10
PITA_000069232	High quality	AT5G49800	MA_952002g0010	shade>sun	Polyketide cyclase/dehydrase and lipid transport superfar		lipid binding-GO:0008289,	14.58878964	2.092791318	0.54360256	3.849855527	0.000118188	0.002016704
PITA_000069238	High quality	AT3G15200	MA_10432868g0030	shade<sun	Tetratricopeptide repeat (TPR)-like superfamily protein;(s		biological_process_unknov	130.9191394	-0.57044614	0.18900637	-3.018131894	0.002543382	0.02709932
PITA_000069287	High quality	AT3G50120	MA_10426384g0020	shade<sun	transmembrane protein, putative (DUF247);(source:Arapc		biological_process_unknov	605.4177495	-0.967886457	0.3373316	-2.869243385	0.004114551	0.039351143
PITA_000069506	High quality	AT2G38110	MA_10429049g0010	shade>sun	bifunctional sn-glycerol-3-phos	GLYCEROL-3-PHOSPHATE	cutin biosynthetic process	63.56138644	0.788095756	0.27302453	2.886538324	0.003895053	0.037695026
PITA_000069863	High quality	AT5G01810	MA_5676077g0010	shade>sun	Encodes a CBL-interacting serir	CBL-INTERACTING PROTEI	abscisic acid-activated sigr	83.57783164	1.304638098	0.28582782	4.564419559	5.01E-06	0.000123845
PITA_000069864	High quality	AT2G01570	MA_10430831g0010	shade>sun	Member of the VHIID/DELLA re	REPRESSOR OF GA1-3 1 (R	DNA-binding transcription	88.59625944	1.261957737	0.43283866	2.915538405	0.003550754	0.035131431
PITA_000069884	Low quality	AT1G19250	MA_10430096g0010	shade<sun	FMO1 is required for full expre	FLAVIN-DEPENDENT MON	cellular response to hypox	1963.379434	-1.006201114	0.34757223	-2.894941022	0.003792298	0.03702739
PITA_000069888	High quality	AT2G46950	MA_454299g0010	shade>sun	cytochrome P450, family 709, s	CYTOCHROME P450, FAM	chloroplast-GO:0009507,c	87.03602065	2.075235913	0.46368252	4.475553494	7.62E-06	0.000180989
PITA_000069901	Low quality	AT5G44460	MA_9257733g0010	shade<sun	calmodulin like 43;(source:Ara	CALMODULIN LIKE 43 (CM	calcium ion binding-GO:00	350.2154652	-12.08196511	3.90690614	-3.092463624	0.001985026	0.022349261
PITA_000070150	High quality	AT1G63970	MA_60257g0010	shade<sun	Encodes a protein with 2C-met	ISOPRENOID F (ISPF)	2-C-methyl-D-erythritol 2,4	155.1280563	-0.709120963	0.13372031	-5.303015992	1.14E-07	3.76E-06
PITA_000070216	Low quality	AT1G26090	MA_10432114g0020	shade<sun	P-loop containing nucleoside triphosphate hydrolases sup		chloroplast-GO:0009507,p	200.663916	-0.792375168	0.23050562	-3.437552539	0.000586997	0.008094073
PITA_000070401	High quality	AT1G29520	MA_10428394g0010	shade>sun	AWPM-19-like family protein;(source:Araport11)		biological_process_unknov	29.87761457	1.217541916	0.29834474	4.080990071	4.48E-05	0.00086931
PITA_000070498	High quality	AT5G17330	MA_442130g0010	shade<sun	Encodes one of two isoforms o	GLUTAMATE DECARBOXYI	calmodulin binding-GO:00	292.8658593	-0.538250662	0.13020835	-4.13376459	3.57E-05	0.000706455
PITA_000070525	Low quality	AT5G06490	MA_117647g0010	shade>sun	RING/U-box superfamily protei	ARABIDOPSIS T??XICOS EI	nucleus-GO:0005634,prote	34.75288309	1.86589908	0.39852866	4.681969666	2.84E-06	7.31E-05
PITA_000070592	Low quality	AT1G58400	MA_777544g0010	shade<sun	Disease resistance protein (CC-NBS-LRR class) family;(sour		ADP binding-GO:0043531	116.8481854	-10.49787186	1.50572705	-6.971962058	3.13E-12	2.37E-10
PITA_000070666	Low quality	AT3G61520	MA_105179g0010	shade<sun	Pentatricopeptide repeat (PPR) superfamily protein;(sour		biological_process_unknov	117.7093402	-0.645453229	0.22079027	-2.923377178	0.003462568	0.03452984
PITA_000070707	High quality	AT2G39220	MA_83822g0010	shade<sun	Phospholipase pPLAIIIa involve	PATATIN-LIKE PROTEIN 6 (	hydrolase activity-GO:001	471.8431435	-1.862636664	0.65616544	-2.838669275	0.004530208	0.042362571
PITA_000070824	High quality	AT3G45970	MA_467939g0010	shade>sun	member of EXPANSIN-LIKE. Na	EXPANSIN-LIKE A1 (EXLA1	cell wall-GO:0005618,extra	36.75131727	1.434980093	0.44474364	3.226533135	0.001252997	0.015265986
PITA_000070862	Low quality	AT5G11900	MA_19414g0010	shade>sun	Translation initiation factor SUI1 family protein;(source:Ar		cytoplasm-GO:0005737,fo	110.9888725	10.08977412	1.24061293	8.132894513	4.19E-16	6.33E-14
PITA_000070882	High quality	AT1G12900	MA_63231g0010	shade<sun	glyceraldehyde 3-phosphate de	GLYCERALDEHYDE 3-PHOS	apoplast-GO:0048046,chlo	2846.75187	-1.861745061	0.31852978	-5.844806848	5.07E-09	2.09E-07
PITA_000070884	High quality	AT5G18260	MA_10591g0010	shade<sun	RING/U-box superfamily protein;(source:Araport11)		nucleus-GO:0005634,prote	60.31537316	-9.545388389	1.54175647	-6.191242615	5.97E-10	2.86E-08
PITA_000070901	High quality	AT3G60720	MA_10429143g0010	shade>sun	Encodes a plasmodesmal prote	PLASMODESMATA-LOCAT	molecular_function_unkn	4.475105704	3.511348204	1.25386427	2.800421304	0.005103595	0.046366234
PITA_000070940	High quality	AT5G43360	MA_10434456g0010	shade<sun	Encodes Pht1;3, a member of t	PHOSPHATE TRANSPORTE	cytoplasm-GO:0005737,in	3610.077064	-1.851092723	0.65458317	-2.827895391	0.004685511	0.043588565
PITA_000071020	Low quality	AT4G38900	MA_161658g0010	shade>sun	Basic-leucine zipper (bZIP) tran	BASIC LEUCINE-ZIPPER 29	cytoplasm-GO:0005737,cy	55.37920375	1.462581796	0.51597008	2.834625186	0.004587948	0.042791437
PITA_000071107	High quality	AT4G19420	MA_10432685g0010	shade<sun	Pectinacetylesterase family prc	PECTIN ACETYLESTERASE	cell wall organization-GO:	143.026457	-2.873912118	0.97279245	-2.954291154	0.003133882	0.03193394
PITA_000071189	High quality	AT3G02750	MA_958274g0010	shade<sun	Protein phosphatase 2C family protein;(source:Araport11)		chloroplast-GO:0009507,c	118.485568	-0.602524285	0.14705245	-4.097342812	4.18E-05	0.000813895
PITA_000071297	Low quality	AT2G18650	MA_229590g0010	shade>sun	RING/U-box superfamily protei	MATERNAL EFFECT EMBR'	embryo development end	43.32283612	1.18266322	0.2967362	3.985571097	6.73E-05	0.001233837
PITA_000071451	High quality	AT1G72310	MA_904294g0010	shade>sun	Encodes a putative RING-H2 zi	(ATL3)	nucleus-GO:0005634,prote	29.22725907	3.389022598	0.51884054	6.531915615	6.49E-11	3.72E-09
PITA_000071600	High quality	AT1G58340	MA_170275g0010	shade<sun	Encodes a plant MATE (multidi	(ZF14)	antiporter activity-GO:001	864.2661593	-1.413620336	0.42597219	-3.318574242	0.000904783	0.01162436
PITA_000071608	Low quality	AT2G41890	MA_208216g0010	shade<sun	curculin-like (mannose-binding) lectin family protein / PAI		calmodulin binding-GO:00	204.9820773	-0.78359588	0.19697879	-3.978072422	6.95E-05	0.001265119
PITA_000071761	Low quality	AT4G20140	MA_87079g0010	shade>sun	Encodes GASSHO1 (GSO1), a p	GASSHO1 (GSO1)	Casparian strip-GO:004822	163.3565924	10.64796737	1.44701707	7.358563785	1.86E-13	1.74E-11
PITA_000071813	Low quality	AT1G46264	MA_110587g0010	shade>sun	Encodes SCHIZORIZA, a membe	HEAT SHOCK TRANSCRIPT	asymmetric cell division-G	39.99823109	1.361717437	0.44493621	3.060477869	0.002209841	0.024305359
PITA_000072089	Low quality	AT3G44200	MA_10430199g0010	shade<sun	Encodes AtNek5, a member of	NIMA (NEVER IN MITOSIS,	cortical microtubule-GO:0	269.8462647	-0.431838364	0.14594949	-2.95882058	0.003088188	0.031595989
PITA_000072200	High quality	AT3G61320	MA_495688g0010	shade>sun	Encodes a bestrophin-like prot	BESTROPHIN-LIKE PROTEI	chloroplast-GO:0009507,c	76.86638708	1.395066112	0.41178968	3.387812242	0.000704525	0.009396147
PITA_000072253	High quality	AT1G24030	MA_9033g0010	shade>sun	Protein kinase superfamily pro	PBS1-LIKE 28 (PBL28)	ATP binding-GO:0005524,i	135.896427	0.656113884	0.15340896	4.276894089	1.90E-05	0.000405744
PITA_000072274	High quality	AT3G05060	MA_513089g0010	shade<sun	SAR DNA-binding protein, putative, strong similarity to SA		box C/D RNP complex-GO:	107.1298166	-1.06315325	0.18974658	-5.603016675	2.11E-08	7.91E-07
PITA_000072312	High quality	AT4G40042	MA_420602g0010	shade>sun	Microsomal signal peptidase 12 kDa subunit (SPC12);(sour		integral component of enc	40.74804669	0.828114345	0.25459398	3.252686248	0.001143196	0.014126122
PITA_000072503	High quality	AT1G76690	MA_592495g0010	shade<sun	Encodes one of the closely rela	12-OXOPHYTODIENOATE	12-oxophytodienoate redu	213.072946	-0.97447864	0.26168736	-3.723827639	0.000196225	0.003142911
PITA_000072521	High quality	AT2G15790	MA_184831g0020	shade<sun	SQN encodes the Arabidopsis I	SQUINT (SQN)	cyclosporin A binding-GO:	248.4084606	-0.742072891	0.15366966	-4.829013705	1.37E-06	3.76E-05
PITA_000072552	High quality	AT1G01630	MA_10436099g0010	shade<sun	Sec14p-like phosphatidylinositol transfer family protein;(s		nucleus-GO:0005634	364.7581479	-0.862052291	0.28563899	-3.017978379	0.002544671	0.027101601
PITA_000072599	High quality	AT3G19810	MA_466704g0010	shade<sun	BTB/POZ domain protein, puta	DOMAIN OF UNKNOWN F	chloroplast-GO:0009507,m	295.6165669	-0.986574276	0.18091182	-5.45334352	4.94E-08	1.75E-06
PITA_000072608	Low quality	AT4G15480	MA_195838g0010	shade<sun	Encodes a protein that might h	(UGT84A1)	cinnamate beta-D-glucosy	632.3137181	-1.921300214	0.3935984	-4.881372003	1.05E-06	2.95E-05
PITA_000072849	High quality	AT3G27810	MA_10320g0010	shade>sun	Encodes a member of the R2R3	MYB DOMAIN PROTEIN 2	gibberellic acid mediated s	60.16852827	9.206815854	1.29589282	7.104612141	1.21E-12	1.00E-10
PITA_000072961	High quality	AT4G13260	MA_31349g0010	shade>sun	Encodes YUC2. Catalyzes conve	YUCCA2 (YUC2)	auxin biosynthetic process	77.52217015	1.394001174	0.31852707	4.376397772	1.21E-05	0.000271936
PITA_000072997	High quality	AT2G28000	MA_10436772g0010	shade<sun	Encodes chaperonin-60 alpha,	CHAPERONIN-60ALPHA (C	apoplast-GO:0048046,chlo	255.8477819	-0.885909979	0.18832262	-4.70421436	2.55E-06	6.64E-05
PITA_000073010	Low quality	AT4G09520	MA_874923g0010	shade<sun	Cofactor-independent phosphoglycerate mutase;(source:/		2,3-bisphosphoglycerate-i	129.5920141	-1.008129783	0.31110377	-3.24049361	0.001193229	0.014615467
PITA_000073314	Low quality	AT4G37420	MA_10973g0010	shade<sun	glycosyltransferase family protein (DUF23);(source:Arapor		biological_process_unknov	106.3956735	-1.772906837	0.50311714	-3.52384502	0.000425333	0.006140181
PITA_000073422	High quality	AT4G03420	MA_10431340g0010	shade>sun	hypothetical protein (DUF789);(source:Araport11)		biological_process_unknov	42.81810012	0.749668041	0.23393613	3.204584215	0.001352577	0.016282575
PITA_000073446	High quality	AT2G37130	MA_10432809g0010	shade>sun	Peroxidase superfamily protein;(source:Araport11)		cytosol-GO:0005829,defer	5.271832446	2.610356317	0.86641456	3.01282601	0.002588273	0.02742699
PITA_000073489	Low quality	AT1G72630	MA_866497g0010	shade>sun	ELF4-like 2;(source:Araport11)	ELF4-LIKE 2 (ELF4-L2)	entrainment of circadian c	126.3383214	0.944554601	0.19902969	4.745797494	2.08E-06	5.49E-05
PITA_000073607	High quality	AT3G09930	MA_5971026g0020	shade<sun	GDSL-motif esterase/acyltransferase/lipase. Enzyme grou		hydrolase activity, acting c	168.8857484	-1.078683376	0.3415531	-3.158171855	0.001587619	0.01863181
PITA_000073609	High quality	AT2G38820	MA_15472g0010	shade>sun	DNA-directed RNA polymerase subunit beta-beta protein,		biological_process_unknov	249.7530574	11.26040195	1.47893172	7.613875476	2.66E-14	2.90E-12
PITA_000073858	High quality	AT5G18840	MA_10430268g0010	shade>sun	Major facilitator superfamily protein;(source:Araport11)		carbohydrate transmembr	27.86963936	1.121422326	0.29530564	3.79749719	0.000146164	0.002436396
PITA_000074055	High quality	AT1G75220	MA_10239544g0010	shade<sun	Encodes a vacuolar glucose exp	ERD6-LIKE 6 (ERDL6)	carbohydrate transmembr	171.063208	-1.208148678	0.27856771	-4.337001827	1.44E-05	0.000317868
PITA_000074149	Low quality	AT3G61460	MA_169947g0010	shade>sun	Encodes a novel ring finger prc	BRASSINOSTEROID-RESPC	brassinosteroid mediated	45.88194405	0.966481085	0.31562688	3.062100091	0.002197899	0.024226846
PITA_000074157	High quality	AT3G52930	MA_101067g0010	shade<sun	Aldolase superfamily protein;(s	FRUCTOSE-BISPHOSPHATI	apoplast-GO:0048046,cell	406.2874815	-1.169555007	0.13881797	-8.425097783	3.60E-17	6.40E-15
PITA_000074183	High quality	AT4G04320	MA_903555g0010	shade<sun	malonyl-CoA decarboxylase family protein;(source:Arapor		acetyl-CoA biosynthetic pr	81.37988017	-1.19515986	0.31688615	-3.77157489	0.00016222	0.002665277
PITA_000074297	High quality	AT3G12580	MA_10436544g0020	shade<sun	heat shock protein 70;(source:	HEAT SHOCK PROTEIN 70	ATP binding-GO:0005524,	134.0590644	-0.499388797	0.17978948	-2.777630731	0.005475681	0.048694171
PITA_000074400	High quality	AT3G03990	MA_9657824g0010	shade<sun	Encodes an alpha/beta hydrola	DWARF 14 (D14)	cellular response to strigo	717.6180473	-1.908030297	0.15655657	-12.18748185	3.62E-34	3.05E-31
PITA_000074460	High quality	AT1G80480	MA_174309g0010	shade<sun	plastid transcriptionally active	PLASTID TRANSCRIPTIONA	chloroplast-GO:0009507,c	250.7400141	-0.583893255	0.21050594	-2.773761468	0.005541229	0.049069452
PITA_000074587	High quality	AT4G25850	MA_82319g0010	shade>sun	OSBP(oxysterol binding protei	OSBP(OXYSTEROL BINDIN	cytosol-GO:0005829,intrac	40.5594214	1.500763758	0.28725884	5.224430276	1.75E-07	5.51E-06
PITA_000074609	High quality	AT4G35450	MA_10427522g0010	shade<sun	A molecular chaperone that bi	ANKYRIN REPEAT-CONTAI	cellular response to cold-G	177.407375	-0.675123101	0.19327132	-3.493136404	0.000477383	0.006771422
PITA_000074722	High quality	AT1G02170	MA_183122g0010	shade>sun	Encodes a type I metacaspase.	METACASPASE 1 (MC1)	cysteine-type endopeptida	55.29676349	9.085190826	1.52206115	5.969005125	2.39E-09	1.04E-07
PITA_000074736	Low quality	AT1G49760	MA_10436565g0010	shade>sun	polyadenylate-binding protein,	POLY(A) BINDING PROTEII	cytoplasm-GO:0005737,cy	61.4972516	1.12508621	0.36021222	3.123398258	0.001787757	0.020438451
PITA_000075032	High quality	AT2G47520	MA_96512g0020	shade>sun	encodes a member of the ERF	ETHYLENE RESPONSE FAC	cellular response to hypox	235.950183	1.979313982	0.51121077	3.871815878	0.000108028	0.001867344
PITA_000075150	Low quality	AT3G29185	MA_31719g0010	shade<sun	Encodes a chloroplast protein t	BIOGENESIS FACTOR REQ	cell division-GO:0051301,c	414.1125977	-1.219286723	0.18820424	-6.47852956	9.26E-11	5.12E-09
PITA_000075157	High quality	AT5G18980	MA_10428851g0010	shade<sun	ARM repeat superfamily protein;(source:Araport11)		biological_process_unknov	628.7735418	-12.92622189	3.90684347	-3.308610129	0.000937603	0.011960722
PITA_000075235	Low quality	AT2G46750	MA_851678g0010	shade>sun	Encodes a homolog of rat L-gul	L -GULONO-1,4-LACTONE	D-arabinono-1,4-lactone o	92.34399661	0.894009554	0.26226935	3.408745798	0.000652623	0.008830012
PITA_000075240	High quality	AT1G20560	MA_10436294g0010	shade<sun	acyl activating enzyme 1;(sourc	ACYL ACTIVATING ENZYM	AMP binding-GO:0016208	481.9920384	-1.108730734	0.28519672	-3.887599911	0.00010124	0.001766976
PITA_000075320	Low quality	AT1G08070	MA_65500g0010	shade<sun	Encodes a chloroplast RNA edi	ORGANELLE TRANSCRIPT I	chloroplast-GO:0009507,c	258.4708382	-1.423182636	0.22068068	-6.449058701	1.13E-10	6.09E-09
PITA_000075356	High quality	AT5G45390	MA_140852g0010	shade<sun	One of several nuclear-encode	CLP PROTEASE P4 (CLPP4)	ATPase binding-GO:00511	415.7765881	-0.994638578	0.22826402	-4.357404115	1.32E-05	0.000292708
PITA_000075371	Low quality	AT1G49230	MA_10426834g0010	shade>sun	RING/U-box superfamily protei	ARABIDOPSIS TOXICOS EN	protein ubiquitination-GO	24.79002153	7.926524471	1.50237805	5.275985278	1.32E-07	4.30E-06
PITA_000075507	High quality	AT3G44620	MA_10429270g0010	shade<sun	protein-tyrosine phosphatase;(source:Araport11)		acid phosphatase activity-(	237.1616186	-1.405330045	0.38096412	-3.688877713	0.000225245	0.003543787
PITA_000075649	High quality	AT5G40380	MA_27101g0010	shade<sun	Encodes a cysteine-rich recept	CYSTEINE-RICH RLK (RECEI	chloroplast-GO:0009507,k	190.6462339	-11.20457371	3.90702453	-2.867802243	0.004133338	0.039485903
PITA_000075797	Low quality	AT4G31390	MA_10436955g0010	shade<sun	Protein kinase superfamily pro	ABC1-LIKE KINASE RELATE	ATP binding-GO:0005524,	618.7163568	-1.439015602	0.3072258	-4.683902134	2.81E-06	7.25E-05
PITA_000075902	High quality	AT1G05577	MA_3651g0010	shade<sun	SOK1 is a DUF966 domain con	SOSEKI1 (SOK1)	apical plasma membrane-(	108.5952152	-1.374329225	0.38519209	-3.567906133	0.000359845	0.005347555
PITA_000076047	High quality	AT4G00620	MA_112201g0010	shade<sun	Amino acid dehydrogenase fan	EMBRYO DEFECTIVE 3127	chloroplast-GO:0009507,c	109.9056165	-1.392796227	0.2465781	-5.648499409	1.62E-08	6.16E-07
PITA_000076088	Low quality	AT1G65440	MA_13648g0010	shade>sun	Related to yeast Spt6 protein,	GLOBAL TRANSCRIPTION I	chromatin assembly or dis	44.85519193	0.705964639	0.2321054	3.04156927	0.002353484	0.025550955
PITA_000076156	High quality	AT4G25810	MA_10430703g0010	shade>sun	xyloglucan endotransglycosylas	XYLOGLUCAN ENDOTRAN	cell wall-GO:0005618,cell	17.92608298	2.361746072	0.53576573	4.408169323	1.04E-05	0.000239448
PITA_000076215	Low quality	AT1G76080	MA_99302g0010	shade<sun	Encodes a thioredoxin like pro	CHLOROPLASTIC DROUGH	cell redox homeostasis-GC	888.7438415	-1.788372474	0.13211125	-13.53686756	9.47E-42	1.71E-38
PITA_000076240	High quality	AT3G25410	MA_100722g0010	shade<sun	Sodium Bile acid symporter family;(source:Araport11)		chloroplast-GO:0009507,c	473.3879476	-0.866611602	0.10624194	-8.156962935	3.44E-16	5.42E-14
PITA_000076317	High quality	AT4G27230	MA_10434390g0020	shade>sun	Encodes HTA2, a histone H2A p	HISTONE H2A 2 (HTA2)	chromatin silencing-GO:00	35.79024349	0.827090125	0.2845402	2.906760155	0.003651931	0.035907094
PITA_000076354	Low quality	AT3G06980	MA_10437125g0010	shade<sun	DEA(D/H)-box RNA helicase family protein;(source:Arapor		cellular defense response-	408.7580879	-1.296630725	0.22908025	-5.660159286	1.51E-08	5.77E-07
PITA_000076456	Low quality	AT4G09340	MA_169781g0010	shade>sun	SPla/RYanodine receptor (SPRY) domain-containing prote		cell surface receptor signa	222.7684361	11.09543652	3.90693476	2.839933913	0.004512288	0.042247406
PITA_000076457	High quality	AT5G16080	MA_893141g0010	shade>sun	carboxyesterase 17;(source:Ar	CARBOXYESTERASE 17 (C	cellular_component_unkn	148.2321635	0.905577733	0.307763	2.942451613	0.003256247	0.032941252
PITA_000076462	Low quality	AT5G15600	MA_110382g0010	shade<sun	SPIRAL1-LIKE4 belongs to a six-	SPIRAL1-LIKE4 (SP1L4)	cortical microtubule organ	187.246721	-1.019829702	0.35023641	-2.911832313	0.003593155	0.035439719
PITA_000076601	High quality	AT5G55050	MA_119222g0010	shade>sun	GDSL-motif esterase/acyltransferase/lipase. Enzyme grou		chloroplast-GO:0009507,li	38.62941007	1.115474401	0.30376205	3.67219802	0.000240473	0.00375758
PITA_000076610	High quality	AT5G03870	MA_48594g0010	shade>sun	Glutaredoxin family protein;(source:Araport11)		nucleus-GO:0005634,plasr	136.7971554	0.410107917	0.1372424	2.988201308	0.002806246	0.029378803
PITA_000076617	High quality	AT1G79620	MA_15546g0010	shade<sun	VRLK1 is a LRR kinase involved	VASCULAR-RELATED RLK 1	ATP binding-GO:0005524,l	966.9035314	-0.845473161	0.30531145	-2.769215381	0.005619148	0.04958534
PITA_000076699	High quality	AT1G33290	MA_93106g0010	shade<sun	P-loop containing nucleoside triphosphate hydrolases sup		ATP binding-GO:0005524,	594.2630205	-0.792533912	0.27318026	-2.901139049	0.003718089	0.036486047
PITA_000076748	High quality	AT1G68760	MA_11165g0010	shade<sun	Encodes a cytosol-localized nu	NUDIX HYDROLASE 1 (NU	8-oxo-7,8-dihydrodeoxygu	140.089477	-2.362028481	0.72672455	-3.25023901	0.001153081	0.014220387
PITA_000076754	Low quality	AT3G58500	MA_10427909g0010	shade>sun	Encodes one of the isoforms o	PROTEIN PHOSPHATASE 2	cytoplasm-GO:0005737,cy	14.73896922	4.258294843	0.78835228	5.401512657	6.61E-08	2.30E-06
PITA_000076762	High quality	AT1G28650	MA_10376648g0010	shade<sun	GDSL-motif esterase/acyltransferase/lipase. Enzyme grou		extracellular region-GO:00	142.2528622	-10.78178455	1.36391033	-7.905053801	2.68E-15	3.50E-13
PITA_000077043	High quality	AT5G39980	MA_112272g0010	shade<sun	PDM3 belongs to the P subfam	EMBRYO LETHAL 3140 (EN	chloroplast-GO:0009507,c	239.7230659	-2.057684256	0.68434452	-3.006795824	0.00264017	0.027859872
PITA_000077046	High quality	AT1G12220	MA_44026g0010	shade>sun	Resistance gene, mediates resi	RESISTANT TO P. SYRINGA	ADP binding-GO:0043531,	144.6100061	0.941330673	0.32843359	2.866121776	0.004155344	0.039651066
PITA_000077173	High quality	AT2G28490	MA_286762g0010	shade<sun	RmlC-like cupins superfamily protein;(source:Araport11)		biological_process_unknov	29.18671001	-8.497475933	1.62920905	-5.215706321	1.83E-07	5.73E-06
PITA_000077247	Low quality	AT5G56940	MA_4773g0010	shade>sun	Ribosomal protein S16 family protein;(source:Araport11)		mitochondrion-GO:00057	71.92793359	0.970133566	0.30890819	3.140523972	0.001686459	0.019465674
PITA_000077283	High quality	AT5G65550	MA_36867g0010	shade<sun	UDP-Glycosyltransferase superfamily protein;(source:Arap		chloroplast-GO:0009507,i	1177.757768	-4.165587618	0.68576431	-6.074372161	1.24E-09	5.70E-08
PITA_000077318	High quality	AT2G45510	MA_169256g0010	shade<sun	member of CYP704A	CYTOCHROME P450, FAM	endoplasmic reticulum-GC	151.127763	-10.86956508	3.90709245	-2.782008675	0.00540236	0.048169589
PITA_000077490	Low quality	AT2G36885	MA_145721g0010	shade<sun	translation initiation factor;(source:Araport11)		chloroplast-GO:0009507,m	190.4936047	-0.855127706	0.12273432	-6.967307397	3.23E-12	2.44E-10
PITA_000077519	High quality	AT2G20560	MA_11777g0010	shade<sun	DNAJ heat shock family proteir	DNAJ PROTEIN (DNAJ)	chaperone binding-GO:00	164.9257521	-0.643663823	0.17399556	-3.699311711	0.000216185	0.003416156
PITA_000077630	Low quality	AT5G06320	MA_10432342g0010	shade>sun	encodes a protein whose sequ	NDR1/HIN1-LIKE 3 (NHL3)	anchored component of p	6.380842512	3.441382419	1.13628041	3.028638354	0.002456586	0.026397567
PITA_000077702	Low quality	AT1G22770	MA_10427223g0010	shade<sun	Together with CONSTANTS (CC	GIGANTEA (GI)	circadian rhythm-GO:0007	272.6936958	-0.838540691	0.14732386	-5.691818711	1.26E-08	4.83E-07
PITA_000077753	Low quality	AT5G59250	MA_698449g0010	shade<sun	Encodes a chloroplast localizec	(HP59)	carbohydrate transmembr	80.78889485	-1.279155016	0.2164208	-5.91049932	3.41E-09	1.44E-07
PITA_000077804	High quality	AT5G53370	MA_150928g0020	shade>sun	pectin methylesterase PCR frag	PECTIN METHYLESTERASE	cell wall modification-GO:	9.87811997	1.990072154	0.67343295	2.955115524	0.00312552	0.031861606
PITA_000077854	High quality	AT3G55240	MA_474901g0010	shade<sun	Overexpression leads to PEL (Pseudo-Etiolation in Light) p		chloroplast-GO:0009507,d	1206.838519	-1.172126937	0.34169695	-3.430311356	0.000602889	0.008277007
PITA_000077880	Low quality	AT4G16265	MA_10427302g0010	shade>sun	One of two highly similar, non-	(NRPB9B)	DNA-directed 5'-3' RNA pc	38.53985701	8.563840599	1.46176028	5.858580721	4.67E-09	1.94E-07
PITA_000078046	High quality	AT2G41430	MA_417026g0010	shade>sun	Encodes hydrophilic protein la	EARLY RESPONSIVE TO DE	cellular response to hypox	32.74984315	0.992931604	0.28342455	3.503336557	0.000459469	0.006569046
PITA_000078059	High quality	AT1G13570	MA_178929g0010	shade<sun	F-box/RNI-like superfamily protein;(source:Araport11)		biological_process_unknov	63.66134154	-1.07319428	0.33537341	-3.19999814	0.001374285	0.016465302
PITA_000078095	High quality	AT5G56860	MA_110551g0010	shade<sun	Encodes a member of the GAT	GATA, NITRATE-INDUCIBL	chloroplast elongation-GO	342.1040527	-1.11258013	0.17240422	-6.453322966	1.09E-10	5.93E-09
PITA_000078151	High quality	AT4G16260	MA_100289g0010	shade>sun	Encodes a putative beta-1,3-endoglucanase that interacts		anchored component of p	16.49763941	2.925150029	0.91359052	3.201817405	0.001365635	0.01640063
PITA_000078241	High quality	AT5G65460	MA_12484g0010	shade<sun	Kinesin that binds cyclin-deper	KINESIN LIKE PROTEIN FO	ATP binding-GO:0005524,	186.0194584	-1.171321844	0.39244918	-2.984645933	0.002839067	0.029648563
PITA_000078262	High quality	AT2G24280	MA_10430728g0010	shade>sun	alpha/beta-Hydrolases superfamily protein;(source:Arapo		chloroplast-GO:0009507,d	76.33947267	1.291961576	0.43694506	2.956805555	0.003108441	0.031738816
PITA_000078275	High quality	AT1G68590	MA_10432955g0010	shade<sun	Ribosomal protein PSRP-3/Ycf6	PLASTID-SPECIﬁC	chloroplast-GO:0009507,c	188.9550474	-1.975945531	0.67084004	-2.945479425	0.003224546	0.03267298
PITA_000078310	Low quality	AT3G23255	MA_5651g0010	shade>sun	tRNA dimethylallyltransferase;(source:Araport11)		ATPase activity-GO:00168	68.10187348	1.961648583	0.70720939	2.773787533	0.005540785	0.049069452
PITA_000078434	Low quality	AT1G01140	MA_10092g0010	shade<sun	Encodes a CBL-interacting prot	CBL-INTERACTING PROTEI	cellular response to potas	72.10070599	-0.562234875	0.20248625	-2.77665705	0.005492109	0.048755731
PITA_000078444	High quality	AT3G25810	MA_10121006g0010	shade<sun	Terpenoid cyclases/Protein prenyltransferases superfamil		(4S)-limonene synthase ac	39688.91475	-2.14499883	0.6012162	-3.567766162	0.000360038	0.005347555
PITA_000078497	Low quality	AT2G36290	MA_18669g0010	shade<sun	alpha/beta-Hydrolases superfamily protein;(source:Arapo		endoplasmic reticulum-GC	151.5611956	-10.87369516	3.90709151	-2.783066416	0.005384778	0.04807225
PITA_000078556	High quality	AT1G78440	MA_26738g0010	shade<sun	Encodes a gibberellin 2-oxidas	ARABIDOPSIS THALIANA G	C-19 gibberellin 2-beta-dic	279.1633219	-1.450218796	0.34603979	-4.19090184	2.78E-05	0.00057156
PITA_000078623	High quality	AT1G22640	MA_8147g0020	shade<sun	MYB-type transcription factor (	MYB DOMAIN PROTEIN 3	response to wounding-GO	543.8258224	-1.594540279	0.1261342	-12.64161734	1.24E-36	1.36E-33
PITA_000078742	High quality	AT3G24240	MA_18697g0010	shade>sun	RGFR1 is a leucine--rich repeat	(RGFR1)	maintenance of root meris	91.08876508	0.8754196	0.28113714	3.113852495	0.001846618	0.02099716
PITA_000079095	High quality	AT3G57070	MA_68307g0010	shade>sun	Glutaredoxin family protein;(source:Araport11)		chloroplast-GO:0009507,p	40.23494517	0.735499225	0.24552346	2.995637231	0.00273872	0.028779387
PITA_000079098	High quality	AT3G57810	MA_20446g0010	shade<sun	Cysteine proteinases superfamily protein;(source:Araport		chloroplast-GO:0009507,m	83.26471096	-0.514808043	0.17346943	-2.967716148	0.003000213	0.03083348
PITA_000079120	High quality	AT5G06290	MA_92546g0010	shade<sun	Encodes a 2-Cys peroxiredoxin	2-CYSTEINE PEROXIREDO	antioxidant activity-GO:00	516.4449283	-1.134177044	0.27814791	-4.077603996	4.55E-05	0.000878687
PITA_000079320	High quality	AT2G38970	MA_15445g0010	shade>sun	Zinc finger (C3HC4-type RING finger) family protein;(sourc		extracellular region-GO:00	42.17654764	0.87775134	0.283664	3.094334669	0.001972548	0.022218701
PITA_000079330	High quality	AT3G10870	MA_10772g0010	shade>sun	Encodes a methyl IAA esterase	METHYL ESTERASE 17 (ME	hydrolase activity, acting c	21.49434264	1.809564238	0.41287229	4.382866785	1.17E-05	0.000265873
PITA_000079442	High quality	AT3G06590	MA_102253g0010	shade<sun	Encodes RITF1, a bHLH transcri	ATBS1 INTERACTING FACT	DNA-binding transcription	336.470898	-12.02419582	3.90691191	-3.077672617	0.00208624	0.023219314
PITA_000079585	Low quality	AT2G44130	MA_10305005g0020	shade<sun	Encodes a member of a family	KISS ME DEADLY 3 (KMD3	cytoplasm-GO:0005737,cy	319.4073011	-1.150329138	0.37289134	-3.084890996	0.002036268	0.022793906
PITA_000079630	High quality	AT5G66120	MA_4908g0010	shade<sun	3-dehydroquinate synthase;(source:Araport11)		3-dehydroquinate synthas	208.1634268	-1.04566612	0.14071312	-7.431191362	1.08E-13	1.07E-11
PITA_000079828	Low quality	AT5G25810	MA_82481g0010	shade>sun	encodes a member of the DREI	TINY (tny)	DNA-binding transcription	56.07618054	1.178298972	0.26576769	4.433567482	9.27E-06	0.000214651
PITA_000079835	High quality	AT3G55800	MA_10427281g0010	shade<sun	Encodes the chloroplast enzym	SEDOHEPTULOSE-BISPHO	apoplast-GO:0048046,carl	413.9076302	-1.172022327	0.16146033	-7.258887042	3.90E-13	3.52E-11

PITA_000079836	High quality	AT3G51830	MA_935712g0010	shade<sun	putative transmembrane prote	SAC DOMAIN-CONTAINING	endoplasmic reticulum-GC	33.47371679	-8.689905846	1.2181982	-7.133408892	9.79E-13	8.31E-11
PITA_000080057	High quality	AT1G48300	MA_585559g0010	shade<sun	Cytosolic iron-sulfur protein wi	DIACYLGLYCEROL ACYLTR	cytosol-GO:0005829,diacy	234.877719	-1.935133349	0.21356033	-9.061295965	1.29E-19	3.13E-17
PITA_000080116	Low quality	AT5G59970	MA_10427181g0010	shade<sun	Histone superfamily protein;(source:Araport11)		nucleus-GO:0005634,plast	84.69727511	0.927842884	0.31136144	2.979954372	0.002882913	0.029994666
PITA_000080189	High quality	AT1G71790	MA_51020g0020	shade<sun	Encodes a heterodimeric actin	CAPPING PROTEIN B (CPB	actin cytoskeleton organiz	159.9907675	-0.69679209	0.18352788	-3.796655346	0.000146661	0.002443066
PITA_000080214	High quality	AT5G48810	MA_15972g0010	shade<sun	Encodes a cytochrome b5 isofc	CYTOCHROME B5 ISOFOR	defense response to bacte	243.1097333	-1.637525823	0.43365076	-3.776139614	0.000159278	0.002628917
PITA_000080233	High quality	AT5G60770	MA_80387g0010	shade<sun	member of High affinity nitrate	NITRATE TRANSPORTER 2.	cellular response to nitrate	252.555429	-1.112798682	0.22651096	-4.912780654	8.98E-07	2.55E-05
PITA_000080340	High quality	AT2G20980	MA_53566g0010	shade<sun	Similar to MCM10, which in otl	MINICHROMOSOME MAII	DNA replication initiation-	262.0913697	-0.786002161	0.201789	-3.895168508	9.81E-05	0.001718646
PITA_000080632	High quality	AT2G23540	MA_10434622g0020	shade<sun	GDSL-motif esterase/acyltransferase/lipase. Enzyme grou		chloroplast-GO:0009507,h	81.40294298	1.180930017	0.20950202	5.636843182	1.73E-08	6.57E-07
PITA_000080646	Low quality	AT3G14170	MA_88653g0010	shade<sun	CORD1 is a member of a novel	CORTICAL MICROTUBULE	chloroplast-GO:0009507,c	472.2247053	-12.51355955	1.48365184	-8.434296529	3.33E-17	5.96E-15
PITA_000080652	High quality	AT1G69550	MA_10437104g0020	shade<sun	disease resistance protein (TIR-NBS-LRR class);(source:Ara		ADP binding-GO:0043531,	113.569732	10.12393774	1.45466529	6.959633793	3.41E-12	2.53E-10
PITA_000080981	Low quality	AT3G07320	MA_10426132g0010	shade<sun	O-Glycosyl hydrolases family 17 protein;(source:Araport11		anchored component of p	174.8388456	-11.07949439	1.49751631	-7.398580115	1.38E-13	1.34E-11
PITA_000081165	High quality	AT4G10770	MA_10267817g0010	shade<sun	oligopeptide transporter	OLIGOPEPTIDE TRANSPOF	integral component of pla	67.79184164	0.669707714	0.23887652	2.803572848	0.005053981	0.046045755
PITA_000081180	High quality	AT5G16770	MA_1261g0010	shade<sun	Member of the R2R3 factor gei	MYB DOMAIN PROTEIN 9	DNA-binding transcription	7728.170797	-2.223525759	0.53695863	-4.140962866	3.46E-05	0.000688426
PITA_000081335	Low quality	AT1G23980	MA_15311g0010	shade<sun	RING/U-box superfamily prote	ARABIDOPSIS T??XICOS EI	nucleus-GO:0005634,prote	5.957787811	5.871457257	1.84356867	3.184832407	0.001448379	0.01724651
PITA_000081372	Low quality	AT5G21930	MA_169909g0010	shade<sun	P-Type ATPase, mediates copp	P-TYPE ATPASE OF ARABII	ATPase-coupled cation tra	347.352702	-1.569747535	0.43820805	-3.582196951	0.000340717	0.00509963
PITA_000081453	High quality	AT5G02160	MA_1640g0010	shade<sun	Zinc-finger domain containing	FTSH5 INTERACTING PROT	chloroplast-GO:0009507,c	316.4108637	-0.857357231	0.12634179	-6.786014636	1.15E-11	7.75E-10
PITA_000081470	Low quality	AT5G47750	MA_10430800g0010	shade<sun	D6PK family kinase involved in	D6 PROTEIN KINASE LIKE :	basipetal auxin transport-(	47.11173464	1.346795866	0.26373687	5.106589303	3.28E-07	9.96E-06
PITA_000081918	High quality	AT5G01410	MA_10426564g0030	shade<sun	Encodes a protein predicted to	REDUCED SUGAR RESPON	amine-lyase activity-GO:0(	682.3731378	-1.688323606	0.22053481	-7.655587682	1.92E-14	2.13E-12
PITA_000082065	High quality	AT2G26710	MA_218197g0020	shade<sun	Encodes a member of the cyto	PHYB ACTIVATION TAGGE	brassinosteroid homeosta	56.08312495	9.104920338	1.35966564	6.696440701	2.14E-11	1.35E-09
PITA_000082093	High quality	AT2G30870	MA_10333127g0010	shade<sun	early dehydration-induced gen	GLUTATHIONE S-TRANSFE	apoplast-GO:0048046,cell	25.00423782	1.340759016	0.33749324	3.972698921	7.11E-05	0.001290284
PITA_000082198	High quality	AT4G02420	MA_168754g0010	shade<sun	Concanavalin A-like lectin prot	L-TYPE LECTIN RECEPTOR	defense response to bacte	304.8561213	-11.88187434	3.90692718	-3.04123261	0.002356117	0.025557539
PITA_000082366	High quality	AT1G79220	MA_696298g0010	shade<sun	Mitochondrial transcription ter	(MTERF17)	chloroplast-GO:0009507,c	383.9249516	-1.371344249	0.21390375	-6.411034247	1.45E-10	7.72E-09
PITA_000082449	High quality	AT5G47840	MA_10427036g0010	shade<sun	adenosine monophosphate kir	ADENOSINE MONOPHOSP	adenylate kinase activity-G	2583.059912	-2.181776177	0.32818709	-6.647964734	2.97E-11	1.83E-09
PITA_000082539	Low quality	AT1G21590	MA_19102g0010	shade<sun	kinase with adenine nucleotide	alpha hydrolases-like dom	nucleus-GO:0005634,plasr	28.65488755	8.135894262	1.5867312	5.127455922	2.94E-07	8.99E-06
PITA_000082667	Low quality	AT1G63450	MA_9990400g0010	shade<sun	Encodes a xyloglucan-specific g	ROOT HAIR SPECIFIC 8 (RF	cell wall biogenesis-GO:00	661.5042579	-2.191643907	0.27423795	-7.991760198	1.33E-15	1.82E-13
PITA_000082809	High quality	AT4G26790	MA_127685g0010	shade<sun	GDSL-motif esterase/acyltransferase/lipase. Enzyme grou		hydrolase activity, acting c	168.7516791	-0.929932202	0.23505452	-3.956240419	7.61E-05	0.001372562
PITA_000083010	High quality	AT3G14260	MA_77009g0010	shade<sun	LURP-one-like protein (DUF567);(source:Araport11)		biological_process_unknov	41.18792526	8.660331397	1.22446012	7.072775391	1.52E-12	1.22E-10
PITA_000083179	Low quality	AT3G61113	MA_129565g0010	shade<sun	Ubiquitin related modifier 1;(s(	UBIQUITIN-RELATED MOD	nucleus-GO:0005634,prote	202.2260716	-11.28974171	3.90700964	-2.889611945	0.003857176	0.03741859
PITA_000083259	High quality	AT4G39330	MA_135446g0010	shade<sun	cinnamyl alcohol dehydrogena	CINNAMYL ALCOHOL DEH	apoplast-GO:0048046,cinn	79.45426584	0.818424248	0.24707808	3.312411436	0.000924954	0.011829281
PITA_000083410	High quality	AT4G23900	MA_10428684g0020	shade<sun	Nucleoside diphosphate kinase family protein;(source:Ara		chloroplast-GO:0009507,C	142.2125909	-0.820748806	0.16048509	-5.11417497	3.15E-07	9.57E-06
PITA_000083435	High quality	AT4G23180	MA_10237752g0010	shade<sun	Encodes a receptor-like protei	CYSTEINE-RICH RLK (RECE	cytosol-GO:0005829,defer	450.1405781	-12.44423973	1.52908852	-8.13833835	4.01E-16	6.20E-14
PITA_000083544	Low quality	AT4G28706	MA_10436385g0010	shade<sun	pfkB-like carbohydrate kinase family protein;(source:Arap		chloroplast-GO:0009507,c	364.7270087	-1.133910286	0.16291437	-6.960161169	3.40E-12	2.53E-10
PITA_000083693	High quality	AT4G26780	MA_315276g0010	shade<sun	unknown function	(AR192)	adenyl-nucleotide exchang	96.32145303	-0.7738778	0.20739722	-3.731379859	0.000190434	0.003066884
PITA_000083910	Low quality	AT5G18700	MA_10431492g0010	shade<sun	Encodes CBL-interacting protei	CBL-INTERACTING PROTEI	plasma membrane-GO:000	15.36845612	2.341827521	0.78430344	2.985869253	0.002827735	0.029542453
PITA_000084024	High quality	AT3G07350	MA_6561322g0010	shade<sun	sulfate/thiosulfate import ATP-binding protein, putative (I		cellular response to hypox	574.3685212	1.710850878	0.52938469	3.231772464	0.00123025	0.015003336
PITA_000084107	High quality	AT1G79630	MA_2329g0010	shade<sun	Protein phosphatase 2C family protein;(source:Araport11)		chloroplast-GO:0009507,c	41.53389082	1.019228025	0.24604642	4.142421734	3.44E-05	0.000685142
PITA_000084122	High quality	AT5G48930	MA_125704g0010	shade<sun	At5g48930 has been shown to	HYDROXYCINNAMOYL-CO	auxin homeostasis-GO:001	103.0379099	1.39701041	0.41205584	3.390342471	0.000698054	0.009329577
PITA_000084169	High quality	AT4G19970	MA_175330g0010	shade<sun	nucleotide-diphospho-sugar transferase family protein;(sc		cytoplasm-GO:0005737	197.7978001	1.877307863	0.48070313	3.9053373	9.41E-05	0.001653695
PITA_000084272	High quality	AT5G55090	MA_72906g0010	shade<sun	member of MEKK subfamily	MITOGEN-ACTIVATED PRC	nucleus-GO:0005634,prote	25.21175418	5.129269923	1.32543317	3.869881976	0.000108888	0.001879642
PITA_000084437	High quality	AT2G36970	MA_10434576g0010	shade<sun	UDP-Glycosyltransferase superfamily protein;(source:Arap		transferase activity, transfe	454.0924252	-12.45682829	1.53497969	-8.11530496	4.85E-16	7.15E-14
PITA_000084443	High quality	AT2G35660	MA_495566g0010	shade<sun	Encodes a member of a novel ¡	(CTF2A)	chloroplast-GO:0009507,F	84.75842623	-0.8401637	0.22694618	-3.70203947	0.000213873	0.003388119
PITA_000084506	Low quality	AT5G56550	MA_345439g0010	shade<sun	Encodes OXIDATIVE STRESS 3 (	OXIDATIVE STRESS 3 (OXS	molecular_function_unkno	9.953726933	6.613036769	1.43525583	4.607566565	4.07E-06	0.000102034
PITA_000084544	Low quality	AT1G29070	MA_10435644g0010	shade<sun	Ribosomal protein L34;(source	PLASTID RIBOSOMAL PRO	chloroplast-GO:0009507,n	365.4009239	-0.996139626	0.0879527	-11.3258564	9.77E-30	5.87E-27
PITA_000084603	Low quality	AT1G28230	MA_5803g0010	shade<sun	Encodes a transporter that trar	PURINE PERMEASE 1 (PUF	chloroplast-GO:0009507,n	57.30910696	9.13796694	1.2812104	7.132292223	9.87E-13	8.35E-11
PITA_000084692	Low quality	AT3G15353	MA_183668g0010	shade<sun	metallothionein, binds to and (	METALLOTHIONEIN 3 (MT	cellular copper ion homeo	507.6793916	-0.678498485	0.16264908	-4.17154835	3.03E-05	0.00061433
PITA_000084730	High quality	AT5G09500	MA_14543g0010	shade<sun	Ribosomal protein S19 family protein;(source:Araport11)		cytoplasm-GO:0005737,cy	171.882439	0.906077363	0.2877441	3.148899871	0.001638863	0.019034007
PITA_000084895	Low quality	AT2G17710	MA_116906g0010	shade<sun	Big1;(source:Araport11)		biological_process_unkno	160.6569892	-0.630882718	0.18418802	-3.425210354	0.000614324	0.00839742
PITA_000084952	Low quality	AT3G19430	MA_10430750g0010	shade<sun	late embryogenesis abundant protein-related / LEA prote		extracellular region-GO:0C	419.4484759	-1.309478841	0.13036921	-10.04438697	9.73E-24	3.77E-21
PITA_000085061	High quality	AT1G49770	MA_93736g0010	shade<sun	Encodes a member of the basi	RETARDED GROWTH OF E	DNA-binding transcription	229.7235785	2.62151882	0.67445794	3.886852934	0.000101552	0.001771195
PITA_000085122	Low quality	AT2G15490	MA_10431618g0010	shade<sun	UDP-glycosyltransferase 73B4;	UDP-GLYCOSYLTRANSFER	chloroplast-GO:0009507,c	47.60078593	1.136291445	0.40905541	2.77784237	0.005472116	0.048679633
PITA_000085628	Low quality	AT5G03940	MA_780476g0010	shade<sun	mutant has Yellow first leaves;	CHLOROPLAST SIGNAL RE(	7S RNA binding-GO:00083	287.0815903	-0.935904783	0.24640474	-3.798241742	0.000145726	0.002430697
PITA_000085662	High quality	AT3G26060	MA_10197498g0010	shade<sun	encodes periredoxin Q which c	PEROXIREDOXIN Q (PRXQ	antioxidant activity-GO:00	1342.327186	-1.697097104	0.22940724	-7.397748638	1.39E-13	1.34E-11
PITA_000085676	High quality	AT5G06720	MA_111431g0010	shade<sun	Encodes a peroxidase with dive	PEROXIDASE 2 (PA2)	defense response to nema	156.4270465	-1.326206456	0.45418533	-2.919967639	0.003500677	0.034766771
PITA_000085789	High quality	AT1G64970	MA_10432303g0020	shade<sun	gamma-tocopherol methyltran	GAMMA-TOCOPHEROL M	chloroplast-GO:0009507,n	48.53325639	1.367213057	0.3611388	3.785838195	0.000153191	0.002538427
PITA_000085874	High quality	AT5G05320	MA_13849g0010	shade<sun	FAD/NAD(P)-binding oxidoreductase family protein;(sourc		chloroplast-GO:0009507,F	84.64625517	-0.908819957	0.21545723	-4.218099113	2.46E-05	0.000513509
PITA_000085978	High quality	AT1G53540	MA_10291578g0010	shade<sun	Member of the class I small he	(HSP17.6C)	cytoplasm-GO:0005737,pr	40.09567486	0.851099553	0.25708281	3.310604669	0.000930946	0.011893853
PITA_000086163	High quality	AT3G15730	MA_6712g0010	shade<sun	Encodes phospholipase D alph	PHOSPHOLIPASE D ALPHA	abscisic acid-activated sigr	497.408277	-0.860678499	0.28070781	-3.066109978	0.002168701	0.02395735
PITA_000086223	High quality	AT3G61120	MA_13933g0010	shade<sun	Encodes AGL13, a member of t	AGAMOUS-LIKE 13 (AGL13	DNA-binding transcription	74.5563627	1.22283161	0.3716931	3.289895892	0.001002245	0.012631988
PITA_000086371	Low quality	AT5G01110	MA_10430089g0010	shade<sun	Tetratricopeptide repeat (TPR)-like superfamily protein;(s		mitochondrion-GO:00057:	773.2291103	-1.306820372	0.1370999	-9.531883948	1.54E-21	4.53E-19
PITA_000086422	High quality	AT1G62390	MA_82470g0010	shade<sun	Encodes one of the 36 carboxy	PHOX2 (Phox2)	cytosol-GO:0005829,mRN/	121.7845898	-0.921399722	0.26016752	-3.541563275	0.000397763	0.005805321
PITA_000086528	High quality	AT5G09220	MA_889393g0010	shade<sun	member of AAAP family The m	AMINO ACID PERMEASE 2	acidic amino acid transpor	203.9130795	10.96787582	3.90695098	2.807272443	0.004996297	0.045720827
PITA_000086570	High quality	AT3G56650	MA_478623g0010	shade<sun	thylakoid lumenal protein (Mo	PSBP-DOMAIN PROTEIN 6	calcium ion binding-GO:0C	242.344187	-0.903353938	0.17249291	-5.237049674	1.63E-07	5.22E-06
PITA_000086610	High quality	AT5G66190	MA_193431g0010	shade<sun	Encodes a leaf-type ferredoxin	FERREDOXIN-NADP(+)-OX	apoplast-GO:0048046,chlc	909.3175934	-1.09617866	0.16061322	-6.824959111	8.80E-12	6.06E-10
PITA_000086655	Low quality	AT4G16600	MA_103998g0010	shade<sun	Nucleotide-diphospho-sugar tr	PLANT GLYCOGENIN-LIKE	transferase activity, transfe	120.9223925	-1.249243475	0.4335438	-2.881470081	0.003958248	0.038247899
PITA_000086772	High quality	AT1G28130	MA_16777g0010	shade<sun	Encodes an IAA-amido synthas	GRETCHEN HAGEN 3.17 (G	acid-amino acid ligase acti	31.85090794	0.955330515	0.29152709	3.276987147	0.001049211	0.013132066
PITA_000086803	High quality	AT1G80770	MA_10431481g0010	shade<sun	P-loop containing nucleoside t	PIGMENT DEFECTIVE 318	nucleus-GO:0005634	224.9280872	-1.019567578	0.29209695	-3.490510839	0.000482098	0.006834465
PITA_000086819	Low quality	AT4G38540	MA_10427607g0010	shade<sun	FAD/NAD(P)-binding oxidoreductase family protein;(sourc		FAD binding-GO:0071949,	44.07585145	-1.923444342	0.3247946	-5.92203294	3.18E-09	1.35E-07
PITA_000086856	Low quality	AT5G66631	MA_89328g0010	shade<sun	Tetratricopeptide repeat (TPR)-like superfamily protein;(s		biological_process_unkno	659.0898659	-1.212310356	0.17524453	-6.917821246	4.59E-12	3.32E-10
PITA_000086975	High quality	AT3G15030	MA_52440g0010	shade<sun	Arabidopsis thaliana TCP famil	TCP FAMILY TRANSCRIPTI	cell differentiation-GO:003	138.589824	-0.735629747	0.20533423	-3.582596743	0.000340196	0.005094853
PITA_000086995	Low quality	AT3G52930	MA_101067g0010	shade<sun	Aldolase superfamily protein;(	FRUCTOSE-BISPHOSPHATI	apoplast-GO:0048046,cell	87.72081039	0.644045354	0.20711943	3.1095361	0.001873814	0.021258473
PITA_000087012	High quality	AT5G22810	MA_95656g0010	shade<sun	GDSL-motif esterase/acyltransferase/lipase. Enzyme grou		extracellular region-GO:0C	89.97051697	0.724743693	0.22722207	3.189583206	0.001424781	0.017013722
PITA_000087198	High quality	AT4G04630	MA_93306g0010	shade<sun	senescence regulator (Protein of unknown function, DUF5		biological_process_unkno	14.09519688	2.91993111	0.57704825	5.06011605	4.19E-07	1.25E-05
PITA_000087226	Low quality	AT3G43660	MA_8280g0010	shade<sun	The gene encodes a putative nodulin-like21 protein.		cellular manganese ion ho	40.63185565	1.890901408	0.63666731	2.969999211	0.002978005	0.030693734
PITA_000087346	High quality	AT5G41460	MA_10431764g0010	shade<sun	transferring glycosyl group transferase (DUF604);(source:/		acetylglucosaminyltransfer	115.7362801	-1.498146546	0.49623688	-3.019014916	0.00253598	0.02704331
PITA_000087366	High quality	AT5G40350	MA_113824g0010	shade<sun	Myb24 transcription factor. M(	MYB DOMAIN PROTEIN 2/	DNA-binding transcription	114.6182139	10.13746727	1.27620784	7.943429683	1.97E-15	2.60E-13
PITA_000087507	High quality	AT5G15490	MA_65570g0010	shade<sun	Encodes one of four UDP-gluc	UDP-GLUCOSE DEHYDROG	carbohydrate metabolic pr	88.15138727	-1.312946891	0.46317309	-2.834678689	0.00458718	0.042791437
PITA_000087508	High quality	AT4G16740	MA_74237g0010	shade<sun	Encodes an (E,E)-alpha-farnese	TERPENE SYNTHASE 03 (TI	(E)-beta-ocimene synthase	354.9994829	-2.382026619	0.47731978	-4.990420959	6.02E-07	1.76E-05
PITA_000087677	High quality	AT5G13930	MA_10426264g0020	shade<sun	Encodes chalcone synthase (CH	TRANSPARENT TESTA 4 (T	auxin polar transport-GO:(	21.7929441	7.741827757	1.4800402	5.230822613	1.69E-07	5.37E-06
PITA_000087848	High quality	AT1G64160	MA_10436984g0050	shade<sun	Encodes a dirigent protein invc	DIRIGENT PROTEIN 5 (DIR	(-)-pinoresinol biosyntheti	28.58684514	2.132093707	0.49159537	4.337090682	1.44E-05	0.000317868
PITA_000087870	High quality	AT3G02645	MA_140987g0010	shade<sun	transmembrane protein, putative (DUF247);(source:Arapc		biological_process_unkno	17.29758028	7.409634045	1.41028267	5.254006308	1.49E-07	4.81E-06
PITA_000087910	High quality	AT3G09310	MA_10435068g0010	shade<sun	membrane protein insertion efficiency factor;(source:Araj		biological_process_unkno	178.6102966	-0.926051077	0.2772307	-3.340362612	0.000836691	0.010891979
PITA_000088023	High quality	AT5G50900	MA_10427334g0010	shade<sun	ARM repeat superfamily protein;(source:Araport11)		biological_process_unkno	118.3089306	0.682486701	0.20280921	3.365166189	0.000764976	0.010085043
PITA_000088418	High quality	AT5G19200	MA_681548g0010	shade<sun	Encodes one of the Arabidopsi	TSC10B (TSC10B)	3-dehydrosphinganine red	94.79106607	0.790025551	0.21230408	3.721198084	0.00019828	0.003168959
PITA_000088502	High quality	AT5G13900	MA_26320g0020	shade<sun	Bifunctional inhibitor/lipid-trar	GLYCOSYLPHOSPHATIDYLI	anchored component of m	40.98144783	0.951629841	0.26581698	3.580019024	0.000343569	0.005130145
PITA_000088566	High quality	AT5G16370	MA_10436072g0030	shade<sun	acyl activating enzyme 5;(sourc	ACYL ACTIVATING ENZYM	chloroplast-GO:0009507,p	18.61612409	1.921135732	0.67968113	2.826525058	0.004705606	0.043727115
PITA_000088610	High quality	AT1G75350	MA_10430444g0010	shade<sun	Ribosomal protein L31;(source	EMBRYO DEFECTIVE 2184	chloroplast-GO:0009507,c	276.6519329	-0.80760941	0.13725497	-5.884008644	4.00E-09	1.68E-07
PITA_000088703	High quality	AT1G80920	MA_10431645g0020	shade<sun	A nuclear encoded soluble pro	(J8)	chloroplast-GO:0009507,c	48.1441944	1.38043329	0.39971144	3.453574622	0.000553209	0.007703996
PITA_000088795	High quality	AT5G02500	MA_10427429g0010	shade<sun	Encodes a member of heat shc	HEAT SHOCK COGNATE PF	apoplast-GO:0048046,ATP	92.29030575	-10.15902144	1.42700116	-7.119140308	1.09E-12	9.16E-11
PITA_000088923	High quality	AT5G53450	MA_85011g0010	shade<sun	OBP3-responsive protein 1;(so	OBP3-RESPONSIVE GENE :	ATP binding-GO:0005524,(	103.6009185	-2.593016163	0.74626203	-3.474672511	0.000511477	0.00721043
PITA_000088969	High quality	AT5G47470	MA_61847g0010	shade<sun	nodulin MtN21-like transporte	USUALLY MULTIPLE ACIDS	plasma membrane-GO:000	167.3032735	-1.71656556	0.5425738	-3.163745777	0.001557528	0.018338399
PITA_000089013	High quality	AT1G22380	MA_167914g0010	shade<sun	Encodes a putative UDP-glucos	UDP-GLUCOSYL TRANSFEF	glucuronosyltransferase ac	50.1233217	1.011662679	0.24103183	4.197216063	2.70E-05	0.00055768
PITA_000089129	Low quality	AT1G32490	MA_277016g0010	shade<sun	Encodes a homolog of the yeas	ENHANCED SILENCING PH	embryo development end	33.02165172	8.33945414	1.20294584	6.932526693	4.13E-12	3.01E-10
PITA_000089328	High quality	AT3G48280	MA_353950g0010	shade<sun	putative cytochrome P450	CYTOCHROME P450, FAM	Golgi apparatus-GO:00057	58.67537756	0.844638197	0.22390127	3.772369	0.000161705	0.002659983
PITA_000089685	High quality	AT2G16850	MA_10426909g0020	shade<sun	plasma membrane intrinsic pro	PLASMA MEMBRANE INTF	cytoplasm-GO:0005737,pl;	270.4320662	-11.7089979	3.90694786	-2.996968044	0.002726793	0.028677948
PITA_000089826	High quality	AT1G60420	MA_10122651g0010	shade<sun	Reduce transmission through ¡	NUCLEOREDOXIN 1 (NRX1	cytosol-GO:0005829,nucle	234.1508549	-1.619502829	0.5767495	-2.807983053	0.004985285	0.045709392
PITA_000089862	Low quality	AT3G03980	MA_10435595g0030	shade<sun	NAD(P)-binding Rossmann-fold superfamily protein;(sourc		chloroplast-GO:0009507	382.3456166	-1.239382481	0.20225853	-6.127714128	8.92E-10	4.14E-08
PITA_000090045	High quality	AT1G67110	MA_171397g0010	shade<sun	cytochrome P450, family 735, s	CYTOCHROME P450, FAM	extracellular region-GO:0C	14541.80523	-2.029049145	0.2193767	-9.249155363	2.26E-20	5.71E-18
PITA_000090046	High quality	AT2G17260	MA_10429132g0010	shade<sun	Encodes a glutamate receptor.	GLUTAMATE RECEPTOR 2	calcium channel activity-G	154.8960463	-10.90559546	1.66148486	-6.563764555	5.25E-11	3.05E-09
PITA_000090050	Low quality	AT2G35930	MA_33190g0010	shade<sun	Encodes a cytoplasmically loca	PLANT U-BOX 23 (PUB23)	cytoplasm-GO:0005737,cy	20.1266697	7.62679279	1.78094968	4.282430242	1.85E-05	0.00039645
PITA_000090060	High quality	AT4G23340	MA_194692g0010	shade<sun	2-oxoglutarate (2OG) and Fe(II)-dependent oxygenase sup		chloroplast-GO:0009507,c	157.626818	-10.93018476	3.90707896	-2.797533624	0.005149441	0.046598096
PITA_000090093	High quality	AT3G02830	MA_1139g0010	shade<sun	Encodes a zinc finger protein tl	ZINC FINGER PROTEIN 1 (Z	cytosol-GO:0005829,DNA	109.5359531	-0.610094193	0.18404196	-3.314973271	0.000916519	0.011733303
PITA_000090576	High quality	AT2G47820	MA_208236g0010	shade<sun	arginine-glutamic acid dipeptide repeat protein;(source:A		biological_process_unkno	128.1388555	-1.860158266	0.60065023	-3.096907624	0.001955508	0.022056311
PITA_000090670	Low quality	AT5G06600	MA_10428769g0010	shade<sun	Encodes a ubiquitin-specific pr	UBIQUITIN-SPECIFIC PROT	cysteine-type endopeptida	34.71528234	-8.745632426	1.66154179	-5.263564534	1.41E-07	4.57E-06
PITA_000090746	High quality	AT4G12290	MA_18326g0010	shade<sun	Copper amine oxidase. Induce(	COPPER AMINE OXIDASE I	aliphatic-amine oxidase ac	47.28826619	-1.641352291	0.39238575	-4.183006845	2.88E-05	0.000588423
PITA_000090778	High quality	AT3G24660	MA_13257g0010	shade<sun	member of Receptor kinase-lik	TRANSMEMBRANE KINASI	ATP binding-GO:0005524,I	43.02135079	1.736319396	0.47441697	3.659901515	0.000252312	0.003925548
PITA_000090826	Low quality	AT3G14440	MA_10434448g0010	shade<sun	Encodes 9-<i>cis</i>-epoxycar	NINE-CIS-EPOXYCAROTEN	9-cis-epoxycarotenoid dio:	1405.531488	-1.384611927	0.46151633	-3.000136394	0.002698587	0.028416857
PITA_000091053	Low quality	AT5G58360	MA_10434319g0010	shade<sun	ovate family protein 3;(source:	OVATE FAMILY PROTEIN 3	DNA binding-GO:0003677,	453.4880307	-1.055562871	0.23186864	-4.552417317	5.30E-06	0.000130233
PITA_000091097	High quality	AT2G24130	MA_10430603g0010	shade<sun	Leucine-rich receptor-like protein kinase family protein;(s		plasma membrane-GO:000	429.4140518	-1.300254329	0.39860475	-3.262014174	0.001106236	0.013723205
PITA_000091272	High quality	AT2G21050	MA_131574g0010	shade<sun	Encodes LAX2 (LIKE AUXIN RES	LIKE AUXIN RESISTANT 2 (	amino acid transmembran	47.41905278	0.737345575	0.26210541	2.813164302	0.004905658	0.045203029
PITA_000091318	High quality	AT5G40380	MA_27101g0010	shade<sun	Encodes a cysteine-rich recept	CYSTEINE-RICH RLK (RECE	chloroplast-GO:0009507,k	38.82969103	8.574799332	2.0746771	4.133076571	3.58E-05	0.000708018
PITA_000091334	High quality	AT3G23810	MA_2976308g0010	shade<sun	S-adenosyl-l-homocysteine (SA	S-ADENOSYL-L-HOMOCYS	adenosylhomocysteinase a	140.7606922	-0.96252366	0.28083935	-3.427310577	0.000609591	0.008342218
PITA_000091390	Low quality	AT3G22110	MA_474079g0010	shade<sun	Encodes the alpha-3 subunit ol	20S PROTEASOME ALPHA	cytoplasm-GO:0005737,cy	129.7471721	-10.64985786	1.91216356	-5.569532895	2.55E-08	9.49E-07
PITA_000091412	Low quality	AT5G48840	MA_10437162g0010	shade<sun	Encodes a pantothenate synthe	HOMOLOG OF BACTERIAL	cytoplasm-GO:0005737,cy	54.43802039	0.678773768	0.24312408	2.791882136	0.005240244	0.047211142
PITA_000091461	High quality	AT3G12110	MA_9015982g0010	shade<sun	Encodes an actin that is expres	ACTIN-11 (ACT11)	actin cytoskeleton organiz	356.7118273	1.13221017	0.24096859	4.698579889	2.62E-06	6.80E-05
PITA_000091467	High quality	AT1G04040	MA_948726g0010	shade<sun	HAD superfamily, subfamily IIIB acid phosphatase;(source		acid phosphatase activity-	21.57221123	1.385032642	0.42661755	3.246544	0.001168154	0.014357135
PITA_000091584	High quality	AT5G05170	MA_10429339g0020	shade<sun	Encodes a cellulose synthase is	CONSTITUTIVE EXPRESSIC	cellulose biosynthetic proc	124.9233835	-1.225838866	0.3977237	-3.0821368	0.002055104	0.022965102
PITA_000091629	High quality	AT5G48220	MA_10433830g0010	shade<sun	Aldolase-type TIM barrel family protein;(source:Araport11		chloroplast-GO:0009507,c	377.9388961	-1.819004952	0.50931012	-3.571507596	0.000354932	0.005281057
PITA_000091837	High quality	AT2G45570	MA_493501g0010	shade<sun	member of CYP76C	CYTOCHROME P450, FAM	heme binding-GO:002003	694.7080932	-3.251938775	0.33045944	-9.840659291	7.52E-23	2.67E-20
PITA_000091851	High quality	AT3G63400	MA_68436g0020	shade<sun	Cyclophilin-like peptidyl-prolyl	cis-trans isomerase family ¡	cyclosporin A binding-GO:	259.5396476	-0.755105641	0.12484311	-6.048436421	1.46E-09	6.61E-08
PITA_000091919	High quality	AT2G37220	MA_320479g0010	shade<sun	Encodes a chloroplast RNA binding protein. A substrate o		chloroplast-GO:0009507,c	153.9634602	2.465874814	0.46088632	5.350288629	8.78E-08	2.95E-06
PITA_000092059	High quality	AT3G52500	MA_176956g0010	shade<sun	Eukaryotic aspartyl protease family protein;(source:Arapo		cell wall-GO:0005618,extr;	413.3473688	-1.28122941	0.30934194	-4.141790213	3.45E-05	0.000686489
PITA_000092077	High quality	AT5G57550	MA_11177g0010	shade<sun	xyloglucan endotransglycosyla	XYLOGLUCAN ENDOTRAN:	extracellular region-GO:0C	20.59848657	7.659460637	1.70073068	4.503629378	6.68E-06	0.000160454
PITA_000092222	High quality	AT1G64190	MA_20480g0010	shade<sun	6-phosphogluconate dehydrog	6-PHOSPHOGLUCONATE [	chloroplast-GO:0009507,c	152.4280607	-10.88192005	3.90708965	-2.785172857	0.005349919	0.047930717
PITA_000092341	Low quality	AT2G20370	MA_100374g0010	shade<sun	Encodes a xyloglucan galactos	MURUS 3 (MUR3)	endomembrane system or	27.88024009	2.110619356	0.58572886	3.603406775	0.000314073	0.004743073
PITA_000092372	High quality	AT4G30825	MA_187850g0010	shade<sun	P-class pentatricopeptide repe	BIOGENESIS FACTOR REQU	chloroplast stroma-GO:000	718.3967872	-1.695473746	0.24362522	-6.959352409	3.42E-12	2.53E-10
PITA_000092427	High quality	AT1G77520	MA_10432987g0020	shade<sun	O-methyltransferase family protein;(source:Araport11)		aromatic compound biosy	46.80262198	1.834220642	0.27177301	6.74909052	1.49E-11	9.77E-10
PITA_000092453	High quality	AT4G14750	MA_98760g0010	shade<sun	Member of IQ67 (CaM binding	IQ-DOMAIN 19 (IQD19)	nucleus-GO:0005634	61.50905666	0.667247714	0.20230978	3.298148529	0.000973246	0.012365377
PITA_000092502	High quality	AT4G38400	MA_489540g0010	shade<sun	member of EXPANSIN-LIKE. Na	EXPANSIN-LIKE A2 (EXLA2	extracellular region-GO:0C	33.70594467	1.239420535	0.39765423	3.116829762	0.001828071	0.020823826
PITA_000092583	High quality	AT4G19600	MA_7608g0020	shade<sun	Encodes a cyclin T partner CYC	(CYCT1;4)	cyclin/CDK positive transc	60.54587204	1.781736277	0.37471285	4.754937752	1.99E-06	5.27E-05
PITA_000092661	High quality	AT1G67090	MA_10239665g0010	shade<sun	Encodes a member of the Rubi	RIBULOSE BISPHOSPHATE	apoplast-GO:0048046,chlc	114.4970459	0.7606738	0.20150331	3.774993976	0.000160012	0.002635853
PITA_000092680	High quality	AT1G07900	MA_170369g0010	shade<sun	LOB domain-containing protein	LOB DOMAIN-CONTAININ	nucleus-GO:0005634	417.6589363	-12.33604806	3.9068833	-3.157516397	0.001591193	0.018639055
PITA_000093007	Low quality	AT3G48560	MA_20909g0010	shade<sun	Catalyzes the formation of acet	CHLORSULFURON/IMIDAZ	acetolactate synthase activ	177.5344867	-0.79709403	0.27001407	-2.952046298	0.003156756	0.032076303
PITA_000093091	High quality	AT2G26690	MA_93406g0010	shade<sun	Major facilitator superfamily p	NRT1/ PTR FAMILY 6.2 (NF	chloroplast-GO:0009507,c	158.8147409	-10.9141489	1.83113108	-5.975218761	2.30E-09	1.00E-07
PITA_000093489	High quality	AT2G45560	MA_10427075g0010	shade<sun	cytochrome P450 monooxyger	CYTOCHROME P450, FAM	chloroplast-GO:0009507,h	584.4422548	-1.970495171	0.43318414	-4.548862712	5.39E-06	0.000132195
PITA_000093737	High quality	AT2G19130	MA_120873g0010	shade<sun	S-locus lectin protein kinase family protein;(source:Arapo		calmodulin binding-GO:00	79.12225429	-9.934862865	1.21258255	-8.193143514	2.54E-16	4.11E-14

PITA_000093795	High quality	AT1G02130	MA_101924g0020	shade>sun	Belongs to the Rab1 GTPase su	RAS 5 (RA-5)	endoplasmic reticulum to	65.97082072	1.567257609	0.38664558	4.053473519	5.05E-05	0.00096634
PITA_000093932	Low quality	AT3G51510	MA_10427935g0010	shade<sun	transmembrane protein;(source:Araport11)		biological_process_unknov	213.3558984	-0.661057679	0.13468679	-4.908110793	9.20E-07	2.61E-05
PITA_000093955	High quality	AT4G31940	MA_10055697g0010	shade<sun	The gene encodes a cytochrom	CYTOCHROME P450, FAM	cellular response to iron io	875.9110077	-2.49596599	0.15043419	-16.59174673	8.00E-62	3.36E-58
PITA_000094412	High quality	AT1G01490	MA_465568g0010	shade<sun	Heavy metal transport/detoxifi	HEAVY METAL PROTEIN 1	chloroplast-GO:0009507,m	182.3091072	-11.14007873	3.90703641	-2.85128613	0.004354277	0.041091796
PITA_000094425	High quality	AT5G48740	MA_10078675g0010	shade<sun	Leucine-rich repeat protein kinase family protein;(source:		chloroplast-GO:0009507,k	458.1510471	-12.46954792	3.90687281	-3.191695382	0.001414404	0.016913832
PITA_000094894	High quality	AT1G74550	MA_10434848g0020	shade<sun	Encodes a tricoumaroylspermi	CYTOCHROME P450, FAM	chloroplast-GO:0009507,d	114.8267718	-1.463055839	0.47592958	-3.074101534	0.002111376	0.0234164
PITA_000095064	High quality	AT4G16730	MA_10436878g0020	shade<sun	In the Col ecotype, no function	TERPENE SYNTHASE 02 (T	chloroplast-GO:0009507,m	151.5611956	-10.87369516	3.90709151	-2.783066416	0.005384778	0.04807225
PITA_000095068	High quality	AT1G80160	MA_34359g0010	shade<sun	Vicinal oxygen chelate (VOC) su	GLYOXYLASE I 7 (GLYI7)	cytoplasm-GO:0005737	158.56023	-1.315448691	0.29636655	-4.438586952	9.06E-06	0.00021048
PITA_000095401	High quality	AT3G54940	MA_62474g0010	shade>sun	Papain family cysteine protease;(source:Araport11)		cysteine-type endopeptida	18.56378291	1.433852864	0.46129422	3.108326093	0.001881503	0.021326524
PITA_000095507	High quality	AT2G29500	MA_10345610g0010	shade>sun	HSP20-like chaperones superfa	(HSP17.6B)	cytoplasm-GO:0005737,pr	187.6372316	10.84782526	3.90696762	2.776533191	0.005494203	0.048755731
PITA_000095672	Low quality	AT5G06990	MA_33507g0010	shade<sun	MIZU-KUSSEI-like protein (Protein of unknown function, D		chloroplast-GO:0009507,h	305.0237283	-1.555652946	0.47504736	-3.274732326	0.001057621	0.0132111
PITA_000095758	High quality	AT5G04150	MA_68586g0010	shade<sun	Encodes a member of the basi	(BHLH101)	cellular response to iron io	73.2325825	-1.951083282	0.70059815	-2.784882135	0.005354718	0.047956675
PITA_000095804	High quality	AT4G24340	MA_10428395g0010	shade<sun	Phosphorylase superfamily protein;(source:Araport11)		catalytic activity-GO:00038	53.44391828	-9.370592287	1.59982108	-5.857275179	4.71E-09	1.95E-07
PITA_000095886	High quality	AT3G22500	MA_10427927g0010	shade>sun	late embryogenesis abundant (	LATE EMBRYOGENESIS AB	cytoplasm-GO:0005737,cy	41.63169925	0.988651022	0.30665186	3.224017741	0.001264056	0.015378429
PITA_000095920	High quality	AT4G12320	MA_52987g0020	shade<sun	member of CYP706A	CYTOCHROME P450, FAM	chloroplast-GO:0009507,c	398.6418819	-1.819015431	0.43569409	-4.174983027	2.98E-05	0.00060709

Table S3 Gene expression in response to SHADE in Scots pine - latitude 67 versus latitude 56 (north versus south)

PITA_ID	Confidence	Best BLAST Arabidopsis	Best BLAST Conifer spp	Expression	Gene Model Description	Primary Gene Symbol	GO term-GO ID	baseMean	log2FoldChange	lfcSE	stat	pvalue	padj
PITA_000000003	Low quality	AT5G65550	MA_36867g0010	shade67>shade56	UDP-Glycosyltransferase superfamily protein;(sou		chloroplast-GO:00095(	438.2282901	14.44810314	3.28467346	4.398642153	1.09E-05	0.001160375
PITA_000000733	High quality	AT1G32540	MA_22141g0010	shade67<shade56	Encodes a protein with 3 p	LSD ONE LIKE 1 (LOL1)	defense response to ot	138.1974179	-24.90067992	5.724129902	-4.350124883	1.36E-05	0.001386852
PITA_000000744	Low quality	AT1G68620	MA_170302g0010	shade67>shade56	alpha/beta-Hydrolases superfamily protein;(source		cellular response to hy	55.27467224	1.303098964	0.391853849	3.325471897	0.00088269	0.033058902
PITA_000001100	Low quality	AT2G13600	MA_58337g0010	shade67>shade56	Encodes a pentatricopeptic	SLOW GROWTH 2 (SLG	chloroplast-GO:00095(	178.6976925	20.99396683	5.979291783	3.511112619	0.000446235	0.019460651
PITA_000001814	High quality	AT4G02750	MA_10431508g0010	shade67>shade56	Tetratricopeptide repeat (TPR)-like superfamily pr		acquisition of seed long	108.2734179	1.543092411	0.388210786	3.974882891	7.04E-05	0.005274346
PITA_000002561	High quality	AT5G47810	MA_101793g0010	shade67>shade56	phosphofructokinase 2;(so	PHOSPHOFRUCTOKINA	6-phosphofructokinase	48.62507631	16.76628618	3.747804618	4.473628667	7.69E-06	0.000869074
PITA_000002732	High quality	AT3G62550	MA_10426703g0020	shade67<shade56	Adenine nucleotide alpha hydrolases-like superfan		nucleus-GO:0005634,v	50.05161744	-21.4020001	4.026320037	-5.315523828	1.06E-07	4.25E-05
PITA_000002981	Low quality	AT1G18390	MA_214776g0020	shade67>shade56	Serine/Threonine kinase fa	LEAF RUST 10 DISEASE	cellular response to ab	14.54502774	21.2174805	5.997644443	3.5376356	0.000403727	0.018105733
PITA_000003101	Low quality	AT2G31900	MA_10433033g0020	shade67<shade56	Encodes an novel myosin is	MYOSIN-LIKE PROTEIN	actin cytoskeleton-GO:	31.60794472	-21.04444739	5.983875055	-3.516859426	0.000436685	0.019270486
PITA_000003240	Low quality	AT2G42975	MA_163154g0020	shade67<shade56	myosin-G heavy chain-like protein;(source:Araport		biological_process_unl	536.8845491	-1.032195861	0.258667106	-3.990441136	6.60E-05	0.00507124
PITA_000003292	High quality	AT5G17920	MA_10090g0010	shade67>shade56	Encodes a cytosolic cobalai	METHIONINE SYNTHES	5-methyltetrahydropte	542.4300982	1.148392347	0.360526984	3.185315932	0.001445961	0.048177084
PITA_000003310	High quality	AT4G17610	MA_10429620g0010	shade67<shade56	tRNA/rRNA methyltransfer	TRNA METHYLTRANSF	chloroplast-GO:00095(	55.90416141	-9.135901812	2.869606276	-3.183677806	0.001454168	0.048333411
PITA_000003897	High quality	AT5G33406	MA_9990713g0010	shade67<shade56	hAT dimerization domain-containing protein / trar		biological_process_unl	324.4409296	-0.762304434	0.198329838	-3.843619512	0.000121233	0.007666876
PITA_000003909	High quality	AT5G48150	MA_10426489g0020	shade67>shade56	Member of GRAS gene fam	PHYTOCHROME A SIGI	callus formation-GO:1(	32.15456999	19.60397765	6.040487698	3.245429613	0.001172736	0.04091509
PITA_000004166	High quality	AT1G67750	MA_10426822g0020	shade67>shade56	Pectate lyase family protein;(source:Araport11)		extracellular region-GC	787.7581117	13.96141432	1.694864202	8.23748257	1.76E-16	1.14E-12
PITA_000004443	High quality	AT5G09590	MA_101964g0010	shade67>shade56	heat shock protein 70 (Hsc	MITOCHONDRIAL HSO	ATP binding-GO:00055	70.78233243	22.15784374	4.852935525	4.565864027	4.97E-06	0.000577209
PITA_000004498	Low quality	AT5G02860	MA_96691g0010	shade67<shade56	Pentatricopeptide repeat (PPR) superfamily protei		biological_process_unl	387.2381932	-0.563589957	0.153843535	-3.66339709	0.000248892	0.012848156
PITA_000004674	Low quality	AT3G09040	MA_112849g0010	shade67>shade56	Pentatricopeptide repeat (	MITOCHONDRIAL RNA	intracellular membran	556.440113	1.296070101	0.37456815	3.46017167	0.000539831	0.022558345
PITA_000004868	Low quality	AT5G42090	MA_914471g0010	shade67<shade56	Lung seven transmembrane receptor family prote		chloroplast-GO:00095(	90.48113788	-22.18975949	5.979626321	-3.710894009	0.000206529	0.01139722
PITA_000005099	Low quality	AT2G24762	MA_62899g0010	shade67>shade56	Encodes a member of the (	GLUTAMINE DUMPER	chloroplast-GO:00095(	105.3626593	30	6.149562898	4.878395505	1.07E-06	0.000151672
PITA_000005443	High quality	AT1G24020	MA_10429068g0010	shade67<shade56	MLP-like protein 423;(sour	MLP-LIKE PROTEIN 423	abscisic acid binding-G	191.4114787	-1.266494848	0.32922458	-3.846902461	0.000119621	0.00758336
PITA_000005554	High quality	AT3G61440	MA_6291g0010	shade67<shade56	Encodes a cysteine synthas	CYSTEINE SYNTHASE C	chloroplast-GO:00095(	224.1360841	-0.567836421	0.177601078	-3.197257745	0.001387409	0.04683316
PITA_000005779	High quality	AT4G30000	MA_58673g0010	shade67<shade56	Dihydropterin pyrophosphokinase / Dihydropteroa		2-amino-4-hydroxy-6-h	165.5888815	-14.01206732	3.524971721	-3.97508645	7.04E-05	0.005274346
PITA_000005890	Low quality	AT1G54520	MA_10432911g0010	shade67<shade56	FLAP1 is a chloroplast mem	FLUCTUATING-LIGHT- /	chloroplast-GO:00095(	799.1148982	-0.651415403	0.19814962	-3.287492571	0.001010839	0.03644066
PITA_000005956	Low quality	AT5G14270	MA_41068g0020	shade67<shade56	Bromodomain protein that	BROMODOMAIN AND	nucleus-GO:0005634,p	38.3628518	-20.90322013	5.984469642	-3.492911048	0.000477786	0.020492746
PITA_000006033	High quality	AT5G01130	MA_72874g0010	shade67>shade56	hypothetical protein (DUF674);(source:Araport11)		biological_process_unl	230.0304958	22.28515807	5.635838805	3.954186563	7.68E-05	0.005575682
PITA_000006141	High quality	AT1G76570	MA_10435703g0010	shade67<shade56	Chlorophyll A-B binding far	LIGHT-HARVESTING CC	chlorophyll binding-GC	204.6702581	-1.188298405	0.330602356	-3.59434343	0.000325211	0.015538373
PITA_000006272	High quality	AT1G12680	MA_10341791g0010	shade67<shade56	phosphoenolpyruvate carb	PHOSPHOENOLPYRUV.	calcium-dependent pro	49.9724367	-30	6.102463821	-4.916047171	8.83E-07	0.000151672
PITA_000006546	Low quality	AT4G14690	MA_10432802g0010	shade67>shade56	Encodes an early light-indu	EARLY LIGHT-INDUCIBI	cellular response to blu	85.67537118	21.41659299	6.036547437	3.547821534	0.000388431	0.017558447
PITA_000007446	High quality	AT2G43180	MA_10425835g0010	shade67>shade56	Phosphoenolpyruvate carboxylase family protein;(		catalytic activity-GO:00	246.8174243	26.38783395	6.036722964	4.37121831	1.24E-05	0.001284578
PITA_000008216	High quality	AT2G39210	MA_10432157g0010	shade67<shade56	Major facilitator superfami	PICLORAM RESISTANT:	chloroplast-GO:00095(	254.1742931	-0.839178078	0.244436365	-3.433114699	0.00059669	0.024179728
PITA_000009135	High quality	AT5G08370	MA_41308g0010	shade67>shade56	Member of Glycoside Hydr	ALPHA-GALACTOSIDAS	alpha-galactosidase act	187.0767475	3.385407765	0.842718376	4.017246876	5.89E-05	0.004609824
PITA_000009354	Low quality	AT4G21970	MA_118009g0010	shade67>shade56	senescence regulator (Protein of unknown functio		biological_process_unl	350.1577984	10.70347334	3.287114827	3.256190885	0.001129178	0.039715287
PITA_000009655	High quality	AT4G02780	MA_10433916g0010	shade67>shade56	Catalyzes the conversion o	GA REQUIRING 1 (GA1	chloroplast-GO:00095(	118.6904836	22.44487107	6.037730602	3.717435002	0.000201256	0.011153602
PITA_000009750	Low quality	AT1G49230	MA_85088g0010	shade67>shade56	RING/U-box superfamily pı	ARABIDOPSIS TOXICOS	protein ubiquitination-	57.47702003	22.8053985	6.038015941	3.776968912	0.000158749	0.009318591
PITA_000010201	High quality	AT5G05270	MA_502949g0010	shade67>shade56	Chalcone-flavanone isomei	CHALCONE ISOMERASI	chalcone isomerase act	309.2508778	0.908570506	0.2822606	3.218906595	0.001286804	0.043997036
PITA_000010492	High quality	AT1G07900	MA_170369g0010	shade67<shade56	LOB domain-containing pro	LOB DOMAIN-CONTAII	biological_process_unl	39.37185821	-30	6.093769543	-4.923061135	8.52E-07	0.000151672
PITA_000010577	High quality	AT2G02990	MA_95383g0020	shade67<shade56	Encodes a member of the ı	RIBONUCLEASE 1 (RNS	aging-GO:0007568,ant	58.04784309	-30	6.097293479	-4.920215847	8.64E-07	0.000151672
PITA_000010939	Low quality	AT4G04630	MA_93306g0010	shade67<shade56	senescence regulator (Protein of unknown functio		biological_process_unl	71.6897664	-19.89129615	4.888103136	-4.069328245	4.71E-05	0.003878145
PITA_000011540	High quality	AT3G07370	MA_10428939g0020	shade67<shade56	Encodes AtCHIP, a new clas	CARBOXYL TERMINUS	cellular response to mi	190.2730511	-1.053789288	0.245013545	-4.300942984	1.70E-05	0.001687226
PITA_000011915	Low quality	AT5G63450	MA_18375g0010	shade67>shade56	cytochrome P450, family 9	CYTOCHROME P450, F.	chloroplast-GO:00095(	195.1294714	24.34256185	4.587291473	5.306521722	1.12E-07	4.31E-05
PITA_000012121	High quality	AT3G12770	MA_10287590g0010	shade67<shade56	Encodes a pentatricopeptic	MITOCHONDRIAL EDIT	chloroplast-GO:00095(	299.6849046	-0.983214734	0.229509008	-4.283991914	1.84E-05	0.001780351
PITA_000013320	Low quality	AT4G13690	MA_10436070g0040	shade67>shade56	RNA-binding protein;(source:Araport11)		biological_process_unl	171.5603436	1.233733779	0.362984295	3.398862695	0.000676667	0.02677005
PITA_000013409	High quality	AT4G28660	MA_18349g0010	shade67<shade56	Similar to PsbW subunit of	PHOTOSYSTEM II REA(	chloroplast-GO:00095(	674.9673008	-0.58788994	0.17611064	-3.338185251	0.000843275	0.031951029
PITA_000013780	High quality	AT1G70700	MA_54375g0010	shade67>shade56	JAZ9 is a protein presumed	(TIFY7)	nucleus-GO:0005634,p	78.29361189	21.24552178	6.034984375	3.520393833	0.000430906	0.019080274
PITA_000014004	High quality	AT2G41790	MA_7967391g0010	shade67<shade56	Insulinase (Peptidase family M16) family protein;(s		cytoplasm-GO:000573	69.47537375	-9.268336759	2.710336809	-3.41962546	0.000627074	0.025191514
PITA_000014289	High quality	AT5G09810	MA_10427661g0030	shade67>shade56	Member of Actin gene fam	ACTIN 7 (ACT7)	cell division-GO:00513(	327.0845029	25.09276269	4.845453975	5.178619552	2.24E-07	7.45E-05
PITA_000014306	High quality	AT1G28440	MA_61409g0010	shade67<shade56	HAESA-like 1;(source:Arap	HAESA-LIKE 1 (HSL1)	kinase activity-GO:001(	22.75591107	-20.07882154	5.987331725	-3.353550874	0.000797817	0.030775716
PITA_000014330	High quality	AT2G46700	MA_3361g0010	shade67>shade56	CDPK-related kinase 3;(sou	CDPK-RELATED KINASE	ATP binding-GO:00055	29.12994796	21.12091526	6.04015144	3.496752601	0.000470958	0.020334137
PITA_000014772	High quality	AT2G26330	MA_5825101g0010	shade67>shade56	Homologous to receptor p	ERECTA (ER)	cellular heat acclimatic	372.8877746	30	6.149303362	4.878601402	1.07E-06	0.000151672
PITA_000015187	High quality	AT1G70520	MA_10435854g0010	shade67>shade56	Encodes a cysteine-rich receptor-like protein kinas		kinase activity-GO:001(	21.10576591	22.43116761	5.69031473	3.941990677	8.08E-05	0.005802112
PITA_000015200	Low quality	AT3G62310	MA_34468g0010	shade67>shade56	RNA helicase family protein;(source:Araport11)		cytosol-GO:0005829,in	116.216452	22.14257532	5.978717445	3.703566112	0.00021259	0.011549807
PITA_000015242	Low quality	AT4G26090	MA_162919g0010	shade67>shade56	Encodes a plasma membra	RESISTANT TO P. SYRII	ADP binding-GO:00435	232.2442401	2.82268695	0.86438033	3.265561293	0.001092474	0.038791767
PITA_000015505	High quality	AT3G52970	MA_10432446g0010	shade67>shade56	member of CYP76G	CYTOCHROME P450, F.	chloroplast-GO:00095(	77.19106141	3.891570491	1.009079304	3.856555651	0.000114996	0.007416809
PITA_000015743	High quality	AT5G27660	MA_10433666g0010	shade67<shade56	Encodes a protein with sim	DEGRADATION OF PER	cellular response to he	81.42570885	-1.113912255	0.282184006	-3.947467723	7.90E-05	0.005702512
PITA_000016189	High quality	AT5G01320	MA_12415g0010	shade67<shade56	Thiamine pyrophosphate dependent pyruvate dec		carboxy-lyase activity-(	77.31912464	-22.2841332	5.245413346	-4.248308328	2.15E-05	0.002049148
PITA_000016560	High quality	AT4G26200	MA_117710g0010	shade67>shade56	Member of a family of prot	1-AMINO-CYCLOPROP/	1-aminocyclopropane-	67.34756805	30	6.150164237	4.877918514	1.07E-06	0.000151672
PITA_000016626	High quality	AT2G25790	MA_10429487g0020	shade67>shade56	Leucine-rich receptor-like p	STERILITY-REGULATIN(	endoplasmic reticulum	62.57468207	19.97358679	6.037724503	3.308131529	0.000939207	0.034528805
PITA_000016896	High quality	AT4G28250	MA_21214g0010	shade67>shade56	putative beta-expansin/alle	EXPANSIN B3 (EXPB3)	extracellular region-GC	69.56701797	22.02428012	6.037043806	3.648189549	0.000264095	0.013380802
PITA_000016905	High quality	AT4G02750	MA_10431508g0010	shade67<shade56	Tetratricopeptide repeat (TPR)-like superfamily pr		acquisition of seed long	229.8989314	-1.613351116	0.455543134	-3.541599016	0.00039771	0.017884545
PITA_000017096	High quality	AT2G26560	MA_468579g0010	shade67>shade56	Encodes a lipid acyl hydrola	PHOSPHOLIPASE A 2A	acylglycerol lipase activ	96.13579878	20.60972412	3.689961825	5.585348873	2.33E-08	1.10E-05
PITA_000017138	High quality	AT5G36110	MA_398469g0010	shade67>shade56	Encodes a member of the (	CYTOCHROME P450, F.	extracellular region-GC	543.6180932	22.65024427	6.035182916	3.7530336	0.000174707	0.01004645
PITA_000017385	Low quality	AT5G44310	MA_41035g0010	shade67>shade56	Late embryogenesis abundant protein (LEA) family		biological_process_unl	96.18418494	30	6.150793027	4.87741985	1.07E-06	0.000151672
PITA_000017574	High quality	AT2G33170	MA_10432079g0010	shade67>shade56	Leucine-rich repeat receptor-like protein kinase fa		plasma membrane-GO	36.14215019	19.740158	5.933967807	3.326637192	0.000879007	0.033016127
PITA_000017588	High quality	AT1G15520	MA_10425791g0020	shade67<shade56	ABC transporter family inv	ATP-BINDING CASSETT	abscisic acid transport-	92.97369152	-30	6.095101301	-4.921985464	8.57E-07	0.000151672
PITA_000017726	Low quality	AT1G60500	MA_10431097g0010	shade67>shade56	Dynamin related protein 4(	DYNAMIN RELATED PR	axon-GO:0030424,cytc	59.42345321	7.518317628	1.640077831	4.584122465	4.56E-06	0.000533769
PITA_000018066	High quality	AT5G17820	MA_4883828g0010	shade67>shade56	Peroxidase superfamily pro	PEROXIDASE 57 (PER5)	cell wall-GO:0005618,e	33.98377969	30	6.151291022	4.877024984	1.08E-06	0.000151672
PITA_000018272	High quality	AT1G69500	MA_10428090g0010	shade67>shade56	Encodes a cytochrome P45	CYTOCHROME P450, F.	alkane 1-monooxygena	107.1275446	30	6.15292716	4.875728124	1.08E-06	0.000151672
PITA_000018661	Low quality	AT3G47520	MA_10431009g0010	shade67>shade56	Encodes a protein with NA	MALATE DEHYDROGEI	apoplast-GO:0048046,	293.0122311	24.76306033	4.701503455	5.267051395	1.39E-07	5.22E-05
PITA_000019412	High quality	AT3G48930	MA_19350g0010	shade67<shade56	Nucleic acid-binding, OB-fc	EMBRYO DEFECTIVE 1(	cell wall-GO:0005618,c	86.91091791	-19.54657759	5.979685627	-3.268830305	0.00107993	0.038536901
PITA_000019615	High quality	AT5G13930	MA_10426264g0020	shade67>shade56	Encodes chalcone synthase	TRANSPARENT TESTA ‹	auxin polar transport-(	20.88692372	11.39337689	2.999368903	3.79859139	0.000145521	0.0087555
PITA_000020294	Low quality	AT5G56600	MA_17008g0010	shade67>shade56	Encodes profilin3, a low-m	PROFILIN 3 (PRF3)	actin filament polymer	112.960939	21.55944504	3.785203871	5.695715681	1.23E-08	6.52E-06
PITA_000020499	High quality	AT2G19130	MA_470235g0010	shade67>shade56	S-locus lectin protein kinase family protein;(source		calmodulin binding-GC	43.3710899	30	6.150162658	4.877919767	1.07E-06	0.000151672
PITA_000020651	High quality	AT2G41480	MA_10426026g0010	shade67<shade56	Encodes a cationic cell-wal	PEROXIDASE 25 (PRX2!	cellular oxidant detoxif	69.3576815	-23.52782515	5.979271841	-3.934898057	8.32E-05	0.005894732
PITA_000020691	High quality	AT3G49260	MA_6756408g0010	shade67>shade56	IQ-domain 21;(source:Arap	IQ-DOMAIN 21 (iqd21)	biological_process_unl	100.9482946	22.85884342	6.036636388	3.78668549	0.00015267	0.009098542
PITA_000021595	High quality	AT5G64260	MA_10432600g0020	shade67<shade56	EXORDIUM like 2;(source:/	EXORDIUM LIKE 2 (EXL	cell wall-GO:0005618,c	10.64800592	-30	6.099095689	-4.918761982	8.71E-07	0.000151672
PITA_000022236	High quality	AT1G76490	MA_363692g0010	shade67>shade56	Encodes a 3-hydroxy-3-me	HYDROXY METHYLGLU	coenzyme A metabolic	200.1074846	23.44790311	4.869589346	4.81517053	1.47E-06	0.000197049
PITA_000022502	High quality	AT1G19180	MA_10430554g0020	shade67>shade56	JAZ1 is a nuclear-localized	JASMONATE-ZIM-DOM	defense response to ba	66.79189089	22.13935621	5.369523681	4.123150866	3.74E-05	0.003292832
PITA_000022505	High quality	AT1G14190	MA_137064g0010	shade67<shade56	Glucose-methanol-choline (GMC) oxidoreductase		chloroplast-GO:00095(	164.0946345	-0.857513346	0.232883333	-3.682158514	0.000231268	0.0121658
PITA_000022622	High quality	AT3G53900	MA_6556215g0010	shade67<shade56	Encodes UPP, a plastidial u	URACIL PHOSPHORIBO	cellular response to ph	160.2214017	-0.700505871	0.202724556	-3.455456428	0.000549362	0.022773551
PITA_000023020	High quality	AT4G31540	MA_482507g0010	shade67>shade56	A member of EXO70 gene	EXOCYST SUBUNIT EX(	cytoplasm-GO:000573	148.3051389	1.483795662	0.405767972	3.656758948	0.000255424	0.013043203
PITA_000023032	High quality	AT5G49720	MA_102312g0010	shade67>shade56	Encodes a membrane-bour	GLYCOSYL HYDROLASE	cell plate-GO:0009504,	898.7332018	12.49876122	3.155215666	3.961301712	7.45E-05	0.005467988
PITA_000023127	High quality	AT3G03380	MA_10435811g0020	shade67<shade56	Encodes a putative DegP p	DEGRADATION OF PER	chloroplast-GO:00095(	53.12595204	-1.333346707	0.405416009	-3.288835855	0.001006027	0.03644066
PITA_000023195	High quality	AT3G62600	MA_44157g0010	shade67>shade56	J domain protein localized	(ATERDJ3B)	cytoplasm-GO:000573	245.8676768	24.39358136	5.97830143	4.080353198	4.50E-05	0.003746124
PITA_000023653	High quality	AT4G16310	MA_10435906g0020	shade67>shade56	FAD-dependent lysine-spe	LSD1-LIKE 3 (LDL3)	mitochondrion-GO:00(	114.1756415	17.80921087	5.255556832	3.388643952	0.000702391	0.027703429
PITA_000023696	High quality	AT1G22900	MA_10434995g0010	shade67>shade56	Disease resistance-responsive (dirigent-like protei		extracellular region-GC	173.7855029	1.625170422	0.357719144	4.543146348	5.54E-06	0.00063458
PITA_000024299	High quality	AT5G38890	MA_63326g0010	shade67<shade56	Nucleic acid-binding, OB-fold-like protein;(source:		cytoplasm-GO:000573	194.5915639	-11.67008985	3.083517544	-3.784667895	0.000153914	0.009102912
PITA_000024478	Low quality	AT4G33495	MA_127513g0010	shade67>shade56	A member of the RPD gene	ROOT PRIMORDIUM D	chloroplast-GO:00095(	73.70573409	30	6.149613569	4.878355308	1.07E-06	0.000151672
PITA_000024824	Low quality	AT2G34640	MA_8815984g0010	shade67<shade56	Present in transcriptionally	PLASTID TRANSCRIPTI(	chloroplast-GO:00095(	222.5662224	-0.810916941	0.236825288	-3.42411466	0.000616806	0.024855844
PITA_000024916	Low quality	AT4G39230	MA_10427035g0010	shade67<shade56	encodes a protein whose s	PHENYLCOUMARAN BI	cytoplasm-GO:000573	697.7105743	-0.758774779	0.231487668	-3.277819435	0.001046123	0.037608335
PITA_000025034	High quality	AT1G05670	MA_68479g0030	shade67>shade56	Pentatricopeptide repeat (PPR-like) superfamily pı		endoplasmic reticulum	106.8183909	21.90195541	6.036261663	3.628397282	0.000285186	0.014029671
PITA_000025189	High quality	AT3G61770	MA_10431963g0010	shade67<shade56	Acid phosphatase/vanadiu	VACUOLAR PROTEIN S(	biological_process_unl	94.95576759	-0.725654132	0.217562534	-3.335381869	0.000851823	0.032138679
PITA_000025760	High quality	AT1G62300	MA_14480g0020	shade67<shade56	Encodes a transcription fac	(WRKY6)	cellular response to bo	37.24176801	-19.97822465	5.981121142	-3.340214012	0.000837139	0.031811264
PITA_000026024	Low quality	AT5G11970	MA_69352g0010	shade67>shade56	ABC family ABC transporter, putative (DUF3511);(s		biological_process_unl	106.1172467	23.48316391	6.037183249	3.88975503	0.000100345	0.006687639
PITA_000026333	High quality	AT5G44640	MA_9186460g0010	shade67>shade56	beta glucosidase 13;(sourc	BETA GLUCOSIDASE 13	beta-glucosidase activi	178.5356228	9.81454723	3.038286947	3.230289766	0.001236648	0.042686532
PITA_000026395	High quality	AT3G28345	MA_107576g0010	shade67>shade56	Encodes an ATP-binding ca	ATP-BINDING CASSETT	ATPase activity-GO:00:	102.0404499	8.192908588	1.866498045	4.389454684	1.14E-05	0.001193752
PITA_000026472	High quality	AT4G27670	MA_8682g0010	shade67>shade56	Encodes Hsp21, a chloropla	HEAT SHOCK PROTEIN	chaperone complex-G(	164.4253136	21.25232711	4.21188239	5.045802599	4.52E-07	0.000133393
PITA_000026647	Low quality	AT4G18390	MA_4774g0010	shade67<shade56	TEOSINTE BRANCHED 1, cy	TEOSINTE BRANCHED :	cell differentiation-GO	113.2557907	-1.014073866	0.257448378	-3.938940587	8.18E-05	0.005844072
PITA_000026747	High quality	AT2G45590	MA_68968g0010	shade67>shade56	Protein kinase superfamily protein;(source:Arapor		nucleus-GO:0005634,p	41.61794956	20.76879576	3.967844509	5.234276623	1.66E-07	6.15E-05
PITA_000027019	Low quality	AT4G37030	MA_10435180g0010	shade67<shade56	membrane protein;(source:Araport11)		biological_process_unl	15.51564647	-20.77458291	5.987608897	-3.469595838	0.000521242	0.02192253
PITA_000027066	High quality	AT5G20900	MA_14247g0030	shade67>shade56	jasmonate-zim-domain pro	JASMONATE-ZIM-DOM	nucleus-GO:0005634,p	56.32587788	1.494555788	0.446386731	3.348118764	0.000813621	0.031184421
PITA_000027981	High quality	AT3G26380	MA_10434513g0010	shade67<shade56	APSE is a member of the G	ARAPASE (APSE)	arabinogalactan protei	2662.576814	-1.003274057	0.14871164	-6.74643933	1.52E-11	2.63E-08
PITA_000028673	High quality	AT5G63020	MA_10428204g0010	shade67>shade56	Nucleotide binding leucine	SUPPRESSORS OF TOP	ADP binding-GO:00435	108.3498612	3.888066337	1.037338924	3.748115727	0.000178168	0.010222834
PITA_000028719	High quality	AT3G47520	MA_10431009g0010	shade67<shade56	Encodes a protein with NA	MALATE DEHYDROGEI	apoplast-GO:0048046,	161.2182233	-24.64008598	5.73253545	-4.29828759	1.72E-05	0.001694627
PITA_000028925	High quality	AT4G20140	MA_10213762g0010	shade67>shade56	Encodes GASSHO1 (GSO1),	GASSHO1 (GSO1)	Casparian strip-GO:004	155.2963044	10.83038575	3.026790581	3.578174791	0.000346002	0.016233366
PITA_000029873	High quality	AT2G24130	MA_11052g0010	shade67<shade56	Leucine-rich receptor-like protein kinase family pro		plasma membrane-GO	75.47006056	-23.53138346	4.244525414	-5.543937464	2.96E-08	1.35E-05
PITA_000030464	High quality	AT1G09230	MA_181629g0010	shade67<shade56	Encodes a U12-type splicec	U11/U12-65K (U11/U1	inflorescence developr	145.6401234	-1.011976589	0.263734519	-3.837103282	0.000124494	0.007797232
PITA_000031241	High quality	AT2G26180	MA_83892g0010	shade67<shade56	Transient Expression of Pro	IQ-DOMAIN 6 (IQD6)	microtubule-GO:00058	35.37099071	-19.22886756	5.192264557	-3.703368222	0.000212756	0.011549807
PITA_000031268	Low quality	AT4G21230	MA_3950g0020	shade67>shade56	Encodes a cysteine-rich rec	CYSTEINE-RICH RLK (RE	extracellular region-GC	86.90985441	1.2096244	0.361656347	3.344679027	0.000823778	0.03139538
PITA_000031809	Low quality	AT4G28100	MA_141029g0010	shade67<shade56	transmembrane protein;(source:Araport11)		anchored component (	320.73074	-0.438388663	0.130729049	-3.353414296	0.000798211	0.030775716
PITA_000032491	High quality	AT5G50375	MA_9262292g0010	shade67>shade56	Converts pentacyclic cyclop	CYCLOPROPYL ISOMER	cycloeucalenol cycloisc	250.9296985	1.571179986	0.474669614	3.310049643	0.000932794	0.034390338
PITA_000032720	High quality	AT2G02010	MA_1610g0010	shade67>shade56	glutamate decarboxylase 4	GLUTAMATE DECARBC	chloroplast-GO:00095(	154.7817164	0.833465568	0.240713843	3.462474601	0.000535232	0.022438325
PITA_000032983	Low quality	AT1G62870	MA_10428390g0010	shade67>shade56	hypothetical protein;(source:Araport11)		chloroplast-GO:00095(	63.49173211	22.90149372	6.038022712	3.792879692	0.00014891	0.008897648
PITA_000033461	Low quality	AT1G01250	MA_115502g0010	shade67>shade56	encodes a member of the DREB subfamily A-4 of E		DNA-binding transcript	170.7213448	23.03975259	5.714923795	4.031506529	5.54E-05	0.004424377
PITA_000033577	High quality	AT2G46080	MA_7578826g0010	shade67<shade56	Encodes a protein related t	BYPASS 2 (BPS2)	embryo development (	60.23184275	-25.37688421	3.75831812	-6.752191644	1.46E-11	2.63E-08
PITA_000033657	High quality	AT5G07280	MA_177039g0010	shade67>shade56	Encodes EMS1 (EXCESS MI	EXCESS MICROSPORO(	anther wall tapetum ce	51.14800956	21.21034842	5.54390592	3.825885347	0.000130303	0.008036537
PITA_000033675	High quality	AT1G53280	MA_10433067g0010	shade67<shade56	Encodes a homolog of anin	DJ-1 HOMOLOG B (DJ1	chloroplast-GO:00095(	375.6852421	-0.667920004	0.172663633	-3.868330544	0.000109583	0.007208183
PITA_000033769	High quality	AT1G52540	MA_10329547g0010	shade67>shade56	Protein kinase superfamily protein;(source:Arapor		ATP binding-GO:00055	76.15075507	19.22074732	3.565727109	5.390414559	7.03E-08	2.90E-05
PITA_000033851	High quality	AT1G57560	MA_197412g0010	shade67>shade56	Member of the R2R3 facto	MYB DOMAIN PROTEII	DNA-binding transcript	23.47773731	30	6.151622148	4.876762467	1.08E-06	0.000151672
PITA_000034037	High quality	AT5G22260	MA_115384g0010	shade67>shade56	Sporophytic factor controll	MALE STERILITY 1 (MS	anther wall tapetum m	86.15682037	21.98399923	5.978729384	3.677035339	0.00023596	0.012365084
PITA_000034222	High quality	AT4G01210	MA_10431531g0010	shade67<shade56	glycosyl transferase family 1 protein;(source:Arap(		endosome-GO:000576	126.8228491	-0.726732396	0.209087532	-3.475732811	0.00050946	0.021566569
PITA_000034250	High quality	AT2G04570	MA_166543g0010	shade67>shade56	GDSL-motif esterase/acyltransferase/lipase. Enzyı		acyl-[acyl-carrier-prote	693.0275158	11.27509293	3.207775106	3.514926251	0.000439876	0.019335762
PITA_000034300	High quality	AT5G17680	MA_135183g0010	shade67>shade56	disease resistance protein (TIR-NBS-LRR class);(sou		ADP binding-GO:00435	92.87473841	10.40280606	3.105936977	3.34932941	0.000810074	0.031131151
PITA_000034498	High quality	AT3G17390	MA_141759g0010	shade67<shade56	S-adenosylmethionine synt	METHIONINE OVER-AC	cell wall-GO:0005618,c	213.708504	-30	6.093644883	-4.923161847	8.52E-07	0.000151672
PITA_000034564	Low quality	AT1G55270	MA_100608g0010	shade67<shade56	SAGL1 is a member of a sm	SMALL AND GLOSSY LE	nucleus-GO:0005634,p	27.04708593	-18.42644408	5.003286617	-3.682867981	0.000230625	0.0121658
PITA_000034721	High quality	AT4G39350	MA_82434g0010	shade67<shade56	Encodes a cellulose syntha	CELLULOSE SYNTHASE	cellulose biosynthetic p	127.2966014	-3.642341872	0.606917229	-6.001381567	1.96E-09	1.50E-06
PITA_000034759	High quality	AT5G62040	MA_112333g0010	shade67<shade56	BFT is a member of The FL(	BROTHER OF FT AND T	cytoplasm-GO:000573	49.60228842	-30	6.105121657	-4.913906993	8.93E-07	0.000151672
PITA_000034868	High quality	AT5G40380	MA_27101g0010	shade67>shade56	Encodes a cysteine-rich rec	CYSTEINE-RICH RLK (RE	chloroplast-GO:00095(	123.1984822	24.06452137	6.036435931	3.986544651	6.70E-05	0.005125203

PITA_000035185	High quality	AT1G11910	MA_101119g0010	shade67<shade56	Encodes an aspartic protei	ASPARTIC PROTEINASE	cytosol-GO:0005829,e	170.6442096	-24.76461823	5.978161092	-4.142514369	3.44E-05	0.003057788
PITA_000035534	High quality	AT5G04770	MA_84286g0010	shade67>shade56	Encodes a member of the	CATIONIC AMINO ACID	amino acid transmemb	177.8645482	13.63720278	3.827134074	3.563293713	0.000366231	0.016968325
PITA_000036089	High quality	AT2G37050	MA_101110g0010	shade67>shade56	Leucine-rich repeat protein kinase family protein;(		ATP binding-GO:00055	558.7341974	22.1489401	5.154547106	4.296971129	1.73E-05	0.00169496
PITA_000036322	High quality	AT3G20390	MA_429896g0010	shade67>shade56	Encodes a plastidial RidA (I	REACTIVE INTERMEDIA	2-iminobutanoate/2-in	46.88410511	30	6.150486328	4.877663066	1.07E-06	0.000151672
PITA_000036478	High quality	AT5G01410	MA_10426564g0030	shade67>shade56	Encodes a protein predicte	REDUCED SUGAR RESP	amine-lyase activity-G(	259.1846581	21.30677216	4.847961092	4.394996527	1.11E-05	0.001175205
PITA_000036918	High quality	AT3G21280	MA_20674g0010	shade67>shade56	Encodes a ubiquitin-specifi	UBIQUITIN-SPECIFIC PI	nucleus-GO:0005634,p	40.26412393	21.07363039	5.318896341	3.962030662	7.43E-05	0.005467988
PITA_000037272	High quality	AT2G22590	MA_940843g0010	shade67>shade56	UDP-Glycosyltransferase superfamily protein;(sou		intracellular membran	74.8017297	7.786633639	2.109977179	3.69038761	0.000223913	0.011926099
PITA_000037323	High quality	AT2G21270	MA_122976g0010	shade67>shade56	ubiquitin fusion degradatic	UBIQUITIN FUSION DE	chloroplast-GO:00095(	188.4852287	20.87490398	6.035878775	3.458469721	0.000543253	0.022628591
PITA_000037580	High quality	AT1G08250	MA_7947g0030	shade67>shade56	Encodes a plastid-localized	AROGENATE DEHYDRA	arogenate dehydratase	767.1859835	1.179437758	0.30388958	3.881139193	0.000103968	0.006893736
PITA_000037585	Low quality	AT2G31510	MA_162954g0010	shade67>shade56	IBR domain-containing pro	ARIADNE 7 (ARI7)	cytoplasm-GO:000573	95.69637323	30	6.149816689	4.878194183	1.07E-06	0.000151672
PITA_000037914	Low quality	AT5G53490	MA_10429821g0010	shade67<shade56	thylakoid lumenal 17.4 kDa	THYLAKOID LUMENAL	biological_process_unl	157.1271524	-0.756969293	0.204180267	-3.707357746	0.000209433	0.011460173
PITA_000038327	High quality	AT2G46080	MA_8873395g0010	shade67>shade56	Encodes a protein related t	BYPASS 2 (BPS2)	embryo development	366.873124	26.4445803	5.977960185	4.423679564	9.70E-06	0.001059708
PITA_000038347	Low quality	AT2G30780	MA_186059g0010	shade67>shade56	Tetratricopeptide repeat (TPR)-like superfamily pr		biological_process_unl	73.02517002	3.193526995	0.621912297	5.135011817	2.82E-07	8.83E-05
PITA_000038689	Low quality	AT2G38820	MA_132127g0010	shade67>shade56	DNA-directed RNA polymerase subunit beta-beta		biological_process_unl	183.6020265	10.82811927	1.787179757	6.058774575	1.37E-09	1.17E-06
PITA_000038987	High quality	AT2G02160	MA_126678g0010	shade67>shade56	CCCH-type zinc finger fami	(ATC3H17)	mRNA binding-GO:000	71.31429336	30	6.14993147	4.878103137	1.07E-06	0.000151672
PITA_000039093	High quality	AT5G01880	MA_41514g0010	shade67>shade56	RING/U-box superfamily p	DAF-LIKE GENE 2 (DAF	protein ubiquitination-	22.03381049	22.5909854	6.046988266	3.735906934	0.00018704	0.010545639
PITA_000039730	High quality	AT5G23530	MA_131537g0010	shade67>shade56	carboxyesterase 18;(sourc	CARBOXYESTERASE 18	carboxylic ester hydrol	2150.686721	3.375766738	0.96140327	3.511291093	0.000445936	0.019460651
PITA_000040257	High quality	AT5G13870	MA_10251013g0010	shade67>shade56	EXGT-A4, endoxyloglucan t	XYLOGLUCAN ENDOTR	cell wall biogenesis-GC	250.9583327	3.924813936	1.082869644	3.624456514	0.00028957	0.014114882
PITA_000040507	High quality	AT5G13170	MA_10429987g0020	shade67>shade56	Encodes a member of the	SENESCENCE-ASSOCIA	carbohydrate transpor	187.3874819	21.7146021	5.051017993	4.299054592	1.72E-05	0.001694627
PITA_000040627	High quality	AT5G08250	MA_215531g0010	shade67>shade56	Cytochrome P450 superfamily protein;(source:Ara		cellular_component_u	74.17321734	19.33881156	6.0106267	3.217436803	0.001293415	0.044118697
PITA_000040888	Low quality	AT2G15490	MA_10433869g0010	shade67<shade56	UDP-glycosyltransferase 73	UDP-GLYCOSYLTRANSI	chloroplast-GO:00095(	83.84185423	-1.691224366	0.419608285	-4.030483732	5.57E-05	0.004424377
PITA_000040988	High quality	AT3G21360	MA_867753g0010	shade67<shade56	2-oxoglutarate (2OG) and Fe(II)-dependent oxyger		cytosol-GO:0005829,n	70.99123184	-8.379727826	1.302592154	-6.433117073	1.25E-10	1.71E-07
PITA_000040998	High quality	AT5G35770	MA_82060g0010	shade67>shade56	A recessive mutation in the	STERILE APETALA (SAP	DNA-binding transcript	36.47332142	2.279348822	0.642460896	3.547840556	0.000388403	0.017558447
PITA_000041197	High quality	AT1G75450	MA_138486g0010	shade67<shade56	This gene used to be called	CYTOKININ OXIDASE 5	cytokinin catabolic pro	66.7617274	-1.351708029	0.380823856	-3.549431073	0.000386065	0.017541697
PITA_000041846	High quality	AT5G52640	MA_10137763g0010	shade67<shade56	Encodes a cytosolic heat sh	HEAT SHOCK PROTEIN	cell wall-GO:0005618,c	28.93475363	-21.60536642	5.982548493	-3.611398462	0.00030455	0.014659022
PITA_000042229	High quality	AT2G47000	MA_138894g0010	shade67<shade56	Encodes an auxin efflux tra	ATP-BINDING CASSETT	ABC-type xenobiotic tr	464.0837569	-1.351187327	0.323720298	-4.173934517	2.99E-05	0.002730381
PITA_000042619	High quality	AT3G24140	MA_141469g0010	shade67>shade56	Encodes a basic helix-loop-	FAMA (FMA)	DNA-binding transcript	68.9156729	0.992065042	0.272786961	3.636775889	0.000276072	0.013746478
PITA_000042736	High quality	AT1G23010	MA_10432422g0010	shade67<shade56	Encodes a protein with mu	LOW PHOSPHATE ROO	cellular response to ph	112.1783038	-23.37193491	5.978883192	-3.909080368	9.26E-05	0.0063205
PITA_000042898	Low quality	AT1G34000	MA_17658g0010	shade67<shade56	Encodes a novel member c	ONE-HELIX PROTEIN 2	chloroplast-GO:00095(	274.1735693	-0.633459866	0.169885899	-3.728737161	0.000192442	0.010780056
PITA_000043219	High quality	AT4G25160	MA_43509g0010	shade67>shade56	Encodes a U-box domain-c	(PUB35)	nucleus-GO:0005634,p	268.7429804	21.90665304	6.035530356	3.629615254	0.000283844	0.013999379
PITA_000043567	High quality	AT5G46220	MA_33920g0010	shade67>shade56	Encodes a protein with alk	TURGOR REGULATION	chloroplast-GO:00095(	466.3622546	24.90392319	5.977458324	4.166306454	3.10E-05	0.002813448
PITA_000043671	Low quality	AT4G26090	MA_10435822g0010	shade67<shade56	Encodes a plasma membra	RESISTANT TO P. SYRIN	ADP binding-GO:00435	291.2263012	-25.23886905	5.977736586	-4.222144734	2.42E-05	0.002262507
PITA_000043744	High quality	AT1G22160	MA_6300g0010	shade67>shade56	senescence-associated family protein (DUF581);(s		cytosol-GO:0005829,ki	238.7982891	0.805167077	0.202739745	3.971431839	7.14E-05	0.005335974
PITA_000043761	High quality	AT1G44110	MA_103138g0010	shade67>shade56	Cyclin A1;(source:Araport1	CYCLIN A1;1 (CYCA1;1)	cyclin-dependent prot	173.3833707	22.92452821	5.97794276	3.834852412	0.00012564	0.007850066
PITA_000043773	High quality	AT5G46330	MA_85663g0010	shade67<shade56	Encodes a leucine-rich rep	FLAGELLIN-SENSITIVE	defense response by c	19.09285717	-30	6.098866294	-4.91894699	8.70E-07	0.000151672
PITA_000044373	High quality	AT1G73340	MA_266173g0010	shade67>shade56	ADTO1 is required for the	ABIETANE DITERPENE	brassinosteroid biosyn	188.7555505	2.305695357	0.699259322	3.297339462	0.000976055	0.035631475
PITA_000044762	High quality	AT3G22590	MA_162063g0010	shade67>shade56	Encodes PLANT HOMOLOG	PLANT HOMOLOGOUS	Cdc73/Paf1 complex-G	66.61607181	22.79006189	6.03745844	3.774777436	0.000160151	0.009375302
PITA_000044855	High quality	AT2G30200	MA_10428989g0010	shade67>shade56	Malonyl-ACP expressed in	EMBRYO DEFECTIVE 3	[acyl-carrier-protein] S	143.6025252	0.878910324	0.206317526	4.259988671	2.04E-05	0.001975366
PITA_000044876	High quality	AT2G31750	MA_10428919g0010	shade67>shade56	Encodes an auxin glycosylt	UDP-GLUCOSYL TRANS	auxin metabolic proce	498.7146575	13.03676329	1.768829387	7.37027742	1.70E-13	5.93E-10
PITA_000044972	High quality	AT3G27540	MA_8029g0010	shade67>shade56	beta-1,4-N-acetylglucosaminyltransferase family p		beta-1,4-mannosylglyc	117.7618844	21.90752019	5.978408233	3.664440322	0.00024788	0.012848156
PITA_000045127	Low quality	AT1G80490	MA_83125g0010	shade67>shade56	Encodes a protein with a Li	TOPLESS-RELATED 1 (T	cytoplasm-GO:000573	59.70873988	21.24984851	5.979444214	3.553816667	0.000379684	0.017345582
PITA_000045419	High quality	AT1G29660	MA_1194g0010	shade67>shade56	GDSL-motif esterase/acyltransferase/lipase. Enzy		apoplast-GO:0048046,	368.9772174	6.314586508	1.927630275	3.275828665	0.001053525	0.037769944
PITA_000045438	High quality	AT4G20140	MA_10428862g0010	shade67<shade56	Encodes GASSHO1 (GSO1),	GASSHO1 (GSO1)	Casparian strip-GO:00	42.67076684	-16.83746958	5.161060159	-3.262405215	0.001104711	0.039172784
PITA_000045609	High quality	AT2G23790	MA_2779g0010	shade67>shade56	calcium uniporter (DUF607);(source:Araport11)		calcium channel activit	1181.704801	14.27942096	2.441859043	5.847766272	4.98E-09	3.01E-06
PITA_000045696	High quality	AT1G60710	MA_48532g0010	shade67>shade56	Encodes ATB2.	(ATB2)	cytoplasm-GO:000573	445.7093295	23.39192794	3.238805684	7.222393136	5.11E-13	1.48E-09
PITA_000045734	High quality	AT3G11180	MA_77600g0010	shade67>shade56	One of 4 paralogs encoding	JASMONATE-INDUCED	cytoplasm-GO:000573	23.29923651	20.02661771	6.049868908	3.310256473	0.000932105	0.034390338
PITA_000045814	High quality	AT1G01490	MA_43035g0010	shade67>shade56	Heavy metal transport/detoxification superfamily		chloroplast-GO:00095(	74.72066596	30	6.149645648	4.878329861	1.07E-06	0.000151672
PITA_000045884	High quality	AT3G07940	MA_10427331g0010	shade67<shade56	Calcium-dependent ARF-type GTPase activating pr		biological_process_unl	56.29712229	-21.67765727	5.981554625	-3.624084144	0.000289987	0.014114882
PITA_000045962	Low quality	AT1G20770	MA_10430983g0010	shade67>shade56	coiled-coil protein;(source:Araport11)		extrinsic component o	84.48512306	1.192666607	0.283785899	4.20269862	2.64E-05	0.002439656
PITA_000046197	Low quality	AT3G10870	MA_101711g0010	shade67>shade56	Encodes a methyl IAA este	METHYL ESTERASE 17	hydrolase activity, acti	288.5681702	11.33744382	1.931247001	5.870530189	4.34E-09	2.69E-06
PITA_000046335	Low quality	AT1G14410	MA_709537g0010	shade67>shade56	Encodes a homolog of the	WHIRLY 1 (WHY1)	chloroplast-GO:00095(	134.9965357	30	6.149210135	4.878675365	1.07E-06	0.000151672
PITA_000046361	High quality	AT5G10630	MA_921184g0010	shade67>shade56	Transcripts of this gene are	HSP70 SUBFAMILY B S	cytoplasm-GO:000573	95.26928406	2.79932913	0.649008876	4.313237054	1.61E-05	0.001614529
PITA_000046705	Low quality	AT1G05200	MA_10429132g0020	shade67>shade56	Encodes a putative glutam	GLUTAMATE RECEPTO	calcium channel activit	92.44923834	20.31528283	6.036973464	3.365143636	0.000765038	0.029857167
PITA_000047072	High quality	AT4G10780	MA_212337g0010	shade67>shade56	LRR and NB-ARC domains-containing disease resis		ADP binding-GO:00435	420.1948249	11.16572346	2.325694653	4.801027274	1.58E-06	0.000207219
PITA_000047320	High quality	AT1G72320	MA_10434208g0010	shade67>shade56	Encodes a member of the	PUMILIO 23 (PUM23)	90S preribosome-GO:	173.1851138	21.01929006	5.978132203	3.516029647	0.000438052	0.01929805
PITA_000047476	High quality	AT4G02280	MA_113423g0010	shade67>shade56	Encodes a protein with suc	SUCROSE SYNTHASE 3	chloroplast-GO:00095(	181.1455698	18.49583213	3.696593389	5.003480281	5.63E-07	0.000151672
PITA_000047565	Low quality	AT4G24310	MA_461971g0010	shade67>shade56	transmembrane protein, p	DUF679 DOMAIN MEN	endomembrane syster	135.5201355	1.783234084	0.543383052	3.281725624	0.001031739	0.03714262
PITA_000047748	High quality	AT4G38620	MA_22140g0010	shade67>shade56	Encodes a R2R3 MYB prote	MYB DOMAIN PROTEII	regulation of phenylpr	134.2912929	3.482609124	0.764303153	4.556580867	5.20E-06	0.000600623
PITA_000047798	High quality	AT5G58440	MA_42929g0010	shade67<shade56	sorting nexin 2A;(source:A	SORTING NEXIN 2A (SN	cytosol-GO:0005829,d	198.5189207	-1.099896503	0.323335398	-3.401720039	0.000669632	0.026532125
PITA_000048230	High quality	AT5G20030	MA_35064g0010	shade67>shade56	Plant Tudor-like RNA-binding protein;(source:Arap		biological_process_unl	50.01851528	21.97528072	4.590546232	4.787073175	1.69E-06	0.000221038
PITA_000048358	Low quality	AT4G00970	MA_962355g0010	shade67>shade56	Encodes a cysteine-rich rec	CYSTEINE-RICH RLK (RI	chloroplast-GO:00095(	67.36591526	23.71103229	6.039326389	3.926105456	8.63E-05	0.005999865
PITA_000048531	High quality	AT3G48080	MA_10437224g0010	shade67>shade56	alpha/beta-Hydrolases superfamily protein;(sourc		lipid metabolic process	308.85492	13.09488793	3.979093669	3.29092226	0.000998595	0.036250674
PITA_000048869	High quality	AT5G09430	MA_182752g0010	shade67<shade56	alpha/beta-Hydrolases superfamily protein;(sourc		cytoplasm-GO:000573	275.580156	-1.409931141	0.345135314	-4.085154676	4.40E-05	0.003693167
PITA_000048882	High quality	AT3G54950	MA_10431235g0020	shade67<shade56	Encodes pPLAIIIbeta, a me	PATATIN-RELATED PH(	acyl-CoA hydrolase act	353.8316551	-25.52963592	4.512265784	-5.65783071	1.53E-08	7.81E-06
PITA_000048907	High quality	AT3G05950	MA_10320441g0010	shade67<shade56	RmlC-like cupins superfamily protein;(source:Arap		cell wall-GO:0005618,e	131.1307553	-23.25188509	5.843335781	-3.979214265	6.91E-05	0.005241327
PITA_000048996	High quality	AT4G23160	MA_10604g0010	shade67>shade56	Encodes a cysteine-rich rec	CYSTEINE-RICH RLK (RI	ATP binding-GO:00055	40.20648036	20.21385312	6.038450393	3.347523256	0.000815371	0.031184421
PITA_000049291	High quality	AT2G28490	MA_286762g0010	shade67>shade56	RmlC-like cupins superfamily protein;(source:Arap		biological_process_unl	14.36638734	8.773520447	2.507098319	3.499472032	0.000466181	0.020161637
PITA_000049509	High quality	AT5G62890	MA_76962g0010	shade67>shade56	Xanthine/uracil permease family protein;(source:		cell wall-GO:0005618,e	91.33303694	8.580948494	1.617519342	5.305005185	1.13E-07	4.31E-05
PITA_000049570	Low quality	AT3G14360	MA_19704g0010	shade67>shade56	alpha/beta-Hydrolases sup	OIL BODY LIPASE 1 (AT	extracellular region-G(	83.97121676	2.214857179	0.658855801	3.361672124	0.000774721	0.03018971
PITA_000049803	Low quality	AT1G21350	MA_98916g0010	shade67>shade56	Thioredoxin superfamily protein;(source:Araport1		antioxidant activity-GC	36.93017701	7.237085854	1.846217435	3.919953153	8.86E-05	0.006073911
PITA_000049820	Low quality	AT1G12860	MA_10433418g0010	shade67<shade56	Encodes ICE2 (Inducer of C	SCREAM 2 (SCRM2)	DNA-binding transcript	49.93833591	-22.98044405	5.980087766	-3.842827222	0.000121625	0.007673008
PITA_000050276	Low quality	AT5G15280	MA_100612g0010	shade67>shade56	Pentatricopeptide repeat (PPR) superfamily protei		biological_process_unl	101.4577637	1.694294715	0.491382925	3.448013	0.000564727	0.023225286
PITA_000050309	Low quality	AT5G56050	MA_7502g0010	shade67>shade56	late embryogenesis abund	(NHL43)	biological_process_unl	62.58515087	1.757546779	0.47900928	3.669128869	0.000243378	0.012677136
PITA_000050345	Low quality	AT3G49430	MA_102384g0010	shade67>shade56	Barta et al (2010) have pro	SERINE/ARGININE-RICI	cytosol-GO:0005829,m	42.75604155	30	6.150348081	4.877772706	1.07E-06	0.000151672
PITA_000050399	High quality	AT3G06210	MA_10434888g0020	shade67>shade56	ARM repeat superfamily protein;(source:Araport1		biological_process_unl	85.36326923	23.08305961	4.608734189	5.008546525	5.48E-07	0.000151672
PITA_000050673	High quality	AT3G10600	MA_78005g0010	shade67>shade56	Encodes a member of the	CATIONIC AMINO ACID	amino acid transmemb	251.8067577	22.27494661	6.035862204	3.690433256	0.000223872	0.011926099
PITA_000050761	Low quality	AT5G01710	MA_307938g0010	shade67>shade56	methyltransferase;(source:Araport11)		Golgi trans cisterna-G(	203.4816067	24.57229046	6.035971806	4.070975022	4.68E-05	0.003863052
PITA_000050835	High quality	AT4G25000	MA_30833g0020	shade67<shade56	Predicted to be secreted p	ALPHA-AMYLASE-LIKE	alpha-amylase activity-	28.25648091	-22.09646659	5.993085615	-3.686993314	0.000226919	0.012036912
PITA_000050838	High quality	AT3G18680	MA_245601g0010	shade67<shade56	Encodes a functional UMP	PLASTID 55 UMP KINA!	chloroplast-GO:00095(	162.7476295	-0.721766121	0.217447398	-3.319267679	0.000902539	0.033560494
PITA_000051999	High quality	AT3G27650	MA_10429262g0010	shade67>shade56	LOB domain-containing pr	LOB DOMAIN-CONTAII	biological_process_unl	302.6174296	23.90429525	5.476334052	4.365017733	1.27E-05	0.001316297
PITA_000052335	Low quality	AT5G05440	MA_43111g0010	shade67>shade56	Encodes a member of the	PYRABACTIN RESISTAN	abscisic acid binding-G	26.63713276	7.916752365	2.428635473	3.259753246	0.001115092	0.03937971
PITA_000052663	High quality	AT1G57560	MA_44263g0010	shade67<shade56	Member of the R2R3 facto	MYB DOMAIN PROTEII	DNA-binding transcript	189.7156802	-20.39720576	5.979279448	-3.411315014	0.000646504	0.025774294
PITA_000052749	Low quality	AT5G15800	MA_5917g0010	shade67>shade56	Encodes a MADS box trans	SEPALLATA1 (SEP1)	DNA-binding transcript	850.4427398	12.07179639	3.246996979	3.717834192	0.000200938	0.011153602
PITA_000052942	High quality	AT5G47230	MA_27309g0010	shade67>shade56	encodes a member of the	ETHYLENE RESPONSIVI	DNA binding-GO:0003	19.20119786	5.560169444	1.411502359	3.939185372	8.18E-05	0.005844072
PITA_000052994	Low quality	AT5G18420	MA_9422g0010	shade67<shade56	CCR4-NOT transcription co	(NOT11)	biological_process_unl	81.51938581	-0.95676857	0.28808294	-3.321156648	0.000896452	0.033381923
PITA_000053319	Low quality	AT1G76150	MA_10431519g0010	shade67>shade56	Encodes a monofunctional	ENOYL-COA HYDRATAS	17-beta-hydroxysteroi	40.32950182	30	6.150537319	4.877622627	1.07E-06	0.000151672
PITA_000053401	High quality	AT4G28780	MA_15196g0010	shade67>shade56	GDSL-motif esterase/acyltransferase/lipase. Enzy		chloroplast-GO:00095(	103.3124638	22.05258932	4.972758382	4.434679432	9.22E-06	0.001015017
PITA_000053531	High quality	AT2G39890	MA_10433063g0010	shade67>shade56	Encodes a proline transpor	PROLINE TRANSPORTE	amino acid transmemb	206.1897955	21.34033138	2.48811725	8.576899414	9.75E-18	1.27E-13
PITA_000053541	High quality	AT3G14740	MA_37406g0010	shade67>shade56	RING/FYVE/PHD zinc finger superfamily protein;(s		nucleus-GO:0005634	193.6956076	2.99295672	0.884820052	3.38255978	0.000718136	0.028196077
PITA_000053623	Low quality	AT1G22030	MA_665833g0010	shade67>shade56	BPS1-like protein;(source:Araport11)		biological_process_unl	282.7903685	22.06439952	6.035531468	3.655750887	0.00025643	0.013068894
PITA_000053721	High quality	AT1G11290	MA_109016g0020	shade67>shade56	Pentatricopeptide Repeat	CHLORORESPIRATORY	chloroplast-GO:00095(	45.04183086	1.411157152	0.428696277	3.291741093	0.000995692	0.036246547
PITA_000053758	Low quality	AT3G57450	MA_19912g0010	shade67>shade56	hypothetical protein;(source:Araport11)		biological_process_unl	38.86430591	23.36759448	6.041589014	3.867789488	0.000109826	0.007208183
PITA_000053764	Low quality	AT5G20480	MA_161278g0010	shade67>shade56	Encodes a predicted leucin	EF-TU RECEPTOR (EFR)	detection of bacterium	47.73808791	21.5739321	6.038567079	3.572690643	0.000353332	0.016547401
PITA_000053765	Low quality	AT5G63930	MA_43145g0010	shade67>shade56	Leucine-rich repeat protein kinase family protein;(		ATP binding-GO:00055	225.8768971	22.19865187	4.253572451	5.218825381	1.80E-07	6.33E-05
PITA_000054044	High quality	AT1G22640	MA_116862g0010	shade67<shade56	MYB-type transcription fac	MYB DOMAIN PROTEII	response to wounding	466.3589348	-2.98483765	0.830617218	-3.593517669	0.000326244	0.015559122
PITA_000054189	High quality	AT1G56280	MA_141147g0010	shade67<shade56	Encodes a gene whose tra	DROUGHT-INDUCED 1	DNA-binding transcript	158.7994142	-13.94309847	3.360942236	-4.148568316	3.35E-05	0.003011298
PITA_000054205	Low quality	AT1G68760	MA_11165g0010	shade67>shade56	Encodes a cytosol-localized	NUDIX HYDROLASE 1 (	8-oxo-7,8-dihydrodeox	35.49306036	8.835601569	2.612542713	3.381993154	0.000719619	0.028211685
PITA_000054462	Low quality	AT4G30780	MA_126742g0010	shade67>shade56	ATP-dependent DNA helicase;(source:Araport11)		biological_process_unl	24.76975413	19.21547188	6.041344109	3.180661709	0.001469391	0.048652755
PITA_000054468	High quality	AT5G25800	MA_116229g0010	shade67>shade56	Polynucleotidyl transferase, ribonuclease H-like su		exonuclease activity-G	49.50666891	30	6.150459709	4.877684176	1.07E-06	0.000151672
PITA_000054484	Low quality	AT2G46690	MA_40440g0010	shade67>shade56	SAUR-like auxin-responsive	SMALL AUXIN UPREGU	molecular_function_ur	34.20847582	24.51982783	5.62596859	4.358330026	1.31E-05	0.001351776
PITA_000054543	High quality	AT3G21420	MA_10431216g0020	shade67>shade56	LATERAL BRANCHING OXIC	LATERAL BRANCHING	cytoplasm-GO:000573	352.4314788	19.53578868	4.159310785	4.696881211	2.64E-06	0.000328832
PITA_000054974	High quality	AT5G53890	MA_101553g0010	shade67<shade56	Encodes a leucine-rich rep	PHYTOSYLFOKINE-ALP	chloroplast-GO:00095(	48.15543775	-23.20519031	5.980276414	-3.880287248	0.000104333	0.006900325
PITA_000055028	High quality	AT1G62020	MA_10428405g0010	shade67>shade56	Member of the Coat Prote	α1 COAT PROTEI	COPI vesicle coat-GO:0	419.722854	23.56530138	4.554618145	5.17393569	2.29E-07	7.45E-05
PITA_000055126	Low quality	AT3G60520	MA_135240g0010	shade67>shade56	zinc ion-binding protein;(source:Araport11)		cellular response to hy	26.33436147	20.85780357	5.920718646	3.52284998	0.000426933	0.018968959
PITA_000055201	High quality	AT4G01070	MA_10433602g0010	shade67>shade56	the glycosyltransferase (U(	(GT72B1)	chloroplast-GO:00095(	352.3110579	24.25317876	6.036429057	4.017802335	5.87E-05	0.004609824
PITA_000055939	High quality	AT1G74580	MA_56119g0010	shade67>shade56	Pentatricopeptide repeat (PPR) superfamily protei		biological_process_unl	36.35419293	20.07503448	5.288798003	3.795765025	0.000147189	0.008815053
PITA_000057328	Low quality	AT1G69550	MA_10235009g0010	shade67<shade56	disease resistance protein (TIR-NBS-LRR class);(sou		ADP binding-GO:00435	40.01474923	-30	6.094044853	-4.922838726	8.53E-07	0.000151672
PITA_000057665	High quality	AT2G39540	MA_25298g0010	shade67>shade56	Gibberellin-regulated family protein;(source:Arap		extracellular region-G(	50.71427885	2.676597667	0.767345834	3.488124322	0.000486422	0.020828792
PITA_000058201	High quality	AT2G32440	MA_107159g0010	shade67>shade56	ent-kaurenoic acid hydroxy	ENT-KAURENOIC ACID	brassinosteroid biosyn	284.9214083	10.88979926	3.006626792	3.62193249	0.00029241	0.014179724
PITA_000058630	High quality	AT5G44050	MA_829125g0010	shade67<shade56	MATE efflux family protein	(ATDTX28)	antiporter activity-GO:	88.17385775	-22.90880686	5.680478256	-4.032901074	5.51E-05	0.004424377
PITA_000058678	High quality	AT3G48770	MA_10426213g0030	shade67>shade56	ATP/DNA binding protein;(source:Araport11)		biological_process_unl	36.93777974	30	6.106334477	4.912931009	8.97E-07	0.000151672
PITA_000058728	High quality	AT4G33270	MA_95353g0010	shade67>shade56	Encodes a CDC20 protein t	CELL DIVISION CYCLE 2	anaphase-promoting c	40.65885045	30	6.150547371	4.877614656	1.07E-06	0.000151672
PITA_000058730	High quality	AT4G01050	MA_157164g0010	shade67<shade56	hydroxyproline-rich glycop	THYLAKOID RHODANE!	chloroplast-GO:00095(	912.4560308	-0.822781971	0.253854973	-3.241149705	0.001190486	0.041312582
PITA_000058827	High quality	AT3G22990	MA_1130411g0010	shade67>shade56	Armadillo-repeat containin	LEAF AND FLOWER REI	anther development-G	298.8103778	24.62963571	5.033159897	4.893473725	9.91E-07	0.000151672
PITA_000058992	High quality	AT5G65380	MA_170499g0010	shade67>shade56	MATE efflux family protein;(source:Araport11)		antiporter activity-GO:	184.4496385	20.12835738	5.150679987	3.907902924	9.31E-05	0.006334747
PITA_000059140	Low quality	AT1G06340	MA_172637g0030	shade67>shade56	Plant Tudor-like protein;(source:Araport11)		biological_process_unl	125.3379211	6.207930527	1.70303083	3.645224983	0.000267158	0.013490501
PITA_000059481	High quality	AT4G10500	MA_186973g0010	shade67>shade56	2-oxoglutarate (2OG) and I	DMR6-LIKE OXYGENAS	chloroplast-GO:00095(	40.49303278	23.2032074	5.850846908	3.965786111	7.32E-05	0.00541718
PITA_000059870	High quality	AT4G02780	MA_10433916g0010	shade67<shade56	Catalyzes the conversion o	GA REQUIRING 1 (GA1	chloroplast-GO:00095(	196.8820031	-1.763150356	0.553392773	-3.186074056	0.001442177	0.048177084
PITA_000059882	High quality	AT3G53810	MA_10434498g0010	shade67<shade56	Concanavalin A-like lectin	L-TYPE LECTIN RECEPT	cytosol-GO:0005829,d	202.0913742	-21.94202053	5.978307765	-3.670272826	0.000242292	0.01266399
PITA_000060057	Low quality	AT3G17390	MA_141759g0010	shade67>shade56	S-adenosylmethionine synt	METHIONINE OVER-AC	cell wall-GO:0005618,c	71.70004802	18.88212744	4.08126079	4.62654273	3.72E-06	0.000447424
PITA_000060080	Low quality	AT5G05280	MA_10436028g0020	shade67<shade56	Encodes a RING-finger E3 I	DEFECTIVE IN ANTHER	anther dehiscence-GO	133.9858628	-19.84925779	5.703951358	-3.47991358	0.000501576	0.021267458
PITA_000060555	High quality	AT1G72470	MA_89549g0010	shade67>shade56	A member of EXO70 gene	EXOCYST SUBUNIT EX(	cytosol-GO:0005829,e	50.838539	1.91883439	0.442912777	4.332307606	1.48E-05	0.001492311
PITA_000060615	Low quality	AT3G21420	MA_10426390g0020	shade67<shade56	LATERAL BRANCHING OXIC	LATERAL BRANCHING	cytoplasm-GO:000573	230.2334919	-25.21640033	4.354250352	-5.791215086	6.99E-09	4.13E-06
PITA_000060772	High quality	AT5G58250	MA_46214g0010	shade67<shade56	Involved in tetrapyrrole bic	EMBRYO DEFECTIVE 3	chlorophyll biosynthet	422.8902938	-0.811631169	0.193065613	-4.203913673	2.62E-05	0.002435256
PITA_000060782	High quality	AT1G55580	MA_10255g0010	shade67<shade56	Encodes a member of the	LATERAL SUPPRESSOR	axillary shoot meristen	85.70076248	-21.55791335	5.984603468	-3.602229198	0.0003155	0.015130029
PITA_000060827	Low quality	AT3G47570	MA_10434490g0020	shade67>shade56	Leucine-rich repeat protein kinase family protein;(		kinase activity-GO:001	59.26253257	21.65162176	6.037866428	3.585972301	0.000335825	0.015928382
PITA_000060911	High quality	AT3G10050	MA_10434827g0010	shade67>shade56	first enzyme in the biosynt	L-O-METHYLTHREONIN	chloroplast-GO:00095(	72.35514342	1.657099132	0.305318922	5.427436737	5.72E-08	2.40E-05
PITA_000061475	Low quality	AT4G08280	MA_10253568g0010	shade67>shade56	Thioredoxin superfamily protein;(source:Araport1		chloroplast-GO:00095(	76.86329793	7.600501975	1.597028361	4.759152787	1.94E-06	0.000252652
PITA_000062104	High quality	AT4G30610	MA_10277041g0010	shade67>shade56	Encodes a secreted glycos	BRI1 SUPPRESSOR 1 (B	brassinosteroid media	323.6653859	11.59089563	2.354564676	4.922734016	8.53E-07	0.000151672

PITA_000062192	Low quality	AT1G11530	MA_164160g0010	shade67<shade56	Encodes a monocysteinic t	C-TERMINAL CYSTEINE	cytoplasm-GO:000573	94.37820703	-19.12941033	5.494838772	-3.481341513	0.000498909	0.021188956
PITA_000062645	High quality	AT2G46750	MA_9092998g0010	shade67<shade56	Encodes a homolog of rat L	L-GULONO-1,4-LACTO	D-arabinono-1,4-lactor	121.6365889	-2.392220651	0.623412752	-3.83729823	0.000124395	0.007797232
PITA_000062737	High quality	AT1G14590	MA_10434802g0010	shade67<shade56	Nucleotide-diphospho-sugar transferase family pr		biological_process_unl	79.69382241	1.081426015	0.309025827	3.49946807	0.000466187	0.020161637
PITA_000062784	High quality	AT2G26770	MA_40637g0020	shade67<shade56	Encodes an expansin. Nam	EXPANSIN A10 (EXPA1	extracellular region-GC	334.5392468	1.744075372	0.420681049	4.145837747	3.39E-05	0.003034553
PITA_000062913	Low quality	AT1G19400	MA_401170g0010	shade67<shade56	Tail-anchored (TA) OEP me	ERYTHRONATE-4PHOS	cellular amino acid me	154.7894782	-1.83731583	0.407321227	-4.510729386	6.46E-06	0.000733282
PITA_000063012	High quality	AT5G65360	MA_10432805g0020	shade67<shade56	Histone superfamily protei	HISTONE 3.1 (H3.1)	chromocenter-GO:001	299.8635922	22.22667017	4.294153958	5.176030106	2.27E-07	7.45E-05
PITA_000063135	High quality	AT1G72840	MA_721749g0010	shade67<shade56	Disease resistance protein (TIR-NBS-LRR class);(so		ADP binding-GO:00435	183.3395246	23.43384009	3.959769312	5.917981135	3.26E-09	2.07E-06
PITA_000063162	High quality	AT5G36930	MA_10426388g0010	shade67<shade56	Disease resistance protein (TIR-NBS-LRR class) fam		ADP binding-GO:00435	55.78958	24.41548048	5.831292771	4.186975588	2.83E-05	0.002605617
PITA_000063176	Low quality	AT1G60500	MA_10431097g0010	shade67<shade56	Dynamin related protein 4	DYNAMIN RELATED PR	axon-GO:0030424,cytc	253.6000407	-22.28943719	5.979388584	-3.727711767	0.000193226	0.01080072
PITA_000063289	High quality	AT3G54420	MA_10435326g0010	shade67<shade56	encodes an EP3 chitinase t	HOMOLOG OF CARRO	cell wall-GO:0005618,c	240.3487299	6.878250046	0.988508181	6.958212565	3.45E-12	7.46E-09
PITA_000063375	High quality	AT5G51950	MA_115358g0010	shade67<shade56	Glucose-methanol-choline (GMC) oxidoreductase		chloroplast-GO:00095	33.12655491	-30	6.094111997	-4.922784487	8.53E-07	0.000151672
PITA_000064299	High quality	AT4G22140	MA_14897g0020	shade67<shade56	Encoding a chromatin rem	EARLY BOLTING IN SHO	chromatin binding-GO	68.90664279	2.828301854	0.764319429	3.700418629	0.000215244	0.011583073
PITA_000064381	High quality	AT4G17900	MA_107063g0010	shade67<shade56	PLATZ transcription factor family protein;(source:		nucleus-GO:0005634,p	213.2058119	21.87829393	6.036120167	3.624562356	0.000289451	0.014114882
PITA_000064516	High quality	AT1G58340	MA_170275g0010	shade67<shade56	Encodes a plant MATE (mu	(ZF14)	antiporter activity-GO:	130.5665064	11.60859728	2.541333894	4.567915027	4.93E-06	0.000574155
PITA_000064698	Low quality	AT3G15110	MA_837813g0010	shade67<shade56	transmembrane protein;(source:Araport11)		biological_process_unl	236.3549326	-0.807657323	0.173270285	-4.661256981	3.14E-06	0.000381723
PITA_000064754	High quality	AT1G11050	MA_54558g0020	shade67<shade56	Protein kinase superfamily protein;(source:Arapor		chloroplast-GO:00095	41.0044262	20.90757589	6.039715956	3.461681981	0.000536811	0.022468266
PITA_000064990	High quality	AT1G12280	MA_133928g0010	shade67<shade56	Encodes a NB-LRR protein	SUPPRESSOR OF MKK1	ADP binding-GO:00435	99.08784241	21.98394461	4.928998424	4.460124091	8.19E-06	0.000909856
PITA_000065289	High quality	AT5G46330	MA_1148983g0010	shade67<shade56	Encodes a leucine-rich rep	FLAGELLIN-SENSITIVE 2	defense response by ca	213.7915341	21.25546345	6.035814149	3.521556981	0.00042902	0.01902918
PITA_000065705	High quality	AT3G20820	MA_175577g0010	shade67<shade56	Leucine-rich repeat (LRR) family protein;(source:A		apoplast-GO:0048046,	66.26655679	-25.2252732	5.982861513	-4.216255574	2.48E-05	0.002314054
PITA_000065777	Low quality	AT3G63400	MA_68436g0020	shade67<shade56	Cyclophilin-like peptidyl-prolyl cis-trans isomerase		cyclosporin A binding-C	96.97717728	30	6.149608524	4.878359311	1.07E-06	0.000151672
PITA_000065848	Low quality	AT5G52960	MA_10017907g0010	shade67<shade56	tRNA dimethylallyltransferase;(source:Araport11)		biological_process_unl	342.919244	-1.055348963	0.18725277	-5.635959145	1.74E-08	8.54E-06
PITA_000065896	Low quality	AT3G22060	MA_18855g0020	shade67<shade56	contains Pfam profile: PF01657 Domain of unknow		cell wall-GO:0005618,c	177.0218062	2.162013779	0.603814016	3.580595552	0.000342812	0.016141971
PITA_000066050	Low quality	AT5G66880	MA_3140839g0010	shade67<shade56	encodes a member of SNF	SUCROSE NONFERMEI	abscisic acid-activated	104.2232736	22.44042307	5.97862161	3.753444278	0.000174421	0.01004645
PITA_000066482	Low quality	AT1G19210	MA_314205g0010	shade67<shade56	encodes a member of the I	(ERF017)	DNA-binding transcript	107.494211	19.76778924	6.036772794	3.274562405	0.001058258	0.037887372
PITA_000066671	High quality	AT3G47550	MA_10435273g0010	shade67<shade56	RING/FYVE/PHD zinc finger superfamily protein;(so		cytoplasm-GO:000573	58.75759569	19.69692869	6.038439459	3.261923685	0.001106589	0.039185925
PITA_000066728	High quality	AT2G05910	MA_571782g0010	shade67<shade56	LURP-one-like protein (DUF567);(source:Araport1		cellular_component_u	148.999131	20.05526596	6.149471093	3.261299331	0.001109029	0.039218886
PITA_000066828	High quality	AT2G46410	MA_9991g0010	shade67<shade56	Nuclear-localized R3-type I	CAPRICE (CPC)	cell differentiation-GO	49.85944889	20.87340917	6.038937511	3.456470469	0.000547299	0.022724281
PITA_000067002	High quality	AT2G36780	MA_10426944g0020	shade67<shade56	UDP-Glycosyltransferase superfamily protein;(sou		chloroplast-GO:00095	341.6231949	9.439338628	1.57819005	5.981116549	2.22E-09	1.60E-06
PITA_000067156	High quality	AT4G19970	MA_10429744g0010	shade67<shade56	nucleotide-diphospho-sugar transferase family pro		cytoplasm-GO:000573	94.40098397	23.10634509	6.036635765	3.827685816	0.000129354	0.008024253
PITA_000067547	Low quality	AT4G17500	MA_166248g0010	shade67<shade56	Encodes a member of the I	ETHYLENE RESPONSIV	cell division-GO:00513	196.8832184	30	6.14907733	4.878780732	1.07E-06	0.000151672
PITA_000067860	Low quality	AT1G77280	MA_121123g0010	shade67<shade56	kinase with adenine nucleotide alpha hydrolases-li		cytoplasm-GO:000573	209.5457671	-21.63187219	4.283393521	-5.050171572	4.41E-07	0.000131876
PITA_000067916	High quality	AT5G51110	MA_167002g0010	shade67<shade56	Encodes a protein involved	SDIR1-INTERACTING PI	4-alpha-hydroxytetrah	669.6218357	-0.685373558	0.204788099	-3.346745049	0.000817664	0.0312081
PITA_000068128	High quality	AT5G57540	MA_8261658g0010	shade67<shade56	Encodes a xyloglucan endo	XYLOGLUCAN ENDOTR	cell wall biogenesis-GC	719.9065165	12.02558573	3.063842957	3.925000692	8.67E-05	0.006011394
PITA_000068237	High quality	AT1G71870	MA_334549g0010	shade67<shade56	MATE efflux family protein	(BIGE1A)	antiporter activity-GO:	85.51874302	1.595030145	0.359736694	4.433882254	9.26E-06	0.001015017
PITA_000068253	High quality	AT3G38280	MA_10435621g0020	shade67<shade56	putative receptor serine/th	PR5-LIKE RECEPTOR KI	extracellular region-GC	86.74738946	21.62934924	6.036616487	3.583025241	0.000339638	0.016029061
PITA_000068863	Low quality	AT5G01380	MA_7129732g0010	shade67<shade56	Homeodomain-like superfamily protein;(source:A		DNA-binding transcript	29.79895172	-22.03163664	5.982715164	-3.682548147	0.000230914	0.0121658
PITA_000069525	High quality	AT2G20030	MA_12352g0010	shade67<shade56	RING/U-box superfamily p	ARABIDOPSIS T??XICO	nucleus-GO:0005634,p	374.6209012	20.88179236	6.037087682	3.458918183	0.00054235	0.022627209
PITA_000070498	High quality	AT5G17330	MA_442130g0010	shade67<shade56	Encodes one of two isofor	GLUTAMATE DECARBC	calmodulin binding-GC	342.3572151	0.816108655	0.176462844	4.624818665	3.75E-06	0.000447448
PITA_000070556	High quality	AT5G40380	MA_10431174g0010	shade67<shade56	Encodes a cysteine-rich rec	CYSTEINE-RICH RLK (RI	chloroplast-GO:00095	11.78881129	-30	6.098407141	-4.919317341	8.68E-07	0.000151672
PITA_000070648	High quality	AT5G28040	MA_172474g0010	shade67<shade56	Member of the GeBP/GPL	VIRF INTERACTING PR	cytoplasm-GO:000573	88.54874287	0.837049834	0.240418305	3.481639355	0.000498354	0.021188956
PITA_000070651	Low quality	AT5G41120	MA_86228g0010	shade67<shade56	Esterase/lipase/thioestera	(ELT4)	chloroplast-GO:00095	47.88116041	30	6.150000326	4.878048522	1.07E-06	0.000151672
PITA_000070862	Low quality	AT1G51900	MA_19414g0010	shade67<shade56	Translation initiation factor	SUI1 family protein;(sc	cytoplasm-GO:000573	81.81815856	10.34061384	1.616618928	6.396444865	1.59E-10	2.07E-07
PITA_000071108	High quality	AT1G22640	MA_180449g0010	shade67<shade56	MYB-type transcription fac	MYB DOMAIN PROTEII	response to wounding	46.99138113	7.649044667	1.85812276	4.116544305	3.85E-05	0.003332135
PITA_000071129	High quality	AT3G49780	MA_168710g0010	shade67<shade56	Phytosulfokine 3 precursor	PHYTOSULFOKINE 4 PI	cell differentiation-GO	173.6421938	-23.10797779	5.753657476	-4.016224095	5.91E-05	0.004615963
PITA_000071154	High quality	AT4G26890	MA_15568g0010	shade67<shade56	Member of MEKK subfami	MITOGEN-ACTIVATED	nucleus-GO:0005634,p	13.31864547	30	6.153762857	4.875065988	1.09E-06	0.000151672
PITA_000071451	High quality	AT1G72310	MA_904294g0010	shade67<shade56	Encodes a putative RING-H	(ATL3)	nucleus-GO:0005634,p	40.70580589	3.633701993	0.857074863	4.23965531	2.24E-05	0.002115874
PITA_000071550	Low quality	AT2G19130	MA_10235107g0010	shade67<shade56	S-locus lectin protein kinase family protein;(source		calmodulin binding-GC	141.8875717	-21.64671931	4.355562734	-4.969901856	6.70E-07	0.000151672
PITA_000071727	High quality	AT1G69550	MA_10436643g0020	shade67<shade56	disease resistance protein (TIR-NBS-LRR class);(sou		ADP binding-GO:00435	141.0578476	23.75772542	6.036158322	3.935901637	8.29E-05	0.005886188
PITA_000071926	High quality	AT5G44510	MA_10221218g0010	shade67<shade56	Encodes TAO1 (Target of A	TARGET OF AVRB OPEI	ADP binding-GO:00435	38.04085682	19.91360524	3.717902758	5.356139345	8.50E-08	3.45E-05
PITA_000071951	High quality	AT4G18910	MA_62314g0010	shade67<shade56	Encodes an aquaporin hon	NOD26-LIKE INTRINSIC	arsenite transmembra	43.24329714	8.317707187	2.604805019	3.193216815	0.001406973	0.047253465
PITA_000072117	High quality	AT1G71695	MA_10275776g0010	shade67<shade56	Peroxidase superfamily protein;(source:Araport11		cell wall-GO:0005618,e	121.0934355	22.5353646	5.935377526	3.796787062	0.000146584	0.008799078
PITA_000072253	High quality	AT1G24030	MA_9033g0010	shade67<shade56	Protein kinase superfamily	PBS1-LIKE 28 (PBL28)	ATP binding-GO:00055	143.2844014	1.361046102	0.218801064	6.220472959	4.96E-10	4.96E-07
PITA_000072491	Low quality	AT3G51895	MA_10245227g0010	shade67<shade56	Encodes a chloroplast-loca	SULFATE TRANSPORTE	anion:anion antiporter	78.11877656	23.08819604	6.037096755	3.82438728	0.000131098	0.008036537
PITA_000072605	High quality	AT3G21480	MA_10427227g0050	shade67<shade56	BRCT domain-containing DNA repair protein;(sour		biological_process_unl	178.5749015	13.09962896	4.102372035	3.193184054	0.001407132	0.047253465
PITA_000072740	High quality	AT1G70740	MA_9861293g0010	shade67<shade56	Protein kinase superfamily protein;(source:Arapor		ATP binding-GO:00055	172.8690065	21.92930083	5.919716325	3.704451299	0.000211849	0.011549807
PITA_000073098	High quality	AT1G61560	MA_40975g0010	shade67<shade56	A member of a large family	MILDEW RESISTANCE I	mitochondrion-GO:00	196.5654061	-9.453687147	2.892282963	-3.268589992	0.001080848	0.038536901
PITA_000073314	Low quality	AT4G37420	MA_10973g0010	shade67<shade56	glycosyltransferase family protein (DUF23);(source		biological_process_unl	170.7214279	9.441345459	1.680222688	5.619103662	1.92E-08	9.24E-06
PITA_000073597	Low quality	AT1G79930	MA_10430733g0010	shade67<shade56	encodes high molecular we	HEAT SHOCK PROTEIN	ATP binding-GO:00055	184.5637206	22.84332847	5.97789431	3.821300157	0.00013275	0.008118675
PITA_000074055	High quality	AT1G75220	MA_10239544g0010	shade67<shade56	Encodes a vacuolar glucose	ERD6-LIKE 6 (ERDL6)	carbohydrate transme	238.2865053	-1.220791943	0.341797256	-3.571684443	0.000354693	0.016574984
PITA_000074356	High quality	AT3G51680	MA_36042g0010	shade67<shade56	NAD(P)-binding Rossmann	SHORT-CHAIN DEHYDR	cytoplasm-GO:000573	186.6561897	23.49471228	6.03725063	3.891624469	9.96E-05	0.006654101
PITA_000074795	High quality	AT2G18650	MA_229590g0010	shade67<shade56	RING/U-box superfamily pr	MATERNAL EFFECT EM	embryo development	133.3501546	1.857976445	0.543902808	3.416008188	0.000635463	0.025449867
PITA_000074957	Low quality	AT4G34760	MA_8495g0010	shade67<shade56	SAUR-like auxin-responsive	SMALL AUXIN UPREGU	mitochondrion-GO:00	217.4075834	25.01499611	6.035833073	4.144414831	3.41E-05	0.003042962
PITA_000075157	High quality	AT5G18980	MA_10428851g0010	shade67<shade56	ARM repeat superfamily protein;(source:Araport1		biological_process_unl	441.5048839	-24.47685937	5.924307807	-4.131598182	3.60E-05	0.003184902
PITA_000075274	High quality	AT1G69870	MA_10435693g0020	shade67<shade56	Encodes a low affinity nitr	NRT1/ PTR FAMILY 2.1	chloroplast-GO:00095	813.9288599	19.63261393	5.095784275	3.852716848	0.000116814	0.007496892
PITA_000075356	High quality	AT5G45390	MA_140852g0010	shade67<shade56	One of several nuclear-enc	CLP PROTEASE P4 (CLP	ATPase binding-GO:00	353.801512	-1.148617191	0.359614036	-3.194027694	0.001403027	0.04724109
PITA_000075382	Low quality	AT1G36050	MA_56684g0010	shade67<shade56	Endoplasmic reticulum vesicle transporter protein		COPII-coated ER to Gol	36.66783795	11.52561438	2.929820877	3.933897281	8.36E-05	0.005897122
PITA_000075811	Low quality	AT5G25500	MA_209704g0010	shade67<shade56	exosome complex exonuclease;(source:Araport11		biological_process_unl	122.4586477	22.96236167	5.794051776	3.963092246	7.40E-05	0.005463133
PITA_000076136	Low quality	AT5G22040	MA_117242g0010	shade67<shade56	ubiquitin carboxyl-terminal hydrolase;(source:Ara		biological_process_unl	286.7044431	21.1665641	5.007090035	4.227318453	2.36E-05	0.002219105
PITA_000076245	High quality	AT1G26560	MA_488148g0010	shade67<shade56	beta glucosidase 40;(sourc	BETA GLUCOSIDASE 40	apoplast-GO:0048046,	221.9542221	-9.768771682	2.456098706	-3.977353052	6.97E-05	0.005265394
PITA_000076438	High quality	AT1G05675	MA_89176g0010	shade67<shade56	UDP-Glycosyltransferase superfamily protein;(sou		quercetin 3-O-glucosyl	6.319093008	30	6.158511009	4.871307359	1.11E-06	0.000151672
PITA_000076457	High quality	AT5G16080	MA_893141g0010	shade67<shade56	carboxyesterase 17;(source	CARBOXYESTERASE 17	cellular_component_u	145.8374629	1.501619129	0.463168817	3.242055759	0.001186708	0.04129172
PITA_000076582	High quality	AT2G16430	MA_10429691g0010	shade67<shade56	Encodes an acid phosphata	PURPLE ACID PHOSPHA	acid phosphatase activ	152.1963584	19.57818204	6.040013133	3.24141382	0.001189384	0.041312582
PITA_000076951	High quality	AT4G23180	MA_10237752g0010	shade67<shade56	Encodes a receptor-like pro	CYSTEINE-RICH RLK (RI	cytosol-GO:0005829,de	427.3905424	25.18988971	3.948169899	6.380143295	1.77E-10	2.19E-07
PITA_000076999	High quality	AT5G48740	MA_9257454g0010	shade67<shade56	Leucine-rich repeat protein kinase family protein;(		chloroplast-GO:00095	308.5703522	24.56327378	5.11454921	4.802627323	1.57E-06	0.000206613
PITA_000077290	High quality	AT4G24340	MA_10428395g0010	shade67<shade56	Phosphorylase superfamily protein;(source:Arapo		catalytic activity-GO:00	252.2530471	1.10327202	0.3344399	3.298864818	0.000970766	0.035488273
PITA_000077719	High quality	AT3G25810	MA_10121006g0010	shade67<shade56	Terpenoid cyclases/Protein	prenyltransferases sup	(4S)-limonene synthas	169.8708236	2.357878436	0.731458713	3.223529086	0.001266214	0.043476148
PITA_000077753	Low quality	AT5G59250	MA_698449g0010	shade67<shade56	Encodes a chloroplast local	(HP59)	carbohydrate transme	86.3363322	-0.958746864	0.281223716	-3.409196338	0.000651546	0.025934113
PITA_000077857	High quality	AT1G74950	MA_68496g0010	shade67<shade56	TIFY domain/Divergent CC	(TIFY10B)	identical protein bindir	62.02920124	13.32289712	2.809783579	4.741609719	2.12E-06	0.000272641
PITA_000077880	Low quality	AT4G16265	MA_10427302g0010	shade67<shade56	One of two highly similar, r	(NRPB9B)	DNA-directed 5'-3' RN	40.59698073	20.62993114	3.764561718	5.480035309	4.25E-08	1.87E-05
PITA_000078207	Low quality	AT2G30350	MA_10429286g0010	shade67<shade56	Excinuclease ABC, C subun	HYL1 INTERACTING GI	5'-flap endonuclease a	231.44407	0.928931771	0.28933159	3.210613026	0.001324522	0.045061476
PITA_000078328	High quality	AT1G52240	MA_10199247g0010	shade67<shade56	Encodes a member of KPP-	ROP (RHO OF PLANTS)	cytoplasm-GO:000573	516.2851418	-0.975351733	0.238080855	-4.096724753	4.19E-05	0.003559347
PITA_000078355	High quality	AT3G10340	MA_10429279g0010	shade67<shade56	Encodes PAL4, a putative a	PHENYLALANINE AMM	ammonia-lyase activity	133.3256481	9.401338628	1.992547747	4.718250111	2.38E-06	0.000301611
PITA_000078455	High quality	AT5G16770	MA_62488g0010	shade67<shade56	Member of the R2R3 facto	MYB DOMAIN PROTEII	DNA-binding transcript	59.65268461	-2.121378581	0.547844122	-3.87223025	0.000107844	0.00711442
PITA_000078497	Low quality	AT2G36290	MA_18669g0010	shade67<shade56	alpha/beta-Hydrolases superfamily protein;(source		endoplasmic reticulum	129.2950403	-10.87204792	2.988613762	-3.637823013	0.000274952	0.013717004
PITA_000078623	High quality	AT1G22640	MA_8147g0020	shade67<shade56	MYB-type transcription fac	MYB DOMAIN PROTEII	response to wounding	899.9364647	1.213595682	0.259098472	4.683916788	2.81E-06	0.000345061
PITA_000079003	Low quality	AT4G23140	MA_10253298g0010	shade67<shade56	Arabidopsis thaliana recep	CYSTEINE-RICH RLK (RI	chloroplast-GO:00095	107.0632182	-23.92102655	5.979507455	-4.000501167	6.32E-05	0.004875118
PITA_000079033	High quality	AT3G11980	MA_55232g0010	shade67<shade56	Similar to fatty acid reduct	MALE STERILITY 2 (MS	alcohol-forming fatty a	179.5851604	1.310737158	0.336380331	3.896592749	9.76E-05	0.006569071
PITA_000079043	High quality	AT3G23730	MA_44609g0010	shade67<shade56	xyloglucan endotransgluco	XYLOGLUCAN ENDOTR	cell wall biogenesis-GC	491.537454	20.07887232	4.051392248	4.956042537	7.19E-07	0.000151672
PITA_000079145	Low quality	AT4G14200	MA_47113g0010	shade67<shade56	Pentatricopeptide repeat (PPR) superfamily protei		biological_process_unl	88.00722888	25.14655935	6.039477034	4.16369815	3.13E-05	0.002835881
PITA_000079217	High quality	AT1G80830	MA_100211g0010	shade67<shade56	Thought to be involved in i	NATURAL RESISTANCE	cadmium ion transmer	130.9694642	10.15177339	2.209210608	4.595203983	4.32E-06	0.000513106
PITA_000079363	Low quality	AT2G01300	MA_120879g0010	shade67<shade56	mediator of RNA polymerase II transcription subu		biological_process_unl	53.42614283	17.83912225	4.328954308	4.120884857	3.77E-05	0.003314156
PITA_000079538	High quality	AT3G50170	MA_86436g0010	shade67<shade56	transmembrane protein, putative (DUF247);(sourc		biological_process_unl	396.1736013	-24.3053443	4.681730869	-5.191529583	2.09E-07	7.04E-05
PITA_000079659	Low quality	AT5G04410	MA_10430340g0010	shade67<shade56	NAC family member, funct	NAC DOMAIN CONTAI	cytoplasm-GO:000573	48.10252328	19.29121183	6.039815747	3.194006678	0.001403129	0.04724109
PITA_000079887	High quality	AT4G14440	MA_95101g0010	shade67<shade56	encodes a cytosolic delta3,	3-HYDROXYACYL-COA	carnitine racemase act	139.1903613	20.26380364	6.036985271	3.356609753	0.000789044	0.030564577
PITA_000080116	High quality	AT5G59970	MA_10427181g0010	shade67<shade56	Histone superfamily protein;(source:Araport11)		nucleus-GO:0005634,p	87.69218597	1.801352031	0.445866683	4.040113557	5.34E-05	0.004313407
PITA_000080147	Low quality	AT2G16920	MA_85108g0010	shade67<shade56	ubiquitin-conjugating enzy	UBIQUITIN-CONJUGAT	cytosol-GO:0005829,n	198.8069696	-25.16580016	5.729574165	-4.392263619	1.12E-05	0.001185241
PITA_000080705	Low quality	AT5G72710	MA_6447g0010	shade67<shade56	Encodes a member of the	CASEIN KINASE 1-LIKE	cytoplasm-GO:000573	160.5046351	-23.46348198	4.319042363	-5.432565834	5.55E-08	2.37E-05
PITA_000080841	High quality	AT1G32860	MA_82642g0010	shade67<shade56	Glycosyl hydrolase superfamily protein;(source:Ar		anchored component	32.47265214	3.224734299	1.006954331	3.202463311	0.001362577	0.046174824
PITA_000080981	Low quality	AT3G07320	MA_10426132g0010	shade67<shade56	O-Glycosyl hydrolases family 17 protein;(source:A		anchored component	104.3155574	-11.57490842	3.577453431	-3.23551617	0.00121423	0.041968442
PITA_000080997	High quality	AT3G50950	MA_10425825g0010	shade67<shade56	Encodes a canonical CC-typ	HOPZ-ACTIVATED RESI	ADP binding-GO:00435	75.00203009	10.54168562	2.104872005	5.008231187	5.49E-07	0.000151672
PITA_000081185	Low quality	AT3G30390	MA_10432714g0010	shade67<shade56	Encodes a putative amino acid transporter.		amino acid transmemb	23.96041834	15.67166199	3.896177791	4.022316954	5.76E-05	0.004556454
PITA_000081421	High quality	AT1G55020	MA_10436100g0010	shade67<shade56	lipoxygenase, a defense ge	LIPOXYGENASE 1 (LOX	cytoplasm-GO:000573	63.97669402	-1.199674481	0.369747532	-3.244577388	0.001176251	0.040982712
PITA_000081622	High quality	AT1G26760	MA_135344g0010	shade67<shade56	SET domain protein 35;(so	SET DOMAIN PROTEIN	biological_process_unl	108.8073035	11.10481594	2.488017362	4.463319313	8.07E-06	0.000904114
PITA_000081811	High quality	AT4G39780	MA_66969g0010	shade67<shade56	encodes a member of the DREB subfamily A-6 of E		DNA-binding transcript	163.4772873	30	6.149216601	4.878670235	1.07E-06	0.000151672
PITA_000082006	Low quality	AT2G44530	MA_10436726g0010	shade67<shade56	Phosphoribosyltransferase	PHOSPHORIBOSYL DIP	5-phosphoribose 1-dip	118.3692638	20.74718434	6.036588323	3.436905622	0.000588401	0.023971329
PITA_000082198	High quality	AT4G02420	MA_168754g0010	shade67<shade56	Concanavalin A-like lectin p	L-TYPE LECTIN RECEPT	defense response to ba	288.8644361	-23.49253732	4.225790647	-5.559323516	2.71E-08	1.26E-05
PITA_000082449	High quality	AT5G47840	MA_10427036g0010	shade67<shade56	adenosine monophosphate	ADENOSINE MONOPH	adenylate kinase activi	11534.90979	-1.331063566	0.365468683	-3.642072843	0.000270452	0.013544462
PITA_000082650	Low quality	AT1G21080	MA_129673g0010	shade67<shade56	DNAJ heat shock N-terminal domain-containing pr		cytoplasm-GO:000573	90.88568263	1.399202464	0.342656552	4.083396206	4.44E-05	0.003709272
PITA_000083010	High quality	AT3G14260	MA_77009g0010	shade67<shade56	LURP-one-like protein (DUF567);(source:Araport1		biological_process_unl	54.56532937	7.546393726	1.773425	4.255265222	2.09E-05	0.002010061
PITA_000083227	High quality	AT3G51420	MA_488133g0010	shade67<shade56	Although this enzyme is pr	STRICTOSIDINE SYNTH	biosynthetic process-G	104.8791875	4.370877989	0.869141439	5.028960527	4.93E-07	0.000144021
PITA_000083389	High quality	AT5G48380	MA_136151g0010	shade67<shade56	Encodes a BAK1-interactin	BAK1-INTERACTING RE	defense response to ba	71.21487412	-2.210674656	0.694054997	-3.185157755	0.001446751	0.048177084
PITA_000084088	High quality	AT5G13840	MA_10290598g0010	shade67<shade56	FIZZY-related 3;(source:Ar	FIZZY-RELATED 3 (FZR	anaphase-promoting c	232.6121607	-22.38872038	4.487360506	-4.989284981	6.06E-07	0.000151672
PITA_000084230	High quality	AT5G05340	MA_10070756g0010	shade67<shade56	Encodes a protein with sec	PEROXIDASE 52 (PRX5	apoplast-GO:0048046,	66.42210485	21.41180754	6.037083228	3.54671399	0.000390068	0.01760181
PITA_000084272	High quality	AT5G55090	MA_72906g0010	shade67<shade56	member of MEKK subfamil	MITOGEN-ACTIVATED	nucleus-GO:0005634,p	20.82140382	5.777318589	1.225520741	4.714174469	2.43E-06	0.000306217
PITA_000084437	High quality	AT2G36970	MA_10434576g0010	shade67<shade56	UDP-Glycosyltransferase superfamily protein;(sou		transferase activity, tra	1031.886414	-23.30535137	3.070249807	-7.590702005	3.18E-14	1.38E-10
PITA_000084575	High quality	AT1G49760	MA_111003g0010	shade67<shade56	polyadenylate-binding prot	POLY(A) BINDING PRO	cytoplasm-GO:000573	66.91220095	2.289437136	0.578015969	3.96085447	7.47E-05	0.005467988
PITA_000084730	High quality	AT5G09500	MA_14543g0010	shade67<shade56	Ribosomal protein S19 family protein;(source:Ara		cytoplasm-GO:000573	206.9061839	1.861467901	0.497109021	3.744586848	0.000180691	0.010299379
PITA_000085160	High quality	AT5G10370	MA_10436572g0010	shade67<shade56	helicase domain-containing protein / IBR domain-c		intracellular anatomic	87.35117844	-22.52113673	5.978783217	-3.766842836	0.000165325	0.009634815
PITA_000085348	High quality	AT2G40340	MA_19420g0010	shade67<shade56	Encodes a member of the I	(DREB2C)	DNA-binding transcript	15.57373148	-2.546711129	0.71592325	-3.557240429	0.000374771	0.017302044
PITA_000085567	Low quality	AT2G45550	MA_621586g0010	shade67<shade56	member of CYP76C	CYTOCHROME P450, F.	chloroplast-GO:00095	493.427051	22.36114044	5.21057011	4.291495935	1.77E-05	0.001727674
PITA_000085776	High quality	AT2G31955	MA_10433768g0010	shade67<shade56	COFACTOR OF NITRATE RE	COFACTOR OF NITRAT	4 iron, 4 sulfur cluster	68.51751958	22.58791733	6.03871469	3.740517394	0.000183642	0.010399169
PITA_000085874	High quality	AT5G05320	MA_13849g0010	shade67<shade56	FAD/NAD(P)-binding oxidoreductase family protei		chloroplast-GO:00095	113.2429707	-0.913609884	0.283531765	-3.222251056	0.001271876	0.043612943
PITA_000086459	Low quality	AT5G19400	MA_10435713g0020	shade67<shade56	Encodes SMG7, a protein t	(SMG7)	meiotic cell cycle-GO:0	12.3193807	30	6.153667752	4.875141332	1.09E-06	0.000151672
PITA_000086876	Low quality	AT1G27180	MA_100966g0020	shade67<shade56	disease resistance protein (TIR-NBS-LRR class);(sou		ADP binding-GO:00435	47.47492741	-18.26982796	4.924288597	-3.710145658	0.00020714	0.011406745
PITA_000086887	Low quality	AT3G57120	MA_10158g0010	shade67<shade56	Protein kinase superfamily protein;(source:Arapor		ATP binding-GO:00055	181.3310986	25.0408598	6.036282673	4.148390848	3.35E-05	0.003011298
PITA_000087155	High quality	AT3G62550	MA_114091g0010	shade67<shade56	Adenine nucleotide alpha hydrolases-like superfar		nucleus-GO:0005634,v	53.25152876	30	6.150277124	4.877828982	1.07E-06	0.000151672
PITA_000087209	High quality	AT3G50170	MA_180326g0010	shade67<shade56	transmembrane protein, putative (DUF247);(sourc		biological_process_unl	79.38859221	30	6.1502825	4.877824718	1.07E-06	0.000151672
PITA_000087350	High quality	AT4G39330	MA_10427985g0010	shade67<shade56	cinnamyl alcohol dehydrog	CINNAMYL ALCOHOL D	apoplast-GO:0048046,	82.93106734	30	6.149548511	4.878406918	1.07E-06	0.000151672
PITA_000087469	Low quality	AT5G20610	MA_10431323g0010	shade67<shade56	Encodes a member of a pla	PLASTID MOVEMENT I	actin filament-based m	25.92393654	19.36395085	6.040503976	3.205684646	0.001347416	0.045780429

PITA_000087546	High quality	AT1G02850	MA_139193g0010	shade67>shade56	beta glucosidase 11;(sourc	BETA GLUCOSIDASE 11	beta-glucosidase activi	33.92870541	30	6.151577054	4.876798216	1.08E-06	0.000151672
PITA_000087912	High quality	AT2G01820	MA_10431591g0010	shade67>shade56	Leucine-rich repeat protein kinase family protein;(		plant-type cell wall-GO	65.54859875	20.45467124	6.037229882	3.388088848	0.000703815	0.027717499
PITA_000087936	High quality	AT1G27170	MA_89418g0010	shade67>shade56	transmembrane receptors / ATP binding protein;(		ADP binding-GO:0043	118.6222186	24.20316421	5.978845725	4.048133256	5.16E-05	0.004219838
PITA_000087950	High quality	AT5G07990	MA_10434709g0010	shade67>shade56	Required for flavonoid 3' h	TRANSPARENT TESTA	extracellular region-GO	143.7720845	23.56763476	6.036123823	3.904431959	9.44E-05	0.006394824
PITA_000087955	Low quality	AT1G14980	MA_10429427g0010	shade67>shade56	Encodes mitochondrial-loc	CHAPERONIN 10 (CPN1	ATP binding-GO:00055	230.6538	21.8851465	6.035551469	3.626039247	0.000287802	0.014087646
PITA_000088105	High quality	AT3G02645	MA_10429343g0010	shade67<shade56	transmembrane protein, putative (DUF247);(sourc		biological_process_unl	156.292012	-25.6817873	5.193882446	-4.944622365	7.63E-07	0.000151672
PITA_000088265	High quality	AT4G36470	MA_10433097g0010	shade67>shade56	S-adenosyl-L-methionine-dependent methyltransf		methylation-GO:00322	57.18290104	8.644322327	2.606846921	3.31600688	0.000913136	0.033817351
PITA_000088795	High quality	AT5G02500	MA_10427429g0010	shade67<shade56	encodes a member of heat	HEAT SHOCK COGNATI	apoplast-GO:0048046,	91.87486045	-25.58523954	5.497302568	-4.654144323	3.25E-06	0.0003933
PITA_000088967	High quality	AT1G10630	MA_24477g0010	shade67>shade56	A member of ARF GTPase f	ADP-RIBOSYLATION FA	copper ion binding-GO	303.0226001	21.45100595	6.03608795	3.553792808	0.000379718	0.017345582
PITA_000089146	High quality	AT2G21090	MA_251664g0010	shade67>shade56	Pentatricopeptide repeat (PPR-like) superfamily pr		mitochondrion-GO:000	101.6895522	22.29160712	6.036503094	3.692801408	0.000221797	0.011862044
PITA_000089315	High quality	AT5G57840	MA_9959852g0010	shade67<shade56	encodes a protein whose sequence is similar to an		cytoplasm-GO:000573	64.53305411	-22.19930858	5.981079266	-3.711589096	0.000205962	0.011390145
PITA_000090473	High quality	AT2G47440	MA_39821g0010	shade67>shade56	Tetratricopeptide repeat (TPR)-like superfamily pr		nucleus-GO:0005634	81.31770089	30	6.14966223	4.878316707	1.07E-06	0.000151672
PITA_000090487	Low quality	AT3G03150	MA_10436059g0010	shade67>shade56	hypothetical protein;(source:Araport11)		biological_process_unl	130.4548767	0.75063732	0.224247374	3.347362809	0.000815844	0.031184421
PITA_000090670	Low quality	AT5G06600	MA_10428769g0010	shade67<shade56	Encodes a ubiquitin-specifi	UBIQUITIN-SPECIFIC PI	cysteine-type endopep	174.4867619	-10.35474639	2.963562801	-3.494019559	0.000475806	0.020441576
PITA_000090867	Low quality	AT2G18670	MA_33549g0010	shade67>shade56	RING/U-box superfamily pı	ARABIDOPSIS T??XICO	cellular response to hy	118.0164144	22.13055902	5.978337798	3.701791328	0.000214083	0.011549807
PITA_000090886	High quality	AT2G22590	MA_10430052g0020	shade67<shade56	UDP-Glycosyltransferase superfamily protein;(sou		intracellular membran	683.0552586	-25.76049252	5.977383414	-4.309660387	1.64E-05	0.001634551
PITA_000092341	Low quality	AT2G20370	MA_100374g0010	shade67>shade56	Encodes a xyloglucan galac	MURUS 3 (MUR3)	endomembrane syster	38.81090008	3.156730104	0.781356216	4.04006526	5.34E-05	0.004313407
PITA_000092371	High quality	AT4G27220	MA_10435615g0010	shade67<shade56	NB-ARC domain-containing disease resistance pro		ADP binding-GO:0043	109.9770456	-1.868025057	0.574391233	-3.2521824	0.001145225	0.040062839
PITA_000092506	High quality	AT2G40000	MA_98236g0010	shade67>shade56	ortholog of sugar beet HS1	ORTHOLOG OF SUGAR	cellular response to hy	21.59127542	19.91528822	6.043555638	3.295293269	0.00098319	0.035841625
PITA_000092582	High quality	AT5G44800	MA_10436824g0010	shade67>shade56	Interacts with transcriptior	CHROMATIN REMODE	nucleus-GO:0005634,p	79.03329567	21.19534669	5.978852347	3.545052705	0.000392535	0.017682425
PITA_000092746	High quality	AT5G48460	MA_10429910g0010	shade67<shade56	Encodes a member of the f	(ATFIM2)	actin filament-GO:000	71.67224667	-21.4371208	5.979118663	-3.585331218	0.000336651	0.015938478
PITA_000093169	High quality	AT5G01720	MA_10426597g0010	shade67>shade56	RAE1 is an F-box protein cc	REGULATION OF ATALI	nucleus-GO:0005634,p	37.93108022	21.41420239	5.98232458	3.579578825	0.000344148	0.016175599
PITA_000093339	Low quality	AT1G48950	MA_10437121g0010	shade67<shade56	C3HC zinc finger-like prote	(MEM1)	biological_process_unl	43.49185697	-9.495386412	1.650680766	-5.752406284	8.80E-09	4.97E-06
PITA_000093424	High quality	AT4G23140	MA_10253298g0010	shade67>shade56	Arabidopsis thaliana recep	CYSTEINE-RICH RLK (RE	chloroplast-GO:00095	295.2165577	23.11298962	6.035527746	3.829489415	0.000128409	0.00798473
PITA_000093533	High quality	AT4G22820	MA_211897g0010	shade67>shade56	A member of the A20/AN1	STRESS ASSOCIATED P	defense response to ot	72.04231012	22.12354304	3.581750998	6.176739546	6.54E-10	6.30E-07
PITA_000093555	Low quality	AT2G22795	MA_649817g0010	shade67>shade56	hypothetical protein;(source:Araport11)		biological_process_unl	133.5551604	20.95206192	6.036127798	3.471109728	0.000518312	0.02187008
PITA_000093588	Low quality	AT5G45190	MA_10111260g0010	shade67>shade56	Encodes a cyclin T partner	CYCT1;5. Plays importa	cyclin/CDK positive tra	275.3030627	20.61485656	6.036127057	3.415245632	0.000637245	0.025481964
PITA_000093795	High quality	AT1G02130	MA_101924g0020	shade67>shade56	Belongs to the Rab1 GTPas	RAS 5 (RA-5)	endoplasmic reticulum	53.86507163	1.618800597	0.455293606	3.555509178	0.000377248	0.017341715
PITA_000093955	High quality	AT4G31940	MA_10055697g0010	shade67>shade56	The gene encodes a cytoch	CYTOCHROME P450, F.	cellular response to iro	1363.452767	1.401921833	0.2715044	5.163532646	2.42E-07	7.78E-05
PITA_000094255	Low quality	AT3G01740	MA_10431392g0010	shade67>shade56	Mitochondrial ribosomal protein L37;(source:Arap		biological_process_unl	107.986113	23.23629554	6.036444758	3.849334579	0.000118439	0.007563809
PITA_000094496	High quality	AT1G19835	MA_10429332g0010	shade67>shade56	TCS1 encodes a coiled-coil	TRICHOME CELL SHAPI	cortical microtubule-G	33.94715311	22.73197536	5.983440794	3.799147705	0.000145195	0.0087555
PITA_000095263	High quality	AT5G48385	MA_10436820g0010	shade67<shade56	FRIGIDA-like protein;(source:Araport11)		nucleus-GO:0005634,p	76.62473525	-20.57054196	5.979356615	-3.440260096	0.000581155	0.023750615
PITA_000095346	High quality	AT1G03100	MA_118036g0010	shade67>shade56	Pentatricopeptide repeat (PPR) superfamily protei		biological_process_unl	88.94421728	22.01827834	5.980474855	3.681693991	0.000231689	0.0121658
PITA_000095775	Low quality	AT3G44680	MA_97739g0010	shade67>shade56	Encodes HDA9 (a RPD3-like	HISTONE DEACETYLASI	cytoplasm-GO:000573	96.67771722	25.2459185	6.038737463	4.180661711	2.91E-05	0.002669571
PITA_000096285	High quality	AT1G75290	MA_16892g0010	shade67>shade56	encodes a protein whose sequence is similar to an		chloroplast-GO:00095	191.2809067	30	6.150543768	4.877617514	1.07E-06	0.000151672
PITA_000096312	High quality	AT5G62040	MA_102863g0010	shade67>shade56	BFT is a member of The FL	BROTHER OF FT AND T	cytoplasm-GO:000573	97.19570579	20.94971567	6.036441072	3.470540907	0.000519411	0.021880934
PITA_000096542	Low quality	AT4G31850	MA_103898g0010	shade67>shade56	encodes a protein containi	PROTON GRADIENT RE	mRNA binding-GO:000	226.7104038	23.45734243	5.072273272	4.624621186	3.75E-06	0.000447448
PITA_000096590	High quality	AT5G50260	MA_103463g0010	shade67<shade56	Encodes a papain-like cyste	CYSTEINE ENDOPEPTIE	anther wall tapetum de	133.1478986	-22.47700013	5.775776728	-3.891597822	9.96E-05	0.006654101
PITA_000096601	Low quality	AT5G53760	MA_211101g0010	shade67>shade56	A member of a large family	MILDEW RESISTANCE I	nucleus-GO:0005634	39.95270954	20.22067456	4.531951343	4.461803102	8.13E-06	0.000906629

Figure S1 Map of Sweden on the left. The pictures to the right describe how the light quality differs from south (bottom picture) to the north (top picture) throughout the year in Sweden. The black areas are darkness (night), blue area is twilight primarily containing far-red light and the yellow area is day light. The x-axis is a 24h cycle and the y-axis is divided into the months of the year beginning January. Adopted from (Abrahamsson, 2011)

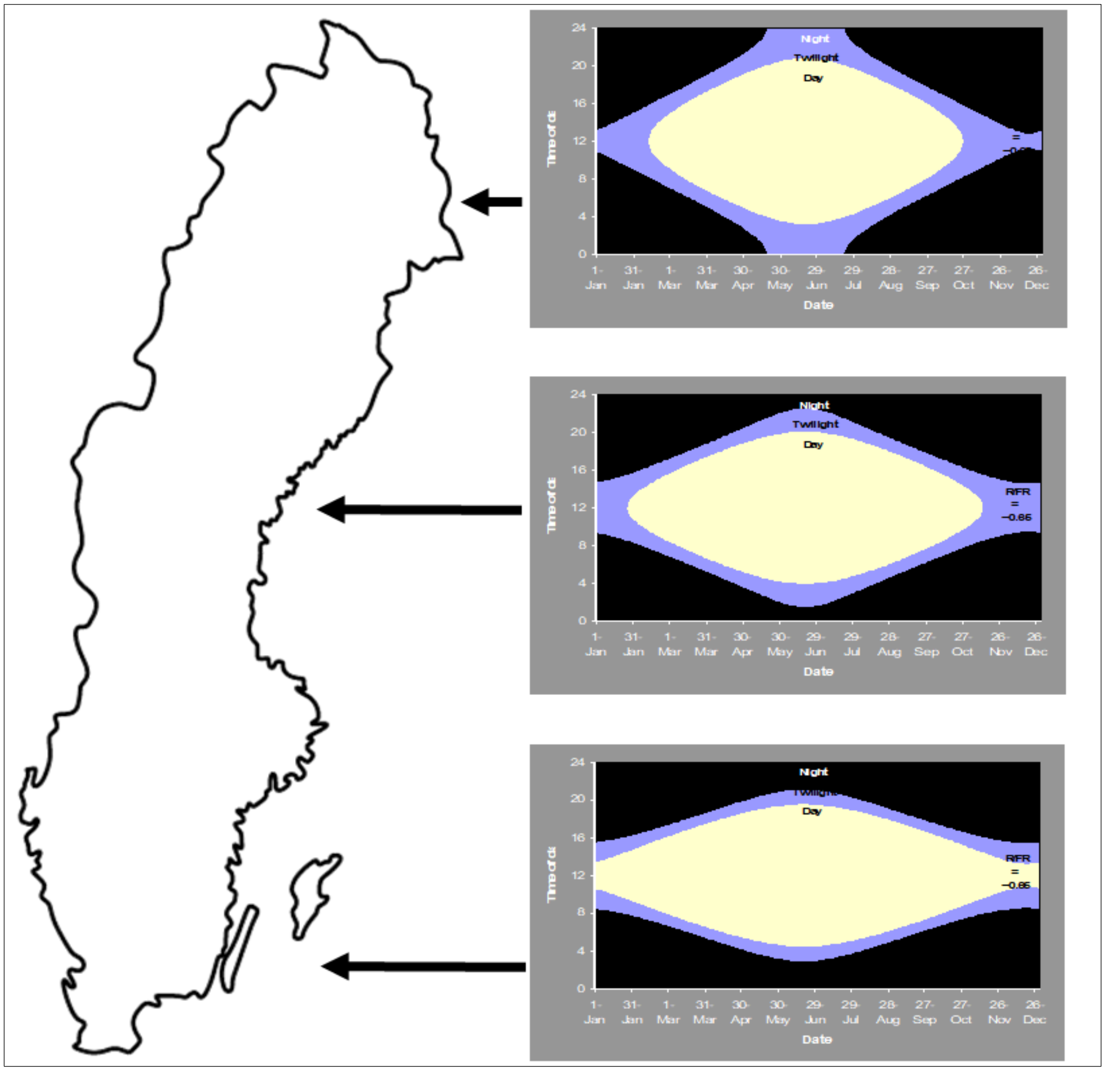

Abrahamsson, S., 2011. Genetic Dissection of Quantitative Traits in Scots Pine. In, Department of Forest Genetics and Plant Physiology. Swedish University of Agricultural Sciences, Umeå.

Figure S2 Venn diagram of the differentially expressed genes under SUN and SHADE for the within latitude comparison in the southern and northern Scots pine population, respectively. 56 SHADE>SUN denotes genes that were up-regulated under SHADE as compared to the SUN, while 56 SHADE<SUN denotes genes that were down-regulated under SHADE as compared to the SUN in southern population. 67 SHADE>SUN denotes genes that were up-regulated under SHADE as compared to the SUN, while 67 SHADE<SUN denotes genes that were down-regulated under SHADE as compared to the SUN in northern population.

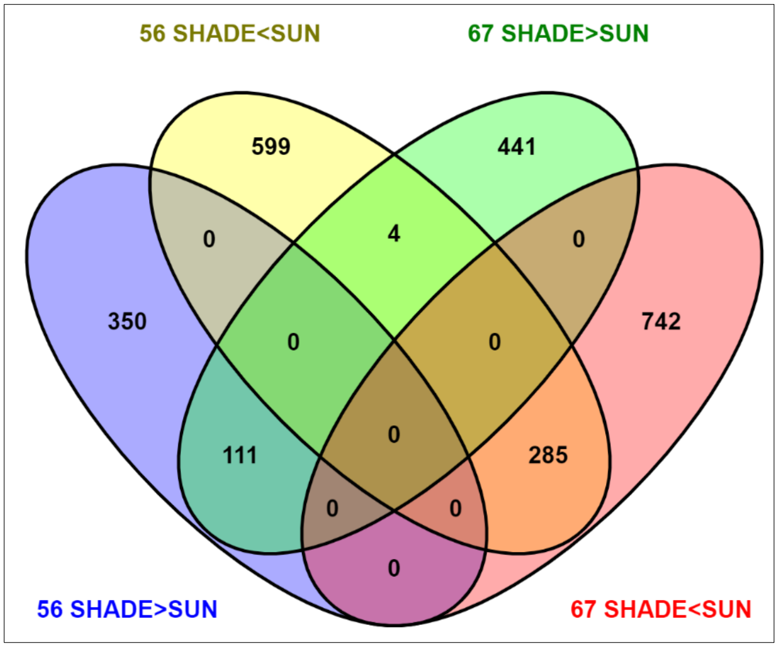

Under SHADE, there was a significantly higher number of genes down-regulated for both the within latitude comparisons (*p-value* < 0.05). 888 genes were down-regulated, and 461 genes were up-regulated under SHADE in the southern population, while 1027 genes were down-regulated and 556 genes were up-regulated under SHADE in the northern population. The north versus south comparison revealed significantly higher number of up-regulated genes (289 genes) under SHADE in the north as compared to the south (136 genes) (*p-value* > 0.05).

Functional categorization by annotation for Gene Ontology (GO) Biological Process, GO Cellular Component and GO Molecular Function, for the differentially regulated genes from respective treatments and comparisons.

Percentage denotes number of annotations to terms in the GOslim category * 100 / number of total annotations to terms in the ontology. Number in the parenthesis denotes number of genes in the respective functional category.

Within latitude comparison in Scots pine in southern population: SHADE was the treatment condition and SUN was used as control. Shade56>Sun56 denotes genes that were up-regulated under SHADE as compared to the SUN in southern population. Shade56<Sun56 denotes genes that were down-regulated under SHADE as compared to the SUN in southern population.

Figure S3 GO Biological Process: Shade56>Sun56

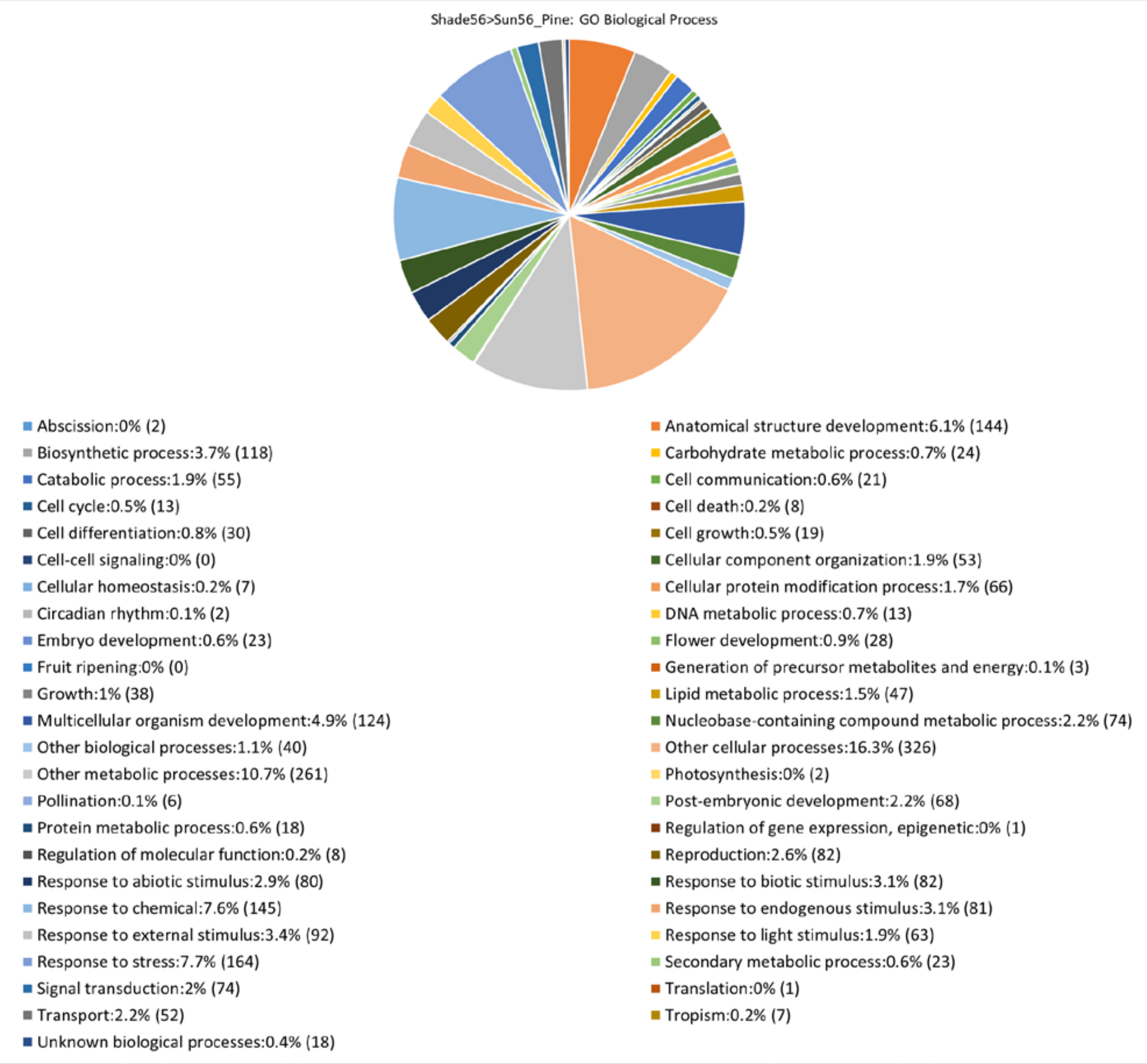

Figure S4 GO Biological Process: Shade56<Sun56

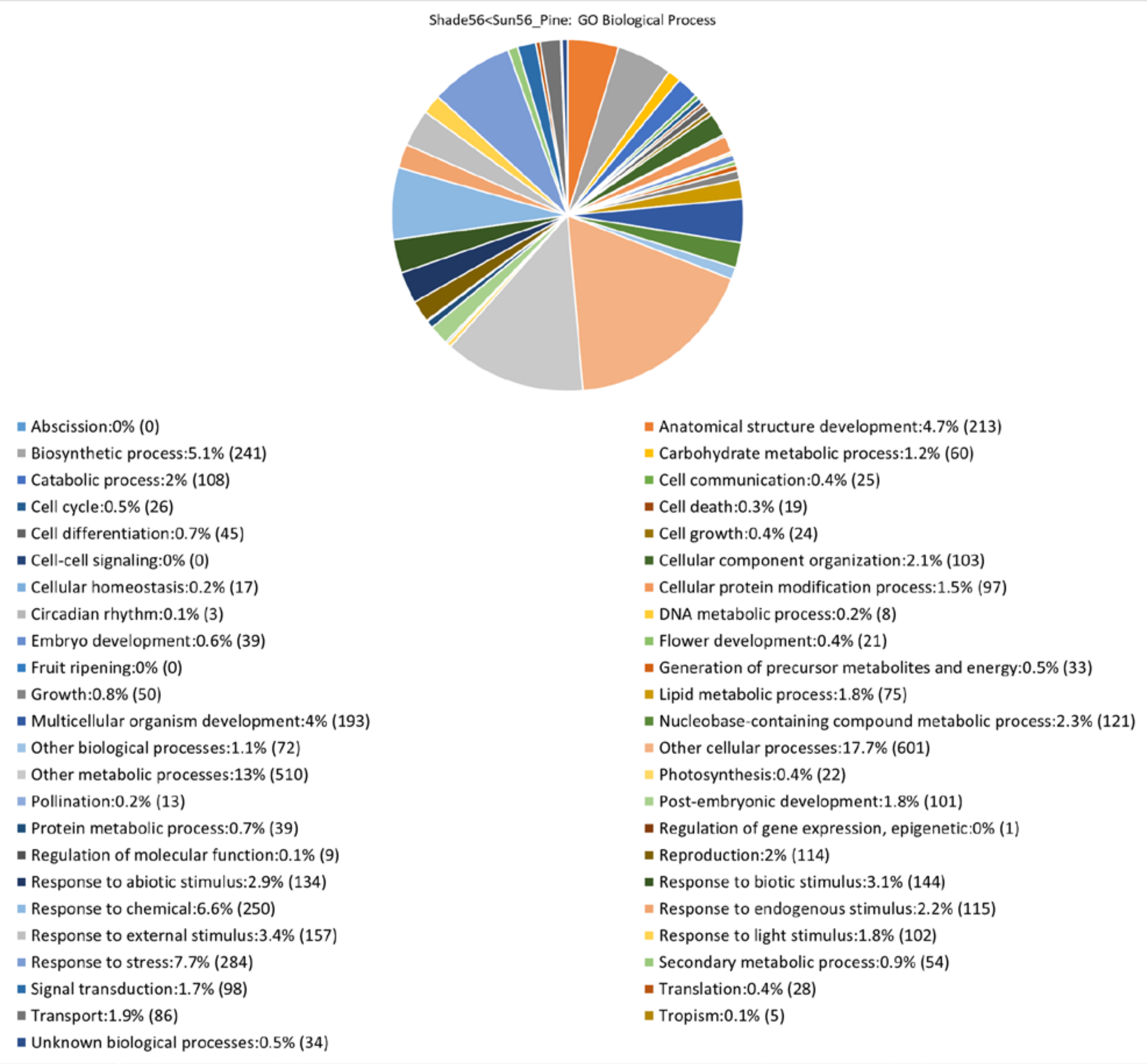

Figure S5 GO Cellular Component: Shade56>Sun56

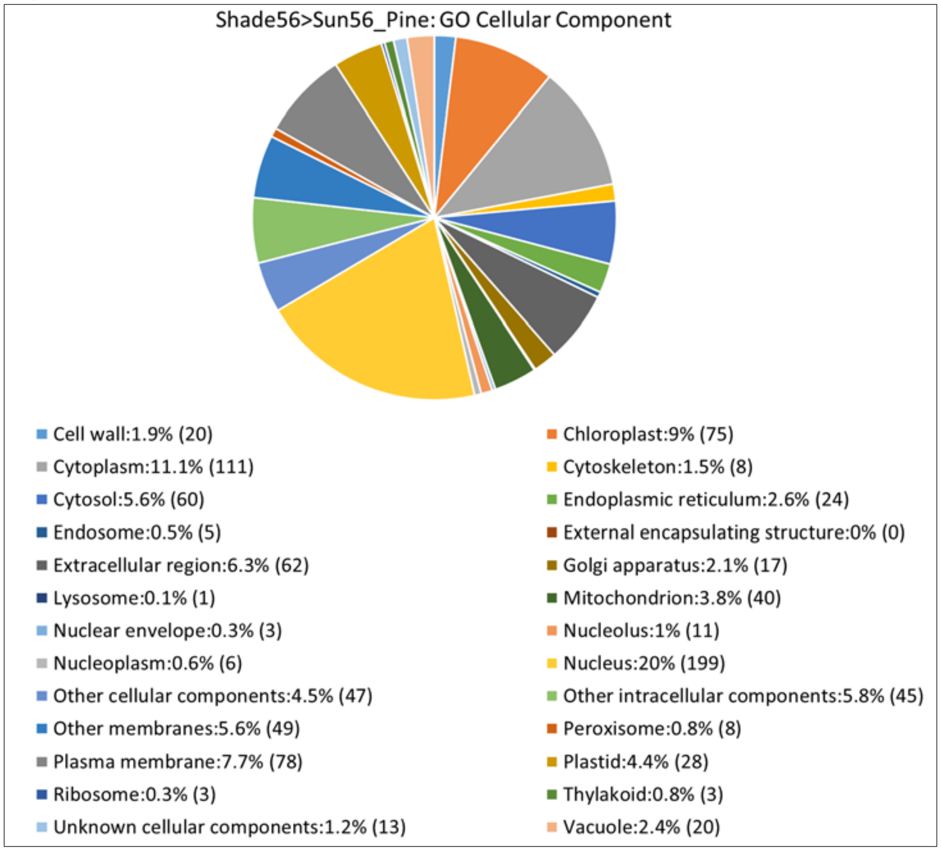

Figure S6 GO Cellular Component: Shade56<Sun56

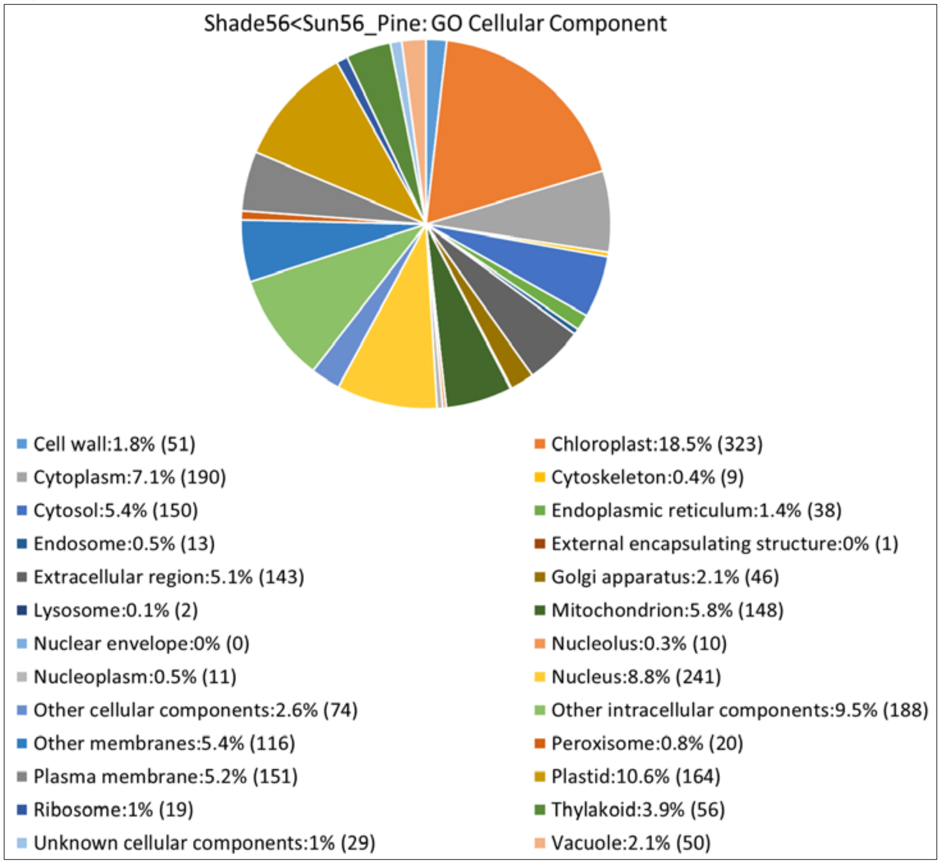

Figure S7 GO Molecular Function: Shade56>Sun56

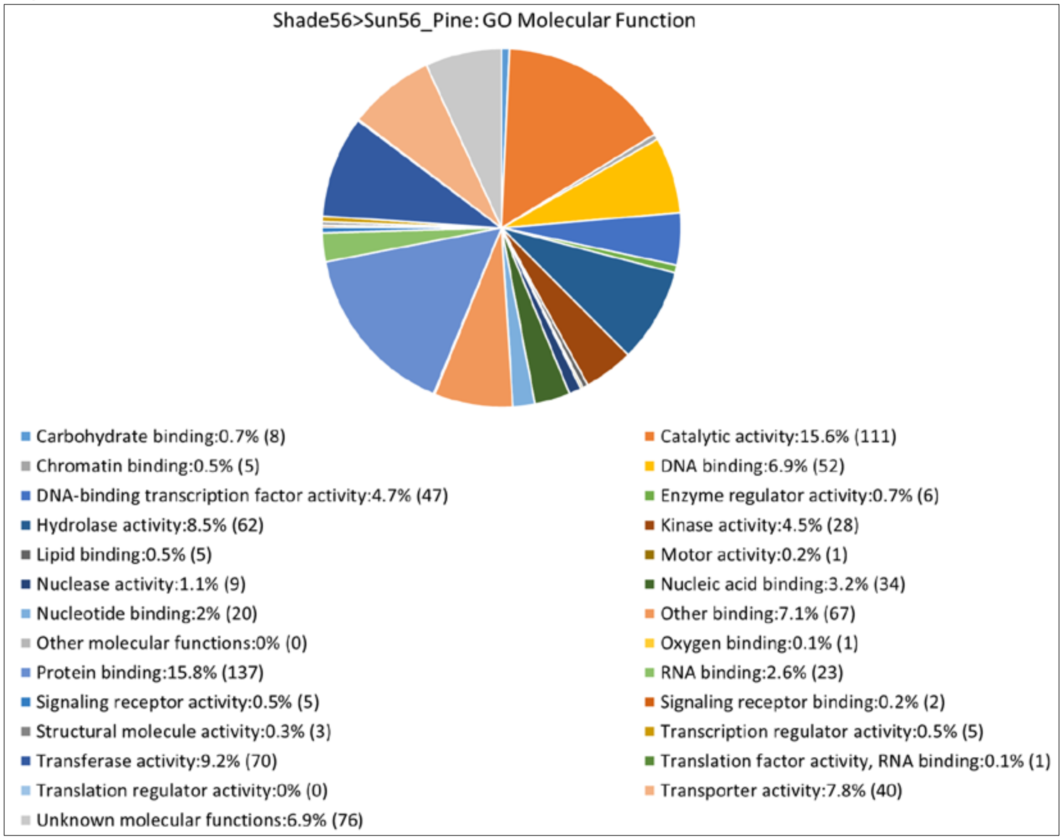

Figure S8 GO Molecular Function: Shade56<Sun56

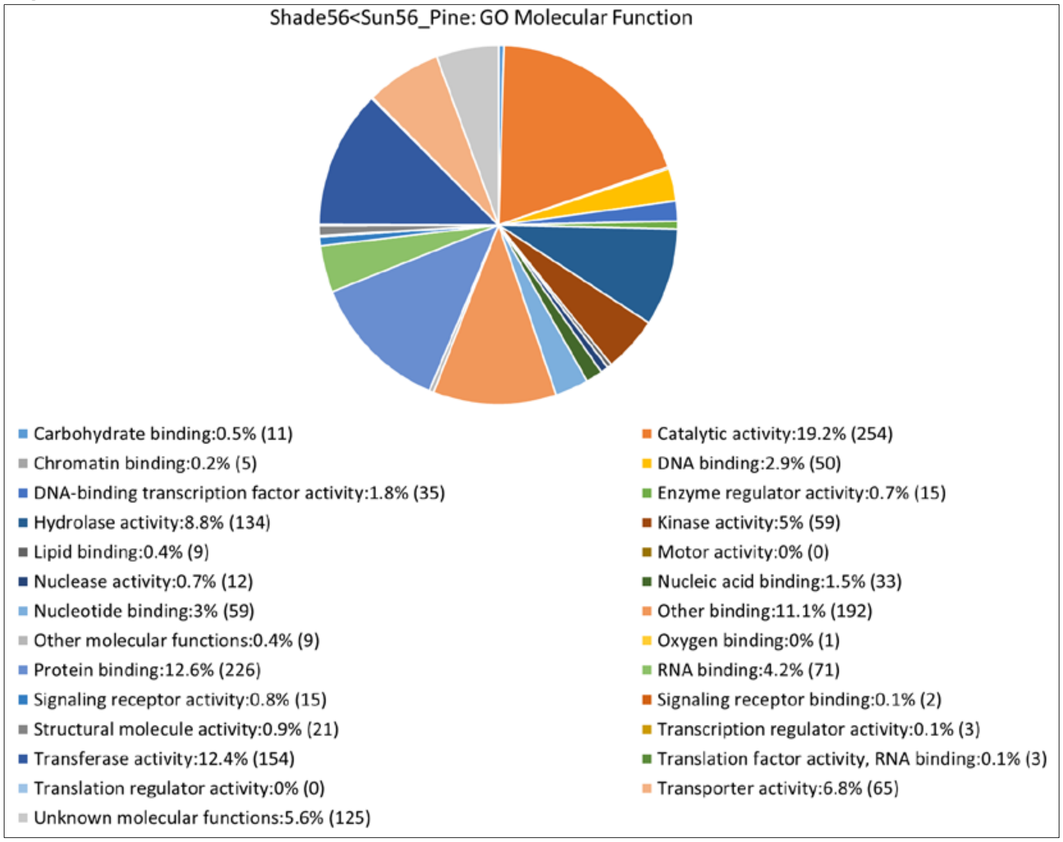

<u>Within latitude comparison in Scots pine in northern population:</u> SHADE was the treatment condition and SUN was used as control. Shade67>Sun67 denotes genes that were up-regulated under SHADE as compared to the SUN in northern population. Shade67<Sun67 denotes genes that were down-regulated under SHADE as compared to the SUN in northern population.

Figure S9 GO Biological Process: Shade67>Sun67

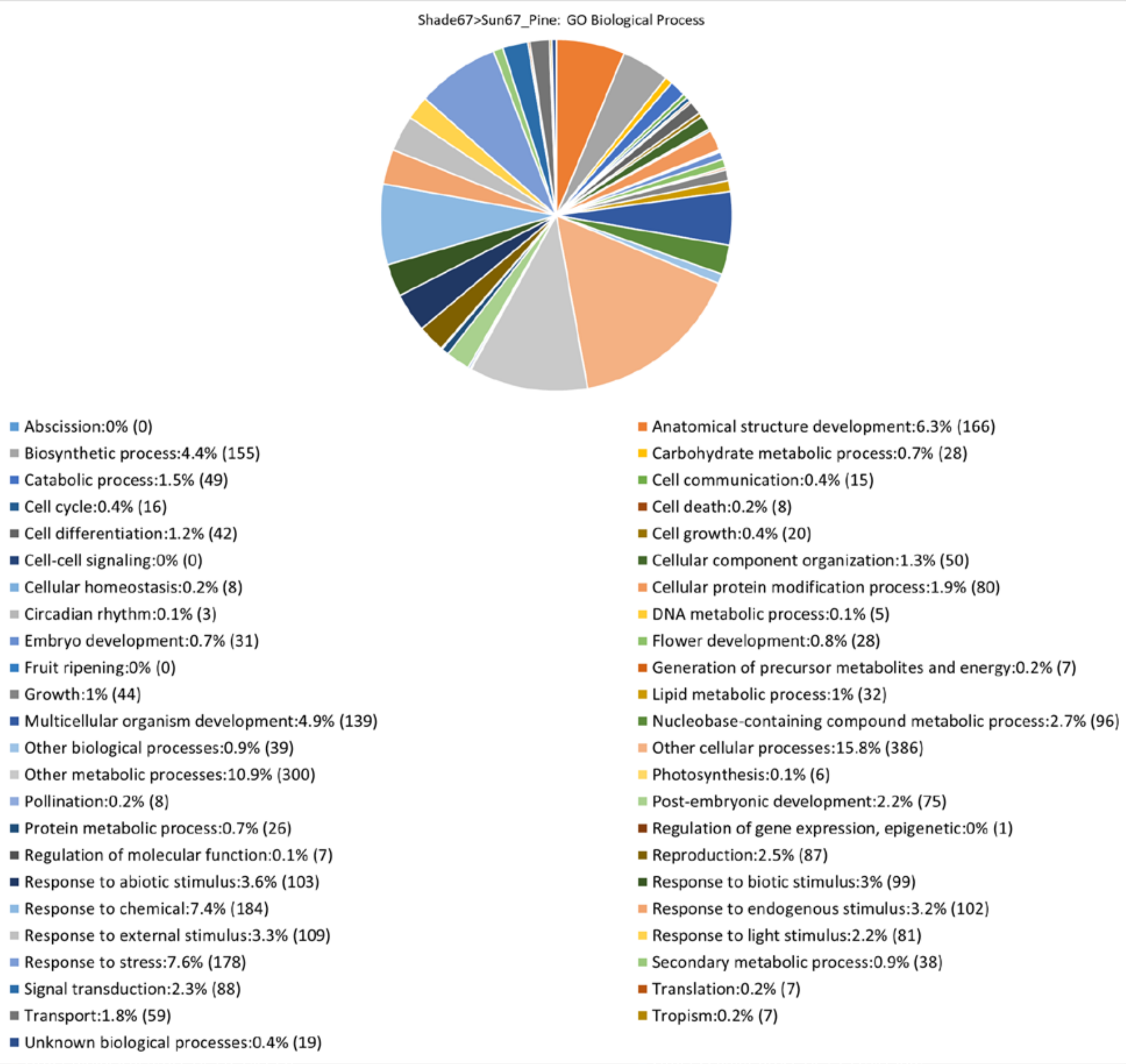

Figure S10 GO Biological Process: Shade67<Sun67

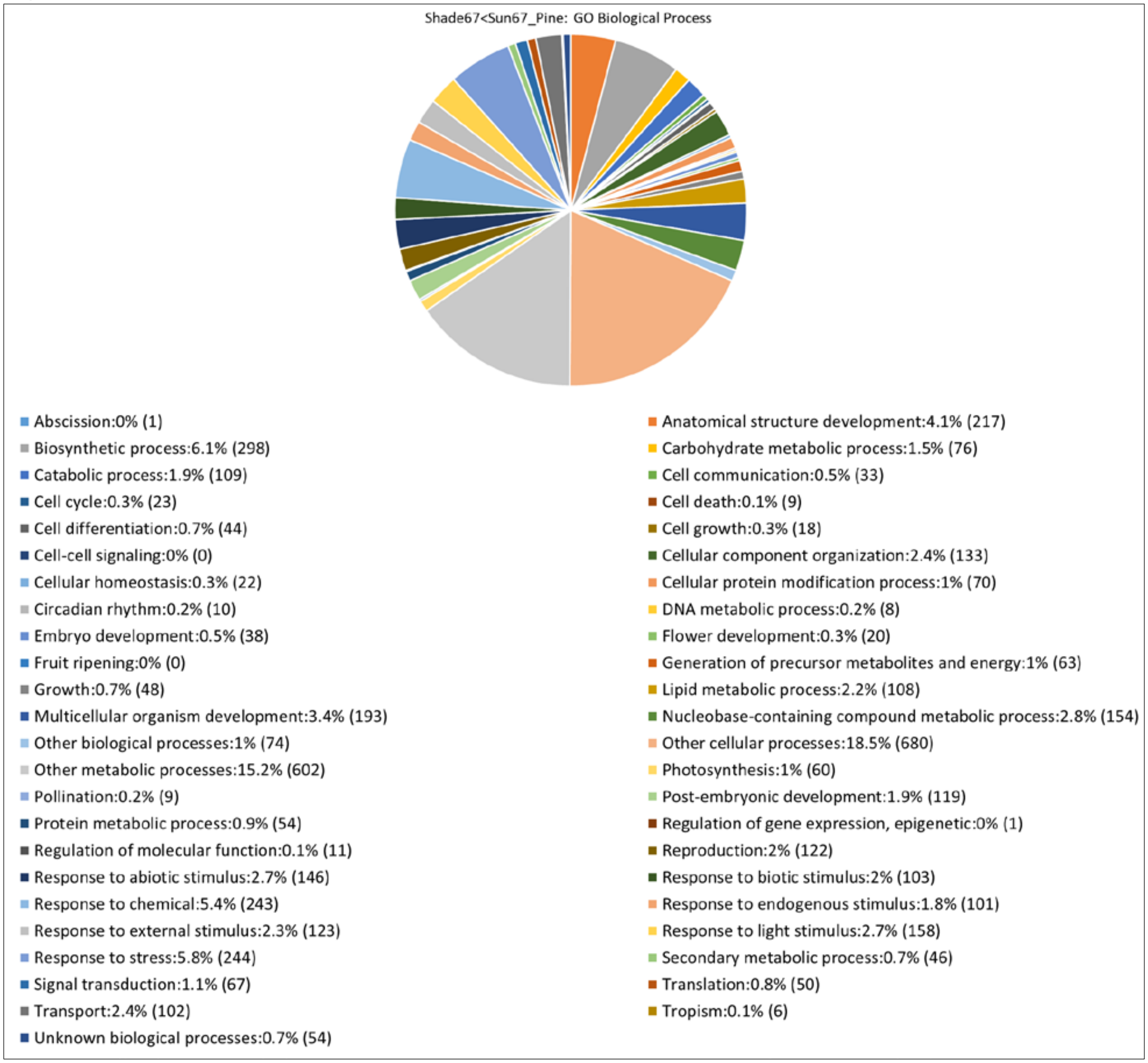

Figure S11 GO Cellular Component: Shade67>Sun67

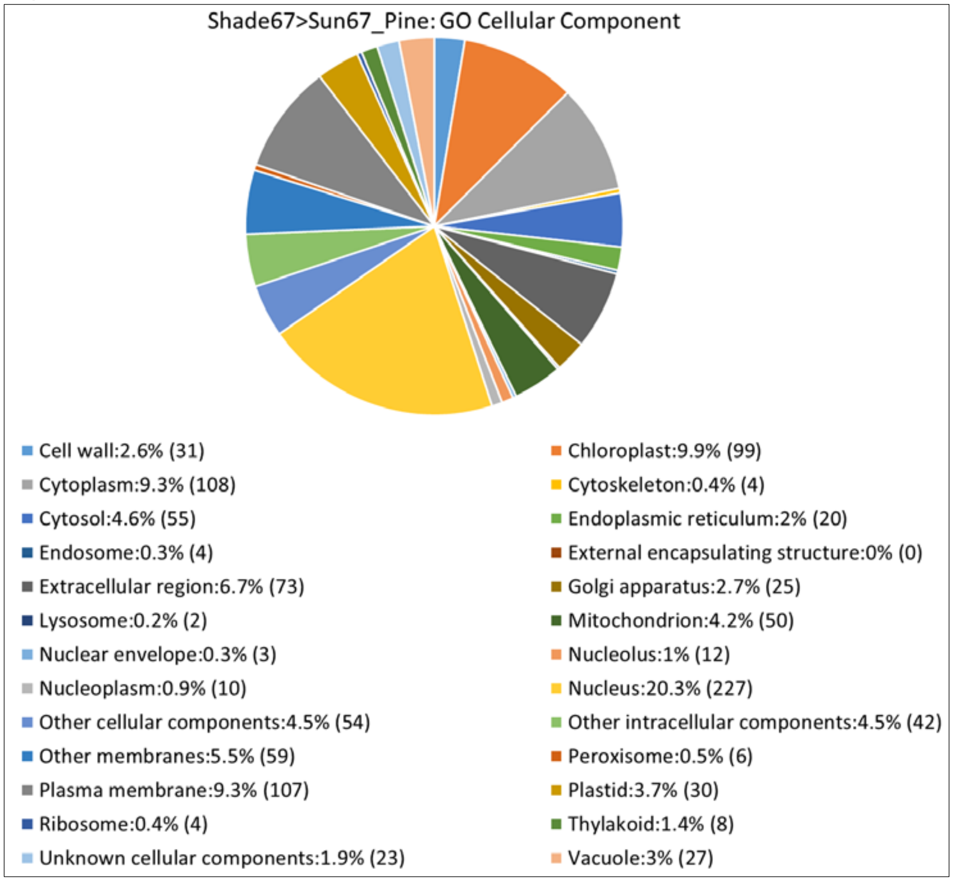

Figure S12 GO Cellular Component: Shade67<Sun67

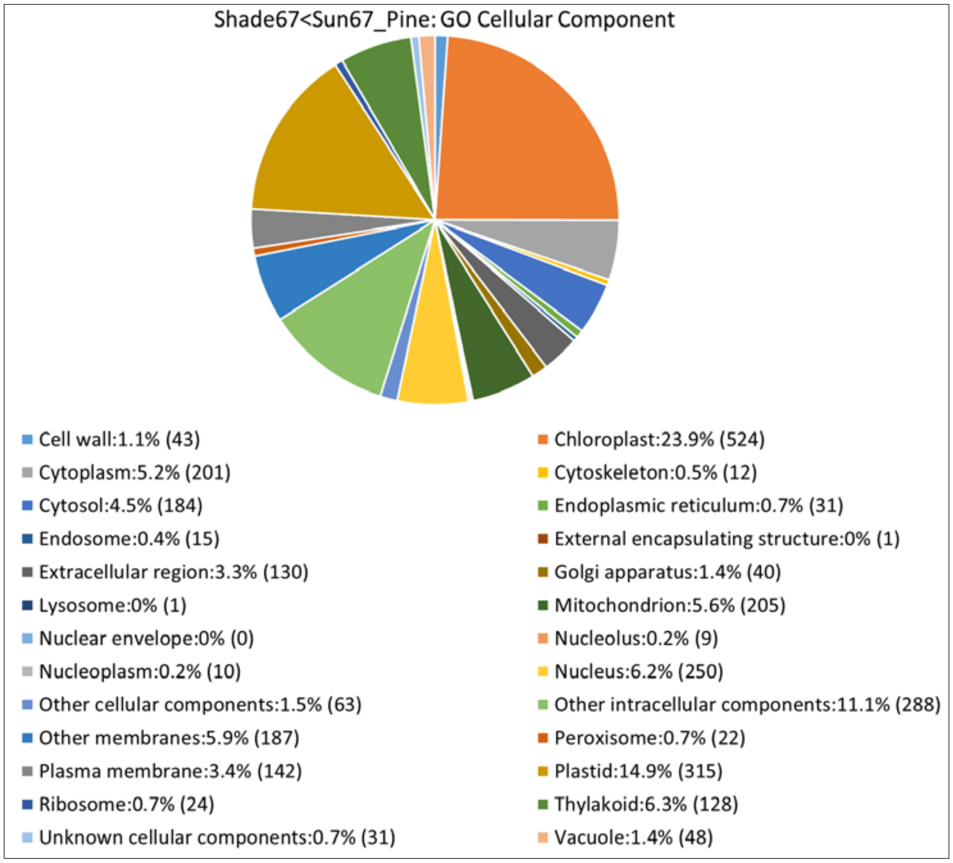

Figure S13 GO Molecular Function: Shade67>Sun67

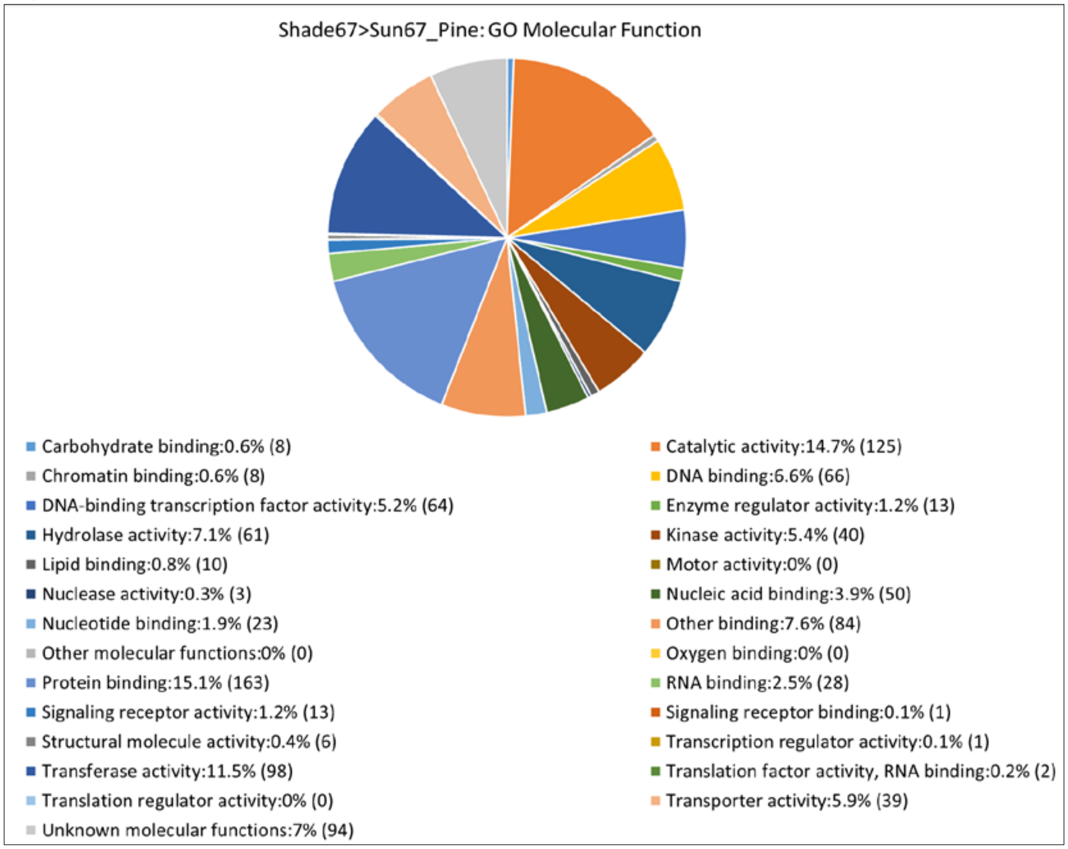

Figure S14 GO Molecular Function: Shade67<Sun67

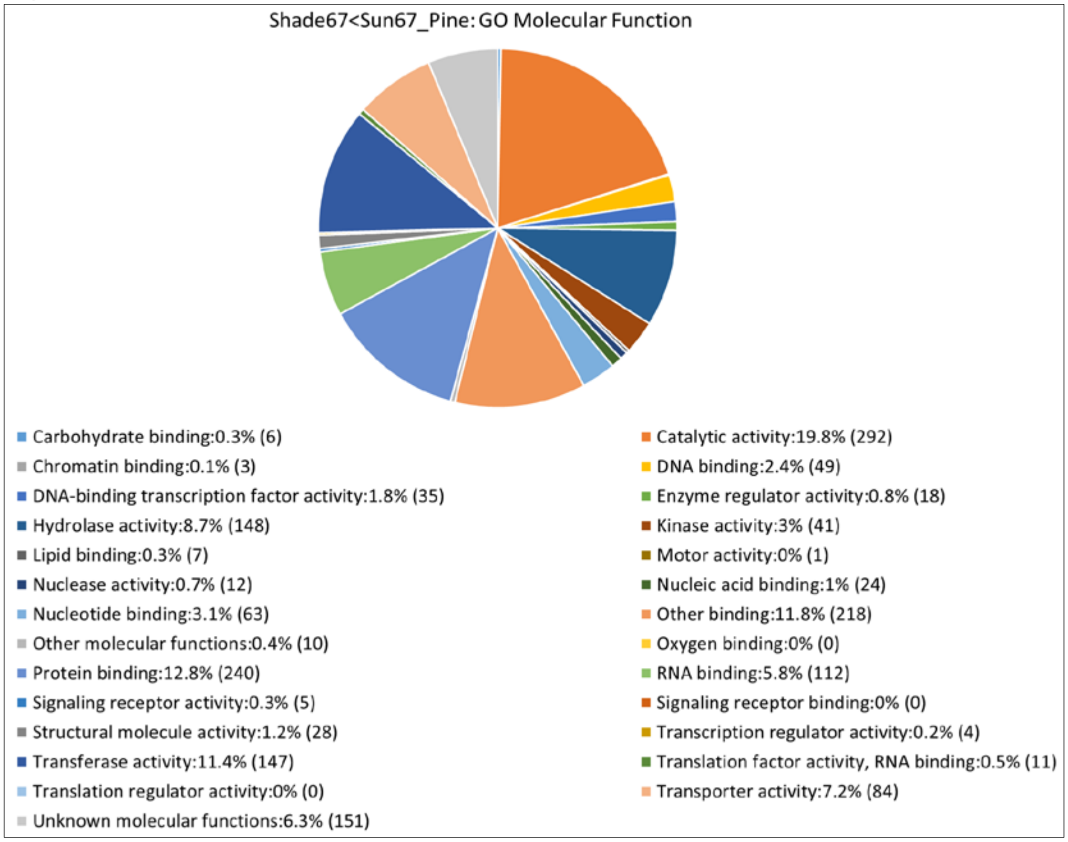

<u>North versus south latitude comparison in Scots pine:</u> North versus south comparison was performed using two-factor design: design=~ treatment + latitude + treatment*latitude. SHADE was the treatment condition and SUN was used as control. Shade67>Shade56 denotes genes that up-regulated under SHADE in the northern population as compared to the southern population. Shade67<Shade56 denotes genes that up-regulated under SHADE in the southern population as compared to the northern population.

Figure S15 GO Biological Process: Shade67>Shade56

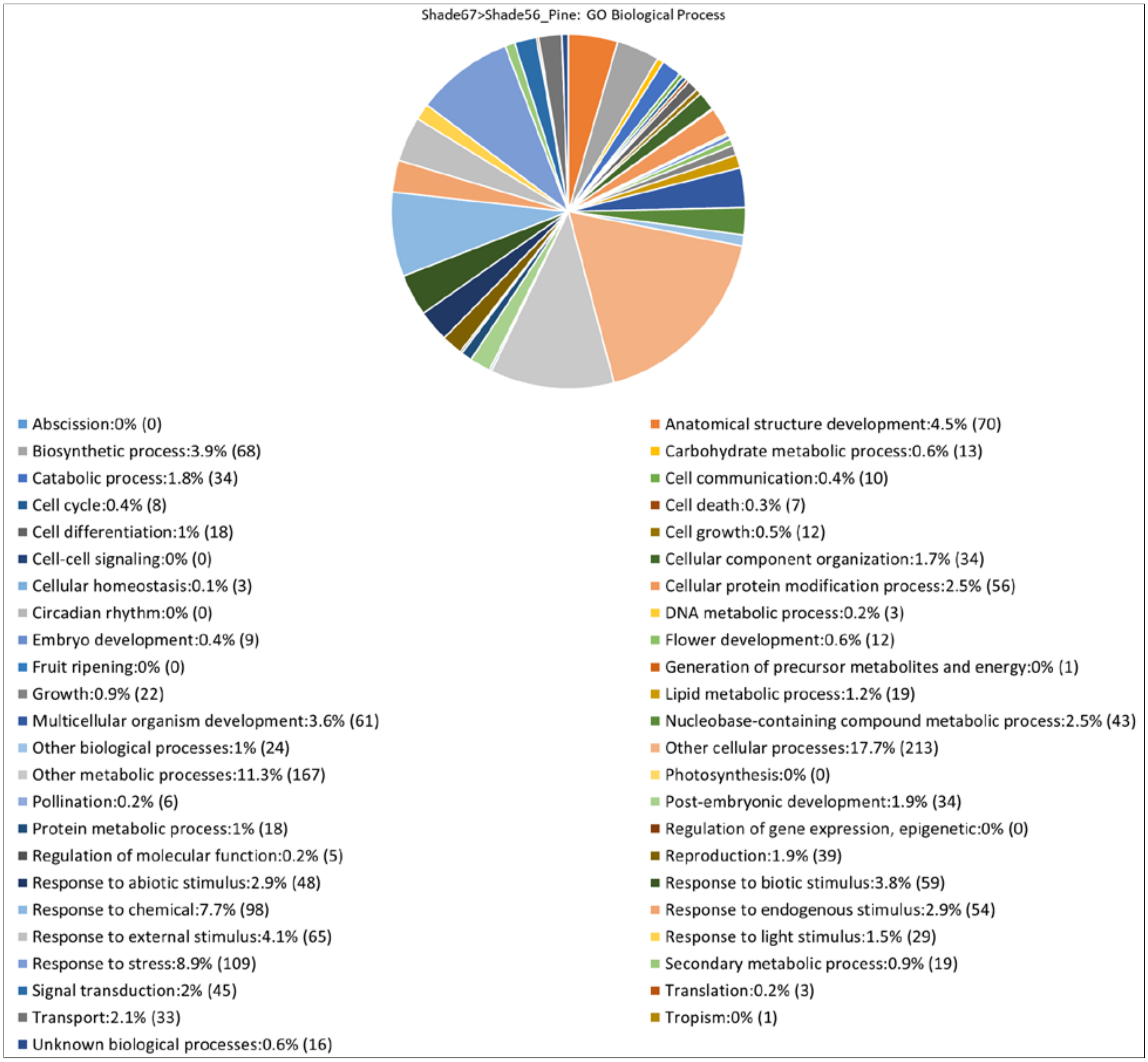

Figure S16 GO Biological Process: Shade67<Shade56

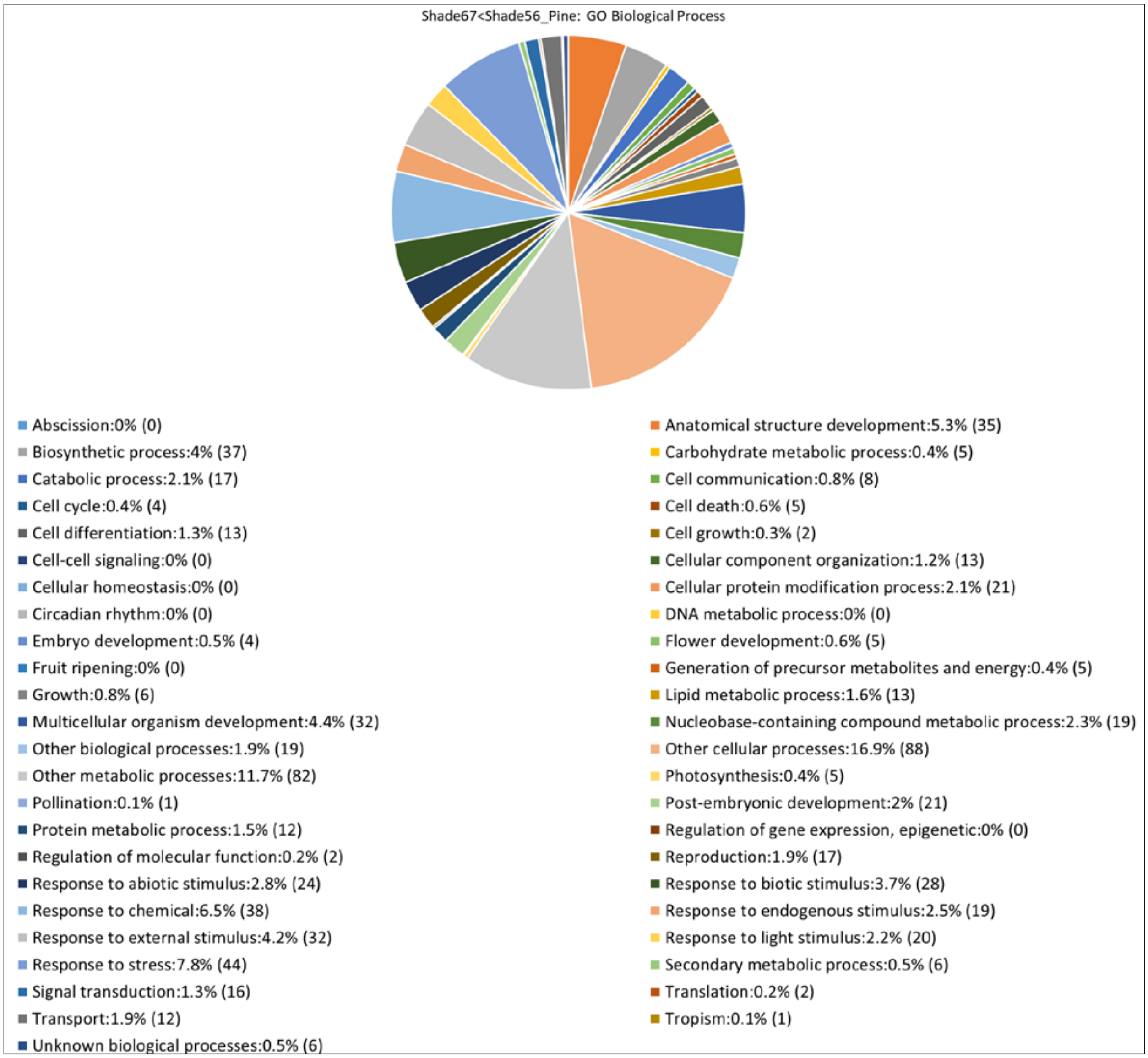

Figure S17 GO Cellular Component: Shade67>Shade56

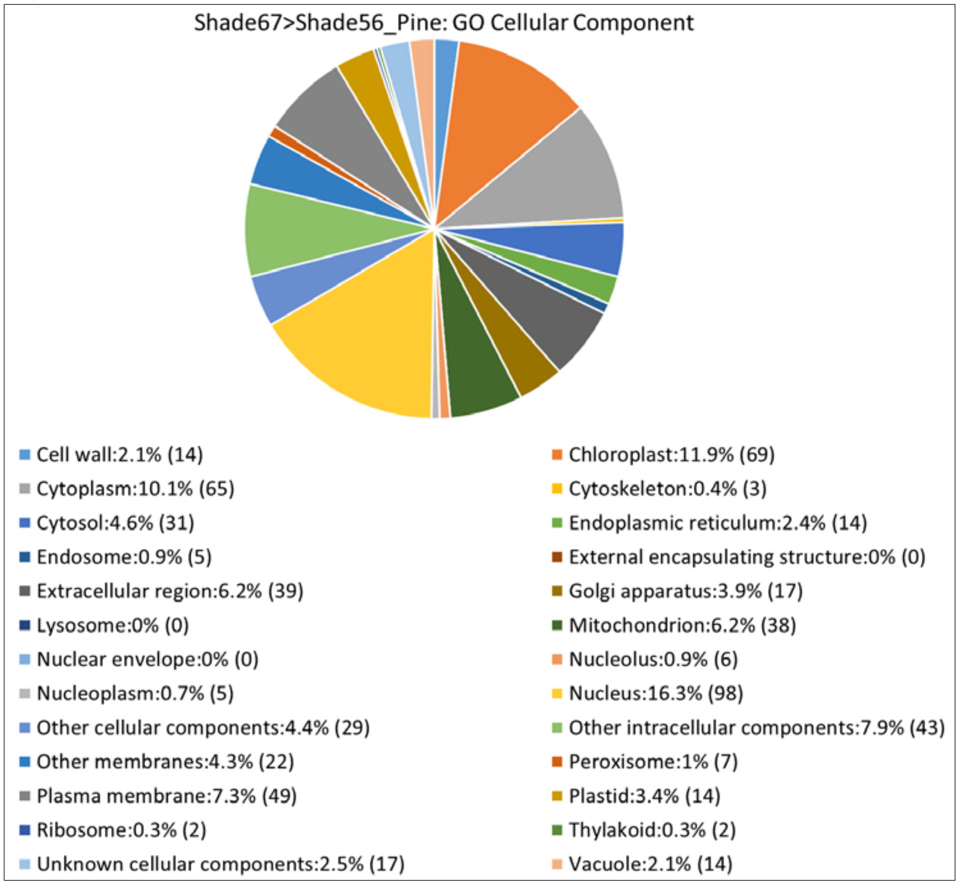

Figure S18 GO Cellular Component: Shade67<Shade56

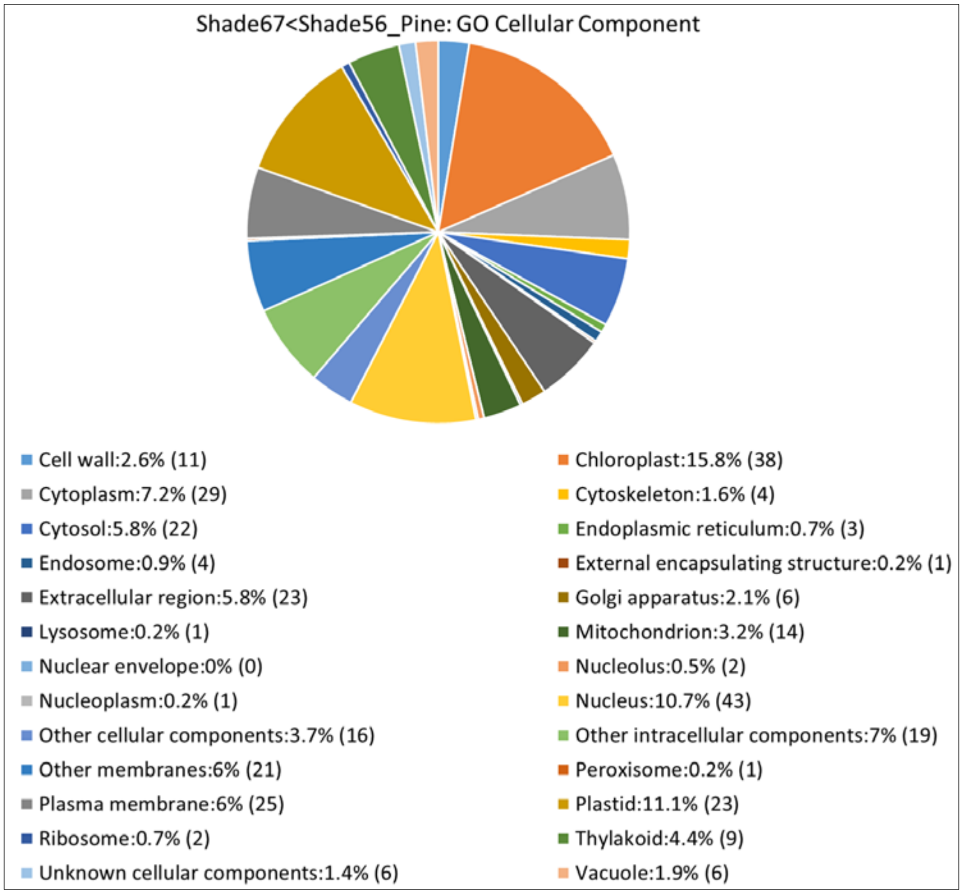

Figure S19 GO Molecular Function: Shade67>Shade56

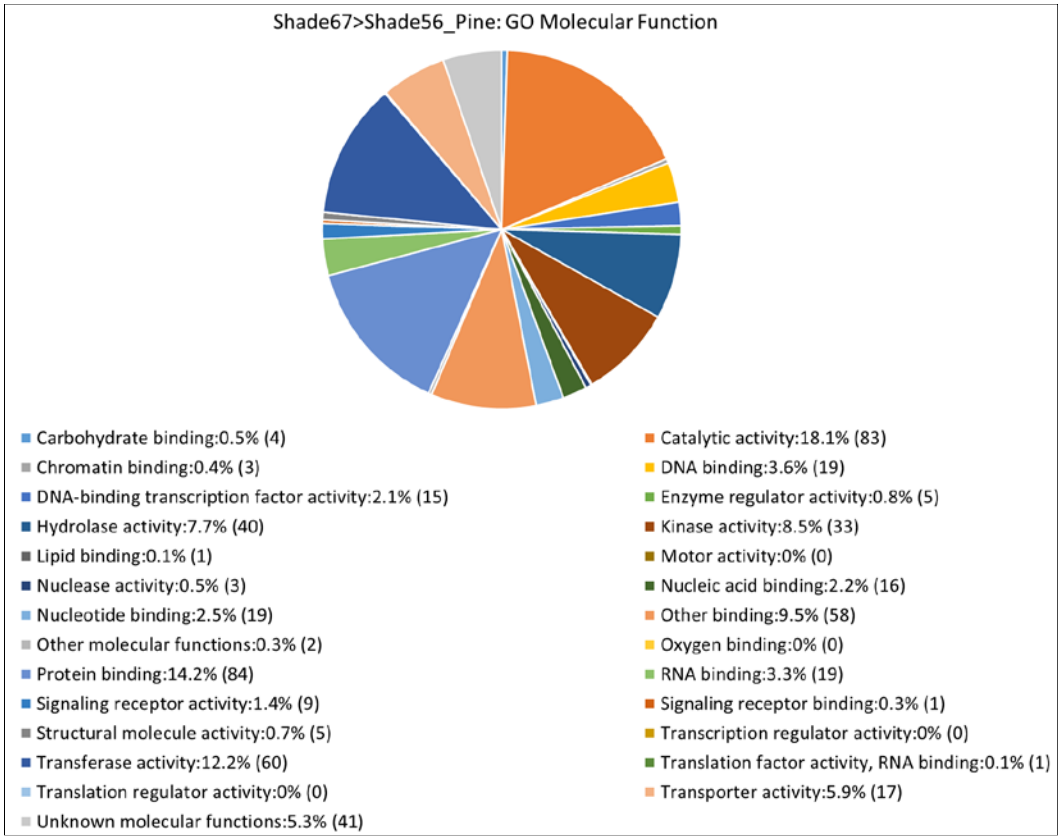

Figure S20 GO Molecular Function: Shade67< Shade56

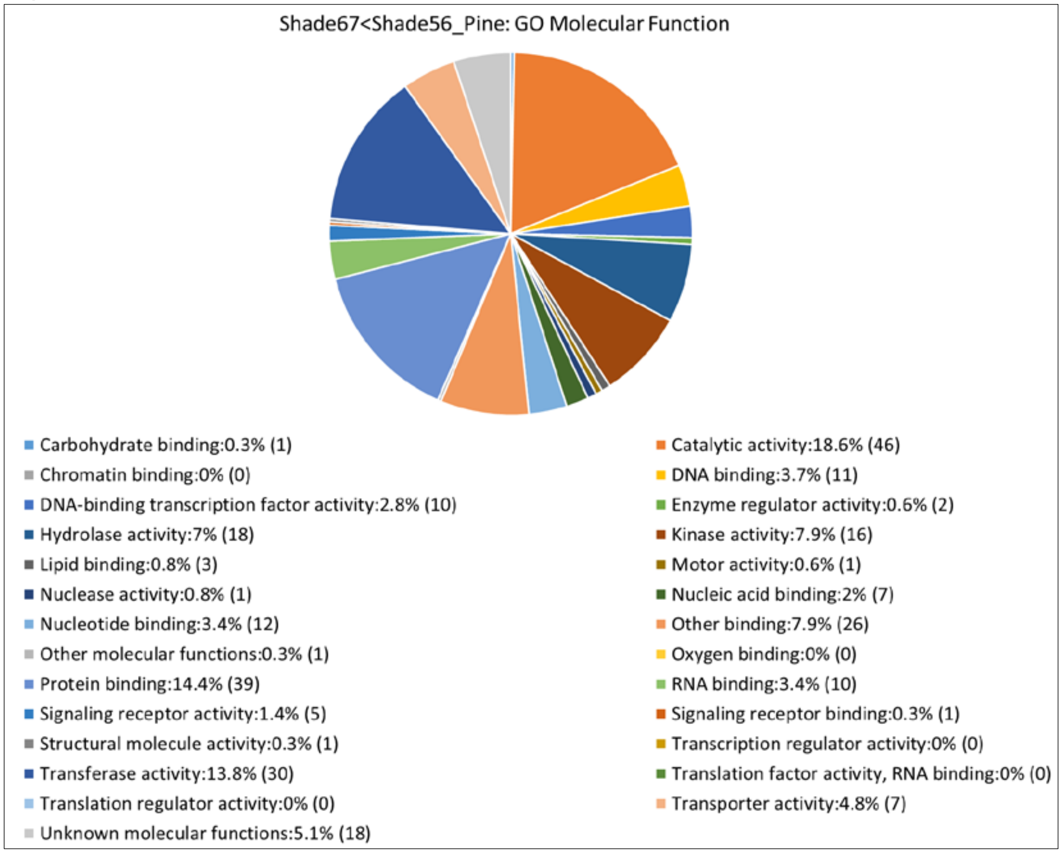

<u>Gene Ontology (GO) enrichment analysis of top 30 pathways for the differentially regulated genes from respective treatments and comparisons (*p-value* cutoff (FDR): 0.05): GO Biological Process, GO Cellular Component and GO Molecular Function</u>

<u>Within latitude comparison in Scots pine in southern population:</u> SHADE was the treatment condition and SUN was used as control. Shade56>Sun56 denotes genes that were up-regulated under SHADE as compared to the SUN in southern population. Shade56<Sun56 denotes genes that were down-regulated under SHADE as compared to the SUN in southern population.

Figure S21 GO Biological Process: Shade56>Sun56

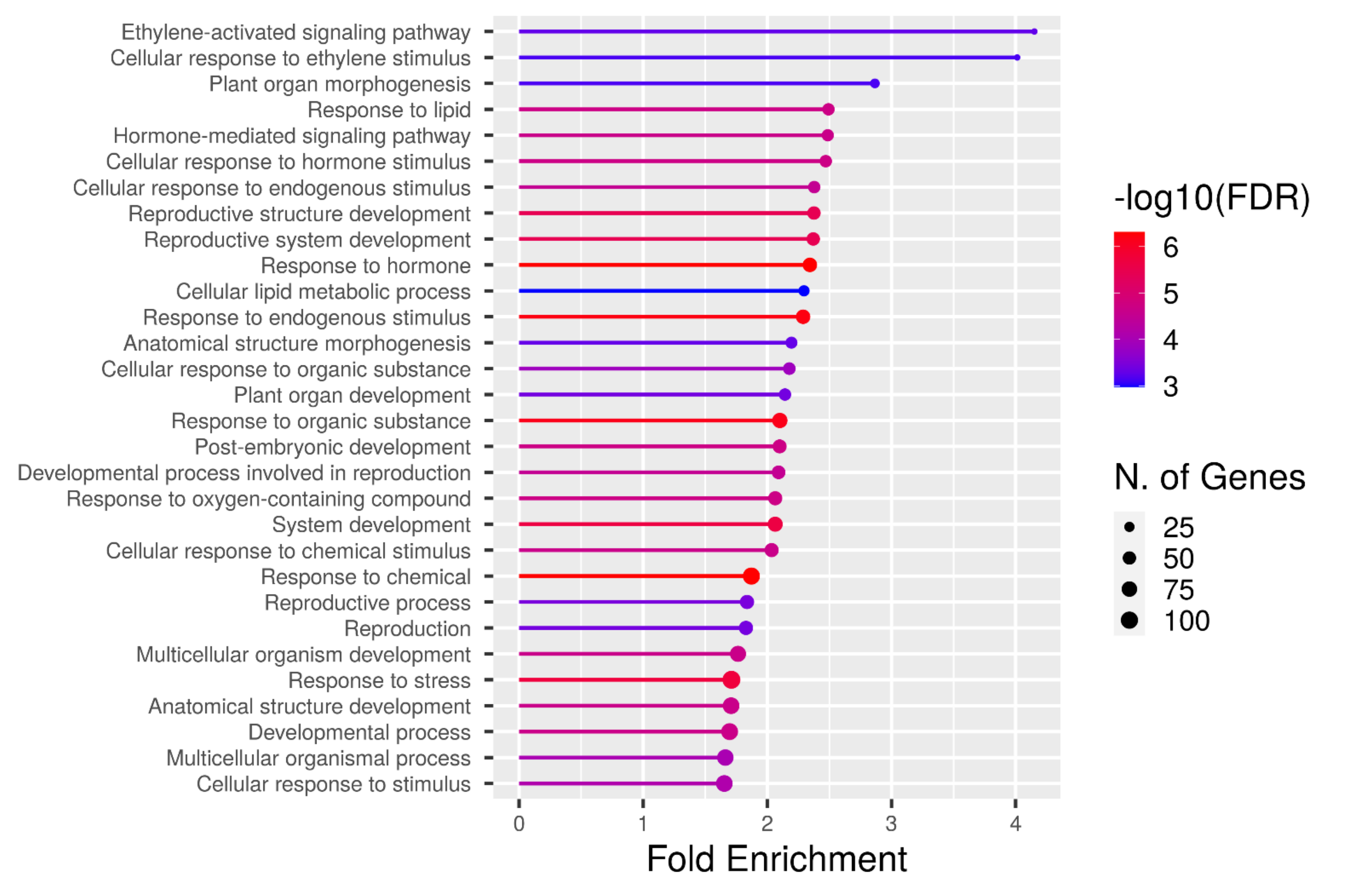

Figure S22 GO Biological Process: Shade56<Sun56

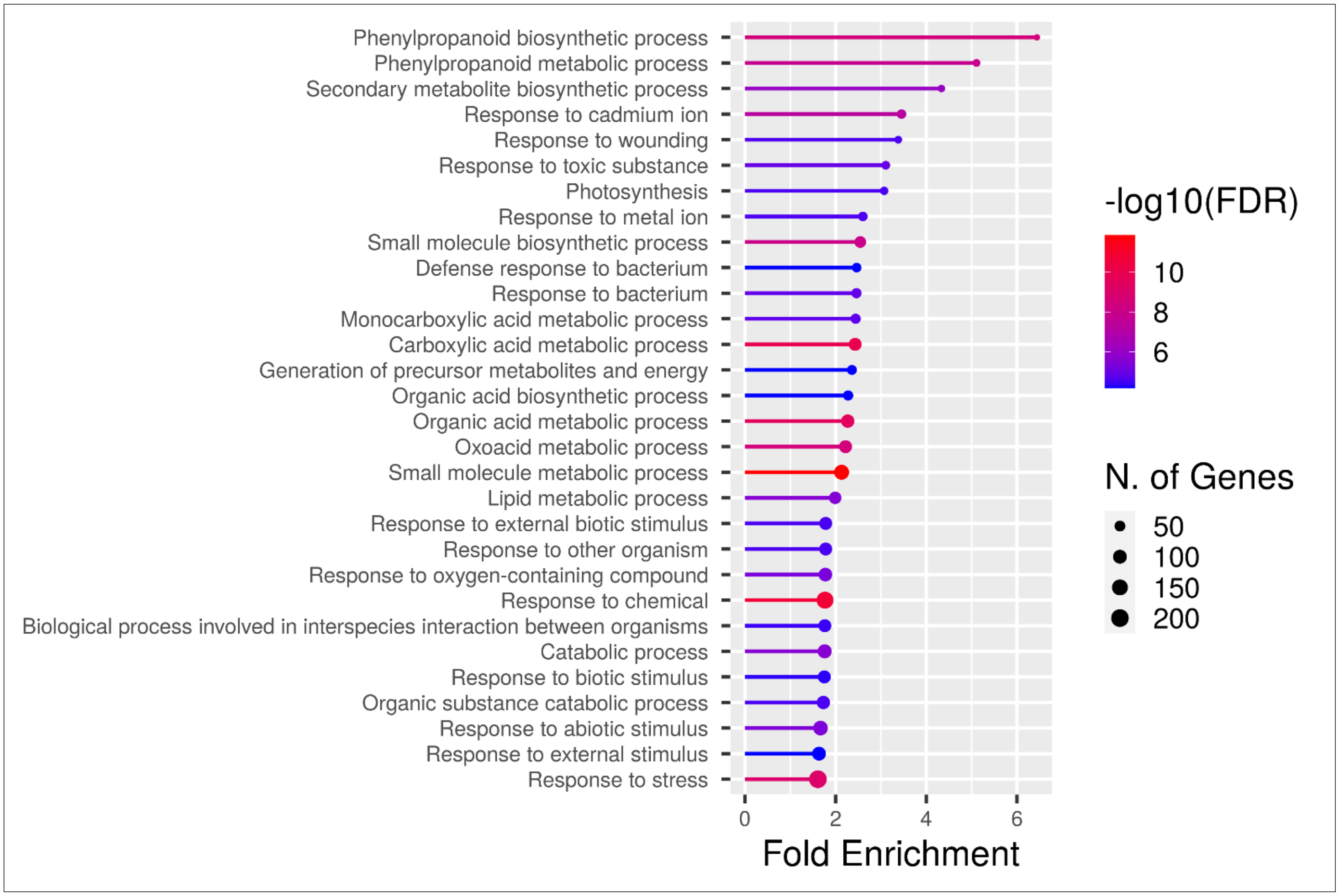

Figure S23 GO Cellular Component: Shade56>Sun56

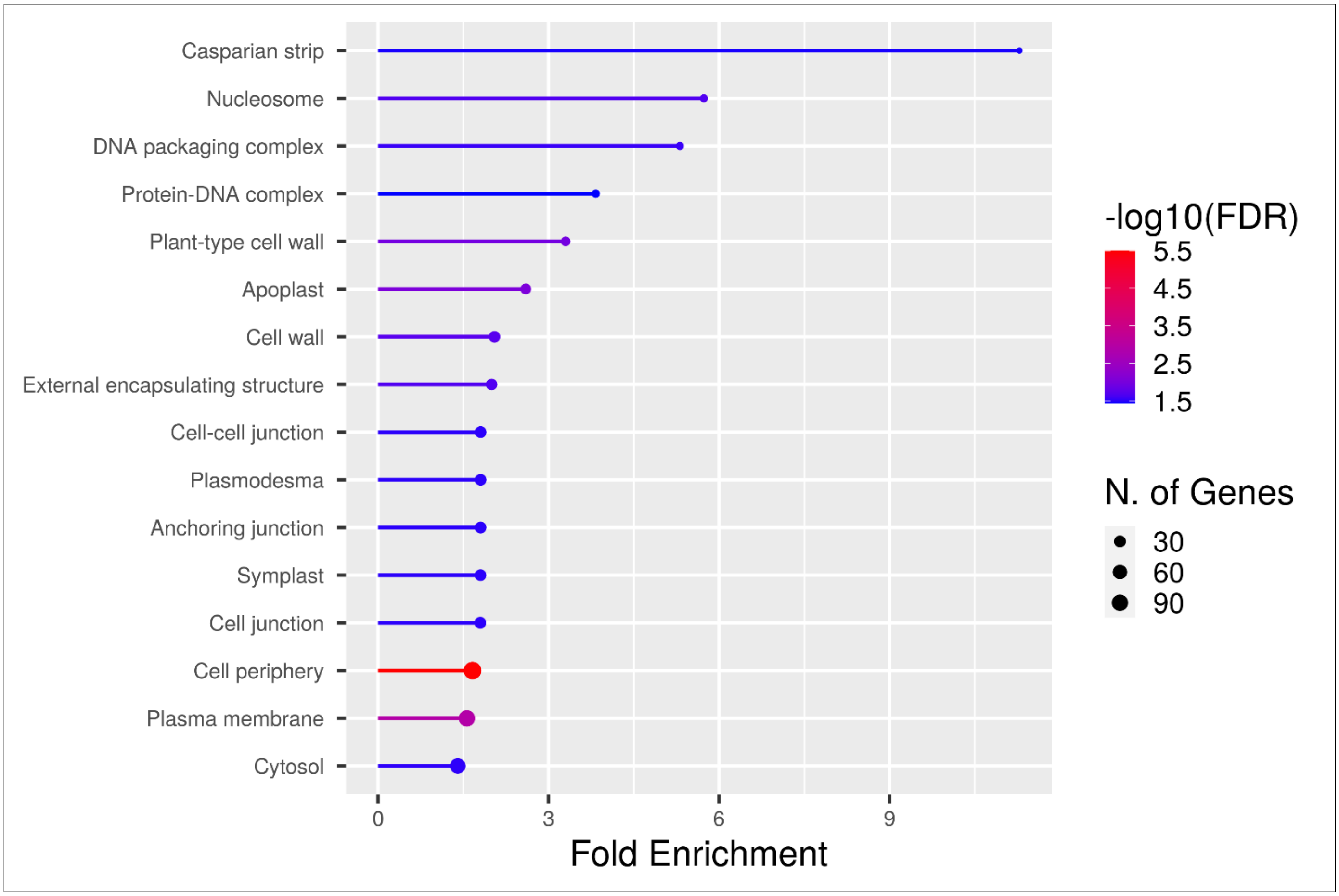

Figure S24 GO Cellular Component: Shade56<Sun56

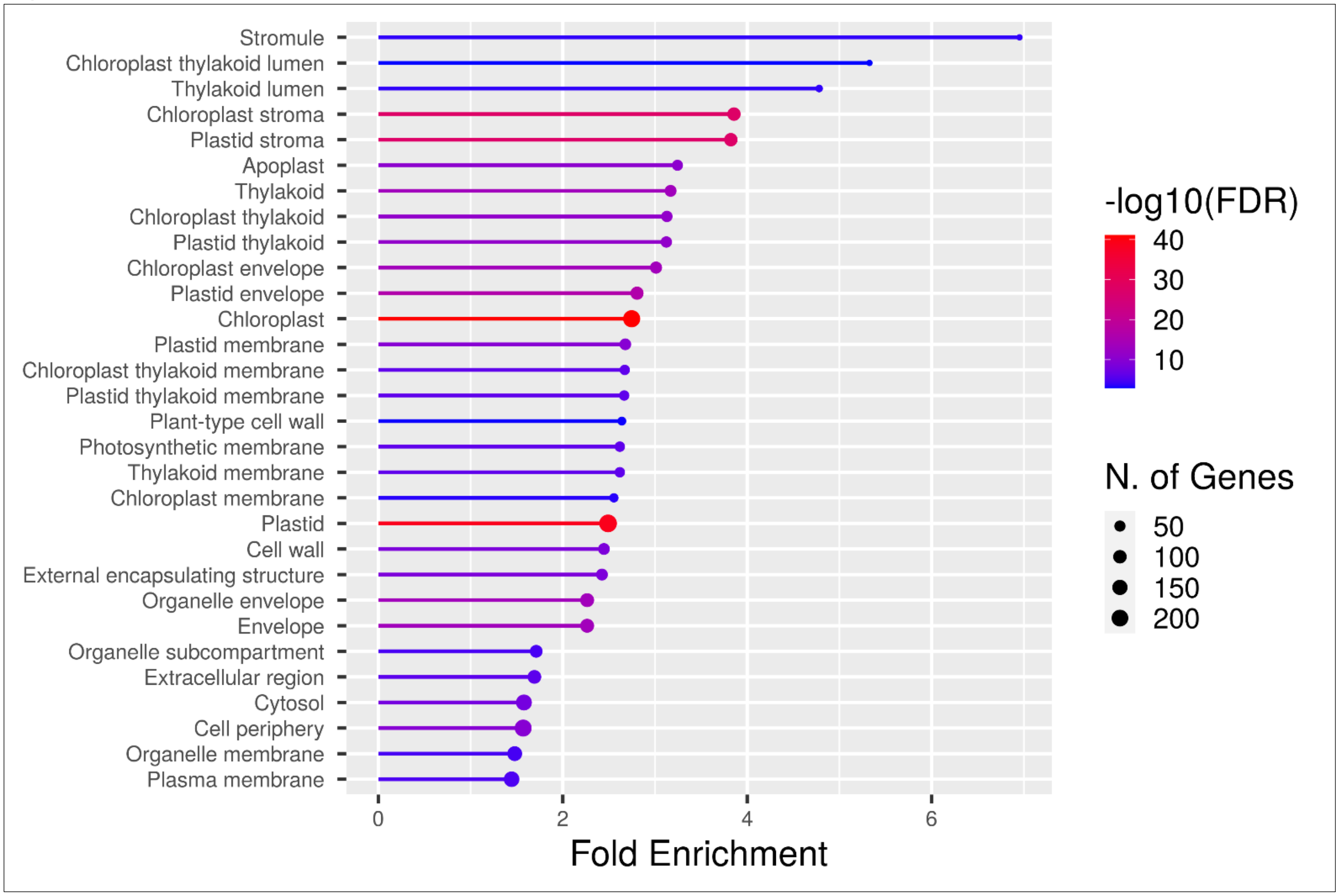

Figure S25 GO Molecular Function: Shade56>Sun56

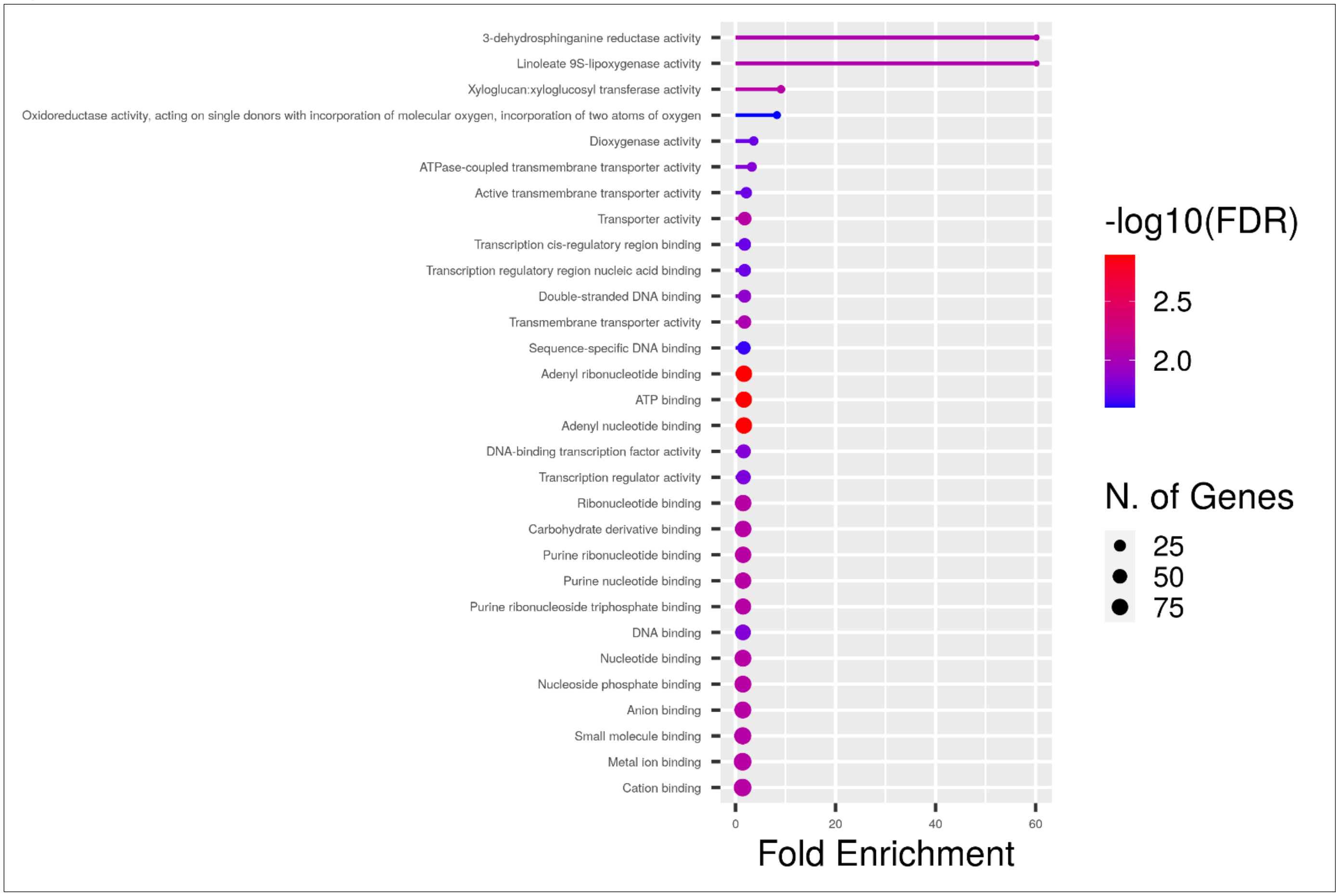

Figure S26 GO Molecular Function: Shade56<Sun56

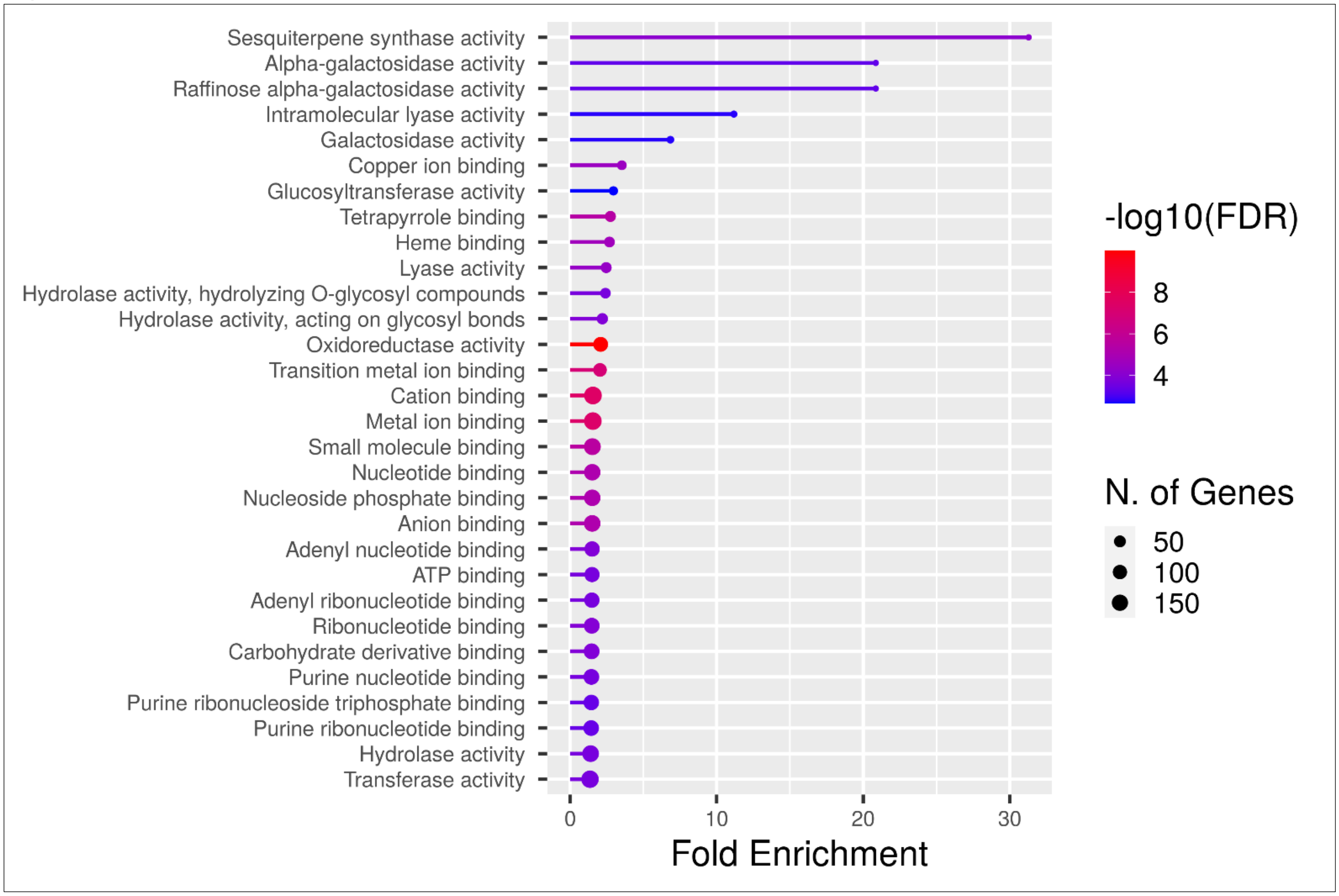

<u>Within latitude comparison in Scots pine in northern population:</u> SHADE was the treatment condition and SUN was used as control. Shade67>Sun67 denotes genes that were up-regulated under SHADE as compared to the SUN in northern population. Shade67<Sun67 denotes genes that were down-regulated under SHADE as compared to the SUN in northern population.

Figure S27 GO Biological Process: Shade67>Sun67

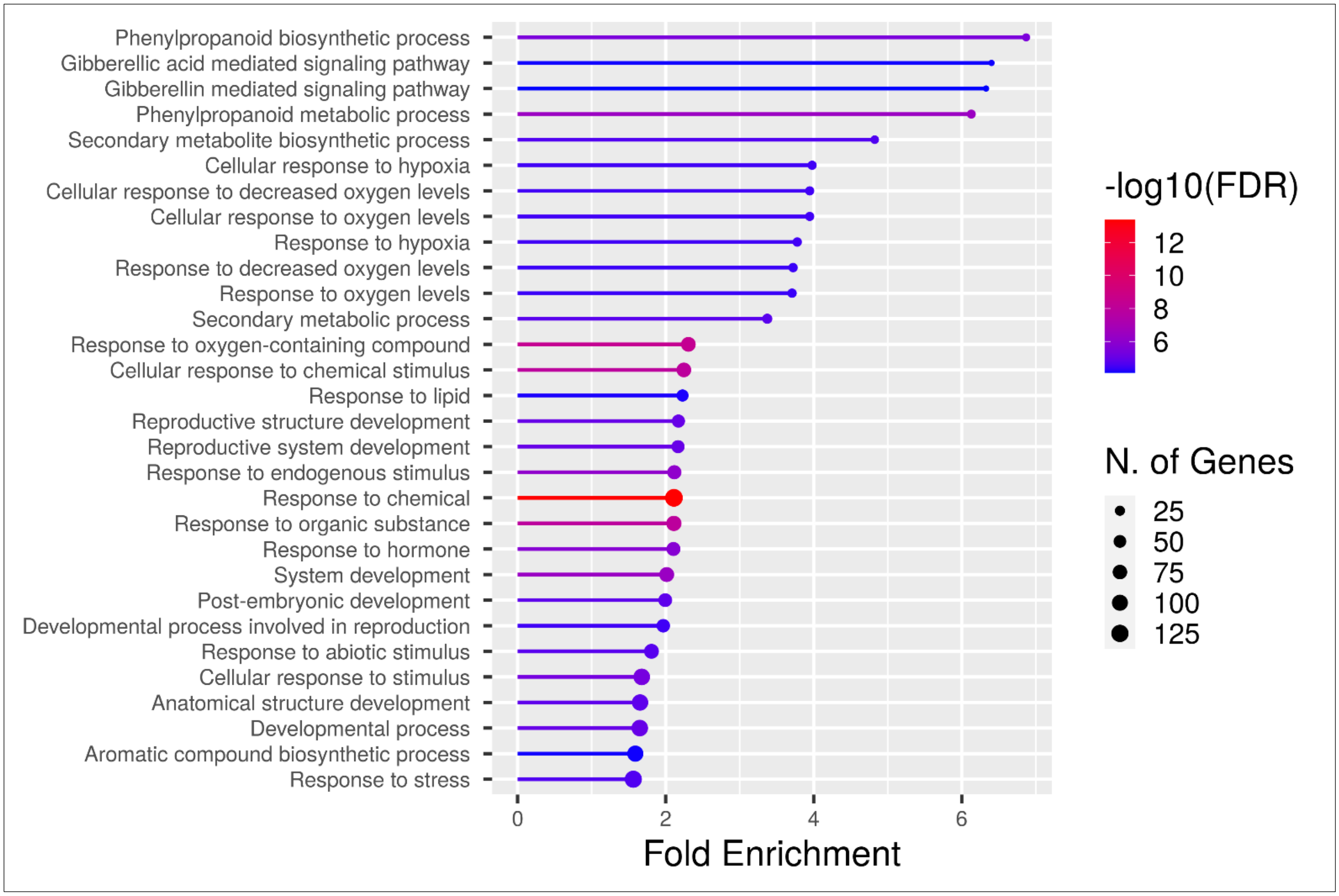

Figure S28 GO Biological Process: Shade67<Sun67

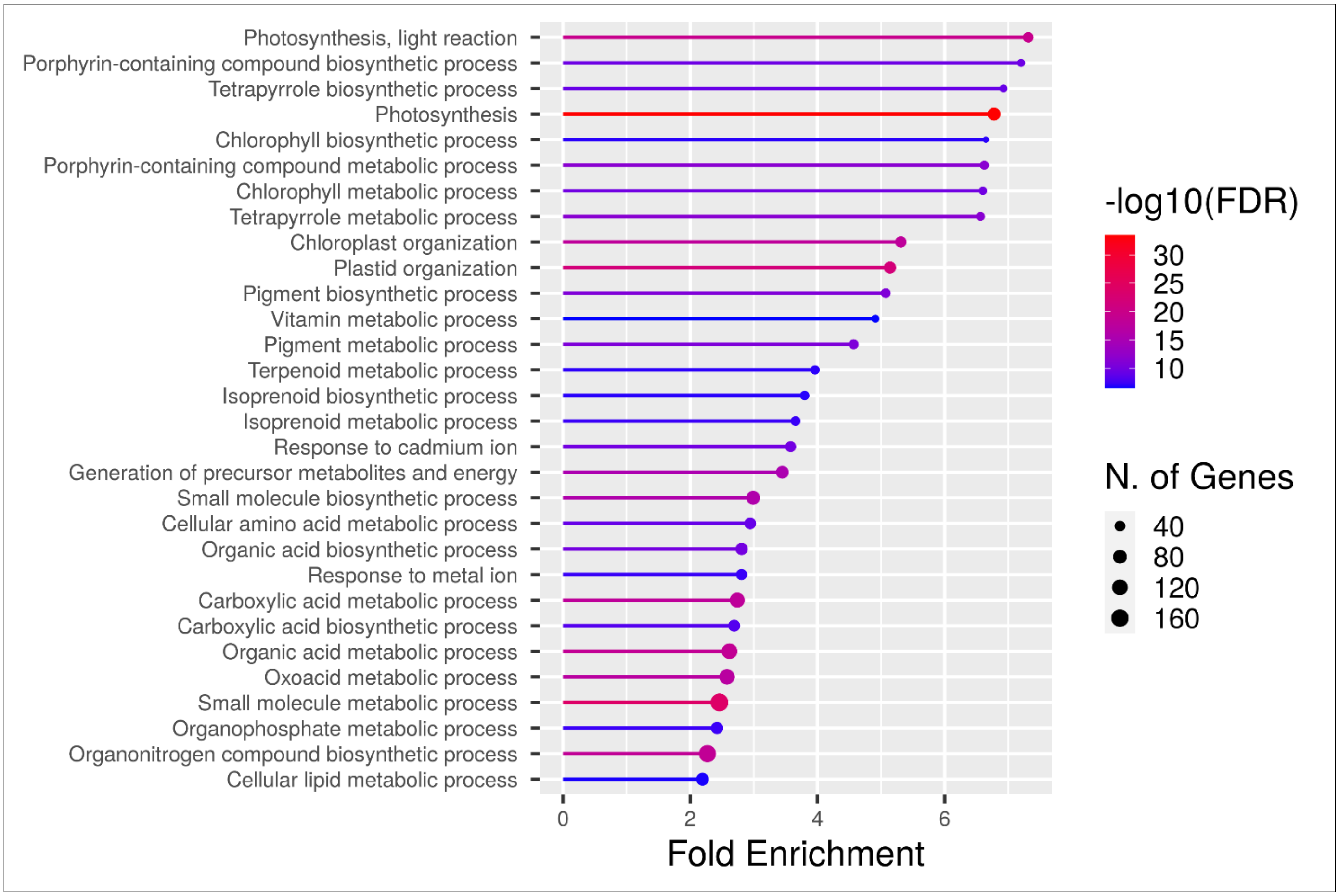

Figure S29 GO Cellular Component: Shade67>Sun67

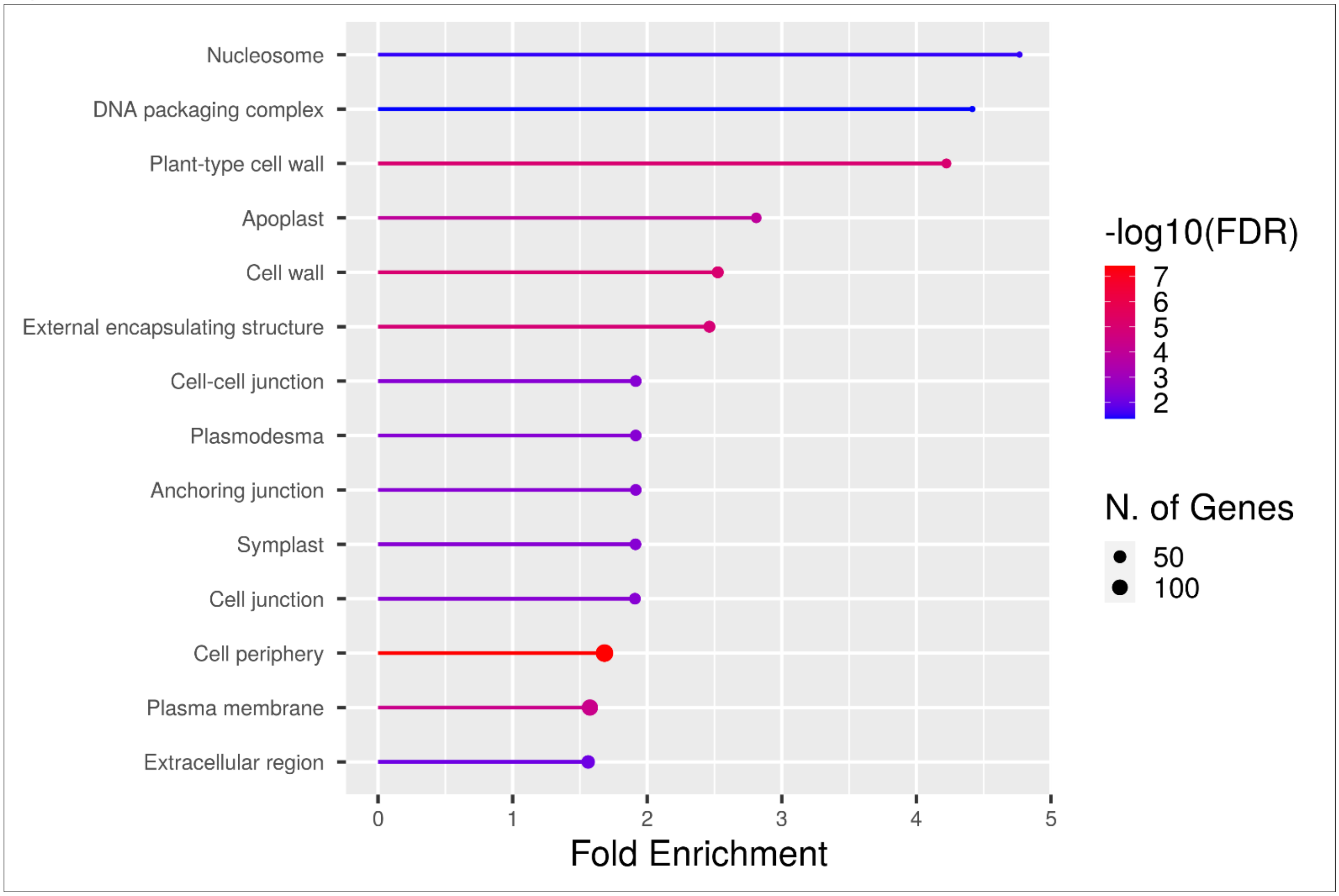

Figure S30 GO Cellular Component: Shade67<Sun67

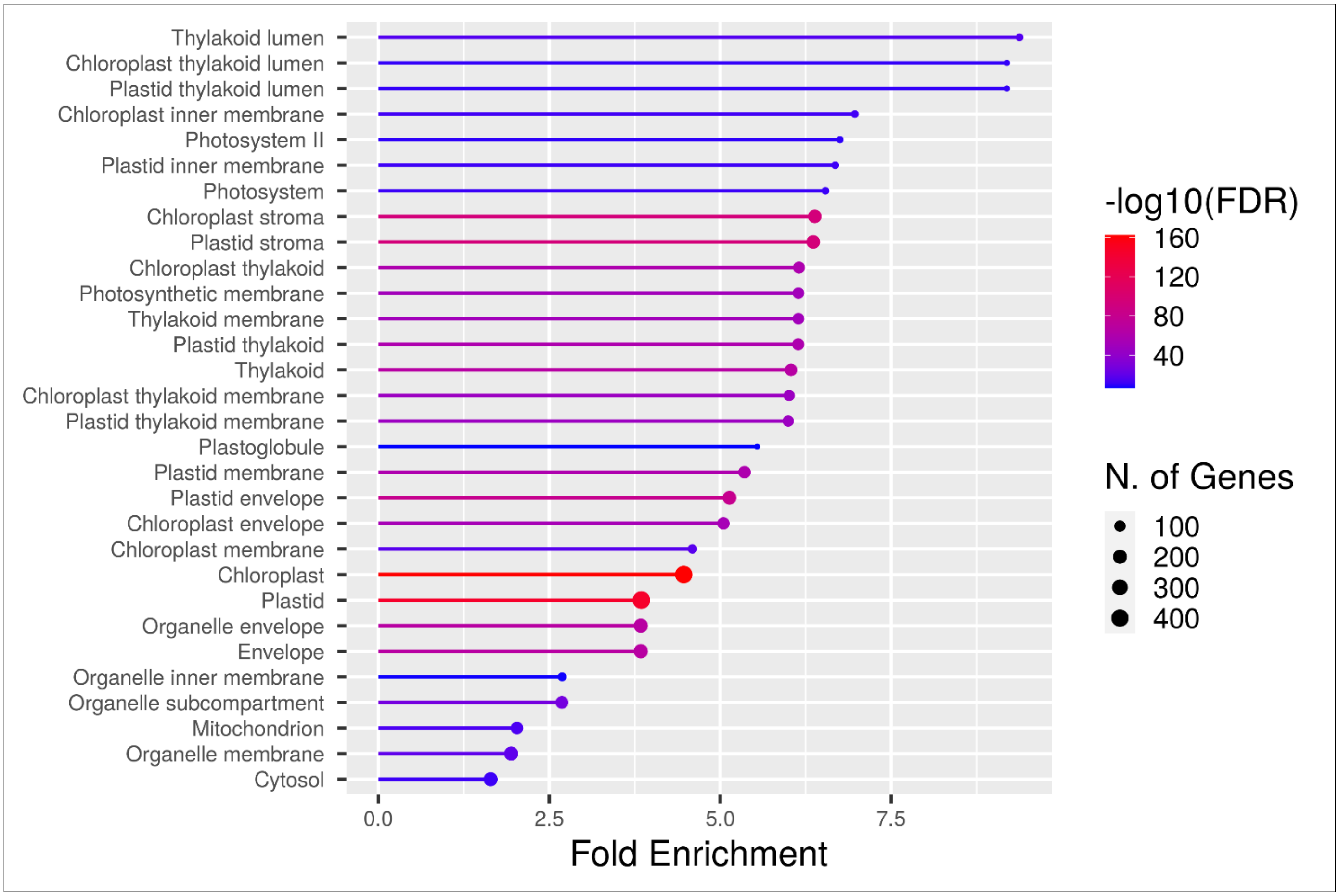

Figure S31 GO Molecular Function: Shade67>Sun67

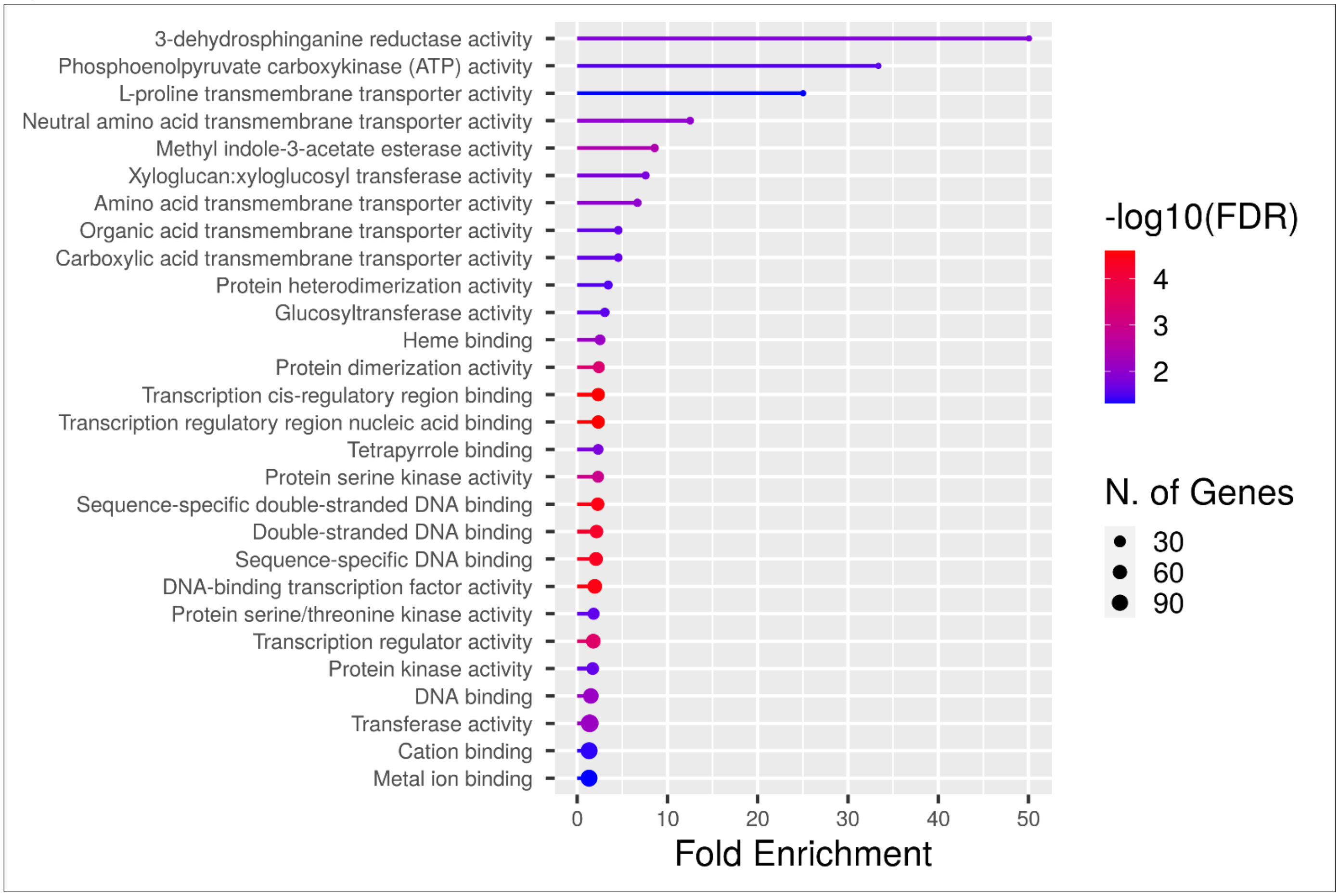

Figure S32 GO Molecular Function: Shade67<Sun67

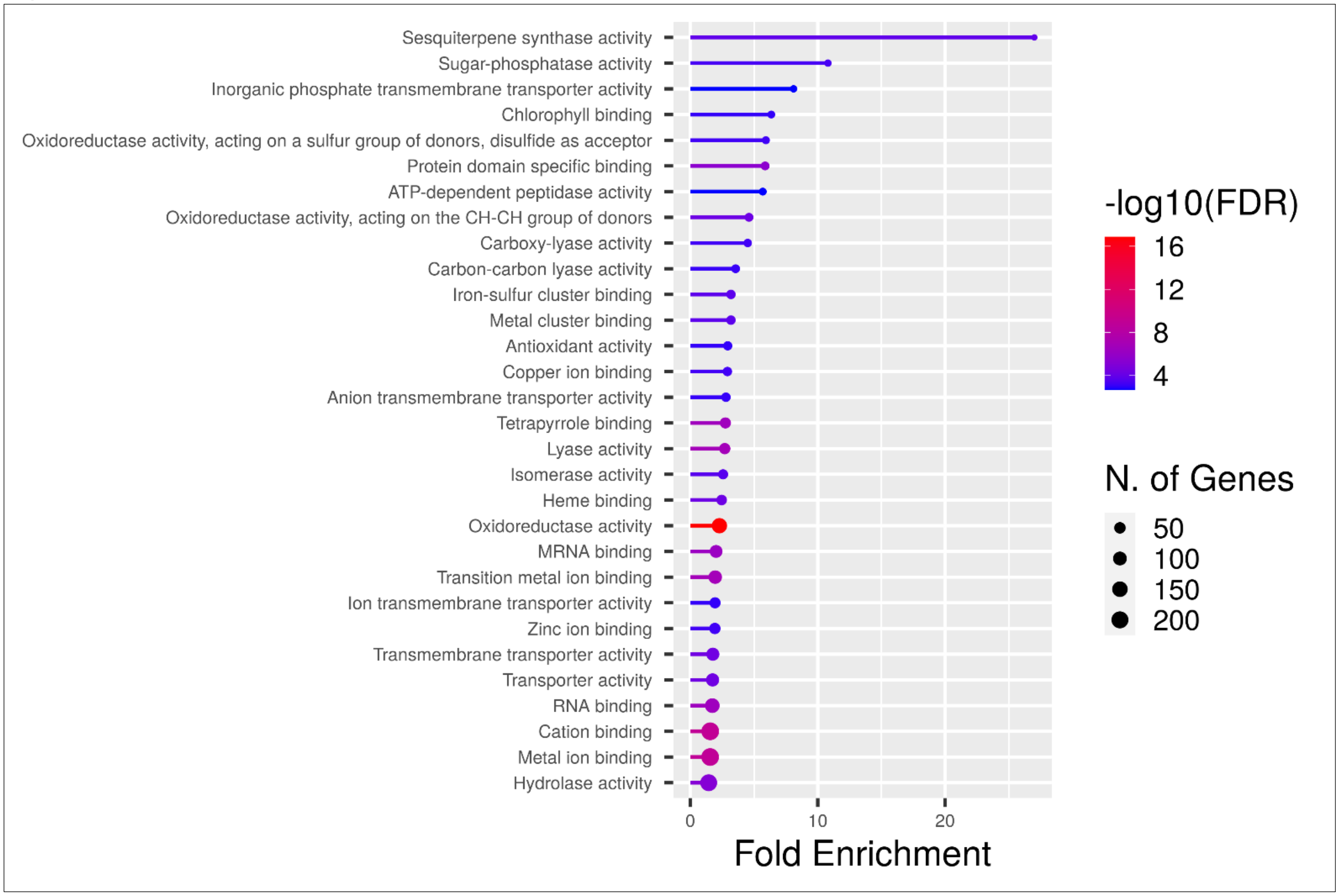

North versus south latitude comparison in Scots pine: North versus south comparison was performed using two-factor design: design=~ treatment + latitude + treatment*latitude. SHADE was the treatment condition and SUN was used as control. Shade67>Shade56 denotes genes that up-regulated under SHADE in the northern population as compared to the southern population. Shade67<Shade56 denotes genes that up-regulated under SHADE in the southern population as compared to the northern population.

Figure S33 GO Molecular Function: Shade67>Shade56

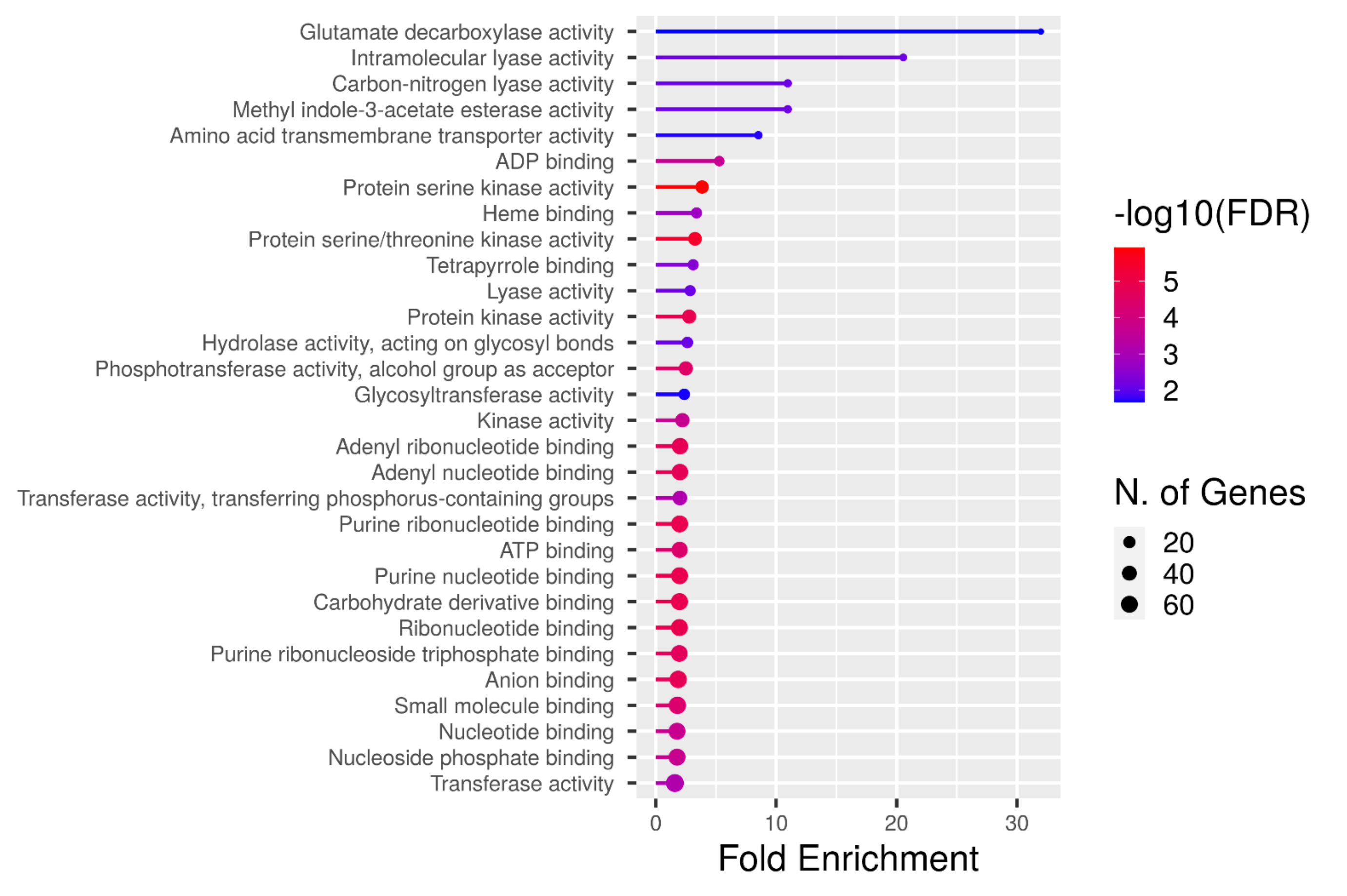

Figure S34 GO Molecular Function: Shade67< Shade56

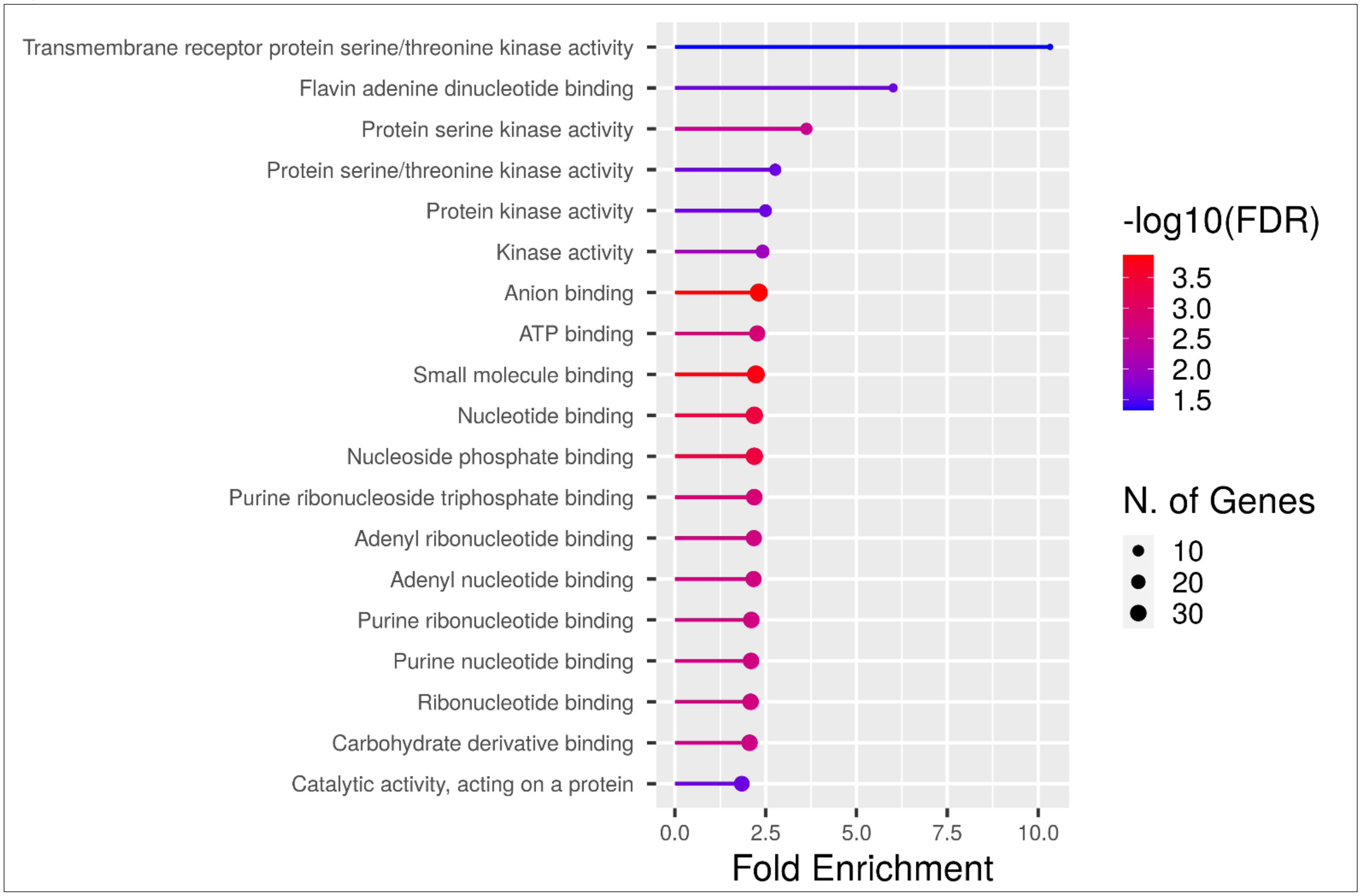

Supplement: Supplementary file 1 — Table S1 Gene expression in response to SHADE in Scots pine at latitude 56 Table S2 Gene expression in response to SHADE in Scots pine at latitude 67 Table S3 Gene expression in response to SHADE in Scots pine ‐ latitude 67 versus latitude 56 (north versus south) Figure S1 Local light conditions in Sweden throughout the year. Figure S2 Venn diagram of the differentially expressed genes under SUN and SHADE for the within latitude comparison in the southern and northern Scots pine population, respectively. Figures S3–S20 Pie charts for functional categorization by annotation (GO Biological Process, GO Cellular Component and GO Molecular Function) for the differentially regulated genes for respective treatments and comparisons in Scots pine. Figures S21–S34 Gene ontology enrichment analysis for the differentially regulated genes for respective treatments and comparisons in Scots pine (GO Biological Process, GO Cellular Component and GO Molecular Function). [file PPL-174-0-s001.pdf]
